# Range‐constrained co‐occurrence simulation reveals little niche partitioning among rock‐dwelling *Montenegrina* land snails (Gastropoda: Clausiliidae)

**DOI:** 10.1111/jbi.13220

**Published:** 2018-04-16

**Authors:** Zoltán Fehér, Katharina Mason, Miklós Szekeres, Elisabeth Haring, Sonja Bamberger, Barna Páll‐Gergely, Péter Sólymos

**Affiliations:** ^1^ Central Research Laboratories Natural History Museum Vienna Vienna Austria; ^2^ 3rd Zoology Department Natural History Museum Vienna Vienna Austria; ^3^ Department of Zoology Hungarian Natural History Museum Budapest Hungary; ^4^ Department of Integrative Zoology University of Vienna Vienna Austria; ^5^ Institute of Plant Biology Biological Research Centre of the Hungarian Academy of Sciences Szeged Hungary; ^6^ Plant Protection Institute Centre for Agricultural Research Hungarian Academy of Sciences Budapest Hungary; ^7^ Department of Biological Sciences University of Alberta Edmonton Alberta Canada

**Keywords:** allopatric distribution, coexistence, competitive exclusion, distribution modelling, geographic range overlap, non‐adaptive speciation, probabilistic null model

## Abstract

**Aim:**

Taxon co‐occurrence analysis is commonly used in ecology, but it has not been applied to range‐wide distribution data of partly allopatric taxa because existing methods cannot differentiate between distribution‐related effects and taxon interactions. Our first aim was to develop a taxon co‐occurrence analysis method that is also capable of taking into account the effect of species ranges and can handle faunistic records from museum databases or biodiversity inventories. Our second aim was to test the independence of taxon co‐occurrences of rock‐dwelling gastropods at different taxonomic levels, with a special focus on the Clausiliidae subfamily Alopiinae, and in particular the genus *Montenegrina*.

**Location:**

Balkan Peninsula in south‐eastern Europe (46N–36N, 13.5E–28E).

**Methods:**

We introduced a taxon‐specific metric that characterizes the occurrence probability at a given location. This probability was calculated as a distance‐weighted mean of the taxon's presence and absence records at all sites. We applied corrections to account for the biases introduced by varying sampling intensity in our dataset. Then we used probabilistic null‐models to simulate taxon distributions under the null hypothesis of no taxon interactions and calculated pairwise and cumulated co‐occurrences. Independence of taxon occurrences was tested by comparing observed co‐occurrences to simulated values.

**Results:**

We observed significantly fewer co‐occurrences among species and intra‐generic lineages of *Montenegrina* than expected under the assumption of no taxon interaction.

**Main conclusions:**

Fewer than expected co‐occurrences among species and intra‐generic clades indicate that species divergence preceded niche partitioning. This suggests a primary role of non‐adaptive processes in the speciation of rock‐dwelling gastropods. The method can account for the effects of distributional constraints in range‐wide datasets, making it suitable for testing ecological, biogeographical, or evolutionary hypotheses where interactions of partly allopatric taxa are in question.

## 1 INTRODUCTION

Speciation is often classified as adaptive or non‐adaptive. It is strictly adaptive when the evolution of a new species is triggered by adaptation to a new niche. This predominantly in situ process is characterized by descendant sister species remaining in sympatry and showing apparent differences in their habitat preferences. Radiations in ancient lakes are prime examples of such “strong” adaptations (Schön & Martens, 2004). The majority of speciation events is allopatric (Coyne & Orr, 2004; Turelli, Barton, & Coyne, 2001), but an increasing number of studies suggests that sympatric speciation, when ranges of sister taxa overlap, is probably less exceptional than previously thought (Bolnick & Fitzpatrick, 2007; Li et al., 2016). In allopatry, sister taxa possess non‐overlapping distribution areas, and their genetic differentiation follows geographical partitioning of the ancestral species’ original range. Divergence is initiated by non‐adaptive processes (e.g. drift) and, even if descendants adapt to distinct habitats, that is a consequence, rather than cause of the speciation (Rundell & Price, 2009). Therefore this process is often termed as “weak” adaptation (Knox, 2004). Some authors claim that allopatric speciation is driven mostly or entirely by non‐adaptive factors (Gittenberger, 1991, 2004; Wilke, Benke, Brändle, Albrecht, & Bichain, 2010), but most speciation events can be best explained by a combination of adaptive and non‐adaptive forces (Dieckmann, Metz, Doebeli, & Tautz, 2004; Olson & Arroyo‐Santos, 2009). Therefore, rather than polarizing the question whether speciation is adaptive or non‐adaptive, we instead ask to what extent it is adaptive or non‐adaptive.

Niche differentiation can be the cause or consequence of speciation: it precedes speciation in “strong” (adaptive) cases, but not necessarily in “weak” (non‐adaptive) cases. We can assume that the less adaptive the speciation process is, the slower is the niche partitioning over time. Therefore, a comparative study of phylogenetic vs. niche divergence can provide indirect evidence for the role and relative significance of the adaptive and non‐adaptive mechanisms in a taxon's evolution (Losos, 2008; Losos & Mahler, 2010). However, there are some shortfalls in implementing this seemingly simple idea in practice. Although there are methods for quantifying niche differences of species coexisting within the same community (Godoy, Kraft, & Levine, 2014), they are not applicable to allopatric species. In allopatry, habitat descriptors and other environmental factors might be used to describe niches (McCormack, Zellmer, & Knowles, 2010). But even if habitat and climate are well‐characterized, they provide little clue about the niche itself because similar habitat preferences of sister taxa cannot be seen as insurmountable evidence for their niche overlap, nor can slight differences in habitat preferences be taken as proof of niche differences (Olson & Arroyo‐Santos, 2015; Soberón, 2007).

When Gittenberger (1991, 2004) considered the rock‐dwelling gastropods (e.g. *Albinaria*) as ideal examples of non‐adaptive radiations, he not only argued that their habitats are similar, but also claimed that there are fewer than expected known cases where more than one *Albinaria* species co‐occur. A practical way to test this hypothesis could be obtaining information indirectly about niche segregation by studying co‐occurrence patterns (Pianka, 2011). However, up to now Gittenberger's field experience‐based assumption remained untested.

This prompted us to investigate co‐occurrence patterns of rock‐dwelling gastropods and to test the hypothesis that observed co‐occurrences of rock‐dwelling gastropod congeners are less frequent in nature than expected under random distribution (Gittenberger, 1991, 2004). In accordance with the competitive exclusion principle (Hardin, 1960), we started with the assumption that frequent co‐existence of two species is an indirect indication that their niches are not identical, otherwise one of them would have excluded the other. On the same basis, no or fewer than expected co‐occurrence of two sympatric species indicates overlap of their niches. We compared not only pairs of species but also those of higher taxa at different stages of taxonomic/phylogenetic relatedness (Godoy et al., 2014). Our goal with this was to identify which was the likely phylogenetic level in their divergence at which niche segregation happened and, hence, to provide indirect information on the significance of adaptation in the process of speciation. As a model system, we chose gastropods native to rocky habitats in the Balkan Peninsula, and primarily the species‐rich door snail genus *Montenegrina* Boettger, 1877. For the phylogenetic perspective we tested co‐occurrences at different levels of taxonomic relatedness: at the genus level within *Montenegrina* (divided into morphology‐based species, as well as intra‐generic clades of the mitochondrial tree), at the subfamily level within the door snail subfamily Alopiinae, at the family level within door snails, at the subclass level within pulmonate gastropods, and at the class level between pulmonate and caenogastropod snails. We also present a methodological framework that is capable of simulating range‐wide occurrence patterns of multiple species with partially overlapping ranges and data obtained by spatially varying survey effort.

## 2 MATERIAL AND METHODS

### 2.1 The study system

**Figure 1:**
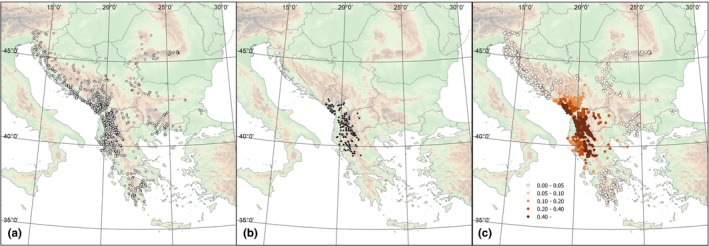
Study area with (a) locations of the studied sites (limited to limestone rock habitats) (b) presence of data of the focal study taxon, genus *Montenegrina* (Table S1.4) and (c) spatial distribution of occurrence probability (*OP*) values for *Montenegrina*. *OP* values were calculated by the “uncorrected” model and the following parameters: *k *=* *3, *d*_0_ **=* *30 km [Colour figure can be viewed at http://wileyonlinelibrary.com]

The main part of the analyses below the genus level was carried out with members of the obligate rock‐dwelling door snail genus *Montenegrina*. Fehér and Szekeres (2016) distinguished 29 species with 106 subspecies based mainly on shell morphology. *Montenegrina* belongs to the same subfamily as *Albinaria* and has similar habitat preferences, but its smaller range allows more comprehensive sampling. The distribution area in the north‐western part of the Balkan Peninsula includes approximately 400 known localities (Figure 1). Mosaical occurrence of the limestone base rock and the preferred habitats (i.e. large bare rock surfaces, rocky woodlands, rocky grasslands, gorges, etc.) is reflected by the insular distribution of *Montenegrina* populations. The species with the largest range is *M. subcristata* (Pfeiffer, 1848), represented by nearly 100 known populations, whereas the ranges of some species (*M. apfelbecki* [Sturany, 1907], *M. haringae* Fehér & Szekeres, 2016; *M. chiasma* Nordsieck, 1972, *M. zilchi* Nordsieck, 1974) may be restricted to single sites. As characteristic for obligate rock‐dwelling gastropods in general, their active dispersal is severely limited. Colonizing new habitat patches or migrating between populations is possible only by jump dispersals, which are relatively rare and distance‐dependent events as shown by molecular evidence (Uit de Weerd, Piel, & Gittenberger, 2004; Uit de Weerd, Schneider, & Gittenberger, 2005). Hence, closely related *Montenegrina* populations/subspecies/species are often found spatially close to each other. The distribution patterns are not entirely allopatric: species ranges partly overlap, and there are at least 10 known cases when two *Montenegrina* species co‐occur at the same locality (Fehér & Szekeres, 2016). *Montenegrina* species, like most of the rock‐dwelling door snails, live on the open rock surface and are relatively abundant locally. They are relatively easy to find by hand collecting (at least the empty shells at the bases of the rocks) and even cursory sampling reliably indicates their actual presence or absence at a locality, thus minimizing false absences at survey locations.

Other land snail taxa were also included in the analyses (see Table S1.1 in the Supporting Information). Several of these, including other genera in the subfamily Alopiinae, are obligate rock‐dwellers like *Montenegrina*. Others can be found in the same rocky habitats as *Montenegrina*, but without being obligate rock‐dwellers, whereas others inhabit the superficial underground compartments around the rocks (Camacho, 1992: 65).

### 2.2 Phylogenetic reconstruction of *Montenegrina*

DNA analyses were carried out using samples from 291 of the 386 *Montenegrina* populations (441 specimens, representing 103 subspecies and 27 species). Most of the material was collected after 2003 and stored in ethanol. Table S1.2 gives the geographical origin and taxonomic identity of the samples used in the phylogenetic reconstruction, as well as the DNA isolation, polymerase chain reaction (PCR) and sequencing methods used. Phylogenetic relationships were inferred from partial sequences of the mitochondrial *cytochrome c oxidase subunit I* (*COI*, 655 bp), the *16S rRNA* (845–866 bp), and the *12S rRNA* (677–713 bp) genes. Sequences are deposited in GenBank (KU307511–KU308245).

*COI* could be unambiguously aligned (655 bp), whereas *16S* and *12S* sequences were aligned with the online version of MAFFT (Katoh & Standley, 2013, http://mafft.cbrc.jp/alignment/software/). The G‐INS‐i iterative refinement algorithm was used with the following settings: gap opening penalty* *=* *1.53, offset value* *=* *0.123 and “leave gappy regions.” From the raw *16S* (960 bp) and *12S* (757 bp) alignments questionably aligned positions were eliminated with GBLOCKS 0.91b (Castresana, 2000), applying all “less stringent” block selection parameters. The lengths of *16S* and *12S* alignments after trimming were 755 and 659 bp, respectively. Thereafter, the three alignments were concatenated.

partitionfinder 1.1.1 (Lanfear, Calcott, Ho, & Guindon, 2012) was used to select the appropriate partitioning scheme and models of sequence evolution. The list of nucleotide evolution models was restricted to those available in the programs used for further analyses. The following 5‐partitions scheme and models were used: *COI* 1st codon position: GTR+I+G, *COI* 2nd codon position: HKY+I, *COI* 3rd codon position: GTR+G, *16S*: GTR+I+G and *12S*: GTR+I+G).

An unconstrained Bayesian tree was calculated using mrbayes 3.2.1 (Ronquist et al., 2012) with the following parameters: a four‐chain (one cold, three heated; *T* = 0.2) Metropolis‐coupled Markov chain Monte Carlo (MCMC) analysis, run for 5 × 10^6^ generations; trees were sampled every 100 generation. The first 20% of trees were discarded as burnin and a 50% majority rule consensus tree was calculated from the remaining trees. Maximum likelihood (ML) analysis was performed by garli 2.0 (Zwickl, 2006). We selected the tree with the best ML score after 20 independent runs with random starting positions, and nodal support was assessed by 200 bootstrap pseudo‐replications.

**Figure 2:**
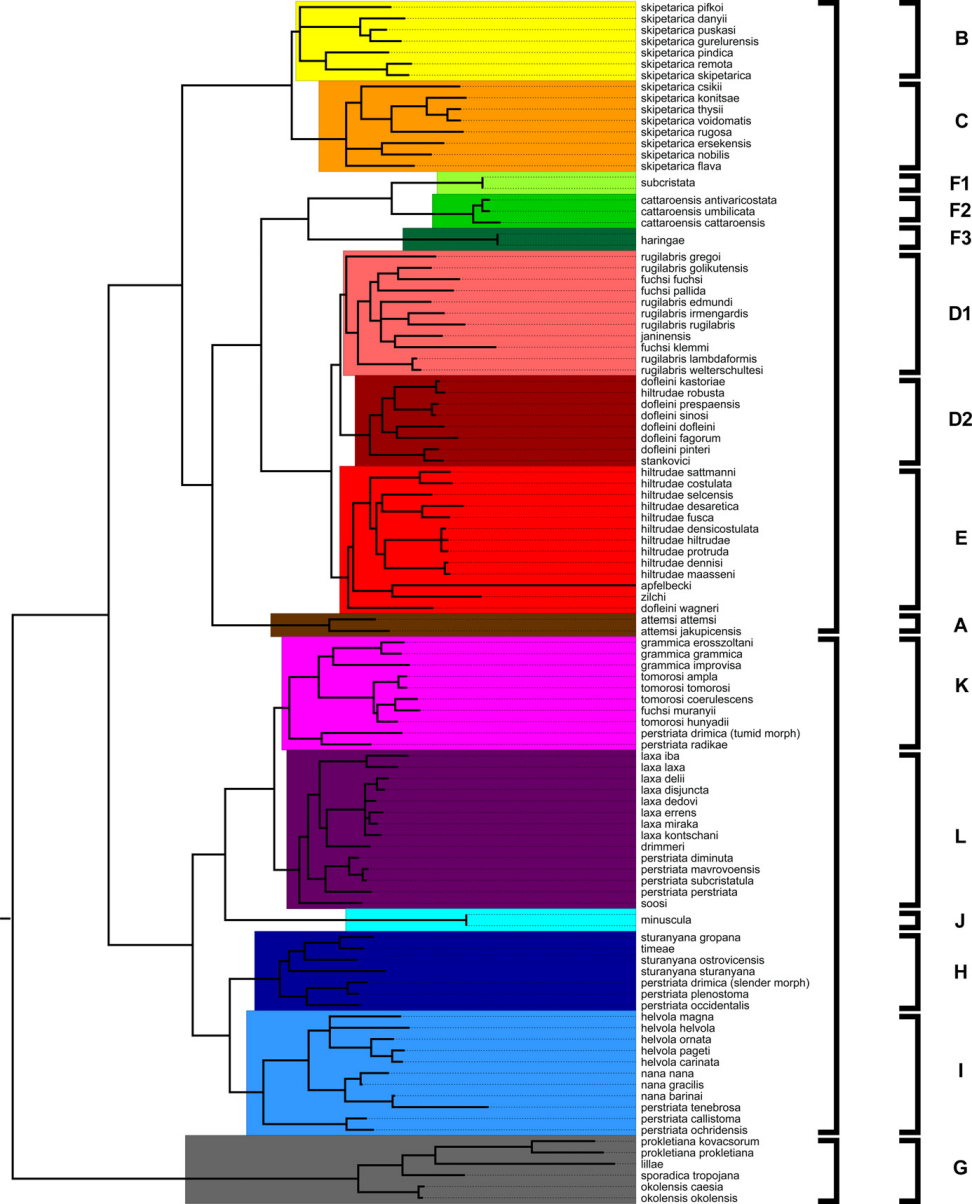
Phylogenetic tree of *Montenegrina*, based on Bayesian inference analysis of mitochondrial (*COI*,* 16S rRNA* and *12S rRNA*) genes. Clade assignments correspond to those of Tables S1.2 and S1.3. The tree was rooted using *Vallatia vallata* (Mousson, 1859) as an outgroup (not illustrated) [Colour figure can be viewed at http://wileyonlinelibrary.com]

The mitochondrial phylogeny of *Montenegrina* was mostly, but not entirely congruent with the morphology‐based system of the genus (Table S1.3). As there are several reported cases of this phenomenon, including related genera in the subfamily Alopiinae (Giokas, 2000; Uit de Weerd & Gittenberger, 2004), this finding was not surprising. Interpreting discrepancies between the mitochondrial tree and the morphology‐based system was beyond the scope of this study. The mitochondrial tree was used only for obtaining a two‐level division of *Montenegrina* into three groups and 15 subgroups according to the tree topology (Figure 2 and Figure S1.1 in Supplementary Information). Populations without available DNA data (95 out of the 386) were allocated to clades according to the positions of the morphologically related populations/consubspecifics.

### 2.3 Distribution data

Taxon distribution data were taken from databases of the Mollusca collections of the Hungarian Natural History Museum (HNHM) and the Natural History Museum Vienna (NHMW). These records originated from opportunistic faunal samplings by various collectors with different sampling effort (i.e. the sites were sampled for differing durations, with a different number of visits or collectors, under different weather conditions and by different collecting methods). We only considered records from georeferenced locations of known vegetation and base rock type. During the fieldwork, hand collecting usually covered a transect of a few hundred metres. Soil/litter samples were also taken from this area. Individuals of the same species collected in such a way are usually kept within the same museum lots under the same locality names. Accordingly, data were merged from surveys done at the same location at different dates, or when geographical coordinates differed only by a few hundred meters.

The study area was delimited around the actual range of *Montenegrina* with an approximately three times larger radius (Figure 1a,b). This region comprised the entire Balkan Peninsula, including the Julian Alps (Slovenia) in the north‐west, the Southern Carpathians (Romania) in the north‐east, the Stara and Strandzha Mountains (Bulgaria) in the east, and the Peloponnese (Greece) in the south. The resulting raw dataset contained taxa from 2500 localities, 1649 of which were different kinds of limestone habitats where *Montenegrina* and other obligate rock‐dwelling gastropods could potentially occur (Fehér & Szekeres, 2016). These habitat types are herein referred to as “potentially suitable habitats.” In order to keep habitat‐related factors (habitat suitability) fixed, and thus allow focusing on range‐related issues, only these 1649 sites were used in further analyses. Their uneven geographical distribution is due partly to the uneven distribution of limestone areas, and partly to the uneven sampling activity within the study area (Figure 1a).

Distribution records were arranged into presence–absence data matrices (*Y* matrices), where rows are sites, columns are taxa, and a *Y*_*it*_ matrix element takes 1 or 0, depending on whether taxon *t* was detected at the site *i*. We made three different *Y* matrices, depending on how the focal study taxon *Montenegrina* was subdivided (*Y1–Y3*, Tables S1.4–6). In all three matrices, other taxa were consistently binned into 46 groups as follows: other taxa in the subfamily Alopiinae were distinguished at the genus level (16 genera); other pulmonate land snails, including those of other clausiliid subfamilies, were distinguished at the family level (29 families); and prosobranch land snails of the superorder Caenogastropoda were treated as a single group. In the *Y1* matrix, *Montenegrina* records were merged at the genus level (Table S1.4). In the *Y2* matrix *Montenegrina* records were divided into 15 subgeneric clades based on the mitochondrial phylogeny (Tables S1.5). Finally, in the *Y3* matrix, *Montenegrina* observations were pooled at the species level, according to the recent shell morphology‐based revision (Table S1.6). This dataset included 27 of the 29 known *Montenegrina* species (herein referred to as morphospecies).

Taxa of the *Y1–Y3* matrices form 1081, 1830 and 2628 taxon pairs respectively. For some of the further analyses these taxon pairs were categorized by the taxonomic/phylogenetic relatedness of their members, particularly whether they are related at the class, subclass, family, subfamily, genus or the intra‐generic clade level (Figure S1.1).

### 2.4 Definition of taxon ranges

We defined taxon ranges as a continuous spatial utilization distribution based on occurrence data for each species, rather than as (binary) range maps. Assuming a presence–absence dataset of T taxa at I sites, we introduced a taxon‐ and site‐specific measure denoted as *OP*_*it*_ (occurrence probability of taxon *t* at site *i*). We used presence–absence status at each site and a spatial weight matrix (*W*) to calculate *OP*. Spatial weights determined the extent that two sites contribute to each other's *OP* metrics, depending on their pairwise geographical distances, and the number of *W* values belonging to each site is equal to the number of sites involved in the study (= the number of rows in the *Y* matrix). The spatial weight between any two sites (*i*,* j*) was defined as a logistic function:

(1) $$W_{\mathrm{ij}}=\frac{1}{1+e^{k(lgd_{\mathrm{ij}}-lgd_{0})}}$$

Where *d*_*ij*_ is the geographical distance between the two sites (in km), *d*_0_ defines the distance where the weight is 0.5 and *k* determines the steepness of the distance decay function (see Figure S2.2). In order to get rid of *d*_*ii*_ **=* *0 values, and thus errors by taking the logarithm of zero, 0.1 km was added to all pairwise distances. Considering the geographical distances between our study sites, as well as the applied *k* and *d*_0_ values, this modification had no significant impact on the results, as indicated by sensitivity analyses that we performed. Thus, *W*_*ij*_ can take a value between 0 and 1 and, unlike in conventional spatial weight matrices (e.g. Murayama, 2012), *W*_*ii*_ was defined as 1.

Based on the *W*_*ij*_ values defined above, the *OP* metric of a given *t* taxon at site *i* is given as

(2) $$OP_{\mathrm{it}}=\frac{\sum_{j=1}^{n}W_{\mathrm{ij}}Y_{\mathrm{jt}}}{\sum_{j=1}^{n}W_{\mathrm{ij}}}$$

where the binary *Y*_*jt*_ value defines whether *t* taxon was present at site *j*. Thus, in practice, *OP*_*it*_ is the sum of *W* values of the presence sites, divided by the sum of *W* values of all sites. *OP* values were calculated for all sites for all taxa, therefore the *OP* matrix has the same dimensions as the raw dataset.

Though, to some extent, this formula takes all sites into account, the proper selection of the two constant parameters (*k* and *d*_0_) serve the purpose of avoiding too large an influence of the sites on their own probability values (undersmoothing), and also preventing a significant contribution by sites at biogeographically irrelevant distances (oversmoothing). Such smoothing is used to incorporate expert knowledge or range maps into species distribution modelling (see Merow, Wilson, & Jetz, 2017) where the level of smoothing is often empirically calibrated. During our exploration of the Balkans we have found that the probability of finding a certain rock‐dwelling gastropod species at a newly explored site depends on the distance from the nearest sites where this species is known to occur. Based on our field experience we have come to suspect that the distance decay of the probability of jump dispersal events can be described by a logistic, rather than a linear function, and the transition between the biogeographically relevant and irrelevant distances might be somewhere between 10 and 100 km. Accordingly, we tried to set *d*_0_ and *k* parameters so that neighbouring sites nearer than 10 km should make a nearly complete (*W*_*i*_ ≈ 1.0) contribution to each other's *OP* calculations, whereas those farther than 100 km apart contribute nearly zero.

To assess the sensitivity of the results to the smoothing parameter settings we tested three different *k*,* d*_0_ pairs ([5, 30], [3, 30], and [10, 50]) to find out how parameter selection influences the simulated co‐occurrences. In practical terms this meant that if the distance of two sites (*i* and *j*) is 10, 30, 50 or 75 km, the *k *=* *5, *d*_0_ **=* *30 settings result in *W*_*ij*_ ≈ 1, 0.5, 0.07 and 0.01 weights respectively. The *k *=* *10, *d*_0_ **=* *50 settings allow larger contributions of somewhat more distant sites, for example those at 30 and 50 km have >0.99 and 0.5 weights, but sites more distant than 75 km have almost as low an effect as under the *k *=* *5, *d*_0_ **=* *30 settings. The *k *=* *3, *d*_0_ **=* *30 settings provide a smoother distribution for the *W*_*ij*_ parameter, as it assigns lower weights to less distant sites (e.g. *W*_*ij*_ ≈ 0.77 at 20 km) and relatively higher weights to more distant ones (e.g. *W*_*ij*_ ≈ 0.06 at 75 km and still *W*_*ij*_ > 0.01 at 140 km) (see Appendix S2).

### 2.5 Range‐constrained co‐occurrence simulation

Presence–absence records were simulated by geographic range‐constrained random selections (simulated presence–absence tables are also denoted as Ψ matrices). The constraints were defined by matrices, calculated from the original *OP* matrix in three ways (Figure S2.3). First, in order to simulate such presence–absence tables where the total sum of presence values approximates that of the original data table, we rescaled the original *OP* matrix by multiplying all elements by the sum of the raw *Y* matrix elements and dividing by the sum of the *OP* matrix elements. Thus, the total sum of this rescaled *OP* matrix (denoted as *OP′*) is equal to the total number of presence records in the observed *Y* data table, but otherwise each element is proportional to those in the raw *OP* matrix (“uncorrected” model). Second, we initially created a vector (*n*) for the number of presence records per location by summing the rows of the *Y* dataset. Next, we randomized the order of its elements (*n′*), and then multiplied by the raw *OP* matrix. The product was finally rescaled so that the total sum of the resulting matrix is equal to the total number of presence records in the observed *Y* data table, but the elements are proportional to the products of the *OP*_*it*_ values of the corresponding site (site *i*) and the number of taxa found on a randomly selected site (“hard” corrected model). Third, in order to avoid eventual zero values in the rescaled *OP′* matrix, we modified the correction vector by adding 1 to each of its elements before multiplying that with the raw *OP* matrix (“soft” corrected model) (Figure S2.3). The “soft” correction stands in between the “uncorrected” and “hard” corrected algorithms in terms of matching the marginal distribution of the input data matrix.

Based on the rescaled *OP′* matrices, occurrence data tables (denoted as _*u*_Ψ*,*_*h*_Ψ and _*s*_Ψ for the uncorrected, the “hard” corrected and the “soft” corrected simulations) were simulated based on unequal probabilities of selection (i.e. a doubling of an $OP_{\mathrm{it}}^{\prime }$ value provides twice the chance for a given taxon in a given site to be selected in each selection round). During the simulation, random factors acted independently in each round, but under the same range‐constraints that were defined by the site‐ and taxon‐specific occurrence probability values. Simulated co‐occurrences (μ) were calculated from Ψ matrices as the number of sites where both taxa were present. These steps of the distribution simulation and co‐ocurrence calculation were repeated 1000 times and minimum, maximum and mean values were calculated for each of the simulated pairwise co‐occurrences. It is important to note that for the “hard” and “soft” corrected models each simulation round started from a newly randomized *n′* vector.

An R script was used to calculate *OP*,* OP′* and corrected *OP′* matrices to make randomizations, to simulate Ψ tables and to calculate co‐occurrences. We used the “nullmodel” and “oecosimu” functions in the “vegan” R extension package (Oksanen et al., 2016) to perform the Monte Carlo simulations for our null‐model analysis. The R script with a worked example is available at the Zenodo Digital Repository (https://doi.org/10.5281/zenodo.1124944). For a two‐sided null hypothesis testing we used the range of the simulated values (minimum to maximum). Although both more than expected and fewer than expected co‐occurrences were recorded, we were mainly interested in the latter, as from the point of our research question the distinction between dissociation and random distribution was of primary importance.

For a better visualization and better comparison of the results, the observed pairwise co‐occurrence counts (*m*) were rescaled relative to the range of the simulated co‐occurrence values (μ)

(3) $$m^{\prime }=\left\{\begin{array}{cc} \frac{\overset{¯}{\mu }-m}{\overset{¯}{\mu }-\mu_{\min}} & if\;m\leq \overset{¯}{\mu }\\ \frac{m-\overset{¯}{\mu }}{\mu_{\max}-\overset{¯}{\mu }} & if\;m>\overset{¯}{\mu } \end{array}\right.$$

Hence, this rescaled value (*m′*) is equal to −1, 0 and +1 when the observed co‐occurrence count is equal to the lowest (μ_*min*_), the mean ($\overset{¯}{\mu }$), or the highest (μ_*max*_) simulated counts respectively. If the observed count is below or above the simulated range, *m′* takes a value lower than −1, or higher than +1.

Simulated pairwise co‐occurrence values of rare and/or largely non‐overlapping taxa are generally very low. In such cases the ranges of simulated values usually include zero, which makes it impossible to assess the lack of observed co‐occurrences, that is the distinction between “expected” and “fewer‐than‐expected” zero co‐occurrence values. This was the case with the subdivided *Montenegrina* data: the more clades we split them into, the more pairwise unassessable zero co‐occurrence counts were obtained. To surmount this, we cumulated pairwise co‐occurrence counts: the observed counts were summed up by the groups as outlined above (Table S1.1), and the same was done with the simulated pairwise counts after each simulation round. Means and ranges (minimum–maximum) were calculated from these cumulated counts and compared to the group sums of the observed co‐occurrences. This kind of calculation helps overcoming the problem caused by the pairwise unassessable zero values, but should be interpreted carefully because pairs of widespead taxa in many presence records may considerably influence, and eventually distort the results.

## 3 RESULTS

### 3.1 Effect of model settings and correction modes

**Figure 3:**
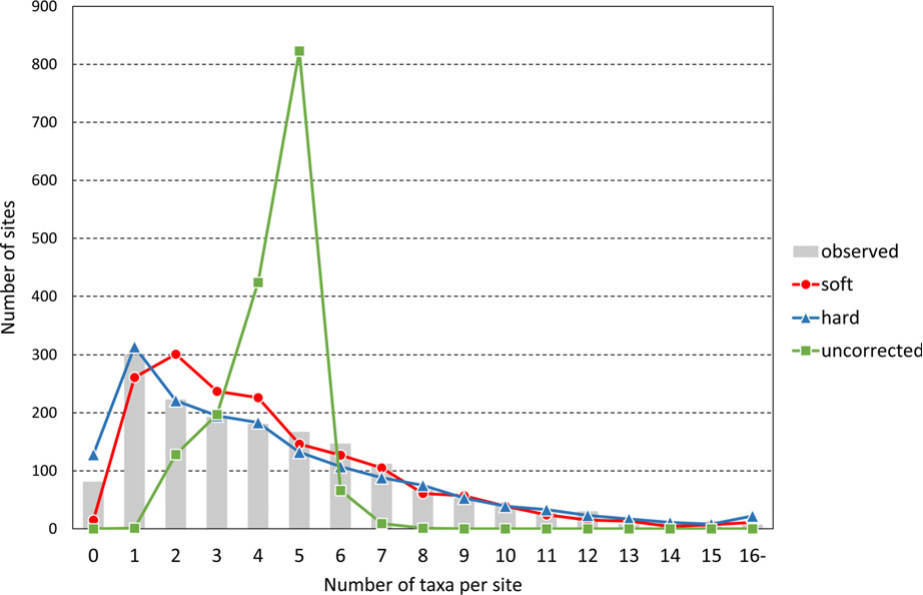
Frequency distributions of observed (bar chart) and simulated (line charts) taxa per site counts of 47 Balkan land snail taxa based on the *Y1* matrix (Table S1.4). Simulations were done with *k *=* *3 and *d*_0_ **=* *30 km smoothing parameters with “hard” (triangles), “soft” (dots) or no (squares) model correction. This is a tool for quick visual assessment of the bias in the raw data. The striking difference between the frequencies of observed taxa per site counts and those simulated without correction indicates some bias, presumably caused by uneven sampling [Colour figure can be viewed at http://wileyonlinelibrary.com]

The *Y1* matrix, in which *Montenegrina* records were merged (47 taxa × 1649 sites, Table S1.4) contained 7033 presence records altogether. Instead of a symmetric shape, with a mode near the 4.3 mean value, the frequency distribution of the taxa per site values was strikingly right‐skewed and platykurtic (the mode was at 1). Hence, the number of sites with zero to two taxa, as well as those with more than six taxa, was higher than expected under a nearly symmetrical distribution (simulated by the “uncorrected” model) assumption (Figure 3).

The total number of observed co‐occurrences was 20,031. Simulations under the “hard” correction, which were based on a taxon per site frequency distribution, yielded almost the same total number of co‐occurrences (20,200–20,219). Uncorrected models, depending on how the *d*_0_ and the *k* parameters were set, simulated 14,627–14,800 total co‐occurrences, whereas models using “soft” correction simulated total co‐occurrences in between those of the “hard” and the “uncorrected” models (18,165–18,186).

Each of the studied taxa occurred together with 2.2–12.8 other taxa (Table S1.1). Those taxa requiring more sampling effort (namely the members of Ferussaciidae, Argnidae, Cochlicopidae, Pupillidae, Valloniidae, Punctidae, Euconulidae, which comprise mainly small‐sized and/or locally rare species that often require special collecting techniques) co‐occured with more than nine other taxa on average. At the other end of the spectrum there were larger‐sized and locally frequent taxa (including most of the alopiinine genera), which are easy to collect and, therefore, are more likely to be found at cursorily sampled sites (Table S1.1).

Out of the 1081 possible taxon pair combinations of the *Y1* matrix, 360 had zero observed values. Depending on the correction modes and model settings, 628–709 of the observed non‐zero and 358–360 of the observed zero co‐occurrence values were within the simulated ranges. The number of observed pairwise co‐occurrences above or below the null‐model simulated ranges were 7–92 and 1–18, respectively (Table 1). The highest values were simulated under the “hard correction,” and the lowest ones under the “uncorrected” way of modeling. The model setting that led to the lowest co‐occurrence counts was the distance decay with steepest slope (*k *=* *5 and *d*_0_ **=* *30 km), assigning the lowest *W* values to distances higher than 70 km (Table 1). The fewest outliers were found when the *k *=* *5 and *d*_0_ **=* *30 km settings were combined with the “hard” correction. This combination of model settings resulted in the lowest higher‐than‐expected and, at the same time, the highest lower‐than‐expected values (Figure S3.4, Table 1).

**Table 1:** Summary of co‐occurrence simulations for 47 Balkan land snail taxa (*Y1* data matrix, Table S1.4). We applied nine different combinations of model corrections and smoothing parameter settings and ran 1000 simulations using each. Ranges (minimum–maximum) of the simulated co‐occurrence counts for each of the 1081 taxon pairs were compared to the observed values. On this basis, taxon pairs were categorized into four groups: observed value is less than the simulated range/observed zero value falls within the simulated range/observed non‐zero value falls within the simulated range/observed value is higher than the simulated range. Detailed outcome of the simulations using the “hard” correction with *k *=* *5 and *d*_0_ **=* *30 parameter settings is shown in Figure S3.4

Model parameters	Type of correction		
	Hard	Soft	Uncorrected
*k *=* *3 and *d*_0_ * *=* *30	18/358/692/13	8/359/686/28	1/360/628/92
*k *=* *10 and *d*_0_ * *=* *50	8/359/706/8	2/360/689/30	1/360/639/81
*k *=* *5 and *d*_0_ * *=* *30	5/360/709/7	2/360/693/26	1/360/634/86

*Montenegrina* did not co‐occur with the families Bradybaenidae, Cochlicellidae and Euconulidae and three alopiinine genera: *Alopia*,* Carinigera* and *Dilataria* (Table S3.7). All these observed zero co‐occurrences were, however, within the simulated ranges. *Montenegrina* co‐occurred at least once with the other 40 taxa in our dataset, and the vast majority of these observed non‐zero co‐occurrence values was also within the simulated pairwise ranges, regardless of the correction types and model settings applied. Three of the nine models simulated more co‐occurrences than observed with the genus *Herilla*. Two of the models simulated fewer co‐occurrences than observed with the family Helicidae, and only one of the models with the families Enidae and Hygromiidae (Table S3.7).

### 3.2 Correlation between co‐occurrences and taxonomic relatedness

**Figure 4:**
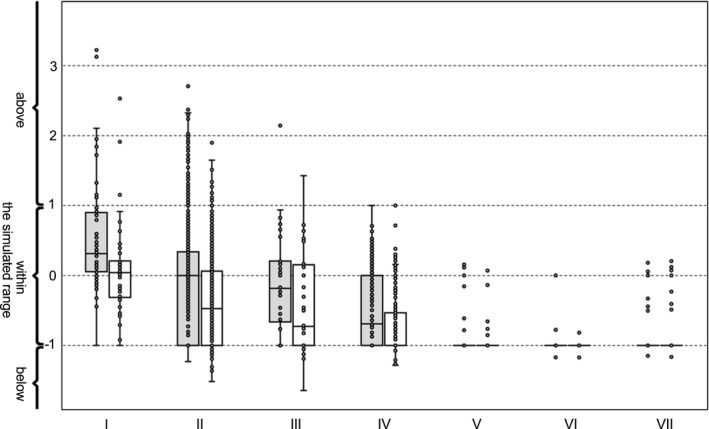
Box and whisker chart of observed pairwise co‐occurrence counts of Balkan land snail taxa categorized into seven different groups based on the taxonomic/phylogenetic relatedness of their members: particularly whether they are pairs of caenogastropods and pulmonates, related at the class level (I); pairs of different pulmonate families, related at the subclass level (II); pairs of alopiinid and non‐alopiinid doorsnails, related at the family level (III); pairs of different alopiinine genera, related at the subfamily level (IV); pairs of *Montenegrina* subclades of different main intra‐generic clades (V); pairs of *Montenegrina* sub‐clades within the same main intra‐generic clades (VI); or pairs of *Montenegrina* morphospecies (VII). For more detailed explanation of how the seven categories were defined see Figure S1.1. Each observed absolute count was rescaled to the range of values simulated with the same taxon pair according to eq. (3). Results of the two simulations, resulting in the most extreme ranges, are illustrated here: the “hard” correction with *k *=* *3 and *d*_0_ **=* *30 km (right) and the “uncorrected” model with *k = *5 and *d*_0_ **=* *30 km parameter settings (left)

We categorized pairwise co‐occurrence counts by taxonomic relatedness. The most extreme outliers were at the class level, where we found only higher‐than‐expected outliers. By contrast, at and below the subfamily level there were no higher‐than‐expected outliers at all. In general, we found that the closer related the taxon pair members were, the lower the observed co‐occurrence counts were relative to the simulated ones on average (Figure 4).

However, at and below the subfamily level, the observed co‐occurrence counts were very often zero (74% among alopiinine genera, 91–93% among *Montenegrina* clades, and 97% among *Montenegrina* species), but most of them fell within the simulated ranges (Figure 4). There were only nine observed co‐occurrences among the 27 *Montenegrina* morphospecies (*Y3* matrix, Table S1.6), one co‐occurrence among the main intra‐generic *Montenegrina* clades, and further six among the subclades (*Y2* matrix, Table S1.5).

**Figure 5:**
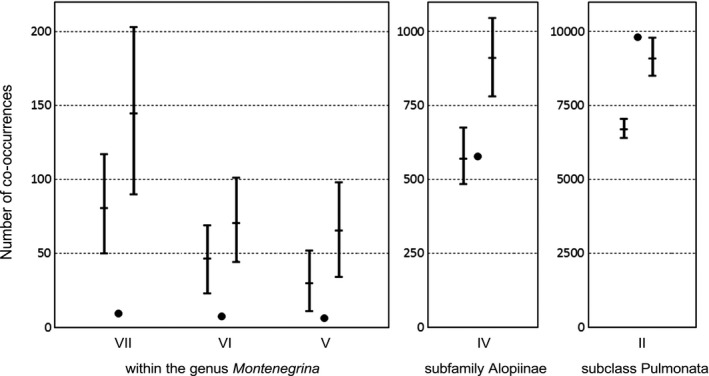
Pairwise co‐occurence counts cumulated for groups of Balkan land snail taxon pairs. The phylogenetic relatedness‐based division is the same as that of Figure S1.1 and Figure 4, but two of them, due to low numbers of elements, where left out. Dots indicate observed values, maximum–mean–minimum lines indicate simulated values. Vertical axes indicate the number of co‐occurrences. Detailed results of the nine different model settings are given in Table 2. Here, only the two most extreme simulated ranges are illustrated; namely those of the “hard” correction with *k *=* *3 and *d*_0_ **=* *30 km (right) and the “uncorrected” model with *k = *5 and *d*_0_ **=* *30 km parameter settings (left)

Cumulated data showed a similar picture. For the 29 pulmonate families the cumulative observed co‐occurrence value was almost within the ranges simulated by the models applying “hard” correction, and well above those simulated by the “soft” and the “uncorrected” models. For the 17 alopiinine genera the cumulative observed co‐occurrence values fell within the ranges simulated by the three “uncorrected” models, and slightly below those that were simulated by the six corrected models. Regardless of the model settings, correction types and the way of division (phylogeny‐ or morphology‐based), total observed co‐occurrences among *Montenegrina* subgroups were far fewer than expected (Figure 5, Table 2).

**Table 2:** Cumulative pairwise co‐occurence counts of Balkan land snail taxa grouped by the phylogenetic/taxonomic relatedness of taxon pair members. These groups are 27 *Montenegrina* morphospecies, *Montenegrina* subclades within the main clades, *Montenegrina* subclades between the main clades, 17 genera in the subfamily Alopiinae and 29 families of the class Pulmonata. Simulations of pairwise co‐occurrences were made with nine different combinations of model corrections and settings. Values of taxon pairs in the same category were summed by each simulation round. Ranges (minimum–maximum), as well as mean values of the cumulated counts were calculated. The two most extreme ranges, namely those simulated by the “hard” correction with *k *=* *3 and *d*_0_ **=* *30 km and by the uncorrected model with *k = *5 and *d*_0_ **=* *30 km parameter settings are illustrated in Figure 5

		Observed values	Means and ranges of simulated values			
			*k* and *d*_0_	Type of correction		
			Settings	Hard	Soft	Uncorrected
*Montenegrina*			3/30	144.4 (90–203)	122.2 (75–192)	86.3 (49–132)
	27 morphospecies	9	10/50	144.3 (91–206)	122.6 (71–184)	85.8 (54–128)
			5/30	132.4 (82–180)	111.9 (77–155)	80.7 (50–117)
			3/30	65.5 (34–98)	55.1 (31–87)	38.4 (18–68)
	Within main clades	6	10/50	60.6 (33–98)	50.7 (26–83)	35.7 (16–58)
			5/30	50.4 (28–89)	42.6 (19–73)	29.9 (11–52)
			3/30	70.5 (44–101)	60.1 (36–91)	42.9 (18–70)
	Btw. main clades	1	10/50	74.0 (45–110)	63.2 (32–97)	45.5 (23–71)
			5/30	73.8 (47–111)	63.4 (38–98)	46.4 (23–69)
Alopiinae among 17 genera			3/30	910 (781–1045)	801 (696–926)	625 (530–727)
		570	10/50	851 (723–973)	758 (639–865)	595 (502–693)
			5/30	815 (697–941)	756 (664–871)	575 (484–675)
Pulmonata among 29 families			3/30	9083 (8511–9790)	8134 (7602–8585)	6477 (6119–6871)
		9798	10/50	9212 (8657–9753)	8245 (7767–8720)	6594 (6212–6986)
			5/30	9326 (8821–9886)	8244 (7694–8770)	6700 (6394–7050)

## 4 DISCUSSION

We evaluated co‐occurrence patterns of rock‐dwelling land snails in a phylogenetic perspective and we found strong support to the assumed dominance of non‐adaptive factors in their speciation (Gittenberger, 1991, 2004). In order to demonstrate this we developed RaCoCOS (range‐constrained co‐occurrence simulation), a method comprising a probabilistic framework to define taxon ranges, a co‐occurrence simulation null model that accounts for taxon ranges, and a method to assess and correct for biases in opportunistically collected biotic data.

### 4.1 A need for range‐constrained co‐occurrence analysis

Elton (1946) suggested that, based on the competitive exclusion principle, co‐occurrence patterns of two or more taxa can provide information about their niches. However, it has rarely been applied to gastropods (an exception is Dillon, 1987), and never to obligate rock‐dwelling gastropods. Frequent and permanent co‐existence of two species is a strong, though indirect, indication that their niches are not identical. No or fewer than expected co‐occurrences indicate the opposite, even if in some cases two species of identical niches might coexist because competitive exclusion has not yet run into completion. It is difficult to assess whether few or zero co‐occurrences of two taxa are actually fewer than expected. Different habitat preferences or non‐overlapping distribution ranges can result in very few or even zero co‐occurrences, which are not fewer than expected. Hence, we can draw conclusions about the niches only when effects by other factors are excluded. In this study we eliminated habitat‐related differences by including only sites at which the habitats were similar.

Controlling for range‐related factors was more challenging, as compared to the size of the study area most of the involved species and genera have smaller range and many of them are partially allopatric. Methods for co‐occurrence analysis are widely used in community ecology research (Gotelli, 2000). In the cases of dispersal‐limited taxa a severe limitation of traditional null‐model techniques is their inability to account for the spatially autocorrelated nature of the species distributions. As a consequence, these methods cannot be applied directly to a study system like ours, comprising partially allopatric species (Stone, Dyan, & Simberloff, 1996). To circumvent this, we introduced RaCoCOS which simulates co‐occurrences under the constraint of the geographic ranges of the taxa.

### 4.2 Definition of geographic ranges

In contrast to range definition methods that sharply outline areas, for instance by drawing convex hulls around the known occurrence records (Connor, Collins, & Simberloff, 2013), in this study, we defined taxon ranges through a spatial distribution kernel based on the probability of occurrence, which is the function of a spatial weight matrix and the known presences and absences of the taxon. This approach can take into account the frequency (number of occurrences relative to the range size) and uneven density of taxa within their ranges, as well as whether two taxa are regionally allopatric or widely interspersed within the range overlap. Its theoretical background is the assumption that current ranges and distribution patterns are the results of various, partly deterministic factors like ancestral locations (“areas of origin” or “areas of refuge”), dispersal limitations (time and spatial dependence of colonization events), and the probabilities of local extinction events. When we found a high proportion of presence records of a given taxon within an area, it was interpreted as an indication that all potentially suitable sites in that area had a high probability of being colonized. Hence, we assigned high occurrence probability values for that taxon to each site in that area. By contrast, a high proportion of absence records (no or just sporadical presences) of a taxon in a given area meant that any site had a low probability of being colonized by the taxon, therefore the sites belonging there received low probability values (see Figure 1b,c for an example).

Mathematically the distance decay of spatial weights can be defined in different ways (Murayama, 2012). Here we chose a logistic function and defined its parameters empirically. To assess the sensitivity of the results to these settings we used three pairs of *k* and *d*_0_ parameters, resulting in different decay curves of the distance function of the *W*_*ij*_ variable between 10 and 100 km (see Figure S2.2). Due to the way the Equation (1) formula is calculated, larger *d*_0_ leads to a larger contribution of more distant sites to the *OP* value of a given location, whereas lower *k* value leads to less abrupt changes of spatial weights with distance. That is, the higher *d*_0_ and the lower *k* are selected for Equation (1), the wider the simulated ranges spread. Thus, a given number of simulated occurrences scatter across a larger area. If ranges spread wider, the overlaps, as well as the number of simulated co‐occurrences, are expected to be higher between allopatric pairs of taxa (e.g. as seen in the cases of *Montenegrina* and *Albinaria*, and *Montenegrina* and *Alopia*). In sympatric taxa, however, wider simulated ranges do not result in proportionally larger range overlap, but a given number of occurrences still scatter across a larger area, and thus fewer co‐occurrences are expected (e.g. as seen with *Montenegrina* and *Strigilodelima*) (Table S3.7).

The distances considered relevant in terms of dispersal and colonizing ability can vary considerably between different animal groups, and it is conceivable that taxon‐specific parameterization of the distance decay would increase the effectiveness of our method. Finding a way to objectively define weight distances was beyond the scope of this study, but this might be a future advancement. A promising approach could be combining spatial filtering methods developed for data sampled at non‐regular grids (Wagner & Dray, 2015) with extensions to presence–absence and presence only datasets.

Nevertheless, the sensitivity analyses indicated that the model was far less sensitive to changes in these parameters than to the application of differing correction types (Tables 1, 2, S3.7).

Once the *d*_0_ and *k* parameters are selected, further points to consider are sampling density and the size of the study area. If, compared to the selected *d*_0_ and *k* parameters, the study area is sampled at too low a density, the *OP* values of each site will be determined mainly (sometimes exclusively) by their own presence–absence statuses. Thus, after fitting model parameters to the study system, the spatial representation of the samples should be in accordance with the selected model parameters. At peripheral sites of the delimited study area the exclusion of sites in neighbouring outside regions might be a further source of bias in the *OP* calculation. To circumvent this, either complete territorial units (e.g. an entire island) should be selected or, when not possible, the study area should contain a reasonably wide peripheral “buffer zone.”

### 4.3 Quality assessment of biotic data

Even with standardized sampling methods locally rare or smaller‐sized species, as well as those preferring cryptic microhabitats, are more likely to be overlooked than others and thus are underrepresented in biotic datasets (Sólymos, 2007). Furthermore, if the sampling effort was not equal the more effort‐demanding taxa may also show aggregation at better explored sites. If we use biotic data collected by different methods for different original purposes, which is usually the case for datasets harvested from various sources, we might reasonably expect the dataset to be biased to some degree. In order to correct this, and/or be able to interpret the analysis outputs properly, it is essential to assess the quality of the datasets.

Considering similar habitats and equal sampling effort, one would expect a nearly symmetric density distribution of taxon per site numbers, and also small differences in the average numbers of other taxa with which a given taxon co‐occurs. If in a dataset the density distribution of taxa per site values considerably deviates from this assumption and certain taxa differ substantially in the average numbers of taxa they co‐occur with, it might be the indication of biased data. Our field experience suggests that our dataset's deviation from the symmetry assumption must be due to uneven sampling, rather than to differences in habitat suitability. In other words, in most cases low taxon richness at a site is primarily due to cursory sampling. The fact that those taxa demanding most sampling effort were found to be associated with the higher taxon richness strongly supports this assumption. At the same time, we might also suppose that some taxa, specifically those requiring least sampling effort (e.g. members of the Alopiinae, upon which our study focused) are more reliably represented in this dataset than others.

The *OP* value of a given taxon at a given site is calculated only on the basis of its presence at the neighbouring sites, regardless of the presence or absence of other taxa. Therefore, the *OP* values, as we calculated them, are not capable of reflecting differences in the sampling efforts. If the simulation of taxon distributions is based only on the rescaled *OP* matrix (as in the “uncorrected” models), the sum of the elements in the simulated Ψ matrices will approximate the total number of the observed presence data (the sum of the *Y* matrix), and the frequency distribution of the simulated matrix will be symmetrical with a mode similar to the mean. The more uneven the sampling that produced the raw presence–absence data, the more right‐skewed would be the frequency distribution of the observed taxa per site values (rows sum of the *Y* matrix). As the number of co‐occurences at a site is equal to (*n* ^2^–*n*)/2, if *n* is the number of presence records, it is easy to foresee that higher *n* implicates exponentially higher co‐occurrence values. When the distribution of *n* value density gets more right‐skewed and platykurtic, that is the number of sites of around average *n* values decrease and those above and below it increase, the total number of co‐occurrences will also increase.

In view of the above, the fact that the sum of observed co‐occurrences was far larger than that we simulated under uncorrected model assumption was primarily due to the deviation of observed data from null distribution, and thus reflected data quality rather than real taxon interactions.

Such bias can be best corrected if the density of taxon per site values of the simulated Ψ matrices approximate those of the observed data. Correction methods that define null hypotheses taking number of taxa per site proportional to observed species‐richness are in general use (e.g. SIM5 type model in Gotelli, 2000). Our assumption, however, was that the biased distribution of species‐richness per site in our dataset is mainly due to uneven sampling and not to differential habitat suitability. Instead of directly using the number of taxa per site values for correction, we introduced an additional step, namely the randomization of the number of taxa per site vector, before each simulation round (Figure S2.3). The correction with this vector ensured that the distribution of simulated species‐richness per site values approximated those of the observed ones (and, therefore, the sum of total co‐occurrences did the same). But, due to the randomization step before each simulation round, the uneven sampling effort can be simulated under the assumption of equal habitat suitability. As the “hard” correction is based on such taxa per site density distribution as that of the input *Y* matrix (Figure 3), the total sum of co‐occurrences simulated under this model correction is close to the observed value.

The “hard” correction outlined above excludes as many sites from each simulation round as have zero presences in the input dataset. It is conceivable that one might encounter *Y* matrices that contain a certain number of sites with zero presence values (either because no taxa were sampled at such sites, or because the sampled ones are excluded from the analysis). The fewer the taxa that comprise an input *Y* matrix and the higher the number of sites without presence records, the stronger the constraint imposed by the “hard” correction method. The “soft” correction was introduced to relax this constraint by not allowing the exclusion of any of the sites from any of the simulation rounds. Due to the taxa per site density distribution on which the simulation was based, the resulting total sum of co‐occurrences was between those of the “hard” and the “uncorrected” models.

The “hard” method corrects according to the average bias of the raw data. As demonstrated above, the representation of different taxa may be differently biased in such opportunistically collected biotic datasets. Accordingly, the “hard” correction method might “overcorrect” for better represented taxa than for others, for example for *Montenegrina* and for the other obligate rock‐dwelling alopiinine genera in this study. Such “overcorrection” might result in false dissociations (Type I error), or might mask existing dissociations (Type II error). As it is usually impossible to precisely define which taxa deviate, and to what extent, from the average bias in a biotic dataset, it is expedient to make simulations with different models and to take them all into account when interpreting the results.

### 4.4 Co‐occurrence in a phylogenetic perspective

When we compared co‐occurrence patterns at different phases of phylogenetic divergence a somewhat similar concept was followed to that of the “age‐range correlation,” where the geographic range overlaps are placed into a phylogenetic perspective (Fitzpatrick & Turelli, 2006). At the family level we assumed no competitive exclusion (Diamond & Gilpin, 1982), and thus we used family level records as “negative control” in our study, expecting neither associations nor dissociations in co‐occurrences. As expected, random co‐occurrence patterns were indicated by corrected models, but under uncorrected model assumptions these appeared to include associations. As it was demonstrated above that raw data are biased, we suspect that any associations at the family level under the uncorrected model assumptions are due to this, rather than to any real interactions between the families. It was more difficult interpreting the patterns found at the genus level among alopiinine door snails. Here corrected models infer some dissociations. If we consider the simulations of these models more realistic, we might assume that even at the genus level there is some degree of competition. An alternative explanation can be that the less biased representation of these taxa in the raw presence–absence datasets leads to the overestimation of their expected co‐occurrences.

Observed co‐occurrences among *Montenegrina* species and subgeneric clades are far fewer than expected under the assumption of random distribution, regardless of the model settings or correction types we applied. Below the genus level the differences between the observed and simulated values are so clear and obvious that it can be interpreted with little doubt as a sign of competitive exclusion and, indirectly, as an indication that the appearance of subgeneric clades and species did not result in considerable niche partitioning. Compared to the phylogenetic divergence, the rate of niche differentiation appears to have lagged behind. This can be viewed as strong support for the hypothesis that the speciation of rock‐dwelling gastropods, at least those belonging to the alopiinine door snails, was driven primarily by non‐adaptive processes (Gittenberger, 1991).

Nevertheless, there are at least 10 known cases when distinct species of *Montenegrina* co‐occur (Fehér & Szekeres, 2016: Table 1). Nine of these were included in the raw presence–absence dataset of this study. Although this number is far lower than expected from any of the simulations, it is worth considering how and why these species can co‐occur. There are two likely explanations. One is that even if most of the *Montenegrina* species have highly similar niche preference, some of them might already diverged in this respect. Then these niche‐diverged species would account for the few co‐occurrences. This might explain two of the observed cases at the shore of Lake Ohrid, where *M. stankovici* (Urbański, 1960) co‐occurs with *M. dofleini pinteri* Nordsieck, 1974 and *M. perstriata ochridensis* (Wagner, 1925). *Montenegrina stankovici* prefers a microhabitat different from those of its congeners, namely exclusively inhabiting rocks in the immediate vicinity of the lake surface. At these two co‐occurrence sites an apparent spatial segregation can be observed on a fine scale as the congeners live somewhat farther from the water. The other possible explanation is that joint occurrences of *Montenegrina* species result from very recent or recurrent colonization events. Such co‐occurrences are assumed to be transient states, before one species sweeps out the other by competitive exclusion. This explanation seems very feasible in at least five of the known cases, where two *Montenegrina* taxa co‐occur in gorges of rivers or streams with drainages hosting both species allopatrically (Fehér & Szekeres, 2016: Table 1). These gorges harbouring descendants of more than one *Montenegrina* species function as natural filters for washed‐away individuals. Although in the drainage area these species occur allopatrically, incidental or recurrent colonization can result in their coexistence at these gorges. If this assumption is correct, such sites can be viewed as “natural experiments” that offer ideal model systems for studying the spatial and temporal dynamics of competitive exclusion.

### 4.5 Prospects

With the use of community null‐modelling techniques we studied co‐occurrence patterns in range‐wide distribution data of gastropods from an evolutionary point of view. In order to draw meaningful conclusions we assembled a large distribution dataset of various gastropod taxa of similar habitats. By using geographical distribution kernels the introduced method proved capable of accounting for range differences in allopatrically distributed taxa. It includes a correction method to balance biased distribution data that arise from differential sampling. This is particulary useful if we harvest presence–absence data from museum databases or public biodiversity repositories. Although it was developed for rock‐dwelling land snails, this null‐modelling framework can be adopted with little alteration for the analysis of large‐scale distribution records of other taxonomic groups. Hence, our approach can provide a blueprint for studies addressing a variety of biogeographical, phylogeographical, evolutionary, or community ecological problems in relation to allopatry.

## BIOSKETCH

The Alpine Land Snails Working Group (http://snails.nhm-wien.ac.at/) is based at the Natural History Museum Vienna with a broad interest in evolutionary biology. Combining classical morphological methods, habitat and distribution data with molecular analyses they try to elucidate the evolutionary and phylogeographical histories of various study systems, primarily gastropods, in the Alpine–Carpathian–Dinaric region.

Author contributions: Z.F., B.P‐G. and P.S. conceived the idea for the study; Z.F. compiled the distribution dataset; P.S. wrote the R script; Z.F. performed the simulations; K.M. and S.B. conducted the laboratory work; Z.F. carried out the molecular data analysis with significant contribution of E.H., K.M., S.B. and M.S.; all authors contributed to data interpretation and writing.
