# Supplementary material for: Range‐constrained co‐occurrence simulation reveals little niche partitioning among rock‐dwelling Montenegrina land snails (Gastropoda: Clausiliidae)
Source: J Biogeogr. 2018 Apr 16;45(6):1444–57. doi: 10.1111/jbi.13220 (PMC6027963; doi:10.1111/jbi.13220)
Supplement: Supplementary file 1 [file JBI-45-1444-s001.pdf]

Journal of Biogeography

SUPPORTING INFORMATION

Fehér Z, Mason K, Szekeres M, Haring E, Bamberger S, Páll-Gergely B, Sólymos P: Range-constrained co-occurrence simulation reveals little niche partitioning among rock-dwelling *Montenegrina* land snails (Gastropoda: Clausiliidae). DOI: 10.1111/jbi.13220

Appendix S1. Material studied

Figure S1.1. A simplified tree and taxon pair matrices to illustrate taxonomic relatedness of of landsnail taxa included in the co-occurrence analysis. The tree is based on the *Y2* matrix (Table S1.5), where the division of genus *Montenegrina* follows the mitochondrial phylogeny. Clade assignments of *Montenegrina* correspond to Tables S1.2 and S1.3.

Table S1.1. Landsnail taxa included in the co-occurrence analysis. The focal group was the genus *Montenegrina* , which was further divided into subgroups in different ways (see Table S1.3). Other taxa were binned into 46 groups as follows: other species of the Alopiinae subfamily were distinguished at the genus level (16 genera); other pulmonate landsnails, including those in other clausiliid subfamilies, were distinguished at the family level (29 families); and gill-breathing species of the superorder Caenogastropoda, were merged into one group. The first column indicates in how many of the total 1649 sites a given taxon is present. The second column indicates the average number of co-occurring taxa.

Table S1.2. Samples used for the *Montenegrina* phylogenetic reconstruction.
These samples were studied and discussed from a shell morphological point of view in the recent revision of the genus (Fehér & Szekeres 2016). Samples can be identified by the museum voucher numbers. Museum acronyms are the following: HNHM (Hungarian Natural History Museum, Budapest); NHMW (Natural History Museum Vienna); SMNS (Stuttgart State Museum of Natural History). The same DNA sample IDs are used in the GenBank entries.
DNA isolation from alcohol-stored specimens was done by the First-DNA all tissue kit (Gen-ial, Troisdorf, Germany) following the manufacturer's protocol. From mummified samples it was done according to the method of Thomsen et al. (2009) modified to gastropods by Páll-Gergely et al. (2015) (denoted as DTT). Partial sequences of the mitochondrial cytochrome oxidase subunit I gene (COI, 655 bp), the 16S rRNA gene (845–866 bp) and the 12S rRNA gene (677–713 bp) were amplified by polymerase chain reaction (PCR) following Harl et al. (2014). In some samples, when the above mentioned primers could not amplify the targeted 16S sequence, we used an alternative primer pair, the 16SarF (Palumbi 1996) with 16SLOrc_rev (Harl et al. 2014), to amplify a smaller 16S DNA fragment. Successfully amplified products were sequenced in both directions at LGC Genomics (Berlin, Germany) using the PCR primers. For the positions of the individual sequences in the phylogenetic tree see Fig. S1.1.

Table S1.3. Subdivision of *Montenegrina* occurrences by mitochondrial clades (into 3 or 15 groups) and by species according to the current morphology based system. Two species, *M. apfelbecki* and *M. chiasma* are missing from the dataset. Due to some discordance between the traditional system and the mitochondrial phylogeny, *M. dofleini, M. skipetarica,* M. hiltrudae and *M. perstriata* records belong to more than one clade. Numbers of occurrences are indicated in brackets. Due to co-occurrences, groups' values are not always equal to the subgroups' sums.
[1] M. s. skipetarica, M. s. danyii, M. s. gurelurensis, M. s. pindica, M. s. pifkoi, M.s. puskasi and M.s. remota belong to Clade B;
M. s. csikii, M. s. ersekensis, M. s. flava, M. s. konitsae, M. s. nobilis, M. s. rugosa, M. s. thysi and M. s. voidomatis belong to Clade C.
[2] M. d. wagneri belongs to Clade E, all other M. dofleini subspecies belong to Clade D2.
[3] M. p. drimica, M. p. occidentalis and M. p. plenostoma belong to Clade H.
[4] M. p. ochridensis, M. p. callistoma and M. p. tenebrosa belong to Clade I.
[5] M. p. radikae belongs to Clade K.
[6] M. p. perstriata, M. p. diminuta and M. p. subcristatula belong to Clade L
[7] M. hiltrudae is paraphyletic because M. h. robusta belongs to the Clade D2.

Table S1.4. Data matrix containing presence–absence records of 47 landsnail taxa in 1649 localities.
In this dataset, taxa were merged into groups as follows: species of the subfamily Alopiinae were grouped at the genus level (17 genera); other pulmonate landsnails, including those in other clausiliid subfamilies, were grouped at the family level (29 families); and gill-breathing landsnails of the superorder Caenogastropoda, merged into one group. This data matrix directly exported to CSV format is used in the demo of the RaCoCOS methodology (see Appendix S2).

Table S1.5. Data matrix containing presence–absence records of 61 landsnail taxa in 1649 localities.
In this dataset, taxa were merged into groups as follows: Montenegrina species were divided into 15 subgeneric groups according to the mitochondrial phylogeny (i.e. clades A, B, C, D1, D2, E, F1, F2, F3, G; H, I, J, K, L, see: Fig. S1.1). All other taxa were grouped as in the initial data matrix.

Table S1.6. Data matrix containing presence–absence records of 73 landsnail taxa in 1649 localities.
In this dataset, taxa were merged into groups as follows: Montenegrina species were divided into 27 species according to the current system of the genus (Fehér and Szekeres 2016). All other taxa were grouped as in the initial data matrix.

References
Harl, J., Páll-Gergely, B., Kirchner, S., Sattmann, H., Duda, M., Kruckenhauser, L. & Haring, E. (2014) Phylogeography of the land snail genus *Orcula* (Orculidae, Stylommatophora) with emphasis on the Eastern Alpine taxa: speciation, hybridization and morphological variation. BMC Evolutionary Biology, 14 (1), 223. http://dx.doi.org/10.1186/s12862-014-0223-y
Páll-Gergely B, Fehér Z, Hunyadi A, Asami T (2015): Revision of the genus *Pseudopomatias* and its relatives (Gastropoda: Cyclophoroidea: Pupinidae). Zootaxa 3937 (1): 1–49.
Palumbi, S.R. (1996) PCR and molecular systematics. In: Hillis, D., Moritz, C. & Mable, B. (Eds.), Molecular Systematics. 2nd Edition. Sinauer Press, Sunderland, pp. 205–248.
Thomsen, P.F., Elias, S., Gilbert, M.T.P., Haile, J., Munch K., Kuzmina, S., Froese, D.G., Sher, A., Holdaway, R.N. & Willerslev, E. (2009) Non-Destructive Sampling of Ancient Insect DNA. PLoS ONE, 4 (4), e5048. http://dx.doi.org/10.1371/journal.pone.0005048

Figure S1.1

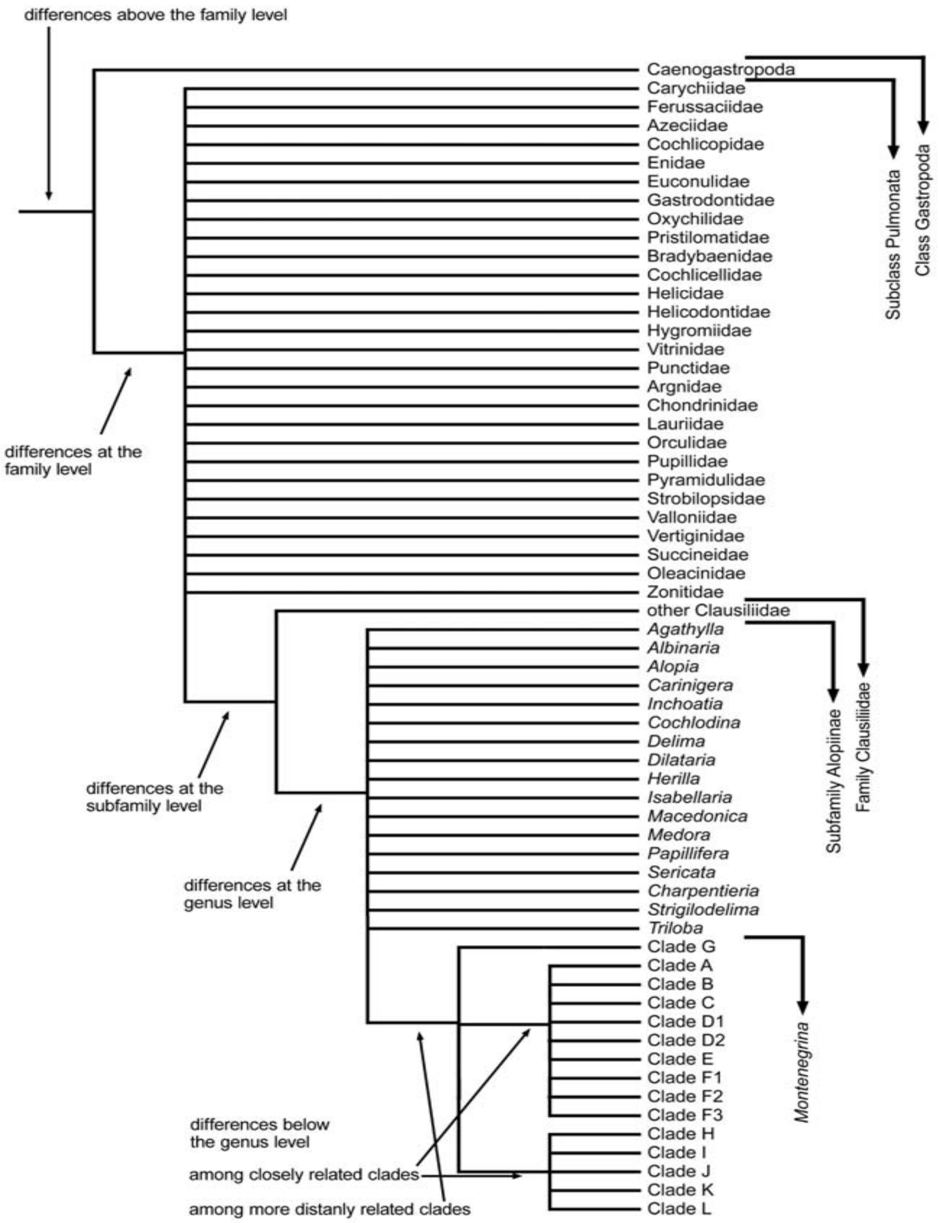

Y1 matrix

		Pulmonata							
				Clausiliidae					
					Alopiinidae				
	Caenogastropoda	Carychiidae	...	Zonitidae	other Clausiliidae	*Agathylla*	...	*Triloba*	*Montenegrina*
Caenogastropoda	--	I	I	I	I	I	I	I	I
Carychiidae		--	II	II	II	II	II	II	II
⋮			--	II	II	II	II	II	II
Zonitidae				--	II	II	II	II	II
other Clausiliidae					--	III	III	III	III
Agathylla						--	IV	IV	IV
⋮							--	IV	IV
Triloba								--	IV
Montenegrina									--

taxonomic/phylogenetic relatedness of the taxon pair members

I: at the class level (Caenogastropoda vs. Pulmonata)
II: at the subclass level (within Pulmonata)
III: at the family level (within Clausiliidae)
IV: at the subfamily level (within Alopiinae)

Y3 matrix

		Pulmonata									
				Clausiliidae							
					Alopiinae						
									Montenegrina		
	Caenogastropoda	Carychiidae	...	Zonitidae	other Clausiliidae	*Agathylla*	...	*Triloba*	*M. attemsi*	...	*M. zilchi*
Caenogastropoda	--	I	I	I	I	I	I	I	I	I	I
Carychiidae		--	II	II	II	II	II	II	II	II	II
⋮			--	II	II	II	II	II	II	II	II
Zonitidae				--	II	II	II	II	II	II	II
other Clausiliidae					--	III	III	III	III	III	III
Agathylla						--	IV	IV	IV	IV	IV
⋮							--	IV	IV	IV	IV
Triloba								--	IV	IV	IV
M. attemsi									--	VII	VII
⋮										--	VII
M. zilchi											--

taxonomic/phylogenetic relatedness of the taxon pair members

I: at the class level (Caenogastropoda vs. Pulmonata)
II: at the subclass level (within Pulmonata)
III: at the family level (within Clausiliidae)
IV: at the subfamily level (within Alopiinae)

VII: at the genus level (within *Montenegrina*)

Y2 matrix

		Pulmonata													
				Clausiliidae											
					Alopiinae										
									Montenegrina						
									A-F			G	H-L		
	Caenogastropoda	Carychiidae	...	Zonitidae	other Clausiliidae	*Agathylla*	...	*Triloba*	clade A	...	clade F3	clade G	clade H	...	clade L
Caenogastropoda	--	I	I	I	I	I	I	I	I	I	I	I	I	I	I
Carychiidae		--	II	II	II	II	II	II	II	II	II	II	II	II	II
...			--	II	II	II	II	II	II	II	II	II	II	II	II
Zonitidae				--	II	II	II	II	II	II	II	II	II	II	II
other Clausiliidae					--	III	III	III	III	III	III	III	III	III	III
Agathylla						--	IV	IV	IV	IV	IV	IV	IV	IV	IV
...							--	IV	IV	IV	IV	IV	IV	IV	IV
Triloba								--	IV	IV	IV	IV	IV	IV	IV
clade A									--	VI	VI	V	V	V	V
...										--	VI	V	V	V	V
clade F3											--	V	V	V	V
clade G												--	V	V	V
clade H													--	VI	VI
...														--	VI
clade L															--

taxonomic/phylogenetic relatedness of the taxon pair members

I: at the class level (Caenogastropoda vs. Pulmonata)
II: at the subclass level (within Pulmonata)
III: at the family level (within Clausiliidae)
IV: at the subfamily level (within Alopiinae)

V: at the genus level (btw. the 3 main clades within *Montenegrina*)
VI: at the intra-generic clade level (within the 3 clades of *Montenegrina*)

Table S1.1

			no. of locations where present	average number of co-occurring taxa	genera included
		Caenogastropoda	799	4.80	*Acicula, Platyla, Renea, Cochlostoma, Hydrocena, Pomatias*
Pulmonata		Carychiidae	6	12.83	*Carychium*
		Ferussaciidae	21	9.19	*Cecilioides, Ferussacia, Hohenwarthia*
		Azeciidae	40	6.08	*Hypnophyla*
		Cochlicopidae	13	9.38	*Cochlicopa*
		Enidae	513	5.56	*Chondrula, Mastus, Napaeopsis, Zebrina, Ena, Jaminia, Pseudochondrula, Merdigera, Multidentula*
		Euconulidae	7	11.57	*Euconulus*
		Gastrodontidae	2	6.50	*Zonitoides*
		Oxychilidae	259	7.25	*Carpathica, Daudebardia, Cellariopsis, Mediterranea, Morlina, Oxychilus, Schistophallus*
		Pristilomatidae	238	7.09	*Gyralina, Lindbergia, Vitrea*
		Bradybaenidae	8	8.00	*Fruticicola*
		Cochlicellidae	4	4.50	*Cochlicella*
		Helicidae	672	5.25	*Arianta, Cattania, Chilostoma, Faustina, Helicigona, Isognomostoma, Superba, Vidovicia, Cepaea, Codringtonia, Cornu, Eobania, Helix*
		Helicodontidae	315	5.20	*Helicodonta, Lindholmiola*
		Hygromiidae	549	5.72	*Trochoidea, Xerocrassa, Candidula, Xerotricha, Cernuella, Monachoides, Hiltrudia, Metafruticicola, Helicopsis, Xerocampylaea, Xerolenta, Xeromunda*
		Vitrinidae	102	7.49	*Oligolimax, Phenacolimax, Eucobresia, Semilimacella, Semilimax, Vitrina, Vitrinobrachium*
		Punctidae	26	10.73	*Punctum*
		Argnidae	33	9.21	*Agardhiella, Argna*
		Chondrinidae	668	5.12	*Chondrina, Granaria, Granopupa, Rupestrella*
		Lauriidae	34	6.82	*Lauria*
		Orculidae	121	7.18	*Odontocyclas, Orcula, Orculella, Pagodulina, Sphyradium*
		Pupillidae	16	9.88	*Pupilla*
		Pyramidulidae	186	7.50	*Pyramidula*
		Strobilopsidae	15	5.93	*Klemmia, Spelaeodiscus, Virpazaria*
		Valloniidae	33	10.67	*Acanthinula, Gittenbergia, Vallonia*
		Vertiginidae	83	8.45	*Columella, Truncatellina, Vertigo*
		Succineidae	5	4.60	*Oxyloma*
		Oleacinidae	275	6.13	*Poiretia*
		Zonitidae	146	6.01	*Aegopis, Allaegopis, Balcanodiscus, Doraegopis, Meledella, Paraegopis, Troglaegopis, Zonites*
	Clausiliidae	other Clausiliidae	293	5.34	*Alinda, Denticularia, Bulgarica, Clausilia, Idyla, Laciniaria, Macrogastra, Ruthenica, Sciocochlea, Pseudalinda, Vestia*
	Alopiinae	*Agathylla*	98	5.53	
		Albinaria	99	3.52	
		Alopia	35	2.20	
		Carinigera	45	4.27	
		Inchoatia	3	6.33	
		Cochlodina	93	6.30	
		Delima	204	4.56	
		Dilataria	2	5.00	
		Herilla	92	4.46	
		Isabellaria	10	4.50	
		Macedonica	44	5.50	
		Medora	78	4.33	
		Papillifera	10	6.70	
		Charpentieria	125	5.90	
		Strigilodelima	208	6.05	
		Triloba	28	7.07	
		Montenegrina	377	4.64	
			Sum: 7033	Mean: 6.53	

DNA_sample_id	Museum voucher	Taxon name	COI	16S	12S	country	locality	type_loc	latitude	longitude	collect_date	collected_by	DNA isolation	COI_primers	16S_primers	12S_primers	Clade
Iva-385-01	HNHM 99551	Vallatia vallata	KU307511	KU307997	KU307919	Greece	Kria		N39.7245	E20.8429	24-Jun-2013	Eross, Feher, Grego	Geni-Al	LCO1490/H2198-Alb	16SLOrc1_fwd/16SLOrc_rev	12SGastFw/12SGastRv	outgroup
M10-270-02	HNHM 99629	Montenegrina laxa iba	KU307512	KU307998		Albania	Tervol		N40.926	E20.2232	26-Aug-2006	Feher, Hunyadi, Huszar, Muranyi	Thomsen et al. (2009)	LCO1490/H2198-Alb	16SLOrc1_fwd/16SLOrc_rev		L
M10-270-03	HNHM 99629	Montenegrina laxa iba	KU307513	KU307999		Albania	Tervol		N40.926	E20.2232	26-Aug-2006	Feher, Hunyadi, Huszar, Muranyi	Thomsen et al. (2009)	LCO1490/H2198-Alb	16SLOrc1_fwd/16SLOrc_rev		L
M12-479-01	NHMW 110430/MN/0103	Montenegrina laxa errans	KU307514	KU308000		Albania	Strelce NE		N40.748	E20.5219	30-Jun-2014	Feher, Grego	Geni-Al	LCO1490/H2198-Alb	16SLOrc1_fwd/16SLOrc_rev		L
M13-296-01	HNHM 99467	Montenegrina skipetarica pifkoi	KU307515	KU308001	KU307920	Albania	above Macukull		N41.6908	E20.1468	08-Aug-2009	Barina, Pifko	Geni-Al	LCO1490/H2198-Alb	16SLOrc1_fwd/16SLOrc_rev	12SGastFw/12SGastRv	B
M13-297-01	HNHM 99468	Montenegrina skipetarica pifkoi	KU307516	KU308002		Albania	above Macukull		N41.6886	E20.1557	08-Aug-2009	Barina, Pifko	Geni-Al	LCO1490/H2198-Alb	16SLOrc1_fwd/16SLOrc_rev		B
M13-297-02	HNHM 99468	Montenegrina skipetarica pifkoi	KU307517	KU308003		Albania	above Macukull		N41.6886	E20.1557	08-Aug-2009	Barina, Pifko	Geni-Al	LCO1490/H2198-Alb	16SLOrc1_fwd/16SLOrc_rev		B
M13-297-03	HNHM 99468	Montenegrina skipetarica pifkoi	KU307518	KU308004		Albania	above Macukull		N41.6886	E20.1557	08-Aug-2009	Barina, Pifko	Geni-Al	LCO1490/H2198-Alb	16SLOrc1_fwd/16SLOrc_rev		B
M13-298-01	HNHM 99469	Montenegrina skipetarica pifkoi	KU307519	KU308005		Albania	above Macukull		N41.6878	E20.1568	08-Aug-2009	Barina, Pifko	Thomsen et al. (2009)	LCO1490/H2198-Alb	16SarF/16SLOrc_rev		B
M13-299-01	HNHM 99470	Montenegrina skipetarica pifkoi	KU307520	KU308006		Albania	Shkol-Den Pass near Macukull		N41.6903	E20.1659	09-Aug-2009	Barina, Pifko	Thomsen et al. (2009)	LCO1490/H2198-Alb	16SarF/16SLOrc_rev		B
M13-304-01	HNHM 99466	Montenegrina skipetarica pifkoi	KU307521			Albania	Macukull	paratype	N41.6962	E20.1333	19-May-2010	Feher, Muranyi, Ujvari	Geni-Al	LCO1490/H2198-Alb			B
M13-304-02	HNHM 99466	Montenegrina skipetarica pifkoi	KU307522			Albania	Macukull		N41.6962	E20.1333	19-May-2010	Feher, Muranyi, Ujvari	Geni-Al	LCO1490/H2198-Alb			B
M13-305-01	NHMW 111209	Montenegrina skipetarica pifkoi	KU307523	KU308007		Albania	Macukull	paratype	N41.6971	E20.1362	19-May-2010	Feher, Muranyi, Ujvari	Geni-Al	LCO1490/H2198-Alb	16SLOrc1_fwd/16SLOrc_rev		B
M14-303-01	HNHM 99517	Montenegrina grammica erossi	KU307524	KU308008		Albania	above the Varosh Spring	paratype	N41.6747	E20.2112	18-May-2010	Feher, Muranyi, Ujvari	Geni-Al	LCO1490/H2198-Alb	16SLOrc1_fwd/16SLOrc_rev		K
M14-322-01	HNHM 99515	Montenegrina grammica erossi		KU308009	KU307921	Albania	W of Shkemb i Skanderbeut	paratype	N41.6461	E20.1807	18-May-2010	Feher, Muranyi, Ujvari	Geni-Al		16SLOrc1_fwd/16SLOrc_rev	12SGastFw/12SGastRv	K
M15-309-02	HNHM 99484	Montenegrina subcristata	KU307525	KU308010	KU307922	Montenegro	Duravci S		N42.1677	E19.196	16-Jun-2012	Feher, Kovacs, Muranyi	Geni-Al	LCO1490/H2198-Alb	16SLOrc1_fwd/16SLOrc_rev	12SGastFw/12SGastRv	F1
M15-309-03	HNHM 99484	Montenegrina subcristata	KU307526			Montenegro	Duravci S		N42.1677	E19.196	16-Jun-2012	Feher, Kovacs, Muranyi	Geni-Al	LCO1490/H2198-Alb			F1
M16-311-02	HNHM 99486	Montenegrina prokletiana kovacsorum	KU307527	KU308011	KU307923	Albania	Toplane	paratype	N42.2339	E19.8741	18-Jun-2012	Feher, Kovacs, Muranyi	Geni-Al	LCO1490/H2198-Alb	16SLOrc1_fwd/16SLOrc_rev	12SGastFw/12SGastRv	G
M17-261-01	HNHM 99497	Montenegrina lillae	KU307528			Albania	Koman	paratype	N42.1087	E19.8264	14-Apr-2006	Eross, Feher, Hunyadi, Muranyi	Geni-Al	LCO1490/H2198-Alb			G
M17-312-01	HNHM 99498	Montenegrina lillae	KU307529	KU308012	KU307924	Albania	Koman	paratype	N42.1087	E19.8264	19-Jun-2012	Feher, Kovacs, Muranyi	Geni-Al	LCO1490/H2198-Alb	16SLOrc1_fwd/16SLOrc_rev	12SGastFw/12SGastRv	G
M17-312-02	NHMW 111227	Montenegrina lillae	KU307530			Albania	Koman	paratype	N42.1087	E19.8264	19-Jun-2012	Feher, Kovacs, Muranyi	Geni-Al	LCO1490/H2198-Alb			G
M18-316-01	HNHM 99503	Montenegrina sturanyi gropana	KU307531			Albania	Bize	paratype	N41.3392	E20.1989	20-Jun-2012	Feher, Kovacs, Muranyi	Geni-Al	LCO1490/H2198-Alb			H
M18-317-01	HNHM 99504	Montenegrina sturanyi gropana	KU307532	KU308013	KU307925	Albania	between Bize and Ballenje	paratype	N41.3604	E20.2412	20-Jun-2012	Feher, Kovacs, Muranyi	Geni-Al	LCO1490/H2198-Alb	16SLOrc1_fwd/16SLOrc_rev	12SGastFw/12SGastRv	H
M20-562-01	HNHM 99472	Montenegrina skipetarica danyii	KU307533	KU308014		Albania	Cidhne N 9 km	paratype	N41.8149	E20.2775	10-Oct-2005	Deli, Eross, Feher, Muranyi	Thomsen et al. (2009)	LCO1490/H2198-Alb	16SarF/16SLOrc_rev		B
M20-562-02	HNHM 99472	Montenegrina skipetarica danyii	KU307534	KU308015		Albania	Cidhne N 9 km		N41.8149	E20.2775	10-Oct-2005	Deli, Eross, Feher, Muranyi	Thomsen et al. (2009)	LCO1490/H2198-Alb	16SarF/16SLOrc_rev		B
M20-562-03	HNHM 99472	Montenegrina skipetarica danyii	KU307535			Albania	Cidhne N 9 km		N41.8149	E20.2775	10-Oct-2005	Deli, Eross, Feher, Muranyi	Thomsen et al. (2009)	LCO1490/H2198-Alb			B
M20-562-04	HNHM 99472	Montenegrina skipetarica danyii	KU307536			Albania	Cidhne N 9 km		N41.8149	E20.2775	10-Oct-2005	Deli, Eross, Feher, Muranyi	Thomsen et al. (2009)	LCO1490/H2198-Alb			B
M21-280-01	HNHM 99506	Montenegrina sturanyi sturanyi	KU307537	KU308016	KU307926	Albania	Qarishte E 5 km	paratype	N41.2505	E20.5034	04-Jul-2008	Barina, Pifko, Vojtko	Geni-Al	LCO1490/H2198-Alb	16SLOrc1_fwd/16SLOrc_rev	12SGastFw/12SGastRv	H
M22-282-01	HNHM 99509	Montenegrina nana barinai	KU307538	KU308017		Albania	Mount Temlishit		N41.5138	E20.3142	29-May-2008	Barina, Pifko	Geni-Al	LCO1490/H2198-Alb	16SLOrc1_fwd/16SLOrc_rev		I
M22-453-01	NHMW 111231	Montenegrina nana barinai	KU307539	KU308018		Albania	Mount Temlishit	paratype	N41.514	E20.316	15-Apr-2014	Feher, Nemeth, Mizsei	Geni-Al	LCO1490/H2198-Alb	16SLOrc1_fwd/16SLOrc_rev		I
M23-348-01	HNHM 99495	Montenegrina sporadica tropojensis	KU307540	KU308019		Albania	Tropoje N 13 km	paratype	N42.4749	E20.1518	08-Jun-2009	Barina, Lunk, Pifko, Schmidt	Geni-Al	LCO1490/H2198-Alb	16SLOrc1_fwd/16SLOrc_rev		G
M23-468-01	HNHM 99494	Montenegrina sporadica tropojensis	KU307541	KU308020		Albania	Tropoje N 13 km	paratype	N42.4692	E20.1554	26-Jun-2014	Angyal, Feher, Grego	Geni-Al	LCO1490/H2198-Alb	16SLOrc1_fwd/16SLOrc_rev		G
M26-356-01		Montenegrina hiltrudae sattmanni	KU307542			Albania	Shuets		N40.6847	E20.9984	26-May-2013	Dedov	Geni-Al	LCO1490/H2198-Alb			E
M27-357-01		Montenegrina hiltrudae sattmanni	KU307543			Albania	Glloboceni		N40.8575	E20.9429	23-Jun-2013	Dedov	Geni-Al	LCO1490/H2198-Alb			E
M28-358-01		Montenegrina hiltrudae costulata	KU307544			Albania	Zaroshka		N40.7668	E20.9277	27-May-2013	Dedov	Geni-Al	LCO1490/H2198-Alb			E
M28-358-02		Montenegrina hiltrudae costulata	KU307545			Albania	Zaroshka		N40.7668	E20.9277	27-May-2013	Dedov	Geni-Al	LCO1490/H2198-Alb			E
M28-358-03		Montenegrina hiltrudae costulata	KU307546			Albania	Zaroshka		N40.7668	E20.9277	27-May-2013	Dedov	Geni-Al	LCO1490/H2198-Alb			E
M29-373-01	NHMW 111212	Montenegrina rugilabris gregoi	KU307547	KU308021		Greece	Kipina Monastery W 1 km		N39.5668	E21.1231	23-Jun-2013	Eross, Feher, Grego	Geni-Al	LCO1490/H2198-Alb	16SLOrc1_fwd/16SLOrc_rev		D1
M29-373-02	NHMW 111212	Montenegrina rugilabris gregoi	KU307548	KU308022		Greece	Kipina Monastery W 1 km		N39.5668	E21.1231	23-Jun-2013	Eross, Feher, Grego	Geni-Al	LCO1490/H2198-Alb	16SLOrc1_fwd/16SLOrc_rev		D1
M29-373-03	NHMW 111212	Montenegrina rugilabris gregoi	KU307549	KU308023		Greece	Kipina Monastery W 1 km		N39.5668	E21.1231	23-Jun-2013	Eross, Feher, Grego	Geni-Al	LCO1490/H2198-Alb	16SLOrc1_fwd/16SLOrc_rev		D1
M29-374-01	NHMW 111213	Montenegrina rugilabris gregoi	KU307550	KU308024		Greece	Kipina Monastery		N39.5681	E21.1313	23-Jun-2013	Eross, Feher, Grego	Geni-Al	LCO1490/H2198-Alb	16SLOrc1_fwd/16SLOrc_rev		D1
M29-374-02	NHMW 111213	Montenegrina rugilabris gregoi	KU307551	KU308025		Greece	Kipina Monastery		N39.5681	E21.1313	23-Jun-2013	Eross, Feher, Grego	Geni-Al	LCO1490/H2198-Alb	16SLOrc1_fwd/16SLOrc_rev		D1
M30-375-01	NHMW 111211	Montenegrina rugilabris gregoi	KU307552	KU308026	KU307927	Greece	Prosilio NE 3 km to Syrrako	paratype	N39.5768	E21.0969	23-Jun-2013	Eross, Feher, Grego	Geni-Al	LCO1490/H2198-Alb	16SLOrc1_fwd/16SLOrc_rev	12SGastFw/12SGastRv	D1
M30-375-02	NHMW 111211	Montenegrina rugilabris gregoi	KU307553	KU308027		Greece	Prosilio NE 3 km to Syrrako	paratype	N39.5768	E21.0969	23-Jun-2013	Eross, Feher, Grego	Geni-Al	LCO1490/H2198-Alb	16SLOrc1_fwd/16SLOrc_rev		D1
M30-375-03	NHMW 111211	Montenegrina rugilabris gregoi	KU307554	KU308028		Greece	Prosilio NE 3 km to Syrrako	paratype	N39.5768	E21.0969	23-Jun-2013	Eross, Feher, Grego	Geni-Al	LCO1490/H2198-Alb	16SLOrc1_fwd/16SLOrc_rev		D1
M31-398-01	HNHM 99583	Montenegrina hiltrudae densicostulata	KU307555	KU308029	KU307928	Greece	Germas W 1.5 km		N40.4431	E21.4102	28-Jun-2013	Eross, Feher, Grego	Geni-Al	LCO1490/H2198-Alb	16SLOrc1_fwd/16SLOrc_rev	12SGastFw/12SGastRv	E
M32-405-01	HNHM 99571	Montenegrina dofleini kastoriae	KU307556	KU308030	KU307929	Greece	Krystallopigi		N40.6357	E21.0903	29-Jun-2013	Eross, Feher, Grego	Geni-Al	LCO1490/H2198-Alb	16SLOrc1_fwd/16SLOrc_rev	12SGastFw/12SGastRv	D2
M32-405-02	HNHM 99571	Montenegrina dofleini kastoriae	KU307557			Greece	Krystallopigi		N40.6357	E21.0903	29-Jun-2013	Eross, Feher, Grego	Geni-Al	LCO1490/H2198-Alb			D2
M32-405-03	HNHM 99571	Montenegrina dofleini kastoriae	KU307558	KU308031		Greece	Krystallopigi		N40.6357	E21.0903	29-Jun-2013	Eross, Feher, Grego	Geni-Al	LCO1490/H2198-Alb	16SLOrc1_fwd/16SLOrc_rev		D2
M32-547-01	NHMW 110430/MN/0049	Montenegrina dofleini kastoriae	KU307559			Greece	Krystallopigi		N40.6344	E21.0915	17-Oct-2014	Feher, Haring, Jaksch, Sattmann	Geni-Al	LCO1490/H2198-Alb			D2
M33-406-01	HNHM 99586	Montenegrina hiltrudae sattmanni	KU307560	KU308032	KU307930	Albania	Tren, Shpella e Trenit		N40.6723	E20.9871	29-Jun-2013	Eross, Feher, Grego	Geni-Al	LCO1490/H2198-Alb	16SLOrc1_fwd/16SLOrc_rev	12SGastFw/12SGastRv	E
M34-416-01	NHMW 110430/MN/0085	Montenegrina nana nana	KU307561	KU308033		Albania	Stebleve S 8.3 km		N41.3218	E20.3939	01-Sep-2013	Reischutz	Geni-Al	LCO1490/H2198-Alb	16SLOrc1_fwd/16SLOrc_rev		I
M36-429-02	HNHM 99638	Montenegrina soosi	KU307562	KU308034		Albania	Arren E, Gurri i Shkalles		N41.9188	E20.2693	08-Oct-2005	Deli, Eross, Feher, Muranyi	Thomsen et al. (2009)	LCO1490/H2198-Alb	16SLOrc1_fwd/16SLOrc_rev		L
M36-485-01	HNHM 99639	Montenegrina soosi	KU307563	KU308035		Albania	Arren		N41.9242	E20.2813	08-Oct-2005	Deli, Eross, Feher, Muranyi	Thomsen et al. (2009)	LCO1490/H2198-Alb	16SLOrc1_fwd/16SLOrc_rev		L
M37-441-01	HNHM 98977	Montenegrina perstriata callistoma	KU307564	KU308036		Albania	Zabzun W		N41.3462	E20.3904	13-Apr-2014	Feher	Geni-Al	LCO1490/H2198-Alb	16SLOrc1_fwd/16SLOrc_rev		I
M38-442-01	HNHM 98978	Montenegrina nana nana	KU307565			Albania	Zabzun SW		N41.3339	E20.3958	13-Apr-2014	Feher	Geni-Al	LCO1490/H2198-Alb			I
M39-445-01	HNHM 98984	Montenegrina perstriata callistoma	KU307566	KU308037		Albania	Klenje N 1.5 km		N41.3792	E20.4687	13-Apr-2014	Feher, Nemeth, Mizsei	Geni-Al	LCO1490/H2198-Alb	16SLOrc1_fwd/16SLOrc_rev		I
M40-448-01	HNHM 98990	Montenegrina perstriata ssp.	KU307567	KU308038		Albania	Gradec		N41.6097	E20.4431	14-Apr-2014	Feher, Mizsei	Geni-Al	LCO1490/H2198-Alb	16SLOrc1_fwd/16SLOrc_rev		H
M41-457-01	HNHM 99019	Montenegrina grammica improvisa	KU307568	KU308039		Albania	Maja e Bastarit, N slope		N41.4361	E20.0485	15-Apr-2014	Feher, Nemeth, Mizsei	Geni-Al	LCO1490/H2198-Alb	16SLOrc1_fwd/16SLOrc_rev		K
M42-473-01	NHMW 111215	Montenegrina rugilabris golikutensis	KU307569	KU308040		Albania	Maja e Golikut, N slope	paratype	N40.2758	E20.0947	28-Jun-2014	Angyal, Feher, Grego	Geni-Al	LCO1490/H2198-Alb	16SLOrc1_fwd/16SLOrc_rev		D1
M42-473-04	NHMW 111215	Montenegrina rugilabris golikutensis	KU307570			Albania	Maja e Golikut, N slope		N40.2758	E20.0947	28-Jun-2014	Angyal, Feher, Grego	Geni-Al	LCO1490/H2198-Alb			D1
M44-479-01	NHMW 111218	Montenegrina hiltrudae selcensis	KU307571	KU308041		Albania	Strelce NE	paratype	N40.748	E20.5219	30-Jun-2014	Feher, Grego	Geni-Al	LCO1490/H2198-Alb	16SLOrc1_fwd/16SLOrc_rev		E
M44-480-01	NHMW 111217	Montenegrina hiltrudae selcensis	KU307572	KU308042	KU307931	Albania	between Strelce and Selce	paratype	N40.7462	E20.5123	30-Jun-2014	Angyal, Eross	Geni-Al	LCO1490/H2198-Alb	16SLOrc1_fwd/16SLOrc_rev	12SGastFw/12SGastRv	E
M45-482-01	HNHM 99641	Montenegrina soosi	KU307573	KU308043		Albania	Tej-Drinit		N41.9423	E20.3593	09-Oct-2005	Deli, Eross, Feher, Muranyi	Thomsen et al. (2009)	LCO1490/H2198-Alb	16SLOrc1_fwd/16SLOrc_rev		L
M46-483-01	HNHM 99640	Montenegrina soosi	KU307574	KU308044		Albania	Drin bridge between Draj-Rec and Zall-Rec		N41.888	E20.3165	09-Oct-2005	Deli, Eross, Feher, Muranyi	Thomsen et al. (2009)	LCO1490/H2198-Alb	16SLOrc1_fwd/16SLOrc_rev		L
M47-484-01	HNHM 99644	Montenegrina soosi	KU307575	KU308045		Albania	Drin bridge between Skavice and Tej-Drinit		N41.9237	E20.354	09-Oct-2005	Deli, Eross, Feher, Muranyi	Thomsen et al. (2009)	LCO1490/H2198-Alb	16SLOrc1_fwd/16SLOrc_rev		L
M48-502-01	HNHM 94918	Montenegrina perstriata subcristatula		KU308046		Macedonia	Nistrovo junction, between Debar and Gostivar		N41.7082	E20.6497	08-Apr-2004	Eross, Feher, Hunyadi	Thomsen et al. (2009)		16SLOrc1_fwd/16SLOrc_rev		L
M49-537-01	NHMW 110430/MN/0040	Montenegrina dofleini dofleini	KU307576	KU308047		Macedonia	Galicica Mts, E of Bugarska Chuka		N41.0019	E20.8523	16-Oct-2014	Feher, Haring, Jaksch, Sattmann	Geni-Al	LCO1490/H2198-Alb	16SLOrc1_fwd/16SLOrc_rev		D2
M49-538-01	NHMW 110430/MN/0041	Montenegrina dofleini dofleini	KU307577	KU308048		Macedonia	Galicica Mts, E of Bugarska Chuka		N41.0033	E20.8488	16-Oct-2014	Feher, Haring, Jaksch, Sattmann	Geni-Al	LCO1490/H2198-Alb	16SLOrc1_fwd/16SLOrc_rev		D2
M49-539-01	NHMW 110430/MN/0042	Montenegrina dofleini dofleini	KU307578	KU308049		Macedonia	Galicica Mts, E of Bugarska Chuka		N41.0037	E20.847	16-Oct-2014	Feher, Haring, Jaksch, Sattmann	Geni-Al	LCO1490/H2198-Alb	16SLOrc1_fwd/16SLOrc_rev		D2
M49-540-01	NHMW 110430/MN/0043	Montenegrina dofleini dofleini	KU307579	KU308050		Macedonia	Galicica Mts, W of Bugarska Chuka		N41.0046	E20.8432	16-Oct-2014	Feher, Haring, Jaksch, Sattmann	Geni-Al	LCO1490/H2198-Alb	16SLOrc1_fwd/16SLOrc_rev		D2
M49-541-01	NHMW 110430/MN/0044	Montenegrina dofleini dofleini	KU307580			Macedonia	Galicica Mts, W of Bugarska Chuka		N41.0051	E20.8462	16-Oct-2014	Feher, Haring, Jaksch, Sattmann	Geni-Al	LCO1490/H2198-Alb			D2
M50-633-01	NHMW 111235	Montenegrina laxa delii	KU307581			Albania	Draj-Rec S	paratype	N41.883	E20.337	02-Jul-2015	Feher, Grego	Geni-Al	LCO1490/H2198-Alb			L
M51-465-01	HNHM 99061	Montenegrina haringae	KU307582	KU308051	KU307932	Albania	Baks-Rrjolle	paratype	N41.8569	E19.5044	04-May-2014	Barina, Pifko, Puskas	Geni-Al	LCO1490/H2198-Alb	16SLOrc1_fwd/16SLOrc_rev	12SGastFw/12SGastRv	F3
M51-602-01	NHMW 111220	Montenegrina haringae	KU307583			Albania	Baks-Rrjolle	paratype	N41.8577	E19.4923	27-May-2015	Deli, Eross, Feher	Geni-Al	LCO1490/H2198-Alb			F3
M52-455-04	HNHM 99011	Montenegrina timeae	KU307584			Albania	Ura e Vashes		N41.4677	E20.1048	15-Apr-2014	Feher, Nemeth, Mizsei	Geni-Al	LCO1490/H2198-Alb			H
M53-618-01	HNHM 99690	Montenegrina hiltrudae desaretica	KU307585			Albania	Glloboceni S 4 km, cave temple	paratype	N40.8422	E20.9616	30-Jun-2015	Eross, Feher, Grego	Geni-Al	LCO1490/H2198-Alb			E
Mam-230-01	HNHM 94866	Montenegrina tomorosi ampla	KU307586	KU308052		Albania	Tomorr Mts, Maja e Ramies S 1 km	paratype	N40.5686	E20.2585	09-Aug-2004	Feher	Geni-Al	LCO1490/H2198-Alb	16SLOrc1_fwd/16SLOrc_rev		K
Mam-230-03	HNHM 94866	Montenegrina tomorosi ampla	KU307587			Albania	Tomorr Mts, Maja e Ramies S 1 km	paratype	N40.5686	E20.2585	09-Aug-2004	Feher	Geni-Al	LCO1490/H2198-Alb			K
Mam-230-04	HNHM 94866	Montenegrina tomorosi ampla	KU307588			Albania	Tomorr Mts, Maja e Ramies S 1 km	paratype	N40.5686	E20.2585	09-Aug-2004	Feher	Geni-Al	LCO1490/H2198-Alb			K
Mam-230-05	HNHM 94866	Montenegrina tomorosi ampla	KU307589			Albania	Tomorr Mts, Maja e Ramies S 1 km	paratype	N40.5686	E20.2585	09-Aug-2004	Feher	Geni-Al	LCO1490/H2198-Alb			K
Map-560-04	NHMW 41166	Montenegrina apfelbecki	KU307590	KU308053		Albania	Mali Shenjt	syntype	N41.85	E20.11	--1905	Buljubasic	Thomsen et al. (2009)	Puzzle	16SLOrc1_fwd/16SLOrc_rev		E
Map-560-05	NHMW 41166	Montenegrina apfelbecki	KU307591	KU308054		Albania	Mali Shenjt	syntype	N41.85	E20.11	--1905	Buljubasic	Thomsen et al. (2009)	Puzzle	16SLOrc1_fwd/16SLOrc_rev		E
Mat-224-01	HNHM 94429	Montenegrina attemsi attemsi	KU307592			Macedonia	Matka Gorge		N41.961	E21.2943	10-Apr-2004	Eross, Feher, Hunyadi	Geni-Al	Puzzle			A
Mat-224-02	HNHM 94429	Montenegrina attemsi attemsi	KU307593	KU308055	KU307933	Macedonia	Matka Gorge		N41.961	E21.2943	10-Apr-2004	Eross, Feher, Hunyadi	Geni-Al	LCO1490/H2198-Alb	16SLOrc1_fwd/16SLOrc_rev	12SGastFw/12SGastRv	A
Mca-495-01	HNHM 94836	Montenegrina okolensis caesia		KU308056		Albania	Qafa Terthores N	paratype	N42.3923	E19.7297	20-Oct-2002	Eross, Feher, Kontschan, Muranyi	Thomsen et al. (2009)		16SLOrc1_fwd/16SLOrc_rev		G
Mca-495-02	HNHM 94836	Montenegrina okolensis caesia	KU307594			Albania	Qafa Terthores N	paratype	N42.3923	E19.7297	20-Oct-2002	Eross, Feher, Kontschan, Muranyi	Thomsen et al. (2009)	LCO1490/H2198-Alb			G
Mce-235-01	HNHM 94909	Montenegrina tomorosi coerulescens	KU307595	KU308057	KU307934	Albania	Tomorr Mts, 6 km N of Maja e Tomorrit		N40.6889	E20.1461	11-Aug-2004	Feher	Geni-Al	LCO1490/H2198-Alb	16SLOrc1_fwd/16SLOrc_rev	12SGastFw/12SGastRv	K
Mci-241-01	HNHM 95051	Montenegrina helvola carinata	KU307596	KU308058		Albania	Greshice S		N40.5356	E19.7964	11-Oct-2004	Feher, Kontschan, Muranyi	Geni-Al	LCO1490/H2198-Alb	16SLOrc1_fwd/16SLOrc_rev		I
Mci-421-01	NHMW 110430/MN/0078	Montenegrina helvola carinata	KU307597	KU308059	KU307935	Albania	Byllis		N40.5447	E19.7389	01-Sep-2013	Reischutz	Geni-Al	LCO1490/H2198-Alb	16SLOrc1_fwd/16SLOrc_rev	12SGastFw/12SGastRv	I
Mci-466-01	HNHM 99618	Montenegrina helvola carinata	KU307598	KU308060		Albania	Pocem		N40.4926	E19.7261	11-May-2014	Barina, Pifko, Puskas	Geni-Al	LCO1490/H2198-Alb	16SLOrc1_fwd/16SLOrc_rev		I
Mck-275-01	HNHM 96845	Montenegrina skipetarica csikii	KU307599			Albania	Bicaj		N41.9897	E20.4202	25-Jun-2007	Danyi, Eross, Feher, Hunyadi, Muranyi	Geni-Al	LCO1490/H2198-Alb			C
Mck-275-03	HNHM 96845	Montenegrina skipetarica csikii	KU307600	KU308061	KU307936	Albania	Bicaj		N41.9897	E20.4202	25-Jun-2007	Danyi, Eross, Feher, Hunyadi, Muranyi	Geni-Al	LCO1490/H2198-Alb	16SLOrc1_fwd/16SLOrc_rev	12SGastFw/12SGastRv	C
Mck-275-04	HNHM 96845	Montenegrina skipetarica csikii	KU307601	KU308062		Albania	Bicaj		N41.9897	E20.4202	25-Jun-2007	Danyi, Eross, Feher, Hunyadi, Muranyi	Geni-Al	LCO1490/H2198-Alb	16SLOrc1_fwd/16SLOrc_rev		C
Mck-275-05	HNHM 96845	Montenegrina skipetarica csikii	KU307602	KU308063		Albania	Bicaj		N41.9897	E20.4202	25-Jun-2007	Danyi, Eross, Feher, Hunyadi, Muranyi	Geni-Al	LCO1490/H2198-Alb	16SLOrc1_fwd/16SLOrc_rev		C
Mck-275-06	HNHM 96845	Montenegrina skipetarica csikii	KU307603			Albania	Bicaj		N41.9897	E20.4202	25-Jun-2007	Danyi, Eross, Feher, Hunyadi, Muranyi	Geni-Al	LCO1490/H2198-Alb			C

Mcl-384-01	HNHM 99561	Montenegrina janinensis	KU307604	KU308064	KU307937	Greece	Perama, cave exit		N39.6971	E20.8433	24-Jun-2013	Eross, Feher, Grego	Geni-Al	LCO1490/H2198-Alb	16SLOrc1_fwd/16SLOrc_rev	12SGastFw/12SGastRv	D1
Mcl-385-01	HNHM 99562	Montenegrina janinensis	KU307605			Greece	Kria		N39.7245	E20.8429	24-Jun-2013	Eross, Feher, Grego	Geni-Al	LCO1490/H2198-Alb			D1
Mco-291-01	HNHM 99612	Montenegrina cattaroensis costata	KU307606	KU308065		Montenegro	Stari Bar 3 km to Mikulici		N42.0973	E19.1458	14-Oct-2008	Danyi, Feher, Kontschan, Muranyi	Geni-Al	LCO1490/H2198-Alb	16SLOrc1_fwd/16SLOrc_rev		F2
Mcr-221-01	HNHM 94449	Montenegrina perstriata drimica	KU307607			Macedonia	Lukovo N 1.5 km		N41.365	E20.605	08-Apr-2004	Eross, Feher, Hunyadi	Geni-Al	LCO1490/H2198-Alb			H
Mcr-221-02	HNHM 94449	Montenegrina perstriata drimica	KU307608			Macedonia	Lukovo N 1.5 km		N41.365	E20.605	08-Apr-2004	Eross, Feher, Hunyadi	Geni-Al	LCO1490/H2198-Alb			H
Mct-286-01	HNHM 99599	Montenegrina cattaroensis cattaroensis	KU307616	KU308072	KU307939	Montenegro	Dobrota N, Ljuta Spring		N42.4862	E18.767	08-Oct-2008	Danyi, Feher, Kontschan, Muranyi	Geni-Al	LCO1490/H2198-Alb	16SLOrc1_fwd/16SLOrc_rev	12SGastFw/12SGastRv	F2
Mct-568-02	HNHM 43127	Montenegrina cattaroensis cattaroensis	KU307617			Montenegro	Kotor, above the city		N42.424	E18.774	09-Jul-1985	Kiss, Pinter	Thomsen et al. (2009)	LCO1490/H2198-Alb			F2
Mct-568-03	HNHM 43127	Montenegrina cattaroensis cattaroensis	KU307618			Montenegro	Kotor, above the city		N42.424	E18.774	09-Jul-1985	Kiss, Pinter	Thomsen et al. (2009)	LCO1490/H2198-Alb			F2
Mct-568-04	HNHM 43127	Montenegrina cattaroensis cattaroensis	KU307619			Montenegro	Kotor, above the city		N42.424	E18.774	09-Jul-1985	Kiss, Pinter	Thomsen et al. (2009)	LCO1490/H2198-Alb			F2
Mct-570-01	HNHM 95412	Montenegrina cattaroensis cattaroensis	KU307620			Montenegro	Kotor 8 km to Njegusi		N42.4035	E18.7753	21-Apr-2000	Eross, Feher	Thomsen et al. (2009)	LCO1490/H2198-Alb			F2
Mct-570-02	HNHM 95412	Montenegrina cattaroensis cattaroensis	KU307621			Montenegro	Kotor 8 km to Njegusi		N42.4035	E18.7753	21-Apr-2000	Eross, Feher	Thomsen et al. (2009)	LCO1490/H2198-Alb			F2
Mct-606-01	NHMW 110430/MN/0146	Montenegrina cattaroensis cattaroensis	KU307622			Montenegro	Kotor		N42.4269	E18.7756	29-May-2015	Deli, Eross, Feher	Geni-Al	LCO1490/H2198-Alb			F2
Mct-607-01	HNHM 110430/MN/0147	Montenegrina cattaroensis cattaroensis	KU307623			Montenegro	Kotor 8 km to Njegusi		N42.4022	E18.7751	29-May-2015	Deli, Eross, Feher	Geni-Al	LCO1490/H2198-Alb			F2
Mct-608-01	HNHM 110430/MN/0148	Montenegrina cattaroensis cattaroensis	KU307624			Montenegro	Kotor 9 km to Njegusi		N42.4095	E18.7787	29-May-2015	Deli, Eross, Feher	Geni-Al	LCO1490/H2198-Alb			F2
Mct-643-01	NHMW 110430/MN/0161	Montenegrina cattaroensis cattaroensis	KU307625			Montenegro	Orahovac		N42.4925	E18.7556	09-Jul-2015	Duda, Haring, Jaksch, Sattmann	Geni-Al	LCO1490/H2198-Alb			F2
Mcu-407-01	HNHM 99590	Montenegrina hiltrudae costulata	KU307626	KU308073	KU307940	Albania	Qafa e Zvezdes		N40.733	E20.8729	29-Jun-2013	Eross, Feher, Grego	Geni-Al	LCO1490/H2198-Alb	16SLOrc1_fwd/16SLOrc_rev	12SGastFw/12SGastRv	E
Mcs-365-01	HNHM 99623	Montenegrina perstriata callistoma	KU307609			Albania	between Klenje and Stebleve		N41.3575	E20.4623	31-May-2013	Juhasz, Kovacs, Magos, Puskas	Geni-Al	LCO1490/H2198-Alb			I
Mcs-433-01	HNHM 98953	Montenegrina perstriata callistoma	KU307610	KU308066		Albania	Funares W		N41.2719	E20.2839	12-Apr-2014	Feher, Nemeth, Mizsei	Geni-Al	LCO1490/H2198-Alb	16SLOrc1_fwd/16SLOrc_rev		I
Mcs-434-01	HNHM 98954	Montenegrina perstriata callistoma	KU307611	KU308067	KU307938	Albania	Funares W 3.5 km		N41.2748	E20.2679	12-Apr-2014	Feher, Nemeth, Mizsei	Geni-Al	LCO1490/H2198-Alb	16SLOrc1_fwd/16SLOrc_rev	12SGastFw/12SGastRv	I
Mcs-443-01	HNHM 98980	Montenegrina perstriata callistoma	KU307612	KU308068		Albania	Stebleve S 1.5 km		N41.325	E20.4457	13-Apr-2014	Feher, Nemeth, Mizsei	Geni-Al	LCO1490/H2198-Alb	16SLOrc1_fwd/16SLOrc_rev		I
Mcs-444-01	HNHM 98982	Montenegrina perstriata callistoma	KU307613	KU308069		Albania	Klenje		N41.3646	E20.4685	13-Apr-2014	Feher, Nemeth, Mizsei	Geni-Al	LCO1490/H2198-Alb	16SLOrc1_fwd/16SLOrc_rev		I
Mcs-446-01	HNHM 98986	Montenegrina perstriata callistoma	KU307614	KU308070		Albania	Ostreni i Madh S 1.2 km		N41.4199	E20.4633	14-Apr-2014	Feher, Nemeth, Mizsei	Geni-Al	LCO1490/H2198-Alb	16SLOrc1_fwd/16SLOrc_rev		I
Mcs-447-01	HNHM 98987	Montenegrina perstriata callistoma	KU307615	KU308071		Albania	Tucep		N41.4404	E20.5052	14-Apr-2014	Feher, Nemeth, Mizsei	Geni-Al	LCO1490/H2198-Alb	16SLOrc1_fwd/16SLOrc_rev		I
Mdc-400-01	HNHM 99584	Montenegrina hiltrudae densicostulata	KU307627	KU308074	KU307941	Greece	between Vogatsiko and Driovouno		N40.3782	E21.416	28-Jun-2013	Eross, Feher, Grego	Geni-Al	LCO1490/H2198-Alb	16SLOrc1_fwd/16SLOrc_rev	12SGastFw/12SGastRv	E
Mdd-360-01		Montenegrina laxa dedovi	KU307628	KU308075		Macedonia	Gorna Belitsa		N41.22	E20.54	10-Jul-2009	Dedov, Minkov	Geni-Al	LCO1490/H2198-Alb	16SLOrc1_fwd/16SLOrc_rev		L
Mde-204-02	HNHM 94830	Montenegrina drimmeri	KU307629	KU308076		Albania	Lunare		N41.6257	E20.2499	26-Jun-2003	Eross, Feher, Kontschan, Muranyi	Geni-Al	LCO1490/H2198-Alb	16SLOrc1_fwd/16SLOrc_rev		L
Mde-300-02	HNHM 99636	Montenegrina drimmeri	KU307630	KU308077		Albania	Lunare		N41.6256	E20.2498	18-May-2010	Feher, Muranyi, Ujvari	Thomsen et al. (2009)	LCO1490/H2198-Alb	16SLOrc1_fwd/16SLOrc_rev		L
Mdj-449-01	HNHM 98995	Montenegrina laxa disjuncta	KU307631	KU308078		Albania	Selishte E 4 km		N41.6308	E20.2983	14-Apr-2014	Feher, Mizsei	Geni-Al	LCO1490/H2198-Alb	16SLOrc1_fwd/16SLOrc_rev		L
Mdm-215-01	HNHM 94431	Montenegrina perstriata diminuta	KU307632	KU308079		Macedonia	Zavoj junction E 1 km, between Ohrid and Resen		N41.1978	E20.9173	07-Apr-2004	Eross, Feher, Hunyadi	Geni-Al	LCO1490/H2198-Alb	16SLOrc1_fwd/16SLOrc_rev		L
Mdm-533-01	NHMW 110430/MN/0101	Montenegrina perstriata diminuta	KU307633			Macedonia	Recica		N41.2202	E20.9115	15-Oct-2014	Feher, Haring, Jaksch, Sattmann	Geni-Al	LCO1490/H2198-Alb			L
Mdm-534-01	NHMW 110430/MN/0102	Montenegrina perstriata diminuta	KU307634		KU307942	Macedonia	Zavoj junction E 1 km, between Ohrid and Resen		N41.1978	E20.9173	15-Oct-2014	Feher, Haring, Jaksch, Sattmann	Geni-Al	LCO1490/H2198-Alb		12SGastFw/12SGastRv	L
Mdn-227-01	HNHM 95393	Montenegrina hiltrudae dennisi	KU307635	KU308080	KU307943	Greece	Zakas E 3 km		N40.0422	E21.2823	15-Jul-2004	Eross, Hunyadi	Geni-Al	LCO1490/H2198-Alb	16SLOrc1_fwd/16SLOrc_rev	12SGastFw/12SGastRv	E
Mdn-368-01	HNHM 99592	Montenegrina hiltrudae dennisi	KU307636	KU308081	KU307944	Greece	Spileo W 0.5 km to Portitsa Farangi		N40.0077	E21.2805	22-Jun-2013	Eross, Feher, Grego	Geni-Al	LCO1490/H2198-Alb	16SLOrc1_fwd/16SLOrc_rev	12SGastFw/12SGastRv	E
Mdn-369-01	HNHM 99591	Montenegrina hiltrudae dennisi	KU307637			Greece	Spileo N 0.5 km to Zakas		N40.008	E21.2847	22-Jun-2013	Eross, Feher, Grego	Geni-Al	LCO1490/H2198-Alb			E
Mdo-272-01		Montenegrina dofleini ssp.	KU307638	KU308082		Albania	Thate Mts, ENE of Buz e Korutes		N40.8024	E20.8741	22-May-2007	Barina, Pifko	Geni-Al	LCO1490/H2198-Alb	16SLOrc1_fwd/16SLOrc_rev		D2
Mdo-272-02		Montenegrina dofleini ssp.	KU307639			Albania	Thate Mts, ENE of Buz e Korutes		N40.8024	E20.8741	22-May-2007	Barina, Pifko	Thomsen et al. (2009)	LCO1490/H2198-Alb			D2
Mdo-272-03		Montenegrina dofleini ssp.	KU307640			Albania	Thate Mts, ENE of Buz e Korutes		N40.8024	E20.8741	22-May-2007	Barina, Pifko	Thomsen et al. (2009)	LCO1490/H2198-Alb			D2
Mdo-544-01	NHMW 110430/MN/0045	Montenegrina dofleini dofleini	KU307641	KU308083	KU307945	Macedonia	Elsani		N41.0345	E20.8105	17-Oct-2014	Feher, Jaksch, Sattmann	Geni-Al	LCO1490/H2198-Alb	16SLOrc1_fwd/16SLOrc_rev	12SGastFw/12SGastRv	D2
Mdo-613-01	HNHM 99567	Montenegrina dofleini dofleini	KU307642			Macedonia	Galicica Mts, Magaro Peak N		N40.9445	E20.827	29-Jun-2015	Eross, Feher, Grego	Geni-Al	LCO1490/H2198-Alb			D2
Mdo-614-01	HNHM 99568	Montenegrina dofleini dofleini	KU307643			Macedonia	Galicica Mts, Magaro Peak N		N40.9439	E20.8283	29-Jun-2015	Feher	Geni-Al	LCO1490/H2198-Alb			D2
Mdo-615-01	HNHM 99569	Montenegrina dofleini dofleini	KU307644			Macedonia	Galicica Mts, Magaro Peak N		N40.9429	E20.8252	29-Jun-2015	Grego	Geni-Al	LCO1490/H2198-Alb			D2
Mdr-217-01	HNHM 94448	Montenegrina perstriata drimica		KU308085		Macedonia	Lukovo S 3.7 km		N41.3302	E20.6372	08-Apr-2004	Eross, Feher, Hunyadi	Geni-Al		16SLOrc1_fwd/16SLOrc_rev		H
Mdr-218-01	HNHM 94447	Montenegrina perstriata drimica	KU307645	KU308086	KU307946	Macedonia	Lukovo S 3 km, Brana Globocica		N41.3355	E20.6345	08-Apr-2004	Eross, Feher, Hunyadi	Geni-Al	LCO1490/H2198-Alb	16SLOrc1_fwd/16SLOrc_rev	12SGastFw/12SGastRv	K
Mdr-218-03	HNHM 94447	Montenegrina perstriata drimica		KU308087		Macedonia	Lukovo S 3 km, Brana Globocica		N41.3355	E20.6345	08-Apr-2004	Eross, Feher, Hunyadi	Geni-Al		16SLOrc1_fwd/16SLOrc_rev		H
Mdr-219-01	HNHM 94446	Montenegrina perstriata drimica		KU308088		Macedonia	Lukovo S 2 km		N41.3407	E20.6267	08-Apr-2004	Eross, Feher, Hunyadi	Geni-Al		16SLOrc1_fwd/16SLOrc_rev		K
Mdr-220-02	HNHM 94445	Montenegrina perstriata drimica		KU308089		Macedonia	Lukovo S		N41.3547	E20.6123	08-Apr-2004	Eross, Feher, Hunyadi	Geni-Al		16SLOrc1_fwd/16SLOrc_rev		K
Mdr-220-04	HNHM 94445	Montenegrina perstriata drimica			KU307947	Macedonia	Lukovo S		N41.3547	E20.6123	08-Apr-2004	Eross, Feher, Hunyadi	Geni-Al			12SGastFw/12SGastRv	K
Mdr-505-01	HNHM 94589	Montenegrina perstriata drimica		KU308090		Macedonia	Lukovo N 2 km		N41.3695	E20.601	08-Apr-2004	Eross, Feher, Hunyadi	Thomsen et al. (2009)		16SLOrc1_fwd/16SLOrc_rev		H
Mdr-520-01	NHMW 110430/MN/0088	Montenegrina perstriata drimica	KU307646	KU308091	KU307948	Macedonia	Lukovo S 3.7 km		N41.3299	E20.6369	15-Oct-2014	Feher, Haring, Jaksch, Sattmann	Geni-Al	LCO1490/H2198-Alb	16SLOrc1_fwd/16SLOrc_rev	12SGastFw/12SGastRv	K
Mdr-521-01	NHMW 110430/MN/0089	Montenegrina perstriata drimica		KU308092	KU307949	Macedonia	Lukovo S 2 km		N41.3397	E20.6273	15-Oct-2014	Feher, Haring, Jaksch, Sattmann	Geni-Al		16SLOrc1_fwd/16SLOrc_rev	12SGastFw/12SGastRv	H
Mdr-523-01	NHMW 110430/MN/0091	Montenegrina perstriata drimica		KU308093		Macedonia	Lukovo S		N41.3545	E20.6124	15-Oct-2014	Feher, Haring, Jaksch, Sattmann	Geni-Al		16SLOrc1_fwd/16SLOrc_rev		H
Mdr-524-01	NHMW 110430/MN/0092	Montenegrina perstriata drimica	KU307647	KU308094		Macedonia	Lukovo N 1 km		N41.3623	E20.6036	15-Oct-2014	Feher, Haring, Jaksch, Sattmann	Geni-Al	LCO1490/H2198-Alb	16SLOrc1_fwd/16SLOrc_rev		H
Mdr-524-02	NHMW 110430/MN/0092	Montenegrina perstriata drimica	KU307648			Macedonia	Lukovo N 1 km		N41.3623	E20.6036	15-Oct-2014	Feher, Haring, Jaksch, Sattmann	Geni-Al	LCO1490/H2198-Alb			H
Mdr-525-01	NHMW 110430/MN/0093	Montenegrina perstriata drimica	KU307649			Macedonia	Lukovo N 1.3 km		N41.3638	E20.6049	15-Oct-2014	Feher, Haring, Jaksch, Sattmann	Geni-Al	LCO1490/H2198-Alb			H
Mdr-525-02	NHMW 110430/MN/0093	Montenegrina perstriata drimica	KU307650	KU308095		Macedonia	Lukovo N 1.3 km		N41.3638	E20.6049	15-Oct-2014	Feher, Haring, Jaksch, Sattmann	Geni-Al	LCO1490/H2198-Alb	16SLOrc1_fwd/16SLOrc_rev		H
Mdr-526-01	NHMW 110430/MN/0094	Montenegrina perstriata drimica	KU307651			Macedonia	Lukovo N 1.5 km		N41.3666	E20.6049	15-Oct-2014	Feher, Haring, Jaksch, Sattmann	Geni-Al	LCO1490/H2198-Alb			H
Mdr-526-02	NHMW 110430/MN/0094	Montenegrina perstriata drimica	KU307652			Macedonia	Lukovo N 1.5 km		N41.3666	E20.6049	15-Oct-2014	Feher, Haring, Jaksch, Sattmann	Geni-Al	LCO1490/H2198-Alb			H
Mdr-527-01	NHMW 110430/MN/0095	Montenegrina perstriata drimica	KU307653			Macedonia	Lukovo N 1.7 km		N41.3672	E20.6046	15-Oct-2014	Feher, Haring, Jaksch, Sattmann	Geni-Al	LCO1490/H2198-Alb			H
Mdr-528-01	NHMW 110430/MN/0096	Montenegrina perstriata drimica	KU307654	KU308096		Macedonia	Lukovo N 2 km		N41.3695	E20.6021	15-Oct-2014	Feher, Haring, Jaksch, Sattmann	Geni-Al	LCO1490/H2198-Alb	16SLOrc1_fwd/16SLOrc_rev		H
Mdr-529-01	NHMW 110430/MN/0097	Montenegrina perstriata drimica	KU307655			Macedonia	Lukovo N 2.4 km		N41.3705	E20.5979	15-Oct-2014	Feher, Haring, Jaksch, Sattmann	Geni-Al	LCO1490/H2198-Alb			H
Mdr-530-02	NHMW 110430/MN/0098	Montenegrina perstriata drimica	KU307656			Macedonia	Lukovo N 2.5 km		N41.3713	E20.5971	15-Oct-2014	Feher, Haring, Jaksch, Sattmann	Geni-Al	LCO1490/H2198-Alb			H
Mdr-531-02	NHMW 110430/MN/0099	Montenegrina perstriata drimica	KU307657			Macedonia	Lukovo N 6 km		N41.3978	E20.5899	15-Oct-2014	Feher, Haring, Jaksch, Sattmann	Geni-Al	LCO1490/H2198-Alb			H
Mdr-532-01	NHMW 110430/MN/0100	Montenegrina perstriata drimica	KU307658			Macedonia	Lukovo N 4 km		N41.3816	E20.5881	15-Oct-2014	Feher, Haring, Jaksch, Sattmann	Geni-Al	LCO1490/H2198-Alb			H
Mek-284-01	HNHM 99533	Montenegrina skipetarica ersekensis	KU307659			Albania	Barmash S 4.5 km, between Leskovik and Korce		N40.2688	E20.6065	17-Aug-2007	Feher, Tamas	Geni-Al	LCO1490/H2198-Alb			C
Mek-477-01	NHMW 99375	Montenegrina skipetarica ersekensis	KU307660	KU308097	KU307950	Albania	Barmash S 4 km, between Leskovik and Korce		N40.2656	E20.6001	29-Jun-2014	Angyal, Eross, Feher, Grego	Geni-Al	LCO1490/H2198-Alb	16SLOrc1_fwd/16SLOrc_rev	12SGastFw/12SGastRv	C
Mem-387-01	HNHM 99697	Montenegrina rugilabris edmundi	KU307661	KU308098	KU307951	Greece	Lista SW 2 km to Lia		N39.7358	E20.4537	25-Jun-2013	Eross, Feher, Grego	Geni-Al	LCO1490/H2198-Alb	16SLOrc1_fwd/16SLOrc_rev	12SGastFw/12SGastRv	D1
Mem-387-02	HNHM 99697	Montenegrina rugilabris edmundi	KU307662			Greece	Lista SW 2 km to Lia		N39.7358	E20.4537	25-Jun-2013	Eross, Feher, Grego	Geni-Al	LCO1490/H2198-Alb			D1
Mem-387-03	HNHM 99697	Montenegrina rugilabris edmundi	KU307663			Greece	Lista SW 2 km to Lia		N39.7358	E20.4537	25-Jun-2013	Eross, Feher, Grego	Geni-Al	LCO1490/H2198-Alb			D1
Mer-410-01	HNHM 99631	Montenegrina laxa errans	KU307664	KU308099		Albania	Cervenake		N40.9444	E20.6109	29-Jun-2013	Eross, Feher, Grego	Geni-Al	LCO1490/H2198-Alb	16SarF/16SLOrc_rev		L
Mer-431-01	HNHM 98950	Montenegrina laxa errans	KU307665	KU308100		Albania	Bishnice SW 4 km		N40.9211	E20.4492	12-Apr-2014	Feher, Nemeth, Mizsei	Geni-Al	LCO1490/H2198-Alb	16SLOrc1_fwd/16SLOrc_rev		L
Mer-432-01	HNHM 98952	Montenegrina laxa errans	KU307666	KU308101		Albania	Stravaj		N41.0042	E20.4343	12-Apr-2014	Feher, Nemeth, Mizsei	Geni-Al	LCO1490/H2198-Alb	16SLOrc1_fwd/16SLOrc_rev		L
Mfa-211-04	HNHM 94450	Montenegrina dofleini fagorum	KU307667	KU308102		Macedonia	Carina W 15.6 km to Trpejca		N40.9625	E20.817	06-Apr-2004	Eross, Feher, Hunyadi	Thomsen et al. (2009)	LCO1490/H2198-Alb	16SLOrc1_fwd/16SLOrc_rev		D2
Mfa-507-01	NHMW 110430/MN/0052	Montenegrina dofleini fagorum	KU307668	KU308103		Macedonia	Galicica Mts, 12 km from the Sv. Naum to Ohrid main road		N40.9627	E20.8171	08-Aug-2014	Feher, Tamas	Geni-Al	LCO1490/H2198-Alb	16SLOrc1_fwd/16SLOrc_rev		D2
Mfa-612-01	NHMW 110430/MN/0054	Montenegrina dofleini fagorum	KU307669			Macedonia	Galicica Mts, Baba observation point		N40.9541	E20.8126	29-Jun-2015	Eross, Feher, Grego	Geni-Al	LCO1490/H2198-Alb			D2
Mfc-209-01	HNHM 94896	Montenegrina hiltrudae fusca	KU307670			Albania	Korit e Bregas	paratype	N40.7693	E20.8305	02-Jul-2003	Eross, Feher, Kontschan, Muranyi	Geni-Al	LCO1490/H2198-Alb			E
Mfc-409-01	HNHM 99596	Montenegrina hiltrudae fusca	KU307671	KU308104	KU307952	Albania	Korit e Bregas		N40.7688	E20.8275	29-Jun-2013	Eross, Feher, Grego	Geni-Al	LCO1490/H2198-Alb	16SLOrc1_fwd/16SLOrc_rev	12SGastFw/12SGastRv	E
Mfl-428-01	HNHM 99544	Montenegrina skipetarica flava	KU307672	KU308105		Albania	Gjinikas		N40.692	E20.5002	16-Oct-2013	Juhasz, Kovacs, Muranyi, Puskas	Geni-Al	LCO1490/H2198-Alb	16SLOrc1_fwd/16SLOrc_rev		C
Mfl-481-01	NHMW 99545	Montenegrina skipetarica flava	KU307673	KU308106		Albania	Gjinikas		N40.6921	E20.5003	30-Jun-2014	Angyal, Eross, Feher, Grego	Geni-Al	LCO1490/H2198-Alb	16SLOrc1_fwd/16SLOrc_rev		C
Mfu-426-01	HNHM 99563	Montenegrina fuchsi fuchsi	KU307674	KU308107	KU307953	Albania	Luadh SW, between Kelcyre and Tepelene		N40.2971	E20.1082	14-Oct-2013	Juhasz, Kovacs, Muranyi, Puskas	Geni-Al	LCO1490/H2198-Alb	16SLOrc1_fwd/16SLOrc_rev	12SGastFw/12SGastRv	D1
Mfu-474-01	HNHM 99333	Montenegrina fuchsi fuchsi	KU307675	KU308108		Albania	Kelcyre W 7 km, Peshtan junction		N40.2961	E20.1076	29-Jun-2014	Angyal, Eross, Feher, Grego	Geni-Al	LCO1490/H2198-Alb	16SLOrc1_fwd/16SLOrc_rev		D1
Mgc-277-01	HNHM 99624	Montenegrina nana gracilis	KU307676	KU308109		Albania	Bulqize E 10 km		N41.5099	E20.3152	30-Jun-2007	Danyi, Eross, Feher, Hunyadi, Muranyi	Geni-Al	LCO1490/H2198-Alb	16SLOrc1_fwd/16SLOrc_rev		I
Mgc-277-02	HNHM 99624	Montenegrina nana gracilis	KU307677	KU308110		Albania	Bulqize E 10 km		N41.5099	E20.3152	30-Jun-2007	Danyi, Eross, Feher, Hunyadi, Muranyi	Geni-Al	LCO1490/H2198-Alb	16SLOrc1_fwd/16SLOrc_rev		I
Mgc-277-03	HNHM 99624	Montenegrina nana gracilis	KU307678	KU308111	KU307954	Albania	Bulqize E 10 km		N41.5099	E20.3152	30-Jun-2007	Danyi, Eross, Feher, Hunyadi, Muranyi	Geni-Al	LCO1490/H2198-Alb	16SLOrc1_fwd/16SLOrc_rev	12SGastFw/12SGastRv	I
Mgm-395-01	HNHM 99628	Montenegrina grammica grammica	KU307679	KU308112	KU307955	Greece	Gramos Mts, W of Epano Arena		N40.309	E20.8981	27-Jun-2013	Eross, Feher, Grego	Geni-Al	LCO1490/H2198-Alb	16SLOrc1_fwd/16SLOrc_rev	12SGastFw/12SGastRv	K
Mhe-415-01	NHMW 110430/MN/0076	Montenegrina helvola helvola	KU307680	KU308113	KU307956	Albania	Kruje		N41.522	E19.7939	01-Sep-2013	Reischutz	Geni-Al	LCO1490/H2198-Alb	16SLOrc1_fwd/16SLOrc_rev	12SGastFw/12SGastRv	I
Mhe-493-01	HNHM 91606	Montenegrina helvola helvola	KU307681			Albania	Noi		N41.5229	E19.8165	26-Oct-2002	Eross, Feher, Kontschan, Muranyi	Thomsen et al. (2009)	LCO1490/H2198-Alb			I
Mhi-399-01	HNHM 99582	Montenegrina hiltrudae densicostulata	KU307682	KU308114	KU307957	Greece	Germas W 1.5 km		N40.443	E21.4111	28-Jun-2013	Eross, Feher, Grego	Geni-Al	LCO1490/H2198-Alb	16SLOrc1_fwd/16SLOrc_rev	12SGastFw/12SGastRv	E
Mhi-578-02	SMNS N5476	Montenegrina hiltrudae hiltrudae	KU307683			Greece	Vogatsikon near Kastoria	paratype	N0	E0	23-Aug-1971	Nordsieck	Geni-Al	LCO1490/H2198-Alb			E
Mib-463-01	HNHM 99036	Montenegrina laxa iba	KU307684	KU308115		Albania	Ibe, Erzen gorge		N41.2598	E19.9676	17-Apr-2014	Feher	Geni-Al	LCO1490/H2198-Alb	16SLOrc1_fwd/16SLOrc_rev		L
Mir-372-01	HNHM 99554	Montenegrina rugilabris irmengardis	KU307685	KU308116	KU307958	Greece	Platanoussa		N39.4279	E21.0106	23-Jun-2013	Eross, Feher, Grego	Geni-Al	LCO1490/H2198-Alb	16SLOrc1_fwd/16SLOrc_rev	12SGastFw/12SGastRv	D1
Mir-372-02	HNHM 99554	Montenegrina rugilabris irmengardis	KU307686	KU308117		Greece	Platanoussa		N39.4279	E21.0106	23-Jun-2013	Eross, Feher, Grego	Geni-Al	LCO1490/H2198-Alb	16SLOrc1_fwd/16SLOrc_rev		D1
Mir-376-01	HNHM 99552	Montenegrina rugilabris irmengardis	KU307687			Greece	Kedros W 2 km to Charakopi		N39.5728	E20.9861	24-Jun-2013	Eross, Feher, Grego	Geni-Al	LCO1490/H2198-Alb			D1
Mir-376-02	HNHM 99552	Montenegrina rugilabris irmengardis	KU307688			Greece	Kedros W 2 km to Charakopi		N39.5728	E20.9861	24-Jun-2013	Eross, Feher, Grego	Geni-Al	LCO1490/H2198-Alb			D1
Mir-377-01	HNHM 99553	Montenegrina rugilabris irmengardis	KU307689			Greece	Charakopi E 4 km		N39.5763	E20.9772	24-Jun-2013	Eross, Feher, Grego	Geni-Al	LCO1490/H2198-Alb			D1
Mir-377-02	HNHM 99553	Montenegrina rugilabris irmengardis	KU307690			Greece	Charakopi E 4 km		N39.5763	E20.9772	24-Jun-2013	Eross, Feher, Grego	Geni-Al	LCO1490/H2198-Alb			D1
Mjk-425-01	HNHM 99520	Montenegrina attemsi jakupicensis	KU307691	KU308118	KU307959	Macedonia	Nezilovo, Babuna Spring		N41.6903	E21.4162	03-Oct-2013	Muranyi, Kovacs	Geni-Al	LCO1490/H2198-Alb	16SLOrc1_fwd/16SLOrc_rev	12SGastFw/12SGastRv	A
Mjk-425-02	HNHM 99520	Montenegrina attemsi jakupicensis	KU307692			Macedonia	Nezilovo, Babuna Spring		N41.6903	E21.4162	03-Oct-2013	Muranyi, Kovacs	Geni-Al	LCO1490/H2198-Alb			A

Mjk-425-03	HNHM 99520	Montenegrina attemsi jakupicensis	KU307693	KU308119		Macedonia	Nezilovo, Babuna Spring		N41.6903	E21.4162	03-Oct-2013	Muranyi, Kovacs	Geni-Al	LCO1490/H2198-Alb	16SLOrc1_fwd/16SLOrc_rev		A
Mjn-382-01	HNHM 99559	Montenegrina janinensis	KU307694			Greece	Ligkiades junction E 2 km, between Amfithea and Spothi		N39.6802	E20.9052	24-Jun-2013	Eross, Feher, Grego	Geni-Al	LCO1490/H2198-Alb			D1
Mjn-383-01	HNHM 99560	Montenegrina janinensis	KU307695	KU308120	KU307960	Greece	Perama, cave entrance		N39.6947	E20.8463	24-Jun-2013	Eross, Feher, Grego	Geni-Al	LCO1490/H2198-Alb	16SLOrc1_fwd/16SLOrc_rev	12SGastFw/12SGastRv	D1
Mkl-472-01	HNHM 99272	Montenegrina fuchsi klemmi		KU308121		Albania	Suhe NE 3 km to Sheper		N40.088	E20.2716	27-Jun-2014	Angyal, Eross, Feher, Grego	Geni-Al		16SLOrc1_fwd/16SLOrc_rev		D1
Mkn-393-01	HNHM 99528	Montenegrina skipetarica konitsae	KU307696	KU308122		Greece	Konitsa		N40.0355	E20.7469	26-Jun-2013	Eross, Feher, Grego	Geni-Al	LCO1490/H2198-Alb	16SLOrc1_fwd/16SLOrc_rev		C
Mkn-394-01	HNHM 99527	Montenegrina skipetarica konitsae	KU307697			Greece	Sarantaporos Gorge near Exochi		N40.1102	E20.7203	26-Jun-2013	Eross, Feher, Grego	Geni-Al	LCO1490/H2198-Alb			C
Mko-240-01	HNHM 94881	Montenegrina laxa kontschani	KU307698			Albania	Qafa Shtylles		N41.3711	E20.0855	09-Oct-2004	Feher, Kontschan, Muranyi	Geni-Al	LCO1490/H2198-Alb			L
Mko-240-02	HNHM 94881	Montenegrina laxa kontschani		KU308123		Albania	Qafa Shtylles		N41.3711	E20.0855	09-Oct-2004	Feher, Kontschan, Muranyi	Geni-Al		16SLOrc1_fwd/16SLOrc_rev		L
Mko-313-01	HNHM 99633	Montenegrina laxa kontschani	KU307699	KU308124	KU307961	Albania	Shen Meri junction E 3 km between Tirana and Bize		N41.3512	E20.0502	20-Jun-2012	Feher, Kovacs, Muranyi	Geni-Al	LCO1490/H2198-Alb	16SLOrc1_fwd/16SLOrc_rev	12SGastFw/12SGastRv	L
Mko-314-01	HNHM 99634	Montenegrina laxa kontschani	KU307700	KU308125	KU307962	Albania	Shen Meri junction E 3.5 km between Tirana and Bize		N41.3507	E20.0583	20-Jun-2012	Feher, Kovacs, Muranyi	Geni-Al	LCO1490/H2198-Alb	16SLOrc1_fwd/16SLOrc_rev	12SGastFw/12SGastRv	L
Mks-401-01	HNHM 99570	Montenegrina dofleini kastoriae	KU307701	KU308126		Greece	Kastoria		N40.5223	E21.2745	28-Jun-2013	Eross, Feher, Grego	Geni-Al	LCO1490/H2198-Alb	16SLOrc1_fwd/16SLOrc_rev		D2
Mks-401-02	HNHM 99570	Montenegrina dofleini kastoriae	KU307702			Greece	Kastoria		N40.5223	E21.2745	28-Jun-2013	Eross, Feher, Grego	Geni-Al	LCO1490/H2198-Alb			D2
Mks-401-03	HNHM 99570	Montenegrina dofleini kastoriae	KU307703			Greece	Kastoria		N40.5223	E21.2745	28-Jun-2013	Eross, Feher, Grego	Geni-Al	LCO1490/H2198-Alb			D2
Mks-548-01	NHMW 110430/MN/0050	Montenegrina dofleini kastoriae	KU307704			Greece	Kastoria		N40.5233	E21.2747	17-Oct-2014	Feher, Haring, Jaksch, Sattmann	Geni-Al	LCO1490/H2198-Alb			D2
Mlk-559-01	NHMW 102868	Montenegrina laxa lakmosensis	KU307705			Greece	Peristeri summit S		N39.681	E21.122	--1988	Sattmann	Thomsen et al. (2009)	LCO1490/H2198-Alb			L
Mlk-559-02	NHMW 102868	Montenegrina laxa lakmosensis		KU308127		Greece	Peristeri summit S		N39.681	E21.122	--1988	Sattmann	Thomsen et al. (2009)		16SLOrc1_fwd/16SLOrc_rev		L
Mlm-386-01	HNHM 99555	Montenegrina rugilabris lambdaformis	KU307706	KU308128		Greece	Lithino, 1 km N of Pateron Monastery		N39.772	E20.618	25-Jun-2013	Eross, Feher, Grego	Geni-Al	LCO1490/H2198-Alb	16SLOrc1_fwd/16SLOrc_rev		D1
Mlx-462-01	HNHM 99035	Montenegrina laxa laxa	KU307707	KU308129		Albania	Ferraj NE 5 km		N41.3948	E19.9	16-Apr-2014	Feher, Nemeth, Mizsei	Geni-Al	LCO1490/H2198-Alb	16SLOrc1_fwd/16SLOrc_rev		L
Mma-367-01	HNHM 99595	Montenegrina hiltrudae maasseni	KU307708	KU308130		Greece	Portitsa Farangi		N39.9965	E21.2855	22-Jun-2013	Eross, Feher, Grego	Geni-Al	LCO1490/H2198-Alb	16SLOrc1_fwd/16SLOrc_rev		E
Mma-367-02	HNHM 99595	Montenegrina hiltrudae maasseni	KU307709	KU308131		Greece	Portitsa Farangi		N39.9965	E21.2855	22-Jun-2013	Eross, Feher, Grego	Geni-Al	LCO1490/H2198-Alb	16SLOrc1_fwd/16SLOrc_rev		E
Mma-367-04	HNHM 99595	Montenegrina hiltrudae maasseni		KU308132		Greece	Portitsa Farangi		N39.9965	E21.2855	22-Jun-2013	Eross, Feher, Grego	Geni-Al		16SLOrc1_fwd/16SLOrc_rev		E
Mmg-281-01	HNHM 99619	Montenegrina helvola magna	KU307710			Albania	Between Fshat and Shkalle		N41.4794	E20.0815	27-May-2008	Barina, Pifko, Pinter	Geni-Al	LCO1490/H2198-Alb			I
Mmg-435-01	HNHM 98962	Montenegrina helvola magna	KU307711	KU308133		Albania	Orenje		N41.2802	E20.21	13-Apr-2014	Feher, Mizsei	Geni-Al	LCO1490/H2198-Alb	16SLOrc1_fwd/16SLOrc_rev		I
Mmg-455-01	HNHM 99010	Montenegrina helvola magna		KU308134		Albania	Ura e Vashes		N41.4677	E20.1048	15-Apr-2014	Feher, Nemeth, Mizsei	Geni-Al		16SLOrc1_fwd/16SLOrc_rev		I
Mmn-206-01	HNHM 94833	Montenegrina minuscula		KU308135	KU307963	Albania	Kurbnesh N 1 km		N41.7883	E20.1068	27-Jun-2003	Eross, Feher, Kontschan, Muranyi	Geni-Al		16SLOrc1_fwd/16SLOrc_rev	12SGastFw/12SGastRv	J
Mmn-252-01	HNHM 96824	Montenegrina minuscula	KU307712	KU308136		Albania	3 km W of Qafa e Murres		N41.6465	E20.1898	11-Oct-2005	Deli, Eross, Feher, Muranyi	Geni-Al	LCO1490/H2198-Alb	16SLOrc1_fwd/16SLOrc_rev		J
Mmn-308-01	HNHM 99625	Montenegrina minuscula	KU307713		KU307964	Albania	Kurbnesh NE 2 km		N41.7952	E20.1117	20-May-2010	Feher, Muranyi, Ujvari	Geni-Al	LCO1490/H2198-Alb		12SGastFw/12SGastRv	J
Mmr-319-01	HNHM 99630	Montenegrina laxa miraka	KU307714	KU308137	KU307965	Albania	Miraka		N41.1634	E20.2301	22-Jun-2012	Feher, Kovacs, Muranyi	Geni-Al	LCO1490/H2198-Alb	16SLOrc1_fwd/16SLOrc_rev	12SGastFw/12SGastRv	L
Mmr-436-01	HNHM 98964	Montenegrina laxa miraka		KU308138		Albania	Lunik S 1 km		N41.2661	E20.3176	13-Apr-2014	Feher, Nemeth, Mizsei	Geni-Al		16SLOrc1_fwd/16SLOrc_rev		L
Mmr-437-01	HNHM 98969	Montenegrina laxa miraka		KU308139		Albania	Lunik E		N41.2776	E20.3375	13-Apr-2014	Feher, Nemeth, Mizsei	Geni-Al		16SLOrc1_fwd/16SLOrc_rev		L
Mmr-464-01	HNHM 99046	Montenegrina laxa miraka	KU307715	KU308140	KU307966	Albania	Mengli		N41.1335	E20.1261	17-Apr-2014	Feher, Nemeth, Mizsei	Geni-Al	LCO1490/H2198-Alb	16SLOrc1_fwd/16SLOrc_rev	12SGastFw/12SGastRv	L
Mmu-226-01	HNHM 94847	Montenegrina fuchsi muranyii	KU307716	KU308141		Albania	Tomorr Mts, Kalaja e Tomorrit	paratype	N40.7025	E20.1093	26-May-2004	Harmos, Muranyi	Geni-Al	LCO1490/H2198-Alb	16SLOrc1_fwd/16SLOrc_rev		K
Mmu-226-02	HNHM 94847	Montenegrina fuchsi muranyii	KU307717			Albania	Tomorr Mts, Kalaja e Tomorrit	paratype	N40.7025	E20.1093	26-May-2004	Harmos, Muranyi	Geni-Al	LCO1490/H2198-Alb			K
Mmu-226-04	HNHM 94847	Montenegrina fuchsi muranyii	KU307718			Albania	Tomorr Mts, Kalaja e Tomorrit	paratype	N40.7025	E20.1093	26-May-2004	Harmos, Muranyi	Geni-Al	LCO1490/H2198-Alb			K
Mmu-226-05	HNHM 94847	Montenegrina fuchsi muranyii	KU307719			Albania	Tomorr Mts, Kalaja e Tomorrit	paratype	N40.7025	E20.1093	26-May-2004	Harmos, Muranyi	Geni-Al	LCO1490/H2198-Alb			K
Mna-439-01	HNHM 98973	Montenegrina nana nana	KU307720	KU308142		Albania	Lunik NE 3 km		N41.2966	E20.3741	13-Apr-2014	Feher, Nemeth, Mizsei	Geni-Al	LCO1490/H2198-Alb	16SLOrc1_fwd/16SLOrc_rev		I
Mno-427-01	HNHM 99536	Montenegrina skipetarica nobilis		KU308143		Albania	Borove S 4 km		N40.2912	E20.6284	15-Oct-2013	Juhasz, Kovacs, Muranyi, Puskas	Geni-Al		16SLOrc1_fwd/16SLOrc_rev		C
Mno-478-01	NHMW 99382	Montenegrina skipetarica nobilis		KU308144	KU307967	Albania	Borove S 4 km		N40.2913	E20.6273	29-Jun-2014	Angyal, Eross, Feher, Grego	Geni-Al		16SLOrc1_fwd/16SLOrc_rev	12SGastFw/12SGastRv	C
Moc-216-02	HNHM 94441	Montenegrina perstriata occidentalis	KU307721	KU308145		Macedonia	Kalista		N41.1493	E20.6493	07-Apr-2004	Eross, Feher, Hunyadi	Geni-Al	LCO1490/H2198-Alb	16SLOrc1_fwd/16SLOrc_rev		H
Moc-216-03	HNHM 94441	Montenegrina perstriata occidentalis	KU307722	KU308146		Macedonia	Kalista		N41.1493	E20.6493	07-Apr-2004	Eross, Feher, Hunyadi	Geni-Al	LCO1490/H2198-Alb	16SLOrc1_fwd/16SLOrc_rev		H
Moc-411-01	HNHM 99615	Montenegrina perstriata occidentalis	KU307723	KU308147	KU307968	Albania	Lin		N41.0693	E20.6468	30-Jun-2013	Eross, Feher, Grego	Geni-Al	LCO1490/H2198-Alb	16SLOrc1_fwd/16SLOrc_rev	12SGastFw/12SGastRv	H
Moh-213-01	HNHM 94434	Montenegrina perstriata ochridensis	KU307724	KU308148	KU307969	Macedonia	Pestani S		N41.0087	E20.8092	06-Apr-2004	Eross, Feher, Hunyadi	Geni-Al	LCO1490/H2198-Alb	16SLOrc1_fwd/16SLOrc_rev	12SGastFw/12SGastRv	I
Moh-214-01	HNHM 94428	Montenegrina perstriata ochridensis	KU307725	KU308149		Macedonia	Ohrid, Izvor Studenicista		N41.103	E20.8143	07-Apr-2004	Eross, Feher, Hunyadi	Geni-Al	LCO1490/H2198-Alb	16SLOrc1_fwd/16SLOrc_rev		I
Moh-452-01	HNHM 99006	Montenegrina perstriata ochridensis	KU307726	KU308150	KU307970	Albania	Zerqan		N41.516	E20.3906	15-Apr-2014	Feher, Nemeth, Mizsei	Geni-Al	LCO1490/H2198-Alb	16SLOrc1_fwd/16SLOrc_rev	12SGastFw/12SGastRv	I
Moh-453-01	HNHM 99007	Montenegrina perstriata ochridensis	KU307727	KU308151		Albania	Mount Temlishit		N41.514	E20.316	15-Apr-2014	Feher, Nemeth, Mizsei	Geni-Al	LCO1490/H2198-Alb	16SLOrc1_fwd/16SLOrc_rev		I
Moh-535-01	NHMW 110430/MN/0079	Montenegrina perstriata ochridensis	KU307728	KU308152		Macedonia	Sipokno		N41.0843	E20.7973	15-Oct-2014	Feher, Haring, Jaksch, Sattmann	Geni-Al	LCO1490/H2198-Alb	16SLOrc1_fwd/16SLOrc_rev		I
Moh-556-01	NHMW 110430/MN/0080	Montenegrina perstriata ochridensis		KU308084		Macedonia	Gradiste		N40.9937	E20.7992	16-Oct-2014	Feher, Haring, Jaksch, Sattmann	Geni-Al		16SLOrc1_fwd/16SLOrc_rev		I
Mok-243-01	HNHM 99613	Montenegrina okolensis okolensis	KU307729	KU308153		Albania	Qafa Valbones		N42.4068	E19.8122	06-Oct-2005	Deli, Eross, Feher, Muranyi	Geni-Al	LCO1490/H2198-Alb	16SLOrc1_fwd/16SLOrc_rev		G
Mok-486-01	HNHM 99614	Montenegrina okolensis okolensis	KU307730	KU308154		Albania	Qafa Valbones		N42.4086	E19.8129	05-Sep-2013	Adam, Puskas, Somay	Geni-Al	LCO1490/H2198-Alb	16SLOrc1_fwd/16SLOrc_rev		G
Mok-486-02	HNHM 99614	Montenegrina okolensis okolensis	KU307731	KU308155		Albania	Qafa Valbones		N42.4086	E19.8129	05-Sep-2013	Adam, Puskas, Somay	Geni-Al	LCO1490/H2198-Alb	16SLOrc1_fwd/16SLOrc_rev		G
Mok-486-03	HNHM 99614	Montenegrina okolensis okolensis	KU307732	KU308156		Albania	Qafa Valbones		N42.4086	E19.8129	05-Sep-2013	Adam, Puskas, Somay	Geni-Al	LCO1490/H2198-Alb	16SLOrc1_fwd/16SLOrc_rev		G
Mor-318-01	HNHM 99617	Montenegrina helvola ornata	KU307733	KU308157	KU307971	Albania	Petresh		N41.103	E20.0065	22-Jun-2012	Feher, Kovacs, Muranyi	Geni-Al	LCO1490/H2198-Alb	16SLOrc1_fwd/16SLOrc_rev	12SGastFw/12SGastRv	I
Mor-498-01	HNHM 94904	Montenegrina helvola ornata	KU307734	KU308158		Albania	Shkame 1 km SE		N40.928	E20.0306	29-Jun-2003	Eross, Feher, Kontschan, Muranyi	Thomsen et al. (2009)	LCO1490/H2198-Alb	16SLOrc1_fwd/16SLOrc_rev		I
Mpa-392-01	HNHM 99566	Montenegrina fuchsi pallida	KU307735	KU308159	KU307972	Greece	Molivdokepastos SW 2 km		N40.0468	E20.5648	26-Jun-2013	Eross, Feher, Grego	Geni-Al	LCO1490/H2198-Alb	16SLOrc1_fwd/16SLOrc_rev	12SGastFw/12SGastRv	D1
Mpa-392-02	HNHM 99566	Montenegrina fuchsi pallida	KU307736			Greece	Molivdokepastos SW 2 km		N40.0468	E20.5648	26-Jun-2013	Eross, Feher, Grego	Geni-Al	LCO1490/H2198-Alb			D1
Mpa-392-03	HNHM 99566	Montenegrina fuchsi pallida	KU307737			Greece	Molivdokepastos SW 2 km		N40.0468	E20.5648	26-Jun-2013	Eross, Feher, Grego	Geni-Al	LCO1490/H2198-Alb			D1
Mpe-223-01	HNHM 94437	Montenegrina perstriata perstriata		KU308160		Macedonia	Galicnik		N41.5937	E20.6577	09-Apr-2004	Eross, Feher, Hunyadi	Geni-Al		16SLOrc1_fwd/16SLOrc_rev		L
Mpe-223-03	HNHM 94437	Montenegrina perstriata perstriata	KU307738			Macedonia	Galicnik		N41.5937	E20.6577	09-Apr-2004	Eross, Feher, Hunyadi	Geni-Al	LCO1490/H2198-Alb			L
Mpe-500-01	HNHM 94436	Montenegrina perstriata perstriata		KU308161		Macedonia	Galicnik E 1 km		N41.5942	E20.6722	09-Apr-2004	Eross, Feher, Hunyadi	Thomsen et al. (2009)		16SLOrc1_fwd/16SLOrc_rev		L
Mpe-511-01	NHMW 110430/MN/0104	Montenegrina perstriata perstriata	KU307739			Macedonia	Galicnik E 1 km		N41.5942	E20.6716	14-Oct-2014	Feher, Haring, Jaksch, Sattmann	Geni-Al	LCO1490/H2198-Alb			L
Mpe-512-01	NHMW 110430/MN/0105	Montenegrina perstriata perstriata	KU307740	KU308162		Macedonia	Galicnik		N41.5936	E20.6575	14-Oct-2014	Feher, Haring, Jaksch, Sattmann	Geni-Al	LCO1490/H2198-Alb	16SLOrc1_fwd/16SLOrc_rev		L
Mpe-513-01	NHMW 110430/MN/0106	Montenegrina perstriata perstriata	KU307741			Macedonia	Galicnik E 11 km		N41.624	E20.6768	14-Oct-2014	Feher, Haring, Jaksch, Sattmann	Geni-Al	LCO1490/H2198-Alb			L
Mpg-471-01	HNHM 99616	Montenegrina helvola pageti	KU307742	KU308163		Albania	Berat		N40.7041	E19.9479	27-Jun-2014	Angyal, Eross, Feher, Grego	Geni-Al	LCO1490/H2198-Alb	16SLOrc1_fwd/16SLOrc_rev		I
Mpi-212-01	HNHM 94438	Montenegrina dofleini pinteri	KU307743	KU308164		Macedonia	Trpejca N		N40.966	E20.7858	06-Apr-2004	Eross, Feher, Hunyadi	Geni-Al	LCO1490/H2198-Alb	16SLOrc1_fwd/16SLOrc_rev		D2
Mpi-212-04	HNHM 94438	Montenegrina dofleini pinteri	KU307744			Macedonia	Trpejca N		N40.966	E20.7858	06-Apr-2004	Eross, Feher, Hunyadi	Geni-Al	LCO1490/H2198-Alb			D2
Mpi-508-01	NHMW 110430/MN/0052	Montenegrina dofleini pinteri	KU307745			Macedonia	Trpejca N		N40.9656	E20.7859	09-Aug-2014	Feher Z, A, T, Tamas L.	Geni-Al	LCO1490/H2198-Alb			D2
Mpi-508-02	NHMW 110430/MN/0052	Montenegrina dofleini pinteri	KU307746			Macedonia	Trpejca N		N40.9656	E20.7859	09-Aug-2014	Feher Z, A, T, Tamas L.	Geni-Al	LCO1490/H2198-Alb			D2
Mpi-508-03	NHMW 110430/MN/0052	Montenegrina dofleini pinteri	KU307747			Macedonia	Trpejca N		N40.9656	E20.7859	09-Aug-2014	Feher Z, A, T, Tamas L.	Geni-Al	LCO1490/H2198-Alb			D2
Mpl-430-01	HNHM 98945	Montenegrina perstriata plenostoma	KU307748	KU308165		Albania	Qafa Thanes E 2 km		N41.0632	E20.6253	12-Apr-2014	Feher, Nemeth, Mizsei	Geni-Al	LCO1490/H2198-Alb	16SLOrc1_fwd/16SLOrc_rev		H
Mpn-396-01	HNHM 99525	Montenegrina skipetarica pindica	KU307749	KU308166	KU307973	Greece	Gramos Mts, NW of Epano Arena		N40.3115	E20.8986	27-Jun-2013	Eross, Feher, Grego	Geni-Al	LCO1490/H2198-Alb	16SLOrc1_fwd/16SLOrc_rev	12SGastFw/12SGastRv	B
Mpn-397-02	HNHM 99526	Montenegrina skipetarica pindica	KU307750			Greece	Gramos Mts, N of Epano Arena		N40.3131	E20.8995	27-Jun-2013	Eross, Feher, Grego	Geni-Al	LCO1490/H2198-Alb			B
Mpo-370-01	HNHM 99593	Montenegrina hiltrudae protruda	KU307751	KU308167		Greece	Aetia E 2 km to Anavrita		N40.0741	E21.2017	22-Jun-2013	Eross, Feher, Grego	Geni-Al	LCO1490/H2198-Alb	16SLOrc1_fwd/16SLOrc_rev		E
Mpo-370-03	HNHM 99593	Montenegrina hiltrudae protruda	KU307752	KU308168		Greece	Aetia E 2 km to Anavrita		N40.0741	E21.2017	22-Jun-2013	Eross, Feher, Grego	Geni-Al	LCO1490/H2198-Alb	16SLOrc1_fwd/16SLOrc_rev		E
Mpr-110-01	HNHM 12040	Montenegrina dofleini prespaensis	KU307753	KU308169	KU307974	Greece	Psarades		N40.8315	E21.0288	10-May-1995	Subai	Geni-Al	LCO1490/H2198-Alb	16SLOrc1_fwd/16SLOrc_rev	12SGastFw/12SGastRv	D2
Mpr-404-01	HNHM 99573	Montenegrina dofleini prespaensis	KU307754		KU307975	Greece	Psarades		N40.8316	E21.0287	28-Jun-2013	Eross, Feher, Grego	Geni-Al	LCO1490/H2198-Alb		12SGastFw/12SGastRv	D2
Mpr-404-02	HNHM 99573	Montenegrina dofleini prespaensis	KU307755			Greece	Psarades		N40.8316	E21.0287	28-Jun-2013	Eross, Feher, Grego	Geni-Al	LCO1490/H2198-Alb			D2
Mpr-404-03	HNHM 99573	Montenegrina dofleini prespaensis	KU307756			Greece	Psarades		N40.8316	E21.0287	28-Jun-2013	Eross, Feher, Grego	Geni-Al	LCO1490/H2198-Alb			D2
Mpr-404-04	HNHM 99573	Montenegrina dofleini prespaensis	KU307757			Greece	Psarades		N40.8316	E21.0287	28-Jun-2013	Eross, Feher, Grego	Geni-Al	LCO1490/H2198-Alb			D2
Mpr-404-05	HNHM 99573	Montenegrina dofleini prespaensis	KU307758			Greece	Psarades		N40.8316	E21.0287	28-Jun-2013	Eross, Feher, Grego	Geni-Al	LCO1490/H2198-Alb			D2
Mpr-545-01	NHMW 110430/MN/0055	Montenegrina dofleini prespaensis	KU307759			Albania	Kallamas		N40.8924	E20.9394	17-Oct-2014	Feher, Haring, Jaksch, Sattmann	Geni-Al	LCO1490/H2198-Alb			D2
Mpr-545-02	NHMW 110430/MN/0055	Montenegrina dofleini prespaensis	KU307760			Albania	Kallamas		N40.8924	E20.9394	17-Oct-2014	Feher, Haring, Jaksch, Sattmann	Geni-Al	LCO1490/H2198-Alb			D2
Mpr-545-03	NHMW 110430/MN/0055	Montenegrina dofleini prespaensis	KU307761			Albania	Kallamas		N40.8924	E20.9394	17-Oct-2014	Feher, Haring, Jaksch, Sattmann	Geni-Al	LCO1490/H2198-Alb			D2
Mpr-549-01	NHMW 110430/MN/0056	Montenegrina dofleini prespaensis	KU307762			Greece	Psarades		N40.8323	E21.0275	18-Oct-2014	Feher, Haring, Jaksch, Sattmann	Geni-Al	LCO1490/H2198-Alb			D2
Mpr-550-01	NHMW 110430/MN/0057	Montenegrina dofleini prespaensis	KU307763	KU308170	KU307976	Greece	Psarades SW, Panayia Eleoussa cave temple		N40.8087	E20.9979	18-Oct-2014	Feher, Haring, Jaksch, Sattmann	Geni-Al	LCO1490/H2198-Alb	16SLOrc1_fwd/16SLOrc_rev	12SGastFw/12SGastRv	D2
Mpr-550-02	NHMW 110430/MN/0057	Montenegrina dofleini prespaensis	KU307764			Greece	Psarades SW, Panayia Eleoussa cave temple		N40.8087	E20.9979	18-Oct-2014	Feher, Haring, Jaksch, Sattmann	Geni-Al	LCO1490/H2198-Alb			D2
Mpr-550-03	NHMW 110430/MN/0057	Montenegrina dofleini prespaensis	KU307765			Greece	Psarades SW, Panayia Eleoussa cave temple		N40.8087	E20.9979	18-Oct-2014	Feher, Haring, Jaksch, Sattmann	Geni-Al	LCO1490/H2198-Alb			D2
Mpr-563-02	HNHM 99572	Montenegrina hiltrudae desaretica	KU307766			Albania	Liqenas, Ishull i Vogel		N40.7915	E20.9324	17-Aug-2007	Feher , Tamas	Thomsen et al. (2009)	LCO1490/H2198-Alb			E
Mpr-616-01	NHMW 110430/MN/0134	Montenegrina dofleini prespaensis	KU307767			Albania	Kallamas E 4 km, Sv. Marena cave temple		N40.8872	E20.9729	29-Jun-2015	Eross, Feher, Grego	Geni-Al	LCO1490/H2198-Alb			D2
Mpr-617-01	NHMW 110430/MN/0135	Montenegrina dofleini prespaensis	KU307768			Albania	Kallamas SE 2 km		N40.8809	E20.959	29-Jun-2015	Eross, Feher, Grego	Geni-Al	LCO1490/H2198-Alb			D2
Mra-503-01	HNHM 94917	Montenegrina perstriata radikae	KU307769	KU308171		Macedonia	Sence junction N 1 km, between Debar and Gostivar		N41.6978	E20.6462	08-Apr-2004	Eross, Feher, Hunyadi	Thomsen et al. (2009)	LCO1490/H2198-Alb	16SLOrc1_fwd/16SLOrc_rev		K
Mra-518-01	NHMW 110430/MN/0086	Montenegrina perstriata radikae	KU307770	KU308172	KU307977	Macedonia	Sence junction N 1 km, between Debar and Gostivar		N41.6994	E20.6478	14-Oct-2014	Feher, Haring, Jaksch, Sattmann	Geni-Al	LCO1490/H2198-Alb	16SLOrc1_fwd/16SLOrc_rev	12SGastFw/12SGastRv	K
Mra-519-01	NHMW 110430/MN/0087	Montenegrina perstriata drimica	KU307771	KU308173		Macedonia	Debar S		N41.4948	E20.5052	14-Oct-2014	Feher, Haring, Jaksch, Sattmann	Geni-Al	LCO1490/H2198-Alb	16SLOrc1_fwd/16SLOrc_rev		H
Mre-238-01	HNHM 94861	Montenegrina skipetarica remota	KU307772	KU308174		Albania	Mat gorge, 11 km W of Ulez junction	paratype	N41.6921	E19.8326	08-Oct-2004	Feher, Kontschan, Muranyi	Geni-Al	LCO1490/H2198-Alb	16SLOrc1_fwd/16SLOrc_rev		B
Mrl-351-01	HNHM 99546	Montenegrina rugilabris rugilabris	KU307773			Greece	Ligiades		N39.7019	E20.8947	04-May-2011	Kontschan, Muranyi, Szederjesi, Ujvari	Geni-Al	LCO1490/H2198-Alb			D1
Mrl-378-01	HNHM 99548	Montenegrina rugilabris rugilabris	KU307774	KU308175		Greece	Ligkiades junction W 1.5 km, between Amfithea and Spothi		N39.6895	E20.8714	24-Jun-2013	Eross, Feher, Grego	Geni-Al	LCO1490/H2198-Alb	16SLOrc1_fwd/16SLOrc_rev		D1
Mrl-379-01	HNHM 99549	Montenegrina rugilabris rugilabris	KU307775			Greece	Ligkiades junction W 1 km, between Amfithea and Spothi		N39.687	E20.874	24-Jun-2013	Eross, Feher, Grego	Geni-Al	LCO1490/H2198-Alb			D1
Mrl-380-01	HNHM 99550	Montenegrina rugilabris rugilabris	KU307776			Greece	Ligkiades junction W 0.5 km, between Amfithea and Spothi		N39.6849	E20.8795	24-Jun-2013	Eross, Feher, Grego	Geni-Al	LCO1490/H2198-Alb			D1

Mrl-381-01	HNHM 99547	Montenegrina rugilabris rugilabris	KU307777	KU308176		Greece	Ligkiades junction E 0.5 km, between Amfithea and Spothi		N39.6828	E20.8887	24-Jun-2013	Eross, Feher, Grego	Geni-Al	LCO1490/H2198-Alb	16SarF/16SLOrc_rev		D1
Mro-554-01	NHMW 110430/MN/0118	Montenegrina hiltrudae robusta	KU307778	KU308177	KU307978	Greece	Gavros 4 km to Kotas		N40.6532	E21.1789	18-Oct-2014	Feher, Haring, Jaksch, Sattmann	Geni-Al	LCO1490/H2198-Alb	16SarF/16SLOrc_rev	12SGastFw/12SGastRv	D2
Mru-229-01	HNHM 94864	Montenegrina skipetarica rugosa	KU307779	KU308178		Albania	Qafa e Devris, between Corovode and Zaloshnje		N40.5536	E20.2783	08-Aug-2004	Feher	Geni-Al	LCO1490/H2198-Alb	16SLOrc1_fwd/16SLOrc_rev		C
Mru-264-01	HNHM 99540	Montenegrina skipetarica rugosa	KU307780	KU308179	KU307979	Albania	Corovode NE 5 km		N40.5223	E20.2574	22-Aug-2006	Feher, Hunyadi, Huszar, Muranyi	Geni-Al	LCO1490/H2198-Alb	16SLOrc1_fwd/16SLOrc_rev	12SGastFw/12SGastRv	C
Mru-264-02	HNHM 99540	Montenegrina skipetarica rugosa	KU307781			Albania	Corovode NE 5 km		N40.5223	E20.2574	22-Aug-2006	Feher, Hunyadi, Huszar, Muranyi	Geni-Al	LCO1490/H2198-Alb			C
Mru-264-05	HNHM 99540	Montenegrina skipetarica rugosa	KU307782			Albania	Corovode NE 5 km		N40.5223	E20.2574	22-Aug-2006	Feher, Hunyadi, Huszar, Muranyi	Geni-Al	LCO1490/H2198-Alb			C
Mru-265-01	HNHM 99538	Montenegrina skipetarica rugosa	KU307783	KU308180	KU307980	Albania	Qafa e Devris, between Corovode and Zaloshnje		N40.5561	E20.2757	22-Aug-2006	Feher, Hunyadi, Huszar, Muranyi	Geni-Al	LCO1490/H2198-Alb	16SLOrc1_fwd/16SLOrc_rev	12SGastFw/12SGastRv	C
Msa-274-02	HNHM 99588	Montenegrina hiltrudae sattmanni	KU307784			Albania	Zvezde N 1.7 km		N40.7419	E20.8635	25-May-2007	Barina, Pifko, Nemeth	Thomsen et al. (2009)	LCO1490/H2198-Alb			E
Msa-274-03	HNHM 99588	Montenegrina hiltrudae sattmanni	KU307785			Albania	Zvezde N 1.7 km		N40.7419	E20.8635	25-May-2007	Barina, Pifko, Nemeth	Thomsen et al. (2009)	LCO1490/H2198-Alb			E
Msa-402-01	HNHM 99585	Montenegrina hiltrudae sattmanni	KU307786	KU308181		Greece	Mikrolimni		N40.7429	E21.1102	28-Jun-2013	Eross, Feher, Grego	Geni-Al	LCO1490/H2198-Alb	16SLOrc1_fwd/16SLOrc_rev		E
Msa-408-01	HNHM 99587	Montenegrina hiltrudae desaretica	KU307787	KU308182	KU307981	Albania	Liqenas NE 1 km		N40.7919	E20.9152	29-Jun-2013	Eross, Feher, Grego	Geni-Al	LCO1490/H2198-Alb	16SLOrc1_fwd/16SLOrc_rev	12SGastFw/12SGastRv	E
Msa-546-01	NHMW 110430/MN/0064	Montenegrina hiltrudae sattmanni	KU307788			Albania	Glloboceni N		N40.858	E20.9426	17-Oct-2014	Feher, Haring, Jaksch, Sattmann	Geni-Al	LCO1490/H2198-Alb			E
Msa-551-01	NHMW 110430/MN/0065	Montenegrina hiltrudae sattmanni	KU307789			Greece	Ag. Achillios junction between Psarades and Vrondero		N40.7998	E21.0715	18-Oct-2014	Feher, Haring, Jaksch, Sattmann	Geni-Al	LCO1490/H2198-Alb			E
Msa-552-01	NHMW 110430/MN/0066	Montenegrina hiltrudae sattmanni	KU307790	KU308183		Greece	Vrondero SE 3 km, Petros Kokkalis cave		N40.7192	E21.0341	18-Oct-2014	Feher, Haring, Jaksch, Sattmann	Geni-Al	LCO1490/H2198-Alb	16SarF/16SLOrc_rev		E
Msi-403-01	HNHM 99580	Montenegrina dofleini sinosi	KU307791		KU307982	Greece	Agios Achillios, near Psarades junction		N40.8101	E21.0703	28-Jun-2013	Eross, Feher, Grego	Geni-Al	LCO1490/H2198-Alb		12SGastFw/12SGastRv	D2
Msi-403-02	HNHM 99580	Montenegrina dofleini sinosi	KU307792			Greece	Agios Achillios, near Psarades junction		N40.8101	E21.0703	28-Jun-2013	Eross, Feher, Grego	Geni-Al	LCO1490/H2198-Alb			D2
Msi-403-03	HNHM 99580	Montenegrina dofleini sinosi	KU307793			Greece	Agios Achillios, near Psarades junction		N40.8101	E21.0703	28-Jun-2013	Eross, Feher, Grego	Geni-Al	LCO1490/H2198-Alb			D2
Msi-553-01	NHMW 110430/MN/0058	Montenegrina dofleini sinosi	KU307794	KU308184		Greece	Agios Achillios, near Psarades junction		N40.8105	E21.0702	18-Oct-2014	Feher, Haring, Jaksch, Sattmann	Geni-Al	LCO1490/H2198-Alb	16SLOrc1_fwd/16SLOrc_rev		D2
Msi-553-02	NHMW 110430/MN/0058	Montenegrina dofleini sinosi	KU307795			Greece	Agios Achillios, near Psarades junction		N40.8105	E21.0702	18-Oct-2014	Feher, Haring, Jaksch, Sattmann	Geni-Al	LCO1490/H2198-Alb			D2
Msi-553-03	NHMW 110430/MN/0058	Montenegrina dofleini sinosi	KU307796			Greece	Agios Achillios, near Psarades junction		N40.8105	E21.0702	18-Oct-2014	Feher, Haring, Jaksch, Sattmann	Geni-Al	LCO1490/H2198-Alb			D2
Msk-202-01	HNHM 93813	Montenegrina skipetarica skipetarica	KU307797	KU308185		Albania	Bushtrice N 2.5 km, Ura e Lapaves		N41.8949	E20.4169	25-Jun-2003	Eross, Feher, Kontschan, Muranyi	Geni-Al	LCO1490/H2198-Alb	16SLOrc1_fwd/16SLOrc_rev		B
Msk-301-01	HNHM 99522	Montenegrina skipetarica skipetarica	KU307798	KU308186	KU307983	Albania	3 km W of Qafa e Murres		N41.6465	E20.1901	18-May-2010	Barina, Feher, Muranyi, Pifko, Ujvari	Geni-Al	LCO1490/H2198-Alb	16SLOrc1_fwd/16SLOrc_rev	12SGastFw/12SGastRv	B
Msk-302-01	HNHM 99523	Montenegrina skipetarica skipetarica		KU308187		Albania	5 km towards Fushë-Lurë from the Peshkopi-Burrel road		N41.6651	E20.2083	18-May-2010	Feher, Muranyi, Ujvari	Geni-Al		16SLOrc1_fwd/16SLOrc_rev		B
Msk-303-01	HNHM 99691	Montenegrina skipetarica puskasi	KU307799	KU308188		Albania	above the Varosh Spring		N41.6747	E20.2112	18-May-2010	Feher, Muranyi, Ujvari	Geni-Al	LCO1490/H2198-Alb	16SLOrc1_fwd/16SLOrc_rev		B
Msk-306-01	HNHM	Montenegrina skipetarica ssp.		KU308189		Albania	Mount Laje, Cidhne S 7 km		N41.6919	E20.2129	19-May-2010	Barina, Pifkó	Geni-Al		16SLOrc1_fwd/16SLOrc_rev		B
Msl-222-02	HNHM 94919	Montenegrina perstriata subcristatula		KU308190		Macedonia	Nicpur junction, between Debar and Gostivar		N41.7205	E20.6683	08-Apr-2004	Eross, Feher, Hunyadi	Geni-Al		16SLOrc1_fwd/16SLOrc_rev		L
Msl-222-04	HNHM 94919	Montenegrina perstriata subcristatula		KU308191		Macedonia	Nicpur junction, between Debar and Gostivar		N41.7205	E20.6683	08-Apr-2004	Eross, Feher, Hunyadi	Geni-Al		16SLOrc1_fwd/16SLOrc_rev		L
Msl-514-01	NHMW 110430/MN/0107	Montenegrina perstriata subcristatula	KU307800	KU308192	KU307984	Macedonia	Nicpur junction, between Debar and Gostivar		N41.7203	E20.6682	14-Oct-2014	Feher, Haring, Jaksch, Sattmann	Geni-Al	LCO1490/H2198-Alb	16SLOrc1_fwd/16SLOrc_rev	12SGastFw/12SGastRv	L
Msl-515-01	NHMW 110430/MN/0108	Montenegrina perstriata subcristatula	KU307801	KU308193		Macedonia	6 km to Nicpur from the Debar to Gostivar road		N41.7655	E20.6666	14-Oct-2014	Feher, Haring, Jaksch, Sattmann	Geni-Al	LCO1490/H2198-Alb	16SLOrc1_fwd/16SLOrc_rev		L
Msl-516-01	NHMW 110430/MN/0109	Montenegrina perstriata subcristatula	KU307802	KU308194	KU307985	Macedonia	Nistrovo junction E 0.5 km, between Debar and Gostivar		N41.7102	E20.6564	14-Oct-2014	Feher, Haring, Jaksch, Sattmann	Geni-Al	LCO1490/H2198-Alb	16SLOrc1_fwd/16SLOrc_rev	12SGastFw/12SGastRv	L
Msl-517-01	NHMW 110430/MN/0110	Montenegrina perstriata subcristatula	KU307803	KU308195		Macedonia	Nistrovo junction, between Debar and Gostivar		N41.7079	E20.65	14-Oct-2014	Feher, Haring, Jaksch, Sattmann	Geni-Al	LCO1490/H2198-Alb	16SLOrc1_fwd/16SLOrc_rev		L
Mso-246-01	HNHM 99637	Montenegrina soosi	KU307804			Albania	Bushtrice River mouth		N41.9352	E20.3632	09-Oct-2005	Deli, Eross, Feher, Muranyi	Geni-Al	LCO1490/H2198-Alb			L
Mso-412-01	NHMW 110430/MN/0112	Montenegrina soosi	KU307805			Albania	Kolesjan S 1.8 km		N41.9613	E20.3955	01-Sep-2013	Reischutz	Geni-Al	LCO1490/H2198-Alb			L
Mso-413-01	NHMW 110430/MN/0113	Montenegrina soosi	KU307806	KU308196	KU307986	Albania	Kolesjan S 7.4 km		N41.926	E20.3872	01-Sep-2013	Reischutz	Geni-Al	LCO1490/H2198-Alb	16SLOrc1_fwd/16SLOrc_rev	12SGastFw/12SGastRv	L
Msr-107-01	HNHM 36936	Montenegrina subcristata	KU307807			Montenegro	Zacir, Pecina u peckom brdu Cave		N42.3367	E18.9971	20-Jul-1972	Pinter, Subai, Szigethy	Geni-Al	LCO1490/H2198-Alb			F1
Msr-288-01	HNHM 99648	Montenegrina subcristata	KU307808	KU308197		Montenegro	Virpazar S 9 km		N42.1912	E19.1082	14-Oct-2008	Danyi, Feher, Kontschan, Muranyi	Geni-Al	LCO1490/H2198-Alb	16SLOrc1_fwd/16SLOrc_rev		F1
Msr-294-01	HNHM 99537	Montenegrina subcristata	KU307809	KU308198		Montenegro	Virpazar S 12 km to Petrovac		N42.2188	E19.0246	14-Oct-2008	Danyi, Feher, Kontschan, Muranyi	Geni-Al	LCO1490/H2198-Alb	16SLOrc1_fwd/16SLOrc_rev		F1
Msr-310-01	HNHM 99489	Montenegrina subcristata	KU307810			Montenegro	Sas		N41.9768	E19.3387	16-Jun-2012	Feher, Kovacs, Muranyi	Geni-Al	LCO1490/H2198-Alb			F1
Msr-355-01	HNHM 99478	Montenegrina subcristata	KU307811			Albania	Vidhgar		N42.0572	E19.3963	14-May-2013	Barina, Pifko	Geni-Al	LCO1490/H2198-Alb			F1
Msr-364-01	HNHM 99535	Montenegrina subcristata	KU307812			Montenegro	Klezna N		N42.0023	E19.2633	26-May-2013	Juhasz, Kovacs, Magos, Puskas	Geni-Al	LCO1490/H2198-Alb			F1
Msr-423-01	HNMW 110430/MN/0119	Montenegrina subcristata	KU307813	KU308199		Montenegro	Vitoja Spring		N42.3252	E19.3623	01-Oct-2013	Reischutz	Geni-Al	LCO1490/H2198-Alb	16SLOrc1_fwd/16SLOrc_rev		F1
Msr-467-01	HNHM 99647	Montenegrina subcristata	KU307814	KU308200		Montenegro	Sutorman Pass		N42.1558	E19.1025	01-Jun-2014	Nemeth	Geni-Al	LCO1490/H2198-Alb	16SLOrc1_fwd/16SLOrc_rev		F1
Msr-489-01	HNHM 91032	Montenegrina subcristata	KU307815			Montenegro	Bozaj W 2 km		N42.309	E19.37	30-Jun-1996	Eross, Feher	Thomsen et al. (2009)	LCO1490/H2198-Alb			F1
Msr-490-01	HNHM 90880	Montenegrina subcristata		KU308201		Montenegro	Buljarica S 2 km		N42.1911	E18.9918	29-Jun-1996	Eross, Feher	Thomsen et al. (2009)		16SLOrc1_fwd/16SLOrc_rev		F1
Msr-506-01	HNHM 95418	Montenegrina subcristata	KU307816	KU308202		Montenegro	Komarno, Jabukov Do pecina		N42.2765	E19.0709	19-Apr-2000	Eross, Feher	Thomsen et al. (2009)	LCO1490/H2198-Alb	16SLOrc1_fwd/16SLOrc_rev		F1
Msr-561-03	NHMW 21644	Montenegrina subcristata	KU307817			Montenegro	Rijeka Brucke near Rijeka Crnojevica		N42.356	E19.024	01-Aug-1934	Bischoff (ex coll. Klemm)	Thomsen et al. (2009)	LCO1490/H2198-Alb			F1
Msr-569-01	HNHM 94951	Montenegrina subcristata	KU307818			Montenegro	Njegus Plateau		N42.4267	E18.8314	12-Oct-2003	Barina, Pifko	Thomsen et al. (2009)	LCO1490/H2198-Alb			F1
Msr-571-01	HNHM 37764	Montenegrina subcristata	KU307819			Montenegro	Njegusi N, Popova Pecina		N42.434	E18.8189	24-Jul-1972	Pinter, Subai, Szigethy	Thomsen et al. (2009)	LCO1490/H2198-Alb			F1
Msr-572-01	HNHM 43121/13	Montenegrina subcristata	KU307820			Montenegro	Dobra Voda N, Tunel Ujtin		N42.0476	E19.136	08-Jul-1985	Kiss, Pinter	Thomsen et al. (2009)	LCO1490/H2198-Alb			F1
Msr-572-02	HNHM 43121/13	Montenegrina subcristata	KU307821			Montenegro	Dobra Voda N, Tunel Ujtin		N42.0476	E19.136	08-Jul-1985	Kiss, Pinter	Thomsen et al. (2009)	LCO1490/H2198-Alb			F1
Msr-574-01	HNHM 43124/4	Montenegrina subcristata	KU307822			Montenegro	Ulici junction on the Podgorica to Cetinje road		N42.379	E18.9978	09-Jul-1985	Kiss, Pinter	Thomsen et al. (2009)	LCO1490/H2198-Alb			F1
Msr-577-01	HNHM 94911	Montenegrina subcristata	KU307823			Montenegro	Zagorak W		N42.6306	E19.0085	07-Oct-2003	Barina, Pifko	Thomsen et al. (2009)	LCO1490/H2198-Alb			F1
Msr-577-02	HNHM 94911	Montenegrina subcristata	KU307824			Montenegro	Zagorak W		N42.6306	E19.0085	07-Oct-2003	Barina, Pifko	Thomsen et al. (2009)	LCO1490/H2198-Alb			F1
Msr-587-01	NHMW 110430/MN/0128	Montenegrina subcristata	KU307825			Montenegro	Poseljani		N42.3058	E19.0481	25-May-2015	Deli, Eross, Feher	Geni-Al	LCO1490/H2198-Alb			F1
Msr-588-01	NHMW 110430/MN/0129	Montenegrina subcristata	KU307826			Montenegro	Obodska Pecina, W of Rijeka Crnojevica		N42.3523	E19.0047	25-May-2015	Deli, Eross, Feher	Geni-Al	LCO1490/H2198-Alb			F1
Msr-589-01	NHMW 110430/MN/0130	Montenegrina subcristata	KU307827			Montenegro	Rijeka Crnojevica NW 6.5 km to Cetinje		N42.3651	E18.9924	26-May-2015	Deli, Eross, Feher	Geni-Al	LCO1490/H2198-Alb			F1
Msr-590-01	NHMW 110430/MN/0131	Montenegrina subcristata	KU307828			Montenegro	Ulici junction E 1 km on the Podgorica to Cetinje road		N42.3815	E19.0084	26-May-2015	Deli, Eross, Feher	Geni-Al	LCO1490/H2198-Alb			F1
Msr-591-01	NHMW 110430/MN/0132	Montenegrina subcristata	KU307829			Montenegro	Dobrska Zupa junction on the Podgorica to Cetinje road		N42.3865	E19.0571	26-May-2015	Deli, Eross, Feher	Geni-Al	LCO1490/H2198-Alb			F1
Msr-592-01	NHMW 110430/MN/0133	Montenegrina subcristata	KU307830			Montenegro	Cetinje		N42.4006	E18.9152	26-May-2015	Deli, Eross, Feher	Geni-Al	LCO1490/H2198-Alb			F1
Msr-597-01	NHMW 110430/MN/0138	Montenegrina subcristata	KU307831			Montenegro	Zaljevo S 1 km		N42.0572	E19.1278	26-May-2015	Deli, Eross, Feher	Geni-Al	LCO1490/H2198-Alb			F1
Msr-597-02	NHMW 110430/MN/0138	Montenegrina subcristata	KU307832			Montenegro	Zaljevo S 1 km		N42.0572	E19.1278	26-May-2015	Deli, Eross, Feher	Geni-Al	LCO1490/H2198-Alb			F1
Msr-597-03	NHMW 110430/MN/0138	Montenegrina subcristata	KU307833			Montenegro	Zaljevo S 1 km		N42.0572	E19.1278	26-May-2015	Deli, Eross, Feher	Geni-Al	LCO1490/H2198-Alb			F1
Msr-604-01	NHMW 110430/MN/0144	Montenegrina subcristata	KU307834			Montenegro	Arbnez S 4 km		N42.0582	E19.3535	28-May-2015	Deli, Eross, Feher	Geni-Al	LCO1490/H2198-Alb			F1
Msr-605-01	NHMW 110430/MN/0145	Montenegrina subcristata	KU307835			Montenegro	Buljarica S 2 km		N42.1906	E18.9898	28-May-2015	Deli, Eross, Feher	Geni-Al	LCO1490/H2198-Alb			F1
Msr-609-01	NHMW 110430/MN/0149	Montenegrina subcristata	KU307836			Montenegro	Njegusi		N42.4316	E18.8126	29-May-2015	Deli, Eross, Feher	Geni-Al	LCO1490/H2198-Alb			F1
Msr-610-01	NHMW 110430/MN/0150	Montenegrina subcristata	KU307837			Montenegro	Niksic S 2 km		N42.7319	E18.9382	30-May-2015	Deli, Eross, Feher	Geni-Al	LCO1490/H2198-Alb			F1
Msr-638-01	NHMW 110430/MN/0156	Montenegrina subcristata	KU307838			Montenegro	Tudjemili N		N42.141	E19.1356	07-Jul-2015	Duda, Haring, Jaksch, Sattmann	Geni-Al	LCO1490/H2198-Alb			F1
Msr-639-01	NHMW 110430/MN/0157	Montenegrina subcristata	KU307839			Montenegro	Sutorman Pass		N42.1568	E19.0996	07-Jul-2015	Duda, Haring, Jaksch, Sattmann	Geni-Al	LCO1490/H2198-Alb			F1
Msr-640-01	NHMW 110430/MN/0158	Montenegrina subcristata	KU307840			Montenegro	Vitoja Spring		N42.3256	E19.3629	08-Jul-2015	Duda, Haring, Jaksch, Sattmann	Geni-Al	LCO1490/H2198-Alb			F1
Msr-642-01	NHMW 110430/MN/0160	Montenegrina subcristata	KU307841			Montenegro	Podgorica Mount Ljubovic		N42.4306	E19.2544	08-Jul-2015	Duda, Haring, Jaksch, Sattmann	Geni-Al	LCO1490/H2198-Alb			F1
Mst-212-01	HNHM 94444	Montenegrina stankovici	KU307842			Macedonia	Trpejca N		N40.9656	E20.7859	06-Apr-2004	Eross, Feher, Hunyadi	Geni-Al	LCO1490/H2198-Alb			D2
Mst-349-01	HNHM 99581	Montenegrina stankovici	KU307843	KU308203	KU307987	Macedonia	Trpejca N		N40.9656	E20.7859	21-Aug-2011	Feher	Geni-Al	LCO1490/H2198-Alb	16SLOrc1_fwd/16SLOrc_rev	12SGastFw/12SGastRv	D2
Mst-494-01	HNHM 90879	Montenegrina stankovici		KU308204		Macedonia	Sv. Naum		N40.914	E20.7406	10-Sep-1994	Feher	Thomsen et al. (2009)		16SLOrc1_fwd/16SLOrc_rev		D2
Mst-496-01	HNHM 94328	Montenegrina stankovici		KU308205		Macedonia	Sv. Naum		N40.914	E20.7406	06-Apr-2004	Eross, Feher, Hunyadi	Thomsen et al. (2009)		16SLOrc1_fwd/16SLOrc_rev		D2
Mst-508-01	NHMW 110430/MN/0059	Montenegrina stankovici	KU307844	KU308206		Macedonia	Trpejca N		N40.9656	E20.7859	09-Aug-2014	Feher Z, A, T, Tamas L.	Geni-Al	LCO1490/H2198-Alb	16SLOrc1_fwd/16SLOrc_rev		D2
Mst-509-01	NHMW 110430/MN/0060	Montenegrina stankovici	KU307845	KU308207		Macedonia	Sv. Naum		N40.914	E20.7406	09-Aug-2014	Feher Z, A, T, Tamas L.	Geni-Al	LCO1490/H2198-Alb	16SLOrc1_fwd/16SLOrc_rev		D2
Mst-510-01	NHMW 110430/MN/0061	Montenegrina stankovici	KU307846	KU308208		Macedonia	Ohrid		N41.1147	E20.7876	10-Aug-2014	Feher Z, A, T, Tamas L.	Geni-Al	LCO1490/H2198-Alb	16SLOrc1_fwd/16SLOrc_rev		D2
Mst-510-11	NHMW 110430/MN/0061	Montenegrina stankovici	KU307847	KU308209		Macedonia	Ohrid		N41.1147	E20.7876	10-Aug-2014	Feher Z, A, T, Tamas L.	Geni-Al	LCO1490/H2198-Alb	16SLOrc1_fwd/16SLOrc_rev		D2
Mst-542-01	NHMW 110430/MN/0062	Montenegrina stankovici	KU307848			Macedonia	Trpejca S		N40.9574	E20.7778	16-Oct-2014	Feher, Haring, Jaksch, Sattmann	Geni-Al	LCO1490/H2198-Alb			D2
Mte-440-01	HNHM 98975	Montenegrina perstriata tenebrosa	KU307849	KU308210		Albania	Maja a Zyllit		N41.3611	E20.3967	13-Apr-2014	Feher	Geni-Al	LCO1490/H2198-Alb	16SLOrc1_fwd/16SLOrc_rev		I
Mti-239-01	HNHM 94857	Montenegrina timeae	KU307850			Albania	Gurri i Bardhe NE 6.7 km		N41.4683	E20.0903	09-Oct-2004	Feher, Kontschan, Muranyi	Geni-Al	LCO1490/H2198-Alb			H
Mti-454-01	HNHM 99009	Montenegrina timeae	KU307851	KU308211	KU307988	Albania	Fshat S 2 km		N41.4718	E20.0967	15-Apr-2014	Feher, Nemeth, Mizsei	Geni-Al	LCO1490/H2198-Alb	16SLOrc1_fwd/16SLOrc_rev	12SGastFw/12SGastRv	H
Mti-458-01	HNHM 99020	Montenegrina timeae	KU307852	KU308212		Albania	Gurri i Bardhe N 5.5 km		N41.471	E20.0779	15-Apr-2014	Feher, Nemeth, Mizsei	Geni-Al	LCO1490/H2198-Alb	16SLOrc1_fwd/16SLOrc_rev		H
Mto-232-01	HNHM 94922	Montenegrina tomorosi tomorosi	KU307853	KU308213		Albania	Tomorr Mts, N of Maja e Kulmakut		N40.5896	E20.2414	09-Aug-2004	Feher	Geni-Al	LCO1490/H2198-Alb	16SLOrc1_fwd/16SLOrc_rev		K
Mto-233-01	HNHM 94921	Montenegrina tomorosi tomorosi	KU307854			Albania	Tomorr Mts, 2.5 km NW of Maja e Kulmakut		N40.5952	E20.2214	09-Aug-2004	Feher	Geni-Al	LCO1490/H2198-Alb			K
Mto-233-02	HNHM 94921	Montenegrina tomorosi tomorosi	KU307855			Albania	Tomorr Mts, 2.5 km NW of Maja e Kulmakut		N40.5952	E20.2214	09-Aug-2004	Feher	Geni-Al	LCO1490/H2198-Alb			K
Mto-233-03	HNHM 94921	Montenegrina tomorosi tomorosi	KU307856			Albania	Tomorr Mts, 2.5 km NW of Maja e Kulmakut		N40.5952	E20.2214	09-Aug-2004	Feher	Geni-Al	LCO1490/H2198-Alb			K
Mx8-266-01	HNHM 99539	Montenegrina skipetarica rugosa	KU307912	KU308240		Albania	Turbehove NE 4.5 km		N40.559	E20.3908	23-Aug-2006	Feher, Hunyadi, Huszar, Muranyi	Geni-Al	LCO1490/H2198-Alb	16SarF/16SLOrc_rev		C
Mum-289-01	HNHM 99604	Montenegrina cattaroensis umbilicata	KU307858	KU308215	KU307990	Montenegro	Stari Bar		N42.0939	E19.1342	14-Oct-2008	Danyi, Feher, Kontschan, Muranyi	Geni-Al	LCO1490/H2198-Alb	16SLOrc1_fwd/16SLOrc_rev	12SGastFw/12SGastRv	F2
Mum-593-01	NHMW 110430/MN/0134	Montenegrina cattaroensis umbilicata	KU307859			Montenegro	Rijeka Rezevici		N42.2271	E18.9117	26-May-2015	Deli, Eross, Feher	Geni-Al	LCO1490/H2198-Alb			F2
Mum-594-01	NHMW 110430/MN/0135	Montenegrina cattaroensis umbilicata	KU307860			Montenegro	Petrovac 3.5 km to Virpazar		N42.2178	E18.9545	26-May-2015	Deli, Eross, Feher	Geni-Al	LCO1490/H2198-Alb			F2
Mum-595-01	NHMW 110430/MN/0136	Montenegrina cattaroensis umbilicata	KU307861			Montenegro	Canj		N42.1649	E19.0069	26-May-2015	Deli, Eross, Feher	Geni-Al	LCO1490/H2198-Alb			F2
Mum-596-01	NHMW 110430/MN/0137	Montenegrina cattaroensis umbilicata	KU307862			Montenegro	Haj Nehaj		N42.153	E19.0237	26-May-2015	Deli, Eross, Feher	Geni-Al	LCO1490/H2198-Alb			F2
Mty-388-01	HNHM 99532	Montenegrina skipetarica thysi	KU307857	KU308214	KU307989	Greece	Vikos gorge, Agia Paraskevi Monastery		N39.891	E20.7538	25-Jun-2013	Eross, Feher, Grego	Geni-Al	LCO1490/H2198-Alb	16SLOrc1_fwd/16SLOrc_rev	12SGastFw/12SGastRv	C
Mvo-389-01	HNHM 99529	Montenegrina skipetarica voidomatis	KU307863	KU308216	KU307991	Greece	Vikos Gorge, Oxia		N39.9067	E20.7519	25-Jun-2013	Eross, Feher, Grego	Geni-Al	LCO1490/H2198-Alb	16SLOrc1_fwd/16SLOrc_rev	12SGastFw/12SGastRv	C
Mvo-390-01	HNHM 99530	Montenegrina skipetarica voidomatis	KU307864	KU308217		Greece	Kapesovo S 6 km, Kokkori's bridge		N39.862	E20.7749	26-Jun-2013	Eross, Feher, Grego	Geni-Al	LCO1490/H2198-Alb	16SarF/16SLOrc_rev		C
Mwa-271-01	HNHM 99576	Montenegrina dofleini wagneri	KU307865	KU308218		Albania	Thate Mts, Podgorije E 4.3 km		N40.8255	E20.8528	20-May-2007	Barina, Pifko, Nemeth	Geni-Al	LCO1490/H2198-Alb	16SLOrc1_fwd/16SLOrc_rev		E

Mwa-564-01	HNHM 99577	Montenegrina dofleini wagneri	KU307866	KU308219		Albania	Thate Mts, Liqenas NW 4.3 km		N40.8183	E20.8728	22-May-2007	Barina, Pifko	Thomsen et al. (2009)	LCO1490/H2198-Alb	16SarF/16SLOrc_rev		E
Mwa-564-02	HNHM 99577	Montenegrina dofleini wagneri	KU307867	KU308220		Albania	Thate Mts, Liqenas NW 4.3 km		N40.8183	E20.8728	22-May-2007	Barina, Pifko	Thomsen et al. (2009)	LCO1490/H2198-Alb	16SarF/16SLOrc_rev		E
Mwe-285-01	HNHM 99556	Montenegrina rugilabris welterschultesi	KU307868			Albania	Petran		N40.2094	E20.4126	18-Aug-2007	Feher, Tamas	Geni-Al	LCO1490/H2198-Alb			D1
Mwe-350-01	HNHM 99557	Montenegrina rugilabris welterschultesi	KU307869	KU308221	KU307993	Albania	Petran		N40.2094	E20.4126	13-Oct-2012	Juhasz, Kovacs, Muranyi, Puskas	Geni-Al	LCO1490/H2198-Alb	16SLOrc1_fwd/16SLOrc_rev	12SGastFw/12SGastRv	D1
Mwo-106-01	HNHM 36723	Montenegrina subcristata	KU307870			Montenegro	Barutan 3 km to Rijeka Crnojevica		N42.3828	E19.1022	20-Jul-1972	Pinter, Subai, Szigethy	Geni-Al	LCO1490/H2198-Alb			F1
Mwo-237-01	HNHM 94509	Montenegrina subcristata	KU307871	KU308222		Albania	Shkoder, Rozafa Hill		N42.0473	E19.4915	14-Aug-2004	Feher	Geni-Al	LCO1490/H2198-Alb	16SLOrc1_fwd/16SLOrc_rev		F1
Mwo-237-03	HNHM 94509	Montenegrina subcristata		KU308223		Albania	Shkoder, Rozafa Hill		N42.0473	E19.4915	14-Aug-2004	Feher	Geni-Al		16SLOrc1_fwd/16SLOrc_rev		F1
Mwo-354-01	HNHM 99649	Montenegrina subcristata	KU307872	KU308224		Albania	Shkoder, Rozafa Hill		N42.0457	E19.4904	14-May-2013	Barina, Pifko	Geni-Al	LCO1490/H2198-Alb	16SLOrc1_fwd/16SLOrc_rev		F1
Mwo-487-01	HNHM 91612	Montenegrina subcristata	KU307873	KU308225		Albania	Torovice		N41.9001	E19.513	21-Oct-2002	Eross, Feher, Kontschan, Muranyi	Thomsen et al. (2009)	LCO1490/H2198-Alb	16SLOrc1_fwd/16SLOrc_rev		F1
Mwo-573-01	HNHM 95285	Montenegrina subcristata	KU307874			Montenegro	Podgorica		N42.4312	E19.253	--1966	Jakucs	Thomsen et al. (2009)	LCO1490/H2198-Alb			F1
Mwo-575-01	HNHM 83481	Montenegrina subcristata	KU307875			Montenegro	Tudjemili N 1 km		N42.1396	E19.1353	19-Apr-2000	Eross, Feher	Thomsen et al. (2009)	LCO1490/H2198-Alb			F1
Mwo-575-02	HNHM 83481	Montenegrina subcristata	KU307876			Montenegro	Tudjemili N 1 km		N42.1396	E19.1353	19-Apr-2000	Eross, Feher	Thomsen et al. (2009)	LCO1490/H2198-Alb			F1
Mwo-576-01	HNHM 95414	Montenegrina subcristata	KU307877			Montenegro	Virpazar		N42.244	E19.093	19-Apr-2000	Eross, Feher	Thomsen et al. (2009)	LCO1490/H2198-Alb			F1
Mwo-576-02	HNHM 95414	Montenegrina subcristata	KU307878			Montenegro	Virpazar		N42.244	E19.093	19-Apr-2000	Eross, Feher	Thomsen et al. (2009)	LCO1490/H2198-Alb			F1
Mwo-579-01	NHMW 110430/MN/0120	Montenegrina subcristata	KU307879			Montenegro	Sipcanik		N42.3707	E19.3139	25-May-2015	Deli, Eross, Feher	Geni-Al	LCO1490/H2198-Alb			F1
Mwo-580-02	NHMW 110430/MN/0121	Montenegrina subcristata	KU307880			Montenegro	Zabljak Crnojevica		N42.3172	E19.1567	25-May-2015	Deli, Eross, Feher	Geni-Al	LCO1490/H2198-Alb			F1
Mwo-580-03	NHMW 110430/MN/0121	Montenegrina subcristata	KU307881			Montenegro	Zabljak Crnojevica		N42.3172	E19.1567	25-May-2015	Deli, Eross, Feher	Geni-Al	LCO1490/H2198-Alb			F1
Mwo-581-01	NHMW 110430/MN/0122	Montenegrina subcristata	KU307882			Montenegro	Virpazar		N42.246	E19.0917	25-May-2015	Deli, Eross, Feher	Geni-Al	LCO1490/H2198-Alb			F1
Mwo-582-01	NHMW 110430/MN/0123	Montenegrina subcristata	KU307883			Montenegro	Godinje E 1 km		N42.2205	E19.1241	25-May-2015	Deli, Eross, Feher	Geni-Al	LCO1490/H2198-Alb			F1
Mwo-583-01	NHMW 110430/MN/0124	Montenegrina subcristata	KU307884			Montenegro	Virpazar SW 3.5 km to Gluhi Do		N42.2256	E19.0659	25-May-2015	Deli, Eross, Feher	Geni-Al	LCO1490/H2198-Alb			F1
Mwo-584-01	NHMW 110430/MN/0125	Montenegrina subcristata	KU307885			Montenegro	Virpazar N 0.5 km to Rijeka Crnojevica		N42.2513	E19.0873	25-May-2015	Deli, Eross, Feher	Geni-Al	LCO1490/H2198-Alb			F1
Mwo-585-01	NHMW 110430/MN/0126	Montenegrina subcristata	KU307886			Montenegro	Virpazar N 1.3 km to Rijeka Crnojevica		N42.2521	E19.0875	25-May-2015	Deli, Eross, Feher	Geni-Al	LCO1490/H2198-Alb			F1
Mwo-586-01	NHMW 110430/MN/0127	Montenegrina subcristata	KU307887			Montenegro	Virpazar N 3.5 km to Rijeka Crnojevica		N42.2547	E19.0896	25-May-2015	Deli, Eross, Feher	Geni-Al	LCO1490/H2198-Alb			F1
Mwo-598-01	NHMW 110430/MN/0139	Montenegrina subcristata	KU307888			Montenegro	Zoganje N 5 km		N41.9794	E19.2591	27-May-2015	Deli, Eross, Feher	Geni-Al	LCO1490/H2198-Alb			F1
Mwo-599-01	NHMW 110430/MN/0140	Montenegrina subcristata	KU307889			Montenegro	Klezna N 1 km		N42.004	E19.263	27-May-2015	Deli, Eross, Feher	Geni-Al	LCO1490/H2198-Alb			F1
Mwo-600-01	NHMW 110430/MN/0141	Montenegrina subcristata	KU307890			Albania	Vallas		N42.0506	E19.4162	27-May-2015	Deli, Eross, Feher	Geni-Al	LCO1490/H2198-Alb			F1
Mwo-601-01	NHMW 110430/MN/0142	Montenegrina subcristata	KU307891			Albania	above Vallas		N42.0524	E19.4182	27-May-2015	Deli, Eross, Feher	Geni-Al	LCO1490/H2198-Alb			F1
Mwo-603-01	NHMW 110430/MN/0143	Montenegrina subcristata	KU307892			Albania	Shkoder, Rozafa Hill		N42.0451	E19.4902	28-May-2015	Deli, Eross, Feher	Geni-Al	LCO1490/H2198-Alb			F1
Mwo-611-01	NHMW 110430/MN/0151	Montenegrina subcristata	KU307893			Albania	Zusi near Shkoder		N42.0386	E19.4811	01-May-2015	Reischutz	Geni-Al	LCO1490/H2198-Alb			F1
Mx1-228-01	HNHM 99594	Montenegrina hiltrudae maasseni	KU307894	KU308226	KU307994	Greece	Portitsa Farangi		N39.9963	E21.2855	15-Jul-2004	Eross, Hunyadi	Geni-Al	LCO1490/H2198-Alb	16SLOrc1_fwd/16SLOrc_rev	12SGastFw/12SGastRv	E
Mx2-236-01	HNHM 94913	Montenegrina tomorosi coerulescens	KU307895	KU308227		Albania	Tomorr Mts, 1.5 km S of Tomorr village		N40.6788	E20.1109	11-Aug-2004	Feher	Geni-Al	LCO1490/H2198-Alb	16SLOrc1_fwd/16SLOrc_rev		K
Mx3-470-01	NHMW 111222	Montenegrina prokletiana kovacsorum	KU307896	KU308228		Albania	3 km W of the Valbona mouth to Tetaj		N42.2623	E19.9911	26-Jun-2014	Angyal, Eross, Feher, Grego	Geni-Al	LCO1490/H2198-Alb	16SarF/16SLOrc_rev		G
Mx4-255-03	HNHM 99455	Montenegrina skipetarica puskasi	KU307897	KU308229		Albania	Cidhne W 1 km		N41.7521	E20.2524	12-Apr-2006	Eross, Feher, Hunyadi, Muranyi	Geni-Al	LCO1490/H2198-Alb	16SLOrc1_fwd/16SLOrc_rev		B
Mx4-256-04	HNHM 99456	Montenegrina skipetarica puskasi	KU307898	KU308230		Albania	Cidhne W 1.5 km		N41.7528	E20.2505	12-Apr-2006	Eross, Feher, Hunyadi, Muranyi	Geni-Al	LCO1490/H2198-Alb	16SLOrc1_fwd/16SLOrc_rev		B
Mx4-366-01	HNHM 99457	Montenegrina skipetarica puskasi	KU307899	KU308231		Albania	Cidhne W		N41.749	E20.255	08-Oct-2012	Juhasz, Kovacs, Muranyi, Puskas	Geni-Al	LCO1490/H2198-Alb	16SLOrc1_fwd/16SLOrc_rev		B
Mx4-619-01	HNHM 99451	Montenegrina skipetarica puskasi	KU307900			Albania	Arras	paratype	N41.7391	E20.297	01-Jul-2015	Eross, Feher, Grego	Geni-Al	LCO1490/H2198-Alb			B
Mx5-249-01	HNHM 99461	Montenegrina skipetarica gurelurensis	KU307901			Albania	Cidhne W 2 km	paratype	N41.7517	E20.26	10-Oct-2005	Eross, Muranyi	Geni-Al	LCO1490/H2198-Alb			B
Mx5-256-05	HNHM 99463	Montenegrina skipetarica gurelurensis	KU307902	KU308232		Albania	Cidhne W 1.5 km		N41.7528	E20.2505	12-Apr-2006	Eross, Feher, Hunyadi, Muranyi	Geni-Al	LCO1490/H2198-Alb	16SLOrc1_fwd/16SLOrc_rev		B
Mx5-256-07	HNHM 99463	Montenegrina skipetarica gurelurensis	KU307903	KU308233		Albania	Cidhne W 1.5 km		N41.7528	E20.2505	12-Apr-2006	Eross, Feher, Hunyadi, Muranyi	Geni-Al	LCO1490/H2198-Alb	16SLOrc1_fwd/16SLOrc_rev		B
Mx5-256-08	HNHM 99463	Montenegrina skipetarica gurelurensis	KU307904	KU308234		Albania	Cidhne W 1.5 km		N41.7528	E20.2505	12-Apr-2006	Eross, Feher, Hunyadi, Muranyi	Geni-Al	LCO1490/H2198-Alb	16SLOrc1_fwd/16SLOrc_rev		B
Mx5-257-02	HNHM 99460	Montenegrina skipetarica gurelurensis	KU307905	KU308235		Albania	Cidhne W 2 km		N41.7525	E20.2455	12-Apr-2006	Eross, Feher, Hunyadi, Muranyi	Geni-Al	LCO1490/H2198-Alb	16SLOrc1_fwd/16SLOrc_rev		B
Mx5-257-03	HNHM 99460	Montenegrina skipetarica gurelurensis	KU307906	KU308236		Albania	Cidhne W 2 km		N41.7525	E20.2455	12-Apr-2006	Eross, Feher, Hunyadi, Muranyi	Geni-Al	LCO1490/H2198-Alb	16SLOrc1_fwd/16SLOrc_rev		B
Mx6-352-01	HNHM 99492	Montenegrina prokletiana prokletiana	KU307907	KU308237	KU307995	Albania	Dragobi	paratype	N42.4364	E19.9847	12-Aug-2012	Barina, Somogyi	Geni-Al	LCO1490/H2198-Alb	16SarF/16SLOrc_rev	12SGastFw/12SGastRv	G
Mx6-352-02	HNHM 99492	Montenegrina prokletiana prokletiana	KU307908			Albania	Dragobi	paratype	N42.4364	E19.9847	12-Aug-2012	Barina, Somogyi	Geni-Al	LCO1490/H2198-Alb			G
Mx6-352-03	HNHM 99492	Montenegrina prokletiana prokletiana	KU307909			Albania	Dragobi	paratype	N42.4364	E19.9847	12-Aug-2012	Barina, Somogyi	Geni-Al	LCO1490/H2198-Alb			G
Mx7-262-02	HNHM 99501	Montenegrina sturanyi ostrovicensis	KU307910	KU308238		Albania	Ostrovica Mts, Maja e Faqekuqit	paratype	N40.5325	E20.4256	21-Aug-2006	Feher, Hunyadi, Huszar, Muranyi	Thomsen et al. (2009)	LCO1490/H2198-Alb	16SLOrc1_fwd/16SLOrc_rev		H
Mx7-262-03	HNHM 99501	Montenegrina sturanyi ostrovicensis		KU308239		Albania	Ostrovica Mts, Maja e Faqekuqit	paratype	N40.5325	E20.4256	21-Aug-2006	Feher, Hunyadi, Huszar, Muranyi	Thomsen et al. (2009)		16SLOrc1_fwd/16SLOrc_rev		H
Mx7-262-04	HNHM 99501	Montenegrina sturanyi ostrovicensis	KU307911			Albania	Ostrovica Mts, Maja e Faqekuqit	paratype	N40.5325	E20.4256	21-Aug-2006	Feher, Hunyadi, Huszar, Muranyi	Thomsen et al. (2009)	LCO1490/H2198-Alb			H
Mvo-391-01	HNHM 99531	Montenegrina skipetarica voidomatis			KU307992	Greece	Kleidonia S 2 km		N39.9674	E20.6646	26-Jun-2013	Eross, Feher, Grego	Geni-Al			12SGastFw/12SGastRv	C
Mx9-268-01	HNHM 99541	Montenegrina tomorosi tomorosi	KU307913	KU308241		Albania	Ujanik		N40.6328	E20.2162	23-Aug-2006	Feher, Hunyadi, Huszar, Muranyi	Geni-Al	LCO1490/H2198-Alb	16SLOrc1_fwd/16SLOrc_rev		K
Mx9-269-02	NHMW 111232	Montenegrina tomorosi hunyadii	KU307914	KU308242		Albania	Tomorr Mts, 1.5 km W of Terove	paratype	N40.7127	E20.1839	24-Aug-2006	Feher, Hunyadi, Huszar, Muranyi	Thomsen et al. (2009)	LCO1490/H2198-Alb	16SLOrc1_fwd/16SLOrc_rev		K
Mzi-371-01	HNHM 99598	Montenegrina zilchi	KU307915	KU308243	KU307996	Greece	Pili		N39.4603	E21.6008	22-Jun-2013	Eross, Feher, Grego	Geni-Al	LCO1490/H2198-Alb	16SLOrc1_fwd/16SLOrc_rev	12SGastFw/12SGastRv	E
Mzi-371-02	HNHM 99598	Montenegrina zilchi	KU307916	KU308244		Greece	Pili		N39.4603	E21.6008	22-Jun-2013	Eross, Feher, Grego	Geni-Al	LCO1490/H2198-Alb	16SLOrc1_fwd/16SLOrc_rev		E
Mzi-371-03	HNHM 99598	Montenegrina zilchi	KU307917	KU308245		Greece	Pili		N39.4603	E21.6008	22-Jun-2013	Eross, Feher, Grego	Geni-Al	LCO1490/H2198-Alb	16SLOrc1_fwd/16SLOrc_rev		E
Mzi-371-04	HNHM 99598	Montenegrina zilchi	KU307918			Greece	Pili		N39.4603	E21.6008	22-Jun-2013	Eross, Feher, Grego	Geni-Al	LCO1490/H2198-Alb			E

Table S1.3

Phylogeny-based		Morpho-taxonomy based	
3 groups	15 groups	27 species	
Clade G (12)	Clade G (12)	*M. lillae*	(2)
		M. okolensis	(2)
		M. prokletiana	(5)
		M. sporadica	(3)
Clade A-F (230)	Clade A (4)	*M. attemsi*	(4)
	Clade B (29)	*M. skipetarica* [1]	(29)
	Clade C (20)		(20)
	Clade D1 (31)	*M. rugilabris*	(20)
		M. fuchsi	(5)
		M. janinensis	(6)
	Clade D2 (31)	*M. hiltrudae* [7]	(1 of 26)
		M. stankovici	(6)
		M. dofleini [2]	(25)
	Clade E (30)		(4)
		M. hiltrudae [7]	(25 of 26)
		M. zilchi	(1)
	Clade F1 (83)	*M. subcristata*	(65)
	Clade F2 (18)	*M. cattaroensis*	(18)
	Clade F3 (2)	*M. haringae*	(2)
Clade H-L (141)	Clade H (26)	*M. perstriata* [3]	(17 of 40)
		M. sturanyi	(5)
		M. timeae	(4)
	Clade I (34)	*M. perstriata* [4]	(19 of 40)
		M. nana	(4)
		M. helvola	(12)
	Clade J (3)	*M. minuscula*	(3)
	Clade K (26)	*M. perstriata* [5]	(5 of 40)
		M. tomorosi	(16)
		M. grammica	(5)
	Clade L (53)	*M. perstriata* [6]	(9 of 40)
		M. laxa	(34)
		M. drimmeri	(1)
		M. soosi	(9)

site_no	site_code	prosobranch	Carychiidae	Ferussaciidae	Azeciidae	Cochlicopidae	Enidae	Euconulidae	Gastrodontidae	Oxychilidae	Pristilomatidae	Bradybaenidae	Cochlicellidae	Helicidae	Helicodontidae	Hygromiidae	Vitrinidae	Punctidae	Argnidae	Chondrinidae	Lauriidae	Orculidae	Pupillidae	Pyramidulidae
3	1992/22	1	0	0	0	0	1	0	0	1	1	0	0	1	0	1	0	0	0	1	0	0	0	0
16	1993/14	1	0	0	0	0	0	0	0	0	0	0	1	1	0	1	0	0	0	0	0	0	0	0
19	1993/16	1	0	0	0	0	0	0	0	1	0	0	0	1	0	1	1	0	0	0	0	0	0	0
20	1993/12	1	0	0	0	0	1	0	0	0	0	0	0	0	1	1	0	0	0	0	0	0	0	0
25	1994/53	0	0	0	0	0	1	0	0	1	1	0	0	1	0	1	0	0	0	0	0	0	0	0
28	1994/57	1	0	0	0	0	1	0	0	0	0	0	0	1	0	1	0	0	0	1	0	0	0	0
33	1994/62	0	0	0	0	0	0	0	0	0	0	0	0	0	0	0	0	0	0	0	0	0	0	0
34	1996/03	0	0	0	0	0	0	0	0	0	0	0	0	0	0	0	0	0	0	1	0	0	0	0
36	1996/05	1	0	0	0	0	0	0	0	0	0	0	0	0	0	0	0	0	0	1	0	0	0	0
38	1996/07	0	0	0	0	0	1	0	0	0	0	0	0	1	0	0	0	0	0	0	0	0	0	0
39	1996/08	0	0	0	0	0	0	0	0	0	0	0	0	0	0	1	0	0	0	0	0	0	0	0
41	1996/10	0	0	0	0	0	1	0	0	0	0	0	0	1	0	0	0	0	0	1	0	0	0	0
42	1996/11	0	0	0	0	0	0	0	0	0	0	0	0	0	0	0	0	0	0	0	0	0	0	0
43	1996/12	0	0	0	0	0	1	0	0	0	0	0	0	0	0	0	0	0	0	1	0	0	0	0
46	1996/15	1	0	0	0	0	0	0	0	0	0	0	0	1	0	0	0	0	0	1	0	0	0	0
48	1996/17	0	0	0	0	0	0	0	0	0	0	0	0	0	0	0	0	0	0	0	0	0	0	0
52	1996/21	1	0	0	0	0	0	0	0	0	0	0	0	1	0	1	0	0	0	0	0	0	0	0
53	1996/22	1	0	0	0	0	0	0	0	0	0	0	0	1	0	1	0	0	0	1	0	0	0	0
54	1996/23	0	0	0	0	0	0	0	0	0	0	0	0	0	0	1	0	0	0	0	0	0	0	0
57	1996/26	0	0	0	0	0	0	0	0	0	0	0	0	0	0	1	0	0	0	0	0	0	0	0
58	1996/27	1	0	0	0	0	0	0	0	0	0	0	0	0	1	1	0	0	0	0	0	0	0	0
59	1996/28	1	0	0	0	0	0	0	0	0	0	0	0	0	0	1	0	0	0	0	0	0	0	0
60	1996/29	1	0	0	0	0	0	0	0	0	0	0	0	0	0	1	0	0	0	0	0	0	0	0
61	1996/30	1	0	1	0	0	1	0	0	1	1	0	0	0	0	0	0	0	1	1	0	1	0	1
62	1996/31	1	0	0	0	0	1	0	0	0	1	0	0	0	0	0	0	0	1	0	0	0	0	0
64	1996/33	1	0	0	0	0	0	0	0	1	0	0	0	1	1	1	0	0	0	1	0	0	0	0
66	1996/35	1	0	0	1	0	0	0	0	0	1	0	0	1	0	1	0	0	0	1	0	0	0	0
70	1996/39	1	0	0	0	0	0	0	0	1	0	0	0	1	1	1	0	0	0	1	0	0	0	0
73	1996/42	1	0	0	0	0	0	0	0	0	0	0	0	0	0	0	0	0	0	0	0	0	0	0
74	1996/43	1	0	0	0	0	0	0	0	1	0	0	0	1	1	0	0	0	0	1	0	0	0	0
75	1996/44	1	0	0	0	0	0	0	0	0	0	0	0	0	0	0	0	0	0	0	0	0	0	0
77	1996/46	1	0	0	0	0	0	0	0	0	0	0	0	0	1	1	0	0	0	1	0	0	0	0
78	1996/47	1	0	0	0	0	0	0	0	0	0	0	0	0	0	0	0	0	0	0	0	0	0	0
79	1996/48	1	0	0	0	0	0	0	0	0	0	0	0	1	0	1	0	0	0	0	0	0	0	0
82	1996/51	1	0	0	0	0	0	0	0	1	0	0	0	0	0	1	0	0	0	1	0	0	0	1
83	1996/52	0	0	0	0	0	0	0	0	0	0	0	0	0	1	1	0	0	0	1	0	0	0	1
84	1996/53	1	0	0	0	0	1	0	0	0	0	0	0	1	0	1	0	0	0	0	0	0	0	1
85	1996/54	1	0	0	0	0	1	0	0	1	1	0	0	1	1	1	0	0	0	1	0	0	0	1
88	1996/57	1	0	0	0	0	1	0	0	0	0	0	0	0	1	1	0	0	0	1	0	0	0	1
89	1996/58	0	0	0	0	0	0	0	0	0	0	0	0	0	0	1	0	0	0	0	0	0	0	0
92	1996/61	0	0	0	0	0	1	0	0	0	0	0	0	0	0	0	0	0	0	0	0	0	0	0
94	1996/63	1	0	0	0	0	0	0	0	0	0	0	0	1	0	1	0	0	0	0	0	0	0	0
95	1996/64	1	0	0	0	0	0	0	0	0	0	0	1	0	1	1	0	0	0	0	0	0	0	0
96	1996/65	1	0	1	1	0	1	0	0	0	1	0	0	1	0	0	0	0	0	0	0	0	0	0
97	1996/66	1	0	1	1	0	0	0	0	0	0	0	0	0	1	1	0	0	0	1	0	0	0	0
98	1996/67	1	0	0	0	0	0	0	0	0	0	0	0	0	1	1	0	0	0	0	0	0	0	0
100	1996/69	1	0	0	0	0	0	0	0	0	0	0	0	0	1	1	0	0	0	1	0	0	0	0
102	1996/71	1	0	1	1	0	0	0	0	0	0	0	0	1	1	1	0	0	0	1	0	0	0	0
103	1996/72	1	0	0	0	0	1	0	0	0	0	0	0	0	0	1	0	0	0	1	0	0	0	0
105	1996/74	1	0	0	0	0	0	0	0	0	0	0	0	1	0	0	0	0	0	1	0	1	0	1
114	1996/83	0	0	0	0	0	1	0	0	0	0	0	0	0	0	1	1	0	0	1	0	1	0	0
115	2000/14	1	0	0	0	0	1	0	0	0	0	0	0	1	0	1	0	0	0	0	0	0	0	0
116	2000/15	0	0	0	0	0	0	0	0	0	0	0	0	0	0	0	0	0	0	0	0	0	0	0
117	2000/16	0	0	0	0	0	0	0	0	0	1	0	0	0	0	1	0	0	0	0	0	0	0	0
118	2000/17	1	0	0	0	0	1	0	0	0	1	0	0	1	0	0	1	1	0	0	0	0	0	0
119	2000/18	1	0	0	0	0	1	0	0	1	1	0	0	1	0	0	1	0	0	0	0	0	0	0
120	2000/19	0	0	0	0	0	0	0	0	0	0	0	0	0	0	0	0	0	0	1	0	0	0	0
122	2000/21	0	0	0	0	0	1	0	0	1	1	0	0	0	0	1	0	0	0	1	0	0	0	0
125	2000/23	1	0	0	0	0	0	0	0	0	0	0	0	1	0	1	1	0	0	0	0	0	0	0
126	2000/24	1	0	0	0	0	0	0	0	0	0	0	0	0	0	0	0	0	0	1	0	0	0	0
127	2000/25	0	0	0	0	0	0	0	0	0	0	0	0	0	0	1	0	0	0	0	0	0	0	0
128	2000/26	0	0	0	0	0	0	0	0	1	0	0	0	0	0	1	0	0	0	1	0	0	0	0
130	2000/28	0	0	0	0	0	1	0	0	0	0	0	0	1	0	1	1	0	0	0	0	0	0	0
131	2000/29	1	0	0	0	0	0	0	0	0	0	0	0	0	0	1	0	0	0	1	0	0	0	0
132	2000/30	0	0	0	0	0	0	0	0	1	0	0	0	0	0	0	1	0	0	0	0	0	0	0
134	2000/32	1	0	0	0	0	0	0	0	0	0	0	0	0	0	0	0	0	0	0	0	0	0	0
135	2000/33	1	0	0	0	0	0	0	0	0	0	0	0	0	0	0	0	0	0	0	0	0	0	0
136	2000/34	1	0	0	1	0	0	0	0	0	0	0	0	1	0	1	0	0	0	0	0	0	0	0
138	2000/35	1	0	0	0	0	1	0	0	1	1	0	0	1	0	1	0	0	0	1	0	0	0	0
141	2000/38	0	0	0	0	0	0	0	0	0	0	0	0	0	0	0	0	0	0	0	0	0	0	1
142	2000/39	1	0	0	0	0	1	0	0	0	1	0	0	0	0	0	0	0	0	0	0	0	0	0
146	2000/43	0	0	0	1	0	0	0	0	0	0	0	0	1	0	1	0	0	0	0	1	0	0	0
147	2000/44	1	0	0	1	0	1	0	0	0	1	0	0	1	0	1	0	1	0	1	0	1	0	0
148	2000/45	1	0	0	0	0	1	0	0	0	0	0	0	0	0	1	0	0	0	1	0	0	0	0
149	2000/46	1	0	0	1	0	1	0	0	0	0	0	0	1	1	1	0	0	0	1	0	0	0	0
150	2000/47	1	0	0	0	0	0	0	0	0	0	0	0	0	0	0	0	0	0	1	0	0	0	0
151	2000/48	1	0	0	0	0	0	0	0	0	0	0	0	0	0	0	0	0	0	0	0	0	0	0
152	2000/49	0	0	0	0	0	0	0	0	0	0	0	0	0	0	0	0	0	0	0	0	0	0	0
153	2000/50	1	0	0	1	0	1	0	0	0	0	0	0	0	0	1	0	0	0	0	0	0	0	0
157	2000/54	1	0	0	0	0	0	0	0	0	0	0	0	1	0	0	0	0	0	1	0	0	0	0
158	2000/55	1	0	0	0	0	1	0	0	1	0	0	0	1	0	1	0	0	0	1	0	0	0	0
159	2000/56	1	0	0	0	0	1	0	0	0	1	0	0	1	0	1	0	0	0	1	0	1	0	0
161	2000/58	1	0	0	0	0	0	0	0	0	0	0	0	1	0	1	0	0	0	0	0	0	0	0
164	2000/61	1	0	0	0	0	0	0	0	0	0	0	0	1	0	0	0	0	0	0	0	0	0	0
165	2001/002	0	0	0	0	0	0	0	0	0	0	0	0	1	0	1	0	0	0	1	0	0	0	0
168	2001/005	0	0	0	0	0	1	0	0	0	0	0	0	0	0	0	0	0	0	1	0	0	0	0
169	2001/006	0	0	0	0	0	0	0	0	0	0	0	0	0	0	1	0	0	0	0	0	0	0	0
170	2001/007	0	0	0	0	0	1	0	0	0	0	0	0	0	0	0	0	0	0	0	0	0	0	0
172	2001/009	1	0	0	0	0	0	0	0	0	0	0	0	0	0	1	0	0	0	0	0	0	0	0
173	2001/010	1	0	0	0	0	1	0	0	0	0	0	0	0	0	1	0	0	0	0	0	0	0	0
174	2001/011	1	0	0	0	0	1	0	0	0	1	0	0	1	0	1	0	0	0	0	0	0	0	0
176	2001/013	0	0	0	0	0	0	0	0	0	0	0	0	0	0	1	0	0	0	1	0	0	0	0
177	2001/014	1	0	0	0	0	0	0	0	1	0	0	0	0	1	1	0	0	0	1	0	0	0	0
178	2001/015	1	0	1	0	0	0	0	0	0	0	0	0	1	0	1	0	0	0	1	0	0	0	1
181	2001/018	1	0	0	0	0	1	0	0	1	0	0	0	1	0	1	0	0	0	1	0	0	0	0
182	2001/019	1	0	0	0	0	1	0	0	0	0	0	0	0	0	1	0	0	0	1	0	0	0	0
183	2001/020	1	0	0	0	0	0	0	0	0	1	0	0	1	0	0	0	0	0	1	0	0	0	0
187	2001/024	1	0	0	0	0	0	0	0	0	0	0	0	1	0	1	0	0	0	0	0	0	0	0
188	2001/025	1	0	0	0	0	0	0	0	0	0	0	0	0	1	1	0	0	0	0	0	0	0	0
189	2001/026	1	0	0	0	0	0	0	0	0	1	0	0	1	1	1	0	0	0	0	0	0	0	0
190	2001/027	1	0	0	0	0	0	0	0	1	0	0	0	1	0	0	0	0	0	0	0	0	0	0
191	2001/028	1	0	0	0	0	0	0	0	0	0	0	0	0	0	0	0	0	0	1	0	0	0	1
194	2001/031	1	0	0	0	0	0	0	0	1	1	0	0	1	1	1	0	0	0	1	0	0	0	1
196	2001/033	1	0	0	0	0	0	0	0	1	0	0	0	1	1	1	0	0	0	1	0	0	0	1
197	2001/034	0	0	0	0	0	0	0	0	0	0	0	0	0	0	0	0	0	0	0	0	0	0	0
199	2001/036	1	0	0	0	0	0	0	0	0	0	0	0	0	1	1	0	0	0	0	0	0	0	1
200	2001/037	0	0	0	0	0	1	0	0	0	0	0	0	0	1	1	0	0	0	0	0	0	0	0
201	2001/038	0	0	0	0	0	0	0	0	0	0	0	0	1	1	1	0	0	0	1	0	0	0	0
202	2001/039	1	0	0	0	0	0	0	0	0	0	0	0	0	0	0	0	0	0	1	0	0	0	0
203	2001/040	0	0	0	0	0	0	0	0	0	0	0	0	0	1	1	0	0	0	1	0	0	0	0
206	2001/042	0	0	0	0	0	0	0	0	0	0	0	0	0	0	0	0	0	0	0	0	0	0	1
208	2001/044	1	0	0	0	0	0	0	0	0	0	0	0	0	1	1	0	0	0	1	0	0	0	0
209	2001/045	1	0	0	0	0	1	0	0	1	0	0	0	0	1	1	0	0	0	0	0	0	0	1
216	2002/095	1	0	0	0	0	1	0	0	0	0	0	0	0	0	1	0	0	0	0	0	0	0	0
218	2002/097	1	0	0	0	0	1	0	0	1	0	0	0	1	1	1	0	0	0	1	0	1	0	0
219	2002/098	1	0	0	0	0	0	0	0	0	0	0	0	1	0	0	0	0	0	0	0	1	0	0
223	2002/102	0	0	0	0	0	1	0	0	0	0	0	0	0	0	0	0	0	0	0	0	0	0	0
226	2002/105	1	0	0	0	0	1	0	0	0	0	0	0	1	1	1	1	0	0	1	0	1	0	1
227	2002/106	0	0	0	0	0	0	0	0	1	0	0	0	0	0	0	0	0	0	0	0	0	0	0
228	2002/107	1	0	0	0	0	0	0	0	0	0	0	0	0	1	1	0	0	0	1	0	0	0	0
229	2002/108	1	0	0	0	0	0	0	0	1	0	0	0	0	1	1	0	1	0	1	0	1	0	0
230	2002/109	1	0	0	0	0	0	0	0	0	0	0	0	1	0	1	1	0	0	1	0	1	0	0
232	2002/111	1	0	0	0	0	1	0	0	0	0	0	0	0	0	1	0	0	0	1	0	1	0	1
233	2002/112	1	0	0	0	0	1	0	0	0	0	0	0	1	1	1	1	0	0	1	0	0	0	1
237	2002/116	0	0	0	0	0	0	0	0	0	0	0	0	0	1	1	0	0	0	0	0	0	0	0
244	2002/123	0	0	0	0	0	1	0	0	1	0	0	0	0	0	1	0	0	0	1	0	0	0	0
253	2002/132	1	0	0	0	0	1	0	1	1	0	0	0	1	0	1	0	0	0	0	0	0	0	0
256	2002/135	0	0	0	0	0	1	0	0	0	0	0	0	0	0	0	0	0	0	0	0	0	0	0
257	2002/136	0	0	0	0	0	1	0	0	0	0	0	0	0	0	1	0	0	0	1	0	0	0	0
260	2002/139	0	0	0	0	0	1	0	0	0	1	0	0	1	1	1	0	0	0	1	0	0	0	0
263	2002/142	1	0	0	0	0	0	0	0	1	0	0	0	1	0	0	0	0	0	1	0	1	0	1
264	2002/143	1	0	0	0	0	0	0	0	0	0	0	0	1	0	0	0	0	0	0	0	0	0	0
265	2002/144	1	0	0	0	0	1	0	0	0	0	0	0	1	1	0	0	0	0	0	0	0	0	0
267	2002/146	1	0	0	0	0	1	0	0	0	0	0	0	0	0	1	0	0	0	0	0	0	0	0
268	2002/147	1	0	0	0	0	1	0	0	1	0	0	0	0	0	1	1	0	0	0	0	0	0	1
269	2002/148	0	0	0	0	0	0	0	0	0	0	0	0	1	0	1	0	0	0	1	0	0	0	0
270	2003/013	0	0	0	0	0	0	0	0	0	0	0	0	0	0	0	0	0	0	1	0	0	0	0
272	2003/015	1	0	0	0	0	1	0	0	1	1	0	0	0	0	0	0	0	0	0	0	0	0	1
277	2003/020	0	0	0	0	0	0	0	0	0	0	0	0	0	0	1	0	0	0	0	0	0	0	0

site_no	site_code	Strobilopsidae	Valloniidae	Vertiginidae	Succineidae	Oleacinidae	Zonitidae	Clausiliidae	Agathylla	Albinaria	Alopia	Carinigera	Inchoatia	Cochlodina	Delima	Dilataria	Herilla	Isabellaria	Macedonica	Medora	Papillifera	Charpentieria	Strigilodelima	Triloba	Montenegrina	habitat_type	Latitude	Longitude
3	1992/22	0	0	0	0	1	0	0	0	0	0	0	0	0	1	0	0	0	0	1	0	0	0	0	1	1	41.518	19.8008
16	1993/14	0	0	0	0	0	0	0	0	0	0	0	0	0	0	0	0	0	0	0	0	1	0	0	0	1	39.8644	20.0153
19	1993/16	0	0	0	0	1	0	0	0	0	0	0	0	0	0	0	0	0	0	0	0	1	0	0	0	1	40.25030556	20.06522222
20	1993/12	0	0	0	0	1	0	0	0	0	0	0	0	0	1	0	0	0	0	0	0	0	0	0	0	2	41.35	19.89
25	1994/53	0	0	0	0	0	0	0	0	0	0	0	0	0	1	0	0	0	0	0	0	0	0	0	1	1	41.5085	19.7961
28	1994/57	0	0	0	0	1	1	0	0	0	0	0	0	0	1	0	0	0	0	0	0	1	1	0	0	1	41.3603	19.8916
33	1994/62	0	0	0	0	0	0	0	0	0	0	0	0	0	1	0	0	0	0	0	0	0	0	0	0	1	41.8677	19.5665
34	1996/03	0	0	0	0	0	0	0	0	0	0	0	0	0	0	0	0	0	0	0	0	0	0	0	0	1	43.9511	19.989
36	1996/05	0	0	0	0	0	0	0	0	0	0	0	0	0	0	0	0	0	0	0	0	0	0	0	0	1	43.5988	19.7196
38	1996/07	0	0	0	0	0	1	0	0	0	0	0	0	0	0	0	0	0	0	0	0	0	0	0	0	1	43.36	19.58
39	1996/08	0	0	0	0	0	1	1	0	0	0	0	0	0	0	0	0	0	0	0	0	0	0	0	0	1	43.34	19.31
41	1996/10	0	0	0	0	0	0	0	0	0	0	0	0	0	0	0	1	0	0	0	0	0	0	0	0	1	43.02238333	19.15628333
42	1996/11	0	0	0	0	0	0	1	0	0	0	0	0	1	0	0	0	0	0	0	0	0	0	0	0	1	43.0003	19.1601
43	1996/12	0	0	0	0	0	0	0	0	0	0	0	0	0	0	0	1	0	0	0	0	0	0	0	0	1	42.9656	19.0952
46	1996/15	0	0	0	0	0	0	1	0	0	0	0	0	0	1	0	1	0	0	0	0	0	0	0	0	1	42.94591667	19.08355
48	1996/17	0	0	0	0	0	1	0	0	0	0	0	0	0	1	0	0	0	0	0	0	0	0	0	0	1	42.8981	19.0534
52	1996/21	0	0	0	0	0	0	0	0	0	0	0	0	0	0	0	0	0	0	0	0	1	0	0	0	1	42.5008	18.6927
53	1996/22	0	0	0	0	1	0	0	0	0	0	0	0	0	0	0	0	0	0	0	0	0	0	0	1	1	42.4213	18.7716
54	1996/23	0	0	0	0	0	0	0	0	0	0	0	0	0	0	0	0	0	0	0	0	0	0	0	0	2	42.1947	18.967
57	1996/26	0	0	0	0	0	0	0	0	0	0	0	0	0	1	0	0	0	0	0	0	0	0	0	0	1	42.2715	19.1254
58	1996/27	0	0	0	0	1	0	0	0	0	0	0	0	0	1	0	0	0	0	0	0	0	0	0	1	1	42.3732	19.3193
59	1996/28	0	0	0	0	0	1	0	0	0	0	0	0	0	0	0	0	0	0	0	0	0	0	0	1	1	42.3193	19.3696
60	1996/29	0	0	0	0	1	0	0	0	0	0	0	0	0	1	0	0	0	0	0	0	0	0	0	0	1	42.3741	19.4647
61	1996/30	0	0	0	0	1	0	0	0	0	0	0	0	0	1	0	0	0	0	0	0	1	0	0	0	1	42.41333333	19.50385
62	1996/31	0	0	0	0	1	0	0	0	0	0	0	0	0	1	0	0	0	0	0	0	0	0	0	0	1	42.3315	19.6005
64	1996/33	0	0	0	0	1	0	0	0	0	0	0	0	0	1	0	0	0	0	0	0	0	0	0	1	1	42.048	19.4976
66	1996/35	0	0	0	0	0	1	0	0	0	0	0	0	0	1	0	0	0	0	0	0	1	0	0	1	1	41.90006667	19.51298333
70	1996/39	0	0	0	0	1	1	0	0	0	0	0	0	0	0	0	0	0	0	1	0	0	1	0	0	1	41.3599	19.9146
73	1996/42	0	0	0	0	0	0	0	0	1	0	0	0	0	0	0	0	0	0	0	0	0	0	0	0	1	40.2627	19.5315
74	1996/43	0	0	0	0	1	1	0	0	0	0	0	0	0	0	0	0	0	0	0	0	0	0	0	0	3	40.2055	19.591
75	1996/44	0	0	0	0	0	0	0	0	0	0	0	0	0	0	0	0	0	0	0	0	0	0	0	0	1	40.1904	19.6049
77	1996/46	0	0	0	0	1	0	0	0	1	0	0	0	0	0	0	0	0	0	0	0	1	0	0	0	1	40.069	19.8588
78	1996/47	0	0	0	0	0	0	0	0	0	0	0	0	0	0	0	0	0	0	0	0	1	0	0	0	1	39.9195	20.1404
79	1996/48	0	0	0	0	1	0	0	0	1	0	0	0	0	0	0	0	0	0	0	0	1	0	0	0	1	39.9479	20.25
82	1996/51	0	0	0	0	1	0	0	0	1	0	0	0	0	0	0	0	0	0	0	0	1	1	0	1	1	40.2086	20.4147
83	1996/52	0	0	0	0	0	0	0	1	0	0	0	0	0	0	0	0	0	0	0	0	0	1	0	0	1	40.1608	20.5682
84	1996/53	0	0	0	0	1	0	0	0	0	0	0	0	0	0	0	0	0	0	0	0	0	0	0	0	1	40.2441	20.6386
85	1996/54	0	0	0	0	1	0	0	0	0	0	0	0	0	0	0	0	0	0	0	0	0	0	0	1	1	40.2656	20.59991667
88	1996/57	0	0	0	0	0	0	0	0	0	0	0	0	0	0	0	0	0	0	0	0	0	1	0	0	1	40.9029	20.7298
89	1996/58	0	0	0	0	0	0	0	0	0	0	0	0	0	0	0	0	0	0	0	0	0	0	0	0	1	43.6308	20.5434
92	1996/61	0	0	0	0	0	0	0	0	0	0	1	0	0	0	0	0	0	0	0	0	0	0	0	0	1	40.44	21.34
94	1996/63	0	0	0	0	1	0	0	0	0	0	0	0	0	0	0	0	0	0	0	0	0	0	0	0	1	39.5603	20.469
95	1996/64	0	0	0	0	0	0	0	0	0	0	0	0	0	0	0	0	0	0	0	1	1	0	0	0	1	39.704	19.806
96	1996/65	0	0	0	0	1	0	1	0	1	0	0	0	0	0	0	0	0	0	0	0	0	0	0	0	1	39.72	19.87
97	1996/66	0	0	0	0	1	0	0	0	1	0	0	0	0	0	0	0	0	0	0	0	0	0	0	0	1	39.73	19.9
98	1996/67	0	0	0	0	0	0	0	0	1	0	0	0	0	0	0	0	0	0	0	0	0	0	0	0	1	39.736	19.798
100	1996/69	0	0	0	0	1	0	0	0	0	0	0	0	0	0	0	0	0	0	0	0	0	0	0	0	1	39.7054	19.7611
102	1996/71	0	0	0	0	1	0	0	0	1	0	0	0	0	0	0	0	0	0	0	0	0	0	0	0	1	39.6436	20.3133
103	1996/72	0	0	0	0	1	0	0	0	0	0	0	1	0	0	0	0	0	0	0	0	0	0	0	0	1	39.5	20.52
105	1996/74	0	0	0	0	0	0	0	0	0	0	0	0	0	0	0	0	0	0	0	0	0	1	0	0	1	39.866	20.717
114	1996/83	0	0	0	0	0	0	1	0	0	0	0	0	0	0	0	0	0	0	0	0	0	0	0	0	1	41.7	20.742
115	2000/14	0	0	0	0	0	0	0	0	0	0	0	0	0	0	0	1	0	0	0	0	0	0	0	0	1	43.28956667	18.85161667
116	2000/15	0	0	0	0	0	0	0	0	0	0	0	0	0	0	0	1	0	0	0	0	0	0	0	0	1	43.2737	18.8428
117	2000/16	0	0	0	0	0	0	0	0	0	0	0	0	0	0	0	1	0	0	0	0	0	0	0	0	1	43.1717	18.8519
118	2000/17	0	0	0	0	0	0	0	0	0	0	0	0	0	0	0	0	0	0	0	0	0	0	0	0	1	42.97508333	18.90563333
119	2000/18	0	0	0	0	0	1	1	0	0	0	0	0	1	0	0	1	0	0	0	0	0	0	0	0	1	42.8932	19.07138333
120	2000/19	0	0	0	0	0	1	0	0	0	0	0	0	0	1	0	0	0	0	0	0	0	0	0	0	1	42.92766667	19.0712
122	2000/21	0	0	0	0	0	0	0	0	0	0	0	0	0	0	0	1	0	0	0	0	0	0	0	0	1	42.9424	19.10423333
125	2000/23	0	0	0	0	0	0	1	0	0	0	0	0	1	0	0	1	0	0	0	0	0	0	0	0	1	43.151	19.27883333
126	2000/24	0	0	0	0	0	0	1	0	0	0	0	0	0	0	0	1	0	0	0	0	0	0	0	0	1	43.1358	19.2999
127	2000/25	0	0	0	0	0	0	0	0	0	0	0	0	0	0	0	1	0	0	0	0	0	0	0	0	1	43.1241	19.3246
128	2000/26	0	0	0	0	0	1	1	0	0	0	0	0	0	0	0	1	0	0	0	0	0	0	0	0	1	43.0726	19.38336667
130	2000/28	0	0	0	0	0	0	0	0	0	0	0	0	1	0	0	0	0	0	0	0	0	0	0	0	1	42.88908333	19.53473333
131	2000/29	0	0	0	0	0	0	0	0	0	0	0	0	0	0	0	0	0	0	0	0	0	0	0	0	1	42.852	19.5271
132	2000/30	0	0	0	0	0	0	1	0	0	0	0	0	0	0	0	0	0	0	0	0	0	0	0	0	3	42.7415	19.6898
134	2000/32	0	0	0	0	0	0	0	0	0	0	0	0	0	0	0	0	0	0	0	0	0	0	0	0	1	42.707	19.3771
135	2000/33	0	0	0	0	0	0	0	0	0	0	0	0	0	0	0	0	0	0	1	0	0	0	0	0	1	42.6908	19.3796
136	2000/34	0	0	0	0	0	1	0	0	0	0	0	0	0	1	0	0	0	0	0	0	0	0	0	1	1	42.2436	19.0952
138	2000/35	0	0	0	0	1	1	0	0	0	0	0	0	0	1	0	0	0	0	0	0	0	0	0	1	1	42.27651667	19.07088333
141	2000/38	0	0	0	0	0	0	0	0	0	0	0	0	0	0	0	0	0	0	0	0	0	0	0	0	1	42.1487	19.12
142	2000/39	0	0	0	0	0	0	0	0	0	0	0	0	0	1	0	0	0	0	0	0	0	0	0	1	1	42.141	19.1377
146	2000/43	0	0	0	0	1	0	0	0	0	0	0	0	0	1	0	0	0	0	0	1	1	0	0	0	1	41.9247	19.2049
147	2000/44	0	1	1	0	1	1	0	0	0	0	0	0	0	1	0	0	0	0	0	0	1	0	0	1	1	41.979361	19.259112
148	2000/45	0	0	0	0	1	1	0	0	0	0	0	0	0	0	0	0	0	0	0	0	1	0	0	1	1	42.002	19.2639
149	2000/46	0	0	0	0	1	1	0	0	0	0	0	0	0	1	0	0	0	0	0	0	0	0	0	1	1	41.9864	19.3156
150	2000/47	0	0	0	0	0	1	0	0	0	0	0	0	0	1	0	0	0	0	0	0	1	0	0	0	1	42.04765	19.3229
151	2000/48	0	0	0	0	0	0	0	0	0	0	0	0	0	0	0	0	0	0	0	0	0	0	0	0	1	42.0645	19.13193333
152	2000/49	0	0	0	0	0	0	0	0	0	0	0	0	0	1	0	0	0	0	0	0	1	0	0	0	1	42.2048	18.9575
153	2000/50	0	0	0	0	1	0	0	0	0	0	0	0	0	1	0	0	0	0	0	0	0	0	0	0	1	42.2766	18.8869
157	2000/54	0	0	0	0	1	1	0	0	0	0	0	0	0	0	0	0	0	0	0	0	0	0	0	0	1	42.42026667	18.7938
158	2000/55	0	0	0	0	0	1	0	1	0	0	0	0	0	0	0	0	0	0	1	0	0	0	0	0	1	42.42156667	18.79798333
159	2000/56	0	0	0	0	0	1	0	0	0	0	0	0	0	1	0	0	0	0	0	0	0	0	0	1	1	42.434	18.8189
161	2000/58	0	0	0	0	0	0	0	1	0	0	0	0	0	0	0	0	0	0	0	0	1	0	0	0	1	42.4744	18.5362
164	2000/61	0	0	0	0	0	1	0	0	0	0	0	0	0	0	0	0	0	0	0	0	0	0	0	0	1	42.59	18.67
165	2001/002	0	0	0	0	0	0	1	0	0	0	0	0	0	0	0	0	0	0	0	0	0	0	0	0	1	43.96716667	19.96116667
168	2001/005	0	0	0	0	0	0	0	0	0	0	0	0	0	0	0	0	0	0	0	0	0	0	0	0	1	43.33	19.669
169	2001/006	0	0	0	0	0	0	0	0	0	0	0	0	0	0	0	0	0	0	0	0	0	0	0	0	1	43.2659	19.7142
170	2001/007	0	0	0	0	0	0	1	0	0	0	0	0	0	0	0	0	0	0	0	0	0	0	0	0	1	43.89483333	19.53866667
172	2001/009	0	0	0	0	0	0	0	0	0	0	0	0	0	1	0	0	0	0	1	0	0	0	0	0	1	42.697	19.374
173	2001/010	0	0	0	0	1	1	0	0	0	0	0	0	0	0	0	0	0	0	1	0	1	0	0	0	1	42.614	19.39
174	2001/011	0	0	0	0	1	1	0	0	0	0	0	0	0	1	0	0	0	0	0	0	0	0	0	0	1	42.51733333	19.19566667
176	2001/013	0	0	0	0	0	0	0	0	0	0	0	0	0	1	0	0	0	0	0	0	0	0	0	0	1	42.11816667	19.57583333
177	2001/014	0	0	0	0	0	0	0	1	0	0	0	0	0	1	0	0	0	0	0	0	0	0	0	1	1	42.0446	19.4945
178	2001/015	0	0	0	0	1	0	0	1	0	0	0	0	0	1	0	0	0	0	0	0	0	0	0	0	1	42.01583333	19.62183333
181	2001/018	0	0	0	0	1	0	0	0	0	0	0	0	0	0	0	0	0	0	0	0	0	0	0	0	1	41.8895	20.12083333
182	2001/019	0	0	0	0	0	1	0	0	0	0	0	0	0	0	0	0	0	0	0	0	0	0	0	0	1	41.92016667	20.14516667
183	2001/020	0	0	0	0	1	0	0	0	0	0	0	0	0	1	0	0	0	0	0	0	0	0	0	0	1	41.76433333	19.7815
187	2001/024	0	0	0	0	0	0	0	0	0	0	0	0	0	1	0	0	0	0	0	0	0	0	0	1	1	41.37963	19.8607
188	2001/025	0	0	0	0	0	0	0	0	0	0	0	0	0	1	0	0	0	0	0	0	1	1	0	1	1	41.3859	19.861
189	2001/026	0	0	0	0	0	0	0	0	0	0	0	0	0	1	0	0	0	0	0	0	0	0	0	1	1	41.39416667	19.88433333
190	2001/027	0	0	0	0	1	1	0	0	0	0	0	0	0	0	0	0	0	0	1	0	0	0	0	1	1	41.40216667	19.90116667
191	2001/028	0	0	0	0	0	0	0	0	0	0	0	0	0	0	0	0	0	0	1	0	0	0	0	0	1	41.35466667	19.916
194	2001/031	0	0	0	0	1	0	0	1	0	0	0	0	0	0	0	0	0	0	1	0	0	1	0	0	1	41.23483333	19.97716667
196	2001/033	0	0	0	0	0	0	0	1	0	0	0	0	0	0	0	0	0	0	0	0	0	1	0	0	1	40.4698	20.2561
197	2001/034	0	0	0	0	0	0	0	0	0	0	0	0	0	0	0	0	0	0	0	0	0	1	0	0	1	40.49466667	20.2705
199	2001/036	0	0	0	0	1	0	0	0	0	0	0	0	0	0	0	0	0	0	0	0	1	1	0	0	1	40.6972	19.9535
200	2001/037	0	0	0	0	0	0	0	0	0	0	0	0	0	0	0	0	0	0	0	0	0	0	0	0	2	40.5865	19.99666667
201	2001/038	0	0	0	0	1	0	0	0	0	0	0	0	0	0	0	0	0	0	0	0	0	1	0	0	1	40.542	20.007
202	2001/039	0	0	0	0	0	0	0	1	0	0	0	0	0	0	0	0	0	0	0	0	0	1	0	0	1	40.50283333	19.9845
203	2001/040	0	0	0	0	1	0	0	0	0	0	0	0	0	0	0	0	0	0	0	0	0	1	0	0	1	40.34783333	20.16016667
206	2001/042	0	0	0	0	0	0	0	0	1	0	0	0	0	0	0	0	0	0	0	0	0	1	0	0	1	40.23683333	20.35183333
208	2001/044	0	0	0	0	0	0	0	0	0	0	0	0	0	0	0	0	0	0	0	0	1	0	0	0	1	40.29366667	20.01716667
209	2001/045	0	0	0	0	0	0	0	0	1	0	0	0	0	0	0	0	0	0	0	0	1	1	0	0	1	40.30133333	20.02166667
216	2002/095	0	0	0	0	0	1	0	0	0	0	0	0	0	1	0	0	0	0	0	0	0	0	0	0	1	42.3201	19.58575
218	2002/097	0	1	0	0	1	1	1	0	0	0	0	0	0	1	0	0	0	0	0	0	0	1	0	0	3	42.39205	19.70716667
219	2002/098	0	1	0	0	0	0	0	0	0	0	0	0	0	1	0	0	0	0	0	0	0	0	0	1	1	42.39228333	19.7297
223	2002/102	0	0	0	0	0	0	0	0	0	0	0	0	0	1	0	0	0	0	0	0	0	0	0	0	1	41.72	19.77
226	2002/105	0	0	0	0	0	1	0	0	0	0	0	0	0	0	0	0	0	0	0	0	0	1	0	0	1	41.83253333	20.10056667
227	2002/106	0	0	0	0	0	0	0	0	0	0	0	0	0	1	0	0	0	0	0	0	1	0	0	0	1	41.34731667	19.89703333
228	2002/107	0	0	0	0	1	0	0	0	0	0	0	0	0	0	0	0	0	0	0	0	0	1	0	0	1	41.32651667	19.94403333
229	2002/108	0	0	0	0	1	0	0	0	0	0	0	0	0	0	0	0	0	0	0	0	0	1	0	0	1	41.32681667	19.95755
230	2002/109	0	0	0	0	0	0	0	0	0	0	0	0	0	0	0	0	0	0	0	0	0	1	0	0	1	41.34036667	19.95628333
232	2002/111	0	0	0	0	1	0	0	0	0	0	0	0	0	0	0	0	0	0	0	0	0	1	0	0	1	41.37216667	20.005
233	2002/112	0	0	0	0	0	0	0	0	0	0	0	0	0	1	0	0	0	0	0	0	0	1	0	0	1	41.35913333	20.0273
237	2002/116	0	0	0	0	0	0	0	0	0	0	0	0	0	0	0	0	0	0	0	0	0	1	0	0	1	41.19851667	19.93473333
244	2002/123	0	0	0	0	1	0	0	0	0	0	0	0	0	0	0	0	0	0	0	0	0	1	0	1	1	41.16566667	20.23773333
253	2002/132	0	0	0	1	0	0	0	0	0	0	0	0	0	0	0	0	0	0	0	0	0	0	0	1	1	41.51596667	20.3906
256	2002/135	0	0	0	0	0	0	0	0	0	0	0	0	0	0	0	0	0	0	0	0	0	1	0	0	1	41.42086667	20.47971667
257	2002/136	0	0	0	0	0	0	0	0	0	0	0	0	0	0	0	0	0	0	0	0	0	1	0	1	1	41.41833333	20.45686667
260	2002/139	0	0	0	0	1	1	0	0	0	0	0	0	0	0	0	0	0	0	0	0	0	0	0	1	1	41.47181667	20.0967
263	2002/142	0	0	0	0	1	1	0	0	0	0	0	0	0	0	0	0	0	0	0	0	0	0	1	0	3	41.52085	19.8983
264	2002/143	0	0	0	0	0	0	0	0	0	0	0	0	0	0	0	0	0	0	1	0	0	0	0	0	1	41.52058333	19.85606667
265	2002/144	0	0	0	0	0	0	0	0	0	0	0	0	0	0	0	0	0	0	1	0	0	0	0	1	1	41.5229	19.81645
267	2002/146	0	0	0	0	1	1	0	0	0	0	0	0	0	1	0	0	0	0	1	0	1	0	0	0	1	42.65166667	19.37838333
268	2002/147	0	0	0	0	1	1	1	0	0	0	0	0	0	1	0	0	0	0	0	0	0	0	0	0	1	42.766	19.389
269	2002/148	0	0	0	0	0	0	1	0	0	0	0	0	1	0	0	0	0	0	0	0	0	0	0	0	1	43.1552	19.78068333
270	2003/013	0	0	0	0	0	0	0	0	0	0	0	0	0	0	0	0	0	0	0	0	0	0	0	0	1	43.97628333	19.9338
272	2003/015	0	0	0	0	1	1	0	0	0	0	0	0	0	0	0	0	0	0	0	0	1	0	0	0	1	42.47108333	19.3651
277	2003/020	0	0	0	0	0	0	0	0	0	0	0	0	0	0	0	0	0	0	0	0	0	0	0	0	1	42.06425	20.49298333

278 2003/021		1	0	0	0	0	0	0	0	0	0	0	0	0	0	1	0	0	0	0	0	0	0	0	0	0	0	0	0	0	0	0	0	0	0	0	0	0	0	0	0	0	0	0	0	0	0	0	1	42.07275	20.4599
281 2003/023		1	0	0	0	0	1	0	0	1	1	0	0	1	1	1	1	0	0	1	0	1	0	0	0	0	0	0	1	0	0	0	0	0	0	0	0	1	0	0	0	0	0	0	0	1	0	1	1	41.95983333	20.39375
286 2003/028		0	0	0	0	0	1	0	0	0	0	0	0	0	0	0	0	0	0	1	0	0	0	1	0	0	0	0	0	0	0	0	0	0	0	0	0	0	0	0	0	0	0	0	0	0	0	0	1	41.63225	20.2217
289 2003/030		0	0	0	0	0	0	0	0	0	0	0	0	0	0	0	0	0	0	1	0	0	0	0	0	0	0	0	0	0	0	0	0	0	0	0	0	0	0	0	0	0	0	0	0	1	1	0	1	41.64333333	20.15716667
293 2003/034		1	0	0	0	0	1	0	0	0	0	0	0	1	0	1	0	0	0	1	0	0	0	1	0	0	0	0	1	0	0	0	0	0	0	0	0	1	0	0	0	0	0	0	0	0	0	0	1	41.69501667	19.81418333
294 2003/035		1	0	0	0	0	0	0	0	0	0	0	0	1	0	1	0	0	0	0	0	0	0	0	0	0	0	0	0	0	0	0	0	0	0	0	0	0	0	0	0	0	0	0	0	0	0	0	1	41.76071667	19.77716667
295 2003/036		1	0	0	0	0	1	0	0	0	0	0	0	1	0	0	0	0	0	1	0	0	0	0	0	0	0	0	1	0	0	0	0	0	0	0	0	0	0	0	0	0	0	0	0	0	0	1	1	41.78833333	20.10681667
296 2003/037		1	0	0	0	0	1	0	0	0	0	0	0	1	0	0	0	0	0	1	0	0	0	1	0	0	0	0	1	0	0	0	0	0	0	0	0	0	0	0	0	0	0	0	0	0	0	0	1	41.83611667	20.17396667
297 2003/037B		0	1	0	0	0	0	1	0	1	0	0	0	1	0	0	0	0	0	0	0	1	0	1	0	1	1	0	0	0	0	0	0	0	0	0	0	0	0	0	0	0	0	0	0	1	0	0	1	41.83174	20.17713
302 2003/041		1	0	0	0	0	1	0	0	0	0	0	0	1	0	0	0	0	0	1	0	0	0	1	0	0	0	0	0	0	0	0	0	0	0	0	0	0	0	0	0	0	0	0	0	0	0	1	1	41.79415	20.11205
303 2003/042		1	0	0	0	0	1	0	0	0	1	0	0	1	1	1	0	0	0	1	0	0	0	1	0	0	0	0	1	1	0	1	0	0	0	0	0	0	0	0	0	0	0	0	0	1	0	0	1	41.2278	19.98141667
305 2003/044		1	0	0	0	0	0	0	0	1	1	0	0	1	1	1	0	0	0	1	0	0	0	1	0	0	0	0	1	0	0	0	0	0	0	0	0	0	0	0	0	0	0	0	0	1	0	1	1	40.92798333	20.03055
306 2003/045		0	0	0	0	0	0	0	0	0	0	0	0	1	0	1	0	0	0	0	0	0	0	0	0	0	0	0	0	0	0	0	0	0	0	0	0	0	0	0	0	0	0	0	0	1	0	0	1	41.11203333	20.08118333
308 2003/047		1	0	0	0	0	1	0	0	1	1	0	0	1	1	1	0	0	0	1	0	0	0	0	0	0	0	0	1	0	0	0	0	0	0	0	0	0	0	0	0	0	0	0	1	1	0	1	1	41.1336	20.12776667
309 2003/048		1	0	0	0	0	1	0	0	0	0	0	0	0	0	0	0	0	0	1	0	0	0	1	0	0	0	0	0	0	0	0	0	0	0	0	0	0	0	0	0	0	0	0	0	1	0	1	1	41.16756667	20.24983333
310 2003/049		1	0	0	0	0	1	0	0	0	0	0	0	1	0	0	0	0	0	0	0	0	0	1	0	0	0	0	0	0	0	0	0	0	0	0	0	0	0	0	0	0	0	0	0	0	1	0	1	41.09621667	20.44281667
312 2003/050		1	0	0	0	0	1	0	0	1	1	0	0	0	0	1	0	0	1	1	0	0	0	0	0	0	0	0	0	0	0	0	0	0	0	0	0	0	0	0	0	0	0	0	1	1	0	1	1	41.0937	20.44025
313 2003/051		1	0	0	0	0	1	0	0	0	0	0	0	0	0	0	0	0	0	1	0	0	0	0	0	0	0	0	0	0	0	0	0	0	0	0	0	0	0	0	0	0	0	0	0	1	0	0	1	41.06506667	20.59711667
318 2003/056		1	0	0	0	0	0	0	0	1	0	0	0	1	0	1	1	0	0	1	0	0	0	1	0	0	0	0	0	1	0	0	0	0	0	0	0	0	0	0	0	0	0	0	0	1	0	0	1	40.93781667	20.45406667
319 2003/057		1	0	0	0	0	1	0	0	1	1	0	0	1	0	0	0	0	1	1	0	0	0	0	0	0	0	0	0	1	0	0	0	0	0	0	0	0	0	0	0	0	0	0	0	1	0	1	1	40.92096667	20.4491
320 2003/058		1	0	0	0	0	0	0	0	0	0	0	0	1	0	0	0	0	0	1	0	1	0	0	0	0	0	0	0	0	0	0	0	0	0	0	0	0	0	0	0	0	0	0	0	1	0	0	1	40.92333333	20.44386667
327 2003/064		0	0	0	0	0	1	0	0	0	0	0	0	0	0	0	0	0	0	0	0	0	0	0	0	0	0	0	0	0	0	0	0	0	0	0	0	0	0	0	0	0	0	0	0	0	0	1	1	40.7891	20.90725
329 2003/066		0	0	0	0	0	0	0	0	0	0	0	0	1	0	1	0	0	0	1	0	0	0	0	0	0	0	0	0	0	0	0	0	0	0	0	0	0	0	0	0	0	0	0	0	1	0	0	1	40.52556667	20.68865
330 2003/067		0	0	0	0	0	1	0	0	0	0	0	0	0	0	1	0	0	0	1	0	0	0	0	0	0	0	0	0	0	0	0	0	0	0	0	0	0	0	0	0	0	0	0	0	1	0	0	1	40.52835	20.63436667
331 2003/068		1	0	0	0	0	1	0	0	0	1	0	0	1	0	0	0	0	0	1	0	0	0	1	0	0	0	0	0	0	0	0	0	0	0	0	0	0	0	0	0	0	0	0	0	0	0	0	1	40.5333	20.62276667
332 2003/069		1	0	0	0	0	1	0	0	0	0	0	0	0	0	1	0	0	0	1	0	0	0	0	0	0	0	0	0	0	0	0	0	0	0	0	0	0	0	0	0	0	0	0	1	0	0	1	1	40.29153333	20.6281
334 2003/071		0	0	0	0	0	0	0	0	0	0	0	0	0	1	0	0	0	0	1	0	0	0	0	0	0	0	0	0	0	0	0	0	0	0	0	0	0	0	0	0	0	0	0	0	0	0	0	1	40.23783333	20.64371667
335 2003/072		0	0	0	0	0	1	0	0	0	0	0	0	0	0	1	0	0	0	0	0	0	0	0	0	0	0	0	0	0	0	0	0	0	0	0	0	0	0	0	0	0	0	0	0	1	0	0	1	40.15266667	20.61966667
336 2003/073		0	0	0	0	0	0	0	0	0	0	0	0	1	0	1	0	0	0	1	0	0	0	1	0	0	0	0	0	0	0	1	0	0	0	0	0	0	0	0	0	0	0	0	0	0	0	0	1	40.15093333	20.59491667
339 2003/076		1	0	0	0	0	1	0	0	1	1	0	0	1	0	0	0	0	0	1	0	0	0	1	0	0	0	0	0	0	0	1	0	0	0	0	0	0	0	0	0	0	0	0	1	1	0	1	1	40.69201667	20.50015
342 2003/078		1	0	0	0	0	1	0	0	0	0	0	0	1	1	0	0	0	0	1	0	0	0	0	0	0	0	0	0	0	0	0	0	0	0	0	0	0	0	0	0	0	0	0	0	1	0	0	1	40.71361667	20.36178333
343 2003/079		1	0	0	0	0	1	0	0	1	1	0	0	0	0	1	0	0	0	1	0	0	0	1	0	0	0	0	1	0	0	1	0	0	0	0	0	0	0	0	0	0	0	0	0	1	0	0	1	40.88303333	20.24433333
345 2003/081		1	0	0	0	0	0	0	0	1	0	0	0	0	0	1	0	0	0	0	0	0	0	0	0	0	0	0	0	0	1	0	0	0	0	0	1	1	0	0	0	0	0	0	0	0	0	0	1	42.38846667	19.7363
346 2003/082		1	0	0	0	0	0	0	0	1	1	0	0	1	0	1	0	1	0	1	0	1	0	0	0	0	0	0	0	0	1	0	0	0	0	0	0	1	0	0	0	0	0	0	0	0	0	0	1	42.40228333	19.76318333
347 2003/083		0	0	0	0	0	0	0	0	0	0	0	0	0	0	1	0	0	0	0	0	0	0	0	0	1	1	0	0	0	0	0	0	0	0	0	0	1	0	0	0	0	0	0	0	0	0	0	1	42.42773333	19.76173333
348 2003/084		1	0	0	0	0	0	0	0	0	0	0	0	1	0	1	0	0	0	0	0	1	0	0	0	0	0	0	0	0	0	0	0	0	0	0	0	1	0	1	0	0	0	0	0	0	0	0	1	42.4438	19.77095
349 2003/085		0	0	0	0	0	0	0	0	0	0	0	0	0	0	0	0	0	0	1	0	0	0	0	0	0	0	0	0	0	1	0	0	0	0	0	0	0	0	0	0	0	0	0	0	0	0	0	2	42.41478333	19.75681667
350 2003/086		1	0	0	0	0	0	1	0	1	0	0	0	1	1	0	0	0	0	0	0	1	0	0	0	0	0	0	0	0	0	0	0	0	0	0	0	1	0	0	0	0	0	0	0	0	0	0	3	42.40188333	19.7493
351 2003/087		0	0	0	0	0	0	0	0	0	0	0	0	0	0	0	0	0	0	0	0	0	0	0	0	0	0	0	1	0	0	0	0	0	0	0	0	1	0	0	0	0	0	0	0	0	0	0	1	42.38951667	19.72703333
353 2003/089		1	0	0	0	0	0	0	0	0	1	0	0	0	0	0	0	0	0	0	0	0	0	0	0	0	0	0	0	0	0	0	0	0	0	0	0	1	0	0	0	0	0	0	0	0	0	0	1	42.43933333	19.53088333
354 2003/090		1	0	0	0	0	1	0	0	0	1	0	0	0	0	0	0	0	0	1	0	0	0	0	0	0	0	0	1	1	0	0	0	0	0	0	0	1	0	0	0	0	0	0	0	0	0	0	1	42.48103333	19.58266667
355 2003/091		1	0	0	0	0	0	0	0	0	1	0	0	0	0	0	0	0	0	0	0	0	0	0	0	0	0	0	1	0	0	0	0	0	0	0	0	0	0	0	0	0	0	0	0	0	0	0	1	42.5077	19.61598333
356 2003/092		1	0	1	0	0	0	0	0	1	1	0	0	0	0	1	0	0	0	1	0	0	0	0	0	0	0	0	0	0	0	0	0	0	0	0	0	1	0	0	0	0	0	0	0	0	0	0	1	42.52241667	19.6319
358 2003/094		0	0	0	0	0	1	0	0	1	0	0	0	0	0	1	0	0	0	1	0	1	0	0	0	0	0	0	0	0	0	0	0	0	0	0	1	0	0	0	0	0	0	0	0	0	0	0	1	42.5364	19.6884
359 2003/095		1	0	0	0	0	0	0	0	0	0	0	0	0	0	0	0	0	0	1	0	0	0	0	0	0	0	0	0	0	0	0	0	0	0	0	0	1	0	0	0	0	0	0	0	0	0	0	1	42.53228333	19.65086667
360	0	0	0	0	0	0	0	0	0	0	0	0	0	1	0	0	0	0	0	0	0	0	0	0	0	0	0	0	0	1	0	0	0	0	0	0	0	0	0	0	0	0	0	0	0	0	0	1	1	42.6306	19.0085
361	0	1	0	0	0	0	0	0	0	0	0	0	0	1	0	0	0	0	0	0	0	0	0	0	0	0	0	0	0	0	0	0	0	0	0	0	0	0	0	0	0	0	0	0	0	0	0	1	1	42.4203	18.8239
362	0	1	0	0	0	0	0	0	0	0	0	0	0	1	0	0	0	0	0	1	0	0	0	0	0	0	0	0	0	0	0	1	0	0	0	0	0	0	0	0	0	0	0	0	0	0	0	0	1	42.5208	18.6908
363 2004/003		0	0	0	0	0	1	0	0	1	0	0	0	1	0	0	0	0	0	0	0	0	0	0	0	0	0	0	0	0	0	0	0	0	0	0	0	0	0	0	0	0	0	0	0	0	0	0	1	41.8265	21.6865
364 2004/004		0	0	0	0	0	1	0	0	0	0	0	0	0	0	0	0	0	0	0	0	0	0	0	0	0	0	0	0	0	1	0	0	0	1	0	0	0	0	0	0	0	0	0	0	0	0	0	1	41.704	21.7885
365 2004/005		0	0	0	0	0	0	0	0	0	0	0	0	1	0	0	0	0	0	1	0	0	0	0	0	0	0	0	0	0	1	0	0	0	1	0	0	0	0	0	0	0	0	0	0	0	0	0	1	41.70633333	21.78133333
367 2004/007		0	0	0	0	0	1	0	0	1	1	0	0	0	0	0	1	0	0	0	0	1	0	1	0	0	0	0	0	0	1	0	0	0	0	0	1	0	0	0	0	0	0	0	0	0	0	1	1	41.681	21.43033333
368 2004/009		1	0	1	0	0	1	0	0	0	1	0	0	1	0	1	1	0	0	1	0	0	0	0	0	0	1	0	0	0	0	0	0	0	1	0	0	0	0	0	0	0	0	0	0	0	0	0	1	41.43683333	21.86716667
369 2004/010		1	0	0	0	0	1	0	0	1	1	0	0	1	0	1	1	0	0	1	0	0	0	0	0	0	0	0	0	0	1	0	0	0	1	0	0	0	0	0	0	0	0	0	0	0	0	0	1	41.43666667	21.81666667
370 2004/011		0	0	0	0	0	1	0	0	0	0	0	0	0	0	1	0	0	0	0	0	0	0	0	0	0	0	0	0	0	1	0	0	0	0	0	0	0	0	0	0	1	0	0	0	0	0	0	1	41.3655	21.7165
373 2004/014		0	0	0	0	0	1	0	0	1	0	0	0	0	1	1	1	0	0	1	0	0	0	1	0	0	1	0	0	0	1	0	0	0	0	0	0	0	0	0	0	0	0	0	0	0	0	0	1	40.935	20.93666667
374 2004/015		0	0	0	0	0	1	0	0	0	0	0	0	0	0	1	0	0	0	0	0	0	0	0	0	0	0	0	0	0	0	0	0	0	0	0	0	0	0	0	0	0	0	0	0	0	0	0	1	40.96883333	20.91016667
376 2004/017		0	0	0	0	0	1	0	0	0	0	0	0	0	1	1	0	0	0	0	0	0	0	0	0	0	0	0	0	0	1	0	0	0	0	0	0	0	0	0	0	0	0	0	0	1	0	0	2	40.95583333	20.82033333
377 2004/018		0	0	0	0	0	1	0	0	1	0	0	0	1	0	0	1	0	0	1	0	0	0	0	0	0	0	0	0	0	0	0	0	0	0	0	0	0	0	0	0	0	0	0	0	1	1	1	1	40.954	20.8125
379 2004/020		0	0	0	0	0	0	0	0	1	1	0	0	0	0	1	1	0	0	0	0	0	0	0	0	0	0	0	0	0	0	0	0	0	0	0	0	0	0	0	0	0	0	0	0	0	1	0	3	40.97116667	20.82066667
380 2004/021		0	0	0	0	0	1	0	0	1	1	0	0	1	0	0	1	0	0	0	0	0	0	0	0	0	0	0	0	0	1	0	0	0	0	0	0	0	0	0	0	0	0	0	0	0	0	1	1	40.96466667	20.81466667
388 2004/028		0	0	0	0	0	1	0	0	1	1	0	0	1	1	1	0	0	0	1	0	0	0	1	0	0	0	0	0	0	1	0	0	0	0	0	0	0	0	0	0	0	0	0	0	1	0	2	1	40.9656	20.78585
391 2004/031		1	0	0	0	0	1	0	0	0	0	0	0	0	0	1	0	0	0	0	0	1	0	1	0	0	0	0	0	0	1	0	0	0	0	0	0	0	0	0	0	0	0	0	0	1	0	0	1	41.074	20.803
392 2004/032		0	0	0	0	0	1	0	0	0	0	0	0	0	1	1	0	0	0	0	0	0	0	1	0	0	0	0	0	0	0	0	0	0	0	0	0	0	0	0	0	0	0	0	0	1	0	1	1	41.103	20.81433333
393 2004/033		0	0	0	0	0	1	0	0	0	0	0	0	1	0	0	0	0	0	0	0	0	0	1	0	0	0	0	0	0	1	0	0	0	0	0	0	0	0	0	0	0	0	0	0	1	0	0	1	41.11133333	20.7905
394 2004/034		0	0	0	0	0	1	0	0	1	1	0	0	1	0	1	0	0	0	1	0	1	0	1	0	1	1	0	0	0	1	0	0	0	0	0	0	0	0	0	0	0	0	0	0	0	0	1	1	41.19783333	20.91733333
396 2004/036		0	0	0	0	0	1	0	0	0	0	0	0	1	0	0	0	0	0	0	0	0	0	0	0	0	0	0	0	0	1	0	0	0	0	0	0	0	0	0	0	0	0	0	0	1	0	0	1	41.13883333	20.7625
397 2004/037		0	0	0	0	0	0	0	0	0	0	0	0	0	0	0	0	0	0	0	0	0	0	0	0	0	0	0	0	0	0	0	0	0	0	0	0	0	0	0	0	0	0	0	0	1	0	0	1	41.18283333	20.63233333
399 2004/038		0	0	0	0	0	1	0	0	1	0	0	0	0	0	0	0	0	0	1	0	0	0	0	0	0	0	0	0	0	0	0	0	0	0	0	0	0	0	0	0	0	0	0	0	1	0	1	1	41.14933333	20.64933333
401 2004/040		0	0	0	0	0	1	0	0	1	0	0	0	0	0	0	0	0	0	0	0	0	0	0	0	0	0	0	0	0	0	0	0	0	0	0	0	0	0	0	0	0	0	0	0	1	0	1	1	41.3355	20.6345
406 2004/045		1	0	0	0	0	1	0	0	1	0	0	0	1	0	1	0	0	0	0	0	0	0	0	0	0	0	0	0	1	0	0	0	0	0	0	0	0	0	0	0	0	0	0	1	1	1	0	3	41.621	20.60916667
418 2004/057		0	0	0	0	0	0	0	0	1	1	0	0	1	0	1	1	0	0	0	0	1	0	1	0	0	0	0	0	0	1	0	0	0	0	0	1	0	0	0	0	0	0	0	0	0	1	0	1	42.02083333	20.902
419 2004/058		0	0	0	0	0	1	0	0	0	0	0	0	0	0	1	0	0	0	0	0	0	0	0	0	0	0	0	0	0	0	0	0	0	0	0	0	0	0	0	0	0	0	0	0	0	0	0	2	42.01566667	20.8855
420 2004/059		0	0	0	0	0	0	0	0	1	0	0	0	0	0	0	0	0	0	0	0	0	0	0	0	0	0	0	0	0	0	0	0	0	0	0	1	0	0	0	0	0	0	0	0	0	0	0	3	42.01133333	20.90233333
421 2004/060		1	0	0	0	0	1	0	0	1	0	0	0	1	0	1	1	0	0	0	0	0	0	1	0	0	0	0	0	0	1	0	0	0	0	0	1	0	0	0	0	0	0	0	0	0	0	1	1	41.961	21.29433333
422 2004/061		0	0	0	0	0	1	0	0	1	0	1	0	1	0	0	0	0	0	1	0	1	0	0	1	0	0	0	0	0	0	0	0	0	0	0	0	0	0	0	0	0	0	0	0	0	0	0	1	43.85766667	21.6145
424	0	0	0	0	0	0	1	0	0	0	0	0	0	0	1	1	0	0	0	1	0	0	0	0	0	0	0	0	0	0	0	0	0	0	0	0	0	0	0	0	0	0	0	0	1	1	0	1	1	40.69865	20.096633
425 2004/075		1	0	0	0	0	0	0	0	1	1	0	0	1	0	0	1	0	0	1	1	0	0	1	0	0	0	0	0	0	1	1	0	0	0	0	0	0	0	0	0	0	0	0	0	0	0	1	1	40.55355	20.27832
426 2004/076		1	0	0	0	0	1	0	0	0	0	0	0	1	0	0	0	0	0	1	0	0	0	0	0	0	0	0	0	0	0	1	0	0	0	0	0	0	0	0	0	0	0	0	0	1	0	0	1	40.55387	20.27148
427 2004/077		1	0	0	0	0	1	0	0	0	0	0	0	0	0	0	0	0	0	0	0	0	0	0	0	0	0	0	0	0	0	0	0	0	0	0	0	0	0	0	0	0	0	0	0	0	0	0	2	40.56024	20.26453
428 2004/078		0	0	0	0	0	1	0	0	0	0	0	0	0	0	0	0	0	0	1	0	0	0	0	0	0	0	0	0	0	0	0	0	0	0	0	0	0	0	0	0	0	0	0	0	0	0	0	1	40.56464	20.26193
429 2004/079		1	0	0	0	0	1	0	0	1	1	0	0	1	0	1	1	0	0	0	0	1	0	1	0	0	0	0	0	0	0	0	0	0	0	0	0	0	0	0	0	0	0	0	0	1	0	1	1	40.56856	20.25854
430 2004/080		0	0	0	0	0	1	0	0	0	0	0	0	0	0	1	0	0	0	0	0	1	0	0	0	0	0	0	0	0	0	0	0	0	0	0	0	0	0	0	0	0	0	0	0	1	0	1	1	40.57754	20.25825
431 2004/081		0	0	0	0	0	0	0	0	0	0	0	0	1	0	1	0	0	0	0	0	0	0	0	0	0	0	0	0	0	0	0	0	0	0	0	0	0	0	0	0	0	0	0	0	1	0	0	1	40.58657	20.24382
432 2004/082		0	0	0	0	0	0	0	0	0	0	0	0	1	0	0	0	0	0	0	0	0	0	0	0	0	0	0	0	0	0	0	0	0	0	0	0	0	0	0	0	0	0	0	0	0	0	1	1	40.58961	20.24137
433 2004/083		0	0	0	0	0	0	0	0	0	0	0	0	1	0	1	1	0	0	0	0	0	0	0	0	0	0	0	0	0	0	0	0	0	0	0	0	0	0	0	0	0	0	0	0	0	0	1	1	40.59128	20.23847
434 2004/084		1	0	0	0	0	0	0	0	1	0	0	0	1	0	0	0	0	0	0	0	0	0	1	0	0	0	0	1	0	0	0	0	0	0	0	0	0	0	0	0	0	0	0	0	1	0	1	1	40.59518	20.22139
435 2004/085		0	0	0	0	0	1	0	0	0	0	0	0	0	0	0	0	0	0	0	0	0	0	0	0	0	0	0	0	0	0	0	0	0	0	0	0	0	0	0	0	0	0	0	0	0	0	0	2	40.59848	20.21548
436 2004/086		0	0	0	0	0	0	0	0	0	0	0	0	1	0	0	0	0	0	0	0	0	0	0	0	0	0	0	0	0	0	0	0	0	0	0	0	0	0	0	0	0	0	0	0	0	0	0	1	40.6248	20.1742
437 2004/087		0	0	0	0	0	0	0	0	0	0	0	0	0	0	0	0	0	0	0	0	0	0	0	0	0	0	0	0	0	0	0	0	0	0	0	0	0	0	0	0	0	0	0	0	0	0	1	1	40.63063	20.16619
439 2004/089		1	0	0	0	0	0	0	0	0	0	0	0	1	0	1	0	0	0	0	0	1	0	1	0	0	0	0	0	0	0	0	0	0	0	0	0	0	0	0	0	0	0	0	0	0	0	1	2	40.64401	20.15902
440 2004/090		1	0	0	0	0	1	0	0	0	0	0	0	0	0	0	0	0	0	0	0	1	0	0	0	0	0	0	0	0	0	0	0	0	0	0	0	0	0	0	0	0	0	0	0	0	0	0	1	40.65156	20.15729
441 2004/091		1	0	0	0	0	1	0	0	0	0	0	0	1	1	0	0	0	0	0	0	0	0	0	0	0	0	0	0	0	0	0	0	0	0	0	0	0	0	0	0	0	0	0	0	1	0	0	1	40.65334	20.16117
442 2004/092		0	0	0	0	0	1	0	0	0	0	0	0	1	0	0	0	0	0	1	0	0	0	0	0	0	0	0	0	0	0	0	0	0	0	0	0	0	0	0	0	0	0	0	0	0	0	0	1	40.6745	20.15773
443 2004/093		1	0	0	0	0	1	0	0	0	0	0	0	1	0	0	0	0	0	0	0	1	0	0	0	0	0	0	0	0	0	0	0	0	0	0	0	0	0	0	0	0	0	0	0	0	0	1	1	40.68722	20.15067
444 2004/094		1	0	0	0	0	1	0	0	0	0	0	0	1	0	0	0	0	0	0	0	0	0	0	0	0	0	0	1	0	0	0	0	0	0	0	0	0	0	0	0	0	0	0	0	0	0	1	1	40.68886	20.14611
445 2004/095		0	0	0	0	0	1	0	0	0	0	0	0	0	0	1	0	0	0	0	0	1	0	0	0	0	0	0	0	0	0	0	0	0	0	0	0	0	0	0	0	0	0	0	0	0	0	0	2	40.69866	20.14496
446 2004/096		0	0	0	0	0	1	0	0	1	0	0	0	0	0	0	1	0	0	0	0	0	0	0	0	0	0	0	0	1	0	0	0	0	0	0	0	0	0	0	0	0	0	0	0	1	1	1	3	40.68797	20.13987
447 2004/097		0	0	0	0	0	0	0	0	0	0	0	0	0	1	0	0	0	0	0	0	0	0	0	0	0	0	0	0	0	0	0	0	0	0	0	0	0	0	0	0	0	0	0	0	0	0	1	3	40.68832	20.1341
448 2004/098		0	0	0	0	0	1	0	0	0	0	0	0	0	0	0	0	0	0	1	0	0	0	0	0	0	0	0	0	0	0	0	0	0	0	0	0	0	0	0	0	0	0	0	0	0	0	0	1	40.68779	20.12874
450 2004/100		1	0	0	0	0	0	0	0	0	0	0	0	1	0	1	0	0	0	1	0	0	0	0	0	0	0	0	1	1	0	0	0	0	0	0	0	0	0	0	0	0	0	0	0	1	0	1	1	40.67884	20.11085
451 2004/101		1	0	0	0	0	0	0	0	0	0	0	0	0	0	0	0	0	0	0	0	0	0	0	0	0	0	0	0	0	0	0	0	0	0	0	0	0	0	0	0	0	0	0	1	0	0	0	1	40.21584	19.60634
452 2004/102		1	0	0	0	0	1	0	0	0	0	0	0	1	0	0	0	0	0	0	0	0	0	0	0	0	0	0	0	0	0	0	0	0	0	0	0	0	0	0	0	0	0	0	1	0	0	0	2	40.21383	19.61098
453 2004/103		0	0	0	0	0	0	0	0	0	0	0	0	0	0	0	0	0	0	0	0	0	0	0	0	0	0	0	0	0	0	0	0	0	0	0	0	0	0	0	0	0	0	0	0	0	0	0	2	40.20966	19.61786
454 2004/104		1	0	0	0	0	1	0	0	0	0	0	0	0	0	0	0	0	0	0	0	0	0	0	0	0	0	0	0	0	0	0	0	0	0	0	0	0	0	0	0	0	0	0	0	0	0	0	1	40.19852	19.59163
456 2004/106		1	0	0	0	0	0	0	0	0	0	0	0	1	0	0	0	0	0	0	0	0	0	0	0	0	0	0	0	0	0	0	0	0	0	0	0	1	0	0	0	0	0	0	0	0	0	0	1	42.66346	19.37159
459 2004/109		1	0	0	0	0	0	0	0	0	0	0	0	0	0	1	0	0	0	1	0	0	0	0	0	0	0	0	1	0	0	0	0	0	0	0	0	0	0	0	0	0	1	0	0	1	0	0	1	41.5065	19.92183333
466 2004/115		0	0	0	0	0	1	0	0	0	0	0	0	0	1	0	0	0	0	0	0	0	0	0	0	0	0	0	0	0	0	0	0	0	0	0	0	0	0	0	0	0	0	0	0	0	0	1	1	41.47083333	20.07783333
472 2004/121		1	0	0	0	0	1	0	0	0	0	0	0	1	0	1	0	0	0	0	0	1	0	0	0	0	0	0	1	0	1	0	0	0	0	0	0	0	0	0	0	0	1	0	0	0	0	1	1	41.35147222	20.11627778
475 2004/124		0	0	0	0	0	0	0	0	0	0	0	0	1	0	1	0	0	0	0	0	0	0	0	0	0	0	0	0	0	0	0	0	0	0	0	0	0	0	0	0	0	0	0	0	1	0	0	1	40.516	20.24263889
476 2004/125		1	0	1	0	0	0	0	0	1	1	0	0	0	1	1	0	0	0	1	0	0	0	0	0	0	0	0	1	0	0	1	0	0	0	0	0	0	0	0	0	0	0	0	1	1	0	1	1	40.5235	20.25311111
480 2004/128		1	0	0	0	0	0	0	0	1	0	0	0	1	1	1	0	0	0	1	0	0	0	0	0	0	0	0	0	0	0	0	0	0	0	0	0	0	0	0	0	0	0	0	1	1	0	1	1	40.53561111	19.79638889
481 2004/130		1	0	0	0	0	0	0	0	0	0	0	0	1	0	1	0	0	0	1	0	0	0	1	0	0	0	0	0	0	0	0	0	0	0	0	0	0	0	0	0	0	0	0	1	0	0	0	1	40.24916667	19.99397222
482 2004/131		0	0	0	0	0	0	0	0	0	0	0	0	0	0	0	0	0	0	0	0	0	0	0	0	0	0	0	0	0	0	0	0	0	0	0	0	0	0	0	0	0	0	0	0	0	0	0	2	40.24077778	19.96152778
483 2004/132		1	0	0	0	0	0	0	0	1	1	0	0	1	0	1	1	0	0	0	0	0	0	1	0	0	0	0	0	0	0	0	0	0	0	0	0	0	0	0	0	0	0	0	1	1	0	0	1	40.21022222	19.96141667
484 2004/133		1	0	1	0	0	1	0	0	1	1	0	0	0	0	1	0	0	0	1	0	0	0	1	0	0	0	0	0	0	0	0	0	0	0	0	0	0	0	0	0	0	0	0	1	0	0	0	2	40.2135	19.93541667
485 2004/134		1	0	0	0	0	0	0	0	1	0	0	0	0	0	1	0	0	0	1	0	0	0	1	0	0	0	0	0	0	0	0	0	0	0	0	0	0	0	0	0	0	0	0	1	0	0	0	1	40.21297222	19.93052778
486 2004/135		1	0	0	0	0	0	0	0	0	0	0	0	0	0	0	0	0	0	1	0	0	0	0	0	0	0	0	0	0	0	0	0	0	0	0	0	0	0	0	0	0	0	0	0	0	0	0	1	40.26347222	20.00708333
487 2004/136		1	0	0	0	0	0	0	0	0	0	0	0	0	1	1	0	0	0	0	0	0	0	0	0	0	0	0	1	0	0	0	0	0	0	0	0	0	0	0	0	0	0	0	1	0	0	0	1	40.29380556	20.00875
489 2004/138		1	0	0	0	0	0	0	0	1	0	0	0	0	1	0	0	0	0	1	0	0	0	0	0	0	0	0	1	0	0	0	1	0	0	0	0	0	0	0	0	0	0	0	0	0	0	1	1	40.08827778	20.27166667
490 2004/139		1	0	0	0	0	0	0	0	1	0	0	0	1	0	0	0	0	0	0	0	0	0	0	0	0	0	0	1	0	0	0	1	0	0	0	0	0	0	0	0	0	0	0	0	0	0	1	1	40.09147222	20.29219444
491 2004/140		1	0	0	0	0	0	0	0	0	0	0	0	0	0	0	0	0	0	0	0	0	0	0	0	0	0	0	0	0	0	0	1	0	0	0	0	0	0	0	0	0	0	0	0	1	0	0	1	40.11641667	20.32941667
492 2004/141		1	0	0	0	0	0	0	0	0	0	0	0	0	0	0	0	0	0	1	0	0	0	0	0	0	0	0	0	0	0	0	1	0	0	0	0	0	0	0	0	0	0	0	0	1	0	0	1	40.17011111	20.31511111
493 2004/142		1	0	0	0	0	1	0	0	0	0	0	0	0	0	0	0	0	0	1	0	0	0	1	0	0	0	0	0	0	0	0	1	0	0	0	0	0	0	0	0	0	0	0	0	1	0	0	1	40.18558333	20.33236111
495 2004/144		1	0	0	0	0	0	0	0	1	1	0	0	0	0	1	0	0	0	0	0	0	0	0	0	0	0	0	1	0	0	0	0	0	0	0	0	0	0	0	0	0	0	0	1	1	0	0	1	40.07444444	20.14063889
496 2004/145		1	0	0	0	0	0	0	0	0	0	0	0	1	0	1	0	0	0	0	0	0	0	0	0	0	0	0	0	0	0	0	0	0	0	0	0	0	0	0	0	0	0	0	1	1	0	0	3	39.92461111	20.1925
499 2004/147		1	0	0	0	0	0	0	0	0	0	0	1	1	1	1	0	0	0	1	0	0	0	0	0	0	0	0	1	0	0	0	0	0	0	0	0	0	0	0	0	0	0	0	1	0	0	0	1	40.05277778	19.81280556
500 2004/148		1	0	0	0	0	0	0	0	0	0	0	0	0	0	0	0	0	0	1	0	0	0	0	0	0	0	0	1	0	0	0	1	0	0	0	0	0	0	0	0	0	0	0	1	0	0	0	1	40.15577778	19.63936111
501 2004/149		0	0	0	0	0	0	0	0	0	0	0	0	0	0	0	0	0	0	0	0	0	0	0	0	0	0	0	1	0	1	0	0	0	0	0	0	1	0	0	0	0	0	0	0	0	0	0	1	42.74833333	19.40666667
502 2004/150		0	0	0	0	0	0	0	0	0	0	0	0	0	0	1	0	0	0	0	0	0	0	0	0	0	0	0	0	0	0	0	0	0	0	0	0	0	0	0	0	0	0	0	0	0	0	0	1	43.29027778	19.714
506 2005/B02		1	0	0	0	0	0	0	0	0	0	0	0	0	0	0	0	0	0	1	0	0	0	0	0	0	0	0	0	0	0	0	0	0	0	0	0	0	0	0	0	0	0	0	0	0	0	0	1	40.26658	20.00596
507 2005/B03		1	0	0	0	0	1	0	0	0	0	0	0	0	0	0	0	0	0	0	0	0	0	0	0	0	0	0	0	0	0	0	0	0	0	0	0	0	0	0	0	0	0	0	0	0	0	0	1	40.26201	19.98091
508 2005/B04		1	0	0	0	0	1	0	0	1	0	0	0	0	0	0	0	0	0	0	0	0	0	0	0	0	0	0	0	0	0	0	0	0	0	0	0	0	0	0	0	0	0	0	0	0	0	0	1	40.26385	19.9704
509 2005/B05		1	0	0	0	0	0	0	0	1	0	0	0	1	0	0	0	0	0	0	0	0	0	0	0	0	0	0	0	0	0	0	0	0	0	0	0	0	0	0	0	0	0	0	0	0	0	0	1	40.26494	19.95902
510 2005/B06		1	0	0	0	0	0	0	0	0	0	0	0	0	0	0	0	0	0	0	0	0	0	0	0	0	0	0	0	0	0	0	0	0	0	0	0	0	0	0	0	0	0	0	0	0	0	0	1	40.27073	19.92616
511 2005/B07		1	0	0	0	0	0	0	0	0	0	0	0	0	0	0	0	0	0	0	0	0	0	1	0	0	0	0	0	0	0	0	0	0	0	0	0	0	0	0	0	0	0	0	0	1	0	0	1	40.27602	19.9013
512 2005/B08		0	0	0	0	0	1	0	0	0	0	0	0	0	0	1	0	0	0	1	0	0	0	0	0	0	0	0	0	0	0	0	0	0	0	0	0	0	0	0	0	0	0	0	0	0	0	0	1	40.27822	19.87347
513 2005/B09		1	0	0	0	0	0	0	0	0	0	0	0	0	0	1	0	0	0	0	0	0	0	0	0	0	0	0	0	0	0	0	1	0	0	0	0	0	0	0	0	0	0	0	0	0	0	0	1	40.2843	19.85666
514 2005/B10		1	0	0	0	0	0	0	0	1	0	0	0	1	0	1	0	0	0	0	0	0	0	0	0	0	0	0	0	0	0	0	0	0	0	0	0	0	0	0	0	0	0	0	0	0	0	0	1	40.28706	19.8528
515 2005/B10B		0	0	0	0	0	0	0	0	0	0	0	0	0	0	0	0	0	0	0	0	0	0	1	0	0	0	0	0	0	0	0	0	0	0	0	0	0	0	0	0	0	0	0	0	0	0	0	1	40.28698	19.8614

517		0	1	0	0	0	0	0	0	0	0	0	0	0	0	0	0	0	0	0	0	0	0	0	0	0	0	0	0	0	0	0	0	0	0	0	0	0	0	0	0	1	0	0	0	0	0	0	0	0	0	1	42.44313	19.76374
518		0	0	0	0	0	0	0	0	0	0	0	0	0	0	0	0	0	0	0	0	0	0	0	0	0	0	0	0	0	0	0	0	0	0	0	0	0	0	0	1	0	0	0	0	0	0	0	0	0	0	1	42.40623	19.80768
520	2005/O02		0	0	0	0	0	0	0	0	0	0	0	0	0	0	0	0	0	0	1	0	0	0	0	0	0	0	0	0	0	0	0	0	0	0	0	0	0	0	0	0	0	0	0	0	0	0	0	0	0	1	40.51995	20.41771
522	2005/O04		0	0	0	0	0	1	0	0	0	0	0	0	0	0	0	0	0	0	0	0	0	0	0	0	0	0	0	0	0	0	0	0	0	0	0	0	0	0	0	0	0	0	0	0	0	0	0	0	0	1	40.52602	20.41528
524	2005/O13		0	0	0	0	0	0	0	0	0	1	0	0	0	0	1	0	0	0	0	0	0	0	1	0	1	0	0	0	0	0	0	0	0	0	0	0	0	0	0	0	0	0	0	0	0	0	0	0	0	1	40.57649	20.44733
525	2005/O15		1	0	0	0	0	0	0	0	0	0	0	0	0	0	0	0	0	0	1	0	0	0	1	0	1	0	0	0	0	0	0	0	1	0	0	0	0	0	0	0	0	0	0	0	0	0	0	0	0	1	40.56065	20.39377
528	2005/043		0	0	0	0	0	1	0	0	0	0	0	0	1	0	1	0	0	0	1	0	0	0	0	0	0	0	0	0	0	0	0	0	0	0	0	0	0	0	1	0	0	0	0	0	0	0	0	0	0	1	42.55023333	19.82476667
529	2005/044		0	0	0	0	0	1	0	0	0	0	0	0	1	0	0	0	0	0	1	0	0	0	0	0	0	0	0	0	0	0	0	0	0	0	0	0	0	1	0	0	0	0	0	0	0	0	0	0	0	1	42.57818333	19.81493333
532	2005/047		1	0	0	0	0	1	0	0	1	0	0	0	1	1	1	0	1	0	1	0	1	0	1	0	1	0	0	0	0	0	1	1	0	0	0	0	0	1	0	0	1	0	0	0	0	0	0	0	0	1	42.57208333	19.73991667
534	2005/049		1	0	0	0	0	1	0	0	1	1	0	0	1	0	1	1	0	0	1	0	0	0	0	0	0	0	0	0	0	0	0	0	0	0	0	0	0	1	1	0	1	0	0	0	0	0	0	0	0	1	42.51563333	19.73713333
540	2005/054		1	0	0	0	0	1	0	0	1	0	0	0	1	0	1	1	0	0	1	0	0	0	0	0	0	0	1	0	0	0	0	1	0	0	0	0	0	1	0	0	1	0	0	0	0	0	0	0	0	1	42.68468333	20.05356667
541	2005/055		1	0	0	0	0	1	0	0	1	0	0	0	1	0	0	0	0	0	1	0	1	0	0	0	0	0	0	0	0	0	0	0	0	0	0	0	0	0	0	0	0	0	0	0	0	0	0	0	0	1	42.7723	20.27901667
544	2005/058		0	0	0	0	0	1	0	0	0	0	0	0	1	0	0	0	0	0	0	0	0	0	0	0	0	0	0	0	0	0	0	0	0	0	0	0	0	0	1	0	0	0	0	0	0	0	0	0	0	1	42.38841667	20.0729
545	2005/059		1	0	0	0	0	1	0	0	0	0	0	0	1	0	0	0	0	0	1	0	0	0	0	0	0	0	0	0	0	0	0	1	0	0	0	0	0	0	1	0	0	0	0	0	0	0	0	0	0	1	42.41638333	20.01271667
546	2005/060		0	0	0	0	0	1	0	0	1	1	0	0	1	0	1	0	0	0	1	0	0	0	1	0	1	0	0	0	0	0	0	0	0	0	0	0	0	0	1	0	0	0	0	0	0	0	0	0	1	1	42.4364	19.98465
547	2005/061		0	0	0	0	0	0	0	0	0	0	0	0	1	0	0	0	0	0	0	0	0	0	0	0	0	0	0	0	0	0	0	0	0	0	0	0	0	0	0	0	0	0	0	0	0	0	0	0	0	1	42.41033333	19.82276667
548	2005/062		0	0	0	0	0	0	0	0	0	0	0	0	1	0	0	0	0	0	0	0	0	0	0	0	0	0	0	0	0	0	0	0	0	0	0	0	0	0	1	0	0	0	0	0	0	0	0	0	0	1	42.41135	19.81475
549	2005/063		1	0	0	0	0	0	0	0	0	0	0	0	1	0	0	0	0	0	0	0	0	0	0	0	0	0	0	0	0	0	0	0	0	0	0	0	0	0	1	0	0	0	0	0	0	0	0	0	1	1	42.40678333	19.8122
552	2005/065		1	0	0	0	0	0	0	0	0	1	0	0	0	0	1	0	0	0	1	0	0	0	1	0	1	0	0	0	0	1	0	0	0	0	0	0	0	0	1	0	0	0	0	0	0	0	0	0	0	1	42.29878333	20.02885
553	2005/066		1	0	0	0	0	0	0	0	1	1	0	0	1	1	1	0	0	1	1	0	0	0	0	0	0	0	0	0	0	1	0	0	0	0	0	0	0	0	1	0	0	0	0	0	0	0	1	0	0	1	42.28676667	20.02145
554	2005/067		1	0	0	0	0	0	0	0	1	1	0	0	1	0	1	0	0	0	1	0	0	0	1	0	1	0	0	0	0	1	0	0	1	0	0	0	0	0	1	0	0	0	0	0	0	0	0	0	1	1	42.26306667	19.99
555	2005/068		0	0	0	0	0	0	0	0	0	0	0	0	1	0	0	0	0	0	0	0	0	0	0	0	0	0	0	0	0	0	0	0	0	0	0	0	0	0	0	0	0	0	0	0	0	0	0	0	0	1	42.2678	19.94943333
557	2005/070		1	0	0	0	0	1	0	0	0	1	0	0	1	0	1	0	0	0	1	0	0	0	1	0	1	0	0	0	0	0	0	0	1	0	0	0	0	0	0	0	0	0	0	0	0	0	1	0	0	1	42.22025	20.09765
561	2005/074		0	0	0	0	0	1	0	0	1	0	0	0	1	1	1	0	0	0	0	0	0	0	0	0	0	0	0	0	0	0	0	0	0	0	0	0	0	1	0	0	0	0	0	0	0	0	0	0	0	1	41.94721667	20.27955
562	2005/075		0	0	0	0	0	1	0	0	0	0	0	0	1	1	1	0	0	0	1	0	0	0	0	0	0	0	0	0	0	1	0	0	0	0	0	0	0	0	0	0	0	0	0	0	0	0	0	0	1	1	41.92416667	20.2813
563	2005/076		1	0	0	0	0	0	0	0	0	1	0	0	1	0	1	0	0	1	1	0	1	0	1	0	1	0	0	1	0	0	0	0	0	0	0	0	0	1	0	0	0	0	0	0	0	0	1	1	0	1	41.9163	20.2526
564	2005/077		1	0	0	0	0	0	0	0	1	1	0	0	1	1	1	1	1	0	1	0	1	0	1	0	1	0	0	0	0	1	0	0	0	0	0	0	0	0	0	0	0	0	0	0	0	0	1	0	1	1	41.91881667	20.26928333
568	2005/081		0	0	0	0	0	0	0	0	0	0	0	0	0	0	0	0	0	0	0	0	0	0	0	0	0	0	0	0	0	0	0	0	0	0	0	0	0	0	0	0	0	0	0	0	0	0	0	0	0	1	41.94046667	20.37601667
569	2005/082		0	0	0	0	0	1	0	0	1	1	0	0	1	0	1	0	0	0	1	0	0	0	0	0	0	0	0	0	0	1	0	0	0	0	0	0	0	0	0	0	0	0	0	0	0	0	0	0	1	1	41.93515	20.3632
570	2005/083		0	0	0	0	0	1	0	0	0	0	0	0	0	0	1	0	0	0	1	0	0	0	1	0	1	0	0	0	0	0	0	0	0	0	0	0	0	0	0	0	0	0	0	0	0	0	0	0	0	1	41.92371667	20.35395
571	2005/084		1	0	0	0	0	1	0	0	0	0	0	0	1	1	1	0	0	0	1	0	0	0	0	0	0	0	0	0	0	1	0	0	0	0	0	0	0	0	0	0	0	0	0	0	0	0	0	0	1	1	41.94231667	20.35933333
572	2005/085		0	0	0	0	0	0	0	0	0	0	0	0	1	0	1	0	0	0	1	0	0	0	0	0	0	0	0	0	0	0	0	0	0	0	0	0	0	0	0	0	0	0	0	0	0	0	1	0	0	1	41.90813333	20.35825
573	2005/086		1	0	0	0	0	1	0	0	0	0	0	0	1	1	1	0	0	0	0	0	0	0	0	0	0	0	0	0	0	1	0	0	0	0	0	0	0	0	0	0	0	0	0	0	0	1	0	0	1	1	41.89078333	20.337
574	2005/087		1	0	0	0	0	1	0	0	1	0	0	0	1	1	1	0	0	0	1	0	0	0	0	0	0	0	0	0	0	1	0	0	0	0	0	0	0	0	0	0	0	0	0	0	0	0	0	0	1	1	41.88803333	20.31651667
576	2005/089		1	0	0	0	0	1	0	0	0	1	0	0	1	1	1	0	0	0	0	0	0	0	0	0	0	0	0	0	0	1	0	0	0	0	0	0	0	0	0	0	0	0	0	0	0	0	1	0	1	1	41.73908333	20.29681667
578	2005/090A		1	0	0	0	0	1	0	0	0	0	0	0	1	0	1	0	0	1	1	0	0	0	0	0	0	0	0	0	0	0	0	0	0	0	0	0	0	0	0	0	0	0	0	0	0	0	1	0	1	1	41.75248333	20.24553333
579	2005/090B		0	0	0	0	0	1	0	0	0	1	0	0	1	1	0	0	0	0	1	0	0	0	0	0	0	0	0	0	0	1	0	0	0	0	0	0	0	0	0	0	0	0	0	0	0	0	0	0	1	1	41.7506	20.26256667
580	2005/091		0	0	0	0	0	0	0	0	0	0	0	0	0	0	0	0	0	0	1	0	0	0	0	0	0	0	0	0	0	0	0	0	0	0	0	0	0	0	0	0	0	0	0	0	0	0	1	0	0	1	41.78555	20.28701667
581	2005/092		1	0	0	0	0	1	0	0	0	0	0	0	0	0	1	0	0	0	1	0	0	0	0	0	0	0	0	0	0	0	0	0	0	0	0	0	0	0	0	0	0	0	0	0	0	0	1	0	0	1	41.7994	20.28061667
582	2005/093		1	0	0	0	0	1	0	0	1	1	0	0	1	1	1	1	0	0	1	0	1	0	0	0	0	0	0	0	0	1	0	0	0	0	0	0	0	0	0	0	0	0	0	0	0	1	1	1	1	3	41.81486667	20.2775
586	2005/097		1	0	0	0	0	0	0	0	0	0	0	0	0	0	0	0	0	0	1	0	0	0	0	0	0	0	0	0	0	1	0	0	0	0	0	0	0	0	0	0	0	0	0	0	0	0	0	0	1	1	41.61865	20.28953333
587	2005/098		0	0	0	0	0	1	0	0	1	0	0	0	1	0	1	0	0	0	1	0	0	0	1	0	1	0	0	0	0	0	0	1	0	0	0	0	0	1	0	0	1	0	0	0	0	0	0	0	0	1	42.73731667	20.3068
588	2005/099		0	0	0	0	0	0	0	0	0	0	0	0	1	0	1	0	0	0	0	0	0	0	0	0	0	0	0	0	0	0	1	0	0	0	0	0	0	0	0	0	0	0	0	0	0	0	0	0	0	1	42.85305	20.20396667
589	2005/100		1	0	0	0	0	0	0	0	0	0	0	0	1	0	1	0	0	0	1	0	0	0	1	0	1	0	0	0	0	0	0	1	0	0	0	0	0	0	0	0	1	0	0	0	0	0	0	0	0	1	42.90683333	20.33436667
590	2005/101		0	0	0	0	1	0	0	0	1	0	0	0	1	0	1	0	0	0	1	0	0	1	0	0	0	0	0	0	0	0	0	1	0	0	0	0	0	1	0	0	1	0	0	0	0	0	0	0	0	1	43.13183333	20.41605
591		0	1	0	0	0	0	0	0	0	0	0	0	0	0	0	0	0	0	0	0	0	0	0	0	0	0	0	0	0	0	0	0	0	0	0	0	0	0	0	0	0	0	0	0	0	0	0	0	0	0	1	42.305	18.8889
593		0	1	0	0	0	0	0	0	0	0	0	0	0	0	0	0	0	0	0	0	0	0	0	0	0	0	0	0	0	0	0	0	0	0	0	0	0	0	0	0	0	0	0	0	0	0	0	0	0	0	1	42.474306	18.53625
596	2006/005		1	0	0	0	0	0	0	0	1	1	0	0	1	1	1	0	0	0	0	0	0	0	0	0	0	0	0	0	0	1	1	0	0	0	0	0	0	0	0	0	0	0	0	0	0	1	1	0	0	1	41.03578333	20.06861667
598	2006/007		1	0	0	0	0	0	0	0	0	0	0	0	0	1	1	0	0	0	1	0	0	0	0	0	0	0	0	0	0	1	0	0	0	0	0	0	0	0	0	0	0	0	0	0	0	0	1	0	0	1	40.74306667	20.12713333
599	2006/008		0	0	0	0	0	0	0	0	0	1	0	0	0	0	0	0	0	0	0	0	0	0	0	0	0	0	0	0	0	0	0	0	0	0	0	0	0	0	0	0	0	0	0	0	0	0	1	0	0	1	41.15756667	20.12466667
600	2006/009		1	0	0	0	0	1	0	0	1	1	0	0	1	0	0	0	0	1	1	0	1	0	1	0	1	0	0	1	0	0	0	0	0	0	0	0	0	0	0	0	0	0	0	0	0	1	1	0	0	1	41.20353333	20.11458333
601	2006/010		1	0	0	0	0	0	0	0	0	0	0	0	1	0	1	0	0	0	1	0	0	0	0	0	0	0	0	0	0	0	1	0	0	0	0	0	0	0	0	0	0	0	0	0	0	0	1	0	0	1	41.21271667	20.1364
602	2006/011		0	0	0	0	0	1	0	0	1	1	0	0	0	0	1	0	0	0	1	0	1	0	0	0	0	0	0	0	0	0	0	0	0	0	0	0	0	0	0	0	0	0	0	0	0	0	1	0	0	1	41.2226	20.12675
604	2006/013		0	0	0	0	0	1	0	0	0	0	0	0	0	0	0	0	0	0	1	0	0	0	0	0	0	0	0	0	0	0	0	0	0	0	0	0	0	0	0	0	0	0	0	0	0	0	1	0	0	1	41.23886667	20.0976
607	2006/016		1	0	0	0	0	1	0	0	1	0	0	0	1	0	1	0	0	0	1	0	1	0	0	0	0	0	0	0	0	1	0	0	0	0	0	0	0	0	0	0	0	0	0	0	0	0	1	0	0	1	41.29093333	20.0832
608	2006/017		0	0	0	0	0	0	0	0	1	0	0	0	0	0	0	1	1	0	0	0	0	0	0	0	0	0	0	0	0	0	0	0	0	0	0	0	0	0	0	0	0	0	0	0	0	0	0	0	0	3	41.2951	20.10085
609	2006/018		1	0	0	0	0	1	0	0	1	1	0	0	0	0	0	0	0	0	1	0	1	0	0	0	0	0	0	0	0	0	0	0	0	0	0	0	0	0	0	0	0	0	0	0	0	0	1	0	0	1	41.29063333	20.11888333
611	2006/020		1	0	0	0	0	1	0	0	0	0	0	0	0	1	0	0	0	0	0	0	0	0	0	0	0	0	0	0	0	1	0	0	0	0	0	0	0	0	0	0	0	0	0	0	0	0	1	0	1	1	41.27475	20.26793333
612	2006/021		0	0	0	0	0	1	0	0	0	0	0	0	0	1	1	0	0	0	1	0	0	0	0	0	0	0	0	0	0	0	0	0	0	0	0	0	0	0	0	0	0	0	0	0	0	0	1	0	0	1	41.266	20.25955
615	2006/024		1	0	0	0	0	1	0	0	1	1	0	0	1	1	0	0	0	0	1	0	0	0	0	0	0	0	0	0	0	0	0	0	0	0	0	0	0	0	0	0	0	0	0	0	0	1	1	0	1	1	41.26601667	20.3182
622	2006/031A		1	0	0	0	0	1	0	0	0	0	0	0	1	1	1	0	0	0	1	0	0	0	1	0	1	0	0	0	0	1	0	0	0	0	0	0	0	0	0	0	0	0	0	0	0	0	0	0	1	1	41.75205	20.25241667
623	2006/031B		1	0	0	0	0	1	0	0	0	0	0	0	1	0	0	0	0	1	1	0	0	0	0	0	0	0	0	0	0	0	0	0	0	0	0	0	0	0	0	0	0	0	0	0	0	0	0	0	1	1	41.75283333	20.25048333
625	2006/032		1	0	0	0	0	1	0	0	0	0	0	0	1	0	1	0	0	0	1	0	0	0	0	0	0	0	0	0	0	0	0	0	0	0	0	0	0	0	0	0	0	0	0	0	0	0	0	0	0	1	41.75713333	20.23888333
628	2006/034		0	0	0	0	0	1	0	0	0	0	0	0	0	0	0	0	0	0	0	0	0	0	0	0	0	0	0	0	0	0	0	0	0	0	0	0	0	0	0	0	0	0	0	0	0	1	0	0	0	1	41.66241667	20.31728333
632	2006/038		0	0	0	0	0	0	0	0	0	0	0	0	0	0	0	0	0	0	1	0	0	0	0	0	0	0	0	0	0	0	0	0	0	0	0	0	0	0	0	0	0	0	0	0	0	0	0	0	1	1	41.64511667	20.18231667
633	2006/039		1	0	0	0	0	1	0	0	0	0	0	0	1	0	1	0	0	0	1	0	0	0	1	0	1	0	0	0	0	1	0	0	0	0	0	0	0	0	0	0	0	0	0	0	0	0	1	1	1	1	41.64605	20.1806
635	2006/041		1	0	0	0	0	0	0	0	0	0	0	0	0	0	0	0	0	0	0	0	0	0	0	0	0	0	0	0	0	0	0	0	0	0	0	0	0	0	0	0	0	0	0	0	0	0	0	0	1	1	41.65025	20.18606667
637	2006/043		1	0	0	0	0	0	0	0	0	0	0	0	1	0	1	0	0	0	1	0	0	0	0	0	0	0	0	0	0	1	0	0	0	0	0	0	0	0	0	0	0	0	0	0	0	0	1	0	1	1	41.63516667	20.12433333
642	2006/048		1	1	0	0	0	1	0	0	1	1	0	0	0	1	1	0	0	1	1	0	1	0	1	0	1	0	0	1	0	1	0	0	1	0	0	0	0	0	1	0	0	0	0	0	0	1	1	0	0	1	42.1217	19.83136667
643	2006/049		1	0	0	0	0	1	0	0	1	1	0	0	1	1	1	0	0	0	0	0	0	0	1	0	1	0	0	0	0	1	1	0	1	0	0	0	0	0	0	0	0	0	0	0	0	0	1	0	0	1	42.11655	19.83738333
644	2006/050		1	0	0	0	0	0	0	0	0	0	0	0	0	1	1	0	0	0	1	0	0	0	0	0	0	0	0	1	0	1	0	0	0	0	0	0	0	0	1	0	0	0	0	0	0	1	0	0	0	1	42.08093333	19.73621667
646	2006/052		1	0	0	0	0	1	0	0	1	0	0	0	0	0	1	0	0	0	1	0	0	0	0	0	0	0	0	0	0	0	1	0	0	0	0	0	0	0	1	0	0	0	0	0	0	0	0	0	1	1	42.18715	19.86955
647	2006/053		1	0	0	0	0	0	0	0	1	1	0	0	0	0	1	0	0	0	1	0	0	0	1	0	1	0	0	0	0	1	0	0	0	0	0	0	0	0	1	0	0	0	0	0	0	0	0	0	0	1	42.20535	19.89178333
649	2006/055		1	0	0	0	1	1	0	0	0	1	0	0	0	1	1	0	1	1	1	0	1	0	0	0	0	0	0	0	0	0	0	0	0	0	0	0	0	0	1	0	0	0	0	0	0	0	0	0	1	1	42.22583333	19.89236667
650	2006/056		1	1	0	0	1	1	0	0	1	1	0	0	1	1	1	0	0	1	1	0	0	1	1	0	1	0	1	1	0	0	0	0	1	0	0	0	0	0	1	0	0	0	0	0	0	1	1	0	1	1	42.22688333	19.905
652	2006/058		0	0	0	0	0	1	0	0	0	0	0	0	0	1	1	0	0	0	0	0	0	0	0	0	0	0	0	0	0	1	0	0	0	0	0	0	0	0	1	0	0	0	0	0	0	0	0	0	1	1	42.06913333	19.41313333
653	2006/059		1	0	0	0	0	0	0	0	0	0	0	0	1	1	1	0	0	0	0	0	0	0	0	0	0	0	0	0	0	0	1	0	0	0	0	0	0	0	1	0	0	0	0	0	0	0	0	0	1	1	42.07236667	19.39463333
654	2006/060		1	0	0	0	0	1	0	0	0	0	0	0	0	0	1	0	0	0	1	0	0	0	0	0	0	0	0	0	1	0	0	0	0	0	0	0	0	0	0	0	0	0	0	0	0	0	0	0	0	1	42.14695	19.54543333
655	2006/061		1	0	0	0	0	0	0	0	0	0	0	0	0	0	0	0	0	0	0	0	0	0	0	0	0	0	0	0	0	0	0	0	0	0	0	0	0	0	0	0	0	0	0	0	0	0	0	0	0	1	42.1523	19.5402
656	2006/062		1	0	0	0	0	0	0	0	0	0	0	0	0	1	0	0	0	0	0	0	0	0	0	0	0	0	0	0	0	1	0	0	0	0	0	0	0	0	1	0	0	0	0	0	0	0	0	0	0	1	42.18966667	19.5621
658	2006/064		1	0	0	0	0	1	0	0	0	1	0	0	0	1	1	0	0	0	0	0	0	0	0	0	0	0	0	0	0	1	0	0	0	0	0	0	0	0	1	0	0	0	0	0	0	0	0	0	0	1	42.2045	19.568
659	2006/065		1	0	0	0	0	0	0	0	1	0	0	0	1	1	1	0	0	0	0	0	0	0	0	0	0	0	0	0	0	1	0	0	0	0	0	0	0	0	1	0	0	0	0	0	0	0	0	0	0	1	42.21146667	19.57916667
662	2006/068		1	0	0	0	0	0	0	0	0	0	0	0	0	0	0	0	0	0	0	0	0	0	0	0	0	0	0	0	0	0	0	0	0	0	0	0	0	0	0	0	0	0	0	0	0	0	0	0	0	1	42.11793333	19.65896667
663	2006/069		1	0	0	0	0	0	0	0	0	0	0	0	0	0	0	0	0	0	0	0	0	0	1	0	1	0	0	0	0	0	0	0	0	0	0	0	0	0	0	0	0	0	0	0	0	0	0	0	0	1	42.12571667	19.66768333
670	2006/075		0	0	0	0	0	0	0	0	0	0	0	0	1	0	1	0	0	0	1	0	0	0	0	0	0	0	0	0	0	0	0	1	0	0	0	0	0	0	0	0	1	0	0	0	0	0	0	0	0	1	43.46708333	19.6512
672		0	1	0	0	0	0	0	0	0	0	0	0	0	0	1	1	0	0	0	1	0	0	0	0	0	0	0	0	0	0	0	0	0	0	1	0	0	0	0	0	0	0	0	0	0	0	0	0	0	0	2	40.143333	19.662222
673		0	1	0	0	0	0	0	0	0	0	0	0	0	0	0	0	0	0	0	1	0	0	0	0	0	0	0	0	0	0	1	0	0	0	0	0	0	0	0	0	0	0	0	0	0	0	0	0	0	0	1	40.143694	19.677
676		0	0	0	0	0	0	0	0	0	0	0	0	0	0	1	0	0	0	0	0	0	0	0	0	0	0	0	0	0	0	0	0	0	0	0	0	0	0	0	0	0	0	0	0	0	0	0	0	0	0	3	40.211944	19.579167
679		0	0	0	0	0	0	0	0	0	1	0	0	0	0	0	0	0	0	0	0	0	0	0	0	0	0	0	0	0	0	0	0	0	0	0	0	0	0	0	0	0	0	0	0	0	0	0	1	0	0	1	39.557917	20.468833
681		0	0	0	0	0	0	0	0	0	0	0	0	0	0	1	1	0	0	0	0	0	0	0	0	0	0	0	0	0	0	0	0	0	0	0	0	0	0	0	0	0	0	0	0	0	0	1	1	0	0	2	39.695278	20.703611
687		0	0	0	0	0	0	1	0	0	0	0	0	0	0	0	0	0	0	0	1	0	0	0	0	0	0	0	0	0	0	0	0	0	0	0	0	0	0	0	0	0	0	0	0	0	0	0	0	0	0	1	40.107694	21.021167
692		0	0	0	0	0	0	0	0	0	0	0	0	0	0	0	1	0	0	0	1	0	0	0	1	0	1	0	0	0	0	0	0	0	0	0	0	0	0	0	0	0	0	0	0	0	0	0	0	0	0	1	40.20301	20.31674
693		0	0	0	0	0	0	0	0	0	0	0	0	0	0	0	1	0	0	0	0	0	0	0	0	0	0	0	0	0	0	0	0	0	0	0	0	0	0	0	0	0	0	0	0	0	0	0	0	0	0	1	40.1933	20.31429
694		0	0	0	0	0	0	0	0	0	0	0	0	0	0	0	0	0	0	0	1	0	0	0	0	0	0	0	0	0	0	0	0	0	0	0	0	0	0	0	0	0	0	0	0	0	0	0	0	0	0	1	40.50547	20.55125
695		0	0	0	0	0	0	0	0	0	1	0	0	0	0	0	0	0	0	0	1	0	0	0	0	0	0	0	0	0	0	0	0	0	0	0	0	0	0	0	0	0	0	0	0	0	0	0	0	0	0	1	40.50864	20.56533
696		0	1	0	0	0	0	0	0	0	0	0	0	0	0	0	0	0	0	0	0	0	0	0	0	0	0	0	0	0	0	0	0	0	0	0	0	0	0	0	0	0	0	0	0	0	0	0	0	0	0	1	40.25182	19.96526
698		0	1	0	0	0	0	0	0	0	0	0	0	0	0	0	1	0	0	0	1	0	0	0	0	0	0	0	0	0	0	0	1	0	0	0	0	0	0	0	0	0	0	0	0	0	0	0	1	0	1	1	40.27581	20.09397
699		0	1	0	0	0	0	0	0	0	0	0	0	0	0	0	0	0	0	0	1	0	0	0	0	0	0	0	0	0	0	0	0	0	0	0	0	0	0	0	0	0	0	0	0	0	0	0	0	0	0	2	40.28722	20.08328
700		0	1	0	0	0	0	0	0	0	0	0	0	0	0	0	0	0	0	0	0	0	0	0	0	0	0	0	0	0	0	0	0	0	0	0	0	0	0	0	0	0	0	0	0	0	0	0	0	0	0	1	40.34056	19.78027
701		0	0	0	0	0	0	1	0	0	0	0	0	0	0	0	0	0	0	0	0	0	0	0	0	0	0	0	0	0	0	0	0	0	0	0	0	0	0	0	0	0	0	0	0	0	0	0	0	0	0	2	40.33741	19.77468
705	2006/134		1	0	0	0	0	1	0	0	1	0	0	0	0	1	1	0	0	0	1	0	0	0	0	0	0	0	0	0	0	0	0	0	0	0	0	0	0	0	0	0	0	0	0	0	0	0	1	0	0	1	40.50051667	20.28601667
706	2006/135		0	0	0	0	0	1	0	0	0	0	0	0	0	0	0	1	0	0	1	0	1	0	0	0	0	0	0	0	0	0	0	0	0	0	0	0	0	0	0	0	0	0	0	0	0	0	0	0	0	1	40.51638333	20.40703333
707	2006/135B		0	0	0	0	0	1	0	0	0	1	0	0	0	0	0	0	0	0	0	0	1	0	0	0	0	0	0	1	0	0	0	0	0	0	0	0	0	0	0	0	0	0	0	0	0	0	0	0	0	1	40.51776667	20.4124
708	2006/136		0	0	0	0	0	1	0	0	1	1	0	0	1	0	1	0	0	0	1	0	0	0	0	0	0	0	0	0	0	0	0	0	0	0	0	0	0	0	0	0	0	0	0	0	0	0	1	0	0	1	40.5219	20.41388333
709	2006/137		0	0	0	0	0	0	0	0	0	0	0	0	0	0	0	0	0	0	0	0	0	0	0	0	0	0	0	0	0	0	0	0	0	0	0	0	0	0	0	0	0	0	0	0	0	0	0	0	0	1	40.52921667	20.4192
710	2006/138		1	0	0	0	0	0	0	0	0	1	0	0	0	0	1	0	0	0	0	0	0	1	0	0	0	0	0	0	0	0	0	1	1	0	0	0	0	0	0	0	0	0	0	0	0	0	0	0	1	1	40.53245	20.42563333
711	2006/139		0	0	0	0	0	0	0	0	1	0	0	0	0	0	0	0	0	0	1	0	0	0	0	0	0	0	0	0	0	0	0	0	0	0	0	0	0	0	0	0	0	0	0	0	0	0	1	0	0	1	40.52453333	20.41633333
713	2006/141		1	0	0	0	0	0	0	0	0	1	0	0	1	0	0	0	0	0	1	1	0	0	1	0	1	0	0	1	0	0	0	0	1	0	0	0	0	0	0	0	0	0	0	0	0	0	1	0	1	1	40.54348333	20.27181667
714	2006/142		0	0	0	0	0	0	0	0	0	0	0	0	0	0	1	0	0	0	0	0	0	0	0	0	0	0	0	0	0	0	0	0	0	0	0	0	0	0	0	0	0	0	0	0	0	0	1	0	0	1	40.5303	20.25753333
715	2006/143		1	0	0	0	0	0	0	0	1	1	0	0	1	1	1	0	0	0	1	0	0	0	1	0	1	0	0	0	0	1	0	0	1	0	0	0	0	0	0	0	0	0	0	0	0	1	1	0	1	1	40.5223	20.2574
717	2006/145		0	0	0	0	0	1	0	0	0	0	0	0	0	0	1	0	0	0	0	0	0	0	0	0	0	0	0	0	0	0	0	0	0	0	0	0	0	0	0	0	0	0	0	0	0	0	0	0	0	2	40.54218333	20.346
718	2006/146		1	0	0	0	0	0	0	0	0	0	0	0	1	0	0	0	0	0	1	0	0	0	1	0	1	0	0	0	0	0	0	0	1	0	0	0	0	0	0	0	0	0	0	0	0	0	1	0	1	1	40.559	20.39083333
719	2006/147		1	0	0	0	0	0	0	0	0	0	0	0	0	0	0	0	0	0	1	0	0	0	0	0	0	0	0	0	0	0	0	0	0	0	0	0	0	0	0	0	0	0	0	0	0	0	0	0	1	1	40.57145	20.28798333
720	2006/148		1	0	0	0	0	0	0	0	0	0	0	0	1	0	0	0	0	0	1	0	0	0	0	0	0	0	0	0	0	0	1	0	0	0	0	0	0	0	0	0	0	0	0	0	0	0	1	0	1	1	40.63281667	20.21615
721	2006/149		0	0	0	0	0	0	0	0	0	0	0	0	1	0	1	0	0	0	0	0	1	1	0	0	0	0	0	0	0	0	0	0	0	0	0	0	0	0	0	0	0	0	0	0	0	0	0	0	1	1	40.64113333	20.15746667
722	2006/150		0	0	0	0	0	0	0	0	0	0	0	0	0	0	0	0	0	0	0	0	0	0	0	0	0	0	0	0	0	0	0	0	0	0	0	0	0	0	0	0	0	0	0	0	0	0	0	0	0	1	40.6186	20.19908333
723	2006/151		1	0	0	0	0	0	0	0	1	1	0	0	0	0	0	0	0	0	1	0	0	0	0	0	0	0	0	0	0	0	0	0	0	0	0	0	0	0	0	0	0	0	0	0	0	0	0	1	0	1	40.68131667	20.18348333
724	2006/152		1	0	0	0	0	1	0	0	0	1	0	0	0	0	0	0	0	0	1	1	0	0	1	0	1	0	1	1	0	1	0	0	1	0	0	0	0	0	0	0	0	0	0	0	0	0	0	0	1	1	40.7127	20.18391667
725	2006/153		1	0	0	0	0	0	0	0	0	0	0	0	0	0	1	0	0	0	1	1	0	0	0	0	0	0	0	0	0	0	0	0	1	0	0	0	0	0	0	0	0	0	0	0	0	0	1	0	0	1	40.72125	20.17201667
727	2006/155		1	0	0	0	0	1	0	0	0	1	0	0	1	1	0	1	0	0	0	0	0	0	0	0	0	0	0	0	0	1	1	0	0	0	0	0	0	0	0	0	0	0	0	0	0	0	1	0	1	1	40.71638333	20.15671667
730	2006/158		1	0	0	0	0	0	0	0	1	0	0	0	1	0	1	0	0	0	1	0	0	0	0	0	0	0	0	0	0	0	0	0	1	1	0	0	0	0	0	0	0	0	0	0	0	0	0	0	1	1	40.92603333	20.22316667
734	2006/162		0	0	0	0	0	0	0	0	0	0	0	0	0	0	0	0	0	0	0	0	0	0	0	0	0	0	0	0	1	0	1	0	0	0	0	0	0	0	1	0	0	0	0	0	0	0	0	0	1	1	42.32533333	19.36275
739		0	0	0	0	0	0	0	0	0	1	0	0	0	1	0	0	0	0	0	0	0	0	0	0	0	0	0	0	0	0	0	0	0	0	0	0	0	0	1	0	0	0	0	0	0	0	0	0	1	0	1	41.760278	20.669722
744		0	1	0	0	0	0	0	0	0	0	0	0	0	0	1	0	0	0	0	0	0	0	0	0	0	0	0	0	0	0	0	0	0	0	0	0	0	0	0	0	0	0	0	0	0	0	0	0	0	0	3	40.976639	20.794083
748		0	0	0	0	0	0	0	0	0	1	0	0	0	0	0	0	0	0	0	0	0	0	0	0	0	0	0	0	0	0	0	0	0	0	0	0	0	0	0	0	0	0	0	0	0	0	0	0	0	0	1	41.843083	22.505917
750		0	0	0	0	0	0	0	0	0	0	0	0	0	1	0	0	0	0	0	1	0	1	0	0	0	0	0	0	0	0	0	0	0	0	0	0	0	0	0	0	0	0	0	0	0	0	0	0	0	1	1	40.82548	20.85281
751		0	0	0	0	0	0	0	0	0	0	0	0	0	1	0	0	0	0	0	1	0	0	0	0	0	0	0	0	0	0	0	0	0	0	0	0	0	0	0	0	0	0	0	0	0	0	0	0	0	0	1	40.8179	20.84941
752		0	0	0	0	0	0	1	0	0	0	0	0	0	0	0	0	0	0	0	0	0	0	0	0	0	0	0	0	0	0	0	0	0	0	0	0	0	0	0	0	0	0	0	0	0	0	0	0	0	0	1	40.81426	20.84022
753		0	0	0	0	0	0	0	0	0	0	0	0	0	0	0	0	0	0	0	0	0	0	0	0	0	0	0	0	0	0	0	0	0	0	0	0	0	0	0	0	0	0	0	0	0	0	0	0	0	1	1	40.80241	20.87408
754		0	0	0	0	0	0	0	0	0	0	0	0	0	0	0	0	0	0	0	0	0	0	0	0	0	0	0	0	0	0	0	0	0	0	0	0	0	0	0	0	0	0	0	0	0	0	0	0	0	1	1	40.81832	20.87283
755		0	0	0	0	0	0	0	0	0	0	0	0	0	0	0	0	0	0	0	0	0	0	0	0	0	0	0	0	0	0	0	0	0	0	0	0	0	0	0	0	0	0	0	0	0	0	0	0	0	1	1	40.82203	20.87886
757		0	0	0	0	0	0	0	0	0	0	0	0	0	0	0	0	0	0	0	1	0	0	0	0	0	0	0	0	0	0	0	0	0	0	0	0	0	0	0	0	0	0	0	0	0	0	0	0	0	1	1	40.74191	20.86351
758		0	1	0	0	0	0	1	0	0	0	0	0	0	0	0	0	0	0	0	1	0	0	0	0	0	0	0	0	0	0	0	0	1	0	0	0	0	0	0	0	0	0	0	0	0	0	0	0	0	1	1	40.7565	20.84945
759		0	1	0	0	0	0	0	0	0	0	0	0	0	0	0	0	0	0	0	1	0	0	0	1	0	1	0	0	0	0	0	0	0	0	0	0	0	0	0	0	0	0	0	0	0	0	0	1	0	0	1	40.53767	20.57924
760	2007/091		0	0	0	0	0	0	0	0	0	0	0	0	1	0	1	0	0	0	0	0	1	0	0	0	0	0	0	0	0	0	0	0	0	0	0	0	0	0	0	0	0	0	0	0	0	1	0	0	0	1	42.3162	18.93421667
761	2007/092		0	0	0	0	0	0	0	0	0	0	0	0	0	0	0	0	0	0	0	0	0	0	0	0	0	0	0	0	0	0	0	0	0	0	0	0	0	0	1	0	0	0	0	0	0	0	0	0	0	1	42.27805556	18.86
763	2007/102		0	0	0	0	0	1	0	0	0	0	0	0	1	1	1	0	0	0	1	0	0	0	0	0	0	0	0	0	0	0	0	0	0	0	0	0	0	0	0	0	0	0	0	0	0	0	0	0	0	1	42.20878333	20.7512
764	2007/103		0	0	0	0	0	0	0	0	1	0	0	0	0	0	1	0	0	0	1	0	0	0	0	0	0	0	0	1	0	0	0	0	0	0	0	0	0	0	0	0	0	0	0	0	0	0	0	0	0	1	42.06631667	20.48795
765	2007/104		0	0	0	0	0	0	0	0	0	0	0	0	0	0	1	0	0	0	0	0	0	0	0	0	0	0	0	0	0	1	0	0	0	0	0	0	0	0	0	0	0	0	0	0	0	0	0	0	0	1	42.05883333	20.50181667
767	2007/106		0	0	0	0	0	0	0	0	1	0	0	0	0	0	0	0	0	0	0	0	0	0	0	0	0	0	0	0	0	0	0	0	0	0	0	0	0	0	0	0	0	0	0	0	0	0	0	0	0	1	41.98666667	20.52858333

774	2007/112	0	0	0	0	0	0	0	0	0	0	0	0	0	0	0	0	0	0	0	0	0	0	0	0	0	0	0	0	0	0	0	0	0	0	0	0	0	0	0	0	0	0	0	0	0	0	0	2	41.99465	20.48498333
775	2007/113	0	0	0	0	0	0	0	0	0	0	0	0	0	0	1	0	0	0	1	0	0	0	0	0	0	0	0	0	0	0	0	0	0	0	0	0	0	0	1	0	0	0	0	0	0	0	0	1	41.99895	20.47168333
776	2007/114	0	0	0	0	0	1	0	0	0	0	0	0	1	0	1	0	0	0	0	0	1	0	0	0	0	0	0	0	0	0	0	0	0	0	0	0	0	0	1	0	0	0	0	0	0	0	0	1	41.99931667	20.46133333
777	2007/115	1	0	0	0	0	1	0	0	1	1	0	0	1	0	1	0	0	0	0	0	1	0	1	0	0	0	0	0	0	0	0	0	0	0	0	1	0	0	1	0	0	0	0	0	0	0	0	3	41.99956667	20.45773333
779	2007/117	0	0	0	0	0	0	0	0	0	0	0	0	0	0	1	0	0	0	0	0	0	0	0	0	0	0	0	0	0	0	0	0	0	0	0	0	0	0	0	0	0	0	0	0	0	0	0	2	42.00946667	20.46965
780	2007/117B	0	0	0	0	0	0	0	0	0	0	0	0	0	0	0	0	0	0	0	0	0	0	0	0	0	0	0	0	0	0	0	0	0	0	0	0	0	0	0	0	0	0	0	0	0	0	0	2	42.01208333	20.46965
783	2007/120	1	0	0	0	0	1	0	0	0	1	0	0	1	1	1	1	0	0	1	0	0	0	1	0	0	0	0	1	0	0	0	0	0	0	0	0	0	0	1	0	0	0	0	0	0	0	1	1	41.98971667	20.4202
784	2007/121	0	0	0	0	0	1	0	0	0	0	0	0	1	0	0	0	0	0	1	0	0	0	0	0	0	0	0	0	0	0	0	0	0	0	0	0	0	0	0	0	0	0	0	0	0	0	1	1	41.92596667	20.38706667
785	2007/122	0	0	0	0	0	0	0	0	0	0	0	0	0	0	0	0	0	0	1	0	0	0	0	0	0	0	0	0	0	0	0	0	0	0	0	0	0	0	0	0	0	0	0	0	0	0	0	1	41.9097	20.409
786	2007/123	1	0	0	0	0	1	0	0	1	0	0	0	1	0	0	1	0	0	1	0	0	0	1	0	0	0	0	1	0	0	0	0	0	0	0	0	0	0	0	0	0	0	0	0	1	0	2	1	41.89498333	20.41733333
789	2007/126	0	0	0	0	0	1	0	0	1	0	0	0	1	0	1	1	0	0	1	0	1	0	0	0	0	1	0	0	0	0	0	0	0	0	0	0	0	0	0	0	0	0	0	0	0	0	0	1	41.81885	20.50266667
790	2007/127	0	0	0	0	0	1	0	0	0	0	0	0	0	0	1	1	0	0	1	0	1	0	1	0	0	0	0	0	0	1	0	0	0	0	0	0	0	0	0	0	0	0	0	0	0	0	0	1	41.8179	20.52701667
792	2007/128	0	0	0	0	0	0	0	0	1	1	0	0	1	0	1	1	0	0	0	0	0	0	0	0	0	0	0	0	0	1	0	0	0	0	0	0	0	0	0	0	0	0	0	0	0	0	0	1	41.81868333	20.53733333
793	2007/129	0	0	0	0	0	0	0	0	0	1	0	0	0	0	0	0	0	0	0	0	0	0	0	0	0	0	0	0	0	0	0	0	0	0	0	0	0	0	0	0	0	0	0	0	0	0	0	1	41.8052	20.56088333
795	2007/131	0	0	0	0	0	0	0	0	0	1	0	0	1	0	0	0	0	0	0	0	0	0	0	0	0	0	0	0	0	0	0	0	0	0	0	0	0	0	0	0	0	0	0	0	0	0	0	1	41.80008333	20.54993333
796	2007/132	0	0	0	0	0	0	0	0	0	0	0	0	0	0	0	0	0	0	0	0	0	0	0	0	0	0	0	0	0	0	0	0	0	0	0	0	0	0	0	0	0	0	0	0	0	0	0	1	41.79526667	20.54228333
797	2007/133	0	0	0	0	0	0	0	0	0	1	0	0	0	0	1	1	0	0	0	0	0	1	0	0	0	0	0	0	0	0	0	0	0	0	0	0	0	0	0	0	0	0	0	0	0	0	0	2	41.79335	20.5439
798	2007/134	0	0	0	0	0	0	0	0	0	0	0	0	0	0	1	0	0	0	0	0	0	0	0	0	0	0	0	0	0	0	0	0	0	0	0	0	0	0	0	0	0	0	0	0	0	0	0	1	41.79705	20.54536667
803	2007/139	0	0	0	0	0	0	0	0	0	1	0	0	1	0	0	1	0	0	0	0	0	0	0	0	0	0	0	0	0	0	0	0	0	0	0	0	0	0	0	0	0	0	0	0	0	0	0	1	41.81535	20.54485
805	2007/141	0	0	0	0	0	0	0	0	0	0	0	0	1	0	0	0	0	0	0	0	0	0	0	0	0	0	0	0	0	1	0	0	0	0	0	0	0	0	0	0	0	0	0	0	0	0	0	1	41.81915	20.52173333
810	2007/146	0	0	0	0	0	1	0	0	1	1	0	0	1	0	0	0	0	0	0	0	0	0	0	0	0	0	0	0	0	0	0	0	0	0	0	0	0	0	0	0	0	0	0	0	0	0	0	1	41.76893333	20.27673333
813	2007/149	1	0	0	0	0	1	0	0	0	1	0	0	0	0	0	0	0	0	1	0	1	0	1	0	0	1	0	1	0	0	0	0	0	0	0	0	0	0	0	0	0	0	0	0	1	0	0	1	41.81365	20.23618333
817	2007/153	1	0	0	0	0	1	0	0	0	0	0	0	0	0	0	0	0	0	1	0	1	0	0	0	0	0	0	1	0	0	0	0	0	0	0	0	0	0	0	0	0	0	0	0	0	0	0	1	41.83818333	20.16646667
819	2007/155	0	0	0	0	0	1	0	0	0	0	0	0	1	0	1	0	0	0	0	0	0	0	0	0	0	0	1	0	0	0	0	0	0	0	0	0	0	0	0	0	0	0	0	0	0	0	1	1	41.50988333	20.31515
823	2007/159	1	0	0	0	0	0	0	0	1	0	0	0	1	0	1	0	0	0	0	0	0	0	0	0	0	0	0	1	0	0	0	0	0	0	0	0	0	0	0	0	0	0	0	0	1	1	1	1	41.43065	20.09196667
828	2007/164	1	0	0	0	0	1	0	0	0	0	0	0	0	0	0	0	0	0	1	0	0	0	0	0	0	0	0	0	0	0	0	0	0	0	0	0	0	0	0	0	0	0	0	0	1	0	0	1	41.32106667	20.14736667
831	2007/167	1	0	0	0	0	1	0	0	1	0	0	0	1	0	1	0	0	0	1	0	0	0	0	0	0	0	0	0	0	0	0	0	0	0	0	0	0	0	1	0	0	0	0	0	1	0	0	1	40.5378	20.56996667
836	2007/172	0	0	1	0	0	1	0	0	1	1	0	0	0	0	1	0	0	0	0	0	0	0	1	0	0	1	0	0	0	1	0	0	0	0	0	0	0	0	0	0	0	0	0	0	0	0	1	1	40.79151667	20.9324
838	2007/174	1	0	0	0	0	0	0	0	0	0	0	0	0	1	1	0	0	0	0	0	0	0	0	0	0	1	0	0	0	0	0	0	0	0	0	0	0	0	0	0	0	0	0	0	0	0	1	1	40.26878333	20.6065
841	2007/177	1	0	0	0	0	1	0	0	1	1	0	0	1	0	1	0	0	0	1	1	0	0	1	0	1	0	0	0	0	0	1	1	0	0	0	0	0	0	0	0	0	0	0	1	1	0	0	1	40.24463333	20.4377
843	2007/179	1	0	1	0	0	1	0	0	0	0	0	0	0	1	1	0	0	0	1	0	0	0	0	0	0	1	0	1	0	0	0	1	0	0	0	0	0	0	0	0	0	0	0	0	0	0	0	1	40.29883333	20.0886
844	0	0	0	0	0	0	0	0	0	0	0	0	0	0	0	0	0	0	0	1	0	0	0	0	0	0	0	0	0	0	0	0	0	0	0	0	0	0	0	0	0	0	0	0	0	1	0	0	1	40.86643	20.42862
845	0	0	0	0	0	0	0	0	0	0	0	0	0	0	0	0	0	0	0	0	0	0	0	0	0	0	0	0	1	0	0	0	0	0	0	0	0	0	0	0	0	0	0	0	0	0	0	0	1	40.84757	20.11453
846	2007/180	1	0	1	0	0	0	0	0	1	1	0	0	1	0	1	0	0	0	0	0	0	0	1	0	0	1	0	1	0	0	0	0	0	0	0	0	0	0	0	0	0	0	0	1	1	0	0	1	40.0727	20.13916667
848	2007/182	1	0	0	0	0	0	0	0	0	0	0	0	0	0	1	0	0	0	0	0	0	0	0	0	0	0	0	0	0	0	0	0	0	0	0	0	0	0	0	0	0	0	0	0	0	0	0	1	39.74535	20.01955
850	2007/184	1	0	0	0	0	0	0	0	0	0	0	0	0	1	1	0	0	0	0	0	0	0	0	0	0	0	0	0	0	0	0	0	0	0	0	0	0	0	0	0	0	0	0	0	0	0	0	1	40.0808	19.76746667
854	2007/188	1	0	0	0	0	0	0	0	1	0	0	0	1	0	0	0	0	0	1	0	0	0	0	0	0	0	0	0	1	0	0	0	0	0	0	0	0	0	0	0	0	1	0	0	0	0	0	1	41.37341667	19.91983333
855	2007/189	1	0	0	0	0	0	0	0	0	1	0	0	1	0	0	0	1	0	0	0	0	0	0	0	1	0	0	0	0	0	0	0	0	0	0	0	0	0	0	0	0	1	0	0	0	0	0	3	41.37311667	19.92003333
856	2007/190	0	0	0	0	0	0	0	0	0	0	0	0	1	0	0	1	0	0	1	0	0	0	0	0	0	0	0	0	1	0	0	0	0	0	0	0	0	0	0	0	0	1	0	0	1	1	0	3	41.3689	19.9221
864	0	1	0	0	0	0	0	0	0	0	0	0	0	0	0	0	0	0	0	0	0	0	0	0	0	0	0	0	0	0	0	0	0	0	0	0	0	0	0	0	0	0	0	0	0	0	0	1	1	41.24978	20.5044
865	2008/110	1	0	0	0	0	0	0	0	0	0	0	0	1	0	1	0	0	0	1	0	0	0	0	0	0	0	0	0	0	0	0	0	0	0	0	0	0	0	0	0	0	1	0	0	0	0	0	1	42.51425	18.48736667
866	2008/111	1	0	0	0	0	1	0	0	0	0	0	0	0	0	0	0	0	0	0	0	0	0	0	0	0	0	0	0	1	0	0	0	0	0	0	0	0	0	0	0	0	1	0	0	0	0	0	1	42.52776667	18.4875
867	2008/112	0	0	0	0	0	0	0	0	0	0	0	0	0	0	0	0	0	0	0	0	0	0	0	0	0	0	0	0	0	0	0	0	0	0	0	0	0	0	0	0	0	0	0	0	0	0	0	3	42.54983333	18.65491667
868	2008/113	1	0	0	0	0	0	0	0	0	0	0	0	0	0	0	0	0	0	0	0	0	0	0	0	0	0	0	0	0	0	0	0	0	0	0	0	0	0	0	0	0	0	0	0	0	0	0	1	42.52365	18.68546667
871	2008/116	1	0	0	1	0	1	0	0	0	1	0	0	1	0	1	0	0	0	1	0	1	0	0	0	0	0	0	1	0	0	1	0	0	0	0	0	0	0	0	0	0	0	0	1	0	0	1	1	42.48616667	18.76703333
872	2008/117	1	0	0	0	0	1	0	0	0	0	0	0	1	0	1	0	0	0	1	0	0	0	1	0	0	0	0	1	0	0	1	0	0	0	0	0	1	0	0	0	0	0	0	1	0	0	1	1	42.40241667	18.77496667
873	2008/118	1	0	0	0	0	1	0	0	0	1	0	0	1	0	0	0	1	0	1	0	0	0	0	0	0	0	0	0	0	0	0	0	0	0	0	0	0	0	0	0	0	0	0	0	0	0	0	1	42.40036667	18.79401667
874	2008/119	0	0	0	0	0	0	0	0	0	0	0	0	1	0	0	0	0	0	0	0	0	0	0	0	0	0	0	0	1	0	0	0	0	0	0	0	0	0	0	0	0	0	0	0	0	0	0	3	42.3999	18.83136667
875	2008/120	1	0	0	0	0	1	0	0	0	0	0	0	0	0	0	0	0	0	0	0	0	0	0	0	0	0	0	0	0	0	0	0	0	0	0	0	0	0	0	0	0	0	0	0	0	0	0	2	42.3989	18.83808333
876	2008/120A	0	0	0	0	0	0	0	0	0	0	0	0	0	0	0	0	0	0	0	0	0	0	0	0	0	0	0	0	0	0	0	0	0	0	0	0	0	0	0	0	0	0	0	0	0	0	0	1	42.39963333	18.83871667
877	2008/121	1	0	0	0	0	0	0	0	0	0	0	0	0	0	0	0	0	0	0	0	0	0	0	0	0	0	0	0	0	0	0	0	0	0	0	0	0	0	0	0	0	0	0	0	0	0	0	2	42.39503333	18.80905
880	2008/124	0	0	0	0	0	0	0	0	0	0	0	0	0	0	0	0	0	0	0	0	0	0	0	0	0	0	0	0	0	0	1	0	0	0	0	0	0	0	0	0	0	0	0	0	0	0	0	1	42.39938333	18.78271667
882	2008/126	0	0	0	0	1	0	0	0	0	0	0	0	0	0	1	0	0	0	0	0	0	0	0	0	0	0	0	0	0	0	0	0	0	0	0	0	0	0	0	0	0	0	0	1	0	0	0	3	42.60271667	18.67483333
883	2008/127	0	0	0	0	0	0	0	0	1	0	0	0	0	0	0	0	0	0	0	0	0	0	0	0	0	0	0	0	0	0	0	0	0	0	0	0	0	0	0	0	0	0	0	0	0	0	0	1	42.65231667	18.67201667
884	2008/128	0	0	0	0	0	1	0	0	0	0	0	0	0	0	0	0	0	0	0	0	0	0	0	0	0	0	0	0	0	0	0	0	0	0	0	0	1	0	0	0	0	0	0	0	0	0	0	3	42.67763333	18.64191667
885	2008/129	1	0	0	0	0	0	0	0	1	0	0	0	1	0	1	0	0	0	0	0	0	0	0	0	0	0	0	0	0	0	0	0	0	0	0	0	1	0	0	0	0	0	0	0	0	0	0	3	42.71938333	18.60241667
886	2008/130	1	0	0	0	0	0	0	0	0	0	0	0	1	0	0	0	0	0	1	0	0	0	0	0	0	0	0	0	0	0	0	0	0	0	0	0	0	0	0	0	0	0	0	0	0	0	0	1	42.76236667	18.82618333
888	2008/132	0	0	0	0	0	0	0	0	0	0	0	0	1	0	1	0	0	0	0	0	0	0	0	0	0	0	0	0	0	0	0	0	0	0	0	0	1	0	0	0	0	0	0	0	0	0	0	2	42.85776667	18.94326667
889	2008/133	0	0	0	0	0	0	0	0	1	1	0	0	1	0	0	0	0	0	0	0	0	0	1	0	0	0	0	0	1	0	0	0	0	0	0	1	0	0	0	0	0	0	0	0	0	0	0	3	42.9017	19.02098333
890	2008/134	1	0	0	0	0	1	0	0	0	1	0	0	1	0	0	0	0	0	1	0	1	0	0	0	0	1	0	0	0	1	0	0	0	0	0	0	1	0	1	0	0	0	0	0	0	0	0	1	42.89493333	19.07943333
891	2008/135	1	0	0	0	0	0	0	0	0	0	0	0	0	0	0	1	0	0	1	0	0	0	0	0	0	0	0	0	0	0	0	0	0	0	0	1	1	0	0	0	0	0	0	0	0	0	0	1	42.94763333	19.09105
892	2008/136	0	0	0	0	0	0	0	0	1	0	0	0	1	0	1	0	0	0	0	0	0	0	0	0	0	0	0	1	1	1	0	0	0	0	0	0	0	0	1	0	0	0	0	0	0	0	0	1	42.94975	19.0993
893	2008/137	0	0	0	0	0	1	0	0	0	0	0	0	0	0	1	0	0	0	1	0	0	0	0	0	0	0	0	0	0	0	0	0	0	0	0	0	1	0	0	0	0	0	0	0	0	0	0	1	42.9622	19.09308333
894	2008/138	0	0	0	0	0	1	0	0	0	0	0	0	1	0	1	0	0	0	1	0	0	0	0	0	0	0	0	0	0	1	0	0	0	0	0	0	0	0	1	0	0	0	0	0	0	0	0	1	42.95765	19.0959
895	2008/139	0	0	0	0	0	0	0	0	0	0	0	0	0	0	0	0	0	0	0	0	0	0	0	0	0	0	0	0	0	0	0	0	0	0	0	0	0	0	0	0	0	0	0	0	0	0	0	2	42.98756667	19.0668
896	2008/140	0	0	0	0	0	0	0	0	1	0	0	0	1	0	1	0	0	0	1	0	0	0	0	0	0	0	0	0	1	1	0	0	0	0	0	0	1	0	1	0	0	0	0	0	0	0	0	1	42.9883	19.06783333
897	2008/141	1	0	0	0	0	0	0	0	0	0	0	0	1	0	1	0	0	0	1	0	0	0	0	0	0	0	0	0	0	0	0	0	0	0	0	0	1	0	1	0	0	0	0	0	0	0	0	1	42.98156667	19.06833333
898	2008/142	1	0	0	0	0	0	0	0	0	0	0	0	1	0	0	0	0	0	0	0	0	0	0	0	0	0	0	0	1	0	0	0	0	0	0	0	0	0	1	0	0	0	0	0	0	0	0	1	42.98718333	19.07993333
899	2008/143	1	0	0	0	0	1	0	0	1	1	0	0	1	0	0	0	0	0	1	0	0	0	0	0	0	0	0	0	0	0	0	0	0	0	0	0	0	0	0	0	0	0	0	0	0	0	0	1	42.9507	19.1735
903	2008/147	0	0	0	0	0	0	0	0	0	0	0	0	1	0	1	0	0	0	1	0	0	0	0	0	0	0	0	1	0	0	0	0	0	0	0	0	1	0	1	0	0	0	0	0	0	0	0	1	42.90093333	19.27321667
904	2008/148	0	0	0	0	0	0	0	0	0	0	0	0	0	0	0	0	0	0	0	0	0	0	0	0	0	0	0	0	0	0	0	0	0	0	0	1	1	0	0	0	0	0	0	0	0	0	0	1	42.88223333	19.29768333
905	2008/149	1	0	0	0	0	1	0	0	0	0	0	0	0	0	0	0	0	0	0	0	0	0	0	0	0	0	0	0	0	0	0	0	0	0	0	0	1	0	0	0	0	0	0	0	0	0	0	1	42.84868333	19.33448333
906	2008/149A	0	0	0	0	0	0	0	0	0	0	0	0	0	0	0	0	0	0	0	0	0	0	0	0	0	0	0	0	0	0	0	0	0	0	0	0	0	0	0	0	0	0	0	0	0	0	0	1	42.81903333	19.38101667
908	2008/151	0	0	0	0	0	0	0	0	0	0	0	0	1	0	1	1	0	0	0	0	0	0	0	0	0	0	0	0	0	0	0	0	0	0	0	0	0	0	1	0	0	0	0	0	0	0	0	1	42.88206667	19.39978333
909	2008/152	1	0	0	0	0	1	0	0	0	0	0	0	1	0	1	0	0	0	1	0	0	0	0	0	0	0	0	0	1	0	0	0	0	0	0	0	0	0	1	0	0	0	0	0	0	0	0	1	42.90301667	19.38221667
910	2008/153	1	0	0	0	0	1	0	0	1	0	0	0	0	0	1	1	1	0	0	0	0	0	1	0	0	0	0	0	0	0	0	0	0	0	0	0	0	0	1	0	0	0	0	0	0	0	0	1	42.89715	19.38566667
911	2008/154	0	0	0	0	0	0	0	0	0	0	0	0	0	0	0	0	0	0	0	0	0	0	0	0	0	0	0	0	0	0	0	0	0	0	0	0	0	0	0	0	0	0	0	0	0	0	0	3	42.89151667	19.38918333
912	2008/155	0	0	0	0	0	1	0	0	0	1	0	0	1	0	0	0	0	0	0	0	0	0	0	0	1	0	0	0	0	0	0	0	0	0	0	1	0	0	1	0	0	0	0	0	0	0	0	1	42.8722	19.47948333
915	2008/158	0	0	0	0	0	0	0	0	1	0	0	0	0	0	0	0	0	1	0	0	0	0	0	0	0	0	0	0	0	0	0	0	0	0	0	1	0	0	0	0	0	0	0	0	0	0	0	2	42.89495	19.64246667
916	2008/159	0	0	0	0	0	0	0	0	0	0	0	0	0	0	0	0	0	0	0	0	0	0	0	0	0	0	0	0	0	0	0	0	0	0	0	0	0	0	0	0	0	0	0	0	0	0	0	2	42.88933333	19.65971667
917	2008/160	0	0	0	0	0	0	0	0	0	0	0	0	1	0	0	0	0	0	0	0	1	0	0	0	0	0	0	0	0	0	0	0	0	0	0	0	0	0	0	0	0	0	0	0	0	0	0	1	42.87968333	19.74111667
919	2008/162	1	0	0	0	0	1	0	0	1	1	0	0	1	1	1	0	1	0	1	0	1	0	0	0	1	1	0	0	0	1	0	0	0	0	0	1	0	0	0	0	0	0	0	0	0	0	0	1	42.51173333	19.8348
921	2008/163R	0	0	0	0	0	0	0	0	0	0	0	0	1	1	0	0	0	1	1	0	0	0	0	0	0	0	0	0	0	1	0	0	0	0	0	1	1	0	1	0	0	0	0	0	0	0	0	1	42.4907	19.81885
922	2008/164	0	0	0	0	0	0	0	0	0	0	0	0	0	0	0	0	0	0	0	0	0	0	0	0	0	0	0	0	0	0	0	0	0	0	0	0	0	0	1	0	0	0	0	0	0	0	0	1	42.49095	19.81231667
923	2008/165	0	0	0	0	1	0	0	0	0	1	0	0	0	0	0	1	0	0	1	0	0	0	0	0	0	0	0	0	0	0	0	0	0	0	0	0	1	0	0	0	0	0	0	0	0	0	0	2	42.50163333	19.82456667
924	2008/166	1	0	0	0	0	1	0	0	1	0	0	0	1	0	1	0	0	0	0	0	0	0	0	0	0	0	0	0	0	1	0	0	0	0	0	0	0	0	0	0	0	0	0	0	0	0	0	1	42.6337	19.85008333
929	2008/171	0	0	0	0	0	1	0	0	1	0	0	0	1	0	1	0	0	0	1	0	1	0	0	0	0	0	0	0	1	1	0	0	0	0	0	0	0	0	1	0	0	0	0	0	0	0	0	1	42.67545	19.52291667
930	2008/172	1	0	0	0	0	1	0	0	0	0	0	0	1	0	0	0	0	0	0	0	0	0	0	0	0	0	0	0	1	1	0	0	0	0	0	0	1	0	1	0	0	0	0	0	0	0	0	1	42.61888333	19.55368333
932	2008/174	0	0	0	0	1	0	0	0	0	0	0	0	1	0	1	0	0	0	0	0	0	0	0	0	0	0	0	0	0	1	0	0	0	0	0	0	1	0	1	0	0	0	0	0	0	0	0	1	42.56941667	19.6025
933	2008/175	0	0	0	0	0	0	0	0	0	0	0	0	0	0	0	0	0	0	0	0	0	0	0	0	0	0	0	0	0	0	0	0	0	0	0	0	0	0	0	0	0	0	0	0	0	0	0	3	42.57495	19.59783333
934	2008/176	0	0	0	0	0	1	0	0	0	0	0	0	1	0	1	0	0	0	0	0	1	0	0	0	0	0	0	0	1	1	0	0	0	0	0	0	1	0	1	0	0	0	0	0	0	0	0	1	42.57885	19.59216667
935	2008/177	0	0	0	0	0	1	0	0	0	0	0	0	0	0	1	0	0	0	0	0	0	0	0	0	0	0	0	0	0	1	0	0	0	0	0	0	1	0	0	0	0	0	0	0	0	0	0	1	42.59308333	19.5801
936	2008/178	1	0	0	0	0	0	0	0	0	0	0	0	0	0	0	0	0	0	0	0	1	0	0	0	0	0	0	1	0	0	0	0	0	0	0	0	1	0	0	0	0	0	0	0	0	0	0	1	42.59936667	19.51166667
937	2008/179	1	0	0	0	0	1	0	0	0	0	0	0	1	0	0	0	0	0	0	0	0	0	0	0	0	0	0	0	1	0	0	0	0	0	0	0	0	0	0	0	0	0	0	0	0	0	0	1	42.54876667	19.45435
938	2008/180	1	0	0	0	0	1	0	0	0	0	0	0	0	0	0	0	0	0	0	0	0	0	0	0	0	0	0	0	1	0	0	0	0	0	0	0	1	0	0	0	0	0	0	0	0	0	0	1	42.51081667	19.38476667
939	2008/181	1	0	0	0	0	1	0	0	0	0	0	0	0	0	0	0	0	0	0	0	0	0	0	0	0	0	0	0	0	0	0	0	0	0	0	0	1	0	0	0	0	0	0	0	0	0	0	1	42.45411667	19.35115
940	2008/182	1	0	1	0	0	1	0	0	1	1	0	0	0	0	0	0	1	1	0	0	1	0	0	1	1	0	0	0	0	0	0	0	0	0	0	0	1	0	0	0	0	0	0	0	0	0	1	1	42.1924	19.107
941	2008/183	0	0	0	0	0	0	0	0	0	0	0	0	1	0	1	0	0	0	0	0	0	0	0	0	0	0	0	1	0	0	0	0	0	0	0	0	0	0	0	0	0	0	0	0	0	0	1	1	42.09386667	19.13423333
942	2008/184	0	0	0	0	0	0	0	0	0	0	0	0	0	0	0	0	0	0	1	0	0	0	0	0	0	0	0	0	0	0	0	0	0	0	0	0	1	0	0	0	0	1	0	1	0	0	1	1	42.10043333	19.1419
943	2008/185	0	0	0	0	0	0	0	0	0	0	0	0	0	0	0	0	0	0	0	0	0	0	0	0	0	0	0	0	0	0	0	0	0	0	0	0	0	0	0	0	0	0	0	0	0	0	1	1	42.09726667	19.14583333
944	2008/185B	1	0	0	0	0	1	0	0	0	0	0	0	0	0	1	0	0	0	1	0	0	0	0	0	0	0	0	0	0	0	0	0	0	0	0	0	0	0	0	0	0	1	0	1	0	0	1	1	42.0978	19.14466667
945	2008/185C	0	0	0	0	0	0	0	0	0	0	0	0	0	0	0	0	0	0	0	0	0	0	0	0	0	0	0	0	0	0	0	0	0	0	0	0	0	0	0	0	0	0	0	0	0	0	1	1	42.09965	19.14263333
946	2008/186	1	0	0	0	0	1	0	0	0	0	0	0	0	0	0	0	0	0	0	0	0	0	0	0	0	0	0	0	0	1	0	0	0	0	0	0	1	0	0	0	0	0	0	0	0	0	1	1	42.21876667	19.0246
948	2008/188	0	0	0	0	0	0	0	0	1	1	0	0	1	0	1	0	0	0	1	0	0	0	0	0	0	0	0	0	0	1	0	0	0	0	0	0	0	0	1	0	0	0	0	0	0	0	0	1	43.07073333	19.86145
951	0	1	0	0	0	0	1	0	0	0	0	0	0	0	0	0	0	0	0	0	0	0	0	0	0	0	0	0	0	0	0	0	0	0	0	0	0	0	0	0	0	0	0	0	0	0	0	0	1	41.69387	19.821554
952	0	1	0	0	0	0	0	0	0	0	0	0	0	0	0	0	0	0	0	0	0	0	0	0	0	0	0	0	1	0	0	0	0	0	0	0	0	0	0	0	0	0	0	0	1	0	0	0	1	41.63089	19.73321
954	0	1	0	0	0	0	0	0	0	0	0	0	0	0	1	0	0	0	0	0	0	0	0	0	0	0	0	0	0	0	0	0	0	0	0	0	0	0	0	0	0	0	0	0	1	0	0	0	1	40.16149	19.65018
955	0	1	0	0	0	0	0	0	0	1	0	0	0	0	0	1	0	0	0	0	0	0	0	0	0	0	0	0	0	0	0	0	0	0	0	0	0	0	0	0	0	0	0	0	1	0	0	0	1	39.77261	20.07333
956	0	1	0	0	0	0	0	0	0	0	0	0	0	0	0	0	0	0	0	0	0	0	0	0	0	0	0	0	0	0	0	0	0	0	0	0	0	0	0	0	0	0	0	0	1	1	0	0	1	39.82265	20.06533
957	0	0	0	0	0	0	0	0	0	0	0	0	0	0	0	0	0	0	0	0	0	0	0	0	0	0	0	0	0	0	0	0	0	0	0	0	0	0	0	0	0	0	0	0	1	0	0	0	1	39.75738	20.08182
959	0	0	0	0	0	0	0	0	0	0	0	0	0	0	0	0	0	0	0	0	0	0	0	0	0	0	0	0	0	0	0	0	0	0	0	0	0	1	0	0	0	0	0	0	0	0	0	0	1	42.34298	20.03205
960	0	1	0	0	0	0	0	0	0	0	0	0	0	0	0	1	0	0	0	1	0	0	0	0	0	0	0	0	0	0	0	0	0	0	0	0	0	0	0	0	0	0	0	0	0	0	0	0	1	42.36478	20.01736
961	0	1	0	0	0	0	0	0	0	0	0	0	0	0	0	0	0	0	0	0	0	0	0	0	0	0	0	0	0	0	0	0	0	0	0	0	0	0	0	0	0	0	0	0	0	0	0	0	2	42.34867	19.99474
962	0	1	0	0	0	0	0	0	0	0	0	0	0	0	0	0	0	0	0	0	0	0	0	0	0	0	0	0	0	0	0	0	0	0	0	0	0	0	0	0	0	0	0	0	0	0	0	0	1	42.35199	19.99302
963	0	0	0	0	0	0	0	0	0	0	0	0	0	0	0	0	0	0	0	0	0	0	0	0	0	0	0	0	0	0	1	0	0	0	0	0	1	0	0	0	0	0	0	0	0	0	0	0	1	42.46327	19.88637
964	0	0	0	0	0	0	0	0	0	0	0	0	0	0	0	1	0	0	0	0	0	1	0	0	0	0	0	0	0	0	0	0	0	0	0	0	0	0	0	1	0	0	0	0	0	0	0	0	2	42.44961	20.13148
965	0	1	0	0	0	0	0	0	0	0	1	0	0	0	0	0	0	0	0	0	0	0	0	0	0	0	0	0	0	0	0	0	0	0	0	0	0	0	0	0	0	0	0	0	0	0	0	0	2	42.46322	20.12577
966	0	0	0	0	0	0	0	0	0	0	0	0	0	0	0	0	0	0	0	0	0	0	0	0	0	0	0	0	0	0	0	0	0	0	0	0	0	0	0	1	0	0	0	0	0	0	0	0	2	42.4678	20.12229
969	2009/123	0	0	0	0	0	0	0	0	0	0	0	0	0	0	1	0	0	0	1	0	0	0	0	0	0	0	0	0	0	1	0	0	0	0	0	0	0	0	0	0	0	0	0	0	0	0	0	1	43.8713	20.11421667
970	2009/124	0	0	0	0	0	0	0	0	0	0	0	0	1	0	1	0	0	0	0	0	0	0	0	0	0	0	0	0	0	0	0	0	0	0	0	0	0	0	1	0	0	0	0	0	0	0	0	1	43.90175	20.19268333
974	2009/128	0	0	0	0	0	0	0	0	0	1	0	0	0	0	0	1	0	0	1	0	0	0	0	0	0	0	0	0	0	1	0	0	0	0	0	1	0	0	0	0	0	0	0	0	0	0	0	1	43.72348333	21.9478
975	2009/129	0	0	0	0	0	1	0	0	1	0	0	0	1	0	1	1	0	0	1	0	1	0	0	0	0	0	0	0	0	1	0	0	0	0	0	0	0	0	0	0	0	0	0	0	0	0	0	1	43.7957	21.90348333
977	0	1	0	0	0	0	0	0	0	0	0	0	0	0	0	0	0	0	0	1	0	0	0	0	0	0	0	0	0	0	0	0	0	0	0	0	0	0	0	0	0	0	0	0	0	0	0	1	1	41.37773	20.03291
978	0	0	0	0	0	0	0	0	0	0	0	0	0	0	0	0	0	0	0	0	0	0	0	0	0	0	0	0	0	0	0	0	0	0	0	0	0	0	0	0	0	0	0	0	0	0	0	1	2	41.68776	20.15676
979	0	0	0	0	0	0	0	0	0	0	0	0	0	0	0	0	0	0	0	0	0	0	0	0	0	0	0	0	0	0	0	0	0	0	0	0	0	0	0	0	0	0	0	0	0	0	0	1	3	41.68861	20.15571
980	0	0	0	0	0	0	0	0	0	0	0	0	0	0	0	0	0	0	0	0	0	0	0	0	0	0	0	0	0	0	0	0	0	0	0	0	0	0	0	0	0	0	0	0	0	0	0	1	1	41.69076	20.14681
981	0	0	0	0	0	0	0	0	0	0	0	0	0	0	0	0	0	0	0	0	0	0	0	0	0	0	0	0	0	0	0	0	0	0	0	0	0	0	0	0	0	0	0	0	0	0	0	1	1	41.69025	20.16586
982	0	1	0	0	0	0	0	0	0	0	0	0	0	0	0	0	0	0	0	1	0	0	0	0	0	0	0	0	0	0	0	0	0	0	0	0	0	0	0	0	0	0	0	0	0	0	0	0	1	40.18113	20.66172
983	0	1	0	0	0	0	0	0	0	0	0	0	0	0	0	1	0	0	0	0	0	0	0	0	0	0	0	0	0	0	0	0	0	0	0	0	0	0	0	0	0	0	0	0	1	0	0	0	1	39.86484	20.02188
984	0	1	0	0	0	0	0	0	0	0	0	0	0	0	0	0	0	0	0	0	0	0	0	0	0	0	0	0	0	0	0	0	0	0	0	0	0	0	0	0	0	0	0	0	1	0	0	0	1	39.68221	20.12899
985	0	1	0	0	0	0	0	0	0	0	0	0	0	1	0	0	0	0	0	0	0	0	0	0	0	0	0	0	0	0	0	0	0	0	0	0	0	0	0	0	0	0	0	0	1	0	0	0	1	40.129	19.67311
986	0	1	0	0	0	0	0	0	0	0	0	0	0	0	0	0	0	0	0	0	0	0	0	0	0	0	0	0	0	0	0	0	0	0	0	0	0	0	0	0	0	0	0	0	0	0	0	0	1	42.29318	19.53328
996	2010/009	0	0	0	0	0	1	0	0	1	0	0	0	0	1	1	0	0	0	1	0	0	0	0	0	0	0	0	1	1	0	0	0	0	0	0	0	0	0	0	0	0	0	0	0	1	1	1	1	41.62558333	20.24983333
997	2010/010	1	0	0	0	0	1	0	0	1	0	0	0	1	0	1	0	1	0	1	0	0	0	1	0	0	1	0	1	0	0	0	0	0	0	0	0	0	0	0	0	0	0	0	0	1	0	2	1	41.64651667	20.19013333
998	2010/011	0	0	0	0	0	0	0	0	0	0	0	0	0	0	0	0	0	0	1	0	0	0	0	0	0	0	0	0	0	0	0	0	0	0	0	0	0	0	0	0	0	0	0	0	1	1	0	1	41.66373333	20.19533333
999	2010/012	0	0	0	0	0	0	0	0	0	0	0	0	0	0	0	0	0	0	1	0	0	0	0	0	0	0	0	0	1	0	0	0	0	0	0	0	0	0	0	0	0	0	0	0	1	0	1	1	41.66508333	20.20828333
1001	2010/013B	1	0	0	0	0	0	0	0	0	0	0	0	1	1	0	0	0	0	0	0	0	0	0	0	0	0	0	0	0	0	0	0	0	0	0	1	0	0	0	0	0	0	0	0	1	0	2	1	41.67465	20.21115
1003	2010/BP1	0	0	0	0	0	0	0	0	0	0	0	0	0	0	0	0	0	0	0	0	0	0	0	0	0	0	0	0	0	0	0	0	0	0	0	0	0	0	0	0	0	0	0	0	0	0	0	2	41.67671	20.2135
1004	2010/015	1	0	0	0	0	0	0	0	0	0	0	0	1	1	0	0	0	0	0	0	0	0	0	0	0	0	0	0	0	0	0	0	0	0	0	0	0	0	0	0	0	0	0	0	1	0	1	1	41.69623333	20.13331667
1005	2010/016	1	0	0	0	0	1	0	0	0	1	0	0	1	1	0	1	0	0	1	0	0	0	0	0	0	1	0	0	0	0	0	0	0	0	0	0	0	0	0	0	0	0	0	0	1	0	1	1	41.69708333	20.13618333

1008	2010/BP2	0	0	0	0	0	0	0	0	0	0	0	0	0	0	0	0	0	0	0	0	0	0	0	0	0	0	0	0	0	0	0	0	0	0	0	0	0	0	0	0	0	0	0	0	0	0	0	0	0	1	1	41.69194	20.21288
1009	2010/BP4	0	0	0	0	0	0	0	0	0	0	0	0	0	0	0	0	0	0	0	0	0	0	0	0	0	0	0	0	0	0	0	0	0	0	0	0	0	0	0	0	0	0	0	0	0	0	0	0	0	1	1	41.68977	20.25569
1010	2010/BP5	0	0	0	0	0	0	0	0	0	0	0	0	0	0	0	0	0	0	0	0	0	0	0	0	0	0	0	0	0	0	0	0	0	0	0	0	0	0	0	0	0	0	0	0	0	0	0	0	0	1	1	41.69413	20.2618
1012	2010/020	1	0	0	0	0	0	0	0	0	1	0	0	0	0	0	0	0	0	1	0	0	0	1	0	0	1	0	0	1	0	0	0	0	0	0	0	0	0	0	0	0	0	0	0	0	0	0	0	0	0	1	41.81346667	20.13973333
1024	2010/032	1	0	0	0	0	1	0	0	0	0	0	0	0	0	0	0	0	0	1	0	1	0	0	0	0	0	0	0	1	0	0	1	0	0	0	0	0	0	0	0	0	0	0	0	0	0	0	0	0	0	1	41.84231667	20.09451667
1025	2010/033	1	0	0	0	0	0	0	0	0	1	0	0	1	0	1	1	1	1	1	0	0	0	1	0	0	1	0	0	1	0	1	0	0	0	0	0	0	0	0	0	0	0	0	0	0	0	0	0	0	0	1	41.85056667	20.09736667
1026	2010/034	1	0	0	0	0	1	0	0	0	0	0	0	1	0	0	0	0	0	0	0	1	0	0	0	0	0	0	0	0	0	0	0	0	0	0	0	0	0	0	0	0	0	0	0	0	0	0	1	0	0	1	41.85456667	20.1217
1028	2010/036	1	0	0	0	0	0	0	0	0	0	0	0	0	0	0	0	0	0	1	0	0	0	0	0	0	0	0	0	0	0	0	0	0	0	0	0	0	0	0	0	0	0	0	0	0	0	0	0	0	0	1	41.86923333	20.11863333
1029	2010/037	0	0	0	0	0	0	0	0	0	0	0	0	1	0	0	0	0	0	0	0	0	0	0	0	0	0	0	0	0	0	0	0	0	0	0	0	0	0	0	0	0	0	0	0	0	0	0	0	0	0	1	41.87066667	20.1085
1030	2010/038	0	0	0	0	0	0	0	0	0	0	0	0	0	0	0	0	0	0	0	0	0	0	0	0	0	0	0	0	0	0	0	0	0	0	0	0	0	0	0	0	0	0	0	0	0	0	0	0	0	0	1	42.13848333	20.49005
1031	2010/039	0	0	0	0	0	0	0	0	0	0	0	0	0	1	0	0	0	0	0	0	0	0	0	0	0	0	0	0	0	0	0	0	0	0	0	0	0	0	0	0	0	0	0	0	0	0	0	0	0	0	3	42.19235	20.53763333
1032	2010/040	0	0	0	0	0	0	0	0	0	0	0	0	0	0	0	0	0	0	0	0	1	0	0	0	0	0	0	0	0	0	0	0	0	0	0	0	0	0	0	0	0	0	0	0	0	0	0	0	0	0	3	42.20076667	20.5333
1033	2010/041	1	0	0	0	0	0	0	0	0	1	0	0	0	0	1	0	0	0	0	0	0	0	0	0	0	0	0	0	0	0	0	0	0	0	0	0	0	0	0	0	0	0	0	0	0	0	0	1	0	0	3	42.20285	20.53378333
1034	2010/042	1	0	0	0	0	1	0	0	0	1	0	0	1	0	1	0	0	0	1	0	1	0	0	0	0	0	0	0	0	0	0	0	0	0	0	0	0	0	0	0	0	0	0	0	0	0	0	1	0	0	2	42.20695	20.52848333
1035	2010/043	1	0	0	0	0	0	0	0	0	0	0	0	1	0	1	0	0	0	0	0	0	0	1	0	0	1	0	0	0	0	1	0	0	0	0	0	0	0	0	1	0	0	0	0	0	0	1	0	0	0	1	42.11456667	19.57471667
1036	2010/044	1	0	0	0	0	0	0	0	0	0	0	0	0	0	0	0	0	0	0	0	0	0	0	0	0	0	0	0	0	0	0	0	0	0	0	0	0	0	0	0	0	0	0	0	0	0	0	0	0	0	1	42.12485	19.6037
1037	2010/045	1	0	0	0	0	0	0	0	0	0	0	0	0	0	0	0	0	0	0	0	0	0	0	0	0	0	0	0	0	0	0	0	0	0	0	0	0	0	0	0	0	0	0	0	0	0	0	0	0	0	1	42.14171667	19.64743333
1038	2010/046	1	0	0	0	0	0	0	0	0	0	0	0	0	0	0	0	0	0	0	0	0	0	0	0	0	0	0	0	0	0	0	0	0	0	0	0	0	0	0	1	0	0	0	0	0	0	0	0	0	0	1	42.16498333	19.69515
1039	2010/047	1	0	0	0	0	0	0	0	0	0	0	0	0	0	0	0	0	0	0	0	0	0	0	0	0	0	0	0	0	0	0	0	0	0	0	0	0	0	0	0	0	0	0	0	0	0	0	0	0	0	1	42.17863333	19.72008333
1040	2010/048	1	0	0	0	0	0	0	0	0	0	0	0	0	0	0	0	0	0	0	0	0	0	0	0	0	0	0	0	0	0	0	0	0	0	0	0	0	0	0	0	0	0	0	0	0	0	0	0	0	0	1	42.207	19.70906667
1041	2010/049	1	0	0	0	0	0	0	0	0	0	0	0	0	0	0	0	0	0	0	0	0	0	0	0	0	0	0	0	0	0	0	0	0	0	0	0	0	0	0	0	0	0	0	0	0	0	0	0	0	0	1	42.21423333	19.70581667
1042	2010/050	0	0	0	0	0	0	0	0	0	0	0	0	0	0	0	0	0	0	0	0	0	0	0	0	0	0	0	0	0	0	0	0	0	0	0	0	0	0	0	1	0	0	0	0	0	0	0	0	0	0	1	42.24528333	19.71691667
1043	2010/051	0	0	0	0	0	1	0	0	0	0	0	0	0	0	0	0	0	0	0	0	0	0	0	0	0	0	0	0	0	0	0	0	0	0	0	0	0	0	0	0	0	0	0	0	0	0	0	0	0	0	1	42.24036667	19.72803333
1045	2010/053	0	0	0	0	0	0	0	0	0	0	0	0	1	0	0	0	0	0	1	0	0	0	0	0	0	0	0	0	0	0	0	0	0	1	0	0	0	0	0	0	0	0	0	0	0	0	0	0	0	0	1	42.25136667	19.74788333
1046	2010/054	1	0	0	0	0	0	0	0	0	0	0	0	0	0	0	0	0	0	0	0	0	0	0	0	0	0	0	0	0	0	0	0	0	0	0	0	0	0	0	1	0	0	0	0	0	0	0	0	0	0	1	42.28976667	19.79683333
1047	2010/055	0	1	0	0	0	0	0	0	1	0	0	0	1	0	1	0	1	0	0	1	0	1	1	0	1	1	0	1	1	0	0	0	0	0	0	0	0	0	0	0	0	0	0	0	0	0	0	0	0	0	1	42.3043	19.7969
1048	2010/056	0	0	0	0	0	0	0	0	0	0	0	0	0	0	0	0	0	0	0	0	0	0	0	0	0	0	0	0	0	0	0	0	0	0	0	0	0	0	0	0	0	0	0	0	0	0	0	0	0	0	3	42.40128333	19.7658
1049	2010/BP8	0	0	0	0	0	0	0	0	0	0	0	0	0	0	0	0	0	0	0	0	0	0	0	0	0	0	0	0	0	0	0	0	0	0	0	0	0	0	0	0	0	1	0	0	0	0	0	0	0	0	3	41.99294	20.4267
1050	2010/BP9	0	0	0	0	0	0	0	0	0	0	0	0	0	0	0	0	0	0	0	0	0	0	0	0	0	0	0	0	0	0	0	0	0	0	0	0	0	0	0	0	0	1	0	0	0	0	0	0	0	0	2	42.00798333	20.44608333
1051	2010/057	0	0	0	0	0	0	0	0	0	0	0	0	0	0	0	0	0	0	0	0	0	0	0	0	0	0	0	0	0	0	0	0	0	0	0	0	0	0	0	0	0	0	0	0	0	0	0	0	0	0	3	42.40161667	19.7577
1055	2010/061	1	0	0	0	0	1	0	0	1	0	1	0	1	1	1	1	0	0	1	0	0	0	0	0	0	0	0	0	0	0	0	0	1	0	0	0	0	0	0	0	0	1	0	0	0	0	0	0	0	0	1	43.21303333	19.76305
1056	2010/062	1	0	0	0	0	1	0	0	0	0	0	0	0	0	0	0	0	0	1	0	0	0	0	0	0	0	0	0	0	0	0	0	0	0	0	0	0	0	0	0	0	0	0	0	0	0	0	0	0	0	1	43.61168333	19.72516667
1057	2010/BP12	0	0	0	0	0	0	0	0	0	0	0	0	0	0	0	0	0	0	1	0	0	0	0	0	0	0	0	0	0	0	0	0	0	0	0	0	0	0	0	0	0	0	0	0	0	0	0	0	0	0	1	41.97755	20.11537
1058	0	0	0	0	0	0	0	0	0	0	0	0	0	1	0	0	0	0	0	0	0	0	0	0	0	0	0	0	0	0	0	0	0	0	0	0	0	0	0	0	0	0	0	0	0	0	0	0	0	0	0	1	42.445683	19.756067
1059	0	0	0	0	0	0	0	0	0	0	0	0	0	1	0	0	0	0	0	0	0	0	0	0	0	0	0	0	0	0	0	0	1	0	0	0	0	0	0	0	0	0	0	0	0	0	0	0	0	0	0	1	42.419	19.64347
1060	0	0	0	0	0	0	0	0	0	0	0	0	0	0	0	0	0	0	0	0	0	0	0	0	0	0	0	0	0	0	0	1	0	0	0	0	0	0	0	0	0	0	0	0	0	0	0	0	0	0	0	1	42.42166	19.67168
1061	0	0	0	0	0	0	0	0	0	0	0	0	0	0	0	0	0	0	0	0	0	0	0	0	0	0	0	0	0	0	0	0	0	0	0	0	0	0	0	0	0	0	1	0	0	0	0	0	0	0	0	1	42.43077	19.69267
1062	0	0	0	0	0	0	0	0	0	0	0	0	0	0	0	0	0	0	0	1	0	0	0	0	0	0	0	0	0	0	0	0	0	0	0	0	0	0	0	0	0	0	0	0	0	0	0	0	0	0	0	1	42.45781	19.71515
1063	0	0	0	0	0	0	0	0	0	0	0	0	0	0	0	0	0	0	0	0	0	1	0	0	0	0	0	0	0	0	0	0	0	0	0	0	0	0	0	0	0	0	0	0	0	0	0	0	0	0	0	1	42.45572	19.77465
1064	0	0	0	0	0	0	0	0	0	0	0	0	0	0	0	0	0	0	0	0	0	0	0	0	0	0	0	0	0	0	0	0	0	0	0	0	0	0	0	0	0	0	1	0	0	0	0	0	0	0	0	1	42.46108	19.71596
1065	0	0	0	0	0	0	0	0	0	0	0	0	0	0	1	0	0	0	0	0	0	0	0	0	0	0	0	0	0	0	0	0	0	0	0	1	0	0	0	0	0	0	0	0	0	0	0	0	0	0	1	2	39.70188333	20.89465
1066	0	0	0	0	0	0	1	0	0	0	0	0	0	0	0	0	0	0	0	1	0	0	0	0	0	0	0	0	0	0	0	0	0	0	0	0	0	0	0	0	0	0	0	0	0	0	0	0	0	0	0	2	39.72825	21.0839
1068	0	0	0	0	0	0	0	0	0	0	0	0	0	0	0	0	0	0	0	0	0	0	0	0	0	0	0	0	0	0	0	0	0	0	0	1	0	0	0	0	0	0	0	0	0	0	0	0	0	0	0	1	39.177167	20.734383
1069	0	0	1	0	0	0	0	0	0	0	0	0	0	0	0	0	0	0	0	0	0	0	0	0	0	0	0	0	0	0	0	0	0	0	0	1	0	0	0	0	0	0	0	0	0	0	0	0	0	0	0	1	38.722717	20.690067
1071	0	0	0	0	0	0	0	0	0	0	0	0	0	0	0	0	0	0	0	1	0	0	0	0	0	0	0	0	0	0	0	0	0	0	0	0	0	0	0	0	0	0	0	0	0	0	0	0	0	0	1	1	41.31676667	20.3774
1072	0	0	1	0	0	0	0	0	0	0	0	0	0	0	0	0	0	0	0	1	0	0	0	1	0	0	1	0	0	0	0	0	0	0	1	1	0	0	0	0	0	0	0	0	0	0	0	0	0	0	0	1	40.8665	20.377333
1073	0	0	1	0	0	0	1	0	0	0	0	0	0	0	0	0	0	0	0	0	0	0	0	0	0	0	0	0	0	0	0	0	0	0	0	1	0	0	0	0	0	0	0	0	0	0	0	0	0	0	0	1	40.08	19.954
1076	2012/103	0	0	0	0	0	0	0	0	0	0	0	0	0	0	0	0	0	0	1	0	0	0	0	0	0	0	0	0	0	0	0	0	1	0	0	0	0	0	0	0	0	0	0	0	0	0	0	0	0	0	1	43.18343333	19.76168333
1077	2012/104	1	0	0	0	1	0	0	0	1	0	0	0	0	0	1	1	0	0	0	0	0	0	0	0	0	0	0	0	0	0	0	0	1	0	0	0	0	0	1	0	0	0	0	0	0	0	0	0	0	0	3	43.02753333	19.40143333
1082	2012/109	1	0	0	0	0	1	0	0	0	1	0	0	0	0	1	0	0	0	1	0	0	0	0	0	0	0	0	0	0	0	0	1	0	0	0	0	0	0	0	1	0	0	0	0	0	0	0	0	0	0	1	42.40636667	19.34983333
1092	2012/119	0	0	0	0	0	1	0	0	0	0	0	0	1	0	1	0	0	0	0	0	0	0	0	0	0	0	0	0	0	0	0	0	0	0	0	0	0	0	0	0	0	0	0	0	0	0	0	0	0	0	3	42.22075	19.11175
1093	2012/120	1	0	0	0	0	0	0	0	0	0	0	0	1	0	0	0	0	0	0	0	0	0	0	0	0	0	0	0	0	0	0	0	0	0	0	0	0	0	0	0	0	0	0	0	0	0	0	0	0	1	1	42.16773333	19.19595
1095	2012/122	1	0	0	0	0	0	0	0	1	1	0	0	1	0	1	0	0	0	1	0	0	0	0	0	0	0	0	0	0	1	0	0	0	0	0	0	0	0	0	0	0	0	0	0	0	0	0	0	0	1	1	41.97678333	19.33868333
1104	2012/131	1	0	0	0	0	0	0	0	0	0	0	0	0	0	0	0	0	0	0	0	0	0	0	0	0	0	0	0	0	0	0	0	0	0	0	0	0	0	0	0	0	0	0	0	0	0	0	0	0	0	1	42.1997	19.81868333
1105	2012/132	1	0	0	0	0	1	0	0	1	1	0	0	1	0	0	0	0	0	0	0	0	0	0	0	0	0	0	0	0	0	0	0	0	0	0	0	0	0	0	0	0	0	0	0	0	0	0	0	0	0	1	42.19455	19.81771667
1106	2012/133	1	0	0	0	0	0	0	0	0	0	0	0	0	0	0	0	0	0	0	0	0	0	0	0	0	0	0	0	0	0	0	0	0	0	0	0	0	0	0	0	0	0	0	0	0	0	0	0	0	0	1	42.1892	19.83275
1108	2012/135	1	0	0	0	0	0	0	0	0	0	0	0	1	0	1	0	0	0	0	0	0	0	0	0	0	0	0	0	0	0	0	0	0	1	0	0	0	0	0	0	0	0	0	0	0	0	0	0	0	1	1	42.23386667	19.87413333
1110	2012/137	1	0	0	0	0	1	0	0	0	0	0	0	0	0	0	0	0	0	1	0	0	0	1	0	0	1	0	0	0	0	1	0	0	0	0	0	0	0	0	1	0	0	0	0	0	0	0	0	0	0	1	42.2652	19.91791667
1113	2012/140	1	0	0	0	0	0	0	0	0	0	0	0	0	0	0	0	0	0	1	0	0	0	1	0	0	1	0	0	0	0	0	0	0	1	0	0	0	0	0	1	0	0	0	0	0	0	0	0	0	0	1	42.2279	19.90705
1114	2012/141	1	0	0	0	0	0	0	0	0	0	0	0	0	0	1	0	0	0	0	0	0	0	0	0	0	0	0	0	0	0	0	0	0	1	0	0	0	0	0	1	0	0	0	0	0	0	0	0	0	1	1	42.10865	19.8264
1119	2012/146	0	0	0	0	0	0	0	0	0	0	0	0	1	0	0	0	0	0	0	0	0	0	0	0	0	0	0	0	0	0	0	1	0	0	0	0	0	0	0	0	0	0	0	0	0	0	0	0	0	0	1	41.36931667	19.98625
1120	2012/147	0	0	0	0	0	0	0	0	0	0	0	0	0	0	0	0	0	0	1	0	0	0	0	0	0	0	0	0	0	0	0	0	0	0	0	0	0	0	0	0	0	0	0	0	0	0	0	0	0	0	1	41.36701667	20.0196
1121	2012/148	0	0	0	0	0	0	0	0	0	0	0	0	0	0	0	0	0	0	0	0	0	0	0	0	0	0	0	0	0	0	0	0	0	0	0	0	0	0	0	0	0	0	0	0	1	0	0	0	0	1	1	41.3512	20.05021667
1122	2012/149	1	0	0	0	0	1	0	0	0	1	0	0	0	1	1	1	1	1	1	0	1	0	1	0	0	1	0	0	1	0	0	0	0	0	0	0	0	0	0	0	0	0	0	0	0	0	0	0	0	1	1	41.35073333	20.05826667
1125	2012/151	1	0	0	0	0	0	0	0	1	0	0	0	1	1	1	1	0	0	0	0	1	0	1	0	0	1	0	1	0	0	1	0	0	0	0	0	0	0	1	0	0	0	0	0	0	0	0	1	1	1	1	41.37053333	20.08546667
1126	2012/152	1	0	0	0	0	0	0	0	0	0	0	0	0	0	0	0	0	0	0	0	0	0	0	0	0	0	0	0	0	0	0	0	0	0	0	0	0	0	0	0	0	0	0	0	0	0	0	1	0	0	3	41.33555	20.15028333
1127	2012/153	0	0	0	0	0	0	0	0	0	0	0	0	0	0	0	0	0	0	1	0	0	0	0	0	0	0	0	0	0	0	0	0	0	0	0	0	0	0	0	0	0	0	0	0	0	0	0	0	0	0	1	41.33453333	20.1636
1129	2012/155	1	0	0	0	0	0	0	0	0	0	0	0	1	0	0	0	0	0	1	0	0	0	0	0	0	0	0	0	0	0	0	0	0	0	0	0	0	0	0	0	0	0	0	0	0	0	0	0	0	1	1	41.33923333	20.19886667
1130	2012/156	1	0	0	0	0	0	0	0	0	0	0	0	0	0	1	0	0	0	1	0	0	0	1	0	0	1	0	0	0	0	0	0	0	0	0	0	0	0	0	0	0	0	0	0	0	0	0	0	0	1	1	41.36035	20.2412
1134	2012/160	1	0	0	0	0	0	0	0	0	0	0	0	0	1	0	1	0	0	1	0	0	0	1	0	0	1	0	0	0	0	0	0	0	0	0	0	0	0	0	0	0	0	0	0	0	0	0	0	1	0	1	41.38821667	20.26055
1135	2012/161	0	0	0	0	0	0	0	0	0	0	0	0	0	0	0	0	0	0	0	0	0	0	0	0	0	0	0	0	0	0	0	0	0	0	0	0	0	0	0	0	0	0	0	0	0	0	0	1	0	0	1	41.37555	20.2686
1136	2012/162	1	0	0	0	0	0	0	0	0	0	0	0	0	0	0	0	0	0	0	0	1	0	0	0	0	0	0	0	0	0	0	0	0	0	0	0	0	0	0	0	0	0	0	0	0	0	0	0	0	1	1	41.33925	20.12878333
1138	2012/164	1	0	0	0	0	1	0	0	1	1	0	0	0	1	1	0	0	0	1	0	0	0	1	0	0	1	0	0	0	0	0	0	0	1	0	0	0	0	0	0	0	0	0	0	0	0	1	1	0	1	1	41.103	20.00645
1139	2012/165	1	0	0	0	0	1	0	0	0	0	0	0	1	1	1	0	0	0	1	0	0	0	0	0	0	0	0	0	0	0	1	0	0	0	0	0	0	0	0	0	0	0	0	0	0	0	1	1	0	1	1	41.16343333	20.23005
1141	0	0	0	0	0	0	1	0	0	0	0	0	0	0	0	0	0	0	0	0	0	0	0	0	0	0	0	0	0	0	0	0	0	0	1	0	0	0	0	0	0	0	0	0	0	0	0	0	0	0	0	1	42.01468	19.62839
1142	0	0	0	0	0	0	0	0	0	0	0	0	0	0	0	0	0	0	0	0	0	0	0	0	0	0	0	0	0	0	0	0	0	0	0	0	0	0	0	0	0	0	0	0	0	0	0	0	0	0	1	1	40.848529	20.86242
1143	0	0	0	0	0	0	0	0	0	0	0	0	0	0	0	0	0	0	0	0	0	0	0	0	0	0	0	0	0	0	0	0	0	0	0	0	0	0	0	0	0	0	0	0	0	0	0	0	0	0	1	2	41.25053	20.50336
1144	0	1	0	0	0	0	0	0	0	0	0	0	0	0	0	0	0	0	0	0	0	0	0	0	0	0	0	0	0	0	0	0	0	0	0	0	0	0	0	0	0	0	0	0	0	0	0	0	0	0	1	1	41.47944	20.08145
1147	2013/001	1	0	0	0	0	1	0	0	0	0	0	0	1	1	1	0	0	0	1	0	0	0	0	0	0	0	0	0	0	0	0	0	1	0	0	0	1	0	0	0	0	0	0	0	0	0	0	0	0	0	1	41.4091788	22.2527529
1148	2013/002	1	0	0	0	0	1	0	0	1	1	0	0	1	1	1	1	0	0	1	0	0	0	1	0	0	1	0	1	1	0	0	1	1	0	0	0	0	0	0	0	0	0	0	1	0	0	0	0	0	1	1	39.9965353	21.2855131
1149	2013/003	0	0	0	0	0	1	0	0	0	0	0	0	1	0	0	0	0	0	0	0	0	0	0	0	0	0	0	0	0	0	0	0	0	0	0	0	0	0	0	0	0	0	0	0	0	0	1	0	0	1	1	40.0076964	21.2805204
1150	2013/004	1	0	0	0	0	0	0	0	0	0	0	0	0	1	1	0	0	0	1	0	0	0	1	0	0	1	0	0	0	0	0	0	1	0	0	0	0	0	0	0	0	0	0	0	0	0	0	0	0	1	1	40.0079535	21.2846782
1151	2013/005	0	0	0	0	0	0	0	0	0	0	0	0	1	1	0	0	0	0	1	0	0	0	0	0	0	0	0	0	0	0	0	0	0	0	0	0	0	0	0	0	0	0	0	0	0	0	0	0	0	0	1	40.0521286	21.2393935
1152	2013/006	1	0	0	0	0	1	0	0	1	0	0	0	1	1	1	0	0	0	1	0	0	1	1	0	1	1	0	0	0	0	0	0	1	0	0	0	0	0	0	0	0	0	0	1	0	0	0	0	0	1	1	40.0741143	21.2016805
1153	2013/007	1	0	0	0	0	1	0	0	1	0	0	0	1	0	1	0	0	0	1	0	0	0	1	0	0	1	0	0	0	0	1	0	1	0	0	0	0	1	0	0	0	0	0	0	0	0	0	1	0	1	1	39.4602844	21.6007841
1154	2013/008	1	0	0	0	0	1	0	0	0	0	0	0	0	1	1	0	0	0	0	0	0	0	0	0	0	0	0	0	0	0	0	0	1	0	0	0	0	0	0	0	0	0	0	0	0	0	0	0	0	0	1	39.4569065	21.59613
1155	2013/009	1	0	0	0	0	1	0	0	0	0	0	0	0	0	1	0	0	0	0	0	0	0	0	0	0	0	0	0	0	0	0	0	0	0	0	0	0	1	0	0	0	0	0	0	0	0	0	0	0	0	1	39.4428565	21.5103952
1156	2013/010	1	0	0	0	0	0	0	0	0	0	0	0	0	0	1	0	0	0	1	0	0	0	0	0	0	0	0	0	0	0	0	0	0	0	0	0	0	0	0	0	0	0	0	0	0	0	0	0	0	0	1	39.4278403	21.4299602
1157	2013/011	0	0	0	0	0	0	0	0	0	0	0	0	1	0	1	0	0	0	1	0	0	0	0	0	0	0	0	0	0	0	0	1	0	0	0	0	0	0	0	0	0	0	0	0	0	0	0	0	0	0	1	39.4613997	21.2938452
1158	2013/012	1	0	0	0	0	0	0	0	0	0	0	0	1	0	1	0	0	0	1	0	0	0	0	0	0	0	0	0	0	0	0	0	0	0	0	0	0	0	0	0	0	0	0	0	0	0	0	0	0	0	1	39.3969136	21.0169787
1159	2013/013	1	0	0	0	0	1	0	0	0	1	0	0	1	0	1	0	0	0	1	0	0	0	1	0	0	1	0	0	0	0	1	0	0	0	0	0	0	0	0	0	0	0	0	0	0	0	0	0	0	1	1	39.4279064	21.0105573
1160	2013/014A	1	0	0	0	0	1	0	0	0	0	0	0	1	0	1	0	0	0	0	0	0	0	0	0	0	0	0	0	0	0	1	0	0	0	0	0	0	0	0	0	0	0	0	0	0	0	0	0	0	1	1	39.5668484	21.1230829
1161	2013/014C	0	0	0	0	0	1	0	0	0	0	0	0	0	0	1	0	0	0	0	0	0	0	1	0	0	1	0	0	0	0	1	0	0	0	0	0	0	0	0	0	0	0	0	0	0	0	0	0	0	1	1	39.5680853	21.1313026
1162	2013/015	0	0	0	0	0	1	0	0	0	0	0	0	1	0	1	0	0	0	1	0	0	0	1	0	0	1	0	0	0	0	1	0	0	0	0	0	0	0	0	0	0	0	0	0	0	0	0	0	0	1	1	39.5767837	21.0969426
1163	2013/016	1	0	0	0	0	1	0	0	0	0	0	0	0	1	1	0	0	0	1	0	0	0	1	0	0	1	0	0	0	0	1	0	0	0	0	0	0	0	0	0	0	0	0	0	0	0	0	0	0	1	1	39.5728428	20.986106
1164	2013/017	1	0	0	0	0	1	0	0	0	0	0	0	0	0	1	0	0	0	0	0	0	0	1	0	0	1	0	0	0	0	0	0	0	0	0	0	0	0	0	0	0	0	0	0	0	0	0	0	0	1	1	39.5762953	20.9771778
1167	2013/020	1	0	0	0	0	1	0	0	0	0	0	0	0	1	1	0	0	0	0	0	0	0	0	0	0	0	0	0	0	0	1	0	0	0	0	0	0	0	0	0	0	0	0	0	0	0	0	1	0	1	1	39.689521	20.8713509
1168	2013/021	0	0	0	0	0	0	0	0	0	0	0	0	0	0	0	0	0	0	0	0	0	0	0	0	0	0	0	0	0	0	1	0	0	0	0	0	0	0	0	0	0	0	0	0	0	0	0	0	0	1	1	39.6870318	20.8740156
1169	2013/022	1	0	0	0	0	0	0	0	0	0	0	0	0	1	1	0	0	0	0	0	0	0	0	0	0	0	0	0	0	0	1	0	0	0	1	0	0	0	0	0	0	0	0	0	0	0	0	0	0	1	1	39.6848876	20.8795284
1170	2013/023	1	0	0	0	0	0	0	0	0	0	0	0	0	1	1	1	0	0	0	0	0	0	0	0	0	0	0	0	0	0	1	0	0	0	1	0	0	0	0	0	0	0	0	0	0	0	0	0	0	1	1	39.6828228	20.8886677
1171	2013/024	1	0	0	0	0	0	0	0	0	0	0	0	0	1	1	0	0	0	0	0	0	0	0	0	0	0	0	0	0	0	1	0	0	0	1	0	0	0	0	0	0	0	0	0	0	0	1	0	0	1	1	39.6801669	20.9052407
1172	2013/025	1	0	0	0	0	1	0	0	0	0	0	0	1	1	0	0	0	0	0	0	0	0	0	0	0	0	0	0	0	0	1	0	0	0	0	0	0	0	0	0	0	0	0	0	0	0	0	0	0	1	1	39.6947455	20.8463411
1173	2013/026	1	0	0	0	0	1	0	0	0	0	0	0	0	1	1	0	0	0	0	0	0	0	0	0	0	0	0	0	0	0	1	0	0	0	0	0	0	0	0	0	0	0	1	0	0	0	0	0	0	1	1	39.697139	20.8432918
1174	2013/027	1	0	0	0	0	1	0	0	0	0	0	0	1	1	1	0	0	0	0	0	0	0	0	0	0	0	0	0	0	0	1	0	0	0	1	0	0	0	0	0	0	0	1	0	0	0	1	0	0	1	1	39.7244725	20.8428578
1175	2013/028	0	0	0	0	0	0	0	0	0	0	0	0	0	0	0	0	0	0	0	0	0	0	0	0	0	0	0	0	0	0	0	0	0	0	1	0	0	0	0	0	0	0	0	0	0	0	0	1	0	0	1	39.7679699	20.6095984
1176	2013/029	1	0	0	0	0	0	0	0	0	0	0	0	1	0	1	0	0	0	0	0	0	0	0	0	0	0	0	0	0	0	0	1	0	0	0	0	0	0	0	0	0	0	0	0	0	0	1	1	0	0	2	39.7642158	20.616866
1177	2013/030	1	0	0	0	0	0	0	0	0	1	0	0	1	1	0	0	0	0	0	0	0	0	0	0	0	0	0	0	0	0	0	0	0	0	1	0	0	0	0	0	0	0	0	0	0	0	1	0	0	0	3	39.7670511	20.6147516
1178	2013/031	1	0	0	0	0	1	0	0	0	0	0	0	1	1	1	0	0	0	1	0	0	0	0	0	0	0	0	0	0	0	1	1	0	0	1	0	0	0	0	0	0	0	0	0	0	0	1	1	0	1	3	39.7720295	20.61797
1179	2013/032	0	0	0	0	0	0	0	0	0	0	0	0	1	0	0	0	0	0	1	0	0	0	0	0	0	0	0	0	0	0	0	0	0	0	1	0	0	0	0	0	0	0	0	0	0	0	1	0	0	1	1	39.7358415	20.4536566
1180	2013/033	1	0	0	0	0	0	0	0	0	0	0	0	1	0	1	0	0	0	0	0	0	0	0	0	0	0	0	0	0	0	0	0	0	0	0	0	0	0	0	0	0	0	0	0	0	0	1	0	0	0	1	39.8777481	20.4657989
1181	2013/034	1	0	0	0	0	0	0	0	1	0	0	0	1	0	1	1	0	0	1	0	0	0	1	0	0	1	0	0	0	0	1	1	0	0	0	0	0	0	0	0	0	0	0	0	0	0	0	1	0	1	1	39.890997	20.7538141
1182	2013/035	1	0	0	0	0	0	0	0	0	0	0	0	0	0	0	0	0	0	0	0	0	0	0	0	0	0	0	0	0	0	0	0	0	0	0	0	0	0	0	0	0	0	0	0	0	0	0	1	0	0	1	39.8950952	20.7413356
1183	2013/036	1	0	0	0	0	0	0	0	0	0	0	0	0	0	0	0	0	0	1	0	0	0	0	0	0	0	0	0	0	0	0	0	0	0	0	0	0	0	0	0	0	0	0	0	0	0	0	0	0	1	1	39.9067313	20.751873
1184	2013/037	1	0	0	0	0	0	0	0	0	0	0	0	1	0	1	0	0	0	1	0	0	0	0	0	0	0	0	0	0	0	1	0	0	0	0	0	0	0	0	0	0	0	0	0	0	0	0	0	0	1	1	39.862	20.7749
1185	2013/038	0	0	0	0	0	0	0	0	0	0	0	0	1	0	0	0	0	0	0	1	0	0	1	0	0	1	0	0	0	0	0	0	0	0	1	0	0	0	0	0	0	0	0	0	0	0	0	1	0	1	1	39.9674078	20.6645715
1186	2013/039	1	0	0	0	0	0	0	0	1	0	0	0	1	0	1	0	0	0	1	0	0	0	1	0	0	1	0	0	0	0	1	0	0	0	0	0	0	0	0	0	0	0	0	0	0	0	1	0	0	1	1	40.0467572	20.5648481
1187	2013/040	1	0	0	0	0	1	0	0	0	0	0	0	1	0	1	0	0	0	0	0	0	0	1	0	0	1	0	0	0	0	0	0	0	0	0	0	0	0	0	0	0	0	0	0	0	0	0	1	0	1	1	40.03549	20.74694
1188	2013/041	1	0	0	0	0	1	0	0	1	0	0	0	1	0	1	0	0	0	1	0	0	0	1	0	0	1	0	0	0	0	0	0	0	0	0	0	0	0	0	0	0	0	0	0	0	0	0	1	0	1	1	40.1102296	20.7202726
1192	2013/045	0	0	0	0	0	0	0	0	0	1	0	0	1	0	0	1	0	0	1	0	1	1	1	0	1	1	0	0	0	0	0	0	0	0	0	0	0	0	0	0	0	0	0	0	0	0	0	0	0	2	1	40.3090431	20.8980564
1193	2013/046	0	0	0	0	0	0	0	0	0	0	0	0	1	0	1	0	0	0	0	0	1	0	0	0	0	0	0	0	0	0	0	0	0	0	0	0	0	0	0	0	0	0	0	0	0	0	0	0	0	1	1	40.3114583	20.8986418
1194	2013/047	0	0	0	0	0	1	0	0	0	0	0	0	1	0	1	0	0	0	0	0	1	0	0	0	0	0	0	0	0	0	0	0	0	0	0	0	0	0	0	0	0	0	0	0	0	0	0	0	0	1	1	40.3130587	20.8994665
1196	2013/049A	0	0	0	0	0	1	0	0	0	0	0	0	1	1	1	0	0	0	0	0	0	0	0	0	0	0	0	0	0	0	0	0	0	0	0	0	0	0	0	0	0	0	0	0	0	0	0	0	0	1	1	40.4430603	21.4102248
1198	2013/050	0	0	0	0	0	1	0	0	0	1	0	0	0	1	1	0	0	0	0	0	0	0	0	0	0	0	0	0	0	0	0	0	0	0	0	0	0	0	0	0	0	0	0	0	0	0	0	0	0	1	1	40.3781789	21.4159985
1201	2013/053	0	0	0	0	0	0	0	0	1	0	0	0	0	1	0	0	0	0	0	0	0	0	0	0	0	0	0	0	0	0	0	0	1	0	0	0	0	0	0	0	0	0	0	0	0	0	0	0	0	0	1	40.5904817	21.1921266
1202	2013/054	0	0	0	0	0	1	0	0	1	0	0	0	0	0	1	0	0	0	0	0	0	0	0	0	0	0	0	0	0	0	0	0	0	0	0	0	0	0	0	0	0	0	0	0	0	0	0	0	0	0	1	40.6555158	21.1811746
1203	2013/055	0	0	0	0	0	0	0	0	1	0	0	0	0	0	0	0	0	0	0	0	0	0	0	0	0	0	0	0	0	0	0	0	1	0	0	0	0	0	0	0	0	0	0	0	0	0	0	1	0	1	1	40.7429279	21.1101852
1205	2013/057	0	0	0	0	0	1	0	0	1	0	0	0	0	1	0	0	0	0	0	0	0	0	0	0	0	0	0	0	0	0	0	0	1	0	0	0	0	0	0	0	0	0	0	0	0	0	0	0	0	1	1	40.8315864	21.0286694
1207	2013/059	0	0	0	0	0	1	0	0	1	0	0	0	0	0	0	1	0	0	0	0	0	0	0	0	0	0	0	0	0	0	0	0	1	0	0	0	0	0	0	0	0	0	0	0	0	0	0	0	0	1	1	40.672265	20.9871246
1208	2013/060	0	0	0	0	0	1	0	0	1	1	0	0	1	0	1	0	0	0	1	0	0	0	0	0	0	0	0	0	0	0	0	0	1	0	0	0	0	0	0	0	0	0	0	0	0	0	0	1	0	1	1	40.7330062	20.87294
1209	2013/061	0	0	0	0	0	1	0	0	1	0	0	0	1	1	0	0	0	0	1	0	0	0	0	0	0	0	0	0	0	0	0	0	1	0	0	0	0	0	0	0	0	0	0	0	0	0	0	0	0	1	1	40.791891	20.9151703
1210	2013/062	0	0	0	0	0	1	0	0	0	0	0	0	1	1	0	0	0	0	0	0	0	0	0	0	0	0	0	0	0	0	0	0	0	0	0	0	0	0	0	0	0	0	0	0	0	0	0	0	0	1	1	40.7688301	20.8274532
1211	2013/063	0	0	0	0	0	1	0	0	1	0	0	0	1	1	1	0	0	0	1	0	0	0	0	0	0	0	0	0	0	0	0	0	0	0	0	0	0	0	0	0	0	0	0	0	0	0	0	1	0	1	1	40.9443849	20.6109453
1213	2013/065	0	0	0	0	0	1	0	0	0	0	0	0	0	0	0	0	0	0	0	0	0	0	0	0	0	0	0	0	0	0	0	0	0	0	0	0	0	0	0	0	0	0	0	0	0	0	0	0	0	1	1	41.0395881	20.6347365
1214	2013/066	1	0	0	0	0	1	0	0	1	0	0	0	1	0	1	0	0	0	1	1	1	0	1	0	0	1	0	0	1	0	0	0	1	0	0	0	0	0	0	0	0	0	0	0	0	0	0	1	0	1	1	41.0692523	20.6467607
1215	2013/067	0	0	0	0	0	1	0	0	0	0	0	0	0	0	1	0	0	0	0	0	0	0	0	0	0	0	0	0	0	0	0	0	0	0	0	0	0	0	0	0	0	0	0	0	0	0	0	0	0	0	2	41.0682353	20.6097743
1216	0	0	0	0	0	0	0	0	0	0	0	0	0	0	0	0	0	0	0	0	0	0	0	0	0	0	0	0	0	0	0	0	0	0	0	0	0	0	0	0	0	0	0	0	0	0	0	0	0	0	1	3	41.68	21.4399
1217	0	0	0	0	0	0	0	0	0	1	1	0	0	0	0	0	0	0	0	0	0	1	0	0	0	0	0	0	0	0	0	0	0	0	0	0	0	0	0	0	1	0	0	0	0	0	0	0	0	0	1	1	41.6827	21.4397
1218	0	0	0	0	0	0	0	0	0	0	0	0	0	0	0	0	0	0	0	0	0	0	0	0	0	0	0	0	0	0	0	0	0	0	0	0	0	0	0	0	0	0	0	0	0	0	0	0	0	0	1	1	40.3983	21.4143
1219	0	0	0	0	0	0	0	0	0	0	0	0	0	0	0	0	0	0	0	0	0	0	0	0	0	0	0	0	0	0	0	0	0	0	0	0	0	0	0	0	0	0	0	0	0	0	0	0	0	0	1	1	39.681	21.122
1221	0	0	0	0	0	0	0	0	0	0	0	0	0	0	0	0	0	0	0	0	0	0	0	0	0	0	0	0	0	0	0	0	0	0	0	0	0	0	0	0	0	0	0	0	0	0	0	0	0	0	1	1	40.684722	20.998417

1222	0	0	0	0	0	0	0	0	0	0	0	0	0	0	0	0	0	0	0	0	0	0	0	0	0	0	0	0	0	0	0	0	0	0	0	0	0	0	0	0	0	0	0	0	0	0	0	0	1	1	40.2967	20.9433
1224	0	0	0	0	0	0	0	0	0	0	0	0	0	0	0	0	0	0	0	0	0	0	0	0	0	0	0	0	0	0	0	0	0	0	0	0	0	0	0	0	0	0	0	0	0	0	0	0	1	1	40.2948	20.9381
1225	0	0	0	0	0	0	0	0	0	0	0	0	0	0	0	0	0	0	0	0	0	0	0	0	0	0	0	0	0	0	0	0	0	0	0	0	0	0	0	0	0	0	0	0	0	0	0	0	1	1	40.766806	20.927722
1226	0	0	0	0	0	0	0	0	0	0	0	0	0	0	0	0	0	0	0	0	0	0	0	0	0	0	0	0	0	0	0	0	0	0	0	0	0	0	0	0	0	0	0	0	0	0	0	0	1	1	40.3117	20.9072
1228	0	0	0	0	0	0	0	0	0	0	0	0	0	0	0	0	0	0	0	0	0	0	0	0	0	0	0	0	0	0	0	0	0	0	0	0	0	0	0	0	0	0	0	0	0	0	0	0	1	1	40.3449	20.8813
1229	0	0	0	0	0	0	0	0	0	0	0	0	0	1	1	0	0	0	0	0	0	0	0	0	0	0	0	0	0	0	0	0	0	0	0	0	0	0	0	0	0	0	0	0	0	0	0	0	1	1	40.9718	20.8788
1231	0	0	0	0	0	0	0	0	0	0	0	0	0	0	0	0	0	0	0	0	0	0	0	0	0	0	0	0	0	0	0	0	0	0	0	0	0	0	0	0	0	0	0	0	0	0	0	0	1	1	39.6986	20.8482
1234	0	0	0	0	0	0	0	0	0	0	0	0	0	0	0	0	0	0	0	0	0	0	0	0	0	0	0	0	0	0	0	0	0	0	0	0	0	0	0	0	0	0	0	0	0	0	0	0	1	1	39.72765	20.823
1236	0	0	0	0	0	0	0	0	0	0	0	0	0	0	0	0	0	0	0	0	0	0	0	0	0	0	0	0	0	0	0	0	0	0	0	0	0	0	0	0	0	0	0	0	0	0	0	0	1	1	39.7828	20.8041
1239	0	0	0	0	0	0	0	0	0	0	0	0	0	0	0	0	0	0	0	0	0	0	0	0	0	0	0	0	0	0	0	0	0	0	0	0	0	0	0	0	0	0	0	0	0	0	0	0	1	1	39.7345	20.7737
1240	0	0	0	0	0	0	0	0	0	0	0	0	0	0	0	0	0	0	0	0	0	0	0	0	0	0	0	0	0	0	0	0	0	0	0	0	0	0	0	0	0	0	0	0	0	0	0	0	1	1	39.9773	20.7644
1241	0	0	0	0	0	0	0	0	0	0	0	0	0	0	0	0	0	0	0	0	0	0	0	0	0	0	0	0	0	0	0	0	0	0	0	0	0	0	0	0	0	0	0	0	0	0	0	0	1	1	40.0706	20.7608
1252	0	0	0	0	0	0	0	0	0	0	0	0	0	0	0	0	0	0	0	0	0	0	0	0	0	0	0	0	0	0	0	0	0	0	0	0	0	0	0	0	0	0	0	0	0	0	0	0	1	1	41.2234	20.537
1253	0	0	0	0	0	0	0	0	0	0	0	0	0	0	0	0	0	0	0	0	0	0	0	0	0	0	0	0	0	0	0	0	0	0	0	0	0	0	0	0	0	0	0	0	0	0	0	0	1	1	41.203	20.532
1254	0	0	0	0	0	0	0	0	0	0	0	0	0	0	0	0	0	0	0	0	0	0	0	0	0	0	0	0	0	0	0	0	0	0	0	0	0	0	0	0	0	0	0	0	0	0	0	0	1	1	41.35749	20.46226
1256	0	0	0	0	0	0	0	0	0	0	0	0	0	0	0	0	0	0	0	0	0	0	0	0	0	0	0	0	0	0	0	0	0	0	0	0	0	0	0	0	0	0	0	0	0	0	0	0	1	1	39.7822	20.3465
1257	0	0	0	0	0	0	0	0	0	0	0	0	0	0	0	0	0	0	0	0	0	0	0	0	0	0	0	0	0	0	0	0	0	0	0	0	0	0	0	0	1	0	0	0	0	0	0	0	1	1	42.56	20.21
1260	0	0	0	0	0	0	0	0	0	0	0	0	0	0	0	0	0	0	0	0	0	0	0	0	0	0	0	0	0	0	0	0	0	0	0	0	0	0	0	0	0	0	0	0	0	0	0	0	1	1	42.05717	19.39625
1263	0	0	0	0	0	0	0	0	0	0	0	0	0	0	0	0	0	0	0	0	0	0	0	0	0	0	0	0	0	0	0	0	0	0	0	0	0	0	0	0	0	0	0	0	0	0	0	0	1	1	42.3539	19.3416
1264	0	0	0	0	0	0	0	0	0	0	0	0	0	0	0	0	0	0	0	0	0	0	0	0	0	0	0	0	0	0	0	0	0	0	0	0	0	0	0	0	0	0	0	0	0	0	0	0	1	1	42.0387	19.2673
1268	0	0	0	0	0	0	0	0	0	0	0	0	0	0	0	0	0	0	0	0	0	0	0	0	0	0	0	0	0	0	0	0	0	0	0	0	0	0	0	0	0	0	0	0	0	0	0	0	1	1	42.1099	19.2321
1269	0	0	0	0	0	0	0	0	0	0	0	0	0	0	0	0	0	0	0	0	0	0	0	0	0	0	0	0	0	0	0	0	0	0	0	0	0	0	0	0	0	0	0	0	0	0	0	0	1	1	42.1479	19.2191
1270	0	0	0	0	0	0	0	0	0	0	0	0	0	0	0	0	0	0	0	0	0	0	0	0	0	0	0	0	0	0	0	0	0	0	0	0	0	0	0	0	0	0	0	0	0	0	0	0	1	1	42.3132	19.1885
1271	0	0	0	0	0	0	0	0	0	0	0	0	0	0	0	0	0	0	0	0	0	0	0	0	0	0	0	0	0	0	0	0	0	0	0	0	0	0	0	0	0	0	0	0	0	0	0	0	1	1	42.1323	19.1434
1272	0	0	0	0	0	0	0	0	0	0	0	0	0	0	0	0	0	0	0	0	0	0	0	0	0	0	0	0	0	0	0	0	0	0	0	0	0	0	0	0	0	0	0	0	0	0	0	0	1	1	42.067	19.14
1273	0	0	0	0	0	0	0	0	0	0	0	0	0	0	0	0	0	0	0	0	0	0	0	0	0	0	0	0	0	0	0	0	0	0	0	0	0	0	0	0	0	0	0	0	0	0	0	0	1	1	42.1373	19.1398
1274	0	0	0	0	0	0	0	0	0	0	0	0	0	0	0	0	0	0	0	0	0	0	0	0	0	0	0	0	0	0	0	0	0	0	0	0	0	0	0	0	0	0	0	0	0	0	0	0	1	1	42.3917	19.133
1277	0	0	0	0	0	0	0	0	0	0	0	0	0	0	0	0	0	0	0	0	0	0	0	0	0	0	0	0	0	0	0	0	0	0	0	0	0	0	0	0	0	0	0	0	0	0	0	0	1	1	42.1512	19.1125
1278	0	0	0	0	0	0	0	0	0	0	0	0	0	0	0	0	0	0	0	0	0	0	0	0	0	0	0	0	0	0	0	0	0	0	0	0	0	0	0	0	0	0	0	0	0	0	0	0	1	1	42.1829	19.1091
1280	0	0	0	0	0	0	0	0	0	0	0	0	0	0	0	0	0	0	0	0	0	0	0	0	0	0	0	0	0	0	0	0	0	0	0	0	0	0	0	0	0	0	0	0	0	0	0	0	1	1	42.1713	19.0982
1286	0	0	0	0	0	0	0	0	0	0	0	0	0	0	0	0	0	0	0	0	0	0	0	0	0	0	0	0	0	0	0	0	0	0	0	0	0	0	0	0	0	0	0	0	0	0	0	0	1	1	42.2057	19.0559
1289	0	0	0	0	0	0	0	0	0	0	0	0	0	0	0	0	0	0	0	0	0	0	0	0	0	0	0	0	0	0	0	0	0	0	0	0	0	0	0	0	0	0	0	0	0	0	0	0	1	1	42.1998	18.9607
1291	0	0	0	0	0	0	0	0	0	0	0	0	0	0	0	0	0	0	0	0	0	0	0	0	0	0	0	0	0	0	0	0	0	0	0	0	0	0	0	0	0	0	0	0	0	0	0	0	1	1	42.42666667	18.83138889
1294	0	0	0	0	0	0	0	0	0	0	0	0	0	0	0	0	0	0	0	0	0	0	0	0	0	0	0	0	0	0	0	0	0	0	0	0	0	0	0	0	0	0	0	0	0	0	0	0	1	1	42.4116	18.7623
1295	0	0	0	0	0	0	0	0	0	0	0	0	0	0	0	0	0	0	0	0	0	0	0	0	0	0	0	0	0	0	0	0	0	0	0	0	0	0	0	0	0	0	0	0	0	0	0	0	1	1	42.413	18.7615
1296	0	0	0	0	0	0	0	0	0	0	0	0	0	0	0	0	0	0	0	0	0	0	0	0	0	0	0	0	0	0	0	0	0	0	0	0	0	0	0	0	0	0	0	0	0	0	0	0	1	1	42.4901	18.7532
1297	1972/001	0	0	0	0	0	1	0	0	0	0	0	0	1	0	0	0	0	0	1	0	0	0	1	0	0	0	1	0	0	0	1	0	0	0	1	0	0	0	0	0	0	0	0	0	0	0	0	0	1	43.3342	22.0792
1298	1972/002	0	0	0	0	0	0	0	0	0	1	0	0	1	0	0	0	0	0	0	0	0	0	0	0	0	0	0	0	0	0	1	0	0	0	1	0	0	0	0	0	0	0	0	0	0	0	0	0	1	43.3302	22.0636
1300	1972/004	1	0	0	0	0	1	0	0	0	0	0	0	1	0	0	0	0	0	1	0	0	0	0	0	0	0	0	0	0	0	1	0	0	0	1	0	0	0	0	0	0	0	0	0	0	0	0	0	1	41.4322	21.8516
1301	1972/005	0	0	0	0	0	0	0	0	0	0	0	0	0	0	0	0	0	0	0	0	0	0	0	0	0	0	0	0	0	0	0	0	0	0	0	0	0	0	0	0	0	0	0	0	0	0	0	0	1	41.361699	21.703273
1306	1972/010	1	0	0	0	0	1	0	0	0	0	0	0	0	0	0	0	0	0	0	0	0	0	0	0	0	0	0	0	0	0	0	0	0	0	0	0	0	0	0	0	0	0	0	0	0	0	0	0	1	41.8949	21.6864
1316	1972/020	1	0	0	0	0	1	0	0	0	0	0	0	0	0	0	0	0	0	1	0	0	0	1	0	0	0	0	0	0	0	0	0	0	0	0	0	0	0	0	0	0	0	0	0	0	1	0	0	1	40.95113	20.8004
1318	1972/022	1	0	0	0	0	0	0	0	0	0	0	0	0	1	0	0	0	0	1	0	0	0	0	0	0	0	0	0	0	0	0	0	0	0	0	0	0	0	0	0	0	0	0	0	0	1	0	0	1	40.9688	20.7905
1319	1972/023	0	0	0	0	0	1	0	0	0	0	0	0	0	0	0	0	0	0	0	0	0	0	0	0	0	0	0	0	0	0	1	0	0	0	0	0	0	0	0	0	0	0	0	0	0	1	0	1	1	41.1109	20.7915
1320	1972/024	0	0	0	0	0	1	0	0	0	0	0	0	0	1	0	0	0	0	0	0	0	0	0	0	0	0	0	0	0	0	0	0	0	0	0	0	0	0	0	0	0	0	0	0	0	1	0	0	1	41.2626	20.6379
1323	1972/027	1	0	0	0	0	1	0	0	0	0	0	0	0	1	0	0	0	0	1	0	0	0	0	0	0	0	0	0	0	0	1	0	0	0	0	0	0	0	0	0	0	0	0	0	0	0	0	1	1	41.4432	20.5374
1324	1972/028	1	0	0	0	0	1	0	0	0	0	0	0	0	0	0	0	0	0	0	0	0	0	0	0	0	0	0	0	0	0	0	0	0	0	0	0	0	0	0	0	0	0	0	0	0	0	0	0	1	41.554	20.6161
1330	1972/034	0	0	0	0	0	1	0	0	0	0	0	0	0	0	0	0	0	0	0	0	0	0	1	0	0	0	0	0	0	0	1	0	0	0	0	0	1	0	0	0	0	0	0	0	0	0	0	0	1	42.7711	20.2804
1331	1972/035	0	0	0	0	0	1	0	0	0	1	0	0	0	0	0	0	0	1	1	0	0	0	1	0	0	0	1	0	0	1	1	0	0	0	0	0	1	0	0	1	0	0	0	0	0	0	0	0	1	42.9524	20.4189
1332	1972/036	1	0	0	0	0	1	0	0	0	0	0	0	0	0	0	0	0	0	1	0	0	0	1	0	0	0	1	0	0	0	1	0	0	0	0	0	0	0	0	0	0	0	0	0	0	0	0	0	1	42.8544	20.2057
1333	1972/037	1	0	0	0	0	1	0	0	0	0	0	0	0	0	0	0	0	0	0	0	0	0	0	0	0	0	0	0	1	1	0	0	0	0	0	0	0	0	0	0	0	0	0	0	0	0	0	0	1	42.7602	19.3896
1334	1972/038	1	0	0	0	0	0	0	0	0	0	0	0	1	0	0	0	0	0	0	0	0	0	0	0	0	0	0	0	1	0	0	0	0	0	0	0	0	1	0	0	0	0	0	0	0	0	0	1	1	42.3828	19.1022
1336	1972/040	0	0	0	0	0	0	0	0	0	1	0	0	1	0	0	0	0	0	0	0	1	0	0	0	0	0	0	0	0	1	1	0	0	0	0	0	0	1	0	0	0	0	0	0	0	0	0	1	1	42.3367	18.9971
1338	1972/042	0	0	0	0	0	0	0	0	0	0	0	0	0	0	0	0	0	0	0	0	0	0	0	0	0	0	0	0	0	0	0	0	0	0	0	0	0	1	0	0	0	0	0	0	0	0	0	0	1	42.2711	19.1241
1339	1972/043	0	0	0	0	0	0	0	0	0	0	0	0	1	0	0	0	0	0	0	0	0	0	0	0	0	0	0	0	1	1	0	0	0	0	0	0	0	1	0	0	0	0	0	0	0	0	0	1	1	42.2248	19.125
1340	1972/044	0	0	0	0	0	0	0	0	0	0	0	0	0	0	0	0	0	0	0	0	0	0	0	0	0	0	0	0	0	0	0	0	0	0	0	0	0	1	0	0	0	0	0	0	0	0	0	0	1	42.2137	19.0152
1342	1972/046	1	0	0	0	0	1	0	0	0	1	0	0	1	0	1	0	0	0	1	0	1	0	0	0	0	0	0	0	1	1	0	1	0	0	0	0	0	0	0	0	0	0	1	0	1	0	0	0	1	42.5136	18.6816
1344	1972/048	1	0	0	0	0	0	0	0	0	0	0	0	0	0	0	0	0	0	0	0	0	0	0	0	0	0	0	0	0	0	0	1	0	0	0	0	0	0	0	0	0	0	0	0	0	0	0	0	1	42.4282	18.811
1346	1972/050	0	0	0	0	0	0	0	0	0	0	0	0	1	0	0	0	0	0	0	0	0	0	0	0	0	0	0	0	0	0	0	0	0	0	0	0	0	0	0	0	0	0	0	0	0	0	0	0	1	42.4302	18.8128
1347	1972/051	0	0	0	1	0	0	0	0	0	0	0	0	0	0	1	0	0	0	0	1	0	0	0	0	0	0	0	0	1	0	0	1	0	0	0	0	0	1	0	0	0	0	0	0	0	0	0	0	1	42.6275	18.1198
1348	1972/052	1	0	0	0	0	0	0	0	0	0	0	0	1	0	0	0	0	0	0	1	0	0	0	0	0	0	0	0	0	0	0	0	0	0	0	0	0	0	0	0	0	0	0	0	1	0	0	0	1	42.6568	18.0823
1350	1972/054	0	0	0	1	0	0	0	0	0	0	0	0	1	0	0	0	0	0	0	0	1	0	0	0	0	0	0	0	1	0	0	0	0	0	0	0	0	0	0	0	0	0	0	0	0	0	0	0	1	42.5853	18.2169
1352	1972/056	1	0	0	1	0	0	0	0	0	1	0	0	1	0	1	0	0	0	1	0	1	0	0	0	0	0	0	0	1	0	0	1	0	0	0	0	0	0	0	0	0	0	0	0	0	0	0	0	1	42.6708	18.1013
1354	1972/058	0	0	0	1	0	0	0	0	0	1	0	0	1	0	1	0	0	0	1	0	0	0	0	0	0	0	0	0	0	0	0	0	0	0	0	0	0	1	0	0	0	0	0	0	0	0	0	0	1	42.7358	17.5982
1355	1972/059	1	0	0	1	0	0	0	0	0	1	0	0	1	0	1	0	0	1	1	0	0	0	0	0	0	0	1	0	1	0	0	1	0	0	0	0	0	1	0	0	0	0	0	0	0	0	0	0	1	42.7368	17.6005
1356	1972/060	1	0	0	1	0	0	0	0	0	1	0	0	1	0	1	0	0	0	1	0	0	0	0	0	0	0	0	0	1	0	0	1	0	0	0	0	0	1	0	0	0	0	0	1	0	0	0	0	1	42.7316	17.5791
1357	1972/061	1	0	0	0	0	0	0	0	0	0	0	0	0	0	0	0	0	0	1	0	0	0	0	0	0	0	0	0	0	0	0	0	0	0	0	0	0	0	0	0	0	0	0	0	0	0	0	0	1	43.0281	17.651
1358	1972/062	0	0	0	0	0	0	0	0	0	0	0	0	1	0	0	0	0	0	1	0	0	0	0	0	0	0	0	0	1	0	0	1	0	0	0	0	0	1	0	0	0	0	0	0	0	0	0	0	1	43.0178	17.6757
1359	1972/063	1	0	0	0	0	0	0	0	0	0	0	0	1	0	1	0	0	0	1	0	0	0	0	0	0	0	0	0	1	0	0	1	0	0	0	0	0	1	0	0	0	0	0	0	0	0	0	0	1	43.0412	17.6605
1360	1972/064	1	0	0	0	0	0	0	0	0	0	0	0	0	0	0	0	0	0	1	0	0	0	0	0	0	0	0	0	0	0	0	0	0	0	0	0	0	0	0	0	0	0	1	0	0	0	0	0	1	43.4008	16.8642
1363	1972/067	0	0	0	0	0	0	0	0	0	0	0	0	0	0	0	0	0	0	0	0	0	0	0	0	0	0	0	0	1	0	1	0	0	0	0	0	0	0	0	0	0	0	0	0	0	0	0	0	1	44.9811	14.9808
1364	1972/068	0	0	0	0	0	0	0	0	0	0	0	0	0	0	0	0	0	0	0	0	0	0	0	0	0	0	0	0	0	0	0	0	0	0	0	0	0	0	0	0	0	0	0	0	0	0	0	0	1	44.7873	15.3672
1365	1972/069	1	0	0	0	0	1	0	0	0	1	0	0	1	0	1	0	0	1	1	0	0	0	1	0	0	0	1	0	1	1	1	0	0	0	0	0	1	0	0	1	0	0	0	0	1	0	0	0	1	44.9015	15.6087
1366	1976/005	1	0	0	0	0	0	0	0	0	1	0	0	0	0	0	0	0	0	0	0	0	0	0	0	0	0	0	0	0	0	0	0	0	0	0	0	0	0	0	0	0	0	0	0	0	0	0	0	1	39.4771	20.3137
1367	1976/006	0	0	0	0	0	0	0	0	0	1	0	0	0	0	0	0	0	0	0	0	0	0	0	0	0	0	0	0	0	0	0	0	0	0	0	0	0	0	0	0	0	0	0	0	0	0	0	0	1	39.5944	20.4918
1368	1976/007	1	0	0	0	0	0	0	0	0	0	0	0	0	0	0	0	0	0	0	0	0	0	0	0	0	0	0	0	0	0	0	0	0	0	0	0	0	0	0	0	0	0	0	0	0	0	0	0	1	39.6442	20.5129
1369	1976/008	0	0	0	0	0	0	0	0	0	1	0	0	0	0	0	0	0	0	0	0	0	0	0	0	0	0	0	0	0	0	0	0	0	0	0	0	0	0	0	0	0	0	0	0	0	0	0	0	1	39.6393	20.9145
1370	1976/009	0	0	0	0	0	0	0	0	0	0	0	0	0	0	0	0	0	0	0	0	0	0	0	0	0	0	0	0	0	0	0	0	0	0	0	0	0	0	0	0	0	0	0	0	0	0	0	0	1	39.7882	21.3874
1371	1976/010	0	0	0	0	0	0	0	0	0	0	0	0	1	0	0	0	0	0	0	0	0	0	0	0	0	0	0	0	0	0	1	0	0	0	1	0	0	0	0	0	0	0	0	0	1	0	0	0	1	40.1131	22.485
1372	1976/011	1	0	0	0	0	0	0	0	0	1	0	0	1	1	0	0	0	0	0	0	0	0	0	0	0	0	0	0	0	0	0	0	0	0	1	0	0	0	0	0	0	0	0	0	1	0	0	0	1	39.8796	22.587
1374	1976/013	0	0	0	0	0	1	0	0	0	0	0	0	0	1	0	0	0	0	1	0	0	0	0	0	0	0	0	0	0	0	0	0	0	0	1	0	0	0	0	0	0	0	0	0	0	0	0	0	1	39.6991	22.2134
1378	1976/017	1	0	0	0	0	0	0	0	0	0	0	0	0	0	0	0	0	0	1	0	0	0	0	0	0	0	0	0	0	0	0	0	0	0	0	0	0	0	0	0	0	0	0	0	0	0	0	0	1	39.7326	21.0589
1383	1976/023	0	0	0	0	0	0	0	0	0	0	0	0	0	0	0	0	0	0	0	0	0	0	0	0	0	0	0	0	0	0	0	0	0	0	0	0	0	0	0	0	0	0	0	0	0	0	0	0	1	39.4959	20.875
1384	1976/024	1	0	0	0	0	0	0	0	0	1	0	0	1	0	0	0	0	0	1	1	0	0	0	0	0	0	1	0	1	0	0	0	0	0	0	0	0	0	0	0	0	0	0	0	0	0	0	0	1	39.4497	20.879
1385	1976/025	1	0	0	0	0	0	0	0	0	1	0	0	0	0	0	0	0	0	1	0	0	0	0	0	0	0	1	0	1	0	0	0	1	0	0	0	0	0	0	0	0	0	0	0	0	0	0	0	1	39.3607	20.888
1386	1976/026	1	0	0	0	0	0	0	0	0	1	0	0	1	0	0	0	0	0	1	0	0	0	0	0	0	0	1	0	1	0	0	0	0	0	0	0	0	0	0	0	0	0	0	0	0	0	0	0	1	38.6419	20.7089
1387	1976/027	1	0	0	0	0	0	0	0	0	0	0	0	0	1	0	0	0	0	0	0	0	0	0	0	0	0	0	0	0	0	0	0	0	0	0	0	0	0	0	0	0	0	0	0	1	0	0	0	1	38.9877	20.783
1388	1976/028	1	0	0	1	0	0	0	0	0	0	0	0	0	0	0	0	0	0	1	0	0	0	0	0	0	0	1	0	1	0	0	0	1	0	0	0	0	0	0	0	0	0	0	0	0	0	0	0	1	38.5006	21.3674
1389	1976/029	1	0	0	1	0	0	0	0	0	0	0	0	0	1	0	0	0	0	1	0	0	0	0	0	0	0	0	0	0	0	0	0	1	0	0	0	0	0	0	0	0	0	0	0	0	0	0	0	1	38.3929	21.8259
1390	1976/030	0	0	0	0	0	0	0	0	0	0	0	0	0	0	0	0	0	0	0	0	0	0	0	0	0	0	0	0	0	0	0	0	0	0	0	0	0	0	0	0	0	0	0	0	0	0	0	0	1	38.1492	22.2296
1391	1976/031	0	0	0	0	0	0	0	0	0	0	0	0	0	0	0	0	0	0	0	0	0	0	0	0	0	0	0	0	0	0	0	0	0	0	0	0	0	0	0	0	0	0	0	0	0	0	0	0	1	38.1295	22.2172
1392	1976/032	0	0	0	0	0	0	0	0	0	0	0	0	1	0	0	0	0	0	0	0	0	0	0	0	0	0	0	0	0	0	0	0	0	0	0	0	0	0	0	0	0	0	0	0	0	0	0	0	1	38.1206	22.1719
1393	1976/033	0	0	0	0	0	0	0	0	0	0	0	0	1	0	0	0	0	0	0	0	0	0	0	0	0	0	0	0	0	0	0	0	0	0	0	0	0	0	0	0	0	0	0	0	0	0	0	0	1	38.0884	22.1746
1394	1976/034	1	0	0	0	0	0	0	0	0	1	0	0	0	1	0	0	0	0	0	0	0	0	0	0	0	0	0	0	0	0	0	0	1	0	0	0	0	0	0	0	0	0	0	0	0	0	0	0	1	38.0286	21.9965
1395	1976/035	1	0	0	0	0	0	0	0	0	0	0	0	0	0	0	0	0	0	0	0	0	0	0	0	0	0	0	0	0	0	0	0	1	0	0	0	0	0	0	0	0	0	0	0	0	0	0	0	1	38.0674	21.8513
1396	1976/036	1	0	0	0	0	0	0	0	0	0	0	0	0	1	0	0	0	0	0	0	0	0	0	0	0	0	0	0	0	0	0	0	0	0	0	0	0	0	0	0	0	0	0	0	0	0	0	0	1	37.6384	21.6307
1397	1976/037	1	0	0	0	0	0	0	0	0	0	0	0	1	1	0	0	0	0	1	0	0	0	0	0	0	0	0	0	0	0	0	0	0	0	0	0	0	0	0	0	0	0	0	0	0	0	0	0	1	37.6989	21.9392
1398	1976/038	1	0	0	0	0	0	0	0	0	1	0	0	0	0	0	0	0	0	1	0	0	0	0	0	0	0	1	0	0	0	0	0	0	0	0	0	0	0	0	0	0	0	0	0	0	0	0	0	1	37.6815	22.0147
1399	1976/039	0	0	0	0	0	0	0	0	0	0	0	0	1	0	0	0	0	0	1	0	0	0	0	0	0	0	0	0	0	0	0	0	0	0	0	0	0	0	0	0	0	0	0	0	0	0	0	0	1	37.6727	22.0534
1400	1976/040	1	0	0	0	0	0	0	0	0	0	0	0	1	0	0	0	0	0	0	0	0	0	0	0	0	0	0	0	0	0	0	0	0	0	0	0	0	0	0	0	0	0	0	0	0	0	0	0	1	37.6515	22.1685
1401	1976/041	0	0	0	0	0	0	0	0	0	0	0	0	0	0	0	0	0	0	0	0	0	0	0	0	0	0	0	0	0	0	0	0	0	0	0	0	0	0	0	0	0	0	0	0	0	0	0	0	1	37.4101	22.1273
1402	1976/042	1	0	0	0	0	0	0	0	0	0	0	0	0	0	0	0	0	0	1	0	1	0	0	0	0	0	0	0	0	0	0	0	0	0	0	0	0	0	0	0	0	0	0	0	0	0	0	0	1	37.3319	22.0743
1403	1976/043	1	0	0	0	0	0	0	0	0	0	0	0	0	1	0	0	0	0	0	0	0	0	0	0	0	0	0	0	0	0	0	0	0	0	0	0	0	0	0	0	0	0	0	0	0	0	0	0	1	37.0364	22.1663
1404	1976/044	1	0	0	0	0	0	0	0	0	1	0	0	1	1	0	0	0	0	1	0	0	0	0	0	0	0	1	0	0	0	0	0	1	0	0	0	0	0	0	0	0	0	0	0	0	0	0	0	1	37.0412	22.2001
1405	1976/045	1	0	0	0	0	0	0	0	0	1	0	0	1	0	0	0	0	0	1	0	1	0	0	0	0	0	0	0	0	0	0	0	0	0	0	0	0	0	0	0	0	0	0	0	0	0	0	0	1	37.1485	21.9418
1406	1976/046	1	0	0	0	0	0	0	0	0	0	0	0	1	1	0	0	0	0	0	0	0	0	0	0	0	0	0	0	0	0	0	0	1	0	0	0	0	0	0	0	0	0	0	0	0	0	0	0	1	37.1722	21.9179
1407	1976/047	1	0	0	0	0	0	0	0	0	0	0	0	1	1	0	0	0	0	1	0	0	0	0	0	0	0	1	0	0	0	0	0	1	0	0	0	0	0	0	0	0	0	0	0	0	0	0	0	1	36.9845	22.1619
1408	1976/048	1	0	0	0	0	0	0	0	0	0	0	0	0	1	0	0	0	0	0	0	0	0	0	0	0	0	0	0	0	0	0	0	0	0	0	0	0	0	0	0	0	0	0	0	0	0	0	0	1	36.9556	22.1913
1409	1976/049	0	0	0	0	0	0	0	0	0	0	0	0	1	0	0	0	0	0	0	0	0	0	0	0	0	0	0	0	0	0	0	0	0	0	0	0	0	0	0	0	0	0	0	0	0	0	0	0	1	36.9313	22.1915
1410	1976/050	1	0	0	0	0	0	0	0	0	1	0	0	1	1	0	0	0	0	1	0	0	0	0	0	0	0	1	0	0	0	0	0	1	0	0	0	0	0	0	0	0	0	0	0	0	0	0	0	1	37.0298	22.1865
1411	1976/051	0	0	0	0	0	0	0	0	0	0	0	0	1	0	0	0	0	0	0	0	0	0	0	0	0	0	0	0	0	0	0	0	0	0	0	0	0	0	0	0	0	0	0	0	0	0	0	0	1	37.0808	22.2753
1412	1976/052	0	0	0	0	0	0	0	0	0	1	0	0	0	1	0	0	0	0	0	0	0	0	0	0	0	0	0	0	0	0	0	0	1	0	0	0	0	0	0	0	0	0	0	0	0	0	0	0	1	37.0745	22.2955
1413	1976/053	0	0	0	0	0	0	0	0	0	0	0	0	0	1	0	0	0	0	0	0	0	0	0	0	0	0	1	0	0	0	0	0	1	0	0	0	0	0	0	0	0	0	0	0	0	0	0	0	1	37.0739	22.3691
1414	1976/054	0	0	0	0	0	0	0	0	0	0	0	0	0	0	0	0	0	0	0	0	0	0	0	0	0	0	0	0	0	0	0	0	1	0	0	0	0	0	0	0	0	0	0	0	0	0	0	0	1	37.2968	22.4175
1415	1976/055	0	0	0	0	0	0	0	0	0	0	0	0	0	0	0	0	0	0	0	0	0	0	0	0	0	0	0	0	0	0	0	0	0	0	0	0	0	0	0	0	0	0	0	0	0	0	0	0	1	37.3813	22.4034
1416	1976/056	0	0	0	0	0	0	0	0	0	0	0	0	0	0	0	0	0	0	0	0	0	0	0	0	0	0	0	0	0	0	0	0	0	0	0	0	0	0	0	0	0	0	0	0	0	0	0	0	1	37.4537	22.4235
1417	1976/057	0	0	0	0	0	0	0	0	0	0	0	0	1	0	0	0	0	0	0	0	0	0	0	0	0	0	0	0	0	0	0	0	1	0	0	0	0	0	0	0	0	0	0	0	0	0	0	0	1	37.5356	22.5136
1419	1976/059	0	0	0	0	0	1	0	0	0	0	0	0	1	1	0	0	0	0	0	0	0	0	0	0	0	0	0	0	0	0	0	0	1	0	0	0	0	0	0	0	0	0	0	0	0	0	0	0	1	37.5232	22.6404
1421	1976/061	0	0	0	0	0	1	0	0	0	0	0	0	1	1	1	0	0	0	1	0	0	0	0	0	0	0	0	0	0	0	0	0	1	0	0	0	0	0	0	0	0	0	0	0	0	0	0	0	1	37.599	22.8001
1422	1976/062	1	0	0	0	0	1	0	0	0	0	0	0	1	1	1	0	0	0	0	0	0	0	0	0	0	0	0	0	0	0	0	0	1	0	0	0	0	0	0	0	0	0	0	0	0	0	0	0	1	37.7291	22.7577
1423	1976/063	0	0	0	0	0	0	0	0	0	0	0	0	0	0	0	0	0	0	0	0	0	0	0	0	0	0	0	0	0	0	0	0	0	0	0	0	0	0	0	0	0	0	0	0	0	0	0	0	1	37.5936	22.9445
1424	1976/064	0	0	0	0	0	0	0	0	0	0	0	0	0	1	0	0	0	0	0	0	0	0	0	0	0	0	0	0	0	0	0	0	0	0	0	0	0	0	0	0	0	0	0	0	0	0	0	0	1	37.6334	23.1599
1425	1976/065	1	0	0	0	0	0	0	0	0	0	0	0	0	1	0	0	0	0	0	0	0	0	0	0	0	0	0	0	0	0	0	0	0	0	0	0	0	0	0	0	0	0	0	0	0	0	0	0	1	37.7334	23.101
1426	1976/066	1	0	0	0	0	0	0	0	0	1	0	0	1	1	0	0	0	0	1	0	0	0	0	0	0	0	1	0	0	0	0	0	0	0	0	0	0	0	0	0	0	0	0	1	0	0	0	0	1	37.9059	22.8792
1427	1976/068	0	0	0	0	0	0	0	0	0	0	0	0	0	0	0	0	0	0	0	0	0	0	0	0	0	0	0	0	0	0	0	0	0	0	0	0	0	0	0	0	0	0	0	0	0	0	0	0	1	37.9699	23.7334
1428	1976/200	0	0	0	0	0	0	0	0	0	0	0	0	1	1	0	0	0	0	0	1	0	0	0	0	0	0	0	0	0	0	0	0	0	0	0	0	0	0	0	0	1	0	0	0	0	0	0	0	1	37.983023	23.747196
1429	1976/201	0	0	0	0	0	0	0	0	0	0	0	0	1	1	0	0	0	0	0	0	0	0	0	0	0	0	0	0	0	0	1	0	0	0	0	0	0	0	0	0	0	0	0	0	0	0	0	0	1	38.168458	24.231141
1432	1981/B/003	0	0	0	0	0	1	0	0	1	1	0	0	1	1	1	1	0	0	0	0	0	0	0	0	0	0	0	0	0	0	1	0	0	0	0	0	0	0	0	0	0	0	0	0	0	0	0	0	1	41.7689	23.4267
1436	1984/A/002	0	0	0	0	0	0	0	0	0	0	0	0	0	0	1	0	0	0	1	0	0	0	0	0	0	0	0	0	0	0	0	0	0	0	0	0	0	0	0	0	0	0	0	0	0	0	0	0	1	43.61949	22.040561
1437	1984/A/003	0	0	0	0	0	1	0	0	1	0	0	0	1	0	1	1	1	0	1	0	1	0	0	0	0	0	0	0	0	0	1	0	0	0	0	0	1	0	0	0	0	0	0	0	0	0	0	0	1	43.595442	22.095047
1438	1984/A/004	0	0	0	0	0	1	0	0	0	0	0	0	1	0	1	0	0	0	1	0	0	0	0	0	0	0	0	0	0	0	0	0	0	0	1	0	0	0	0	0	0	0	0	0	0	0	0	0	1	43.340293	22.091135
1439	1984/A/005	0	0	0	0	0	0	0	0	1	0	0	0	1	0	0	0	0	0	0	0	0	0	0	0	0	0	0	0	0	0	1	0	0	0	0	0	0	0	0	0	0	0	0	0	0	0	0	0	1	41.52419	23.391398
1442	1984/A/008	0	0	0	0	0	1	0	0	1	0	1	0	1	0	1	0	0	0	0	0	1	0	0	0	0	0	0	0	0	0	1	0	0	0	0	0	0	0	0	0	0	1	0	0	0	0	0	0	1	42.175572	23.981482
1443	1984/A/009	0	0	0	0	0	0	0	0	1	0	0	0	1	0	0	0	0	0	0	0	0	0	0	0	0	0	0	0	0	0	0	0	0	0	0	0	0	0	0	0	0	1	0	0	0	0	0	0	1	42.161665	23.970234
1445	1984/A/011	0	0	0	0	0	0	0	0	1	1	0	0	1	1	1	0	0	0	0	0	0	0	0	0	0	0	0	0	0	0	1	0	0	0	0	0	0	0	0	0	0	1	0	0	0	0	0	0	1	41.683493	24.363566
1447	1984/A/013	0	0	0	0	0	1	0	0	0	0	0	0	1	0	1	0	0	0	0	0	0	0	0	0	0	0	0	0	0	0	1	0	0	0	0	0	0	0	0	0	0	0	0	0	0	0	0	0	1	41.850135	24.683023
1448	1984/A/014	0	0	0	0	0	1	0	0	0	0	0	0	1	0	1	0	0	0	1	0	0	0	0	0	0	0	0	0	0	0	1	0	0	0	0	0	0	0	0	0	0	0	0	0	0	0	0	0	1	41.841983	24.679184
1449	1984/A/015	0	0	0	0	0	1	0	0	0	0	0	0	0	0	1	0	0	0	0	0	0	0	0	0	0	0	0	0	0	0	0	0	0	0	0	0	0	0	0	0	0	0	0	0	0	0	0	0	1	41.879742	24.69584
1450	1984/A/016	1	0	0	0	0	1	0	0	1	0	0	0	1	1	1	0	0	0	1	0	1	0	0	0	0	0	0	0	0	0	1	0	0	0	0	0	0	0	0	0	0	0	0	0	0	0	0	0	1	41.941993	24.849907
1451	1984/A/017	1	1	1	0	1	1	0	0	1	1	1	0	1	0	0	0	0	0	0	0	1	0	0	0	1	1	1	0	0	0	1	0	0	0	0	0	0	0	0	0	0	0	0	0	0	0	0	0	1	41.934101	24.849523

1453	1984/A/019	1	0	0	0	0	1	0	0	0	0	0	0	1	0	1	0	0	0	0	0	0	0	0	0	0	0	0	0	0	1	0	0	0	0	0	0	0	0	0	0	0	0	0	0	0	0	0	1	42.685732	26.356405
1454	1984/A/020	1	0	0	0	0	1	0	0	0	0	0	0	0	1	1	0	0	0	0	0	0	0	0	0	0	0	0	0	0	1	0	0	0	0	0	0	0	0	0	0	0	0	0	0	0	0	0	1	42.701669	26.350307
1465	1984/A/031	1	0	0	0	1	0	0	0	1	0	1	0	1	1	1	0	1	1	1	0	0	0	1	0	1	1	0	0	0	1	0	0	0	0	0	0	0	0	0	0	0	0	0	0	0	0	0	1	42.957798	25.432085
1466	1984/A/032	0	0	0	0	0	0	0	0	0	0	0	0	0	0	0	0	0	0	0	0	0	0	0	0	0	0	0	0	0	1	0	0	0	0	0	0	0	0	0	0	1	0	0	0	0	0	0	1	42.748159	25.321604
1467	1984/A/033	1	0	0	0	0	1	1	0	1	1	0	0	1	0	1	1	1	0	1	0	1	0	1	0	0	1	0	0	0	1	0	0	0	0	0	0	0	0	0	0	1	0	0	0	0	0	0	1	41.818016	24.589782
1469	1984/A/035	0	0	0	0	0	1	0	0	0	0	0	0	0	0	1	0	0	0	1	0	0	0	0	0	0	0	0	0	0	1	0	0	0	0	0	0	0	0	0	0	0	0	0	0	0	0	0	1	41.868899	24.691987
1470	1984/A/036	0	0	0	0	0	1	0	0	1	1	0	0	1	1	1	0	0	0	0	0	0	0	0	0	0	0	0	0	0	1	0	0	0	0	0	0	0	0	0	0	0	0	0	0	0	0	0	1	41.987505	24.875516
1471	1984/A/037	0	0	0	0	0	1	0	0	0	0	0	0	0	1	0	0	0	0	0	0	0	0	0	0	0	0	0	0	0	1	0	0	0	0	0	0	0	0	0	0	0	0	0	0	0	0	0	1	43.071718	23.398599
1475	1984/A/041	0	0	0	0	0	0	0	0	0	0	0	0	1	0	0	0	0	0	0	0	0	0	0	0	0	0	0	0	0	0	0	0	0	0	0	0	0	0	0	0	0	0	0	0	0	0	0	1	43.621563	22.684626
1476	1984/A/042	1	0	0	0	0	1	0	0	1	0	0	0	1	1	1	0	0	0	1	0	1	0	1	0	0	0	0	0	0	1	0	0	0	0	0	1	0	0	0	0	1	0	0	0	0	0	0	1	43.651163	22.693433
1477	1984/A/043	1	0	0	0	0	1	0	0	1	0	0	0	1	1	1	1	0	0	1	0	1	0	1	0	0	0	0	0	0	1	0	0	0	0	0	1	0	0	0	0	1	0	0	0	0	0	0	1	43.656246	22.716653
1478	1984/A/044	0	0	0	0	0	0	0	0	0	0	0	0	0	0	1	0	0	0	0	0	0	0	0	0	0	0	0	0	0	1	0	0	0	0	0	0	0	0	0	0	0	0	0	0	0	0	0	1	43.992936	22.886648
1479	1984/A/045	0	0	0	0	0	1	0	0	0	0	0	0	0	0	1	0	0	0	1	0	0	0	0	0	0	0	0	0	0	0	0	0	0	0	0	0	0	0	0	0	0	0	0	0	0	0	0	1	43.727011	22.583722
1480	1984/A/046	0	0	0	0	0	1	0	0	0	0	0	0	1	0	1	0	0	0	0	0	0	0	0	0	0	0	0	0	0	0	0	0	0	0	0	0	0	0	0	0	0	0	0	0	0	0	0	1	43.73204	22.577151
1481	1984/A/047	1	0	0	0	1	1	0	0	1	0	0	0	1	0	1	1	0	0	1	0	1	0	0	0	0	0	0	0	0	1	0	0	0	0	0	1	0	0	1	0	0	0	0	0	0	0	0	1	44.029196	21.96296
1482	1984/A/048	0	0	0	0	0	1	0	0	1	0	0	0	1	0	1	0	0	0	1	0	1	0	0	0	0	0	0	0	0	1	0	0	0	0	0	0	0	0	0	0	0	0	0	0	0	0	0	1	43.856	21.627
1483	1985/C/001	0	0	0	0	0	0	0	0	0	0	0	0	0	0	1	0	0	0	0	0	0	0	0	0	0	0	0	0	0	1	0	0	0	0	0	0	0	0	0	0	0	0	0	0	0	0	0	1	44.1834	20.550021
1487	1985/C/005	0	0	0	0	0	1	0	0	0	0	0	0	1	1	1	0	0	0	0	0	0	0	0	0	0	1	0	0	0	1	0	0	0	0	0	0	0	0	0	0	0	0	0	0	0	0	0	1	41.891847	21.679516
1490	1985/C/008	1	0	0	0	0	1	0	0	1	0	0	0	1	1	0	1	0	0	0	0	0	0	0	0	0	0	0	0	0	0	0	0	0	0	0	0	0	0	0	0	0	0	0	0	0	0	0	1	40.944085	20.789567
1494	1985/C/012	1	0	0	0	0	0	0	0	0	0	0	0	0	1	1	0	0	0	0	0	0	0	0	0	0	0	0	0	0	0	0	0	0	0	0	0	0	0	0	0	0	0	0	0	0	0	1	1	40.993184	20.800593
1495	1985/C/013	0	0	0	0	0	1	0	0	0	0	0	0	1	0	1	0	0	0	0	0	0	0	0	0	0	0	0	0	0	0	0	0	0	0	0	0	0	0	0	0	0	0	0	0	0	0	1	1	41.358007	20.606138
1498	1985/C/016	0	0	0	0	0	1	0	0	1	1	0	0	1	0	1	1	0	0	1	0	1	0	0	0	1	1	0	0	0	1	0	0	0	0	0	1	0	0	0	0	0	0	0	0	0	0	0	1	41.71	20.694
1499	1985/C/017	0	0	0	0	0	0	0	0	0	0	0	0	1	0	1	0	0	0	0	0	0	0	0	0	0	0	0	0	0	1	0	0	0	0	0	1	0	0	0	0	0	0	0	0	0	0	0	1	42.692118	20.084411
1501	1985/C/019	0	0	0	0	0	0	0	0	0	0	0	0	1	0	1	0	0	0	0	0	0	0	0	0	0	0	0	0	0	1	0	0	0	0	0	0	0	0	1	0	0	0	0	0	0	0	0	1	42.842122	19.870445
1502	1985/C/020	0	0	0	0	0	1	0	0	0	0	0	0	0	0	0	0	0	0	1	0	0	0	0	0	0	0	0	0	0	0	0	0	0	0	0	0	0	0	0	0	0	0	0	0	0	0	0	1	42.943649	19.57763
1503	1985/C/021	1	0	0	0	0	0	0	0	0	0	0	0	1	0	1	0	0	0	0	0	0	0	0	0	0	0	0	0	0	0	0	0	0	0	0	0	0	0	0	0	0	0	0	0	0	0	0	1	42.23209	19.025966
1504	1985/C/022	0	0	0	0	0	1	0	0	0	0	0	0	0	0	1	0	0	0	0	0	0	0	0	0	0	0	0	0	0	0	0	0	0	0	0	0	1	0	0	0	0	0	0	0	0	0	0	1	42.214829	18.95099
1505	1985/C/023	1	0	0	1	0	1	0	0	0	1	0	0	1	0	1	0	0	0	1	0	0	0	0	0	0	0	0	1	1	0	0	0	0	0	0	0	0	0	0	0	0	0	1	0	0	0	1	1	42.04756	19.136
1508	1985/C/026	1	0	0	0	0	0	0	0	0	0	0	0	0	0	0	0	0	0	1	0	0	0	0	0	0	0	0	0	0	0	0	0	0	0	0	0	1	0	0	0	0	0	1	0	0	0	0	1	42.053714	19.348619
1510	1985/C/028	1	0	0	0	0	0	0	0	0	0	0	0	0	0	1	0	0	0	0	0	0	0	0	0	0	0	0	1	0	0	0	0	0	0	0	0	1	0	0	0	0	0	0	0	0	0	1	1	42.431962	19.254932
1511	1985/C/029	1	0	0	0	0	0	0	0	0	0	0	0	1	0	1	0	0	0	0	0	0	0	0	0	0	0	0	1	1	0	0	0	0	0	0	0	0	0	0	0	0	0	0	0	0	0	1	1	42.390161	19.11594
1512	1985/C/030	1	0	0	0	0	0	0	0	0	0	0	0	0	0	0	0	0	0	0	0	0	0	0	0	0	0	0	0	0	0	0	0	0	0	0	0	0	0	0	0	0	0	0	0	0	0	1	1	42.379008	18.997754
1513	1985/C/031	1	0	0	0	0	1	0	0	0	0	0	0	1	0	1	0	0	0	0	0	0	0	0	0	0	0	0	1	1	0	0	0	0	0	0	0	0	0	0	0	0	0	0	0	0	0	0	1	42.433791	18.810255
1514	1985/C/032	1	0	0	0	0	1	0	0	0	0	0	0	0	0	1	0	0	0	0	0	0	0	0	0	0	0	0	1	0	0	1	0	0	0	0	0	1	0	0	0	0	0	0	0	0	0	1	1	42.423762	18.800793
1517	1985/C/036	1	0	0	0	0	0	0	0	1	0	0	0	0	0	1	0	0	0	0	0	0	0	0	0	0	0	0	0	1	0	0	0	0	0	0	0	1	0	0	0	0	0	0	0	0	0	0	1	42.845041	17.705071
1518	1985/C/037	0	0	0	0	0	0	0	0	0	0	0	0	1	0	0	0	0	0	1	0	0	0	0	0	0	0	0	0	0	0	1	0	0	0	0	0	1	0	0	0	0	0	0	0	0	0	0	1	42.876242	17.528588
1519	1985/C/038	1	0	0	0	0	0	0	0	0	0	0	0	1	0	1	0	0	0	0	0	0	0	0	0	0	0	0	1	1	0	0	0	0	0	0	0	1	0	0	0	0	0	0	0	0	0	0	1	42.919166	17.423351
1521	1985/C/040	1	0	0	0	0	0	0	0	0	0	0	0	1	0	1	0	0	0	1	0	0	0	0	0	0	0	0	1	1	0	0	0	0	0	0	0	0	0	0	0	0	0	0	0	0	0	0	1	42.9655	17.309
1522	1985/C/041	1	0	0	0	0	0	0	0	0	0	0	0	1	0	1	0	0	0	1	0	0	0	0	0	0	0	0	1	1	0	0	0	0	0	0	0	1	0	0	0	0	0	0	0	0	0	0	1	43.003484	17.083073
1524	1985/C/043	1	0	0	1	0	0	0	0	0	0	0	0	1	0	1	0	0	0	0	0	0	0	0	0	0	0	0	1	1	0	1	0	0	0	0	0	1	0	0	0	0	0	0	0	0	0	0	1	42.719076	17.657937
1525	1985/C/044	1	0	0	1	0	0	0	0	0	0	0	0	1	0	1	0	0	0	0	0	0	0	0	0	0	0	0	1	1	0	0	0	0	0	0	0	1	0	0	0	0	0	0	0	0	0	0	1	42.736467	17.599828
1527	1985/C/046	0	0	0	0	0	0	0	0	0	0	0	0	1	0	0	0	0	0	0	0	0	0	0	0	0	0	0	0	1	0	1	0	0	0	0	0	1	0	0	0	0	0	0	0	0	0	0	1	42.903278	17.640621
1528	1985/C/047	1	0	0	1	0	0	0	0	0	0	0	0	1	0	1	0	0	0	1	0	0	0	0	0	0	0	0	1	1	0	1	0	0	0	0	0	1	0	0	0	0	0	0	0	0	0	0	1	42.969128	17.529852
1529	1985/C/048	0	0	0	0	0	0	0	0	0	0	0	0	0	0	1	0	0	0	0	0	0	0	0	0	0	0	0	0	0	0	1	0	0	0	0	0	1	0	0	0	0	0	0	0	0	0	0	1	43.046437	17.475945
1530	1985/C/049	0	0	0	0	0	0	0	0	0	0	0	0	0	0	1	0	0	0	0	0	0	0	0	0	0	0	0	0	0	0	0	0	0	0	0	0	1	0	0	0	0	0	0	0	0	0	0	1	43.156082	17.246553
1531	1985/C/050	0	0	0	0	0	0	0	0	0	0	0	0	1	0	0	0	0	0	1	0	0	0	0	0	0	0	0	0	1	0	0	0	0	0	0	0	0	0	0	0	0	1	0	0	0	0	0	1	43.368441	16.935677
1534	1985/C/054	1	0	0	0	0	0	0	0	0	0	0	0	0	0	0	0	0	0	1	0	0	0	0	0	0	0	0	0	0	0	0	0	0	0	0	0	1	0	0	0	0	1	0	0	0	0	0	1	43.41601	16.907626
1535	1985/C/055	1	0	0	1	0	0	0	0	0	0	0	0	0	0	0	0	0	0	1	0	0	0	0	0	0	0	0	1	0	0	1	0	0	0	0	0	1	0	0	0	0	0	0	0	0	0	0	1	43.296438	17.861796
1536	1985/C/056	1	0	0	0	0	0	0	0	0	0	0	0	1	0	0	0	0	0	1	0	0	0	0	0	0	0	0	1	0	0	0	0	0	0	0	0	0	0	0	0	0	0	0	0	0	0	0	1	43.281596	18.207305
1537	1985/C/057	0	0	0	0	0	1	0	0	0	0	0	0	0	0	1	0	0	0	1	0	0	0	0	0	0	0	0	0	0	0	0	0	0	0	0	0	1	0	0	0	0	0	0	0	0	0	0	1	43.2824	18.262585
1539	1985/C/059	0	0	0	0	0	1	0	0	0	0	0	0	1	0	1	0	0	0	0	0	1	0	0	0	0	0	0	0	0	0	0	0	0	0	0	0	1	0	0	0	0	0	0	0	0	0	0	1	43.244063	18.606136
1542	1985/C/062	0	0	0	0	0	0	0	0	0	0	0	0	1	0	1	0	0	0	1	0	0	0	0	0	0	0	0	0	1	0	0	0	0	0	0	0	0	0	1	0	0	0	0	0	0	0	0	1	43.293596	18.851268
1543	1985/C/063	0	0	0	0	0	0	0	0	0	0	0	0	1	0	1	0	0	0	1	0	0	0	0	0	0	0	0	0	0	0	0	0	0	0	0	0	0	0	1	0	0	0	0	0	0	0	0	1	43.210107	18.858728
1544	1985/C/064	1	0	0	0	0	1	0	0	0	0	0	0	1	0	1	0	0	0	0	0	0	0	0	0	0	0	0	1	1	0	0	0	0	0	0	0	1	0	0	0	0	0	0	0	0	0	0	1	42.795624	18.96801
1545	1985/C/065	1	0	0	0	0	0	0	0	0	0	0	0	1	0	0	0	0	0	1	0	0	0	0	0	0	0	0	0	0	0	0	0	0	0	0	0	1	0	1	0	0	0	0	0	0	0	0	1	42.862667	19.070286
1548	1985/C/067	0	0	0	0	0	0	0	0	0	0	0	0	0	0	1	0	0	0	1	0	0	0	0	0	0	0	0	0	1	0	0	0	0	0	0	0	0	0	1	0	0	0	0	0	0	0	0	1	43.03511	19.392942
1549	1985/C/068	0	0	0	0	0	0	0	0	0	0	0	0	0	0	0	0	0	0	1	0	0	0	0	0	0	0	0	0	0	0	0	0	0	0	0	0	0	0	0	0	0	0	0	0	0	0	0	1	42.84201	20.111764
1550	1985/C/069	0	0	0	0	0	1	0	0	0	0	0	0	0	0	0	0	0	0	1	0	0	0	0	0	0	0	0	0	0	1	0	0	0	0	0	0	0	0	1	0	0	0	0	0	0	0	0	1	43.131007	20.421484
1551	1985/C/070	1	0	0	0	0	0	0	0	0	0	0	0	1	0	1	0	0	0	0	0	0	0	0	0	0	0	0	0	0	0	0	0	0	0	0	0	0	0	1	0	0	0	0	0	0	0	0	1	43.755724	19.232471
1553	1985/C/072	0	0	0	0	0	0	0	0	0	0	0	0	1	0	1	0	0	0	1	0	0	0	0	0	0	0	0	0	0	0	0	0	0	0	0	0	0	0	0	0	0	0	0	0	0	0	0	1	43.851662	18.515263
1554	1985/C/073	1	0	0	0	0	0	0	0	0	1	0	0	1	0	1	0	0	0	0	0	0	0	0	0	0	0	0	0	1	1	0	0	0	0	0	1	0	0	0	0	0	0	0	0	0	0	0	1	43.736487	18.565115
1557	1985/C/076	1	0	0	0	0	0	0	0	0	0	1	0	1	0	1	0	0	0	0	0	0	0	0	0	0	0	0	0	1	1	0	0	0	0	0	0	0	0	0	0	0	0	0	0	0	0	0	1	44.337867	17.270223
1561	1986/E/004	1	0	1	0	0	1	0	0	0	1	0	0	1	1	1	0	0	0	0	0	0	0	0	0	0	0	0	0	0	1	0	0	0	1	0	0	0	0	0	0	0	0	0	0	0	0	0	1	41.171945	24.323948
1562	1986/E/005	0	0	0	0	0	1	0	0	0	0	0	0	1	1	1	0	0	0	0	0	0	0	0	0	0	0	0	0	0	0	0	0	0	0	0	0	0	0	0	0	0	0	0	0	0	0	0	1	41.227009	24.607051
1563	1986/E/006	1	0	0	0	0	1	0	0	0	0	0	0	1	1	1	0	0	0	0	0	0	0	0	0	0	0	0	0	0	1	0	0	0	1	0	0	0	0	0	0	0	0	0	0	0	0	0	1	41.203751	24.723207
1565	1986/E/008	1	0	0	0	0	0	0	0	0	0	0	0	0	1	1	0	0	0	0	0	0	0	0	0	0	0	0	0	0	0	0	0	0	0	0	0	0	0	0	0	0	0	0	0	0	0	0	1	40.780752	24.681705
1566	1986/E/009	1	0	0	0	0	1	0	0	0	0	0	0	0	1	1	0	0	0	0	0	0	0	0	0	0	0	0	0	0	1	0	0	0	1	0	0	0	0	0	0	0	0	0	0	0	0	0	1	40.644632	24.51576
1567	1986/E/010	0	0	1	0	0	1	0	0	1	1	0	0	1	1	1	0	0	0	1	1	0	1	0	0	0	1	0	0	0	1	0	0	0	1	0	0	0	0	0	0	0	0	0	0	0	0	0	1	40.660941	24.691194
1568	1986/E/011	1	0	0	0	0	0	0	0	0	0	0	0	1	1	1	0	0	0	0	0	0	0	0	0	0	0	0	0	0	0	0	0	0	0	0	0	0	0	0	0	0	0	0	0	0	0	0	1	40.603636	24.732485
1569	1986/E/012	1	0	0	0	0	1	0	0	1	0	0	0	1	1	1	0	0	0	0	0	0	0	0	0	0	0	0	0	0	1	0	0	0	1	0	0	0	0	0	0	0	0	0	0	0	0	0	1	41.082345	24.760755
1570	1986/E/013	1	0	0	0	0	1	1	0	1	1	0	0	1	1	1	0	1	0	1	1	0	1	1	0	0	1	0	0	0	1	0	0	0	1	0	0	0	0	0	0	0	0	0	0	0	0	0	1	41.02333333	24.64178333
1571	1986/E/014	1	0	0	0	0	1	0	0	0	0	0	0	1	1	1	0	0	0	0	0	0	0	0	0	0	0	0	0	0	1	0	0	0	1	0	0	0	0	0	0	0	0	0	0	0	0	0	1	41.012068	24.28474
1574	1986/E/018	1	0	0	0	0	0	0	0	0	1	0	0	1	1	1	0	0	0	1	1	0	0	1	0	0	1	0	0	0	1	0	0	0	1	0	0	0	0	0	0	0	0	0	0	0	0	0	1	39.3076	22.404957
1575	1986/E/019	0	0	0	0	0	0	0	0	0	0	0	0	0	1	1	0	0	0	0	0	0	0	0	0	0	0	0	0	0	0	0	0	0	0	0	0	0	0	0	0	0	0	0	0	0	0	0	1	38.796728	22.536564
1576	1986/E/020	0	0	0	0	0	1	0	0	0	0	0	0	1	0	1	0	0	0	0	0	0	0	0	0	0	0	0	0	0	0	0	0	0	0	0	0	0	0	0	1	0	0	0	0	0	0	0	1	38.493601	23.160416
1577	1986/E/021	0	0	0	0	0	1	0	0	0	0	0	0	1	1	1	0	0	0	0	0	0	0	0	0	0	0	0	0	0	1	0	0	0	0	0	0	0	0	0	1	0	0	0	0	0	0	0	1	38.475762	23.164042
1579	1986/E/075	0	0	0	0	0	0	0	0	0	0	0	0	1	1	1	0	0	0	0	0	0	0	0	0	0	0	0	0	0	0	0	0	0	0	0	0	0	0	0	0	0	0	0	0	0	0	0	1	37.989281	23.337772
1580	1986/E/076	0	0	0	0	0	1	0	0	0	0	0	0	0	0	1	0	0	0	0	0	0	0	0	0	0	0	0	0	0	0	0	0	0	0	0	0	0	0	0	1	0	0	0	0	0	0	0	1	37.977354	23.260457
1581	1986/E/077	0	0	0	0	0	1	0	0	0	0	0	0	1	1	1	0	0	0	0	0	0	0	0	0	0	0	0	0	0	0	0	1	0	0	0	0	0	0	0	1	0	0	0	0	0	0	0	1	37.974496	22.977521
1582	1986/E/078	1	0	0	0	0	0	0	0	0	0	0	0	0	0	0	0	0	0	0	0	0	0	0	0	0	0	0	0	0	0	0	1	0	0	0	0	0	0	0	0	0	0	0	0	0	0	0	1	37.832427	23.025536
1583	1986/E/079	1	0	0	0	0	0	0	0	0	0	0	0	1	1	1	0	0	0	0	0	0	0	0	0	0	0	0	0	0	0	0	1	0	0	0	0	0	0	0	0	0	0	0	0	0	0	0	1	37.742248	23.114409
1584	1986/E/080	0	0	1	0	0	1	0	0	0	1	0	0	1	1	1	0	0	0	1	0	0	0	1	0	0	1	0	0	0	0	0	1	0	0	0	0	0	0	0	0	0	0	0	0	0	0	0	1	37.56682	22.801439
1587	1986/E/083	0	0	0	0	0	1	0	0	0	0	0	0	1	1	0	0	0	0	0	0	0	0	0	0	0	0	0	0	0	0	0	1	0	0	0	0	0	0	0	0	0	0	0	0	0	0	0	1	37.533663	22.563775
1588	1986/E/084	0	0	0	0	0	0	0	0	0	0	0	0	1	1	1	0	0	0	0	0	0	0	0	0	0	0	0	0	0	0	0	1	0	0	0	0	0	0	0	0	0	0	0	0	0	0	0	1	36.844295	22.605407
1589	1986/E/085	0	0	0	0	0	1	0	0	0	0	0	0	1	1	1	0	0	0	0	0	0	0	0	0	0	0	0	0	0	0	0	1	0	0	0	0	0	0	0	0	0	0	0	0	0	0	0	1	36.687741	23.052125
1590	1986/E/086	0	0	0	0	0	1	0	0	0	0	0	0	0	1	1	0	0	0	0	0	0	0	0	0	0	0	0	1	1	0	0	1	0	0	0	0	0	0	0	0	0	0	0	0	0	0	0	1	36.674475	23.036393
1591	1986/E/087	0	0	0	0	0	0	0	0	0	0	0	0	0	1	1	0	0	0	0	0	0	0	0	0	0	0	0	0	0	0	0	1	0	0	0	0	0	0	0	0	0	0	0	0	0	0	0	1	36.692747	23.031056
1592	1986/E/088	0	0	0	0	0	1	0	0	0	0	0	0	0	1	1	0	0	0	0	0	0	0	0	0	0	0	0	0	0	0	0	1	0	0	0	0	0	0	0	0	0	0	0	0	0	0	0	1	36.682394	22.41508
1593	1986/E/089	0	0	0	0	0	0	0	0	1	0	0	0	0	1	1	0	0	0	0	0	0	0	0	0	0	0	0	1	1	0	0	1	0	0	0	0	0	0	0	0	0	0	0	0	0	0	0	1	36.625884	22.381854
1594	1986/E/090	0	0	0	0	0	0	0	0	0	0	0	0	0	1	1	0	0	0	0	0	0	0	0	0	0	0	0	1	1	0	0	1	0	0	0	0	0	0	0	0	0	0	0	0	0	0	0	1	36.483369	22.402312
1595	1986/E/091	0	0	0	0	0	0	0	0	0	0	0	0	0	0	1	0	0	0	0	0	0	0	0	0	0	0	0	1	1	0	0	1	0	0	0	0	0	0	0	0	0	0	0	0	0	0	0	1	36.452475	22.464682
1596	1986/E/092	0	0	0	0	0	0	0	0	0	0	0	0	0	1	0	0	0	0	0	0	0	0	0	0	0	0	0	0	0	0	0	1	0	0	0	0	0	0	0	0	0	0	0	0	0	0	0	1	37.068759	22.375788
1597	1986/E/093	0	0	0	0	0	0	0	0	0	0	0	0	1	0	1	0	0	0	0	0	0	0	1	0	0	0	0	1	0	0	0	1	0	0	0	0	0	0	0	0	0	0	0	0	0	0	0	1	37.092664	22.352155
1598	1986/E/094	0	0	0	0	0	0	0	0	0	0	0	0	0	0	0	0	0	0	0	0	0	0	0	0	0	0	0	0	0	0	0	1	0	0	0	0	0	0	0	0	0	0	0	0	0	0	0	1	37.095257	22.333108
1599	1986/E/095	0	0	0	0	0	0	0	0	0	0	0	0	1	0	1	0	0	0	0	0	0	0	0	0	0	0	0	0	0	0	0	1	0	0	0	0	0	0	0	0	0	0	0	0	0	0	0	1	37.091672	22.324695
1600	1986/E/096	0	0	0	0	0	0	0	0	0	0	0	0	0	0	0	0	0	0	0	0	0	0	0	0	0	0	0	0	0	0	0	1	0	0	0	0	0	0	0	0	0	0	0	0	0	0	0	1	37.099391	22.232647
1601	1986/E/097	1	0	0	0	0	1	0	0	0	0	0	0	1	1	1	0	0	0	0	0	0	0	0	0	0	0	0	1	1	0	0	1	0	0	0	0	0	0	0	0	0	0	0	0	0	0	0	1	37.186082	21.924932
1602	1986/E/098	1	0	0	0	0	1	0	0	0	0	0	0	0	1	0	0	0	0	0	0	0	0	0	0	0	0	0	0	0	0	0	1	0	0	0	0	0	0	0	0	0	0	0	1	0	0	0	1	37.63755	21.629628
1603	1986/E/099	0	0	0	0	0	0	0	0	0	0	0	1	1	0	1	0	0	0	0	0	0	0	0	0	0	0	0	0	0	0	0	0	0	0	0	0	0	0	0	0	0	0	0	0	0	0	0	1	38.311419	21.78118
1604	1986/E/100	1	0	0	0	0	1	0	0	1	0	0	0	1	1	1	0	0	0	0	0	0	0	0	0	0	0	0	0	0	0	0	1	0	0	0	0	0	0	0	0	0	0	0	0	0	0	0	1	38.376707	22.080272
1605	1986/E/101	1	0	0	0	0	1	0	0	0	0	0	0	1	1	1	0	0	0	1	0	0	0	0	0	0	0	0	0	0	0	0	0	0	0	0	0	0	0	0	1	0	0	0	0	0	0	0	1	38.482236	22.501286
1606	1986/E/102	0	0	0	0	0	1	0	0	0	0	0	0	0	1	1	0	0	0	0	0	0	0	0	0	0	0	0	0	0	0	0	0	0	0	0	0	0	0	0	1	0	0	0	0	0	0	0	1	38.555217	22.406658
1607	1986/E/103	1	0	0	0	0	0	0	0	0	0	0	0	1	0	0	0	0	0	0	0	0	0	0	0	0	0	0	0	0	0	0	1	0	0	0	0	0	0	0	1	0	0	0	0	0	0	0	1	38.57392	22.348678
1609	1986/E/105	1	0	0	0	0	1	0	0	0	0	0	0	1	0	1	0	0	0	0	0	0	0	0	0	0	0	0	1	0	0	0	1	0	0	0	0	0	0	0	0	0	0	0	0	1	0	0	1	39.68131	20.898251
1610	1986/E/106	1	0	0	0	0	1	0	0	1	0	0	0	1	1	1	1	0	0	0	0	0	0	0	0	0	0	0	0	0	0	0	0	0	0	0	0	0	0	0	0	0	0	0	0	1	0	0	1	39.784415	20.738764
1611	1986/E/107	1	0	0	0	0	1	0	0	1	0	0	0	0	0	1	0	0	0	0	0	0	0	0	0	0	0	0	0	0	0	0	0	0	0	0	0	0	0	0	0	0	0	0	0	1	0	0	1	40.050345	20.749602
1614	1987/D/001	1	0	0	0	0	0	0	0	1	0	0	0	1	0	1	0	0	0	1	0	0	0	0	0	0	0	0	0	1	1	0	0	0	0	0	1	0	0	0	0	0	0	0	0	0	0	0	1	46.369673	14.100606
1615	1987/D/002	1	0	0	0	0	0	0	0	1	0	0	0	1	0	0	0	0	0	0	0	0	0	0	0	0	0	0	0	0	1	0	0	0	0	0	1	0	0	0	0	0	0	0	0	0	0	0	1	46.298636	13.943756
1616	1987/D/003	1	0	0	0	0	0	0	0	0	0	0	0	1	0	1	0	0	0	1	0	0	0	0	0	0	0	0	0	0	0	0	0	0	0	0	1	0	0	0	0	0	0	0	0	0	0	0	1	46.296556	13.961751
1617	1987/D/004	0	0	0	0	0	0	0	0	1	0	0	0	0	0	1	0	0	0	0	0	0	0	0	0	0	0	0	0	1	1	0	0	0	0	0	1	0	0	0	0	0	0	0	1	0	0	0	1	46.323938	14.596928
1618	1987/D/005	1	0	0	0	0	0	1	0	1	1	0	0	1	0	1	0	0	0	0	0	1	0	0	0	0	0	0	0	1	1	0	0	0	0	0	1	0	0	0	0	0	0	0	0	0	0	0	1	46.327317	14.589992
1619	1987/D/006	0	0	0	0	0	0	0	0	0	0	0	0	0	0	1	0	0	0	0	0	0	0	0	0	0	0	0	0	1	1	0	0	0	0	0	0	0	0	0	0	0	0	0	0	0	0	0	1	46.303703	14.657447
1620	1987/D/007	0	0	0	0	0	0	0	0	0	0	0	0	0	0	1	0	0	0	0	0	0	0	0	0	0	0	0	0	0	0	0	0	0	0	0	1	0	0	0	0	0	0	0	0	0	0	0	1	46.298533	14.643751
1621	1987/D/008	1	0	0	0	0	0	0	0	0	0	0	0	1	0	1	0	0	0	0	0	0	0	0	0	0	0	0	0	0	1	0	0	0	0	0	1	0	0	0	0	0	0	0	1	0	0	0	1	46.485119	13.785666
1622	1987/D/009	1	0	0	0	0	0	0	0	0	0	1	0	1	0	1	0	0	0	0	0	0	0	0	0	0	0	0	0	1	1	0	0	0	0	0	0	0	0	0	0	0	0	0	0	0	0	0	1	46.280004	13.551622
1623	1987/D/010	1	0	0	0	0	0	0	0	1	1	0	0	1	1	1	1	0	0	1	0	1	0	0	0	0	0	0	0	1	1	0	0	0	0	0	1	0	0	0	0	0	0	0	0	0	0	0	1	46.108894	13.900068
1624	1987/D/011	1	0	0	0	0	0	0	0	1	1	0	0	1	0	1	0	0	0	0	0	0	0	0	0	0	0	0	0	1	1	0	0	0	0	0	1	0	0	0	0	0	0	0	0	0	0	0	1	45.820166	14.290147
1625	1987/D/012	1	0	0	0	0	0	0	0	1	0	0	0	1	0	1	0	0	0	1	0	0	0	0	0	0	0	0	0	1	1	0	0	0	0	0	1	0	0	0	0	0	0	0	0	0	0	0	1	45.819192	14.236644
1626	1987/D/013	1	0	0	0	0	0	0	0	0	0	0	0	1	0	1	0	0	0	1	0	0	0	0	0	0	0	0	1	0	0	0	0	0	0	0	0	0	0	0	0	0	0	0	0	0	0	0	1	45.294283	13.598113
1627	1987/D/014	1	0	0	0	0	0	0	0	0	0	1	0	1	0	1	0	0	0	1	0	0	0	0	0	0	0	0	1	0	1	0	0	0	0	0	0	0	0	0	0	0	0	0	0	0	0	0	1	45.364321	13.758298
1628	1987/D/015	1	0	0	0	0	1	0	0	0	1	0	0	1	0	1	0	0	0	1	1	0	0	1	0	0	0	0	1	0	1	0	0	0	0	0	0	0	0	0	0	0	1	0	0	0	0	0	1	45.377726	13.883069
1629	1987/D/016	1	0	0	0	0	0	0	0	0	0	0	0	0	0	0	0	0	0	1	0	0	0	0	0	0	0	0	1	0	0	0	0	0	0	0	0	0	0	0	0	0	0	0	0	0	0	0	1	45.086872	14.088397
1630	1987/D/017	0	0	0	0	0	1	0	0	0	0	0	0	0	0	1	0	0	0	1	1	0	0	0	0	0	0	0	1	0	0	0	0	0	0	0	0	0	0	0	0	0	0	0	1	0	0	0	1	45.521615	13.869281
1631	1987/D/018	1	0	1	0	0	1	1	0	0	1	0	0	1	0	1	0	1	0	1	1	0	0	1	0	1	1	0	1	0	1	0	0	0	0	0	1	0	0	0	0	0	1	0	0	0	0	0	1	45.463534	13.89747
1634	1987/D/021	0	0	0	0	0	0	0	0	0	0	0	0	1	0	1	0	0	0	0	0	0	0	0	0	0	0	0	0	1	0	0	0	0	0	0	0	0	0	0	0	0	0	0	0	0	0	0	1	44.52652	15.091466
1635	1987/D/022	1	0	0	0	0	0	0	0	0	0	0	0	0	0	0	0	0	0	1	0	0	0	0	0	0	0	0	0	0	0	0	0	0	0	0	0	1	0	0	0	0	1	0	0	0	0	0	1	44.526272	15.171664
1636	1987/D/023	1	0	0	0	0	0	0	0	0	0	0	0	1	0	0	0	0	0	1	0	0	0	0	0	0	0	0	0	1	0	0	0	0	0	0	0	1	1	0	0	0	0	0	0	0	0	0	1	44.248375	15.809073
1637	1987/D/024	1	0	0	0	0	0	0	0	0	0	0	0	1	0	0	0	0	0	1	0	0	0	0	0	0	0	0	1	0	0	0	0	0	0	0	0	0	0	0	0	0	0	0	0	0	0	0	1	44.220206	15.736781
1639	1987/D/026	1	0	0	0	0	1	0	0	0	1	0	0	1	0	1	0	0	0	1	0	0	0	0	0	0	0	0	0	1	0	0	0	0	0	0	1	0	0	0	0	0	0	0	1	0	0	0	1	45.265949	15.223881
1640	1987/D/027	1	0	0	0	0	0	0	0	0	0	0	0	0	0	1	0	0	0	0	0	0	0	0	0	0	0	0	0	0	0	0	0	0	0	0	0	0	0	0	0	0	0	0	1	0	0	0	1	45.227777	15.040426
1641	1987/D/028	1	0	0	0	1	0	0	0	1	0	0	0	0	0	1	1	0	0	0	0	1	0	1	0	1	1	0	0	1	1	0	0	0	0	0	1	0	0	0	0	0	0	0	0	0	0	0	1	44.99815	15.131953
1642	1987/D/029	1	0	0	0	0	0	0	0	0	0	0	0	1	0	0	0	0	0	0	1	0	0	0	0	0	0	0	1	0	0	0	0	0	0	0	0	0	0	0	0	0	1	0	0	0	0	0	1	44.985169	14.923147
1644	1988/C/062	0	0	0	0	0	0	0	0	0	0	0	0	0	1	0	0	0	0	0	0	0	0	0	0	0	0	0	0	0	0	0	0	0	0	0	0	0	0	0	0	0	0	0	0	0	0	0	1	38.016204	22.946963
1645	1988/C/063	1	0	0	0	0	0	0	0	0	1	0	0	0	1	0	0	0	0	0	0	0	0	0	0	0	0	0	0	0	0	0	0	0	0	0	0	0	0	0	0	0	0	0	0	0	0	0	1	38.027857	22.853764
1646	1988/C/064	1	0	0	0	0	0	0	0	0	0	0	0	1	1	0	0	0	0	1	0	0	0	0	0	0	0	0	0	0	0	0	0	0	0	0	0	0	0	0	0	0	0	0	0	0	0	0	1	37.764578	23.124674
1647	1988/C/065	1	0	0	0	0	0	0	0	0	0	0	0	1	1	0	0	0	0	0	0	0	0	0	0	0	0	0	0	0	0	0	0	0	0	0	0	0	0	0	0	0	0	0	0	0	0	0	1	37.67959	23.134569
1648	1988/C/066	0	0	0	0	0	0	0	0	0	0	0	0	1	1	0	0	0	0	0	0	0	0	0	0	0	0	0	0	0	0	0	0	0	0	0	0	0	0	0	0	0	0	0	0	0	0	0	1	37.570801	23.149432
1649	1988/C/067	0	0	0	0	0	0	0	0	0	0	0	0	0	1	0	0	0	0	0	0	0	0	0	0	0	0	0	0	0	0	0	0	0	0	0	0	0	0	0	0	0	0	0	0	0	0	0	1	37.557065	23.374457
1650	1988/C/068	0	0	0	0	0	0	0	0	0	0	0	0	0	1	0	0	0	0	0	0	0	0	0	0	0	0	0	0	0	0	0	0	0	0	0	0	0	0	0	0	0	0	0	0	0	0	0	1	37.397067	23.241321
1651	1988/C/069	0	0	0	0	0	0	0	0	0	1	0	0	0	1	0	0	0	0	0	0	0	0	0	0	0	0	0	0	0	0	0	0	0	0	0	0	0	0	0	0	0	0	0	0	0	0	0	1	37.521176	22.859773

1652	1988/C/070	0	0	0	0	0	0	0	0	0	0	0	0	0	1	0	0	0	0	0	0	0	0	0	0	0	0	0	0	0	0	0	0	0	0	0	0	0	0	0	0	0	0	0	0	0	0	0	1	37.513674	22.736441
1653	1988/C/071	1	0	0	0	0	0	0	0	0	0	0	0	0	1	0	0	0	0	0	0	0	0	0	0	0	0	0	0	0	0	0	0	0	0	0	0	0	0	0	0	0	0	0	0	0	0	0	1	37.383882	22.744632
1654	1988/C/072	1	0	0	0	0	0	0	0	0	0	0	0	0	1	0	0	0	0	0	0	0	0	0	0	0	0	0	0	0	0	0	0	0	0	0	0	0	0	0	0	0	0	0	0	0	0	0	1	37.339378	22.792704
1655	1988/C/073	0	0	0	0	0	0	0	0	0	0	0	0	0	1	0	0	0	0	0	0	0	0	0	0	0	0	0	0	0	0	0	0	0	0	0	0	0	0	0	0	0	0	0	0	0	0	0	1	37.146349	22.892263
1656	1988/C/074	0	0	0	0	0	0	0	0	0	0	0	0	1	1	0	0	0	0	0	0	0	0	0	0	0	0	0	0	0	0	0	0	0	0	0	0	0	0	0	0	0	0	0	0	0	0	0	1	37.098999	22.870154
1657	1988/C/075	0	0	0	0	0	0	0	0	0	0	0	0	0	1	0	0	0	0	0	0	0	0	0	0	0	0	0	0	0	0	0	0	0	0	0	0	0	0	0	0	0	0	0	0	0	0	0	1	37.164461	22.839021
1658	1988/C/076	0	0	0	0	0	0	0	0	0	0	0	0	0	1	0	0	0	0	0	0	0	0	0	0	0	0	0	0	0	0	0	0	0	0	0	0	0	0	0	0	0	0	0	0	0	0	0	1	37.163004	22.810529
1660	1988/C/078	0	0	0	0	0	0	0	0	0	0	0	0	0	1	0	0	0	0	0	0	0	0	0	0	0	0	0	0	0	0	0	0	0	0	0	0	0	0	0	0	0	0	0	0	0	0	0	1	37.025115	22.554651
1661	1988/C/079	0	0	0	0	0	0	0	0	0	0	0	0	0	1	0	0	0	0	0	0	0	0	0	0	0	0	0	0	0	0	0	0	0	0	0	0	0	0	0	0	0	0	0	0	0	0	0	1	37.008464	22.649885
1662	1988/C/080	0	0	0	0	0	0	0	0	0	0	0	0	1	0	0	0	0	0	0	0	0	0	0	0	0	0	0	0	0	0	0	0	0	0	0	0	0	0	0	0	0	0	0	0	0	0	0	1	37.081998	22.282809
1664	1988/C/082	0	0	0	0	0	0	0	0	0	0	0	0	1	0	0	0	0	0	0	0	0	0	0	0	0	0	0	0	0	0	0	0	0	0	0	0	0	0	0	0	0	0	0	0	0	0	0	1	37.49026	22.349446
1666	1988/C/084	1	0	0	0	0	0	0	0	0	0	0	0	0	0	0	0	0	0	0	0	0	0	0	0	0	0	0	0	0	0	0	0	0	0	0	0	0	0	0	0	0	0	0	0	0	0	0	1	37.487291	21.917913
1667	1988/C/085	1	0	0	0	0	0	0	0	0	0	0	0	0	1	0	0	0	0	0	0	0	0	0	0	0	0	0	0	0	0	0	0	0	0	0	0	0	0	0	0	0	0	0	0	0	0	0	1	37.448929	21.910396
1671	1992/A/002	1	0	0	0	0	0	0	0	0	0	0	0	1	1	0	0	0	0	1	0	0	0	0	0	0	0	0	0	0	0	0	0	0	0	0	0	0	0	0	0	0	0	0	0	0	0	0	1	37.692119	23.139573
1674	1992/A/005	1	0	0	0	0	0	0	0	0	0	0	0	1	1	0	0	0	0	1	0	0	0	0	0	0	1	0	0	0	0	0	0	0	0	0	0	0	0	0	0	0	0	1	0	0	0	0	1	37.889167	22.869722
1675	1992/A/006	1	0	0	0	0	0	0	0	0	0	0	0	0	1	0	0	0	0	0	0	0	0	0	0	0	0	0	0	0	0	0	0	0	0	0	0	0	0	0	0	0	0	0	0	0	0	0	1	37.982925	22.97693
1677	1992/A/008	1	0	0	0	0	0	0	0	0	0	0	0	0	1	0	0	0	0	0	0	0	0	0	0	0	0	0	0	0	0	0	0	0	0	0	0	0	0	0	0	0	0	0	0	0	0	0	1	37.840649	22.899091
1679	1992/A/010	1	0	0	0	0	0	0	0	0	0	0	0	1	1	0	0	0	0	1	0	0	0	0	0	0	0	0	0	0	0	0	0	0	0	0	0	0	0	0	0	0	0	0	0	0	0	0	1	37.76	22.88
1680	1992/A/011	0	0	0	0	0	0	0	0	0	0	0	0	1	0	0	0	0	0	0	0	0	0	0	0	0	0	0	0	0	0	0	0	0	0	0	0	0	0	0	0	0	0	0	0	0	0	0	1	37.753545	22.877767
1681	1992/A/012	1	0	0	0	0	0	0	0	0	0	0	0	1	1	0	0	0	0	0	0	0	0	0	0	0	0	0	0	0	0	0	0	0	0	0	0	0	0	0	0	0	0	0	0	0	0	0	1	37.756399	22.819928
1682	1992/A/013	1	0	0	0	0	0	0	0	0	0	0	0	1	1	0	0	0	0	0	0	0	0	0	0	0	0	0	0	0	0	0	0	0	0	0	0	0	0	0	0	0	0	0	0	0	0	0	1	37.739199	22.878892
1684	1992/A/015	1	0	0	0	0	0	0	0	0	0	0	0	1	1	0	0	0	0	1	0	0	0	0	0	0	0	0	0	0	0	0	0	0	0	0	0	0	0	0	0	0	0	0	0	0	0	0	1	37.710454	22.83472
1685	1992/A/016	0	0	0	0	0	0	0	0	0	0	0	0	1	1	0	0	0	0	1	0	0	0	0	0	0	0	0	0	0	0	0	0	0	0	0	0	0	0	0	0	0	0	0	0	0	0	0	1	37.638091	22.715355
1686	1992/A/017	0	0	0	0	0	0	0	0	0	0	0	0	0	1	0	0	0	0	1	0	0	0	0	0	0	0	0	0	0	0	0	0	0	0	0	0	0	0	0	0	0	0	0	0	0	0	0	1	37.520101	22.729946
1688	1992/A/019	0	0	0	0	0	0	0	0	0	0	0	0	1	1	0	0	0	0	1	0	0	0	0	0	0	0	0	0	0	0	0	0	0	0	0	0	0	0	0	0	0	0	0	0	0	0	0	1	37.339458	22.763359
1689	1992/A/020	1	0	0	0	0	0	0	0	0	0	0	0	1	1	0	0	0	0	1	0	0	0	0	0	0	1	0	0	0	0	0	0	0	0	0	0	0	0	0	0	0	0	0	0	0	0	0	1	37.328701	22.754276
1690	1992/A/021	0	0	0	0	0	0	0	0	0	0	0	0	0	1	0	0	0	0	0	0	0	0	0	0	0	0	0	0	0	0	0	0	0	0	0	0	0	0	0	0	0	0	0	0	0	0	0	1	37.264714	22.846941
1693	1992/A/024	0	0	0	0	0	0	0	0	0	0	0	0	0	1	0	0	0	0	1	0	0	0	0	0	0	0	0	0	0	0	0	0	0	0	0	0	0	0	0	0	0	0	0	0	0	0	0	1	37.152598	22.793423
1694	1992/A/025	0	0	0	0	0	0	0	0	0	0	0	0	1	1	0	0	0	0	0	0	0	0	0	0	0	0	0	0	0	0	0	0	0	0	0	0	0	0	0	0	0	0	0	0	0	0	0	1	37.143151	22.773645
1703	1992/A/034	0	0	0	0	0	0	0	0	0	0	0	0	0	1	0	0	0	0	0	0	0	0	0	0	0	0	0	0	0	0	0	0	0	0	0	0	0	0	0	0	0	0	0	0	0	0	0	1	37.322857	22.14248
1704	1992/A/035	0	0	0	0	0	0	0	0	0	0	0	0	0	1	0	0	0	0	0	0	0	0	0	0	0	0	0	0	0	0	0	0	0	0	0	0	0	0	0	0	0	0	0	0	0	0	0	1	37.319287	22.156376
1705	1992/A/036	1	0	0	0	0	0	0	0	0	0	0	0	0	1	0	0	0	0	1	0	0	0	0	0	0	0	0	0	0	0	0	0	0	0	0	0	0	0	0	0	0	0	0	0	0	0	0	1	37.26833	22.21764
1706	1992/A/037	0	0	0	0	0	0	0	0	0	0	0	0	1	0	0	0	0	0	0	0	0	0	0	0	0	0	0	0	0	0	0	0	0	0	0	0	0	0	0	0	0	0	0	0	0	0	0	1	37.2443	22.247248
1709	1992/A/040	0	0	0	0	0	0	0	0	0	0	0	0	0	1	0	0	0	0	0	0	0	0	0	0	0	0	0	0	0	0	0	0	0	0	0	0	0	0	0	0	0	0	0	0	0	0	0	1	37.026496	22.539432
1712	1992/A/043	0	0	0	0	0	0	0	0	0	0	0	0	0	1	0	0	0	0	0	0	0	0	0	0	0	0	0	0	0	0	0	0	0	0	0	0	0	0	0	0	0	0	0	0	0	0	0	1	36.939206	22.773784
1714	1992/A/045	1	0	0	0	0	0	0	0	0	0	0	0	0	1	0	0	0	0	0	0	0	0	0	0	0	0	0	0	0	0	0	0	0	0	0	0	0	0	0	0	0	0	0	0	0	0	0	1	36.688618	23.054773
1715	1992/A/046	1	0	0	0	0	0	0	0	0	0	0	0	0	1	0	0	0	0	1	0	0	0	0	0	0	0	0	0	0	0	0	0	0	0	0	0	0	0	0	0	0	0	0	0	0	0	0	1	36.688185	23.04962
1716	1992/A/047	0	0	0	0	0	0	0	0	0	0	0	0	0	1	0	0	0	0	0	0	0	0	0	0	0	0	0	0	0	0	0	0	0	0	0	0	0	0	0	0	0	0	0	0	0	0	0	1	36.773364	22.836695
1717	1992/A/048	1	0	0	0	0	0	0	0	0	0	0	0	0	1	0	0	0	0	0	0	0	0	0	0	0	0	0	0	0	0	0	0	0	0	0	0	0	0	0	0	0	0	0	0	0	0	0	1	36.801821	22.492526
1718	1992/A/049	0	0	0	0	0	0	0	0	0	0	0	0	0	0	0	0	0	0	0	0	0	0	0	0	0	0	0	0	0	0	0	0	0	0	0	0	0	0	0	0	0	0	0	0	0	0	0	1	36.815865	22.461376
1719	1992/A/050	0	0	0	0	0	0	0	0	0	0	0	0	0	1	0	0	0	0	0	0	0	0	0	0	0	0	0	0	0	0	0	0	0	0	0	0	0	0	0	0	0	0	0	0	0	0	0	1	36.978042	22.428122
1720	1992/A/051	0	0	0	0	0	0	0	0	0	0	0	0	1	0	0	0	0	0	0	0	0	0	0	0	0	0	0	0	0	0	0	0	0	0	0	0	0	0	0	0	0	0	0	0	0	0	0	1	36.966356	22.388656
1721	1992/A/052	0	0	0	0	0	0	0	0	0	0	0	0	0	1	0	0	0	0	0	0	0	0	0	0	0	0	0	0	0	0	0	0	0	0	0	0	0	0	0	0	0	0	0	0	0	0	0	1	36.669997	22.381176
1722	1992/A/053	0	0	0	0	0	0	0	0	0	0	0	0	0	1	0	0	0	0	0	0	0	0	0	0	0	0	0	0	0	0	0	0	0	0	0	0	0	0	0	0	0	0	0	0	0	0	0	1	36.638337	22.380651
1723	1992/A/054	1	0	0	0	0	0	0	0	0	0	0	0	1	0	0	0	0	0	0	0	0	0	0	0	0	0	0	0	0	0	0	0	0	0	0	0	0	0	0	0	0	0	0	0	0	0	0	1	36.770166	22.346468
1724	1992/A/055	0	0	0	0	0	0	0	0	0	0	0	0	1	1	0	0	0	0	0	0	0	0	0	0	0	0	0	0	0	0	0	0	0	0	0	0	0	0	0	0	0	0	0	0	0	0	0	1	36.869246	22.244546
1726	1992/A/057	1	0	0	0	0	0	0	0	0	0	0	0	0	1	0	0	0	0	0	0	0	0	0	0	0	0	0	0	0	0	0	0	0	0	0	0	0	0	0	0	0	0	0	0	0	0	0	1	37.648027	21.73378
1727	1992/A/058	0	0	0	0	0	0	0	0	0	0	0	0	0	1	0	0	0	0	0	0	0	0	0	0	0	0	0	0	0	0	0	0	0	0	0	0	0	0	0	0	0	0	0	0	0	0	0	1	37.701074	21.909719
1728	1992/A/059	0	0	0	0	0	0	0	0	0	0	0	0	0	0	0	0	0	0	0	0	0	0	0	0	0	0	0	0	0	0	0	0	0	0	0	0	0	0	0	0	0	0	0	0	0	0	0	1	37.693193	21.980218
1730	1992/A/061	0	0	0	0	0	0	0	0	0	0	0	0	1	1	0	0	0	0	0	0	0	0	0	0	0	0	0	0	0	0	0	0	0	0	0	0	0	0	0	0	0	0	0	0	0	0	0	1	37.674799	22.129208
1731	1992/A/062	0	0	0	0	0	0	0	0	0	0	0	0	1	0	0	0	0	0	0	0	0	0	0	0	0	0	0	0	0	0	0	0	0	0	0	0	0	0	0	0	0	0	0	0	0	0	0	1	37.824065	22.142862
1734	1992/A/065	1	0	0	0	0	0	0	0	0	0	0	0	0	1	0	0	0	0	0	0	0	0	0	0	0	0	0	0	0	0	0	0	0	0	0	0	0	0	0	0	0	0	0	0	0	0	0	1	38.054329	22.138124
1735	1992/A/066	1	0	0	0	0	0	0	0	0	0	0	0	1	1	0	0	0	0	1	0	0	0	0	0	0	0	0	0	0	0	0	0	0	0	0	0	0	0	0	0	0	0	0	0	0	0	0	1	38.368601	22.127928
1736	1992/A/067	0	0	0	0	0	0	0	0	0	0	0	0	1	0	0	0	0	0	0	0	0	0	0	0	0	0	0	0	0	0	0	0	0	0	0	0	0	0	0	0	0	0	0	0	0	0	0	1	38.34786	22.313012
1738	1992/A/069	1	0	0	0	0	0	0	0	0	0	0	0	1	1	0	0	0	0	1	0	0	0	0	0	0	0	0	0	0	0	0	0	0	0	0	0	0	0	0	0	0	0	0	0	0	0	0	1	38.452649	22.70147
1739	1992/A/070	1	0	0	0	0	0	0	0	0	0	0	0	0	1	0	0	0	0	0	0	0	0	0	0	0	0	0	0	0	0	0	0	0	0	0	0	0	0	0	0	0	0	0	0	0	0	0	1	38.171966	23.375409
1740	1994/01	1	0	0	0	0	0	0	0	0	0	0	0	1	1	0	0	0	0	1	0	0	0	0	0	0	0	0	0	0	0	0	0	0	0	0	0	0	0	0	0	0	0	0	0	0	0	0	1	45.781	14.205
1741	1994/02	1	0	0	0	0	0	0	0	0	0	0	0	0	0	0	0	0	0	0	0	0	0	0	0	0	1	0	1	0	0	0	0	0	0	0	0	0	0	0	0	0	0	0	0	0	0	0	1	45.64	14.17
1742	1994/03	1	0	0	0	0	0	0	0	0	0	0	0	1	0	0	0	0	0	1	0	0	0	0	0	0	1	0	1	0	1	0	0	0	0	0	0	0	0	0	0	0	0	0	0	0	0	0	1	45.51	14.29
1744	1994/05	1	0	0	0	0	0	0	0	0	0	0	0	0	0	0	0	0	0	1	0	0	0	0	0	0	0	0	0	0	0	0	0	0	0	0	0	0	0	0	0	0	0	0	0	0	0	0	1	45.13	14.83
1745	1994/06	0	0	0	0	0	0	0	0	0	0	0	0	1	0	0	1	0	0	1	1	0	0	0	0	0	0	0	1	0	1	0	0	0	0	0	0	0	0	0	0	0	0	0	0	0	0	0	1	44.98	14.99
1746	1994/07	1	0	0	0	0	0	0	0	0	0	0	0	1	0	0	0	0	0	1	0	0	0	0	0	0	0	0	1	0	0	0	0	0	0	0	0	0	0	0	0	0	1	0	0	0	0	0	1	44.99	14.98
1747	1994/08	1	0	0	0	0	0	0	0	0	1	0	0	1	0	0	0	0	0	1	1	0	0	0	0	0	0	0	1	0	0	0	0	0	0	0	0	0	0	0	0	0	0	0	1	0	0	0	1	44.78	14.76
1748	1994/09	1	0	0	0	0	1	0	0	0	0	0	0	1	0	0	0	0	0	1	0	0	0	0	0	0	0	0	1	0	0	0	0	0	0	0	0	0	0	0	0	0	1	0	1	0	0	0	1	44.72	14.92
1749	1994/10	1	0	0	0	0	0	0	0	0	0	0	0	0	0	0	0	0	0	1	0	0	0	0	0	0	0	0	1	0	0	0	0	0	0	0	0	0	0	0	0	0	1	0	0	0	0	0	1	45.12	14.83
1750	1994/11	1	0	0	0	0	0	0	0	0	0	0	0	0	0	0	0	0	0	0	0	0	0	0	0	0	0	0	0	0	0	0	0	0	0	0	1	0	0	0	0	0	0	0	0	0	0	0	1	45.31	14.72
1751	1994/12	1	0	0	0	0	0	0	0	0	0	0	0	1	0	0	0	0	0	1	0	0	0	1	0	0	0	0	1	0	0	0	0	0	0	0	0	0	0	0	0	0	0	0	0	0	0	0	3	45.36	14.77
1752	1994/13	1	0	0	0	0	0	0	0	0	1	0	0	0	0	0	0	0	0	1	0	0	0	0	0	0	0	0	1	0	0	0	0	0	0	0	0	0	0	0	0	0	0	0	0	0	0	0	1	45.35	13.76
1753	1994/14	1	0	0	0	0	1	0	0	0	0	0	0	1	0	0	0	0	0	1	0	0	0	0	0	0	0	0	1	0	0	0	0	0	0	0	0	0	0	0	0	0	0	0	0	0	0	0	1	45.36	13.75
1754	1994/15	1	0	0	0	0	0	0	0	0	0	0	0	0	0	0	0	0	0	1	0	0	0	1	0	0	0	0	0	0	1	0	0	0	0	0	0	0	0	0	0	0	0	0	0	0	0	0	1	45.13	13.76
1760	1995/40	0	0	0	0	0	0	0	0	0	0	0	0	0	0	0	0	0	0	1	0	0	0	0	0	0	0	0	0	0	0	0	0	0	0	0	0	0	0	0	0	0	0	0	0	0	0	0	1	44.91	22.37
1761	1995/41	0	0	0	0	0	0	0	0	0	0	0	0	0	0	0	0	0	0	0	0	0	0	0	0	0	0	0	0	0	1	0	0	0	0	0	0	0	0	0	0	0	0	0	0	0	0	0	1	45.2	22.32
1763	1995/43	0	0	0	0	0	1	0	0	0	0	0	0	1	1	0	0	0	0	0	0	0	0	0	0	0	0	0	0	0	1	0	0	0	1	0	0	0	0	0	0	0	0	0	0	0	0	0	1	41.328	23.354
1764	1995/44	0	0	0	0	0	0	0	0	0	0	0	0	0	1	0	0	0	0	0	0	0	0	0	0	0	0	0	0	0	1	0	0	0	0	0	0	0	0	0	0	0	0	0	0	0	0	0	1	40.719	23.65
1766	1995/46	0	0	0	0	0	1	0	0	0	0	0	0	0	0	0	0	0	0	1	0	0	0	1	0	0	0	0	0	0	1	0	0	0	0	0	0	0	0	0	0	1	0	0	0	0	0	0	1	40.886	24.072
1767	1995/47	0	0	0	0	0	1	0	0	0	0	0	0	0	1	0	0	0	0	0	0	0	0	1	0	0	0	0	0	0	0	0	0	0	0	0	0	0	0	0	0	1	0	0	0	0	0	0	1	40.899	24.045
1768	1995/48	0	0	0	0	0	1	0	0	0	0	0	0	1	0	0	0	0	0	1	0	0	0	0	0	0	0	0	0	0	1	0	0	0	0	0	0	0	0	0	0	1	0	0	0	0	0	0	3	40.913	24.072
1770	1995/50	0	0	0	0	0	1	0	0	0	0	0	0	0	1	0	0	0	0	1	0	0	0	1	0	0	0	0	0	0	0	0	0	0	1	0	0	0	0	0	0	0	0	0	0	0	0	0	1	40.799	23.928
1771	1995/51	1	0	0	0	0	1	0	0	0	0	0	0	0	1	0	0	0	0	0	0	0	0	0	0	0	0	0	0	0	0	0	0	0	0	0	0	0	0	0	0	0	0	0	0	0	0	0	1	40.725	23.695
1772	1995/52	1	0	0	0	0	1	0	0	0	1	0	0	0	1	0	0	0	0	0	0	0	0	0	0	0	0	0	0	0	1	0	0	0	1	0	0	0	0	0	0	1	0	0	0	0	0	0	1	40.752	24.09
1773	1995/53	1	0	0	0	0	1	0	0	0	0	0	0	0	0	0	0	0	0	0	0	0	0	0	0	0	0	0	0	0	0	0	0	0	1	0	0	0	0	0	0	0	0	0	0	0	0	0	1	40.745	24.018
1774	1995/54	1	0	0	0	0	1	0	0	0	0	0	0	1	1	0	0	0	0	1	0	0	0	0	0	0	0	0	0	0	1	0	0	0	1	0	0	0	0	0	0	0	0	0	0	0	0	0	2	41.017	24.612
1775	1995/55	1	0	0	0	0	1	0	0	0	0	0	0	1	1	0	0	0	0	0	0	0	0	0	0	0	0	0	0	0	1	0	0	0	1	0	0	0	0	0	0	0	0	0	0	0	0	0	1	41.02	24.289
1776	1995/56	0	0	0	0	0	0	0	0	0	0	0	0	1	0	0	0	0	0	0	0	0	0	0	0	0	0	0	0	0	1	0	0	0	0	0	0	0	0	0	0	1	0	0	0	0	0	0	1	40.739	24.686
1777	1995/57	1	0	0	0	0	1	0	0	0	0	0	0	0	0	0	0	0	0	0	0	0	0	0	0	0	0	0	0	0	1	0	0	0	0	0	0	0	0	0	0	1	0	0	0	0	0	0	1	40.739	24.722
1778	1995/58	0	0	0	0	0	1	0	0	0	0	0	0	0	1	0	0	0	0	1	1	0	0	0	0	0	0	0	0	0	1	0	0	0	1	0	0	0	0	0	0	0	0	0	0	0	0	0	1	41.208	23.91
1779	1995/59	0	0	0	0	0	1	0	0	0	0	0	0	1	0	0	0	0	0	0	0	0	0	0	0	0	0	0	0	0	0	0	0	0	0	0	0	0	0	0	0	0	0	0	0	0	0	0	1	41.288	23.973
1780	1995/60	1	0	0	0	0	1	0	0	0	0	0	0	0	1	0	0	0	0	0	0	0	0	0	0	0	0	0	0	0	1	0	0	0	1	0	0	0	0	0	0	0	0	0	0	0	0	0	1	41.06	23.91
1781	1995/61	1	0	0	0	0	1	0	0	0	0	0	0	0	1	0	0	0	0	0	0	0	0	0	0	0	0	0	0	0	0	0	0	0	1	0	0	0	0	0	0	0	0	0	0	0	0	0	1	41.208	23.973
1783	1995/63	0	0	0	0	0	1	0	0	0	0	0	0	1	1	1	0	0	0	0	0	0	0	0	0	0	0	0	0	0	1	0	0	0	0	0	1	0	0	0	0	1	0	0	0	0	0	0	1	43.04	23.412
1785	1996/02	0	0	0	0	0	0	0	0	0	0	0	0	0	0	0	0	0	0	1	0	0	0	0	0	0	0	0	0	0	0	0	0	0	0	0	0	0	0	1	0	0	0	0	0	0	0	0	1	44.14	19.93
1788	1997/18	0	0	0	0	0	0	0	0	0	0	0	0	0	0	0	0	0	0	0	0	0	0	0	0	0	0	0	0	0	0	0	0	0	0	0	0	0	0	0	0	0	0	0	1	0	0	0	1	44.29	15.45
1791	1997/21	1	0	0	0	0	0	0	0	0	0	0	0	1	0	0	0	0	0	1	0	0	0	0	0	0	0	0	1	0	0	0	0	0	0	0	0	0	0	0	0	0	1	0	0	0	0	0	1	43.91	15.98
1792	1997/22	0	0	0	0	0	0	0	0	0	1	0	0	0	0	0	0	0	0	1	1	0	0	0	0	0	0	0	0	0	0	0	0	0	0	0	0	0	0	0	0	0	0	0	0	0	0	0	1	43.8	15.97
1794	1997/24	0	0	0	0	0	0	0	0	0	0	0	0	1	0	0	0	0	0	0	0	0	0	0	0	0	0	0	0	0	0	0	0	0	0	0	0	0	0	0	0	0	1	0	0	0	0	0	1	42.94	17.1
1795	1997/25	0	0	0	0	0	0	0	0	0	0	0	0	1	0	0	0	0	0	0	0	0	0	0	0	0	0	0	0	0	0	0	0	0	0	0	0	0	0	0	0	0	1	0	0	0	0	0	1	42.93	17.01
1796	1997/26	1	0	0	0	0	0	0	0	0	0	0	0	1	0	0	0	0	0	1	0	0	0	0	0	0	0	0	0	0	0	1	0	0	0	0	0	0	0	0	0	0	0	0	0	0	0	0	1	42.99	17.17
1797	1997/27	1	0	0	0	0	0	0	0	0	0	0	0	1	0	0	0	0	0	1	0	0	0	0	0	0	0	0	0	0	0	0	0	0	0	0	0	0	0	0	0	0	0	0	0	0	0	0	1	42.99	17.06
1798	1997/28	0	0	0	1	0	0	0	0	0	0	0	0	0	0	0	0	0	0	1	0	0	0	0	0	0	0	0	0	0	0	1	0	0	0	0	0	0	0	0	0	0	0	0	0	0	0	0	1	42.96	17.29
1799	1997/29	0	0	0	0	0	0	0	0	0	0	0	0	0	0	0	0	0	0	0	0	0	0	0	0	0	0	0	0	1	0	1	0	0	0	0	0	0	0	0	0	0	0	0	0	0	0	0	1	42.924167	17.407
1800	1997/30	0	0	0	0	0	0	0	0	0	0	0	0	0	0	0	0	0	0	0	0	0	0	0	0	0	0	0	0	0	0	0	0	0	0	0	0	0	0	0	0	0	0	0	0	0	0	0	1	42.84	17.63
1801	1997/31	0	0	0	0	0	0	0	0	0	0	0	0	1	0	1	0	0	0	0	1	0	0	0	0	0	0	0	1	0	0	1	0	0	0	0	0	1	0	0	0	0	0	1	0	0	0	0	1	42.8456	17.704
1803	1997/33	1	0	0	1	0	0	0	0	0	0	0	0	1	0	0	0	0	0	1	0	0	0	0	0	0	0	0	0	0	0	0	0	0	0	0	0	0	0	0	0	0	1	0	0	0	0	0	1	43.16	17.24
1804	1997/34	1	0	0	0	0	0	0	0	0	0	0	0	1	0	0	0	0	0	1	0	0	0	0	0	0	0	0	0	0	0	0	0	0	0	0	0	0	0	0	0	0	1	0	0	0	0	0	1	43.19	17.17
1805	1997/35	0	0	0	0	0	0	0	0	0	0	0	0	1	0	0	0	0	0	0	0	0	0	0	0	0	0	0	0	0	0	0	0	0	0	0	0	0	0	0	0	0	1	0	0	0	0	0	1	43.22	17.12
1806	1997/36	1	0	0	1	0	0	0	0	0	0	0	0	0	0	0	0	0	0	1	0	0	0	0	0	0	0	0	1	0	0	0	0	0	0	0	0	0	0	0	0	0	1	0	0	0	0	0	1	43.32	17
1808	1997/38	1	0	0	0	0	0	0	0	0	1	0	0	1	0	0	0	0	0	1	0	0	0	0	0	0	0	0	0	0	1	0	0	0	0	0	1	0	0	0	0	0	0	0	0	0	0	0	1	44.87	15.6
1809	1997/40	1	0	0	0	0	0	0	0	0	0	0	0	0	0	0	0	0	1	0	0	0	0	0	1	0	0	0	0	0	1	0	0	0	0	0	1	0	0	1	0	1	0	0	0	0	0	0	3	44.88	21.73
1810	1997/41	0	0	0	0	0	0	0	0	0	0	0	0	0	0	0	0	0	0	1	0	0	0	0	0	0	0	0	0	0	1	0	0	0	0	0	0	0	0	1	0	0	0	0	0	0	0	0	3	45.03	21.86
1811	1997/42	0	0	0	0	0	0	0	0	0	0	0	0	0	0	0	0	0	0	1	0	0	0	1	0	0	0	0	0	0	0	0	0	0	0	0	0	0	0	0	0	0	0	0	0	0	0	0	1	44.88	22.42
1812	1997/43	1	0	0	0	0	0	0	0	0	0	0	0	1	0	0	0	0	0	1	0	0	0	1	1	0	0	0	0	0	1	0	0	0	0	0	0	0	0	0	0	1	0	0	0	0	0	0	1	44.88	22.45
1813	1997/44	1	0	0	0	0	0	0	0	0	0	0	0	0	0	0	0	0	0	1	0	0	0	0	1	0	0	0	0	0	0	0	0	0	0	0	1	0	0	0	0	0	0	0	0	0	0	0	1	44.96	22.48
1814	1997/45	1	0	0	0	0	1	0	0	0	0	0	0	0	0	0	0	0	0	0	0	0	0	0	0	0	0	0	0	0	0	0	0	0	0	0	0	0	0	1	0	0	0	0	0	0	0	0	1	44.71	22.5
1815	1997/46	1	0	0	0	0	1	0	0	0	0	0	0	0	0	0	0	0	0	1	0	0	0	0	0	0	0	0	0	0	0	0	0	0	0	0	0	0	0	0	0	1	0	0	0	0	0	0	1	43.78	22.31
1816	1997/47	0	0	0	0	0	1	0	0	0	0	0	0	0	1	0	0	0	0	1	0	0	0	0	0	0	0	0	0	0	0	0	0	0	1	0	0	0	0	0	0	1	0	0	0	0	0	0	1	43.5395	22.2888
1817	1997/48	1	0	0	0	0	1	0	0	0	0	0	0	1	1	0	0	0	0	1	0	0	0	0	0	0	0	0	0	0	1	0	0	0	0	0	0	0	0	0	0	1	0	0	0	0	0	0	3	41.954	24.844
1818	1997/49	0	0	0	0	0	1	0	0	0	0	0	0	0	0	0	0	0	0	0	0	0	0	0	0	0	0	0	0	0	1	0	0	0	0	0	0	0	0	0	0	0	0	0	0	0	0	0	2	41.888	24.661
1819	1997/50	0	0	0	0	0	0	0	0	0	1	0	0	0	1	0	1	0	0	1	0	0	0	0	0	0	0	0	0	0	1	0	0	0	0	0	0	0	0	0	0	1	0	0	0	0	0	0	3	41.795	24.607
1820	1997/51	0	0	0	0	0	0	0	0	0	0	0	0	1	0	0	1	0	0	0	0	0	0	1	0	0	0	0	0	0	1	0	0	0	0	0	0	0	0	0	0	1	0	0	0	0	0	0	1	41.802	24.57
1821	1997/52	0	0	0	0	0	1	0	0	0	0	0	0	1	1	0	0	0	0	0	0	0	0	0	0	0	0	0	0	0	1	0	0	0	0	0	0	0	0	0	0	0	0	0	0	0	0	0	1	42.007	24.89
1822	1997/53	0	0	0	0	0	0	0	0	0	0	0	0	0	0	0	0	0	0	0	0	0	0	0	0	0	0	0	0	0	1	0	0	0	0	0	0	0	0	0	0	0	0	0	0	0	0	0	1	42.677	26.325
1828	1997/59	0	0	0	0	0	1	0	0	0	1	0	0	0	0	0	0	0	0	0	1	0	0	0	0	0	1	0	0	0	1	0	0	0	0	0	0	0	0	0	0	0	0	0	0	0	0	0	1	42.03	27.624
1832	1997/63	0	0	0	0	0	1	0	0	0	0	0	0	0	0	0	0	0	0	0	0	0	0	0	0	0	0	0	0	0	1	0	0	0	0	0	0	0	0	0	0	0	0	0	0	0	0	0	1	42.026	27.477
1833	1997/64	0	0	0	0	0	0	0	0	0	0	0	0	0	0	0	0	0	1	1	0	0	0	0	1	0	0	0	0	0	1	0	0	1	0	0	0	0	0	0	0	0	0	0	0	0	0	0	1	45.18	23.82
1834	1997/65	0	0	0	0	0	0	0	0	0	0	0	0	1	0	0	0	0	0	1	0	0	0	0	1	0	0	0	0	0	0	0	0	1	0	0	0	0	0	0	0	0	0	0	0	0	0	0	1	45.14	23.14
1835	1997/70	1	0	0	0	0	0	0	0	0	0	0	0	0	0	0	0	0	0	1	0	0	0	0	0	0	0	0	0	0	0	0	0	0	0	0	1	0	0	0	0	0	1	0	0	0	0	0	1	45.24	13.93
1836	1997/71	1	0	0	0	0	0	0	0	0	0	0	0	0	0	0	0	0	0	1	0	0	0	1	0	0	0	0	1	0	0	0	0	0	0	0	0	0	0	0	0	0	1	0	0	0	0	0	1	45.14	13.73
1839	1997/74	0	0	0	0	0	0	0	0	0	0	0	0	0	0	0	0	0	0	0	0	0	0	0	0	0	0	0	0	0	0	0	0	0	0	0	0	0	0	0	0	0	0	0	0	0	0	0	1	45.09	14.13
1840	1997/75	0	0	0	0	0	0	0	0	0	0	0	0	0	0	0	0	0	0	0	0	0	0	0	0	0	0	0	0	0	0	0	0	0	0	0	0	0	0	0	0	0	0	0	0	0	0	0	1	45.14	14.19
1842	1997/77	1	0	0	0	0	0	0	0	0	0	0	0	1	0	0	0	0	0	1	0	0	0	1	0	0	0	0	0	0	1	0	0	0	0	0	0	0	0	0	0	0	0	0	0	0	0	0	1	45.4	13.97
1843	1997/78	1	0	0	0	0	0	0	0	0	0	0	0	0	0	0	1	0	0	1	0	0	0	0	0	0	0	0	0	0	0	0	0	0	0	0	0	0	0	0	0	0	0	0	0	0	0	0	1	45.65	14.02
1845	1998/03	0	0	0	0	0	0	0	0	0	0	0	0	0	0	0	0	0	0	0	0	0	0	0	0	0	0	0	0	0	1	0	0	0	0	0	0	0	0	0	0	0	0	0	0	0	0	0	1	45.55	24.26
1847	1998/05	0	0	0	0	0	0	0	0	0	0	0	0	0	0	0	0	0	0	0	0	0	0	0	0	0	0	0	0	0	1	0	0	0	0	0	0	0	0	0	0	0	0	0	0	0	0	0	1	45.4	25.15
1848	1998/06	0	0	0	0	0	0	0	0	0	0	0	0	0	0	0	0	0	0	0	0	0	0	1	0	0	0	0	0	0	1	0	0	1	0	0	0	0	0	0	0	0	0	0	0	0	0	0	1	45.42	25.21
1849	1998/07	0	0	0	0	0	0	0	0	0	0	0	0	0	0	0	0	0	0	1	0	0	0	0	0	0	0	0	0	0	1	0	0	1	0	0	0	0	0	0	0	0	0	0	0	0	0	0	1	45.44	25.23
1850	1998/08	0	0	0	0	0	0	0	0	0	0	0	0	0	0	0	0	0	0	0	0	0	0	0	0	0	0	0	0	0	0	0	0	1	0	0	0	0	0	0	0	0	0	0	0	0	0	0	1	45.51	25.36
1851	1998/09	0	0	0	0	0	0	0	0	0	0	0	0	0	0	0	0	0	0	1	0	0	0	0	0	0	0	0	0	0	1	0	0	1	0	0	0	0	0	0	0	1	0	0	0	0	0	0	1	45.57	25.26
1852	1998/10	0	0	0	0	0	0	0	0	0	0	0	0	0	0	0	0	0	0	0	0	0	0	0	0	0	0	0	0	0	1	0	0	1	0	0	0	0	0	0	0	0	0	0	0	0	0	0	1	45.56	25.28
1853	1998/11	0	0	0	0	0	0	0	0	0	1	0	0	0	0	0	0	0	0	1	0	0	0	1	0	0	0	0	0	0	1	0	0	1	0	0	0	0	0	0	0	0	0	0	0	0	0	0	1	45.51	25.5
1854	1998/12	0	0	0	0	0	0	0	0	0	0	0	0	0	0	0	0	0	0	0	0	0	0	0	0	0	0	0	0	0	1	0	0	0	0	0	0	0	0	0	0	0	0	0	0	0	0	0	1	45.56	25.6

1855	1998/13	0	0	0	0	0	0	0	0	0	1	0	0	0	0	0	0	0	0	1	0	0	0	1	0	0	0	0	0	0	1	0	0	1	0	0	0	0	0	0	0	0	0	0	0	0	0	0	1	45.51	25.89
1856	1998/14	0	0	0	0	0	0	0	0	0	0	0	0	0	0	0	0	0	0	0	0	0	0	0	0	0	0	0	0	0	1	0	0	0	0	0	0	0	0	0	0	0	0	0	0	0	0	0	1	45.51	25.9
1857	1998/15	0	0	0	0	0	1	0	0	0	0	0	0	0	0	0	0	0	0	1	0	0	0	0	0	0	0	0	0	0	1	0	0	1	0	0	1	0	0	0	0	0	0	0	0	0	0	0	1	45.49	25.93
1858	1998/16	0	0	0	0	0	0	0	0	0	0	0	0	0	0	0	0	0	0	0	0	0	0	0	0	0	0	0	0	0	0	0	0	1	0	0	0	0	0	0	0	0	0	0	0	0	0	0	1	45.49	25.94
1859	1998/17	0	0	0	0	0	0	0	0	0	0	0	0	0	0	0	0	0	0	0	0	0	0	0	0	0	0	0	0	0	0	0	0	1	0	0	0	0	0	0	0	0	0	0	0	0	0	0	1	45.51	25.93
1860	1998/18	0	0	0	0	0	0	0	0	0	0	0	0	0	0	0	0	0	0	0	0	0	0	0	0	0	0	0	0	0	0	0	0	1	0	0	0	0	0	0	0	0	0	0	0	0	0	0	1	45.4926	25.9767
1861	1998/19	0	0	0	0	0	0	0	0	0	0	0	0	0	0	0	0	0	0	0	0	0	0	0	0	0	0	0	0	0	0	0	0	1	0	0	0	0	0	0	0	0	0	0	0	0	0	0	1	45.48	25.97
1862	1998/20	0	0	0	0	0	0	0	0	0	0	0	0	0	0	0	0	0	0	0	0	0	0	0	0	0	0	0	0	0	0	0	0	0	0	0	0	0	0	0	0	0	0	0	0	0	0	0	1	45.4888	25.9732
1863	1998/21	0	0	0	0	0	0	0	0	0	0	0	0	0	0	0	0	0	0	0	0	0	0	0	0	0	0	0	0	0	0	0	0	0	0	0	0	0	0	0	0	0	0	0	0	0	0	0	1	45.4945	25.9717
1864	1998/22	0	0	0	0	0	0	0	0	0	0	0	0	0	0	0	0	0	0	0	0	0	0	0	0	0	0	0	0	0	0	0	0	1	0	0	0	0	0	0	0	0	0	0	0	0	0	0	1	45.5	25.95
1865	1998/23	0	0	0	0	0	0	0	0	0	0	0	0	0	0	0	0	0	0	0	0	0	0	0	0	0	0	0	0	0	1	0	0	0	0	0	0	0	0	0	0	0	0	0	0	0	0	0	1	45.482	25.93
1866	1998/24	0	0	0	0	0	0	0	0	0	0	0	0	0	0	0	0	0	0	0	0	0	0	0	0	0	0	0	0	0	0	0	0	1	0	0	0	0	0	0	0	0	0	0	0	0	0	0	1	45.5	25.9
1867	1998/25	0	0	0	0	0	0	0	0	0	0	0	0	0	0	0	0	0	0	0	0	0	0	0	0	0	0	0	0	0	1	0	0	1	0	0	0	0	0	0	0	0	0	0	0	0	0	0	1	45.488	25.892
1868	1998/26	0	0	0	0	0	0	0	0	0	0	0	0	0	0	0	0	0	0	0	0	0	0	0	0	0	0	0	0	0	1	0	0	0	0	0	0	0	0	0	0	0	0	0	0	0	0	0	1	45.488	25.877
1869	1998/27	1	0	0	0	0	0	0	0	0	0	0	0	0	0	0	0	0	0	0	0	0	0	0	0	0	0	0	0	0	0	0	0	0	0	0	0	0	0	0	0	0	0	0	0	0	0	0	1	44.38	16.09
1871	1998/29	1	0	0	0	0	0	0	0	0	0	0	0	1	0	0	0	0	0	1	1	0	0	1	0	0	1	0	1	0	0	0	0	0	0	0	0	0	0	0	0	0	0	0	0	0	0	0	1	43.85	16.16
1872	1998/30	1	0	0	1	0	0	0	0	0	0	0	0	1	0	0	0	0	0	0	0	0	0	0	0	0	0	0	1	0	0	0	0	0	0	0	0	0	0	0	0	0	0	0	0	0	0	0	1	43.74	15.9
1873	1998/31	1	0	0	0	0	0	0	0	0	0	0	0	0	0	1	0	0	0	1	0	0	0	0	0	0	0	0	1	0	0	0	0	0	0	0	0	0	0	0	0	0	0	0	0	0	0	0	1	43.72	16.22
1874	1998/32	1	0	0	0	0	0	0	0	0	0	0	0	0	0	0	0	0	0	0	0	0	0	0	0	0	0	0	0	0	0	0	0	0	0	0	0	0	0	0	0	0	0	0	0	0	0	0	1	43.59	16.5
1875	1998/33	1	0	0	0	0	0	0	0	0	0	0	0	0	0	0	0	0	0	1	0	0	0	0	0	0	0	0	0	0	0	0	0	0	0	0	0	0	0	0	0	0	0	0	0	0	0	0	1	43.69	16.54
1876	1998/34	1	0	0	0	0	0	0	0	0	0	0	0	1	0	0	0	0	0	0	0	0	0	0	0	0	0	0	1	0	0	0	0	0	0	0	0	0	0	0	0	0	0	0	0	0	0	0	1	43.71	16.57
1877	1998/35	1	0	0	0	0	0	0	0	0	0	0	0	1	0	0	0	0	0	1	0	0	0	0	0	0	0	0	0	0	0	0	0	0	0	0	0	0	0	0	0	0	1	0	0	0	0	0	1	43.9138	16.396
1878	1998/36	0	0	0	0	0	0	0	0	0	0	0	0	0	0	0	0	0	0	1	0	0	0	0	0	0	0	0	0	0	0	0	0	0	0	0	0	0	0	0	0	0	1	0	0	0	0	0	1	43.4	16.88
1880	1998/38	0	0	0	0	0	0	0	0	0	0	0	0	1	0	0	0	0	0	0	0	0	0	0	0	0	0	0	0	0	0	1	0	0	0	0	0	0	0	0	0	0	0	0	0	0	0	0	1	43.11	17.35
1881	1998/39	1	0	0	0	0	0	0	0	0	0	0	0	0	0	0	0	0	0	1	0	0	0	0	0	0	0	0	1	0	0	1	0	0	0	0	0	0	0	0	0	0	0	0	1	0	0	0	1	42.57	18.29
1882	1998/40	1	0	0	0	0	0	0	0	0	0	0	0	0	0	0	0	0	0	1	0	0	0	0	0	0	0	0	0	0	0	1	0	0	0	0	0	0	0	0	0	0	0	0	0	0	0	0	1	42.56	18.33
1883	1998/41	1	0	0	1	0	0	0	0	0	0	0	0	1	0	0	0	0	0	1	0	0	0	0	0	0	0	0	1	0	0	1	0	0	0	0	0	0	0	0	0	0	0	0	0	0	0	0	1	42.6	18.23
1884	1998/42	0	0	0	0	0	0	0	0	0	0	0	0	0	0	0	0	0	0	0	0	1	0	0	0	0	0	0	0	0	0	1	0	0	0	0	0	0	0	0	0	0	0	0	0	0	0	0	1	42.59	18.28
1885	1998/43	0	0	0	0	0	0	0	0	0	0	0	0	0	0	1	0	0	0	0	0	0	0	0	0	0	0	0	0	0	0	1	0	0	0	0	0	0	0	0	0	0	0	0	0	0	0	0	1	42.6	18.07
1886	1998/44	1	0	0	1	0	0	0	0	0	0	0	0	1	0	1	0	0	0	0	0	0	0	0	0	0	0	0	1	1	0	1	0	0	0	0	0	1	0	0	0	0	0	1	0	0	0	0	1	42.8402	17.6956
1888	1998/46	1	0	0	0	0	0	0	0	0	0	0	0	0	0	0	0	0	0	0	0	0	0	0	0	0	0	0	0	0	0	0	0	0	0	0	0	0	0	0	0	0	1	0	0	0	0	0	1	43.41	16.9
1890	1998/48	0	0	0	0	0	0	0	0	0	0	0	0	0	0	0	0	0	0	0	0	0	0	0	0	0	0	0	0	0	0	0	0	0	0	0	0	0	0	0	0	0	1	0	0	0	0	0	1	43.48	16.84
1891	1998/49	1	0	0	0	0	0	0	0	0	0	0	0	1	0	0	0	0	0	0	0	0	0	0	0	0	0	0	1	0	0	0	0	0	0	0	0	0	0	0	0	0	1	0	0	0	0	0	1	43.44	16.9
1892	1998/50	1	0	0	0	0	0	0	0	0	0	0	0	1	0	0	0	0	0	1	0	0	0	0	0	0	0	0	0	0	0	0	0	0	0	0	0	0	0	0	0	0	1	0	0	0	0	0	1	43.34	17.11
1893	1998/51	0	0	0	0	0	0	0	0	0	0	0	0	0	0	1	0	0	0	1	0	0	0	0	0	0	0	0	1	0	0	0	0	0	0	0	0	0	0	0	0	0	0	0	0	0	0	0	1	43.4545	17.2
1895	1999/01	1	0	0	0	0	0	0	0	0	1	0	0	0	0	1	1	0	0	0	0	1	0	0	0	0	0	0	0	0	1	0	0	0	0	0	1	0	0	0	0	0	0	0	0	0	0	0	3	45.22	15.54
1897	1999/03	1	0	0	0	0	1	0	0	0	0	0	0	0	0	0	1	0	0	1	0	0	0	0	0	0	0	0	0	0	1	0	0	0	0	0	0	0	0	0	0	0	0	0	0	0	0	0	1	44.58	15.75
1898	1999/04	1	0	0	0	0	0	0	0	0	0	0	0	1	0	0	0	0	0	1	0	0	0	0	0	0	0	0	0	0	0	0	0	0	0	0	0	0	0	0	0	0	1	0	0	0	0	0	1	44.25	15.81
1899	1999/05	1	0	0	0	0	1	0	0	0	0	0	0	1	0	0	0	0	0	1	0	0	0	0	0	0	0	0	1	0	0	0	0	0	0	0	0	0	0	0	0	0	1	0	0	0	0	0	1	44.25	15.65
1901	1999/07	1	0	0	0	0	0	0	0	0	0	0	0	0	0	0	0	0	0	0	0	0	0	0	0	0	0	0	0	0	0	0	0	0	0	0	0	0	0	0	0	0	0	0	0	0	0	0	1	44.18	15.73
1902	1999/08	1	0	0	0	0	0	0	0	0	0	0	0	1	0	0	0	0	0	1	0	0	0	0	0	0	0	0	1	0	0	0	0	0	0	0	0	0	0	0	0	0	0	0	0	0	0	0	1	44.011	16.038
1904	1999/10	1	0	0	0	0	0	0	0	0	0	0	0	0	0	0	0	0	0	0	0	0	0	0	0	0	0	0	0	0	0	0	0	0	0	0	0	0	0	0	0	0	0	0	0	0	0	0	1	43.89	16.08
1905	1999/100	0	0	0	0	0	0	0	0	0	0	0	0	0	0	0	0	0	0	0	0	0	0	0	0	0	0	0	0	0	0	0	0	0	0	0	0	0	0	0	0	1	0	0	0	0	0	0	1	45.38	23.91
1907	1999/102	0	0	0	0	0	0	0	0	0	0	0	0	0	0	0	0	0	0	0	0	0	0	0	0	0	0	0	0	0	0	0	0	0	0	0	0	0	0	0	0	0	0	0	0	0	0	0	1	45.41135	23.913183
1908	1999/103	1	0	0	0	0	0	0	0	0	1	0	0	0	0	0	1	0	0	1	0	0	0	1	1	0	1	0	0	0	1	0	0	1	0	0	1	0	0	0	0	0	0	0	0	0	0	0	1	46.467367	22.842183
1910	1999/12	1	0	0	0	0	0	0	0	0	0	0	0	1	0	0	0	0	0	1	0	0	0	0	0	0	0	0	1	0	0	0	0	0	0	0	0	0	0	0	0	0	0	0	0	0	0	0	1	44.0335	16.1914
1912	1999/13	1	0	0	0	0	0	0	0	0	0	0	0	1	0	0	0	0	0	0	0	0	0	0	0	0	0	0	0	0	0	0	0	0	0	0	0	0	0	0	0	0	0	0	0	0	0	0	1	43.92	16.46
1914	1999/15	1	0	0	0	0	1	0	0	0	0	0	0	1	0	0	0	0	0	0	0	0	0	0	0	0	0	0	1	0	0	0	0	0	0	0	0	0	0	0	0	0	0	0	0	0	0	0	1	43.76	16.63
1916	1999/17	1	0	0	0	0	0	0	0	0	0	0	0	0	0	0	0	0	0	0	0	0	0	0	0	0	0	0	0	0	0	0	0	0	0	0	0	0	0	0	0	0	0	0	0	0	0	0	1	43.71	16.58
1917	1999/18	1	0	0	0	0	0	0	0	0	0	0	0	0	0	0	0	0	0	1	0	0	0	0	0	0	0	0	0	0	0	0	0	0	0	0	0	0	0	0	0	0	0	0	0	0	0	0	1	43.68	16.52
1918	1999/19	0	0	0	0	0	0	0	0	0	0	0	0	0	0	0	0	0	0	0	0	0	0	0	0	0	0	0	0	0	0	0	0	0	0	0	0	0	0	0	0	0	0	0	0	0	0	0	1	43.69	16.49
1919	1999/20	1	0	0	0	0	1	0	0	0	0	0	0	0	0	0	0	0	0	1	0	0	0	0	0	0	0	0	0	0	0	0	0	0	0	0	0	0	0	0	0	0	0	0	0	0	0	0	1	43.58	16.6
1922	1999/23	1	0	0	1	0	0	0	0	0	0	0	0	1	0	0	0	0	0	1	0	0	0	0	0	0	0	0	0	0	0	0	0	0	0	0	0	1	0	0	0	0	1	0	0	0	0	0	1	43.458	16.763
1923	1999/24	0	0	0	0	0	0	0	0	0	0	0	0	1	0	0	0	0	0	0	0	0	0	0	0	0	0	0	0	0	0	0	0	0	0	0	0	0	0	0	0	0	0	0	0	0	0	0	1	43.44	16.94
1926	1999/27	1	0	0	0	0	0	0	0	0	0	0	0	1	0	0	0	0	0	1	1	0	0	0	0	0	0	0	0	0	0	1	0	0	0	0	0	0	0	0	0	0	0	0	0	0	0	0	1	43.08	17.36
1927	1999/28	1	0	0	0	0	0	0	0	0	0	0	0	0	0	0	0	0	0	0	0	0	0	0	0	0	0	0	0	0	0	1	0	0	0	0	0	0	0	0	0	0	0	0	0	0	0	0	1	43.04	17.43
1928	1999/29	1	0	0	0	0	0	0	0	0	0	0	0	1	0	0	0	0	0	1	0	0	0	0	0	0	0	0	0	0	0	1	0	0	0	0	0	0	0	0	0	0	0	0	0	0	0	0	1	43.14	17.73
1929	1999/30	0	0	0	0	0	0	0	0	0	0	0	0	0	0	0	0	0	0	0	0	0	0	0	0	0	0	0	0	0	0	1	0	0	0	0	0	0	0	0	0	0	0	0	0	0	0	0	1	43.26	17.9
1930	1999/31	1	0	0	0	0	0	0	0	0	1	0	0	1	0	1	0	0	0	1	0	0	0	0	0	0	1	0	1	0	0	0	0	0	0	0	0	0	0	0	0	0	0	0	0	0	0	0	1	43.44	17.93
1931	1999/32	1	0	0	1	0	0	0	0	0	0	0	0	1	0	0	0	0	0	1	0	0	0	0	0	0	0	0	0	0	0	0	0	0	0	0	0	0	0	0	0	0	0	0	0	0	0	0	1	43.42	17.87
1932	1999/33	1	0	0	0	0	0	0	0	0	0	0	0	1	0	0	0	0	0	1	0	0	0	0	0	0	0	0	0	0	0	1	0	0	0	0	0	0	0	0	0	0	0	0	0	0	0	0	1	43.28	17.87
1933	1999/34	0	0	0	0	0	0	0	0	0	0	0	0	1	0	0	0	0	0	0	0	0	0	0	0	0	0	0	0	0	0	1	0	0	0	0	0	0	0	0	0	0	0	0	0	0	0	0	1	42.98	17.54
1936	1999/37	1	0	0	0	0	0	0	0	0	0	0	0	1	0	0	0	0	0	1	0	0	0	0	0	0	0	0	0	0	0	1	0	0	0	0	0	0	0	0	0	0	0	0	0	0	0	0	1	42.92	17.42
1937	1999/38	0	0	0	0	0	0	0	0	0	0	0	0	0	0	0	0	0	0	1	0	0	0	0	0	0	0	0	0	0	0	1	0	0	0	0	0	0	0	0	0	0	0	0	0	0	0	0	1	42.7	18.04
1938	1999/39	0	0	0	0	0	0	0	0	0	0	0	0	0	0	0	0	0	0	0	0	0	0	0	0	0	0	0	0	0	0	1	0	0	0	0	0	0	0	0	0	0	0	0	0	0	0	0	1	42.62	18.12
1939	1999/40	0	0	0	0	0	0	0	0	0	0	0	0	1	0	1	0	0	0	1	0	0	0	0	0	0	0	0	0	0	0	1	0	0	0	0	0	0	0	0	0	0	0	0	0	0	0	0	1	42.61	18.13
1940	1999/41	0	0	0	0	0	0	0	0	0	0	0	0	1	0	0	0	0	0	0	0	0	0	0	0	0	0	0	0	0	0	1	0	0	0	0	0	0	0	0	0	0	0	1	0	0	0	0	1	42.58	18.22
1941	1999/42	1	0	0	0	0	0	0	0	0	0	0	0	0	0	0	0	0	0	1	0	0	0	0	0	0	0	0	0	0	0	1	0	0	0	0	0	0	0	0	0	0	0	0	0	0	0	0	1	42.63	18.18
1942	1999/43	1	0	0	0	0	0	0	0	0	0	0	0	1	0	0	0	0	0	1	0	0	0	0	0	0	0	0	0	0	0	1	0	0	0	0	0	0	0	0	0	0	0	0	0	0	0	0	1	42.64	18.19
1943	1999/44	1	0	0	0	0	0	0	0	0	0	0	0	1	0	0	0	0	0	0	0	0	0	0	0	0	0	0	1	0	0	1	0	0	0	0	0	0	0	0	0	0	0	0	0	0	0	0	1	42.64	18.16
1944	1999/45	0	0	0	0	0	0	0	0	0	0	0	0	0	0	1	0	0	0	0	0	0	0	0	0	0	0	0	0	0	0	1	0	0	0	0	0	0	0	0	0	0	0	0	0	0	0	0	1	42.65	18.12
1945	1999/46	0	0	0	0	0	0	0	0	0	0	0	0	1	0	1	0	0	0	0	0	0	0	0	0	0	0	0	0	0	0	1	0	0	0	0	0	0	0	0	0	0	0	0	0	0	0	0	1	42.99	17.53
1948	1999/49	0	0	0	0	0	0	0	0	0	0	0	0	1	0	0	0	0	0	0	0	0	0	0	0	0	0	0	0	0	0	0	0	0	0	0	0	0	0	0	0	0	1	0	0	0	0	0	1	43.31	17.04
1949	1999/50	1	0	0	0	0	0	0	0	0	0	0	0	0	0	0	0	0	0	1	0	0	0	0	0	0	0	0	0	0	0	0	0	0	0	0	0	0	0	0	0	0	0	0	0	0	0	0	1	43.78	15.83
1952	1999/52	0	0	0	0	0	0	0	0	0	0	0	0	0	0	0	0	0	0	0	0	0	0	0	0	0	0	0	0	0	0	0	0	0	0	0	0	0	0	0	0	0	0	0	0	0	0	0	1	43.57	15.87
1954	1999/54	0	0	0	0	0	0	0	0	0	0	0	0	0	0	0	0	0	0	0	0	0	0	0	0	0	0	0	0	0	0	0	0	0	0	0	0	0	0	0	0	0	0	0	0	0	0	0	1	43.18	16.45
1955	1999/55	1	0	0	0	0	0	0	0	0	1	0	0	1	0	0	0	0	0	1	0	0	0	0	0	0	0	0	0	0	0	0	0	0	0	0	0	0	0	0	0	0	0	0	0	0	0	0	2	45.65	14.22
1956	1999/56	1	0	0	0	0	0	0	0	0	0	0	0	0	0	0	0	0	0	0	0	0	0	0	0	0	0	0	0	0	0	0	0	0	0	0	0	0	0	0	0	0	0	0	0	0	0	0	1	45.32	14.54
1957	1999/57	0	0	0	0	0	0	0	0	0	0	0	0	1	0	0	0	0	0	0	1	0	0	0	0	0	0	0	1	0	0	0	0	0	0	0	0	0	0	0	0	0	0	0	1	0	0	0	1	45.03	14.58
1958	1999/58	1	0	0	0	0	0	0	0	0	1	0	0	0	0	0	0	0	0	1	0	0	0	0	0	0	1	0	1	0	0	0	0	0	0	0	0	0	0	0	0	0	0	0	0	0	0	0	1	44.97	14.67
1959	1999/59	1	0	0	0	0	0	0	0	0	0	0	0	0	0	0	0	0	0	1	1	0	0	0	0	0	0	0	1	0	0	0	0	0	0	0	0	0	0	0	0	0	0	0	0	0	0	0	1	44.93	14.92
1960	1999/60	1	0	0	0	0	0	0	0	0	0	0	0	1	0	0	0	0	0	1	0	0	0	0	0	0	0	0	1	0	0	0	0	0	0	0	0	0	0	0	0	0	1	0	0	0	0	0	1	44.53	15.14
1961	1999/61	1	0	0	0	0	0	0	0	0	0	0	0	1	0	0	0	0	0	1	0	0	0	0	0	0	0	0	0	0	0	0	0	0	0	0	0	0	0	0	0	0	0	0	0	0	0	0	1	44.4	15.25
1962	1999/62	1	0	0	0	0	0	0	0	0	1	0	0	1	0	0	0	0	0	1	0	1	0	0	0	0	0	0	1	1	0	0	0	0	0	0	0	0	0	0	0	0	1	1	0	0	0	0	1	44.3	15.47
1963	1999/63	1	0	0	0	0	0	0	0	0	0	0	0	1	0	0	0	0	0	0	0	0	0	0	0	0	0	0	0	0	0	0	0	0	0	0	0	0	0	0	0	0	1	0	0	0	0	0	1	44.325	15.475
1964	1999/64	1	0	0	0	0	0	0	0	0	1	0	0	1	0	0	0	0	0	0	0	0	0	1	0	0	0	0	0	0	0	0	0	0	0	0	1	0	0	0	0	0	0	0	0	0	0	0	2	44.35	15.48
1965	1999/65	1	0	0	0	0	1	0	0	0	0	0	0	0	0	0	0	0	0	1	0	0	0	0	0	0	0	0	1	0	0	0	0	0	0	0	0	0	0	0	0	0	0	0	0	0	0	0	1	43.96	15.57
1966	1999/66	0	0	0	0	0	0	0	0	0	0	0	0	1	0	0	0	0	0	0	0	0	0	0	0	0	0	0	0	0	0	0	0	0	0	0	0	0	0	0	0	0	0	0	0	0	0	0	1	43.95	15.64
1968	1999/68	1	0	0	0	0	0	0	0	0	0	0	0	0	0	0	0	0	0	1	0	0	0	0	0	0	0	0	1	0	1	0	0	0	0	0	0	0	0	0	0	0	0	0	0	0	0	0	1	45.35	13.75
1969	1999/69	0	0	0	0	0	0	0	0	0	0	0	0	0	0	0	0	0	0	0	0	0	0	0	0	0	0	0	0	0	0	0	0	0	0	0	0	0	0	0	0	0	0	0	0	0	0	0	1	45.118607	13.807904
1971	1999/71	1	0	0	0	0	0	0	0	0	0	0	0	0	0	0	0	0	0	1	0	0	0	0	0	0	0	0	0	0	0	0	0	0	0	0	0	0	0	0	0	0	0	0	0	0	0	0	1	45.52	14.18
1972	1999/72	1	0	0	0	0	0	0	0	0	0	0	0	0	0	0	0	0	0	1	0	0	0	0	0	0	1	0	0	0	1	0	0	0	0	0	1	0	0	0	0	0	0	0	0	0	0	0	1	45.454033	14.282588
1973	1999/73	1	0	0	0	0	0	0	0	0	0	0	0	0	0	0	0	0	0	1	0	0	0	0	0	0	0	0	0	0	0	0	0	0	0	0	0	0	0	0	0	0	0	0	0	0	0	0	1	45.975406	13.650926
1974	1999/74	1	0	0	0	0	0	0	0	0	0	0	0	1	1	0	0	0	0	1	0	0	0	0	0	0	0	0	1	0	1	0	0	0	0	0	0	0	0	0	0	0	0	0	0	0	0	0	1	45.998094	13.635327
1975	1999/75	1	0	0	0	0	0	0	0	0	0	0	0	1	0	0	0	0	0	0	0	0	0	0	0	0	0	0	0	0	0	0	0	0	0	0	0	0	0	0	0	0	0	0	0	0	0	0	1	46.106428	13.683212
1977	1999/77	1	0	0	0	0	0	0	0	0	0	0	0	1	1	0	0	0	0	0	0	0	0	0	0	0	0	0	0	0	0	0	0	0	0	0	0	0	0	0	0	0	0	0	0	0	0	0	1	46.250935	13.581232
1978	1999/78	1	0	0	0	0	0	0	0	0	0	0	0	0	0	0	0	0	0	1	0	1	0	0	0	0	0	0	1	0	1	0	0	0	0	0	0	0	0	0	0	0	0	0	0	0	0	0	1	45.87	13.9
1980	1999/80	1	0	0	0	0	0	0	0	0	0	0	0	1	0	0	0	0	0	0	0	0	0	0	0	0	0	0	0	0	0	0	0	0	0	0	0	0	0	0	0	0	0	0	0	0	0	0	1	46.27	13.88
1981	1999/88	1	0	0	0	0	0	0	0	0	0	0	0	1	0	0	0	0	0	1	0	0	0	0	0	0	0	0	0	0	0	0	0	0	0	0	0	0	0	0	0	0	0	0	0	0	0	0	1	46.42	15.26
1982	1999/89	1	0	0	0	0	0	0	0	0	0	0	0	1	0	0	0	0	0	0	0	0	0	0	0	0	0	0	0	0	0	0	0	0	0	0	0	0	0	0	0	0	0	0	0	0	0	0	1	46.34	13.56
1983	1999/90	1	0	0	0	0	0	0	0	0	0	0	0	1	0	0	0	0	0	1	0	0	0	0	0	0	0	0	0	0	0	0	0	0	0	0	0	0	0	0	0	0	0	0	0	0	0	0	1	46.3	13.48
1984	1999/92	0	0	0	0	0	0	0	0	0	1	0	0	0	0	0	0	0	0	1	0	0	0	1	1	0	0	0	0	0	1	0	0	0	0	0	1	0	0	0	0	0	0	0	0	0	0	0	1	46.04	22.87
1985	1999/93	0	0	0	0	0	0	0	0	0	0	0	0	0	0	0	0	0	0	1	0	0	0	0	0	0	0	0	0	0	1	0	0	1	0	0	0	0	0	0	0	0	0	0	0	0	0	0	1	45.76	22.89
1986	1999/94	0	0	0	0	0	0	0	0	0	0	0	0	0	0	0	0	0	0	1	0	0	0	1	0	0	0	0	0	0	0	0	0	0	0	0	0	0	0	0	0	0	0	0	0	0	0	0	1	45.53	23.14
1987	1999/95	0	0	0	0	0	0	0	0	0	1	0	0	0	0	0	1	0	0	1	0	0	0	1	1	0	1	0	0	0	1	0	0	0	0	0	1	0	0	0	0	0	0	0	0	0	0	0	1	45.45	23.32
1988	1999/96	0	0	0	0	0	0	0	0	0	1	0	0	0	0	0	0	0	0	1	0	0	1	0	0	0	1	0	0	0	1	0	0	0	0	0	1	0	0	0	0	0	0	0	0	0	0	0	1	45.47	23.18
1990	1999/98	0	0	0	0	0	0	0	0	0	1	0	0	0	0	0	0	0	0	1	0	0	0	1	1	0	0	0	0	0	1	0	0	1	0	0	0	0	0	0	0	1	0	0	0	0	0	0	1	45.16	24.05
1991	1999/99	0	0	0	0	0	0	0	0	0	0	0	0	0	0	0	0	0	0	1	0	0	0	0	0	0	0	0	0	0	1	0	0	1	0	0	1	0	0	0	0	1	0	0	0	0	0	0	1	45.22	24.22
1992	2000/01	0	0	0	0	0	0	0	0	0	0	0	0	1	0	0	0	0	0	1	0	0	0	0	0	0	0	0	0	0	0	0	0	0	0	0	0	0	0	1	0	0	0	0	0	0	0	0	1	44.68653333	17.1946
1993	2000/02	0	0	0	0	0	0	0	0	0	0	0	0	0	0	0	0	0	0	1	0	0	0	0	0	0	0	0	0	0	0	0	0	0	0	0	0	0	0	1	0	0	0	0	0	0	0	0	1	44.60721667	17.13863333
1994	2000/03	1	0	0	0	0	0	0	0	0	1	0	0	0	0	0	0	0	0	1	0	0	0	0	0	0	0	0	0	0	1	0	0	0	0	0	0	0	0	1	0	0	0	0	0	0	0	0	1	44.47268333	17.19521667
1995	2000/04	1	0	0	0	0	0	0	0	0	0	0	0	0	0	0	0	0	0	1	0	0	0	0	0	0	0	0	0	0	1	0	0	0	0	0	0	1	0	0	0	0	0	0	0	0	0	0	1	44.4105	17.2607
1996	2000/05	1	0	0	0	0	1	0	0	0	0	0	0	1	0	0	0	0	0	1	0	0	0	0	0	0	0	0	0	0	1	0	0	0	0	0	0	0	0	0	0	0	0	0	0	0	0	0	1	44.347	17.263
1998	2000/07	0	0	0	0	0	0	0	0	0	0	0	0	0	0	0	0	0	0	1	0	0	0	0	0	0	0	0	1	0	0	0	0	0	0	0	0	0	0	0	0	0	0	0	0	0	0	0	1	44.22948333	17.66846667
1999	2000/08	1	0	0	0	0	0	0	0	0	0	0	0	1	0	0	0	0	0	1	0	0	0	0	0	0	0	0	0	0	1	0	0	0	0	0	1	0	0	0	0	0	0	0	0	0	0	0	2	44.27886667	17.59206667
2002	2000/11	1	0	0	0	0	0	0	0	0	0	0	0	1	0	0	0	0	0	1	0	0	0	0	0	0	0	0	0	0	1	0	0	0	0	0	0	0	0	1	0	0	0	0	0	0	0	0	1	43.76993333	18.39406667
2003	2000/12	1	0	0	0	0	0	0	0	0	0	0	0	1	0	0	0	0	0	1	0	0	0	0	0	0	0	0	0	0	0	0	0	0	0	0	1	0	0	1	0	0	0	0	0	0	0	0	1	43.70976667	18.43921667
2004	2000/13	1	0	0	0	0	0	0	0	0	0	0	0	0	0	0	1	0	0	1	0	0	0	0	0	0	0	0	0	0	1	0	0	0	0	0	0	0	0	0	0	0	0	0	0	0	0	0	1	43.57601667	18.53733333
2005	2000/62	1	0	0	0	0	1	0	0	0	1	0	0	1	0	0	0	0	0	0	0	0	0	0	0	0	0	0	0	0	0	1	0	0	0	0	0	0	0	0	0	0	0	0	1	0	0	0	1	42.7	18.32
2006	2000/63	0	0	0	0	0	1	0	0	0	0	0	0	1	0	0	0	0	0	0	0	0	0	0	0	0	0	0	0	1	0	0	0	0	0	0	0	0	0	1	0	0	0	0	0	0	0	0	1	43.055	18.506
2007	2000/64	0	0	0	0	0	1	0	0	0	0	0	0	0	0	0	0	0	0	0	0	0	0	0	0	0	0	0	0	0	0	0	0	0	0	0	0	0	0	1	0	0	0	0	0	0	0	0	1	43.231649	18.40225
2008	2000/65	0	0	0	0	0	0	0	0	0	0	0	0	0	0	1	0	0	0	0	0	0	0	0	0	0	0	0	0	0	0	0	0	0	0	0	0	0	0	1	0	0	0	0	0	0	0	0	1	43.24776667	18.38158333
2009	2000/66	0	0	0	0	0	0	0	0	0	0	0	0	1	0	0	0	0	0	0	0	0	0	0	0	0	0	0	0	0	0	0	0	0	0	0	0	0	0	1	0	0	0	0	0	0	0	0	1	43.25101667	18.33955
2010	2000/67	1	0	0	0	0	0	0	0	0	1	0	0	0	0	0	0	0	0	1	0	0	0	0	0	0	0	0	0	0	0	0	0	0	0	0	0	0	0	0	0	0	0	0	0	0	0	0	1	43.35058333	17.89568333
2011	2000/68	0	0	0	0	0	0	0	0	0	0	0	0	0	0	0	0	0	0	1	0	0	0	0	0	0	0	0	0	0	0	0	0	0	0	0	0	0	0	1	0	0	0	0	0	0	0	0	1	43.6674	17.95896667
2012	2000/86	0	0	0	0	0	0	0	0	0	1	0	0	0	0	0	0	0	0	0	0	0	0	0	0	0	0	0	0	0	1	0	0	1	0	0	1	0	0	0	0	1	0	0	0	0	0	0	1	45.35648333	23.85803333
2013	2000/87	0	0	0	0	0	0	0	0	0	1	0	0	0	0	0	0	0	0	1	0	0	0	1	0	0	0	0	0	0	1	0	0	1	0	0	0	0	0	0	0	1	0	0	0	0	0	0	1	45.41753333	23.9158
2014	2000/88	0	0	0	0	0	0	0	0	0	0	0	0	0	0	0	0	0	0	1	0	0	0	0	0	0	0	0	0	0	0	0	0	1	0	0	1	0	0	0	0	0	0	0	0	0	0	0	1	45.39733333	23.904
2015	2000/88B	0	0	0	0	0	0	0	0	0	0	0	0	0	0	0	0	0	0	0	0	0	0	0	0	0	0	0	0	0	0	0	0	0	0	0	0	0	0	0	0	1	0	0	0	0	0	0	1	45.39445	23.91086667
2016	2000/89	0	0	0	0	0	0	0	0	0	1	0	0	0	0	0	0	0	0	1	0	0	0	1	0	0	0	0	0	0	1	0	0	1	0	0	1	0	0	0	0	0	0	0	0	0	0	0	1	45.55243333	25.89996667
2017	2000/90	0	0	0	0	0	0	0	0	0	0	0	0	0	0	0	0	0	0	0	0	0	0	0	0	0	0	0	0	0	0	0	0	1	0	0	0	0	0	0	0	0	0	0	0	0	0	0	1	45.56693333	25.88425
2018	2000/91	0	0	0	0	0	0	0	0	0	0	0	0	0	0	0	0	0	0	0	0	0	0	0	0	0	0	0	0	0	1	0	0	1	0	0	1	0	0	0	0	0	0	0	0	0	0	0	1	45.5461	25.8905
2019	2000/92	0	0	0	0	0	0	0	0	0	0	0	0	0	0	0	0	0	0	0	0	0	0	0	0	0	0	0	0	0	1	0	0	1	0	0	0	0	0	0	0	0	0	0	0	0	0	0	1	45.55161667	25.90681667
2020	2000/93	0	0	0	0	0	0	0	0	0	0	0	0	0	0	0	0	0	0	0	0	0	0	1	0	0	0	0	0	0	1	0	0	1	0	0	0	0	0	0	0	0	0	0	0	0	0	0	1	45.56238333	25.93621667
2021	2000/94	0	0	0	0	0	0	0	0	0	0	0	0	0	0	0	0	0	0	0	0	0	0	0	0	0	0	0	0	0	1	0	0	1	0	0	0	0	0	0	0	0	0	0	0	0	0	0	1	45.55963333	25.89625
2022	2000/95	0	0	0	0	0	0	0	0	0	0	0	0	0	0	0	0	0	0	0	0	0	0	0	0	0	0	0	0	0	1	0	0	1	0	0	0	0	0	0	0	0	0	0	0	0	0	0	1	45.55736667	25.89158333
2023	2000/96	0	0	0	0	0	0	0	0	0	0	0	0	0	0	0	0	0	0	0	0	0	0	0	0	0	0	0	0	0	0	0	0	1	0	0	0	0	0	0	0	0	0	0	0	0	0	0	1	45.56066667	25.8836
2024	2000/98	0	0	0	0	0	0	0	0	0	0	0	0	0	0	0	0	0	0	1	0	0	0	1	0	0	0	0	0	0	0	0	0	1	0	0	0	0	0	0	0	0	0	0	0	0	0	0	1	45.53561667	25.90751667
2025	2000/99	0	0	0	0	0	0	0	0	0	0	0	0	0	0	0	0	0	0	0	0	0	0	0	0	0	0	0	0	0	1	0	0	1	0	0	0	0	0	0	0	0	0	0	0	0	0	0	1	45.56756667	25.60383333
2027	2002/001	0	0	0	0	0	1	0	0	0	0	0	0	1	0	0	0	0	0	1	0	0	0	0	0	0	0	0	0	0	0	0	0	0	1	0	0	0	0	0	0	0	0	0	0	0	0	0	1	43.333195	22.141906

2028	2002/002	0	0	0	0	0	1	0	0	0	0	0	0	1	0	0	0	0	0	1	0	0	0	0	0	0	0	0	0	0	1	0	0	0	1	0	0	0	0	0	0	0	0	0	0	0	0	0	1	43.33568333	22.13305
2030	2002/056	1	0	0	0	0	0	0	0	0	0	0	0	1	0	0	0	0	0	0	0	0	0	0	0	0	0	0	0	0	1	0	0	0	0	0	1	0	0	0	0	0	0	0	0	0	0	0	1	45.36228	14.760714
2031	2002/057	0	0	0	0	0	1	0	0	0	0	0	0	1	0	0	0	0	0	0	0	0	0	0	0	0	0	0	0	0	0	0	0	0	0	0	1	0	0	0	0	0	0	0	0	0	0	0	1	45.30163333	14.67318333
2032	2002/058	1	0	0	0	0	1	0	0	0	0	0	0	1	0	0	0	0	0	0	0	0	0	0	0	0	0	0	1	0	1	0	0	0	0	0	1	0	0	0	0	0	0	0	0	0	0	0	1	45.29755	14.65805
2033	2002/059	1	0	0	0	0	0	0	0	0	0	0	0	1	0	0	0	0	0	1	0	0	0	0	0	0	0	0	0	0	0	0	0	0	0	0	0	0	0	0	0	0	1	0	0	0	0	0	1	45.28635	14.64531667
2034	2002/060	1	0	0	0	0	1	0	0	0	0	0	0	1	0	0	0	0	0	0	0	0	0	0	0	0	0	0	1	0	0	0	0	0	0	0	0	0	0	0	0	0	1	0	0	0	0	0	1	45.28463333	14.62855
2035	2002/061	0	0	0	0	0	0	0	0	0	0	0	0	0	0	0	0	0	0	1	0	0	0	0	0	0	0	0	0	0	0	0	0	0	0	0	0	0	0	0	0	0	0	0	0	0	0	0	1	45.324709	14.538775
2036	2002/062	1	0	0	0	0	1	0	0	0	1	0	0	1	0	0	0	0	0	1	0	0	0	0	0	0	1	0	0	0	0	0	0	0	0	0	0	0	0	0	0	0	0	0	0	0	0	0	1	45.33175	14.54301667
2037	2002/063	1	0	0	0	0	0	0	0	0	0	0	0	1	0	0	0	0	0	0	0	0	0	0	0	0	0	0	0	0	0	0	0	0	0	0	0	0	0	0	0	0	1	0	0	0	0	0	1	44.98261667	14.9754
2038	2002/064	0	0	0	0	0	0	0	0	0	0	0	0	1	0	0	0	0	0	1	0	0	0	0	0	0	0	0	1	0	0	0	0	0	0	0	0	0	0	0	0	0	0	0	0	0	0	0	1	44.96866	14.973473
2039	2002/065	1	0	0	0	0	0	0	0	0	0	0	0	0	0	0	0	0	0	1	1	0	0	0	0	0	0	0	1	0	0	0	0	0	0	0	0	0	0	0	0	0	1	0	0	0	0	0	1	44.97933333	14.94933333
2040	2002/066	1	0	0	0	0	1	0	0	0	0	0	0	1	0	0	0	0	0	1	0	0	0	0	0	0	0	0	0	0	0	0	0	0	0	0	0	0	1	0	0	0	1	0	0	0	0	0	1	44.52836667	15.14398333
2041	2002/067	0	0	0	0	0	1	0	0	0	0	0	0	0	0	0	0	0	0	0	0	0	0	0	0	0	0	0	0	0	0	0	0	0	0	0	0	1	0	0	0	0	0	0	0	0	0	0	1	44.44111667	15.51913333
2042	2002/068	1	0	0	0	0	1	0	0	0	0	0	0	1	0	0	0	0	0	1	0	0	0	0	0	0	0	0	1	0	0	0	0	0	0	0	0	1	0	0	0	0	1	0	0	0	0	0	1	44.20158333	15.69965
2043	2002/069	0	0	0	0	0	0	0	0	0	0	0	0	1	0	0	0	0	0	0	0	0	0	0	0	0	0	0	1	0	0	0	0	0	0	0	0	1	0	0	0	0	0	0	0	0	0	0	1	43.93758333	15.44123333
2044	2002/070	1	0	0	0	0	0	0	0	0	0	0	0	1	0	0	0	0	0	1	1	0	0	0	0	0	0	0	1	0	0	0	0	0	0	0	0	0	0	0	0	0	0	0	0	0	0	0	1	43.5279	15.9689
2045	2002/071	1	0	0	0	0	1	0	0	0	0	0	0	0	0	0	0	0	0	0	0	0	0	0	0	0	1	0	0	0	0	0	0	0	0	0	0	1	0	0	0	0	0	0	0	0	0	0	1	43.55986667	16.40255
2046	2002/072	1	0	0	1	0	0	0	0	0	0	0	0	0	0	0	0	0	0	1	1	0	0	0	0	0	0	0	1	0	0	0	0	0	0	0	0	0	0	0	0	0	0	0	0	0	0	0	1	43.5092	16.42173333
2047	2002/073	0	0	0	0	0	1	0	0	0	0	0	0	0	0	0	0	0	0	1	0	0	0	0	0	0	0	0	1	0	0	0	0	0	0	0	0	1	0	0	0	0	0	0	0	0	0	0	1	43.56105	16.5228
2048	2002/074	1	0	0	0	0	1	0	0	0	0	0	0	0	0	0	0	0	0	1	0	0	0	0	0	0	0	0	0	0	0	0	0	0	0	0	0	1	0	0	0	0	0	0	0	0	0	0	1	43.56001667	16.51918333
2049	2002/075	0	0	0	0	0	0	0	0	0	0	0	0	0	0	0	0	0	0	1	0	0	0	0	0	0	0	0	1	0	0	0	0	0	0	0	0	0	0	0	0	0	1	0	0	0	0	0	1	43.56223333	16.52906667
2050	2002/076	1	0	0	1	0	0	0	0	0	0	0	0	1	0	1	0	0	0	1	0	1	0	1	0	0	0	0	1	0	0	0	0	0	0	0	0	1	0	0	0	0	1	0	0	0	0	0	1	43.44803333	16.69458333
2051	2002/077	0	0	0	0	0	0	0	0	0	0	0	0	0	0	0	0	0	0	0	0	0	0	0	0	0	0	0	0	0	0	0	0	0	0	0	0	0	0	0	0	0	0	0	0	0	0	0	1	43.42926667	16.86666667
2052	2002/078	1	0	0	1	0	1	0	0	0	0	0	0	1	0	0	0	0	0	1	0	0	0	0	0	0	0	0	1	0	0	0	0	0	0	0	0	0	0	0	0	0	1	0	0	0	0	0	1	43.43131667	16.88768333
2053	2002/079	1	0	0	1	0	0	0	0	0	0	0	0	0	0	0	0	0	0	1	0	0	0	0	0	0	0	0	1	0	0	0	0	0	0	0	0	1	0	0	0	0	1	0	0	0	0	0	1	43.40301667	16.89371667
2054	2002/080	1	0	0	0	0	0	0	0	0	0	0	0	1	0	0	0	0	0	1	0	0	0	0	0	0	0	0	0	0	0	0	0	0	0	0	0	0	0	0	0	0	1	0	0	0	0	0	1	43.29345	17.0519
2055	2002/081	1	0	0	0	0	0	0	0	0	0	0	0	0	0	0	0	0	0	1	0	0	0	0	0	0	0	0	0	0	0	0	0	0	0	0	0	0	0	0	0	0	0	0	0	0	0	0	1	43.25765	17.09398333
2056	2002/082	0	0	0	0	0	1	0	0	0	0	0	0	0	0	0	0	0	0	0	0	0	0	0	0	0	0	0	0	0	0	0	0	0	0	0	0	0	0	0	0	0	1	0	0	0	0	0	1	43.31243333	17.06221667
2057	2002/083	1	0	0	0	0	0	0	0	0	0	0	0	1	0	0	0	0	0	1	0	0	0	0	0	0	0	0	0	0	0	0	0	0	0	0	0	1	0	0	0	0	1	0	0	0	0	0	1	43.31596667	17.05396667
2058	2002/084	0	0	0	0	0	0	0	0	0	0	0	0	0	0	0	0	0	0	1	0	0	0	0	0	0	0	0	1	0	0	0	0	0	0	0	0	0	0	0	0	0	0	0	0	0	0	0	1	43.2586	17.10341667
2059	2002/085	1	0	0	0	0	0	0	0	0	0	0	0	1	0	0	0	0	0	1	0	0	0	0	0	0	0	0	1	0	0	0	0	0	0	0	0	0	0	0	0	0	1	0	0	0	0	0	1	43.23421667	17.1046
2060	2002/086	0	0	0	0	0	0	0	0	0	0	0	0	1	0	0	0	0	0	0	0	0	0	0	0	0	0	0	1	0	0	0	0	0	0	0	0	0	0	0	0	0	1	0	0	0	0	0	1	43.22788333	17.10986667
2061	2002/087	1	0	0	0	0	0	0	0	0	0	0	0	0	0	0	0	0	0	1	0	0	0	0	0	0	0	0	0	0	0	0	0	0	0	0	0	0	0	0	0	0	1	0	0	0	0	0	1	43.44278333	16.70153333
2062	2002/088	0	0	0	0	0	0	0	0	0	0	0	0	1	0	0	0	0	0	1	0	0	0	0	0	0	0	0	0	0	0	0	0	0	0	0	0	0	0	0	0	0	1	0	0	0	0	0	1	43.40358333	16.88048333
2063	2002/089	0	0	0	0	0	0	0	0	0	0	0	0	1	0	0	0	0	0	1	0	0	0	0	0	0	0	0	1	0	0	0	0	0	0	0	0	1	0	0	0	0	1	0	0	0	0	0	1	43.83178333	17.00788333
2064	2002/090	0	0	0	0	0	1	0	0	0	0	0	0	0	0	0	0	0	0	1	0	0	0	0	0	0	0	0	1	0	0	0	0	0	0	0	0	0	0	0	0	0	0	0	0	0	0	0	1	44.34876667	16.36025
2065	2002/091	1	0	0	0	0	0	0	0	0	0	0	0	1	0	0	0	0	0	1	0	0	1	0	0	0	0	0	0	0	1	0	0	0	0	0	0	0	0	0	0	0	0	0	0	0	0	0	1	44.17978333	19.87128333
2066	2004/071	1	0	0	0	0	0	0	0	0	0	0	0	1	0	0	0	0	0	0	0	0	0	0	0	0	0	0	0	0	0	0	0	0	0	0	0	0	0	0	0	0	0	0	1	0	0	0	1	45.334736	13.827767
2067	2004/072	1	0	0	0	0	1	0	0	0	0	0	0	0	1	0	0	0	0	1	1	0	0	0	0	0	1	0	0	0	1	0	0	0	0	0	1	0	0	0	0	0	0	0	0	0	0	0	1	45.368641	14.015313
2068	2004/073	0	0	0	0	0	0	0	0	0	0	0	0	1	0	0	0	0	0	0	0	0	0	0	0	0	0	0	0	0	0	0	0	0	0	0	0	0	0	0	0	0	0	0	0	0	0	0	1	45.348651	14.050464
2071	2005/025	0	0	0	0	0	1	0	0	0	0	0	0	0	0	0	0	0	0	1	0	0	0	0	0	0	0	0	0	0	0	0	0	1	0	0	0	0	0	0	0	0	0	0	0	0	0	0	1	46.56373333	23.6796
2072	2005/039	0	0	0	0	0	0	0	0	0	0	0	0	0	0	0	0	0	0	0	0	0	0	0	0	0	0	0	0	0	0	0	0	0	0	0	0	0	0	0	0	0	0	0	0	0	0	0	3	45.171	19.82625
2073	2005/040	0	0	0	0	0	0	0	0	0	0	0	0	0	0	0	0	0	0	1	0	0	0	0	0	0	0	0	0	0	0	0	0	0	0	0	0	0	0	1	0	0	0	0	0	0	0	0	1	44.16038333	19.88308333
2082	2006/096	0	0	0	0	0	0	0	0	0	0	0	0	0	0	0	0	0	0	1	0	0	0	0	0	0	0	0	0	0	0	0	0	0	0	0	0	0	0	0	0	0	0	0	0	0	0	0	1	43.9065	15.97451667
2087	2006/101	0	0	0	0	0	0	0	0	0	0	0	0	0	0	0	0	0	0	1	0	0	0	0	0	0	0	0	0	0	0	0	0	0	0	0	0	0	0	0	0	0	0	0	0	0	0	0	1	43.3814	16.9108
2100	2007/004	0	0	0	0	0	0	0	0	0	0	0	0	0	0	0	0	0	0	0	0	0	0	0	0	0	0	0	0	0	0	0	0	0	0	0	0	0	0	0	0	0	0	0	0	0	0	0	1	41.28333333	23.33323333
2102	2007/006	0	0	0	0	0	0	0	0	0	0	0	0	0	0	0	0	0	0	0	0	0	0	0	0	0	0	0	0	0	1	0	0	0	1	0	0	0	0	0	0	0	0	0	0	0	0	0	3	41.39915	23.50523333
2103	2007/007	0	0	0	0	0	0	0	0	0	0	0	0	0	0	0	0	0	0	0	0	0	0	0	0	0	0	0	0	0	0	0	0	0	0	0	0	0	0	0	0	0	0	0	0	0	0	0	3	41.39273333	23.4776
2109	2007/013	0	0	0	0	0	0	0	0	0	0	0	0	0	0	0	0	0	0	0	0	0	0	0	0	0	0	0	0	0	1	0	0	0	1	0	0	0	0	0	0	1	0	0	0	0	0	0	1	41.34541667	23.67438333
2111	2007/015	0	0	0	0	0	0	0	0	0	0	0	0	0	0	0	0	0	0	0	0	0	0	0	0	0	0	0	0	0	0	0	0	0	1	0	0	0	0	0	0	0	0	0	0	0	0	0	1	41.28466667	23.85523333
2112	2007/016	0	0	0	0	0	0	0	0	0	0	0	0	0	0	0	0	0	0	0	0	0	0	0	0	0	0	0	0	0	1	0	0	0	0	0	0	0	0	0	0	0	0	0	0	0	0	0	1	41.2211	23.89316667
2113	2007/017	0	0	0	0	0	0	0	0	0	0	0	0	0	0	0	0	0	0	0	0	0	0	0	0	0	0	0	0	0	0	0	0	0	1	0	0	0	0	0	0	0	0	0	0	0	0	0	2	41.28955	24.02718333
2114	2007/018	0	0	0	0	0	0	0	0	0	0	0	0	0	0	0	0	0	0	0	0	0	0	0	0	0	0	0	0	0	0	0	0	0	1	0	0	0	0	0	0	0	0	0	0	0	0	0	3	41.29303333	24.00703333
2117	2007/021	0	0	0	0	0	0	0	0	0	0	0	0	0	0	0	0	0	0	0	0	0	0	0	0	0	0	0	0	0	0	0	0	0	0	0	0	0	0	0	0	0	0	0	0	0	0	0	1	41.38876667	24.16796667
2119	2007/023	0	0	0	0	0	0	0	0	0	0	0	0	0	0	0	0	0	0	0	0	0	0	0	0	0	0	0	0	0	0	0	0	0	0	0	0	0	0	0	0	0	0	0	0	0	0	0	1	41.42803333	24.21821667
2127	2007/031	0	0	0	0	0	0	0	0	0	0	0	0	0	0	0	0	0	0	0	0	0	0	0	0	0	0	0	0	0	0	0	0	0	0	0	0	0	0	0	0	0	0	0	0	0	0	0	1	41.30973333	24.5066
2128	2007/032	0	0	0	0	0	0	0	0	0	0	0	0	0	0	0	0	0	0	0	0	0	0	0	0	0	0	0	0	0	0	0	0	0	0	0	0	0	0	0	0	0	0	0	0	0	0	0	2	41.1662	24.64311667
2129	2007/033	0	0	0	0	0	0	0	0	0	0	0	0	0	0	0	0	0	0	0	0	0	0	0	0	0	0	0	0	0	0	0	0	0	1	0	0	0	0	0	0	0	0	0	0	0	0	0	1	41.15873333	24.60915
2130	2007/034	0	0	0	0	0	0	0	0	0	0	0	0	0	0	0	0	0	0	0	0	0	0	0	0	0	0	0	0	0	0	0	0	0	1	0	0	0	0	0	0	0	0	0	0	0	0	0	1	41.03515	24.63235
2131	2007/035	0	0	0	0	0	0	0	0	0	0	0	0	0	0	0	0	0	0	0	0	0	0	0	0	0	0	0	0	0	1	0	0	0	1	0	0	0	0	0	0	0	0	0	0	0	0	0	1	41.02708333	24.63613333
2137	2007/041	0	0	0	0	0	0	0	0	0	0	0	0	0	0	0	0	0	0	0	0	0	0	0	0	0	0	0	0	0	0	0	0	0	1	0	0	0	0	0	0	0	0	0	0	0	0	0	1	41.09325	24.7713
2139	2007/043	0	0	0	0	0	0	0	0	0	0	0	0	0	0	0	0	0	0	0	0	0	0	0	0	0	0	0	0	0	0	0	0	0	0	0	0	0	0	0	0	0	0	0	0	0	0	0	1	41.20825	24.86253333
2142	2007/046	0	0	0	0	0	0	0	0	0	0	0	0	0	0	0	0	0	0	0	0	0	0	0	0	0	0	0	0	0	0	0	0	0	0	0	0	0	0	0	0	0	0	0	0	0	0	0	3	41.29185	24.82905
2143	2007/047	0	0	0	0	0	0	0	0	0	0	0	0	0	0	0	0	0	0	0	0	0	0	0	0	0	0	0	0	0	0	0	0	0	0	0	0	0	0	0	0	0	0	0	0	0	0	0	1	41.29141667	24.84611667
2145	2007/048	0	0	0	0	0	0	0	0	0	0	0	0	0	0	0	0	0	0	0	0	0	0	0	0	0	0	0	0	0	0	0	0	0	0	0	0	0	0	0	0	0	0	0	0	0	0	0	1	41.24546667	24.90455
2146	2007/049	0	0	0	0	0	0	0	0	0	0	0	0	0	0	0	0	0	0	0	0	0	0	0	0	0	0	0	0	0	0	0	0	0	0	0	0	0	0	0	0	0	0	0	0	0	0	0	1	41.24673333	24.9137
2149	2007/052	0	0	0	0	0	0	0	0	0	0	0	0	0	0	0	0	0	0	0	0	0	0	0	0	0	0	0	0	0	1	0	0	0	0	0	0	0	0	0	0	0	0	0	0	0	0	0	1	41.18175	25.28118333
2151	2007/054	0	0	0	0	0	0	0	0	0	0	0	0	0	0	0	0	0	0	0	0	0	0	0	0	0	0	0	0	0	0	0	0	0	0	0	0	0	0	0	0	0	0	0	0	0	0	0	1	41.1902	25.29586667
2152	2007/055	0	0	0	0	0	0	0	0	0	0	0	0	0	0	0	0	0	0	0	0	0	0	0	0	0	0	0	0	0	1	0	0	0	0	0	0	0	0	0	0	0	0	0	0	0	0	0	1	41.11408333	25.8388
2154	2007/057	0	0	0	0	0	0	0	0	0	0	0	0	0	0	0	0	0	0	0	0	0	0	0	0	0	0	0	0	0	1	0	0	0	0	0	0	0	0	0	0	0	0	0	0	0	0	0	1	40.93728333	25.9117
2156	2007/059	0	0	0	0	0	0	0	0	0	0	0	0	0	0	0	0	0	0	0	0	0	0	0	0	0	0	0	0	0	1	0	0	0	0	0	0	0	0	0	0	0	0	0	0	0	0	0	2	40.90133333	25.6058
2157	2007/060	0	0	0	0	0	0	0	0	0	0	0	0	0	0	0	0	0	0	0	0	0	0	0	0	0	0	0	0	0	1	0	0	0	0	0	0	0	0	0	0	0	0	0	0	0	0	0	1	40.92886667	25.5023
2160	2007/063	0	0	0	0	0	0	0	0	0	0	0	0	0	0	0	0	0	0	0	0	0	0	0	0	0	0	0	0	0	0	0	0	0	0	0	0	0	0	0	0	0	0	0	0	0	0	0	1	40.64511667	27.01278333
2173	2007/076	0	0	0	0	0	0	0	0	0	0	0	0	0	0	0	0	0	0	0	0	0	0	0	0	0	0	0	0	0	1	0	0	0	0	0	0	0	0	0	0	0	0	0	0	0	0	0	1	41.83931667	27.55618333
2175	2007/078	0	0	0	0	0	0	0	0	0	0	0	0	0	0	0	0	0	0	0	0	0	0	0	0	0	0	0	0	0	0	0	0	0	0	0	0	0	0	0	0	0	0	0	0	0	0	0	3	42.02935	27.47363333
2176	2007/079	0	0	0	0	0	0	0	0	0	0	0	0	0	0	0	0	0	0	0	0	0	0	0	0	0	0	0	0	0	0	0	0	0	0	0	0	0	0	0	0	0	0	0	0	0	0	0	1	42.08348333	27.4277
2188	2008/084	0	0	0	0	0	0	0	0	0	0	0	0	0	0	0	0	0	0	0	0	0	0	0	0	0	0	0	0	0	0	0	0	0	0	0	0	0	0	0	0	0	0	0	0	0	0	0	1	44.51944444	14.5075
2189	2008/085	0	0	0	0	0	0	0	0	0	0	0	0	0	0	0	0	0	0	0	0	0	0	0	0	0	0	0	0	0	0	0	0	0	0	0	0	0	0	0	0	0	0	0	0	0	0	0	1	44.69333333	14.39222222
2190	2008/086	1	0	0	0	0	0	0	0	0	0	0	0	0	0	0	0	0	0	1	0	0	0	0	0	0	0	0	0	0	0	0	0	0	0	0	0	0	0	0	0	0	0	0	0	0	0	0	1	44.908	14.35593333
2191	2008/087	1	0	0	0	0	0	0	0	0	0	0	0	0	0	0	0	0	0	1	0	0	0	0	0	0	0	0	0	0	0	0	0	0	0	0	0	0	0	0	0	0	0	0	0	0	0	0	1	44.88881667	14.33096667
2192	2008/088	1	0	0	0	0	0	0	0	0	0	0	0	0	0	0	0	0	0	0	0	0	0	0	0	0	0	0	0	0	0	0	0	0	0	0	0	0	0	0	0	0	0	0	0	0	0	0	1	44.98111667	14.45193333
2193	2008/089	0	0	0	0	0	0	0	0	0	0	0	0	0	0	0	0	0	0	0	0	0	0	0	0	0	0	0	0	0	0	0	0	0	0	0	0	0	0	0	0	0	0	0	0	0	0	0	1	45.21166667	14.55638889
2194	2008/101	1	0	0	0	0	0	0	0	0	0	0	0	0	0	0	0	0	0	0	0	0	0	0	0	0	0	0	0	0	0	0	0	0	0	0	0	0	0	0	0	0	0	0	0	0	0	0	1	43.45548333	16.92051667
2196	2008/103	1	0	0	0	0	1	0	0	0	1	0	0	1	0	1	0	0	0	1	0	0	0	0	0	0	0	0	1	0	0	0	0	0	0	0	0	0	0	0	0	0	1	0	0	0	0	0	1	43.44933333	17.2112
2197	2008/104	0	0	0	0	0	0	0	0	0	0	0	0	0	0	0	0	0	0	0	0	0	0	0	0	0	0	0	0	0	0	0	0	0	0	0	0	0	0	0	0	0	1	0	0	0	0	0	1	43.44995	17.21423333
2198	2008/105	1	0	0	0	0	0	0	0	0	0	0	0	0	0	0	0	0	0	0	0	0	0	0	0	0	0	0	0	0	0	1	0	0	0	0	0	0	0	0	0	0	0	0	0	0	0	0	1	43.06936667	17.41998333
2199	2008/106	0	0	0	0	0	0	0	0	0	0	0	0	0	0	0	0	0	0	0	0	0	0	0	0	0	0	0	0	0	0	1	0	0	0	0	0	0	0	0	0	0	0	0	0	0	0	0	1	42.78776667	17.89316667
2200	2008/107	0	0	0	0	0	0	0	0	0	0	0	0	1	0	0	0	0	0	0	0	0	0	0	0	0	0	0	0	0	0	1	0	0	0	0	0	0	0	0	0	0	0	0	0	0	0	0	1	42.70488333	17.99001667
2201	2008/108	0	0	0	0	0	0	0	0	0	0	0	0	0	0	0	0	0	0	0	0	0	0	0	0	0	0	0	0	0	0	0	0	0	0	0	0	0	0	0	0	0	0	0	0	0	0	0	2	42.64276667	18.1226
2202	2008/109	0	0	0	0	0	0	0	0	0	0	0	0	0	0	0	0	0	0	0	0	0	0	0	0	0	0	0	0	0	0	0	0	0	0	0	0	0	0	0	0	0	0	0	0	0	0	0	1	42.5346	18.37683333
2211	2009/095	0	0	0	0	0	0	0	0	0	0	0	0	0	0	0	0	0	0	0	0	0	0	0	0	0	0	0	0	0	0	0	0	0	0	0	0	0	0	1	0	0	0	0	0	0	0	0	1	44.71026667	16.63946667
2218	2009/102	0	0	0	0	0	0	0	0	0	0	0	0	0	0	0	0	0	0	0	0	0	0	0	0	0	0	0	0	0	0	0	0	0	0	0	0	0	0	1	0	0	0	0	0	0	0	0	1	44.61281667	17.98883333
2223	2009/107	0	0	0	0	0	0	0	0	0	0	0	0	1	0	0	0	0	0	1	0	0	0	0	0	0	0	0	0	0	0	0	0	0	0	0	0	1	0	0	0	0	0	0	0	0	0	0	1	43.83841667	17.59053333
2224	2009/108	0	0	0	0	0	0	0	0	0	0	0	0	0	0	0	0	0	0	0	0	0	0	0	0	0	0	0	0	0	0	0	0	0	0	0	0	1	0	0	0	0	0	0	0	0	0	0	1	43.81568333	17.61398333
2227	2009/111	0	0	0	0	0	0	0	0	0	0	0	0	0	0	0	0	0	0	1	0	0	0	0	0	0	0	0	0	0	0	0	0	0	0	0	0	0	0	0	0	0	0	0	0	0	0	0	1	43.65678333	17.94911667
2228	2009/112	0	0	0	0	0	0	0	0	0	0	0	0	0	0	0	0	0	0	0	0	0	0	0	0	0	0	0	0	0	0	0	0	0	0	0	0	1	0	0	0	0	0	0	0	0	0	0	1	43.67118333	17.98403333
2230	2009/114	0	0	0	0	0	0	0	0	0	0	0	0	1	0	0	0	0	0	0	0	0	0	0	0	0	0	0	0	0	0	0	0	0	0	0	0	0	0	0	0	0	0	0	0	0	0	0	1	43.80691667	18.18841667
2232	2009/116	0	0	0	0	0	0	0	0	0	0	0	0	0	0	0	0	0	0	1	0	0	0	0	0	0	0	0	0	0	0	0	0	0	0	0	0	0	0	0	0	0	0	0	0	0	0	0	3	43.95118333	18.0881
2235	2009/119	0	0	0	0	0	0	0	0	0	0	0	0	0	0	1	0	0	0	1	0	0	0	0	0	0	0	0	0	0	0	0	0	0	0	0	0	0	0	0	0	0	0	0	0	0	0	0	1	43.81911667	18.95545
2236	2009/120	0	0	0	0	0	0	0	0	0	0	0	0	0	0	1	0	0	0	1	0	0	0	0	0	0	0	0	0	0	0	0	0	0	0	0	0	0	0	0	0	0	0	0	0	0	0	0	1	43.78521667	19.01055
2237	2009/121	0	0	0	0	0	0	0	0	0	0	0	0	0	0	1	0	0	0	0	0	0	0	0	0	0	0	0	0	0	0	0	0	0	0	0	0	0	0	1	0	0	0	0	0	0	0	0	1	43.7395	19.00311667
2238	2009/122	0	0	0	0	0	0	0	0	0	0	0	0	0	0	1	0	0	0	0	0	0	0	0	0	0	0	0	0	0	0	0	0	0	0	0	0	0	0	1	0	0	0	0	0	0	0	0	1	43.75431667	19.41788333
2239	2009/130	1	0	0	0	0	0	0	0	1	1	0	0	1	0	0	1	0	1	1	0	1	1	1	0	1	0	0	0	0	1	0	0	0	0	0	0	0	0	0	0	1	0	0	0	0	0	0	1	44.02966667	22.22335
2245	2009/136	0	0	0	0	0	0	0	0	0	0	0	0	0	0	0	0	0	0	0	0	0	0	0	0	0	0	0	0	0	0	0	0	0	0	0	0	0	0	0	0	0	0	0	0	0	0	0	3	43.13558333	23.14086667
2247	2009/138	1	1	0	0	0	1	1	0	1	1	0	0	1	0	1	1	1	0	0	0	0	0	1	1	0	0	0	0	0	1	0	0	0	0	0	0	0	0	0	0	1	0	0	0	0	0	0	1	43.20453333	23.4936
2248	2009/139	1	0	0	0	1	1	0	0	0	0	0	0	1	0	1	0	0	0	1	0	1	1	1	0	1	1	0	0	0	1	0	0	0	0	0	0	0	0	0	0	1	0	0	0	0	0	0	1	43.1907	23.53028333
2250	2009/141	0	0	0	0	0	0	0	0	0	0	0	0	1	0	0	0	0	0	0	0	0	0	0	0	0	0	0	0	0	0	0	0	0	0	0	0	0	0	0	0	1	0	0	0	0	0	0	3	42.9735	24.1594
2251	2009/142	0	0	0	0	0	0	0	0	0	0	0	0	0	0	0	0	0	0	0	0	0	0	0	0	0	0	0	0	0	0	0	0	0	0	0	0	0	0	0	0	0	0	0	0	0	0	0	3	42.97863333	24.16246667
2255	2009/146	0	0	0	0	0	1	0	0	0	0	0	0	0	0	0	0	0	0	0	0	0	0	0	0	0	0	0	0	0	0	0	0	0	0	0	0	0	0	0	0	0	0	0	0	0	0	0	1	43.18118333	24.07088333
2261	2009/152	0	0	0	0	0	0	0	0	0	0	0	0	1	0	0	0	0	0	0	0	0	0	0	0	0	0	0	0	0	0	0	0	0	0	0	0	0	0	0	0	0	0	0	0	0	0	0	1	43.70436667	24.89595
2264	2009/155	0	0	0	0	0	0	0	0	0	0	0	0	0	0	0	0	0	0	1	0	0	0	0	0	0	0	0	0	0	1	0	0	0	0	0	0	0	0	0	0	1	0	0	0	0	0	0	1	43.15883333	22.58093333
2265	2009/156	0	0	0	0	0	1	0	1	1	1	0	0	0	0	0	0	0	0	1	0	1	0	0	0	0	0	0	0	0	0	0	0	0	0	0	0	0	0	0	0	1	0	0	0	0	0	0	1	43.18791667	22.59583333
2267	2009/158	0	0	0	0	0	0	0	0	0	0	0	0	0	0	0	0	0	0	0	0	0	0	0	0	0	0	0	0	0	0	0	0	0	0	0	0	0	0	0	0	0	0	0	0	0	0	0	1	43.32	22.17316667
2276	2011/010	1	0	0	0	0	0	0	0	0	0	0	0	0	0	0	0	0	0	0	0	0	0	0	0	0	0	0	0	0	1	0	0	0	1	0	0	0	0	0	0	0	0	0	0	0	0	0	1	39.95808333	23.92501667
2277	2011/011	0	0	0	0	0	0	0	0	0	0	0	0	1	0	1	0	0	0	0	0	0	0	0	0	0	0	0	0	0	0	0	0	0	1	0	0	0	0	0	0	0	0	0	0	0	0	0	2	40.37558333	23.16671667
2279	2012/213	1	0	0	0	0	0	0	0	0	0	0	0	0	0	0	0	0	0	1	0	0	0	0	0	0	0	0	0	0	0	1	0	0	0	0	0	1	0	0	0	0	0	0	0	0	0	0	1	42.9131	17.4228
2281	2014/002	0	0	0	0	0	0	0	0	0	0	0	0	0	0	0	0	0	0	0	0	0	0	0	0	0	0	0	0	0	0	0	0	0	0	0	0	0	0	0	0	0	0	0	0	0	0	0	2	41.0505	20.6441
2282	2014/003	1	0	0	0	0	1	0	0	0	0	0	0	1	1	1	0	0	0	1	0	0	0	0	0	0	0	0	0	0	0	0	0	0	0	0	0	0	0	0	0	0	0	0	0	1	0	1	2	41.0632	20.6253
2286	2014/007	1	0	0	0	0	1	0	0	0	1	0	0	1	1	1	0	0	0	1	0	0	0	0	0	0	0	0	0	0	0	0	0	0	0	0	0	0	0	0	0	0	0	0	0	0	0	1	1	41.0042	20.4343
2289	2014/010	0	0	0	0	0	0	0	0	0	0	0	0	0	0	0	0	0	0	0	0	0	0	0	0	0	0	0	0	0	0	0	0	0	0	0	0	0	0	0	0	0	0	0	0	0	0	1	1	41.2719	20.2839
2291	2014/012	0	0	0	0	0	0	0	0	0	0	0	0	0	0	0	0	0	0	1	0	0	0	0	0	0	0	0	0	0	0	0	0	0	0	0	0	0	0	0	0	0	0	0	0	1	0	0	2	41.2727	20.2703
2292	2014/013	0	0	0	0	0	1	0	0	0	0	0	0	0	0	0	0	0	0	0	0	0	0	0	0	0	0	0	0	0	0	0	0	0	0	0	0	0	0	0	0	0	0	0	0	1	0	0	1	41.2672	20.261
2293	2014/014	0	0	0	0	0	0	0	0	0	0	0	0	0	0	0	0	0	0	0	0	0	0	0	0	0	0	0	0	0	0	0	0	0	0	0	0	0	0	0	0	0	0	0	0	0	0	0	2	41.2652	20.2422
2294	2014/015	1	0	0	0	0	1	0	0	0	0	0	0	1	0	1	0	0	0	1	0	0	0	1	0	0	0	0	0	0	0	0	0	0	0	0	0	0	0	0	0	0	0	0	0	1	0	1	1	41.2802	20.21
2297	2014/018	1	0	0	0	0	1	0	0	1	1	0	0	1	0	1	0	0	0	0	0	0	0	0	0	0	0	0	0	0	0	0	0	0	0	0	0	0	0	0	0	0	0	0	0	0	0	1	1	41.2776	20.3375
2298	2014/019	1	0	0	0	0	0	0	0	0	0	0	0	0	0	1	0	0	0	1	0	0	0	0	0	0	0	0	0	0	0	0	0	0	0	0	0	0	0	0	0	0	0	0	0	1	0	1	1	41.2966	20.3741
2299	2014/020	0	0	0	0	0	1	0	0	0	0	0	0	0	0	0	0	0	0	1	0	0	0	0	0	0	0	0	0	0	0	0	0	0	0	0	0	0	0	0	0	0	0	0	0	1	0	1	1	41.3611	20.3967
2300	2014/021	0	0	0	0	0	0	0	0	0	0	0	0	0	0	0	0	0	0	0	0	0	0	0	0	0	0	0	0	0	0	0	0	0	0	0	0	0	0	0	0	0	0	0	0	1	0	1	1	41.3462	20.3904
2301	2014/022	0	0	0	0	0	0	0	0	0	0	0	0	0	0	0	0	0	0	1	0	0	0	0	0	0	0	0	0	0	0	0	0	0	0	0	0	0	0	0	0	0	0	0	0	0	0	1	1	41.3339	20.3958
2302	2014/023	1	0	0	0	0	0	0	0	0	1	0	0	0	1	0	0	0	0	1	0	0	0	1	0	0	0	0	0	0	0	0	0	0	0	0	0	0	0	0	0	0	0	0	1	1	0	0	3	41.3238	20.4224
2303	2014/024	0	0	0	0	0	1	0	0	1	0	0	0	0	0	1	0	0	0	1	0	0	0	0	0	0	0	0	0	0	0	0	0	0	0	0	0	0	0	0	0	0	0	0	0	0	0	1	1	41.325	20.4457
2304	2014/025	0	0	0	0	0	1	0	0	1	0	0	0	0	0	0	1	0	0	1	0	0	0	0	0	0	0	0	0	0	0	0	0	0	0	0	0	0	0	0	0	0	0	0	0	1	0	1	1	41.3646	20.4685
2305	2014/026	0	0	0	0	0	0	0	0	0	0	0	0	0	0	0	0	0	0	1	0	0	0	0	0	0	0	0	0	0	0	0	0	0	0	0	0	0	0	0	0	0	0	0	0	0	0	1	1	41.3792	20.4687
2307	2014/028	0	0	0	0	0	0	0	0	0	0	0	0	1	0	0	0	0	0	0	0	0	0	0	0	0	0	0	0	0	0	0	0	0	0	0	0	0	0	0	0	0	0	0	0	0	0	1	1	41.4199	20.4633
2308	2014/029	1	0	0	0	0	1	0	0	1	0	0	0	1	1	1	0	0	0	0	0	0	0	0	0	0	0	0	0	0	0	0	0	0	0	0	0	0	0	0	0	0	0	0	1	1	1	1	1	41.4404	20.5052
2312	2014/033	1	0	0	0	1	1	0	0	1	0	0	0	1	0	0	0	0	0	0	0	0	0	0	0	0	0	0	1	0	1	0	0	0	0	0	0	0	0	0	0	0	0	0	0	1	0	1	1	41.6097	20.4431
2314	2014/035	1	0	0	0	0	1	0	0	1	0	0	0	1	1	0	1	0	0	1	0	0	0	1	0	0	0	0	1	1	0	0	0	0	0	0	0	0	0	0	0	0	0	0	0	1	0	1	1	41.6308	20.2983
2315	2014/036	1	0	0	0	0	1	0	0	1	0	0	0	1	0	1	0	0	0	1	0	0	0	0	0	0	1	0	1	1	1	0	0	0	0	0	0	0	0	0	0	0	0	0	0	1	0	1	1	41.628	20.2741
2316	2014/037	0	0	0	0	0	0	0	0	0	0	0	0	0	0	0	0	0	0	0	0	0	0	0	0	0	0	0	0	0	0	0	0	0	0	0	0	0	0	0	0	0	0	0	0	0	0	1	1	41.6228	20.2886
2317	2014/038	0	0	0	0	0	0	0	0	0	0	0	0	0	0	0	0	0	0	0	0	0	0	0	0	0	0	0	0	0	0	0	0	0	0	0	0	0	0	0	0	0	0	0	1	0	0	0	1	41.6541	20.3129

2321	2014/042	0	0	0	0	0	0	0	0	0	0	0	0	0	1	1	0	0	0	0	0	0	0	0	0	0	0	0	0	0	0	0	0	0	0	0	0	0	0	0	0	0	0	0	0	0	0	0	2	1	41.514	20.316
2324	2014/045	1	0	0	0	0	1	0	0	0	1	0	0	1	1	1	0	0	0	0	0	0	0	0	0	0	0	0	1	1	0	0	0	0	0	0	0	0	0	0	0	0	0	0	0	0	0	0	2	1	41.4677	20.1048
2325	2014/046	0	0	0	0	0	0	0	0	0	0	0	0	1	1	1	0	0	0	1	0	0	0	0	0	0	0	0	1	0	0	0	0	0	0	0	0	0	0	0	0	0	0	0	0	0	0	0	1	1	41.4685	20.0904
2326	2014/047	1	0	0	0	0	0	0	0	0	0	0	0	0	0	0	1	0	0	1	0	1	0	0	0	0	0	0	0	0	0	0	0	0	0	0	0	0	0	0	0	0	0	0	0	1	0	1	3	41.4361	20.0485
2329	2014/050	1	0	1	0	0	1	0	0	0	0	0	0	1	0	1	0	0	0	1	0	0	0	1	0	0	0	0	1	0	0	1	0	0	0	0	0	1	0	0	0	0	0	0	1	0	0	1	1	41.6919	19.8318
2330	2014/051	1	0	0	0	0	1	0	0	0	0	0	0	1	1	1	0	0	0	1	0	0	0	0	0	0	0	0	1	0	1	0	0	0	0	0	0	1	0	0	0	0	0	0	0	0	0	0	1	41.7002	19.7052
2331	2014/052	0	0	0	0	0	0	0	0	0	0	0	0	0	0	0	0	0	0	0	0	0	0	0	0	0	0	0	0	0	0	0	0	0	0	0	0	1	0	0	0	0	0	0	0	1	0	0	3	41.6388	19.7286
2332	2014/053	0	0	0	0	0	0	0	0	0	0	0	0	0	0	0	0	0	0	1	0	0	0	0	0	0	0	0	0	0	0	1	0	0	0	0	0	1	0	0	0	0	0	0	0	0	0	0	1	41.6296	19.733
2334	2014/055	1	0	0	0	0	0	0	0	0	0	0	0	0	0	0	0	0	0	0	0	0	0	0	0	0	0	0	0	0	0	0	0	0	0	0	0	0	0	0	0	0	0	0	0	0	0	1	1	41.4412	19.8636
2335	2014/056	1	0	0	0	0	0	0	0	0	0	0	0	1	0	0	0	0	0	0	0	0	0	0	0	0	0	0	0	0	0	0	0	0	0	0	0	0	0	0	0	0	0	0	0	0	0	1	1	41.3948	19.9
2336	2014/057	1	0	0	0	0	1	0	0	1	1	0	0	1	1	1	0	0	0	1	0	0	0	1	0	0	0	0	1	1	0	1	0	0	0	0	0	0	0	0	0	0	0	0	0	1	0	1	1	41.2598	19.9676
2338	2014/058	1	0	0	0	0	0	0	0	0	1	0	0	0	0	0	0	0	0	0	0	0	0	0	0	0	0	0	0	0	1	0	0	0	0	0	0	0	0	0	0	0	0	0	0	1	0	0	1	41.1005	20.1249
2343	2014/063	1	0	0	0	0	0	0	0	1	1	0	0	1	0	1	0	0	1	1	0	0	0	0	0	1	1	0	0	0	1	0	0	0	0	0	1	0	0	1	0	0	0	0	0	0	0	0	1	43.85131809	19.82230472
2346	2014/066	0	0	0	0	0	0	0	0	0	0	0	0	1	0	0	0	0	0	0	0	0	0	0	0	0	0	0	0	0	0	0	0	0	0	0	0	0	0	0	0	0	0	0	0	0	0	0	1	42.39132207	19.73029716
2348	2014/068	1	0	0	0	0	1	0	0	1	1	0	0	1	0	0	0	0	0	1	0	0	0	0	0	0	0	0	1	1	0	1	0	0	0	0	0	0	0	0	0	0	0	0	0	0	0	0	1	42.13060297	19.71060824
2349	2014/069	1	0	0	0	0	1	0	0	0	0	0	0	1	0	0	0	0	0	1	0	1	0	0	0	0	0	0	0	1	0	1	0	0	0	0	0	0	0	0	0	0	0	0	0	0	0	0	1	42.12730469	19.71185186
2350	2014/070	0	0	0	0	0	1	0	0	0	0	0	0	1	0	0	0	0	0	0	0	0	0	0	0	0	0	0	1	0	0	0	0	0	0	0	0	0	0	0	0	0	0	0	0	0	0	0	1	41.85994897	20.118936
2351	2014/071	1	0	0	0	0	1	0	0	0	0	0	0	1	1	0	0	0	0	0	0	0	0	0	0	0	0	0	0	0	0	0	0	0	0	0	0	0	0	0	0	0	0	0	0	0	0	0	1	41.85607913	20.12075471
2352	2014/072	0	0	0	0	0	0	0	0	1	0	0	0	1	0	0	0	0	0	0	0	0	0	0	0	0	0	0	0	0	0	0	0	0	0	0	0	0	0	0	0	0	0	0	0	0	0	0	3	41.84553872	20.12990389
2353	2014/073	1	0	0	0	0	1	0	0	0	0	0	0	1	1	0	0	0	0	1	0	0	0	0	0	0	0	0	1	1	0	0	0	0	0	0	0	0	0	0	0	0	0	0	0	0	0	0	3	41.8524242	20.12344865
2354	2014/073B	0	0	0	0	0	0	0	0	0	0	0	0	0	0	0	0	0	0	0	0	0	0	0	0	0	0	0	0	0	0	0	0	0	0	0	0	0	0	0	0	0	0	0	0	0	0	0	1	41.85440015	20.12372442
2355	2014/074	0	0	0	0	0	0	0	0	0	0	0	0	0	0	0	0	0	0	0	0	0	0	0	0	0	0	0	0	0	0	0	0	0	0	0	0	0	0	0	0	0	0	0	0	0	0	0	1	41.84756345	20.11680348
2356	2014/075	0	0	0	0	0	0	0	0	0	0	0	0	0	0	0	0	0	0	0	0	0	0	0	0	0	0	0	0	0	0	0	0	0	0	0	0	0	0	0	0	0	0	0	0	0	0	0	1	41.84325482	20.11186
2357	2014/076	1	0	0	0	0	0	0	0	0	0	0	0	0	0	0	0	0	0	1	0	0	0	0	0	0	0	0	0	0	0	0	0	0	0	0	0	0	0	0	0	0	0	0	0	1	0	0	1	41.85947715	20.14933557
2358	2014/077	0	0	0	0	0	1	0	0	0	0	0	0	0	0	1	0	0	0	0	0	1	0	0	0	0	0	0	0	0	0	0	0	0	0	0	0	0	0	0	0	0	0	0	0	0	0	0	1	41.87470883	20.16569914
2359	2014/078	1	0	0	0	0	1	0	0	0	0	0	0	0	0	1	0	0	0	1	0	1	0	0	0	0	0	0	0	0	0	0	0	0	0	0	0	0	0	0	0	0	0	0	0	1	0	0	1	41.87930086	20.16633684
2360	2014/079	1	0	0	0	0	1	0	0	0	0	0	0	1	0	0	0	0	0	1	0	1	0	0	0	0	0	0	1	1	0	0	0	0	0	0	0	0	0	0	0	0	0	0	0	1	0	0	1	41.88691397	20.17148634
2361	2014/080	0	0	0	0	0	0	0	0	0	0	0	0	1	1	0	0	0	0	0	0	0	0	0	0	0	0	0	0	0	0	0	0	0	0	0	0	0	0	0	0	0	0	0	0	1	0	0	1	41.89182946	20.1873734
2362	2014/081	1	0	0	0	0	1	0	0	1	1	0	0	1	1	0	0	0	0	1	0	1	0	0	0	0	0	0	0	0	0	0	0	0	0	0	0	0	0	0	0	0	0	0	0	1	0	0	3	41.89971976	20.19479549
2363	2014/082	0	0	0	0	0	0	0	0	1	0	0	0	1	0	0	0	0	0	1	0	0	0	0	0	0	0	0	0	0	0	0	0	0	0	0	0	0	0	0	0	0	0	0	0	0	0	0	1	41.90106925	20.19663281
2364	2014/083	0	0	0	0	0	0	0	0	0	0	0	0	0	0	0	0	0	0	0	0	0	0	0	0	0	0	0	0	0	0	0	0	0	0	0	0	0	0	0	0	0	0	0	0	0	0	0	3	41.89704459	20.19823501
2365	2014/083B	0	0	0	0	0	0	0	0	0	0	0	0	0	0	0	0	0	0	0	0	0	0	0	0	0	0	0	0	0	0	0	0	0	0	0	0	0	0	0	0	0	0	0	0	1	0	0	3	41.89556535	20.1974347
2367	2014/085	0	0	0	0	0	0	0	0	0	0	0	0	0	0	0	0	0	0	1	0	0	0	0	0	0	0	0	0	0	0	0	0	0	0	0	0	0	0	0	0	0	0	0	0	0	0	0	1	41.88615172	20.18682674
2368	2014/086	0	0	0	0	0	0	0	0	0	0	0	0	0	0	0	0	0	0	0	0	0	0	0	0	0	0	0	0	0	0	0	0	0	0	0	0	0	0	0	0	0	0	0	0	0	0	0	1	41.88390059	20.1724915
2369	2014/087	0	0	0	0	0	0	0	0	0	0	0	0	0	0	0	0	0	0	1	0	0	0	0	0	0	0	0	0	0	0	0	0	0	0	0	0	0	0	0	0	0	0	0	0	1	0	0	1	41.9201	20.1989
2370	2014/089	0	0	0	0	0	0	0	0	0	0	0	0	0	0	0	0	0	0	1	0	0	0	0	0	0	0	0	0	0	0	0	0	0	0	0	0	0	0	0	0	0	0	0	0	0	0	0	1	41.92943822	20.18502354
2371	2014/090	0	0	0	0	0	1	0	0	0	0	0	0	0	1	0	0	0	0	0	0	0	0	0	0	0	0	0	0	1	0	0	0	0	0	0	0	1	0	0	0	0	0	0	0	0	1	0	3	41.88749274	20.17427199
2372	2014/091	1	0	0	0	0	1	0	0	0	0	0	0	1	1	0	0	0	0	1	0	0	0	0	0	0	0	0	0	0	1	0	0	0	0	0	0	0	0	0	1	0	0	0	0	0	0	0	3	42.55327636	20.24923646
2373	2014/092	1	0	0	0	0	1	0	0	0	0	0	0	1	0	0	0	0	0	1	0	0	0	0	0	0	0	0	0	0	0	0	0	0	0	0	0	0	1	0	0	0	0	0	0	0	0	0	1	42.43131464	20.14990035
2374	2014/093	0	0	0	0	0	0	0	0	0	0	0	0	0	0	0	0	0	0	0	0	0	0	0	0	0	0	0	0	0	0	0	0	0	0	0	0	0	1	0	0	0	0	0	0	0	0	0	3	42.45462262	20.15778135
2375	2014/094	1	0	0	0	0	1	0	0	0	0	0	0	1	0	0	0	0	0	1	0	0	0	0	0	0	0	0	0	0	1	0	0	0	0	0	0	1	0	0	1	0	0	0	0	0	0	1	3	42.4692	20.1554
2376	2014/095	1	0	0	0	0	0	0	0	0	0	0	0	1	0	0	0	0	0	1	0	1	0	1	0	0	0	0	0	0	1	0	0	0	0	0	0	0	0	0	1	0	0	0	0	0	0	1	3	42.474	20.152
2378	2014/097	1	0	0	0	0	0	0	0	1	0	0	0	1	1	1	0	0	0	1	0	0	0	1	0	0	0	0	1	0	0	0	0	0	0	0	0	0	0	0	0	0	0	1	1	1	0	1	1	40.70414077	19.94787167
2381	2014/100	1	0	0	0	0	0	0	0	0	0	0	0	1	0	0	0	0	0	0	0	0	0	0	0	0	0	0	0	0	0	0	1	0	0	0	0	0	0	0	0	0	0	0	1	0	0	0	1	40.11851538	20.0142897
2382	2014/101	1	0	0	0	0	0	0	0	0	0	0	0	0	0	0	0	0	0	0	0	0	0	0	0	0	0	0	0	0	0	0	1	0	0	0	0	0	0	0	0	0	0	0	1	0	0	0	1	40.11658821	20.00020718
2383	2014/102	1	0	0	0	0	0	0	0	0	0	0	0	0	0	1	0	0	0	0	0	0	0	1	0	0	0	0	0	0	0	0	0	0	0	0	0	0	0	0	0	0	0	0	1	0	0	0	1	40.0949	19.991734
2384	2014/103	1	0	0	0	0	1	0	0	1	0	0	0	1	0	1	1	0	0	0	0	0	0	0	0	0	0	0	0	0	0	0	1	0	0	0	0	0	0	0	0	0	0	0	1	1	0	0	1	40.08816979	19.96313788
2385	2014/104	1	0	0	0	0	1	0	0	0	0	0	0	1	0	1	0	0	0	1	0	0	0	1	0	0	0	0	0	1	0	0	0	0	0	0	0	0	0	0	0	0	0	0	0	0	0	1	1	40.27575167	20.09470141
2386	2014/105	0	0	0	0	0	0	0	0	1	1	0	0	1	0	0	0	0	0	1	0	0	0	0	0	0	0	0	0	0	0	0	1	0	0	0	0	0	0	0	0	0	0	0	0	0	0	0	1	40.258292	20.07898903
2388	2014/107	1	0	0	0	0	0	0	0	0	0	0	0	0	0	1	0	0	0	1	0	0	0	1	0	0	0	0	1	0	0	0	1	0	0	0	0	0	0	0	0	0	0	0	0	1	0	1	1	40.29608474	20.10760124
2389	2014/108	1	0	0	0	0	0	0	0	1	0	0	0	1	0	1	0	0	0	1	0	0	0	1	0	0	0	0	1	0	0	0	0	0	0	0	0	0	0	0	0	0	0	0	0	1	0	1	3	40.12257448	20.48048562
2391	2014/110	0	0	0	0	0	0	0	0	0	0	0	0	0	0	0	0	0	0	0	0	0	0	0	0	0	0	0	0	0	0	0	0	0	0	0	0	0	0	0	0	0	0	0	0	0	0	1	1	40.24180354	20.64135958
2394	2014/113	1	0	0	0	0	1	0	0	1	0	0	0	1	0	0	0	0	0	1	0	0	0	1	0	0	0	0	0	0	0	1	0	0	0	0	0	0	0	0	0	0	0	0	0	1	0	0	1	40.73013313	20.50389863
2395	2014/114	1	0	0	0	0	0	0	0	0	0	0	0	1	0	0	1	0	0	0	0	0	0	0	0	0	0	0	0	1	0	1	0	0	0	0	0	0	0	0	0	0	0	0	0	1	0	2	3	40.74798541	20.52191997
2396	2014/115	1	0	0	0	0	1	0	0	0	0	0	0	0	0	0	0	0	0	1	0	0	0	1	0	0	0	0	0	0	0	1	0	0	0	0	0	0	0	0	0	0	0	0	0	0	0	1	1	40.74616964	20.51225656
2398	2014/117	1	0	0	0	0	0	0	0	0	0	0	0	1	0	1	0	0	0	1	0	0	0	0	0	1	0	0	0	0	0	1	0	0	0	0	0	0	0	0	0	0	1	0	0	0	0	0	1	41.00030777	20.24742781
2400	2014/119	0	0	0	0	0	1	0	0	1	0	0	0	0	0	0	1	0	0	0	0	0	0	0	0	0	0	0	0	0	1	0	0	0	0	0	0	0	0	0	0	0	0	0	0	0	0	1	1	40.96267	20.81707
2402	2014/121	0	0	1	0	0	1	0	0	1	0	0	0	0	0	0	1	0	0	1	0	0	0	0	0	0	1	0	0	0	1	0	0	0	0	0	0	0	0	0	0	0	0	0	0	0	0	1	1	40.914	20.7406
2403	2014/122	0	0	0	0	0	0	0	0	0	0	0	0	0	0	0	0	0	0	0	0	0	0	0	0	0	0	0	0	0	0	0	0	0	0	0	0	0	0	0	0	0	0	0	0	0	0	1	1	41.1147	20.7876
2404	BPP2014/5	0	0	0	0	0	0	0	0	0	0	0	0	0	0	0	0	0	0	0	0	0	0	0	0	0	0	0	1	0	0	0	0	0	0	0	0	0	1	0	0	0	0	0	0	0	0	1	2	41.85691	19.50439
2405	BPP2014/14A	0	0	0	0	0	0	0	0	0	0	0	0	0	0	0	0	0	0	0	0	0	0	0	0	0	0	0	1	0	0	0	0	0	0	0	0	0	0	0	0	0	0	0	0	0	0	0	2	40.42874	19.33526
2406	BPP2014/19	1	0	0	0	0	0	0	0	0	0	0	0	0	0	0	0	0	0	0	0	0	0	0	0	0	0	0	0	0	0	0	0	0	0	0	0	0	0	0	0	0	0	0	0	0	0	0	2	39.7175	20.01643
2407	BPP2014/23D	1	0	0	0	0	0	0	0	0	0	0	0	0	0	0	0	0	0	0	0	0	0	0	0	0	0	0	0	0	0	0	1	0	0	0	0	0	0	0	0	0	0	0	1	0	0	0	2	39.90273	20.17079
2408	BPP2014/25	1	0	0	0	0	0	0	0	0	0	0	0	0	0	0	0	0	0	1	0	0	0	0	0	0	0	0	0	0	0	0	0	0	0	0	0	0	0	0	0	0	0	0	0	0	0	0	2	40.24988	20.11788
2409	BPP2014/25B	1	0	0	0	0	0	0	0	0	0	0	0	0	0	0	0	0	0	0	0	0	0	0	0	0	0	0	0	0	0	0	1	0	0	0	0	0	0	0	0	0	0	0	0	0	0	0	1	40.24512	20.11061
2410	BPP2014/26	0	0	0	0	0	0	0	0	0	0	0	0	0	0	0	0	0	0	0	0	0	0	0	0	0	0	0	0	0	0	0	0	0	0	0	0	0	0	0	0	0	0	0	0	1	0	1	1	40.49264	19.72609
2412	2014/123	0	0	0	0	0	0	0	0	0	0	0	0	0	0	1	0	0	0	0	0	0	0	0	0	0	0	0	0	0	1	0	0	0	0	0	1	0	0	0	0	0	0	0	0	0	0	0	3	41.65089098	20.71347621
2413	2014/124	0	0	0	0	0	1	0	0	1	1	0	0	0	0	1	1	0	0	1	0	0	0	0	0	0	0	0	0	0	0	0	0	0	0	0	0	0	0	0	0	0	0	0	0	0	0	1	3	41.5942097	20.67159771
2414	2014/125	0	0	0	0	0	1	0	0	1	0	0	0	1	0	1	1	0	0	0	0	0	0	0	0	0	0	0	0	0	0	0	0	0	0	0	0	0	0	0	0	0	0	0	0	0	1	1	1	41.59357175	20.65752852
2415	2014/126	0	0	0	0	0	1	0	0	0	1	0	0	0	0	1	1	0	0	0	0	0	0	0	0	0	0	0	0	0	1	0	0	0	0	0	0	0	0	0	0	0	0	0	0	0	0	1	1	41.62395129	20.67683933
2416	2014/127	0	0	0	0	0	0	0	0	1	0	0	0	0	0	0	1	0	0	1	0	0	0	0	0	0	0	0	0	0	1	0	0	0	0	0	0	0	0	0	0	0	0	0	0	0	0	0	1	41.64226826	20.69765336
2417	2014/128	0	0	0	0	0	0	0	0	1	1	0	0	1	0	0	0	0	0	0	0	1	0	0	0	0	0	0	0	0	1	0	0	0	0	0	0	0	0	0	0	0	0	0	0	0	0	0	1	41.64685031	20.70980569
2418	2014/129	0	0	0	0	0	1	0	0	1	0	0	0	1	0	1	0	0	0	1	0	0	0	1	0	0	1	0	0	0	1	0	0	0	0	0	1	0	0	0	0	0	0	0	0	0	1	0	1	41.71171619	20.71232286
2419	2014/130	0	0	0	0	0	1	0	0	1	0	0	0	1	0	0	0	0	0	1	0	0	0	0	0	0	0	0	0	0	1	0	0	0	0	0	0	0	0	0	0	0	0	0	0	0	0	1	1	41.72026657	20.66822106
2420	2014/131	0	0	0	0	0	0	0	0	0	0	0	0	0	0	0	0	0	0	1	0	0	0	0	0	0	0	0	0	0	0	0	0	0	0	0	1	0	0	0	0	0	0	0	0	0	0	1	1	41.76549599	20.66659589
2421	2014/132	0	0	0	0	0	1	0	0	1	0	0	0	0	0	1	0	0	0	0	0	0	0	0	0	0	0	0	0	0	0	0	0	0	0	0	1	0	0	0	0	0	0	0	0	0	0	1	1	41.7102	20.6564
2422	2014/133	0	0	0	0	0	1	0	0	1	0	0	0	1	0	1	0	0	0	1	0	0	0	0	0	0	0	0	0	0	1	0	0	0	0	0	1	0	0	0	0	0	0	0	0	0	1	1	1	41.70793352	20.6500164
2423	2014/134	0	0	0	0	0	0	0	0	1	0	0	0	1	0	0	0	0	0	0	0	0	0	0	0	0	0	0	0	0	1	0	0	0	0	0	0	0	0	0	0	0	0	0	0	0	0	1	1	41.69940996	20.64784809
2424	2014/135	0	0	0	0	0	0	0	0	0	0	0	0	0	0	0	0	0	0	0	0	0	0	0	0	0	0	0	0	0	0	0	0	0	0	0	0	0	0	0	0	0	0	0	0	0	0	1	2	41.49481357	20.50522431
2425	2014/136	0	0	0	0	0	0	0	0	0	0	0	0	0	0	0	0	0	0	0	0	0	0	0	0	0	0	0	0	0	0	0	0	0	0	0	0	0	0	0	0	0	0	0	0	1	0	0	1	41.44393735	20.55075321
2426	2014/137	0	0	0	0	0	0	0	0	0	0	0	0	0	0	0	0	0	0	0	0	0	0	0	0	0	0	0	0	0	0	0	0	0	0	0	0	0	0	0	0	0	0	0	0	0	0	0	1	41.2997583	20.6200454
2427	2014/138	0	0	0	0	0	1	0	0	1	1	0	0	1	0	1	0	0	0	0	0	0	0	0	0	0	0	0	0	0	0	0	0	0	0	0	0	0	0	0	0	0	0	0	1	1	0	1	1	41.32985277	20.63693494
2428	2014/139	1	0	0	0	0	1	0	0	1	0	0	0	1	1	0	0	0	0	1	0	0	0	0	0	0	0	0	0	0	1	0	0	0	0	0	0	0	0	0	0	0	0	0	0	1	0	1	1	41.3397	20.6273
2430	2014/141	1	0	0	0	0	1	0	0	1	1	0	0	1	1	1	0	0	0	1	0	0	0	0	0	0	0	0	0	0	1	0	0	0	0	0	0	0	0	0	0	0	0	0	0	1	0	1	1	41.35451392	20.61244343
2431	2014/142	0	0	0	0	0	1	0	0	0	0	0	0	1	0	0	0	0	0	0	0	0	0	0	0	0	0	0	0	0	0	0	0	0	0	0	0	0	0	0	0	0	0	0	1	1	0	1	1	41.36230314	20.60361788
2432	2014/143	1	0	0	0	0	1	0	0	0	0	0	0	0	0	0	0	0	0	1	0	0	0	0	0	0	0	0	0	0	0	0	0	0	0	0	0	0	0	0	0	0	0	0	0	0	0	1	1	41.36378967	20.60489343
2433	2014/144	1	0	0	0	0	1	0	0	1	1	0	0	1	0	1	0	0	0	0	0	0	0	0	0	0	0	0	0	0	0	0	0	0	0	0	0	0	0	0	0	0	0	0	0	0	0	1	1	41.36664086	20.60492738
2434	2014/145	0	0	0	0	0	0	0	0	0	0	0	0	0	0	0	0	0	0	0	0	0	0	0	0	0	0	0	0	0	0	0	0	0	0	0	0	0	0	0	0	0	0	0	0	0	0	1	1	41.36720781	20.60463049
2435	2014/146	1	0	0	0	0	1	0	0	1	1	0	0	1	0	1	1	0	0	1	0	0	0	0	0	0	1	0	0	0	1	0	0	0	0	0	0	0	0	0	0	0	0	0	0	0	0	1	1	41.36945827	20.60207795
2436	2014/147	0	0	0	0	0	0	0	0	0	0	0	0	1	0	0	0	0	0	0	0	0	0	0	0	0	0	0	0	0	1	0	0	0	0	0	0	0	0	0	0	0	0	0	0	0	0	1	1	41.37047214	20.59794609
2437	2014/148	0	0	0	0	0	0	0	0	1	0	0	0	0	0	0	0	0	0	0	0	0	0	0	0	0	0	0	0	0	0	0	0	0	0	0	0	0	0	0	0	0	0	0	0	0	0	1	1	41.37134554	20.59709994
2438	2014/149	0	0	0	0	0	0	0	0	0	0	0	0	0	0	0	0	0	0	0	0	0	0	0	0	0	0	0	0	0	0	0	0	0	0	0	0	0	0	0	0	0	0	0	0	0	0	0	1	41.40042353	20.58493369
2439	2014/150	1	0	0	0	0	1	0	0	0	0	0	0	0	0	0	0	0	0	0	0	0	0	0	0	0	0	0	0	0	0	0	0	0	0	0	0	0	0	0	0	0	0	0	0	0	0	1	1	41.39784526	20.58994733
2440	2014/151	0	0	0	0	0	0	0	0	0	0	0	0	1	0	1	0	0	0	0	0	0	0	0	0	0	0	0	0	0	0	0	0	0	0	0	0	0	0	0	0	0	0	0	0	0	0	1	1	41.38158244	20.58807707
2441	2014/152	0	0	0	0	0	0	0	0	1	1	0	0	1	1	0	1	0	0	1	0	0	0	1	0	1	0	0	0	0	1	0	0	0	0	0	0	0	0	0	0	0	0	0	0	0	0	1	1	41.22019627	20.91150999
2443	2014/154	0	0	0	0	0	0	0	0	0	0	0	0	0	0	0	0	0	0	0	0	0	0	0	0	0	0	0	0	0	1	0	0	0	0	0	0	0	0	0	0	0	0	0	0	1	0	1	1	41.084319	20.79733053
2444	2014/155	0	0	0	0	0	1	0	0	0	0	0	0	0	0	1	1	0	0	1	0	0	0	0	0	0	0	0	0	0	0	0	0	0	0	0	0	0	0	0	0	0	0	0	0	0	0	1	2	40.99335699	20.85693542
2445	2014/156	0	0	0	0	0	1	0	0	1	0	0	0	0	1	1	1	0	0	0	0	0	0	0	0	0	0	0	0	0	0	0	0	0	0	0	0	0	0	0	0	0	0	0	0	0	0	1	2	41.0026045	20.85337671
2447	2014/158	0	0	0	0	0	1	0	0	0	0	0	0	0	0	1	0	0	0	0	0	0	0	0	0	0	0	0	0	0	0	0	0	0	0	0	0	0	0	0	0	0	0	0	0	0	0	1	2	41.00331821	20.84876633
2448	2014/159	1	0	0	0	0	1	0	0	1	0	0	0	0	0	1	1	0	0	0	0	1	0	0	0	0	0	0	0	0	0	0	0	0	0	0	0	0	0	0	0	0	0	0	0	0	0	1	2	41.00371292	20.84695542
2449	2014/160	1	0	0	0	0	0	0	0	0	0	0	0	1	0	0	0	0	0	0	0	1	0	0	0	0	0	0	0	0	1	0	0	0	0	0	0	0	0	0	0	0	0	0	0	0	0	1	2	41.00456486	20.84318834
2450	2014/161	1	0	0	0	0	1	0	0	0	0	0	0	0	0	1	0	0	0	0	0	1	0	0	0	0	0	0	0	0	0	0	0	0	0	0	0	0	0	0	0	0	0	0	0	0	0	1	2	41.00506509	20.84616811
2451	2014/162	1	0	0	0	0	1	0	0	1	0	0	0	1	1	1	0	0	0	1	0	0	0	0	0	0	0	0	0	0	1	0	0	0	0	0	0	0	0	0	0	0	0	0	0	1	0	1	1	40.95738078	20.77780623
2452	2014/163	1	0	0	0	0	1	0	0	0	0	0	0	0	1	0	0	0	0	1	0	0	0	0	0	0	0	0	0	0	1	0	0	0	0	0	0	0	0	0	0	0	0	0	0	1	0	2	2	40.9937315	20.79917514
2453	2014/164	1	0	0	0	0	1	0	0	1	0	0	0	0	1	1	0	0	0	0	0	0	0	0	0	0	0	0	0	0	0	0	0	0	0	0	0	0	0	0	0	0	0	0	0	1	0	1	1	41.00917499	20.80557724
2454	2014/165	1	0	0	0	0	1	0	0	0	0	0	0	1	1	1	0	0	0	1	0	0	0	0	0	0	0	0	0	0	0	0	0	0	0	0	0	0	0	0	0	0	0	0	0	1	0	1	1	41.03454868	20.81052901
2455	2014/166	0	0	0	0	0	1	0	0	0	0	0	0	0	0	1	1	0	0	1	0	0	0	0	0	0	0	0	0	0	1	0	0	0	0	0	0	0	0	0	0	0	0	0	0	1	1	1	1	40.96742992	20.81490646
2456	2014/167	0	0	0	0	0	1	0	0	0	0	0	0	1	1	1	0	0	0	0	0	0	0	0	0	0	0	0	0	0	1	0	0	0	0	0	0	0	0	0	0	0	0	0	0	0	0	1	1	40.89242723	20.9393851
2457	2014/168	0	0	0	0	0	0	0	0	0	0	0	0	1	0	0	0	0	0	0	0	0	0	0	0	0	0	0	0	0	0	0	0	0	0	0	0	0	0	0	0	0	0	0	0	0	0	1	1	40.85800904	20.94259998
2458	2014/169	0	0	0	0	0	0	0	0	0	0	0	0	0	1	0	0	0	0	0	0	0	0	0	0	0	0	0	0	0	0	0	0	0	0	0	0	0	0	0	0	0	0	0	0	1	0	0	2	40.81220319	20.90447774
2459	2014/170	0	0	0	0	0	0	0	0	0	0	0	0	1	0	1	1	0	0	1	0	0	0	1	0	0	0	0	0	0	0	0	0	0	0	0	0	0	0	0	0	0	0	0	0	0	0	1	1	40.6344356	21.09154883
2460	2014/171	1	0	0	0	0	1	0	0	0	0	0	0	1	1	1	0	0	0	1	0	0	0	0	0	0	0	0	0	0	1	0	0	0	0	0	0	0	0	0	0	0	0	0	0	0	0	1	1	40.52332627	21.27467615
2462	2014/173	0	0	0	0	0	0	0	0	0	0	0	0	0	0	0	0	0	0	0	0	0	0	0	0	0	0	0	0	0	0	0	0	0	0	0	0	0	0	0	0	0	0	0	0	0	0	1	1	40.80872211	20.99792314
2463	2014/174	0	0	0	0	0	1	0	0	0	0	0	0	0	1	1	0	0	0	0	0	0	0	0	0	0	0	0	0	0	0	0	0	0	0	0	0	0	0	0	0	0	0	0	0	1	0	0	1	40.81996283	21.05221474
2464	2014/175	0	0	0	0	0	1	0	0	1	0	0	0	1	0	1	0	0	0	0	0	0	0	0	0	0	0	0	0	0	0	0	0	0	0	0	0	0	0	0	0	0	0	0	0	0	0	1	1	40.79977711	21.07147157
2465	2014/176	0	0	0	0	0	1	0	0	1	0	0	0	1	1	0	0	0	0	1	0	0	0	0	0	0	0	0	0	1	0	0	0	0	0	0	0	0	0	0	0	0	0	0	0	1	0	1	1	40.71921872	21.03408761
2466	2014/177	0	0	0	0	0	1	0	0	1	0	0	0	1	1	1	0	0	0	0	0	0	0	0	0	0	0	0	0	0	1	0	0	0	0	0	0	0	0	0	0	0	0	0	0	0	0	1	1	40.81053067	21.07019073
2467	2014/178	0	0	0	0	0	1	0	0	1	0	0	0	0	1	1	0	0	0	0	0	0	0	0	0	0	0	0	0	0	0	0	0	0	0	0	0	0	0	0	0	0	0	0	0	0	0	1	1	40.65323889	21.17894132
2468	2014/179	0	0	0	0	0	1	0	0	0	0	0	0	0	0	1	0	0	0	0	0	0	0	0	0	0	0	0	0	0	0	0	0	0	0	0	0	0	0	0	0	0	0	0	0	0	0	0	2	41.37982568	21.74413774
2473	2015/004	1	0	0	0	0	0	0	0	0	0	0	0	1	0	1	0	0	0	0	0	0	0	0	0	0	0	0	0	1	0	0	0	0	0	0	0	0	1	0	0	0	0	0	0	0	0	0	1	42.643544	19.377819
2474	2015/005	1	0	0	0	0	0	0	0	0	0	0	0	0	0	0	0	0	0	0	0	0	0	0	0	0	0	0	0	1	0	0	0	0	0	0	0	0	0	0	0	0	0	0	0	0	0	0	1	42.62853	19.372128
2475	2015/006	1	0	0	0	0	0	0	0	0	0	0	0	0	0	1	0	0	0	0	0	0	0	0	0	0	0	0	0	1	0	0	0	0	0	0	0	0	1	0	0	0	0	0	0	0	0	1	1	42.37066	19.313889
2476	2015/007	0	0	0	0	0	0	0	0	0	0	0	0	1	0	0	0	0	0	0	0	0	0	0	0	0	0	0	0	1	0	0	0	0	0	0	0	0	1	0	0	0	0	0	0	0	0	1	1	42.317159	19.156679
2477	2015/008	0	0	0	0	0	0	0	0	0	0	0	0	0	0	0	0	0	0	0	0	0	0	0	0	0	0	0	0	0	0	0	0	0	0	0	0	0	1	0	0	0	0	0	0	0	0	0	1	42.314265	19.185085
2478	2015/009	0	0	0	0	0	0	0	0	0	0	0	0	0	0	0	0	0	0	0	0	0	0	0	0	0	0	0	0	0	0	0	0	0	0	0	0	0	1	0	0	0	0	0	0	0	0	0	1	42.280996	19.137376
2479	2015/010	0	0	0	0	0	0	0	0	0	0	0	0	0	0	1	0	0	0	0	0	0	0	0	0	0	0	0	0	0	0	0	0	0	0	0	0	0	1	0	0	0	0	0	0	0	0	1	1	42.245994	19.091719
2480	2015/011	0	0	0	0	0	0	0	0	0	0	0	0	1	0	0	0	0	0	0	0	0	0	0	0	0	0	0	0	0	0	0	0	0	0	0	0	0	1	0	0	0	0	0	0	1	0	0	1	1	42.220461	19.12407
2481	2015/012	1	0	0	0	0	0	0	0	0	0	0	0	0	0	0	0	0	0	0	0	0	0	0	0	0	0	0	0	0	0	0	0	0	0	0	0	0	1	0	0	0	0	0	0	0	0	1	1	42.225604	19.065948
2482	2015/013	0	0	0	0	0	0	0	0	0	0	0	0	0	0	0	0	0	0	0	0	0	0	0	0	0	0	0	0	0	0	0	0	0	0	0	0	0	0	0	0	0	0	0	0	0	0	1	1	42.251329	19.087318
2483	2015/014	0	0	0	0	0	1	0	0	0	0	0	0	0	0	1	0	0	0	0	0	0	0	0	0	0	0	0	0	0	0	0	0	0	0	0	0	0	0	0	0	0	0	0	0	0	0	1	1	42.252115	19.087521
2484	2015/015	1	0	0	0	0	0	0	0	0	0	0	0	0	0	1	0	0	0	0	0	0	0	0	0	0	0	0	0	0	0	0	0	0	0	0	0	0	1	0	0	0	0	0	0	0	0	1	1	42.254687	19.089632
2485	2015/016	1	0	0	1	0	1	0	0	0	0	0	0	0	0	0	0	0	0	0	0	0	0	0	0	0	0	0	1	0	0	0	0	0	0	0	0	0	1	0	0	0	0	0	0	0	0	1	1	42.305786	19.04807
2486	2015/017	1	0	0	0	0	0	0	0	0	0	0	0	1	0	0	0	0	0	0	0	0	0	0	0	0	0	0	0	1	0	0	0	0	0	0	0	0	0	0	0	0	0	0	0	0	0	1	1	42.352334	19.004744
2488	2015/019	1	0	0	0	0	0	0	0	0	0	0	0	0	0	1	0	0	0	0	0	0	0	0	0	0	0	0	0	1	0	0	0	0	0	0	0	0	1	0	0	0	0	0	0	0	0	1	1	42.365065	18.992377
2489	2015/020	1	0	0	0	0	1	0	0	0	0	0	0	1	0	0	0	0	0	0	0	1	0	0	0	0	0	0	0	1	0	0	0	0	0	0	0	0	0	0	0	0	0	0	0	0	0	1	1	42.381462	19.008372
2490	2015/021	1	0	0	0	0	0	0	0	0	0	0	0	1	0	0	0	0	0	0	0	0	0	0	0	0	0	0	1	0	0	0	0	0	0	0	0	0	1	0	0	0	0	0	0	0	0	1	1	42.386509	19.057116
2491	2015/022	0	0	0	0	0	0	0	0	0	0	0	0	0	0	0	0	0	0	0	0	0	0	0	0	0	0	0	0	0	0	0	0	0	0	0	0	0	0	0	0	0	0	0	0	0	0	0	1	42.375799	18.986275
2492	2015/023	1	0	0	0	0	0	0	0	0	0	0	0	0	0	1	0	0	0	0	0	0	0	0	0	0	0	0	0	0	0	0	0	0	0	0	0	0	1	0	0	0	0	0	0	0	0	1	1	42.400564	18.915211
2493	2015/024	1	0	0	0	0	0	0	0	0	0	0	0	0	0	0	0	0	0	0	0	0	0	0	0	0	0	0	0	0	0	0	0	0	0	0	0	0	0	0	0	0	0	0	1	0	0	0	1	42.305591	18.940213
2494	2015/025	1	0	0	0	0	0	0	0	1	0	0	0	0	0	1	0	0	0	0	0	0	0	0	0	0	0	0	0	0	0	0	0	0	0	0	0	0	0	0	0	0	0	0	1	0	0	1	1	42.227072	18.911729

2495	2015/026		0	0	0	0	0	1	0	0	0	0	0	0	0	0	1	0	0	0	1	0	0	0	0	0	0	0	0	0	0	0	1	0	0	0	0	0	0	0	0	0	0	0	0	1	0	0	1	1	42.217765	18.954533
2496	2015/027		1	0	0	0	0	0	0	0	0	0	0	0	0	0	0	0	0	0	1	0	0	0	0	0	0	0	0	0	0	0	0	0	0	0	0	0	1	0	0	0	0	0	0	0	0	0	1	1	42.164913	19.006938
2497	2015/028		1	0	0	1	0	0	0	0	0	0	0	0	0	0	1	0	0	0	1	0	0	0	0	0	0	0	0	0	0	0	0	0	0	0	0	0	0	0	0	0	0	0	0	0	0	0	1	1	42.153035	19.023731
2498	2015/029		1	0	0	0	0	0	0	0	0	0	0	0	0	0	0	0	0	0	0	0	0	0	0	0	0	0	0	0	0	0	0	0	0	0	0	0	1	0	0	0	0	0	0	0	0	0	0	1	42.137167	19.047373
2500	2015/031		0	0	0	0	0	1	0	0	0	0	0	0	0	0	0	0	0	0	0	0	0	0	0	0	0	0	0	0	0	0	0	0	0	0	0	0	0	0	0	0	0	0	0	0	0	0	1	1	42.057189	19.127777
2504	2015/035		0	0	0	0	0	0	0	0	0	0	0	0	0	0	0	0	0	0	0	0	0	0	0	0	0	0	0	0	0	0	0	0	0	0	0	0	1	0	0	0	0	0	0	1	0	0	1	1	42.050584	19.416234
2505	2015/036		0	0	0	0	0	0	0	0	0	0	0	0	0	0	0	0	0	0	0	0	0	0	0	0	0	0	0	0	0	0	0	0	0	0	0	0	0	0	0	0	0	0	0	0	0	0	0	1	42.051539	19.417129
2506	2015/037		1	0	0	0	0	1	0	0	0	0	0	0	0	0	1	0	0	0	1	0	0	0	0	0	0	0	0	0	0	0	1	0	0	0	0	0	1	0	0	0	0	0	0	1	0	0	1	1	42.052444	19.418228
2508	2015/039		0	0	0	0	0	0	0	0	0	0	0	0	0	0	0	0	0	0	0	0	0	0	0	0	0	0	0	0	0	0	0	0	0	0	0	0	1	0	0	0	0	0	0	0	0	0	0	1	41.898021	19.442456
2509	2015/040		0	0	0	0	0	0	0	0	0	0	0	0	0	0	1	0	0	0	0	0	0	0	0	0	0	0	0	0	0	0	0	0	0	0	0	0	1	0	0	0	0	0	0	0	0	0	1	1	41.857694	19.492337
2510	2015/041		0	0	0	1	0	1	0	0	0	0	0	0	1	0	0	0	0	0	0	0	0	0	0	0	0	0	0	1	1	0	0	0	0	0	0	0	1	0	0	0	0	0	0	1	0	0	0	1	41.876295	19.459235
2511	2015/042		0	0	0	1	0	0	0	0	0	1	0	0	0	0	0	0	0	0	0	0	0	0	0	0	0	0	0	0	0	0	0	0	0	0	0	0	1	0	0	0	0	0	0	0	0	0	0	1	41.883146	19.453315
2512	2015/043		1	0	0	0	0	1	0	0	0	1	0	0	0	0	1	0	0	0	0	0	0	0	0	0	0	0	0	0	0	0	0	0	0	0	0	0	1	0	0	0	0	0	0	1	0	0	0	1	41.916055	19.426763
2513	2015/044		0	0	0	0	0	0	0	0	0	0	0	0	0	0	1	0	0	0	0	0	0	0	0	0	0	0	0	0	0	0	0	0	0	0	0	0	1	0	0	0	0	0	0	0	0	0	0	1	41.927042	19.438694
2516	2015/047		1	0	0	0	0	1	0	0	0	1	0	0	0	1	0	0	0	0	1	0	0	0	0	0	0	0	0	0	1	0	0	0	0	0	0	0	1	0	0	0	0	0	0	1	0	0	1	1	42.058248	19.353537
2517	2015/048		1	0	0	1	0	0	0	0	0	0	0	0	0	0	0	0	0	0	0	0	0	0	0	0	0	0	0	0	1	0	0	0	0	0	0	0	1	0	0	0	0	0	0	0	0	0	1	1	42.190589	18.989795
2518	2015/049		1	0	0	0	0	1	0	0	0	0	0	0	0	0	0	0	0	0	0	0	0	0	0	0	0	0	0	0	0	0	0	0	0	0	0	0	0	0	0	0	0	0	0	0	0	0	0	1	42.431244	18.754857
2519	2015/050		1	0	0	0	0	0	0	0	0	1	0	0	1	0	1	0	0	0	1	0	0	0	0	0	0	0	0	1	0	0	0	0	0	0	0	0	1	0	0	0	0	0	0	0	0	0	1	1	42.4269	18.7756
2521	2015/052		0	0	0	0	0	0	0	0	0	0	0	0	1	0	0	0	0	0	0	0	0	0	0	0	0	0	0	0	0	0	0	0	0	0	0	0	0	0	0	0	0	0	0	0	0	0	1	1	42.409482	18.778685
2522	2015/053		1	0	0	0	0	1	0	0	0	1	0	0	1	0	0	0	0	0	0	0	0	0	1	0	0	0	0	0	1	0	0	0	0	0	0	0	0	0	0	0	0	0	0	0	0	0	0	1	42.399268	18.783629
2523	2015/054		1	0	0	0	0	0	0	0	0	0	0	0	1	0	0	0	0	0	1	0	0	0	0	0	0	0	0	1	1	0	0	0	0	0	0	0	0	0	0	0	0	0	0	0	0	0	1	1	42.431639	18.812576
2526	2015/057		1	0	0	0	0	0	0	0	0	0	0	0	0	0	1	0	0	0	1	0	0	0	0	0	0	0	0	1	0	0	0	0	0	0	0	0	0	0	0	0	0	1	0	0	0	0	0	1	42.487689	18.560329
2527	2015/058		0	0	0	0	0	0	0	0	1	0	0	0	0	0	1	0	0	0	0	0	0	0	0	0	0	0	0	0	0	0	0	0	0	0	0	0	1	0	0	0	0	0	0	0	0	0	0	1	42.445502	18.504971
2528	2015/059		1	0	0	0	0	0	0	0	0	1	0	0	1	0	0	0	0	0	1	0	1	0	0	0	0	0	0	0	0	0	0	0	0	0	0	0	0	0	0	0	0	0	0	0	0	0	1	1	42.731904	18.938231
2529	2015/060		1	0	0	0	0	1	0	0	0	0	0	0	1	0	0	0	0	0	0	0	0	0	0	0	0	0	0	0	1	0	0	0	0	0	0	0	1	0	0	0	0	1	0	1	0	0	0	1	42.675074	19.029887
2534	2015/065		0	0	0	0	0	1	0	0	1	0	0	0	0	1	0	1	0	0	1	0	0	0	0	0	0	0	0	0	0	1	0	0	0	0	0	0	0	0	0	0	0	0	0	0	0	1	1	3	40.94447231	20.82700524
2535	2015/065B		0	0	0	0	0	0	0	0	1	1	0	0	0	0	0	0	0	0	0	0	0	0	0	0	0	0	0	0	0	1	0	0	0	0	0	0	0	0	0	0	0	0	0	0	0	0	1	2	40.94391751	20.82827174
2536	2015/065C		0	0	0	0	0	1	0	0	0	1	0	0	1	0	0	0	0	0	0	0	1	0	0	0	0	0	0	0	0	1	0	0	0	0	0	0	0	0	0	0	0	0	0	0	0	0	1	1	40.9429225	20.82516398
2537	2015/066		0	0	0	0	0	1	0	0	0	0	0	0	0	0	1	0	0	0	0	0	0	0	0	0	0	0	0	0	0	1	0	0	0	0	0	0	0	0	0	0	0	0	0	0	0	0	1	1	40.88723372	20.97285219
2538	2015/067		0	0	0	0	0	1	0	0	1	0	0	0	0	1	0	0	0	0	0	0	0	0	0	0	0	0	0	0	0	1	0	0	0	0	0	0	0	0	0	0	0	0	0	0	0	0	1	1	40.88087177	20.95902013
2540	2015/069		0	0	0	0	0	0	0	0	1	0	0	0	0	0	0	0	0	0	0	0	0	0	0	0	0	0	0	0	0	0	0	0	0	0	0	0	0	0	0	0	0	0	0	0	0	0	0	1	40.86990623	20.92949186
2541	2015/070		0	0	0	0	0	1	0	0	0	0	0	0	0	1	1	0	0	0	1	0	0	0	0	0	0	0	0	0	0	1	0	0	0	0	0	0	0	0	0	0	0	0	0	0	0	0	1	1	40.84218845	20.96164442
2542	2015/071		0	0	0	0	0	0	0	0	0	0	0	0	0	0	0	0	0	0	0	0	0	0	0	0	0	0	0	0	0	0	0	0	0	0	0	0	0	0	0	0	0	0	0	0	0	0	1	1	40.8584	20.9474
2544	2015/073		0	0	0	0	0	0	0	0	0	0	0	0	0	0	0	0	0	0	0	0	0	0	0	0	0	0	0	0	0	0	0	0	0	0	0	0	0	0	0	0	0	0	0	0	0	0	0	1	41.30277654	20.62352984
2545	2015/074		0	0	0	0	0	0	0	0	1	0	0	0	0	0	0	0	0	0	0	0	0	0	0	0	0	0	0	0	0	0	0	0	0	0	0	0	0	0	0	0	0	0	0	0	0	0	0	3	41.39583377	20.58952983
2546	2015/075		0	0	0	0	0	0	0	0	0	1	0	0	1	0	0	0	0	0	0	0	0	0	0	0	0	0	0	0	0	0	0	0	0	0	0	0	0	0	0	0	0	0	0	0	0	0	0	1	41.39471672	20.59765541
2551	2015/079		0	0	0	0	0	1	0	0	1	1	0	0	1	1	1	0	0	1	1	0	0	0	0	0	0	0	0	0	0	0	0	0	0	0	0	0	0	0	0	0	0	0	0	0	0	0	0	1	41.85151082	20.32823372
2552	2015/080		0	0	0	0	0	0	0	0	0	0	0	0	0	0	0	0	0	1	0	0	0	0	0	0	0	0	0	0	0	0	0	0	0	0	0	0	0	0	0	0	0	0	0	0	0	0	0	1	41.84653482	20.33040832
2555	2015/083		1	0	0	0	0	1	0	0	1	0	0	0	1	0	1	0	0	0	0	0	0	0	0	0	0	0	0	0	0	0	0	0	0	0	0	0	0	0	0	0	0	0	0	0	0	0	1	1	41.88304371	20.33700555
2556	2015/084		0	0	0	0	0	0	0	0	0	1	0	0	0	0	0	0	0	1	0	0	0	0	0	0	0	0	0	0	0	0	0	0	0	0	0	0	0	0	0	0	0	0	0	0	0	0	1	1	41.9253802	20.35690787
2558	2015/086		0	0	0	0	0	0	0	0	0	0	0	0	0	0	0	0	0	0	0	0	0	0	0	0	0	0	0	0	0	0	0	0	0	0	0	0	0	0	0	0	0	0	0	0	0	0	0	1	41.94538445	20.37157402
2559	2015/087		1	0	0	0	0	1	0	0	0	1	0	0	0	0	0	0	0	1	1	0	0	0	0	0	0	0	0	1	0	0	0	0	0	0	0	0	0	0	0	0	0	0	0	0	0	0	0	1	41.95019332	20.38137884
2560	2015/088		0	0	0	0	0	1	0	0	0	0	0	0	1	0	0	0	0	0	0	0	0	0	0	0	0	0	0	0	0	0	0	0	0	0	0	0	0	0	0	0	0	0	0	0	0	0	0	1	42.10154166	20.42091854
2561	2015/089		0	0	0	0	0	0	0	0	0	0	0	0	0	0	0	0	0	0	0	0	0	0	0	0	0	0	0	0	0	0	0	0	0	0	0	0	0	0	0	0	0	0	0	0	0	0	0	1	42.19204559	20.41665978
2562	2015/090		0	0	0	0	0	1	0	0	0	0	0	0	1	0	0	0	0	0	1	0	0	0	0	0	0	0	0	0	0	0	0	0	0	0	0	0	1	0	0	0	0	0	0	0	0	0	0	1	42.34468063	20.03529819
2563	2015/091		1	0	0	0	0	1	0	0	1	1	0	0	1	1	0	0	1	0	1	0	0	0	0	0	1	0	0	0	0	1	0	0	0	0	0	1	0	0	0	0	0	0	0	0	0	1	0	3	42.36687088	20.02698384
2564	2015/092		1	0	0	0	0	0	0	0	1	0	0	0	1	0	0	0	0	0	1	0	0	0	0	0	0	0	0	0	0	1	0	0	0	0	0	1	0	0	0	0	0	0	0	0	0	1	0	3	42.36780253	20.009351
2567	2015/094		0	0	0	0	0	0	0	0	0	0	0	0	0	0	0	0	0	0	0	0	0	0	0	0	0	0	0	0	0	0	0	0	0	0	0	0	1	0	0	0	0	0	0	0	0	0	0	1	42.35562044	20.0701711
2574	2015/101		1	0	0	0	0	0	0	0	0	1	0	0	0	0	0	0	0	0	1	0	0	0	0	1	0	0	0	0	0	0	0	0	0	0	0	0	1	0	0	0	0	0	0	0	0	0	0	1	42.12477369	19.61498422
2575	2015/102		1	0	0	0	0	1	0	0	0	1	0	0	0	0	0	0	0	0	0	0	0	0	0	0	0	0	0	0	0	0	0	0	0	0	0	0	1	0	0	0	0	0	0	0	0	0	0	1	42.32498316	19.59398403
2576	2015/103		1	0	1	0	0	1	0	0	0	1	0	0	0	0	1	0	0	0	0	0	0	0	0	1	0	0	0	0	0	0	0	0	0	0	0	0	1	0	0	0	0	0	0	0	0	0	0	1	42.33062913	19.60567578
2577	2015/104		1	0	0	0	0	0	0	0	0	0	0	0	0	0	0	0	0	0	0	0	0	0	0	0	0	0	0	0	0	0	0	0	0	0	0	0	0	0	0	0	0	0	0	0	0	0	0	1	42.276362	19.516466
2578	2015/105		0	0	0	0	0	0	0	0	0	1	0	0	0	0	0	0	0	0	0	0	0	0	0	1	0	0	0	0	0	0	0	0	0	0	0	0	0	0	0	0	0	0	0	0	0	0	0	1	42.31955177	19.36934819
2581		0	1	0	0	0	0	0	0	0	0	0	0	0	0	0	0	0	0	0	1	0	0	0	0	0	0	0	0	0	0	0	0	0	0	0	0	0	0	0	0	0	0	0	0	0	0	0	0	3	46.378056	14.207361
2582		0	0	0	0	0	0	0	0	0	0	0	0	0	0	0	0	0	0	0	1	0	0	0	0	0	0	0	0	0	0	0	0	0	0	0	0	0	0	0	0	0	0	0	0	0	0	0	0	3	40.107694	21.021167
2583		0	0	0	0	0	0	0	0	0	0	0	0	0	0	0	0	0	0	0	1	0	0	0	0	0	0	0	0	0	0	0	0	0	0	0	0	0	0	0	0	0	0	0	0	0	0	0	0	1	44.60935	22.274417
2584	DL85		0	0	0	0	0	0	0	0	0	0	0	0	0	0	0	0	0	0	1	0	0	0	0	0	0	0	0	0	0	0	0	0	0	0	0	0	0	0	0	0	0	0	0	0	0	0	0	1	45.029186	14.497518
2585	DL250		0	0	0	0	0	0	0	0	0	0	0	0	0	0	0	0	0	0	1	0	0	0	0	0	0	0	0	0	0	0	0	0	0	0	0	0	0	0	0	0	0	0	0	0	0	0	0	1	44.6988	14.9082
2586	DL152		0	0	0	0	0	0	0	0	0	0	0	0	0	0	0	0	0	0	1	0	0	0	0	0	0	0	0	0	0	0	0	0	0	0	0	0	0	0	0	0	0	0	0	0	0	0	0	1	44.292212	15.175082
2587		0	1	0	0	0	0	0	0	0	0	0	0	0	0	0	0	0	0	0	0	0	0	0	0	0	0	0	0	0	0	0	0	0	0	0	0	0	0	0	0	0	0	0	0	0	0	0	0	1	43.511722	18.671806
2588		0	1	0	0	0	0	0	0	0	0	0	0	0	0	0	0	0	0	0	0	0	0	0	0	0	0	0	0	0	0	0	0	0	0	0	0	0	0	0	0	0	0	0	0	0	0	0	0	1	43.724183	18.604183
2589		0	1	0	0	0	0	0	0	0	0	0	0	0	0	0	0	0	0	0	0	0	0	0	0	0	0	0	0	0	0	0	0	0	0	0	0	0	0	0	0	0	0	0	0	0	0	0	0	1	43.7661	18.54655
2590		0	1	0	0	0	0	0	0	0	0	0	0	0	0	0	0	0	0	0	0	0	0	0	0	0	0	0	0	0	0	0	0	0	0	0	0	0	0	0	0	0	0	0	0	0	0	0	0	3	46.366542	14.306749
2591	DL20		1	0	0	0	0	0	0	0	0	0	0	0	0	0	0	0	0	0	0	0	0	0	0	0	0	0	0	0	0	0	0	0	0	0	0	0	0	0	0	0	0	0	0	0	0	0	0	3	46.406944	14.146111
2592	DL246		1	0	0	0	0	0	0	0	0	0	0	0	0	0	0	0	0	0	0	0	0	0	0	0	0	0	0	0	0	0	0	0	0	0	0	0	0	0	0	0	0	0	0	0	0	0	0	1	44.782167	14.985567
2593		0	1	0	0	0	0	0	0	0	0	0	0	0	0	0	0	0	0	0	0	0	0	0	0	0	0	0	0	0	0	0	0	0	0	0	0	0	0	0	0	0	0	0	0	0	0	0	0	1	44.17295	16.5761
2594		0	1	0	0	0	0	0	0	0	0	0	0	0	0	0	0	0	0	0	0	0	0	0	0	0	0	0	0	0	0	0	0	0	0	0	0	0	0	0	0	0	0	0	0	0	0	0	0	1	44.276211	15.874985
2595		0	1	0	0	0	0	0	0	0	0	0	0	0	0	0	0	0	0	0	0	0	0	0	0	0	0	0	0	0	0	0	0	0	0	0	0	0	0	0	0	0	0	0	0	0	0	0	0	1	45.745363	14.46735
2596	DL90		0	0	0	0	0	0	0	0	0	0	0	0	0	0	0	0	0	0	0	0	0	0	0	0	0	0	0	0	0	0	0	0	0	0	0	0	0	0	1	0	0	0	0	0	0	0	0	1	43.289528	18.617778
2597		0	0	0	0	0	0	0	0	0	0	0	0	0	0	0	0	0	0	0	0	0	0	0	0	0	0	0	0	0	0	0	0	0	0	0	0	0	0	0	1	0	0	0	0	0	0	0	0	1	44.60935	22.274417
2598		0	0	0	0	0	0	0	0	0	0	0	0	0	0	0	0	0	0	0	0	0	0	0	0	0	0	0	0	0	0	0	0	1	0	0	0	0	0	0	0	0	0	0	0	0	0	0	0	1	37.640833	22.206611
2599		0	0	0	0	0	0	0	0	0	0	0	0	0	0	0	0	0	0	0	0	0	0	0	0	0	0	0	0	0	0	0	0	1	0	0	0	0	0	0	0	0	0	0	0	0	0	0	0	1	36.8871	22.67085
2600		0	0	0	0	0	0	0	0	0	0	0	0	0	0	0	0	0	0	0	0	0	0	0	0	0	0	0	0	0	0	0	0	1	0	0	0	0	0	0	0	0	0	0	0	0	0	0	0	1	36.804183	22.6181
2601		0	0	0	0	0	0	0	0	0	0	0	0	0	0	0	0	0	0	0	0	0	0	0	0	0	0	0	0	0	0	0	0	1	0	0	0	0	0	0	0	0	0	0	0	0	0	0	0	1	37.335467	22.644983
2602		0	0	0	0	0	0	0	0	0	0	0	0	0	0	0	0	0	0	0	0	0	0	0	0	0	0	0	0	0	0	0	0	1	0	0	0	0	0	0	0	0	0	0	0	0	0	0	0	1	37.094917	22.33285
2603		0	0	0	0	0	0	0	0	0	0	0	0	0	0	0	0	0	0	0	0	0	0	0	0	0	0	0	0	0	0	0	0	1	0	0	0	0	0	0	0	0	0	0	0	0	0	0	0	1	37.079183	22.291133
2604		0	0	0	0	0	0	0	0	0	0	0	0	0	0	0	0	0	0	0	0	0	0	0	0	0	0	0	0	0	0	0	0	1	0	0	0	0	0	0	0	0	0	0	0	0	0	0	0	1	38.084983	22.033933
2605		0	1	0	0	0	0	0	0	0	0	0	0	0	0	0	0	0	0	0	0	0	0	0	0	0	0	0	0	0	0	0	0	1	0	0	0	0	0	0	0	0	0	0	0	0	0	0	0	1	37.243117	21.82745
2606		0	0	0	0	0	0	0	0	0	0	0	0	0	0	0	0	0	0	0	0	0	0	0	0	0	0	0	0	0	0	0	0	1	0	0	0	0	0	0	0	0	0	0	0	0	0	0	0	1	37.539633	22.046467
2607		0	0	0	0	0	0	0	0	0	0	0	0	0	0	0	0	0	0	0	0	0	0	0	0	0	0	0	0	0	0	0	0	1	0	0	0	0	0	0	0	0	0	0	0	0	0	0	0	1	37.533667	22.053183
2608		0	0	0	0	0	0	0	0	0	0	0	0	0	0	0	0	0	0	0	0	0	0	0	0	0	0	0	0	0	0	0	0	1	0	0	0	0	0	0	0	0	0	0	0	0	0	0	0	1	37.622183	22.050617
2609		0	0	0	0	0	0	0	0	0	0	0	0	0	0	0	0	0	0	0	0	0	0	0	0	0	0	0	0	0	0	0	0	1	0	0	0	0	0	0	0	0	0	0	0	0	0	0	0	1	37.9338	22.211783
2610		0	0	0	0	0	0	0	0	0	0	0	0	0	0	0	0	0	0	0	0	0	0	0	0	0	0	0	0	0	0	0	0	1	0	0	0	0	0	0	0	0	0	0	0	0	0	0	0	2	37.942383	22.23445
2611		0	1	0	0	0	0	0	0	0	0	0	0	0	0	0	0	0	0	0	0	0	0	0	0	0	0	0	0	0	0	0	0	1	0	0	0	0	0	0	0	0	0	0	0	0	0	0	0	1	37.9337	22.166183
2612		0	0	0	0	0	0	0	0	0	0	0	0	0	0	0	0	0	0	0	0	0	0	0	0	0	0	0	0	0	0	0	0	1	0	0	0	0	0	0	0	0	0	0	0	0	0	0	0	1	38.36505	22.128117
2613		0	0	0	0	0	0	0	0	0	0	0	0	0	0	0	0	0	0	0	0	0	0	0	0	0	0	0	0	0	0	0	0	1	0	0	0	0	0	0	0	0	0	0	0	0	0	0	0	1	38.347367	22.3141
2614		0	1	0	0	0	0	0	0	0	0	0	0	0	0	0	0	0	0	0	0	0	0	0	0	0	0	0	0	0	0	0	0	1	0	0	0	0	0	0	0	0	0	0	0	0	0	0	0	1	38.563783	22.348983
2615		0	1	0	0	0	0	0	0	0	0	0	0	0	0	0	0	0	0	0	0	0	0	0	0	0	0	0	0	0	0	0	0	1	0	0	0	0	0	0	0	0	0	0	0	0	0	0	0	1	38.600117	22.326617
2616		0	1	0	0	0	0	0	0	0	0	0	0	0	0	0	0	0	0	0	0	0	0	0	0	0	0	0	0	0	0	0	0	0	0	0	0	0	0	0	0	0	0	0	0	0	0	0	0	1	38.420278	22.242222
2617		0	1	0	0	0	0	0	0	0	0	0	0	0	0	0	0	0	0	0	0	0	0	0	0	0	0	0	0	0	0	0	0	0	0	0	0	0	0	0	0	0	0	0	0	0	0	0	0	1	38.624722	23.844444
2618		0	1	0	0	0	0	0	0	0	0	0	0	0	0	0	0	0	0	0	0	0	0	0	0	0	0	0	0	0	0	0	0	0	0	0	0	0	0	0	0	0	0	0	0	0	0	0	0	1	37.186667	21.913889
2619		0	1	0	0	0	0	0	0	0	0	0	0	0	0	0	0	0	0	0	0	0	0	0	0	0	0	0	0	0	0	0	0	0	0	0	0	0	0	0	0	0	0	0	0	0	0	0	0	1	39.967778	20.665278
2620		0	1	0	0	0	0	0	0	0	0	0	0	0	0	0	0	0	0	0	0	0	0	0	0	0	0	0	0	0	0	0	0	0	0	0	0	0	0	0	0	0	0	0	0	0	0	0	0	1	38.398778	22.005556
2621		0	1	0	0	0	0	1	0	0	0	0	0	0	1	0	0	0	0	0	1	0	0	0	0	0	0	0	0	0	0	1	0	0	0	0	0	0	0	0	1	0	0	0	0	0	0	0	0	1	44.101144	21.469257
2622		0	0	0	0	0	0	0	0	0	0	0	0	0	0	0	0	0	0	0	1	0	0	0	0	0	0	0	0	0	0	1	0	0	0	0	0	0	0	0	1	0	0	0	0	0	0	0	0	1	43.974361	21.515333
2623		0	1	0	0	0	0	0	0	0	0	0	0	0	1	0	0	0	0	0	1	0	0	0	0	0	1	0	0	0	0	1	0	0	0	0	0	0	0	0	1	0	0	0	0	0	0	0	0	1	44.028972	21.957889
2624		0	0	0	0	0	0	0	0	0	0	0	0	0	1	0	0	0	0	0	1	0	0	0	1	0	0	0	0	0	0	1	0	0	0	1	0	0	0	0	0	0	1	0	0	0	0	0	0	1	43.281361	22.062111
2625		0	0	0	0	0	0	0	0	0	0	0	0	0	1	0	0	0	0	0	1	0	0	0	0	0	0	0	0	0	0	1	0	0	0	0	0	0	0	0	0	0	0	0	0	0	0	0	0	1	43.795306	21.9035
2626		0	0	0	0	0	0	1	0	0	0	0	0	0	0	0	0	0	0	0	0	0	0	0	0	0	0	0	0	0	0	0	0	0	0	0	0	0	0	0	0	0	0	0	0	0	0	0	0	2	41.80347	21.300001
2627		0	1	0	0	0	0	0	0	0	0	0	0	0	1	0	0	0	0	0	1	0	0	0	0	0	0	0	0	0	0	0	0	0	0	0	0	0	0	0	0	0	1	0	0	0	0	0	0	1	43.020667	22.364306
2628		0	1	0	0	0	0	0	0	0	0	0	0	0	1	0	0	0	0	0	1	0	0	0	1	0	0	0	0	0	0	0	0	0	0	0	0	0	0	0	0	0	1	0	0	0	0	0	0	1	43.030389	22.388806
2629		0	0	0	0	0	0	0	0	0	0	1	0	0	0	0	0	0	0	0	1	0	0	0	0	0	0	0	0	0	0	0	0	0	0	0	0	0	0	0	0	0	1	0	0	0	0	0	0	1	43.429222	22.4175
2630		0	0	0	0	0	0	1	0	0	0	0	0	0	0	1	0	0	0	0	1	0	0	0	0	0	0	0	0	0	0	1	0	0	0	0	0	0	0	0	0	0	1	0	0	0	0	0	0	1	43.536528	22.285278
2631		0	0	0	0	0	0	0	0	0	0	0	0	0	0	0	0	0	0	0	1	0	0	0	0	0	0	0	0	0	0	0	0	0	0	0	0	0	0	0	0	0	0	0	0	0	0	0	0	1	43.760471	22.310321
2632		0	0	0	0	0	0	1	0	0	0	0	0	0	1	0	0	0	0	0	0	0	0	0	0	0	0	0	0	0	0	0	0	0	0	0	0	0	0	0	0	0	0	0	0	0	0	0	0	1	43.834444	21.9545
2633		0	1	0	0	0	0	1	0	0	1	0	0	0	1	0	0	0	0	1	1	0	0	0	1	0	1	1	0	0	0	1	0	0	0	0	0	0	0	0	0	0	1	0	0	0	0	0	0	3	44.382417	22.341694
2634		0	1	0	0	0	0	0	0	0	1	1	0	0	0	0	0	0	0	1	1	0	0	0	0	0	1	1	0	0	0	1	0	0	0	0	0	0	0	0	0	0	0	0	0	0	0	0	0	1	44.292306	22.277583
2635		0	1	0	0	0	0	1	0	0	1	1	0	0	1	0	0	1	1	1	1	0	1	1	1	0	1	1	0	0	0	1	0	0	0	0	0	0	0	0	0	0	0	0	0	0	0	0	0	1	44.185727	22.154682
2636		0	1	0	0	0	0	0	0	0	1	1	0	0	1	0	1	1	1	1	1	0	1	1	1	0	1	1	0	0	0	1	0	0	0	0	0	1	0	0	1	0	0	0	0	0	0	0	0	1	44.61375	22.275222
2637		0	0	0	0	0	0	0	0	0	0	0	0	0	1	0	0	0	0	0	0	0	1	0	0	0	0	0	0	0	0	1	0	0	0	0	0	0	0	0	0	0	0	0	0	0	0	0	0	3	44.441475	21.953446

site_no	site_code	prosobranch	Monti_L	habitat_type	Latitude	Longitude
3	1992/22	1	0	1	41.518	19.8008
16	1993/14	1	0	1	39.8644	20.0153
19	1993/16	1	0	1	40.25030556	20.06522222
20	1993/12	1	0	2	41.35	19.89
25	1994/53	0	0	1	41.5085	19.7961
28	1994/57	1	0	1	41.3603	19.8916
33	1994/62	0	0	1	41.8677	19.5665
34	1996/03	0	0	1	43.9511	19.989
36	1996/05	1	0	1	43.5988	19.7196
38	1996/07	0	0	1	43.36	19.58
39	1996/08	0	0	1	43.34	19.31
41	1996/10	0	0	1	43.02238333	19.15628333
42	1996/11	0	0	1	43.0003	19.1601
43	1996/12	0	0	1	42.9656	19.0952
46	1996/15	1	0	1	42.94591667	19.08355
48	1996/17	0	0	1	42.8981	19.0534
52	1996/21	1	0	1	42.5008	18.6927
53	1996/22	1	0	1	42.4213	18.7716
54	1996/23	0	0	2	42.1947	18.967
57	1996/26	0	0	1	42.2715	19.1254
58	1996/27	1	0	1	42.3732	19.3193
59	1996/28	1	0	1	42.3193	19.3696
60	1996/29	1	0	1	42.3741	19.4647
61	1996/30	1	0	1	42.41333333	19.50385
62	1996/31	1	0	1	42.3315	19.6005
64	1996/33	1	0	1	42.048	19.4976
66	1996/35	1	0	1	41.90006667	19.51298333
70	1996/39	1	0	1	41.3599	19.9146
73	1996/42	1	0	1	40.2627	19.5315
74	1996/43	1	0	3	40.2055	19.591
75	1996/44	1	0	1	40.1904	19.6049
77	1996/46	1	0	1	40.069	19.8588
78	1996/47	1	0	1	39.9195	20.1404
79	1996/48	1	0	1	39.9479	20.25
82	1996/51	1	0	1	40.2086	20.4147
83	1996/52	0	0	1	40.1608	20.5682
84	1996/53	1	0	1	40.2441	20.6386
85	1996/54	1	0	1	40.2656	20.59991667
88	1996/57	1	0	1	40.9029	20.7298
89	1996/58	0	0	1	43.6308	20.5434
92	1996/61	0	0	1	40.44	21.34
94	1996/63	1	0	1	39.5603	20.469
95	1996/64	1	0	1	39.704	19.806
96	1996/65	1	0	1	39.72	19.87
97	1996/66	1	0	1	39.73	19.9
98	1996/67	1	0	1	39.736	19.798
100	1996/69	1	0	1	39.7054	19.7611
102	1996/71	1	0	1	39.6436	20.3133
103	1996/72	1	0	1	39.5	20.52
105	1996/74	1	0	1	39.866	20.717
114	1996/83	0	0	1	41.7	20.742
115	2000/14	1	0	1	43.28956667	18.85161667
116	2000/15	0	0	1	43.2737	18.8428
117	2000/16	0	0	1	43.1717	18.8519
118	2000/17	1	0	1	42.97508333	18.90563333
119	2000/18	1	0	1	42.8932	19.07138333
120	2000/19	0	0	1	42.92766667	19.0712
122	2000/21	0	0	1	42.9424	19.10423333
125	2000/23	1	0	1	43.151	19.27883333
126	2000/24	1	0	1	43.1358	19.2999
127	2000/25	0	0	1	43.1241	19.3246
128	2000/26	0	0	1	43.0726	19.38336667
130	2000/28	0	0	1	42.88908333	19.53473333
131	2000/29	1	0	1	42.852	19.5271
132	2000/30	0	0	3	42.7415	19.6898
134	2000/32	1	0	1	42.707	19.3771
135	2000/33	1	0	1	42.6908	19.3796
136	2000/34	1	0	1	42.2436	19.0952
138	2000/35	1	0	1	42.27651667	19.07088333
141	2000/38	0	0	1	42.1487	19.12
142	2000/39	1	0	1	42.141	19.1377
146	2000/43	0	0	1	41.9247	19.2049
147	2000/44	1	0	1	41.979361	19.259112
148	2000/45	1	0	1	42.002	19.2639
149	2000/46	1	0	1	41.9864	19.3156
150	2000/47	1	0	1	42.04765	19.3229
151	2000/48	1	0	1	42.0645	19.13193333
152	2000/49	0	0	1	42.2048	18.9575
153	2000/50	1	0	1	42.2766	18.8869
157	2000/54	1	0	1	42.42026667	18.7938
158	2000/55	1	0	1	42.42156667	18.79798333
159	2000/56	1	0	1	42.434	18.8189
161	2000/58	1	0	1	42.4744	18.5362
164	2000/61	1	0	1	42.59	18.67
165	2001/002	0	0	1	43.96716667	19.96116667
168	2001/005	0	0	1	43.33	19.669
169	2001/006	0	0	1	43.2659	19.7142
170	2001/007	0	0	1	43.89483333	19.53866667
172	2001/009	1	0	1	42.697	19.374
173	2001/010	1	0	1	42.614	19.39
174	2001/011	1	0	1	42.51733333	19.19566667
176	2001/013	0	0	1	42.11816667	19.57583333
177	2001/014	1	0	1	42.0446	19.4945
178	2001/015	1	0	1	42.01583333	19.62183333
181	2001/018	1	0	1	41.8895	20.12083333
182	2001/019	1	0	1	41.92016667	20.14516667
183	2001/020	1	0	1	41.76433333	19.7815
187	2001/024	1	1	1	41.37963	19.8607
188	2001/025	1	1	1	41.3859	19.861
189	2001/026	1	1	1	41.39416667	19.88433333
190	2001/027	1	1	1	41.40216667	19.90116667
191	2001/028	1	0	1	41.35466667	19.916
194	2001/031	1	0	1	41.23483333	19.97716667
196	2001/033	1	0	1	40.4698	20.2561
197	2001/034	0	0	1	40.49466667	20.2705
199	2001/036	1	0	1	40.6972	19.9535
200	2001/037	0	0	2	40.5865	19.99666667
201	2001/038	0	0	1	40.542	20.007
202	2001/039	1	0	1	40.50283333	19.9845
203	2001/040	0	0	1	40.34783333	20.16016667
206	2001/042	0	0	1	40.23683333	20.35183333
208	2001/044	1	0	1	40.29366667	20.01716667
209	2001/045	1	0	1	40.30133333	20.02166667
216	2002/095	1	0	1	42.3201	19.58575
218	2002/097	1	0	3	42.39205	19.70716667
219	2002/098	1	0	1	42.39228333	19.7297
223	2002/102	0	0	1	41.72	19.77
226	2002/105	1	0	1	41.83253333	20.10056667
227	2002/106	0	0	1	41.34731667	19.89703333
228	2002/107	1	0	1	41.32651667	19.94403333
229	2002/108	1	0	1	41.32681667	19.95755
230	2002/109	1	0	1	41.34036667	19.95628333
232	2002/111	1	0	1	41.37216667	20.005
233	2002/112	1	0	1	41.35913333	20.0273
237	2002/116	0	0	1	41.19851667	19.93473333
244	2002/123	0	1	1	41.16566667	20.23773333
253	2002/132	1	0	1	41.51596667	20.3906
256	2002/135	0	0	1	41.42086667	20.47971667
257	2002/136	0	0	1	41.41833333	20.45686667
260	2002/139	0	0	1	41.47181667	20.0967
263	2002/142	1	0	3	41.52085	19.8983
264	2002/143	1	0	1	41.52058333	19.85606667
265	2002/144	1	0	1	41.5229	19.81645
267	2002/146	1	0	1	42.65166667	19.37838333
268	2002/147	1	0	1	42.766	19.389
269	2002/148	0	0	1	43.1552	19.78068333
270	2003/013	0	0	1	43.97628333	19.9338
272	2003/015	1	0	1	42.47108333	19.3651
277	2003/020	0	0	1	42.06425	20.49298333
278	2003/021	1	0	1	42.07275	20.4599
281	2003/023	1	1	1	41.95983333	20.39375
286	2003/028	0	0	1	41.63225	20.2217
289	2003/030	0	0	1	41.64333333	20.15716667
293	2003/034	1	0	1	41.69501667	19.81418333
294	2003/035	1	0	1	41.76071667	19.77716667
295	2003/036	1	0	1	41.78833333	20.10681667
296	2003/037	1	0	1	41.83611667	20.17396667
297	2003/037B	0	0	1	41.83174	20.17713
302	2003/041	1	0	1	41.79415	20.11205
303	2003/042	1	0	1	41.2278	19.98141667
305	2003/044	1	0	1	40.92798333	20.03055
306	2003/045	0	0	1	41.11203333	20.08118333
308	2003/047	1	1	1	41.1336	20.12776667
309	2003/048	1	1	1	41.16756667	20.24983333
310	2003/049	1	0	1	41.09621667	20.44281667
312	2003/050	1	1	1	41.0937	20.44025
313	2003/051	1	0	1	41.06506667	20.59711667
318	2003/056	1	0	1	40.93781667	20.45406667
319	2003/057	1	1	1	40.92096667	20.4491
320	2003/058	1	0	1	40.92333333	20.44386667
327	2003/064	0	0	1	40.7891	20.90725
329	2003/066	0	0	1	40.52556667	20.68865
330	2003/067	0	0	1	40.52835	20.63436667
331	2003/068	1	0	1	40.5333	20.62276667
332	2003/069	1	0	1	40.29153333	20.6281
334	2003/071	0	0	1	40.23783333	20.64371667
335	2003/072	0	0	1	40.15266667	20.61966667
336	2003/073	0	0	1	40.15093333	20.59491667
339	2003/076	1	0	1	40.69201667	20.50015
342	2003/078	1	0	1	40.71361667	20.36178333
343	2003/079	1	0	1	40.88303333	20.24433333
345	2003/081	1	0	1	42.38846667	19.7363
346	2003/082	1	0	1	42.40228333	19.76318333
347	2003/083	0	0	1	42.42773333	19.76173333
348	2003/084	1	0	1	42.4438	19.77095

349	2003/085		0	0	0	0	0	0	0	0	0	0	0	0	0	0	0	0	0	0	0	0	0	1	0	0	0	0	0	0	0	0	0	0	1	0	0	0	0	0	0	0	0	0	0	0	0	0	0	0	0	0	0	0	0	0	0	0	0	0	0	0	0	0	0	0	0	0	0	0	0	2	42.41478333	19.75681667
350	2003/086		1	0	0	0	0	0	1	0	1	0	0	0	1	1	0	0	0	0	0	0	0	0	0	1	0	0	0	0	0	0	0	0	0	0	0	0	0	0	0	1	0	0	0	0	0	0	0	0	0	0	0	0	0	0	0	0	0	0	0	0	0	0	0	0	0	0	0	0	0	3	42.40188333	19.7493
351	2003/087		0	0	0	0	0	0	0	0	0	0	0	0	0	0	0	0	0	0	0	0	0	0	0	0	0	0	0	0	0	0	1	0	0	0	0	0	0	0	0	1	0	0	0	0	0	0	0	0	0	0	0	0	0	0	0	0	0	0	0	0	0	0	0	0	0	0	0	0	0	1	42.38951667	19.72703333
353	2003/089		1	0	0	0	0	0	0	0	0	1	0	0	0	0	0	0	0	0	0	0	0	0	0	0	0	0	0	0	0	0	0	0	0	0	0	0	0	0	0	1	0	0	0	0	0	0	0	0	0	0	0	0	0	0	0	0	0	0	0	0	0	0	0	0	0	0	0	0	0	1	42.43933333	19.53088333
354	2003/090		1	0	0	0	0	1	0	0	0	1	0	0	0	0	0	0	0	0	0	0	0	1	0	0	0	0	0	0	0	0	1	1	0	0	0	0	0	0	0	1	0	0	0	0	0	0	0	0	0	0	0	0	0	0	0	0	0	0	0	0	0	0	0	0	0	0	0	0	0	1	42.48103333	19.58266667
355	2003/091		1	0	0	0	0	0	0	0	0	1	0	0	0	0	0	0	0	0	0	0	0	0	0	0	0	0	0	0	0	0	1	0	0	0	0	0	0	0	0	0	0	0	0	0	0	0	0	0	0	0	0	0	0	0	0	0	0	0	0	0	0	0	0	0	0	0	0	0	0	1	42.5077	19.61598333
356	2003/092		1	0	1	0	0	0	0	0	1	1	0	0	0	0	1	0	0	0	0	0	0	1	0	0	0	0	0	0	0	0	0	0	0	0	0	0	0	0	0	1	0	0	0	0	0	0	0	0	0	0	0	0	0	0	0	0	0	0	0	0	0	0	0	0	0	0	0	0	0	1	42.52241667	19.6319
358	2003/094		0	0	0	0	0	1	0	0	1	0	0	0	0	0	1	0	0	0	0	0	0	1	0	1	0	0	0	0	0	0	0	0	0	0	0	0	0	0	1	0	0	0	0	0	0	0	0	0	0	0	0	0	0	0	0	0	0	0	0	0	0	0	0	0	0	0	0	0	0	1	42.5364	19.6884
359	2003/095		1	0	0	0	0	0	0	0	0	0	0	0	0	0	0	0	0	0	0	0	0	1	0	0	0	0	0	0	0	0	0	0	0	0	0	0	0	0	0	1	0	0	0	0	0	0	0	0	0	0	0	0	0	0	0	0	0	0	0	0	0	0	0	0	0	0	0	0	0	1	42.53228333	19.65086667
360		0	0	0	0	0	0	0	0	0	0	0	0	0	1	0	0	0	0	0	0	0	0	0	0	0	0	0	0	0	0	0	0	1	0	0	0	0	0	0	0	0	0	0	0	0	0	0	0	0	0	0	0	0	0	0	0	0	0	0	1	0	0	0	0	0	0	0	1	42.6306	19.0085
361		0	1	0	0	0	0	0	0	0	0	0	0	0	1	0	0	0	0	0	0	0	0	0	0	0	0	0	0	0	0	0	0	0	0	0	0	0	0	0	0	0	0	0	0	0	0	0	0	0	0	0	0	0	0	0	0	0	0	0	1	0	0	0	0	0	0	0	1	42.4203	18.8239
362		0	1	0	0	0	0	0	0	0	0	0	0	0	1	0	0	0	0	0	0	0	0	1	0	0	0	0	0	0	0	0	0	0	0	1	0	0	0	0	0	0	0	0	0	0	0	0	0	0	0	0	0	0	0	0	0	0	0	0	0	0	0	0	0	0	0	0	1	42.5208	18.6908
363	2004/003		0	0	0	0	0	1	0	0	1	0	0	0	1	0	0	0	0	0	0	0	0	0	0	0	0	0	0	0	0	0	0	0	0	0	0	0	0	0	0	0	0	0	0	0	0	0	0	0	0	0	0	0	0	0	0	0	0	0	0	0	0	0	0	0	0	0	0	0	0	1	41.8265	21.6865
364	2004/004		0	0	0	0	0	1	0	0	0	0	0	0	0	0	0	0	0	0	0	0	0	0	0	0	0	0	0	0	0	0	0	0	1	0	0	0	1	0	0	0	0	0	0	0	0	0	0	0	0	0	0	0	0	0	0	0	0	0	0	0	0	0	0	0	0	0	0	0	0	1	41.704	21.7885
365	2004/005		0	0	0	0	0	0	0	0	0	0	0	0	1	0	0	0	0	0	0	0	0	1	0	0	0	0	0	0	0	0	0	0	1	0	0	0	1	0	0	0	0	0	0	0	0	0	0	0	0	0	0	0	0	0	0	0	0	0	0	0	0	0	0	0	0	0	0	0	0	1	41.70633333	21.78133333
367	2004/007		0	0	0	0	0	1	0	0	1	1	0	0	0	0	0	1	0	0	1	0	0	0	0	1	0	1	0	0	0	0	0	0	1	0	0	0	0	0	1	0	0	0	0	0	0	0	0	0	0	0	0	0	0	0	0	0	0	1	0	0	0	0	0	0	0	0	0	0	0	0	0	0	0	1	41.681	21.43033333
368	2004/009		1	0	1	0	0	1	0	0	0	1	0	0	1	0	1	1	0	0	1	0	0	1	0	0	0	0	0	1	0	0	0	0	0	0	0	1	0	0	0	0	0	0	0	0	0	0	0	0	0	0	0	0	0	0	0	0	0	0	0	0	0	0	0	0	0	0	0	0	0	1	41.43683333	21.86716667
369	2004/010		1	0	0	0	0	1	0	0	1	1	0	0	1	0	1	1	0	0	1	0	0	1	0	0	0	0	0	0	0	0	0	1	0	0	0	1	0	0	0	0	0	0	0	0	0	0	0	0	0	0	0	0	0	0	0	0	0	0	0	0	0	0	0	0	0	0	0	0	0	1	41.43666667	21.81666667
370	2004/011		0	0	0	0	0	1	0	0	0	0	0	0	0	0	1	0	0	0	0	0	0	0	0	0	0	0	0	0	0	0	0	1	0	0	0	0	0	0	0	0	0	0	1	0	0	0	1	0	0	0	0	0	0	0	0	0	0	0	0	0	0	0	0	0	0	0	0	0	0	1	41.3655	21.7165
373	2004/014		0	0	0	0	0	1	0	0	1	0	0	0	0	1	1	1	0	0	1	0	0	1	0	0	0	1	0	0	1	0	0	0	1	0	0	0	0	0	0	0	0	0	0	0	0	0	0	0	0	0	0	0	0	0	0	0	0	0	0	0	0	0	0	0	0	0	0	0	0	1	40.935	20.93666667
374	2004/015		0	0	0	0	0	1	0	0	0	0	0	0	0	0	1	0	0	0	0	0	0	0	0	0	0	0	0	0	0	0	0	0	0	0	0	0	0	0	0	0	0	0	0	0	0	0	0	0	0	0	0	0	0	0	0	0	0	0	0	0	0	0	0	0	0	0	0	0	0	1	40.96883333	20.91016667
376	2004/017		0	0	0	0	0	1	0	0	0	0	0	0	0	1	1	0	0	0	0	0	0	0	0	0	0	0	0	0	0	0	0	0	1	0	0	0	0	0	0	0	0	0	0	0	0	0	0	0	0	0	0	1	0	0	0	0	0	0	0	0	0	0	0	0	0	0	0	0	0	2	40.95583333	20.82033333
377	2004/018		0	0	0	0	0	1	0	0	1	0	0	0	1	0	0	1	0	0	1	0	0	1	0	0	0	0	0	0	0	0	0	0	0	0	0	0	0	0	0	0	0	0	0	0	0	0	0	0	0	0	0	1	1	0	0	0	0	0	1	0	0	0	0	0	0	0	0	0	0	1	40.954	20.8125
379	2004/020		0	0	0	0	0	0	0	0	1	1	0	0	0	0	1	1	0	0	1	0	0	0	0	0	0	0	0	0	0	0	0	0	0	0	0	0	0	0	0	0	0	0	0	0	0	0	0	0	0	0	0	0	1	0	0	0	0	0	0	0	0	0	0	0	0	0	0	0	0	3	40.97116667	20.82066667
380	2004/021		0	0	0	0	0	1	0	0	1	1	0	0	1	0	0	1	0	0	1	0	0	0	0	0	0	0	0	0	0	0	0	0	1	0	0	0	0	0	0	0	0	0	0	0	0	0	0	0	0	0	0	0	0	0	0	0	0	0	1	0	0	0	0	0	0	0	0	0	0	1	40.96466667	20.81466667
388	2004/028		0	0	0	0	0	1	0	0	1	1	0	0	1	1	1	0	0	0	0	0	0	1	0	0	0	1	0	0	0	0	0	0	1	0	0	0	0	0	0	0	0	0	0	0	0	0	0	0	0	0	0	1	0	0	0	0	0	0	2	0	0	0	0	0	0	0	0	0	0	1	40.9656	20.78585
391	2004/031		1	0	0	0	0	1	0	0	0	0	0	0	0	0	1	0	0	0	0	0	0	0	0	1	0	1	0	0	0	0	0	0	1	0	0	0	0	0	0	0	0	0	0	0	0	0	0	0	0	0	0	1	0	0	0	0	0	0	0	0	0	0	0	0	0	0	0	0	0	1	41.074	20.803
392	2004/032		0	0	0	0	0	1	0	0	0	0	0	0	0	1	1	0	0	0	0	0	0	0	0	0	0	0	0	0	0	0	0	0	0	0	0	0	0	0	0	0	0	0	0	0	0	0	0	0	0	0	0	1	0	0	0	0	0	0	0	0	0	0	0	1	0	0	0	0	0	1	41.103	20.81433333
393	2004/033		0	0	0	0	0	1	0	0	0	0	0	0	1	0	0	0	0	0	0	0	0	0	0	0	0	1	0	0	0	0	0	0	1	0	0	0	0	0	0	0	0	0	0	0	0	0	0	0	0	0	0	1	0	0	0	0	0	0	0	0	0	0	0	0	0	0	0	0	0	1	41.11133333	20.7905
394	2004/034		0	0	0	0	0	1	0	0	1	1	0	0	1	0	1	0	0	0	0	0	0	1	0	1	0	1	0	1	1	0	0	0	1	0	0	0	0	0	0	0	0	0	0	0	0	0	0	0	0	0	0	0	0	0	0	0	0	0	0	0	0	0	0	0	0	0	0	0	1	1	41.19783333	20.91733333
396	2004/036		0	0	0	0	0	1	0	0	0	0	0	0	1	0	0	0	0	0	0	0	0	0	0	0	0	0	0	0	0	0	0	0	1	0	0	0	0	0	0	0	0	0	0	0	0	0	0	0	0	0	0	1	0	0	0	0	0	0	0	0	0	0	0	0	0	0	0	0	0	1	41.13883333	20.7625
397	2004/037		0	0	0	0	0	0	0	0	0	0	0	0	0	0	0	0	0	0	0	0	0	0	0	0	0	0	0	0	0	0	0	0	0	0	0	0	0	0	0	0	0	0	0	0	0	0	0	0	0	0	0	1	0	0	0	0	0	0	0	0	0	0	0	0	0	0	0	0	0	1	41.18283333	20.63233333
399	2004/038		0	0	0	0	0	1	0	0	1	0	0	0	0	0	0	0	0	0	0	0	0	1	0	0	0	0	0	0	0	0	0	0	0	0	0	0	0	0	0	0	0	0	0	0	0	0	0	0	0	0	0	1	0	0	0	0	0	0	0	0	0	0	1	0	0	0	0	0	0	1	41.14933333	20.64933333
401	2004/040		0	0	0	0	0	1	0	0	1	0	0	0	0	0	0	0	0	0	0	0	0	0	0	0	0	0	0	0	0	0	0	0	0	0	0	0	0	0	0	0	0	0	0	0	0	0	0	0	0	0	0	1	0	0	0	0	0	0	0	0	0	0	0	0	0	1	0	0	0	1	41.3355	20.6345
406	2004/045		1	0	0	0	0	1	0	0	1	0	0	0	1	0	1	0	0	0	0	0	0	0	0	0	0	0	0	0	0	0	0	1	0	0	0	0	0	0	0	0	0	0	0	0	0	0	0	0	0	0	1	1	1	0	0	0	0	0	0	0	0	0	0	0	0	0	0	0	0	3	41.621	20.60916667
418	2004/057		0	0	0	0	0	0	0	0	1	1	0	0	1	0	1	1	0	0	1	0	0	0	0	1	0	1	0	0	0	0	0	0	1	0	0	0	0	0	1	0	0	0	0	0	0	0	0	0	0	0	0	0	1	0	0	0	0	0	0	0	0	0	0	0	0	0	0	0	0	1	42.02083333	20.902
419	2004/058		0	0	0	0	0	1	0	0	0	0	0	0	0	0	1	0	0	0	0	0	0	0	0	0	0	0	0	0	0	0	0	0	0	0	0	0	0	0	0	0	0	0	0	0	0	0	0	0	0	0	0	0	0	0	0	0	0	0	0	0	0	0	0	0	0	0	0	0	0	2	42.01566667	20.8855
420	2004/059		0	0	0	0	0	0	0	0	1	0	0	0	0	0	0	0	0	0	0	0	0	0	0	0	0	0	0	0	0	0	0	0	0	0	0	0	0	0	1	0	0	0	0	0	0	0	0	0	0	0	0	0	0	0	0	0	0	0	0	0	0	0	0	0	0	0	0	0	0	3	42.01133333	20.90233333
421	2004/060		1	0	0	0	0	1	0	0	1	0	0	0	1	0	1	1	0	0	1	0	0	0	0	0	0	1	0	0	0	0	0	0	1	0	0	0	0	0	1	0	0	0	0	0	0	0	0	0	0	0	0	0	0	0	1	0	0	0	0	0	0	0	0	0	0	0	0	0	0	1	41.961	21.29433333
422	2004/061		0	0	0	0	0	1	0	0	1	0	1	0	1	0	0	0	0	0	0	0	0	1	0	1	0	0	1	0	0	0	0	0	0	0	0	0	0	0	0	0	0	0	0	0	0	0	0	0	0	0	0	0	0	0	0	0	0	0	0	0	0	0	0	0	0	0	0	0	0	1	43.85766667	21.6145
424		0	0	0	0	0	0	1	0	0	0	0	0	0	0	1	1	0	0	0	0	0	0	1	0	0	0	0	0	0	0	0	0	0	0	0	0	0	0	0	0	0	0	0	0	0	0	0	0	0	0	0	1	1	0	0	0	0	0	1	0	0	0	0	0	0	0	0	0	0	0	1	40.69865	20.096633
425	2004/075		1	0	0	0	0	0	0	0	1	1	0	0	1	0	0	1	0	0	1	0	0	1	1	0	0	1	0	0	0	0	0	0	1	1	0	0	0	0	0	0	0	0	0	0	0	0	0	0	0	0	0	0	0	0	0	0	1	0	0	0	0	0	0	0	0	0	0	0	0	1	40.55355	20.27832
426	2004/076		1	0	0	0	0	1	0	0	0	0	0	0	0	0	0	0	0	0	0	0	0	1	0	0	0	0	0	0	0	0	0	0	0	1	0	0	0	0	0	0	0	0	0	0	0	0	0	0	0	0	0	1	0	0	0	0	0	0	0	0	0	0	0	0	0	0	0	0	0	1	40.55387	20.27148
427	2004/077		1	0	0	0	0	1	0	0	0	0	0	0	0	0	0	0	0	0	0	0	0	0	0	0	0	0	0	0	0	0	0	0	0	0	0	0	0	0	0	0	0	0	0	0	0	0	0	0	0	0	0	0	0	0	0	0	0	0	0	0	0	0	0	0	0	0	0	0	0	2	40.56024	20.26453
428	2004/078		0	0	0	0	0	1	0	0	0	0	0	0	0	0	0	0	0	0	0	0	0	1	0	0	0	0	0	0	0	0	0	0	0	0	0	0	0	0	0	0	0	0	0	0	0	0	0	0	0	0	0	0	0	0	0	0	0	0	0	0	0	0	0	0	0	0	0	0	0	1	40.56464	20.26193
429	2004/079		1	0	0	0	0	1	0	0	1	1	0	0	1	0	1	1	0	0	1	0	0	0	0	1	0	1	0	0	0	0	0	0	0	0	0	0	0	0	0	0	0	0	0	0	0	0	0	0	0	0	0	1	0	0	0	0	0	0	0	0	0	0	0	0	0	1	0	0	0	1	40.56856	20.25854
430	2004/080		0	0	0	0	0	1	0	0	0	0	0	0	0	0	1	0	0	0	0	0	0	0	0	1	0	0	0	0	0	0	0	0	0	0	0	0	0	0	0	0	0	0	0	0	0	0	0	0	0	0	0	1	0	0	0	0	0	0	0	0	0	0	0	0	0	1	0	0	0	1	40.57754	20.25825
431	2004/081		0	0	0	0	0	0	0	0	0	0	0	0	1	0	1	0	0	0	0	0	0	0	0	0	0	0	0	0	0	0	0	0	0	0	0	0	0	0	0	0	0	0	0	0	0	0	0	0	0	0	0	1	0	0	0	0	0	0	0	0	0	0	0	0	0	0	0	0	0	1	40.58657	20.24382
432	2004/082		0	0	0	0	0	0	0	0	0	0	0	0	1	0	0	0	0	0	0	0	0	0	0	0	0	0	0	0	0	0	0	0	0	0	0	0	0	0	0	0	0	0	0	0	0	0	0	0	0	0	0	0	0	0	0	0	0	0	0	0	0	0	0	0	0	1	0	0	0	1	40.58961	20.24137
433	2004/083		0	0	0	0	0	0	0	0	0	0	0	0	1	0	1	1	0	0	1	0	0	0	0	0	0	0	0	0	0	0	0	0	0	0	0	0	0	0	0	0	0	0	0	0	0	0	0	0	0	0	0	0	0	0	0	0	0	0	0	0	0	0	0	0	0	1	0	0	0	1	40.59128	20.23847
434	2004/084		1	0	0	0	0	0	0	0	1	0	0	0	1	0	0	0	0	0	0	0	0	0	0	0	0	1	0	0	0	0	1	0	0	0	0	0	0	0	0	0	0	0	0	0	0	0	0	0	0	0	0	1	0	0	0	0	0	0	0	0	0	0	0	0	0	1	0	0	0	1	40.59518	20.22139
435	2004/085		0	0	0	0	0	1	0	0	0	0	0	0	0	0	0	0	0	0	0	0	0	0	0	0	0	0	0	0	0	0	0	0	0	0	0	0	0	0	0	0	0	0	0	0	0	0	0	0	0	0	0	0	0	0	0	0	0	0	0	0	0	0	0	0	0	0	0	0	0	2	40.59848	20.21548
436	2004/086		0	0	0	0	0	0	0	0	0	0	0	0	1	0	0	0	0	0	0	0	0	0	0	0	0	0	0	0	0	0	0	0	0	0	0	0	0	0	0	0	0	0	0	0	0	0	0	0	0	0	0	0	0	0	0	0	0	0	0	0	0	0	0	0	0	0	0	0	0	1	40.6248	20.1742
437	2004/087		0	0	0	0	0	0	0	0	0	0	0	0	0	0	0	0	0	0	0	0	0	0	0	0	0	0	0	0	0	0	0	0	0	0	0	0	0	0	0	0	0	0	0	0	0	0	0	0	0	0	0	0	0	0	0	0	0	0	0	0	0	0	0	0	0	1	0	0	0	1	40.63063	20.16619
439	2004/089		1	0	0	0	0	0	0	0	0	0	0	0	1	0	1	0	0	0	0	0	0	0	0	1	0	1	0	0	0	0	0	0	0	0	0	0	0	0	0	0	0	0	0	0	0	0	0	0	0	0	0	0	0	0	0	0	0	0	0	0	0	0	0	0	0	1	0	0	0	2	40.64401	20.15902
440	2004/090		1	0	0	0	0	1	0	0	0	0	0	0	0	0	0	0	0	0	0	0	0	0	0	1	0	0	0	0	0	0	0	0	0	0	0	0	0	0	0	0	0	0	0	0	0	0	0	0	0	0	0	0	0	0	0	0	0	0	0	0	0	0	0	0	0	0	0	0	0	1	40.65156	20.15729
441	2004/091		1	0	0	0	0	1	0	0	0	0	0	0	1	1	0	0	0	0	0	0	0	0	0	0	0	0	0	0	0	0	0	0	0	0	0	0	0	0	0	0	0	0	0	0	0	0	0	0	0	0	0	1	0	0	0	0	0	0	0	0	0	0	0	0	0	0	0	0	0	1	40.65334	20.16117
442	2004/092		0	0	0	0	0	1	0	0	0	0	0	0	1	0	0	0	0	0	0	0	0	1	0	0	0	0	0	0	0	0	0	0	0	0	0	0	0	0	0	0	0	0	0	0	0	0	0	0	0	0	0	0	0	0	0	0	0	0	0	0	0	0	0	0	0	0	0	0	0	1	40.6745	20.15773
443	2004/093		1	0	0	0	0	1	0	0	0	0	0	0	1	0	0	0	0	0	0	0	0	0	0	1	0	0	0	0	0	0	0	0	0	0	0	0	0	0	0	0	0	0	0	0	0	0	0	0	0	0	0	0	0	0	0	0	0	0	0	0	0	0	0	0	0	1	0	0	0	1	40.68722	20.15067
444	2004/094		1	0	0	0	0	1	0	0	0	0	0	0	1	0	0	0	0	0	0	0	0	0	0	0	0	0	0	0	0	0	1	0	0	0	0	0	0	0	0	0	0	0	0	0	0	0	0	0	0	0	0	0	0	0	0	0	0	0	0	0	0	0	0	0	0	1	0	0	0	1	40.68886	20.14611
445	2004/095		0	0	0	0	0	1	0	0	0	0	0	0	0	0	1	0	0	0	0	0	0	0	0	1	0	0	0	0	0	0	0	0	0	0	0	0	0	0	0	0	0	0	0	0	0	0	0	0	0	0	0	0	0	0	0	0	0	0	0	0	0	0	0	0	0	0	0	0	0	2	40.69866	20.14496
446	2004/096		0	0	0	0	0	1	0	0	1	0	0	0	0	0	0	1	0	0	1	0	0	0	0	0	0	0	0	0	0	0	0	1	0	0	0	0	0	0	0	0	0	0	0	0	0	0	0	0	0	0	0	1	1	0	0	0	0	0	0	0	0	0	0	0	0	1	0	0	0	3	40.68797	20.13987
447	2004/097		0	0	0	0	0	0	0	0	0	0	0	0	0	1	0	0	0	0	0	0	0	0	0	0	0	0	0	0	0	0	0	0	0	0	0	0	0	0	0	0	0	0	0	0	0	0	0	0	0	0	0	0	0	0	0	0	0	0	0	0	0	0	0	0	0	1	0	0	0	3	40.68832	20.1341
448	2004/098		0	0	0	0	0	1	0	0	0	0	0	0	0	0	0	0	0	0	0	0	0	1	0	0	0	0	0	0	0	0	0	0	0	0	0	0	0	0	0	0	0	0	0	0	0	0	0	0	0	0	0	0	0	0	0	0	0	0	0	0	0	0	0	0	0	0	0	0	0	1	40.68779	20.12874
450	2004/100		1	0	0	0	0	0	0	0	0	0	0	0	1	0	1	0	0	0	0	0	0	1	0	0	0	0	0	0	0	0	1	1	0	0	0	0	0	0	0	0	0	0	0	0	0	0	0	0	0	0	0	1	0	0	0	0	0	0	0	0	0	0	0	0	0	1	0	0	0	1	40.67884	20.11085
451	2004/101		1	0	0	0	0	0	0	0	0	0	0	0	0	0	0	0	0	0	0	0	0	0	0	0	0	0	0	0	0	0	0	0	0	0	0	0	0	0	0	0	0	0	0	0	0	0	0	0	0	0	1	0	0	0	0	0	0	0	0	0	0	0	0	0	0	0	0	0	0	1	40.21584	19.60634
452	2004/102		1	0	0	0	0	1	0	0	0	0	0	0	1	0	0	0	0	0	0	0	0	0	0	0	0	0	0	0	0	0	0	0	0	0	0	0	0	0	0	0	0	0	0	0	0	0	0	0	0	0	1	0	0	0	0	0	0	0	0	0	0	0	0	0	0	0	0	0	0	2	40.21383	19.61098
453	2004/103		0	0	0	0	0	0	0	0	0	0	0	0	0	0	0	0	0	0	0	0	0	0	0	0	0	0	0	0	0	0	0	0	0	0	0	0	0	0	0	0	0	0	0	0	0	0	0	0	0	0	0	0	0	0	0	0	0	0	0	0	0	0	0	0	0	0	0	0	0	2	40.20966	19.61786
454	2004/104		1	0	0	0	0	1	0	0	0	0	0	0	0	0	0	0	0	0	0	0	0	0	0	0	0	0	0	0	0	0	0	0	0	0	0	0	0	0	0	0	0	0	0	0	0	0	0	0	0	0	0	0	0	0	0	0	0	0	0	0	0	0	0	0	0	0	0	0	0	1	40.19852	19.59163
456	2004/106		1	0	0	0	0	0	0	0	0	0	0	0	1	0	0	0	0	0	0	0	0	0	0	0	0	0	0	0	0	0	0	0	0	0	0	0	0	0	0	1	0	0	0	0	0	0	0	0	0	0	0	0	0	0	0	0	0	0	0	0	0	0	0	0	0	0	0	0	0	1	42.66346	19.37159
459	2004/109		1	0	0	0	0	0	0	0	0	0	0	0	0	0	1	0	0	0	0	0	0	1	0	0	0	0	0	0	0	0	1	0	0	0	0	0	0	0	0	0	0	0	0	0	0	0	0	0	0	1	0	0	1	0	0	0	0	0	0	0	0	0	0	0	0	0	0	0	0	1	41.5065	19.92183333
466	2004/115		0	0	0	0	0	1	0	0	0	0	0	0	0	1	0	0	0	0	0	0	0	0	0	0	0	0	0	0	0	0	0	0	0	0	0	0	0	0	0	0	0	0	0	0	0	0	0	0	0	0	0	0	0	0	0	0	0	0	0	0	0	0	1	0	0	0	0	0	0	1	41.47083333	20.07783333
472	2004/121		1	0	0	0	0	1	0	0	0	0	0	0	1	0	1	0	0	0	0	0	0	0	0	1	0	0	0	0	0	0	1	0	1	0	0	0	0	0	0	0	0	0	0	0	0	0	0	0	0	1	0	0	0	0	0	0	0	0	0	0	0	0	0	0	0	0	0	0	1	1	41.35147222	20.11627778
475	2004/124		0	0	0	0	0	0	0	0	0	0	0	0	1	0	1	0	0	0	0	0	0	0	0	0	0	0	0	0	0	0	0	0	0	0	0	0	0	0	0	0	0	0	0	0	0	0	0	0	0	0	0	1	0	0	0	0	0	0	0	0	0	0	0	0	0	0	0	0	0	1	40.516	20.24263889
476	2004/125		1	0	1	0	0	0	0	0	1	1	0	0	0	1	1	0	0	0	0	0	0	1	0	0	0	0	0	0	0	0	1	0	0	1	0	0	0	0	0	0	0	0	0	0	0	0	0	0	0	0	1	1	0	0	0	0	1	0	0	0	0	0	0	0	0	0	0	0	0	1	40.5235	20.25311111
480	2004/128		1	0	0	0	0	0	0	0	1	0	0	0	1	1	1	0	0	0	0	0	0	1	0	0	0	0	0	0	0	0	0	0	0	0	0	0	0	0	0	0	0	0	0	0	0	0	0	0	0	0	1	1	0	0	0	0	0	0	0	0	0	0	0	1	0	0	0	0	0	1	40.53561111	19.79638889
481	2004/130		1	0	0	0	0	0	0	0	0	0	0	0	1	0	1	0	0	0	0	0	0	1	0	0	0	1	0	0	0	0	0	0	0	0	0	0	0	0	0	0	0	0	0	0	0	0	0	0	0	0	1	0	0	0	0	0	0	0	0	0	0	0	0	0	0	0	0	0	0	1	40.24916667	19.99397222
482	2004/131		0	0	0	0	0	0	0	0	0	0	0	0	0	0	0	0	0	0	0	0	0	0	0	0	0	0	0	0	0	0	0	0	0	0	0	0	0	0	0	0	0	0	0	0	0	0	0	0	0	0	0	0	0	0	0	0	0	0	0	0	0	0	0	0	0	0	0	0	0	2	40.24077778	19.96152778
483	2004/132		1	0	0	0	0	0	0	0	1	1	0	0	1	0	1	1	0	0	1	0	0	0	0	0	0	1	0	0	0	0	0	0	0	0	0	0	0	0	0	0	0	0	0	0	0	0	0	0	0	0	1	1	0	0	0	0	0	0	0	0	0	0	0	0	0	0	0	0	0	1	40.21022222	19.96141667
484	2004/133		1	0	1	0	0	1	0	0	1	1	0	0	0	0	1	0	0	0	0	0	0	1	0	0	0	1	0	0	0	0	0	0	0	0	0	0	0	0	0	0	0	0	0	0	0	0	0	0	0	0	1	0	0	0	0	0	0	0	0	0	0	0	0	0	0	0	0	0	0	2	40.2135	19.93541667
485	2004/134		1	0	0	0	0	0	0	0	1	0	0	0	0	0	1	0	0	0	0	0	0	1	0	0	0	1	0	0	0	0	0	0	0	0	0	0	0	0	0	0	0	0	0	0	0	0	0	0	0	0	1	0	0	0	0	0	0	0	0	0	0	0	0	0	0	0	0	0	0	1	40.21297222	19.93052778
486	2004/135		1	0	0	0	0	0	0	0	0	0	0	0	0	0	0	0	0	0	0	0	0	1	0	0	0	0	0	0	0	0	0	0	0	0	0	0	0	0	0	0	0	0	0	0	0	0	0	0	0	0	0	0	0	0	0	0	0	0	0	0	0	0	0	0	0	0	0	0	0	1	40.26347222	20.00708333
487	2004/136		1	0	0	0	0	0	0	0	0	0	0	0	0	1	1	0	0	0	0	0	0	0	0	0	0	0	0	0	0	0	1	0	0	0	0	0	0	0	0	0	0	0	0	0	0	0	0	0	0	0	1	0	0	0	0	0	0	0	0	0	0	0	0	0	0	0	0	0	0	1	40.29380556	20.00875
489	2004/138		1	0	0	0	0	0	0	0	1	0	0	0	0	1	0	0	0	0	0	0	0	1	0	0	0	0	0	0	0	0	1	0	0	0	1	0	0	0	0	0	0	0	0	0	0	0	0	0	0	0	0	0	0	0	0	0	1	0	0	0	0	0	0	0	0	0	0	0	0	1	40.08827778	20.27166667
490	2004/139		1	0	0	0	0	0	0	0	1	0	0	0	1	0	0	0	0	0	0	0	0	0	0	0	0	0	0	0	0	0	1	0	0	0	1	0	0	0	0	0	0	0	0	0	0	0	0	0	0	0	0	0	0	0	0	0	1	0	0	0	0	0	0	0	0	0	0	0	0	1	40.09147222	20.29219444
491	2004/140		1	0	0	0	0	0	0	0	0	0	0	0	0	0	0	0	0	0	0	0	0	0	0	0	0	0	0	0	0	0	0	0	0	0	1	0	0	0	0	0	0	0	0	0	0	0	0	0	0	0	0	1	0	0	0	0	0	0	0	0	0	0	0	0	0	0	0	0	0	1	40.11641667	20.32941667
492	2004/141		1	0	0	0	0	0	0	0	0	0	0	0	0	0	0	0	0	0	0	0	0	1	0	0	0	0	0	0	0	0	0	0	0	0	1	0	0	0	0	0	0	0	0	0	0	0	0	0	0	0	0	1	0	0	0	0	0	0	0	0	0	0	0	0	0	0	0	0	0	1	40.17011111	20.31511111
493	2004/142		1	0	0	0	0	1	0	0	0	0	0	0	0	0	0	0	0	0	0	0	0	1	0	0	0	1	0	0	0	0	0	0	0	0	1	0	0	0	0	0	0	0	0	0	0	0	0	0	0	0	0	1	0	0	0	0	0	0	0	0	0	0	0	0	0	0	0	0	0	1	40.18558333	20.33236111
495	2004/144		1	0	0	0	0	0	0	0	1	1	0	0	0	0	1	0	0	0	0	0	0	0	0	0	0	0	0	0	0	0	1	0	0	0	0	0	0	0	0	0	0	0	0	0	0	0	0	0	0	0	1	1	0	0	0	0	0	0	0	0	0	0	0	0	0	0	0	0	0	1	40.07444444	20.14063889
496	2004/145		1	0	0	0	0	0	0	0	0	0	0	0	1	0	1	0	0	0	0	0	0	0	0	0	0	0	0	0	0	0	0	0	0	0	0	0	0	0	0	0	0	0	0	0	0	0	0	0	0	0	1	1	0	0	0	0	0	0	0	0	0	0	0	0	0	0	0	0	0	3	39.92461111	20.1925
499	2004/147		1	0	0	0	0	0	0	0	0	0	0	1	1	1	1	0	0	0	0	0	0	1	0	0	0	0	0	0	0	0	1	0	0	0	0	0	0	0	0	0	0	0	0	0	0	0	0	0	0	0	1	0	0	0	0	0	0	0	0	0	0	0	0	0	0	0	0	0	0	1	40.05277778	19.81280556
500	2004/148		1	0	0	0	0	0	0	0	0	0	0	0	0	0	0	0	0	0	0	0	0	1	0	0	0	0	0	0	0	0	1	0	0	0	1	0	0	0	0	0	0	0	0	0	0	0	0	0	0	0	1	0	0	0	0	0	0	0	0	0	0	0	0	0	0	0	0	0	0	1	40.15577778	19.63936111
501	2004/149		0	0	0	0	0	0	0	0	0	0	0	0	0	0	0	0	0	0	0	0	0	0	0	0	0	0	0	0	0	0	1	0	1	0	0	0	0	0	0	1	0	0	0	0	0	0	0	0	0	0	0	0	0	0	0	0	0	0	0	0	0	0	0	0	0	0	0	0	0	1	42.74833333	19.40666667
502	2004/150		0	0	0	0	0	0	0	0	0	0	0	0	0	0	1	0	0	0	0	0	0	0	0	0	0	0	0	0	0	0	0	0	0	0	0	0	0	0	0	0	0	0	0	0	0	0	0	0	0	0	0	0	0	0	0	0	0	0	0	0	0	0	0	0	0	0	0	0	0	1	43.29027778	19.714
506	2005/B02		1	0	0	0	0	0	0	0	0	0	0	0	0	0	0	0	0	0	0	0	0	1	0	0	0	0	0	0	0	0	0	0	0	0	0	0	0	0	0	0	0	0	0	0	0	0	0	0	0	0	0	0	0	0	0	0	0	0	0	0	0	0	0	0	0	0	0	0	0	1	40.26658	20.00596
507	2005/B03		1	0	0	0	0	1	0	0	0	0	0	0	0	0	0	0	0	0	0	0	0	0	0	0	0	0	0	0	0	0	0	0	0	0	0	0	0	0	0	0	0	0	0	0	0	0	0	0	0	0	0	0	0	0	0	0	0	0	0	0	0	0	0	0	0	0	0	0	0	1	40.26201	19.98091
508	2005/B04		1	0	0	0	0	1	0	0	1	0	0	0	0	0	0	0	0	0	0	0	0	0	0	0	0	0	0	0	0	0	0	0	0	0	0	0	0	0	0	0	0	0	0	0	0	0	0	0	0	0	0	0	0	0	0	0	0	0	0	0	0	0	0	0	0	0	0	0	0	1	40.26385	19.9704
509	2005/B05		1	0	0	0	0	0	0	0	1	0	0	0	1	0	0	0	0	0	0	0	0	0	0	0	0	0	0	0	0	0	0	0	0	0	0	0	0	0	0	0	0	0	0	0	0	0	0	0	0	0	0	0	0	0	0	0	0	0	0	0	0	0	0	0	0	0	0	0	0	1	40.26494	19.95902
510	2005/B06		1	0	0	0	0	0	0	0	0	0	0	0	0	0	0	0	0	0	0	0	0	0	0	0	0	0	0	0	0	0	0	0	0	0	0	0	0	0	0	0	0	0	0	0	0	0	0	0	0	0	0	0	0	0	0	0	0	0	0	0	0	0	0	0	0	0	0	0	0	1	40.27073	19.92616
511	2005/B07		1	0	0	0	0	0	0	0	0	0	0	0	0	0	0	0	0	0	0	0	0	0	0	0	0	1	0	0	0	0	0	0	0	0	0	0	0	0	0	0	0	0	0	0	0	0	0	0	0	0	0	1	0	0	0	0	0	0	0	0	0	0	0	0	0	0	0	0	0	1	40.27602	19.9013
512	2005/B08		0	0	0	0	0	1	0	0	0	0	0	0	0	0	1	0	0	0	0	0	0	1	0	0	0	0	0	0	0	0	0	0	0	0	0	0	0	0	0	0	0	0	0	0	0	0	0	0	0	0	0	0	0	0	0	0	0	0	0	0	0	0	0	0	0	0	0	0	0	1	40.27822	19.87347
513	2005/B09		1	0	0	0	0	0	0	0	0	0	0	0	0	0	1	0	0	0	0	0	0	0	0	0	0	0	0	0	0	0	0	0	0	0	1	0	0	0	0	0	0	0	0	0	0	0	0	0	0	0	0	0	0	0	0	0	0	0	0	0	0	0	0	0	0	0	0	0	0	1	40.2843	19.85666
514	2005/B10		1	0	0	0	0	0	0	0	1	0	0	0	1	0	1	0	0	0	0	0	0	0	0	0	0	0	0	0	0	0	0	0	0	0	0	0	0	0	0	0	0	0	0	0	0	0	0	0	0	0	0	0	0	0	0	0	0	0	0	0	0	0	0	0	0	0	0	0	0	1	40.28706	19.8528
515	2005/B10B		0	0	0	0	0	0	0	0	0	0	0	0	0	0	0	0	0	0	0	0	0	0	0	0	0	1	0	0	0	0	0	0	0	0	0	0	0	0	0	0	0	0	0	0	0	0	0	0	0	0	0	0	0	0	0	0	0	0	0	0	0	0	0	0	0	0	0	0	0	1	40.28698	19.8614
517		0	1	0	0	0	0	0	0	0	0	0	0	0	0	0	0	0	0	0	0	0	0	0	0	0	0	0	0	0	0	0	0	0	0	0	0	0	0	0	0	0	0	1	0	0	0	1	0	0	0	0	0	0	0	0	0	0	0	0	0	0	0	0	0	0	0	0	0	0	0	1	42.44313	19.76374
518		0	0	0	0	0	0	0	0	0	0	0	0	0	0	0	0	0	0	0	0	0	0	0	0	0	0	0	0	0	0	0	0	0	0	0	0	0	0	0	0	1	0	0	0	0	0	0	0	0	0	0	0	0	0	0	0	0	0	0	0	0	0	0	0	0	0	0	0	0	0	1	42.40623	19.80768
520	2005/O02		0	0	0	0	0	0	0	0	0	0	0	0	0	0	0	0	0	0	0	0	0	1	0	0	0	0	0	0	0	0	0	0	0	0	0	0	0	0	0	0	0	0	0	0	0	0	0	0	0	0	0	0	0	0	0	0	0	0	0	0	0	0	0	0	0	0	0	0	0	1	40.51995	20.41771
522	2005/O04		0	0	0	0	0	1	0	0	0	0	0	0	0	0	0	0	0	0	0	0	0	0	0	0	0	0	0	0	0	0	0	0	0	0	0	0	0	0	0	0	0	0	0	0	0	0	0	0	0	0	0	0	0	0	0	0	0	0	0	0	0	0	0	0	0	0	0	0	0	1	40.52602	20.41528
524	2005/O13		0	0	0	0	0	0	0	0	0	1	0	0	0	0	1	0	0	0	0	0	0	0	0	0	0	1	0	0	0	0	0	0	0	0	0	0	0	0	0	0	0	0	0	0	0	0	0	0	0	0	0	0	0	0	0	0	0	0	0	0	0	0	0	0	0	0	0	0	0	1	40.57649	20.44733
525	2005/O15		1	0	0	0	0	0	0	0	0	0	0	0	0	0	0	0	0	0	0	0	0	1	0	0	0	1	0	0	0	0	0	0	0	1	0	0	0	0	0	0	0	0	0	0	0	0	0	0	0	0	0	0	0	0	0	0	0	0	0	0	0	0	0	0	0	0	0	0	0	1	40.56065	20.39377
528	2005/043		0	0	0	0	0	1	0	0	0	0	0	0	1	0	1	0	0	0	0	0	0	1	0	0	0	0	0	0	0	0	0	0	0	0	0	0	0	0	0	1	0	0	0	0	0	0	0	0	0	0	0	0	0	0	0	0	0	0	0	0	0	0	0	0	0	0	0	0	0	1	42.55023333	19.82476667
529	2005/044		0	0	0	0	0	1	0	0	0	0	0	0	1	0	0	0	0	0	0	0	0	1	0	0	0	0	0	0	0	0	0	0	0	0	0	0	0	0	1	0	0	0	0	0	0	0	0	0	0	0	0	0	0	0	0	0	0	0	0	0	0	0	0	0	0	0	0	0	0	1	42.57818333	19.81493333
532	2005/047		1	0	0	0	0	1	0	0	1	0	0	0	1	1	1	0	1	0	0	1	0	1	0	1	0	1	0	0	0	0	0	1	1	0	0	0	0	0	1	0	0	1	0	0	0	1	0	0	0	0	0	0	0	0	0	0	0	0	0	0	0	0	0	0	0	0	0	0	0	1	42.57208333	19.73991667
534	2005/049		1	0	0	0	0	1	0	0	1	1	0	0	1	0	1	1	0	0	1	0	0	1	0	0	0	0	0	0	0	0	0	0	0	0	0	0	0	0	1	1	0	1	0	0	0	1	0	0	0	0	0	0	0	0	0	0	0	0	0	0	0	0	0	0	0	0	0	0	0	1	42.51563333	19.73713333
540	2005/054		1	0	0	0	0	1	0	0	1	0	0	0	1	0	1	1	0	0	1	0	0	1	0	0	0	0	1	0	0	0	0	1	0	0	0	0	0	1	0	0	1	0	0	0	0	1	0	0	0	0	0	0	0	0	0	0	0	0	0	0	0	0	0	0	0	0	0	0	0	1	42.68468333	20.05356667
541	2005/055		1	0	0	0	0	1	0	0	1	0	0	0	1	0	0	0	0	0	0	0	0	1	0	1	0	0	0	0	0	0	0	0	0	0	0	0	0	0	0	0	0	0	0	0	0	0	0	0	0	0	0	0	0	0	0	0	0	0	0	0	0	0	0	0	0	0	0	0	0	1	42.7723	20.27901667
544	2005/058		0	0	0	0	0	1	0	0	0	0	0	0	1	0	0	0	0	0	0	0	0	0	0	0	0	0	0	0	0	0	0	0	0	0	0	0	0	0	0	1	0	0	0	0	0	0	0	0	0	0	0	0	0	0	0	0	0	0	0	0	0	0	0	0	0	0	0	0	0	1	42.38841667	20.0729
545	2005/059		1	0	0	0	0	1	0	0	0	0	0	0	1	0	0	0	0	0	0	0	0	1	0	0	0	0	0	0	0	0	0	1	0	0	0	0	0	0	0	1	0	0	0	0	0	0	0	0	0	0	0	0	0	0	0	0	0	0	0	0	0	0	0	0	0	0	0	0	0	1	42.41638333	20.01271667
546	2005/060		0	0	0	0	0	1	0	0	1	1	0	0	1	0	1	0	0	0	0	0	0	1	0	0	0	1	0	0	0	0	0	0	0	0	0	0	0	0	0	1	0	0	0	0	0	0	0	0	0	0	0	0	0	1	0	0	0	0	0	0	0	0	0	0	0	0	0	0	0	1	42.4364	19.98465
547	2005/061		0	0	0	0	0	0	0	0	0	0	0	0	1	0	0	0	0	0	0	0	0	0	0	0	0	0	0	0	0	0	0	0	0	0	0	0	0	0	0	0	0	0	0	0	0	0	0	0	0	0	0	0	0	0	0	0	0	0	0	0	0	0	0	0	0	0	0	0	0	1	42.41033333	19.82276667
548	2005/062		0	0	0	0	0	0	0	0	0	0	0	0	1	0	0	0	0	0	0	0	0	0	0	0	0	0	0	0	0	0	0	0	0	0	0	0	0	0	0	1	0	0	0	0	0	0	0	0	0	0	0	0	0	0	0	0	0	0	0	0	0	0	0	0	0	0	0	0	0	1	42.41135	19.81475
549	2005/063		1	0	0	0	0	0	0	0	0	0	0	0	1	0	0	0	0	0	0	0	0	0	0	0	0	0	0	0	0	0	0	0	0	0	0	0	0	0	0	1	0	0	0	0	0	0	0	0	0	0	0	0	0	1	0	0	0	0	0	0	0	0	0	0	0	0	0	0	0	1	42.40678333	19.8122
552	2005/065		1	0	0	0	0	0	0	0	0	1	0	0	0	0	1	0	0	0	0	0	0	1	0	0	0	1	0	0	0	0	1	0	0	0	0	0	0	0	0	1	0	0	0	0	0	0	0	0	0	0	0	0	0	0	0	0	0	0	0	0	0	0	0	0	0	0	0	0	0	1	42.29878333	20.02885
553	2005/066		1	0	0	0	0	0	0	0	1	1	0	0	1	1	1	0	0	1	0	0	1	1	0	0	0	0	0	0	0	0	1	0	0	0	0	0	0	0	0	1	0	0	0	0	0	0	0	0	0	0	0	1	0	0	0	0	0	0	0	0	0	0	0	0	0	0	0	0	0	1	42.28676667	20.02145
554	2005/067		1	0	0	0	0	0	0	0	1	1	0	0	1	0	1	0	0	0	0	0	0	1	0	0	0	1	0	0	0	0	1	0	0	1	0	0	0	0	0	1	0	0	0	0	0	0	0	0	0	0	0	0	0	1	0	0	0	0	0	0	0	0	0	0	0	0	0	0	0	1	42.26306667	19.99
555	2005/068		0	0	0	0	0	0	0	0	0	0	0	0	1	0	0	0	0	0	0	0	0	0	0	0	0	0	0	0	0	0	0	0	0	0	0	0	0	0	0	0	0	0	0	0	0	0	0	0	0	0	0	0	0	0	0	0	0	0	0	0	0	0	0	0	0	0	0	0	0	1	42.2678	19.94943333
557	2005/070		1	0	0	0	0	1	0	0	0	1	0	0	1	0	1	0	0	0	0	0	0	1	0	0	0	1	0	0	0	0	0	0	0	1	0	0	0	0	0	0	0	0	0	0	0	0	0	0	0	0	0	1	0	0	0	0	0	0	0	0	0	0	0	0	0	0	0	0	0	1	42.22025	20.09765
561	2005/074		0	0	0	0	0	1	0	0	1	0	0	0	1	1	1	0	0	0	0	0	0	0	0	0	0	0	0	0	0	0	0	0	0	0	0	0	0	0	1	0	0	0	0	0	0	0	0	0	0	0	0	0	0	0	0	0	0	0	0	0	0	0	0	0	0	0	0	0	0	1	41.94721667	20.27955
562	2005/075		0	0	0	0	0	1	0	0	0	0	0	0	1	1	1	0	0	0	0	0	0	1	0	0	0	0	0	0	0	0	1	0	0	0	0	0	0	0	0	0	0	0	0	0	0	0	0	0	0	0	0	0	0	0	0	0	0	0	0	0	0	0	0	0	0	0	0	0	1	1	41.92416667	20.2813
563	2005/076		1	0	0	0	0	0	0	0	0	1	0	0	1	0	1	0	0	1	0	0	1	1	0	1	0	1	0	0	1	0	0	0	0	0	0	0	0	0	1	0	0	0	0	0	0	0	0	0	0	0	0	1	1	0	0	0	0	0	0	0	0	0	0	0	0	0	0	0	0	1	41.9163	20.2526
564	2005/077		1	0	0	0	0	0	0	0	1	1	0	0	1	1	1	1	1	0	1	1	0	1	0	1	0	1	0	0	0	0	1	0	0	0	0	0	0	0	0	0	0	0	0	0	0	0	0	0	0	0	0	1	0	0	0	0	0	0	0	0	0	0	0	0	0	0	0	0	1	1	41.91881667	20.26928333
568	2005/081		0	0	0	0	0	0	0	0	0	0	0	0	0	0	0	0	0	0	0	0	0	0	0	0	0	0	0	0	0	0	0	0	0	0	0	0	0	0	0	0	0	0	0	0	0	0	0	0	0	0	0	0	0	0	0	0	0	0	0	0	0	0	0	0	0	0	0	0	0	1	41.94046667	20.37601667
569	2005/082		0	0	0	0	0	1	0	0	1	1	0	0	1	0	1	0	0	0	0	0	0	1	0	0	0	0	0	0	0	0	1	0	0	0	0	0	0	0	0	0	0	0	0	0	0	0	0	0	0	0	0	0	0	0	0	0	0	0	0	0	0	0	0	0	0	0	0	0	1	1	41.93515	20.3632
570	2005/083		0	0	0	0	0	1	0	0	0	0	0	0	0	0	1	0	0	0	0	0	0	1	0	0	0	1	0	0	0	0	0	0	0	0	0	0	0	0	0	0	0	0	0	0	0	0	0	0	0	0	0	0	0	0	0	0	0	0	0	0	0	0	0	0	0	0	0	0	0	1	41.92371667	20.35395
571	2005/084		1	0	0	0	0	1	0	0	0	0	0	0	1	1	1	0	0	0	0	0	0	1	0	0	0	0	0	0	0	0	1	0	0	0	0	0	0	0	0	0	0	0	0	0	0	0	0	0	0	0	0	0	0	0	0	0	0	0	0	0	0	0	0	0	0	0	0	0	1	1	41.94231667	20.35933333
572	2005/085		0	0	0	0	0	0	0	0	0	0	0	0	1	0	1	0	0	0	0	0	0	1	0	0	0	0	0	0	0	0	0	0	0	0	0	0	0	0	0	0	0	0	0	0	0	0	0	0	0	0	0	1	0	0	0	0	0	0	0	0	0	0	0	0	0	0	0	0	0	1	41.90813333	20.35825
573	2005/086		1	0	0	0	0	1	0	0	0	0	0	0	1	1	1	0	0	0	0	0	0	0	0	0	0	0	0	0	0	0	1	0	0	0	0	0	0	0	0	0	0	0	0	0	0	0	0	0	0	0	1	0	0	0	0	0	0	0	0	0	0	0	0	0	0	0	0	0	1	1	41.89078333	20.337
574	2005/087		1	0	0	0	0	1	0	0	1	0	0	0	1	1	1	0	0	0	0	0	0	1	0	0	0	0	0	0	0	0	1	0	0	0	0	0	0	0	0	0	0	0	0	0	0	0	0	0	0	0	0	0	0	0	0	0	0	0	0	0	0	0	0	0	0	0	0	0	1	1	41.88803333	20.31651667
576	2005/089		1	0	0	0	0	1	0	0	0	1	0	0	1	1	1	0	0	0	0	0	0	0	0	0	0	0	0	0	0	0	1	0	0	0	0	0	0	0	0	0	0	0	0	0	0	0	0	0	0	0	0	1	0	0	0	1	0	0	0	0	0	0	0	0	0	0	0	0	0	1	41.73908333	20.29681667
578	2005/090A		1	0	0	0	0	1	0	0	0	0	0	0	1	0	1	0	0	1	0	0	1	1	0	0	0	0	0	0	0	0	0	0	0	0	0	0	0	0	0	0	0	0	0	0	0	0	0	0	0	0	0	0	0	0	0	1	0	0	0	0	0	0	0	0	0	0	0	0	0	1	41.75248333	20.24553333
579	2005/090B		0	0	0	0	0	1	0	0	0	1	0	0	1	1	0	0	0	0	0	0	0	1	0	0	0	0	0	0	0	0	1	0	0	0	0	0	0	0	0	0	0	0	0	0	0	0	0	0	0	0	0	0	0	0	0	1	0	0	0	0	0	0	0	0	0	0	0	0	0	1	41.7506	20.26256667
580	2005/091		0	0	0	0	0	0	0	0	0	0	0	0	0	0	0	0	0	0	0	0	0	1	0	0	0	0	0	0	0	0	0	0	0	0	0	0	0	0	0	0	0	0	0	0	0	0	0	0	0	0	0	1	0	0	0	0	0	0	0	0	0	0	0	0	0	0	0	0	0	1	41.78555	20.28701667
581	2005/092		1	0	0	0	0	1	0	0	0	0	0	0	0	0	1	0	0	0	0	0	0	1	0	0	0	0	0	0	0	0	0	0	0	0	0	0	0	0	0	0	0	0	0	0	0	0	0	0	0	0	0	1	0	0	0	0	0	0	0	0	0	0	0	0	0	0	0	0	0	1	41.7994	20.28061667
582	2005/093		1	0	0	0	0	1	0	0	1	1	0	0	1	1	1	1	0	0	1	0	0	1	0	1	0	0	0	0	0	0	1	0	0	0	0	0	0	0	0	0	0	0	0	0	0	0	0	0	0	0	1	1	1	0	0	1	0	0	0	0	0	0	0	0	0	0	0	0	0	3	41.81486667	20.2775
586	2005/097		1	0	0	0	0	0	0	0	0	0	0	0	0	0	0	0	0	0	0	0	0	1	0	0	0	0	0	0	0	0	1	0	0	0	0	0	0	0	0	0	0	0	0	0	0	0	0	0	0	0	0	0	0	0	0	0	0	0	0	0	0	0	0	0	0	0	0	0	1	1	41.61865	20.28953333
587	2005/098		0	0	0	0	0	1	0	0	1	0	0	0	1	0	1	0	0	0	0	0	0	1	0	0	0	1	0	0	0	0	0	0	1	0	0	0	0	0	1	0	0	1	0	0	0	1	0	0	0	0	0	0	0	0	0	0	0	0	0	0	0	0	0	0	0	0	0	0	0	1	42.73731667	20.3068
588	2005/099		0	0	0	0	0	0	0	0	0	0	0	0	1	0	1	0	0	0	0	0	0	0	0	0	0	0	0	0	0	0	0	1	0	0	0	0	0	0	0	0	0	0	0	0	0	0	0	0	0	0	0	0	0	0	0	0	0	0	0	0	0	0	0	0	0	0	0	0	0	1	42.85305	20.20396667
589	2005/100		1	0	0	0	0	0	0	0	0	0	0	0	1	0	1	0	0	0	0	0	0	1	0	0	0	1	0	0	0	0	0	0	1	0	0	0	0	0	0	0	0	1	0	0	0	1	0	0	0	0	0	0	0	0	0	0	0	0	0	0	0	0	0	0	0	0	0	0	0	1	42.90683333	20.33436667
590	2005/101		0	0	0	0	1	0	0	0	1	0	0	0	1	0	1	0	0	0	0	0	0	1	0	0	1	0	0	0	0	0	0	0	1	0	0	0	0	0	1	0	0	1	0	0	0	1	0	0	0	0	0	0	0	0	0	0	0	0	0	0	0	0	0	0	0	0	0	0	0	1	43.13183333	20.41605
591		0	1	0	0	0	0	0	0	0	0	0	0	0	0	0	0	0	0	0	0	0	0	0	0	0	0	0	0	0	0	0	0	0	0	0	0	0	0	0	0	0	0	0	0	0	0	0	0	0	0	0	0	0	0	0	0	0	0	0	0	0	0	0	0	0	0	0	0	0	0	1	42.305	18.8889
593		0	1	0	0	0	0	0	0	0	0	0	0	0	0	0	0	0	0	0	0	0	0	0	0	0	0	0	0	0	0	0	0	0	0	0	0	0	0	0	0	0	0	0	0	0	0	0	0	0	0	0	0	0	0	0	0	0	0	0	0	0	0	0	0	0	0	0	0	0	0	1	42.474306	18.53625
596	2006/005		1	0	0	0	0	0	0	0	1	1	0	0	1	1	1	0	0	0	0	0	0	0	0	0	0	0	0	0	0	0	1	1	0	0	0	0	0	0	0	0	0	0	0	0	0	0	0	0	0	0	1	1	0	0	0	0	0	0	0	0	0	0	0	0	0	0	0	0	0	1	41.03578333	20.06861667
598	2006/007		1	0	0	0	0	0	0	0	0	0	0	0	0	1	1	0	0	0	0	0	0	1	0	0	0	0	0	0	0	0	1	0	0	0	0	0	0	0	0	0	0	0	0	0	0	0	0	0	0	0	0	1	0	0	0	0	0	0	0	0	0	0	0	0	0	0	0	0	0	1	40.74306667	20.12713333
599	2006/008		0	0	0	0	0	0	0	0	0	1	0	0	0	0	0	0	0	0	0	0	0	0	0	0	0	0	0	0	0	0	0	0	0	0	0	0	0	0	0	0	0	0	0	0	0	0	0	0	0	0	0	1	0	0	0	0	0	0	0	0	0	0	0	0	0	0	0	0	0	1	41.15756667	20.12466667
600	2006/009		1	0	0	0	0	1	0	0	1	1	0	0	1	0	0	0	0	1	0	0	1	1	0	1	0	1	0	0	1	0	0	0	0	0	0	0	0	0	0	0	0	0	0	0	0	0	0	0	0	0	1	1	0	0	0	0	0	0	0	0	0	0	0	0	0	0	0	0	0	1	41.20353333	20.11458333
601	2006/010		1	0	0	0	0	0	0	0	0	0	0	0	1	0	1	0	0	0	0	0	0	1	0	0	0	0	0	0	0	0	0	1	0	0	0	0	0	0	0	0	0	0	0	0	0	0	0	0	0	0	0	1	0	0	0	0	0	0	0	0	0	0	0	0	0	0	0	0	0	1	41.21271667	20.1364
602	2006/011		0	0	0	0	0	1	0	0	1	1	0	0	0	0	1	0	0	0	0	0	0	1	0	1	0	0	0	0	0	0	0	0	0	0	0	0	0	0	0	0	0	0	0	0	0	0	0	0	0	0	0	1	0	0	0	0	0	0	0	0	0	0	0	0	0	0	0	0	0	1	41.2226	20.12675
604	2006/013		0	0	0	0	0	1	0	0	0	0	0	0	0	0	0	0	0	0	0	0	0	1	0	0	0	0	0	0	0	0	0	0	0	0	0	0	0	0	0	0	0	0	0	0	0	0	0	0	0	0	0	1	0	0	0	0	0	0	0	0	0	0	0	0	0	0	0	0	0	1	41.23886667	20.0976
607	2006/016		1	0	0	0	0	1	0	0	1	0	0	0	1	0	1	0	0	0	0	0	0	1	0	1	0	0	0	0	0	0	1	0	0	0	0	0	0	0	0	0	0	0	0	0	0	0	0	0	0	0	0	1	0	0	0	0	0	0	0	0	0	0	0	0	0	0	0	0	0	1	41.29093333	20.0832
608	2006/017		0	0	0	0	0	0	0	0	1	0	0	0	0	0	0	1	1	0	1	1	0	0	0	0	0	0	0	0	0	0	0	0	0	0	0	0	0	0	0	0	0	0	0	0	0	0	0	0	0	0	0	0	0	0	0	0	0	0	0	0	0	0	0	0	0	0	0	0	0	3	41.2951	20.10085
609	2006/018		1	0	0	0	0	1	0	0	1	1	0	0	0	0	0	0	0	0	0	0	0	1	0	1	0	0	0	0	0	0	0	0	0	0	0	0	0	0	0	0	0	0	0	0	0	0	0	0	0	0	0	1	0	0	0	0	0	0	0	0	0	0	0	0	0	0	0	0	0	1	41.29063333	20.11888333
611	2006/020		1	0	0	0	0	1	0	0	0	0	0	0	0	1	0	0	0	0	0	0	0	0	0	0	0	0	0	0	0	0	1	0	0	0	0	0	0	0	0	0	0	0	0	0	0	0	0	0	0	0	0	1	0	0	0	0	0	0	0	0	0	0	0	1	0	0	0	0	0	1	41.27475	20.26793333
612	2006/021		0	0	0	0	0	1	0	0	0	0	0	0	0	1	1	0	0	0	0	0	0	1	0	0	0	0	0	0	0	0	0	0	0	0	0	0	0	0	0	0	0	0	0	0	0	0	0	0	0	0	0	1	0	0	0	0	0	0	0	0	0	0	0	0	0	0	0	0	0	1	41.266	20.25955
615	2006/024		1	0	0	0	0	1	0	0	1	1	0	0	1	1	0	0	0	0	0	0	0	1	0	0	0	0	0	0	0	0	0	0	0	0	0	0	0	0	0	0	0	0	0	0	0	0	0	0	0	0	1	1	0	0	0	0	0	0	0	0	0	0	0	0	0	0	0	0	1	1	41.26601667	20.3182
622	2006/031A		1	0	0	0	0	1	0	0	0	0	0	0	1	1	1	0	0	0	0	0	0	1	0	0	0	1	0	0	0	0	1	0	0	0	0	0	0	0	0	0	0	0	0	0	0	0	0	0	0	0	0	0	0	0	0	1	0	0	0	0	0	0	0	0	0	0	0	0	0	1	41.75205	20.25241667
623	2006/031B		1	0	0	0	0	1	0	0	0	0	0	0	1	0	0	0	0	1	0	0	1	1	0	0	0	0	0	0	0	0	0	0	0	0	0	0	0	0	0	0	0	0	0	0	0	0	0	0	0	0	0	0	0	0	0	1	0	0	0	0	0	0	0	0	0	0	0	0	0	1	41.75283333	20.25048333
625	2006/032		1	0	0	0	0	1	0	0	0	0	0	0	1	0	1	0	0	0	0	0	0	1	0	0	0	0	0	0	0	0	0	0	0	0	0	0	0	0	0	0	0	0	0	0	0	0	0	0	0	0	0	0	0	0	0	0	0	0	0	0	0	0	0	0	0	0	0	0	0	1	41.75713333	20.23888333
628	2006/034		0	0	0	0	0	1	0	0	0	0	0	0	0	0	0	0	0	0	0	0	0	0	0	0	0	0	0	0	0	0	0	0	0	0	0	0	0	0	0	0	0	0	0	0	0	0	0	0	0	0	1	0	0	0	0	0	0	0	0	0	0	0	0	0	0	0	0	0	0	1	41.66241667	20.31728333
632	2006/038		0	0	0	0	0	0	0	0	0	0	0	0	0	0	0	0	0	0	0	0	0	1	0	0	0	0	0	0	0	0	0	0	0	0	0	0	0	0	0	0	0	0	0	0	0	0	0	0	0	0	0	0	0	0	0	0	0	0	0	0	0	0	0	0	0	0	1	0	1	41.64511667	20.18231667
633	2006/039		1	0	0	0	0	1	0	0	0	0	0	0	1	0	1	0	0	0	0	0	0	1	0	0	0	1	0	0	0	0	1	0	0	0	0	0	0	0	0	0	0	0	0	0	0	0	0	0	0	0	0	1	1	0	0	0	0	0	0	0	0	0	0	0	0	0	0	1	0	1	41.64605	20.1806
635	2006/041		1	0	0	0	0	0	0	0	0	0	0	0	0	0	0	0	0	0	0	0	0	0	0	0	0	0	0	0	0	0	0	0	0	0	0	0	0	0	0	0	0	0	0	0	0	0	0	0	0	0	0	0	0	0	0	1	0	0	0	0	0	0	0	0	0	0	0	0	0	1	41.65025	20.18606667
637	2006/043		1	0	0	0	0	0	0	0	0	0	0	0	1	0	1	0	0	0	0	0	0	1	0	0	0	0	0	0	0	0	1	0	0	0	0	0	0	0	0	0	0	0	0	0	0	0	0	0	0	0	0	1	0	0	0	1	0	0	0	0	0	0	0	0	0	0	0	0	0	1	41.63516667	20.12433333
642	2006/048		1	1	0	0	0	1	0	0	1	1	0	0	0	1	1	0	0	1	0	0	1	1	0	1	0	1	0	0	1	0	1	0	0	1	0	0	0	0	0	1	0	0	0	0	0	0	0	0	0	0	1	1	0	0	0	0	0	0	0	0	0	0	0	0	0	0	0	0	0	1	42.1217	19.83136667
643	2006/049		1	0	0	0	0	1	0	0	1	1	0	0	1	1	1	0	0	0	0	0	0	0	0	0	0	1	0	0	0	0	1	1	0	1	0	0	0	0	0	0	0	0	0	0	0	0	0	0	0	0	0	1	0	0	0	0	0	0	0	0	0	0	0	0	0	0	0	0	0	1	42.11655	19.83738333
644	2006/050		1	0	0	0	0	0	0	0	0	0	0	0	0	1	1	0	0	0	0	0	0	1	0	0	0	0	0	0	1	0	1	0	0	0	0	0	0	0	0	1	0	0	0	0	0	0	0	0	0	0	1	0	0	0	0	0	0	0	0	0	0	0	0	0	0	0	0	0	0	1	42.08093333	19.73621667
646	2006/052		1	0	0	0	0	1	0	0	1	0	0	0	0	0	1	0	0	0	0	0	0	1	0	0	0	0	0	0	0	0	0	1	0	0	0	0	0	0	0	1	0	0	0	0	0	0	0	0	0	0	0	0	0	1	0	0	0	0	0	0	0	0	0	0	0	0	0	0	0	1	42.18715	19.86955

647	2006/053	1	0	0	0	0	0	0	0	1	1	0	0	0	0	1	0	0	0	1	0	0	0	1	0	0	0	0	1	0
649	2006/055	1	0	0	0	1	1	0	0	0	1	0	0	0	1	1	0	1	1	1	0	1	0	0	0	0	0	0	0	0
650	2006/056	1	1	0	0	1	1	0	0	1	1	0	0	1	1	1	0	0	1	1	0	0	1	1	0	1	1	0	0	0
652	2006/058	0	0	0	0	0	1	0	0	0	0	0	0	0	1	1	0	0	0	0	0	0	0	0	0	0	0	0	1	0
653	2006/059	1	0	0	0	0	0	0	0	0	0	0	0	1	1	1	0	0	0	0	0	0	0	0	0	0	0	0	0	1
654	2006/060	1	0	0	0	0	1	0	0	0	0	0	0	0	0	1	0	0	0	1	0	0	0	0	0	0	0	1	0	0
655	2006/061	1	0	0	0	0	0	0	0	0	0	0	0	0	0	0	0	0	0	0	0	0	0	0	0	0	0	0	0	0
656	2006/062	1	0	0	0	0	0	0	0	0	0	0	0	0	1	0	0	0	0	0	0	0	0	0	0	0	0	0	1	0
658	2006/064	1	0	0	0	0	1	0	0	0	1	0	0	0	1	1	0	0	0	0	0	0	0	0	0	0	0	0	1	0
659	2006/065	1	0	0	0	0	0	0	0	1	0	0	0	1	1	1	0	0	0	0	0	0	0	0	0	0	0	0	1	0
662	2006/068	1	0	0	0	0	0	0	0	0	0	0	0	0	0	0	0	0	0	0	0	0	0	0	0	0	0	0	0	0
663	2006/069	1	0	0	0	0	0	0	0	0	0	0	0	0	0	0	0	0	0	0	0	0	0	1	0	0	0	0	0	0
670	2006/075	0	0	0	0	0	0	0	0	0	0	0	0	1	0	1	0	0	0	1	0	0	0	0	0	0	0	0	0	0
672	0	1	0	0	0	0	0	0	0	0	0	0	0	0	1	1	0	0	0	1	0	0	0	0	0	0	0	0	0	0
673	0	1	0	0	0	0	0	0	0	0	0	0	0	0	0	0	0	0	0	1	0	0	0	0	0	0	0	0	1	0
676	0	0	0	0	0	0	0	0	0	0	0	0	0	0	1	0	0	0	0	0	0	0	0	0	0	0	0	0	0	0
679	0	0	0	0	0	0	0	0	0	1	0	0	0	0	0	0	0	0	0	0	0	0	0	0	0	0	0	0	0	0
681	0	0	0	0	0	0	0	0	0	0	0	0	0	0	1	1	0	0	0	0	0	0	0	0	0	0	0	0	0	0
687	0	0	0	0	0	0	1	0	0	0	0	0	0	0	0	0	0	0	0	1	0	0	0	0	0	0	0	0	0	0
692	0	0	0	0	0	0	0	0	0	0	0	0	0	0	0	1	0	0	0	1	0	0	0	1	0	0	0	0	0	0
693	0	0	0	0	0	0	0	0	0	0	0	0	0	0	0	1	0	0	0	0	0	0	0	0	0	0	0	0	0	0
694	0	0	0	0	0	0	0	0	0	0	0	0	0	0	0	0	0	0	0	1	0	0	0	0	0	0	0	0	0	0
695	0	0	0	0	0	0	0	0	0	1	0	0	0	0	0	0	0	0	0	1	0	0	0	0	0	0	0	0	0	0
696	0	1	0	0	0	0	0	0	0	0	0	0	0	0	0	0	0	0	0	0	0	0	0	0	0	0	0	0	0	0
698	0	1	0	0	0	0	0	0	0	0	0	0	0	0	0	1	0	0	0	1	0	0	0	0	0	0	0	0	0	1
699	0	1	0	0	0	0	0	0	0	0	0	0	0	0	0	0	0	0	0	1	0	0	0	0	0	0	0	0	0	0
700	0	1	0	0	0	0	0	0	0	0	0	0	0	0	0	0	0	0	0	0	0	0	0	0	0	0	0	0	0	0
701	0	0	0	0	0	0	1	0	0	0	0	0	0	0	0	0	0	0	0	0	0	0	0	0	0	0	0	0	0	0
705	2006/134	1	0	0	0	0	1	0	0	1	0	0	0	0	1	1	0	0	0	1	0	0	0	0	0	0	0	0	0	0
706	2006/135	0	0	0	0	0	1	0	0	0	0	0	0	0	0	0	1	0	0	1	0	1	0	0	0	0	0	0	0	0
707	2006/135B	0	0	0	0	0	1	0	0	0	1	0	0	0	0	0	0	0	0	0	0	1	0	0	0	0	1	0	0	0
708	2006/136	0	0	0	0	0	1	0	0	1	1	0	0	1	0	1	0	0	0	1	0	0	0	0	0	0	0	0	0	0
709	2006/137	0	0	0	0	0	0	0	0	0	0	0	0	0	0	0	0	0	0	0	0	0	0	0	0	0	0	0	0	0
710	2006/138	1	0	0	0	0	0	0	0	0	1	0	0	0	0	1	0	0	0	0	0	0	1	0	0	0	0	0	0	0
711	2006/139	0	0	0	0	0	0	0	0	1	0	0	0	0	0	0	0	0	0	1	0	0	0	0	0	0	0	0	0	0
713	2006/141	1	0	0	0	0	0	0	0	0	1	0	0	1	0	0	0	0	0	1	1	0	0	1	0	0	1	0	0	0
714	2006/142	0	0	0	0	0	0	0	0	0	0	0	0	0	0	1	0	0	0	0	0	0	0	0	0	0	0	0	0	0
715	2006/143	1	0	0	0	0	0	0	0	1	1	0	0	1	1	1	0	0	0	1	0	0	0	1	0	0	0	0	1	0
717	2006/145	0	0	0	0	0	1	0	0	0	0	0	0	0	0	1	0	0	0	0	0	0	0	0	0	0	0	0	0	0
718	2006/146	1	0	0	0	0	0	0	0	0	0	0	0	1	0	0	0	0	0	1	0	0	0	1	0	0	0	0	0	0
719	2006/147	1	0	0	0	0	0	0	0	0	0	0	0	0	0	0	0	0	0	1	0	0	0	0	0	0	0	0	0	0
720	2006/148	1	0	0	0	0	0	0	0	0	0	0	0	1	0	0	0	0	0	1	0	0	0	0	0	0	0	0	0	1
721	2006/149	0	0	0	0	0	0	0	0	0	0	0	0	1	0	1	0	0	0	0	0	1	1	0	0	0	0	0	0	0
722	2006/150	0	0	0	0	0	0	0	0	0	0	0	0	0	0	0	0	0	0	0	0	0	0	0	0	0	0	0	0	0
723	2006/151	1	0	0	0	0	0	0	0	1	1	0	0	0	0	0	0	0	0	1	0	0	0	0	0	0	0	0	0	0
724	2006/152	1	0	0	0	0	1	0	0	0	1	0	0	0	0	0	0	0	0	1	1	0	0	1	0	1	1	0	1	0
725	2006/153	1	0	0	0	0	0	0	0	0	0	0	0	0	0	1	0	0	0	1	1	0	0	0	0	0	0	0	0	0
727	2006/155	1	0	0	0	0	1	0	0	0	1	0	0	1	1	0	1	0	0	0	0	0	0	0	0	0	0	0	1	1
730	2006/158	1	0	0	0	0	0	0	0	1	0	0	0	1	0	1	0	0	0	1	0	0	0	0	0	0	0	0	0	0
734	2006/162	0	0	0	0	0	0	0	0	0	0	0	0	0	0	0	0	0	0	0	0	0	0	0	0	0	0	1	0	1
739	0	0	0	0	0	0	0	0	0	1	0	0	0	1	0	0	0	0	0	0	0	0	0	0	0	0	0	0	0	0
744	0	1	0	0	0	0	0	0	0	0	0	0	0	0	1	0	0	0	0	0	0	0	0	0	0	0	0	0	0	0
748	0	0	0	0	0	0	0	0	0	1	0	0	0	0	0	0	0	0	0	0	0	0	0	0	0	0	0	0	0	0
750	0	0	0	0	0	0	0	0	0	0	0	0	0	1	0	0	0	0	0	1	0	1	0	0	0	0	0	0	0	0
751	0	0	0	0	0	0	0	0	0	0	0	0	0	1	0	0	0	0	0	1	0	0	0	0	0	0	0	0	0	0
752	0	0	0	0	0	0	1	0	0	0	0	0	0	0	0	0	0	0	0	0	0	0	0	0	0	0	0	0	0	0
753	0	0	0	0	0	0	0	0	0	0	0	0	0	0	0	0	0	0	0	0	0	0	0	0	0	0	0	0	0	0
754	0	0	0	0	0	0	0	0	0	0	0	0	0	0	0	0	0	0	0	0	0	0	0	0	0	0	0	0	0	0
755	0	0	0	0	0	0	0	0	0	0	0	0	0	0	0	0	0	0	0	0	0	0	0	0	0	0	0	0	0	0
757	0	0	0	0	0	0	0	0	0	0	0	0	0	0	0	0	0	0	0	1	0	0	0	0	0	0	0	0	0	0
758	0	1	0	0	0	0	1	0	0	0	0	0	0	0	0	0	0	0	0	1	0	0	0	0	0	0	0	0	0	0
759	0	1	0	0	0	0	0	0	0	0	0	0	0	0	0	0	0	0	0	1	0	0	0	1	0	0	0	0	0	0
760	2007/091	0	0	0	0	0	0	0	0	0	0	0	0	1	0	1	0	0	0	0	0	1	0	0	0	0	0	0	0	0
761	2007/092	0	0	0	0	0	0	0	0	0	0	0	0	0	0	0	0	0	0	0	0	0	0	0	0	0	0	0	0	0
763	2007/102	0	0	0	0	0	1	0	0	0	0	0	0	1	1	1	0	0	0	1	0	0	0	0	0	0	0	0	0	0
764	2007/103	0	0	0	0	0	0	0	0	1	0	0	0	0	0	1	0	0	0	1	0	0	0	0	0	0	1	0	0	0
765	2007/104	0	0	0	0	0	0	0	0	0	0	0	0	0	0	1	0	0	0	0	0	0	0	0	0	0	0	0	1	0
767	2007/106	0	0	0	0	0	0	0	0	1	0	0	0	0	0	0	0	0	0	0	0	0	0	0	0	0	0	0	0	0
774	2007/112	0	0	0	0	0	0	0	0	0	0	0	0	0	0	0	0	0	0	0	0	0	0	0	0	0	0	0	0	0
775	2007/113	0	0	0	0	0	0	0	0	0	0	0	0	0	0	1	0	0	0	1	0	0	0	0	0	0	0	0	0	0
776	2007/114	0	0	0	0	0	1	0	0	0	0	0	0	1	0	1	0	0	0	0	0	1	0	0	0	0	0	0	0	0
777	2007/115	1	0	0	0	0	1	0	0	1	1	0	0	1	0	1	0	0	0	0	0	1	0	1	0	0	0	0	0	0
779	2007/117	0	0	0	0	0	0	0	0	0	0	0	0	0	0	1	0	0	0	0	0	0	0	0	0	0	0	0	0	0
780	2007/117B	0	0	0	0	0	0	0	0	0	0	0	0	0	0	0	0	0	0	0	0	0	0	0	0	0	0	0	0	0
783	2007/120	1	0	0	0	0	1	0	0	0	1	0	0	1	1	1	1	0	0	1	0	0	0	1	0	0	0	0	1	0
784	2007/121	0	0	0	0	0	1	0	0	0	0	0	0	1	0	0	0	0	0	1	0	0	0	0	0	0	0	0	0	0
785	2007/122	0	0	0	0	0	0	0	0	0	0	0	0	0	0	0	0	0	0	1	0	0	0	0	0	0	0	0	0	0
786	2007/123	1	0	0	0	0	1	0	0	1	0	0	0	1	0	0	1	0	0	1	0	0	0	1	0	0	0	0	1	0
789	2007/126	0	0	0	0	0	1	0	0	1	0	0	0	1	0	1	1	0	0	1	0	1	0	0	0	0	1	0	0	0
790	2007/127	0	0	0	0	0	1	0	0	0	0	0	0	0	0	1	1	0	0	1	0	1	0	1	0	0	0	0	0	0
792	2007/128	0	0	0	0	0	0	0	0	1	1	0	0	1	0	1	1	0	0	0	0	0	0	0	0	0	0	0	0	0
793	2007/129	0	0	0	0	0	0	0	0	0	1	0	0	0	0	0	0	0	0	0	0	0	0	0	0	0	0	0	0	0
795	2007/131	0	0	0	0	0	0	0	0	0	1	0	0	1	0	0	0	0	0	0	0	0	0	0	0	0	0	0	0	0
796	2007/132	0	0	0	0	0	0	0	0	0	0	0	0	0	0	0	0	0	0	0	0	0	0	0	0	0	0	0	0	0
797	2007/133	0	0	0	0	0	0	0	0	0	1	0	0	0	0	1	1	0	0	0	0	0	1	0	0	0	0	0	0	0
798	2007/134	0	0	0	0	0	0	0	0	0	0	0	0	0	0	1	0	0	0	0	0	0	0	0	0	0	0	0	0	0
803	2007/139	0	0	0	0	0	0	0	0	0	1	0	0	1	0	0	1	0	0	0	0	0	0	0	0	0	0	0	0	0
805	2007/141	0	0	0	0	0	0	0	0	0	0	0	0	1	0	0	0	0	0	0	0	0	0	0	0	0	0	0	0	0
810	2007/146	0	0	0	0	0	1	0	0	1	1	0	0	1	0	0	0	0	0	0	0	0	0	0	0	0	0	0	0	0
813	2007/149	1	0	0	0	0	1	0	0	0	1	0	0	0	0	0	0	0	0	1	0	1	0	1	0	0	1	0	1	0
817	2007/153	1	0	0	0	0	1	0	0	0	0	0	0	0	0	0	0	0	0	1	0	1	0	0	0	0	0	0	1	0
819	2007/155	0	0	0	0	0	1	0	0	0	0	0	0	1	0	1	0	0	0	0	0	0	0	0	0	0	0	1	0	0
823	2007/159	1	0	0	0	0	0	0	0	1	0	0	0	1	0	1	0	0	0	0	0	0	0	0	0	0	0	0	1	0
828	2007/164	1	0	0	0	0	1	0	0	0	0	0	0	0	0	0	0	0	0	1	0	0	0	0	0	0	0	0	0	0
831	2007/167	1	0	0	0	0	1	0	0	1	0	0	0	1	0	1	0	0	0	1	0	0	0	0	0	0	0	0	0	0
836	2007/172	0	0	1	0	0	1	0	0	1	1	0	0	0	0	1	0	0	0	0	0	0	0	1	0	0	1	0	0	0
838	2007/174	1	0	0	0	0	0	0	0	0	0	0	0	0	1	1	0	0	0	0	0	0	0	0	0	0	1	0	0	0
841	2007/177	1	0	0	0	0	1	0	0	1	1	0	0	1	0	1	0	0	0	1	1	0	0	1	0	1	0	0	0	0
843	2007/179	1	0	1	0	0	1	0	0	0	0	0	0	0	1	1	0	0	0	1	0	0	0	0	0	0	1	0	1	0
844	0	0	0	0	0	0	0	0	0	0	0	0	0	0	0	0	0	0	0	1	0	0	0	0	0	0	0	0	0	0
845	0	0	0	0	0	0	0	0	0	0	0	0	0	0	0	0	0	0	0	0	0	0	0	0	0	0	0	0	0	1
846	2007/180	1	0	1	0	0	0	0	0	1	1	0	0	1	0	1	0	0	0	0	0	0	0	1	0	0	1	0	1	0
848	2007/182	1	0	0	0	0	0	0	0	0	0	0	0	0	0	1	0	0	0	0	0	0	0	0	0	0	0	0	0	0
850	2007/184	1	0	0	0	0	0	0	0	0	0	0	0	0	1	1	0	0	0	0	0	0	0	0	0	0	0	0	0	0
854	2007/188	1	0	0	0	0	0	0	0	1	0	0	0	1	0	0	0	0	0	1	0	0	0	0	0	0	0	0	0	1
855	2007/189	1	0	0	0	0	0	0	0	0	1	0	0	1	0	0	0	1	0	0	0	0	0	0	0	1	0	0	0	0
856	2007/190	0	0	0	0	0	0	0	0	0	0	0	0	1	0	0	1	0	0	1	0	0	0	0	0	0	0	0	0	1
864	0	1	0	0	0	0	0	0	0	0	0	0	0	0	0	0	0	0	0	0	0	0	0	0	0	0	0	0	0	0
865	2008/110	1	0	0	0	0	0	0	0	0	0	0	0	1	0	1	0	0	0	1	0	0	0	0	0	0	0	0	0	0
866	2008/111	1	0	0	0	0	1	0	0	0	0	0	0	0	0	0	0	0	0	0	0	0	0	0	0	0	0	0	0	1
867	2008/112	0	0	0	0	0	0	0	0	0	0	0	0	0	0	0	0	0	0	0	0	0	0	0	0	0	0	0	0	0
868	2008/113	1	0	0	0	0	0	0	0	0	0	0	0	0	0	0	0	0	0	0	0	0	0	0	0	0	0	0	0	0
871	2008/116	1	0	0	1	0	1	0	0	0	1	0	0	1	0	1	0	0	0	1	0	1	0	0	0	0	0	0	1	0
872	2008/117	1	0	0	0	0	1	0	0	0	0	0	0	1	0	1	0	0	0	1	0	0	0	1	0	0	0	0	1	0
873	2008/118	1	0	0	0	0	1	0	0	0	1	0	0	1	0	0	0	1	0	1	0	0	0	0	0	0	0	0	0	0
874	2008/119	0	0	0	0	0	0	0	0	0	0	0	0	1	0	0	0	0	0	0	0	0	0	0	0	0	0	0	0	1
875	2008/120	1	0	0	0	0	1	0	0	0	0	0	0	0	0	0	0	0	0	0	0	0	0	0	0	0	0	0	0	0
876	2008/120A	0	0	0	0	0	0	0	0	0	0	0	0	0	0	0	0	0	0	0	0	0	0	0	0	0	0	0	0	0
877	2008/121	1	0	0	0	0	0	0	0	0	0	0	0	0	0	0	0	0	0	0	0	0	0	0	0	0	0	0	0	0
880	2008/124	0	0	0	0	0	0	0	0	0	0	0	0	0	0	0	0	0	0	0	0	0	0	0	0	0	0	0	0	0
882	2008/126	0	0	0	0	1	0	0	0	0	0	0	0	0	0	1	0	0	0	0	0	0	0	0	0	0	0	0	0	0
883	2008/127	0	0	0	0	0	0	0	0	1	0	0	0	0	0	0	0	0	0	0	0	0	0	0	0	0	0	0	0	0
884	2008/128	0	0	0	0	0	1	0	0	0	0	0	0	0	0	0	0	0	0	0	0	0	0	0	0	0	0	0	0	0
885	2008/129	1	0	0	0	0	0	0	0	1	0	0	0	1	0	1	0	0	0	0	0	0	0	0	0	0	0	0	0	0
886	2008/130	1	0	0	0	0	0	0	0	0	0	0	0	1	0	0	0	0	0	1	0	0	0	0	0	0	0	0	0	0
888	2008/132	0	0	0	0	0	0	0	0	0	0	0	0	1	0	1	0	0	0	0	0	0	0	0	0	0	0	0	0	0
889	2008/133	0	0	0	0	0	0	0	0	1	1	0	0	1	0	0	0	0	0	0	0	0	0	1	0	0	0	0	0	1
890	2008/134	1	0	0	0	0	1	0	0	0	1	0	0	1	0	0	0	0	0	1	0	1	0	0	0	0	1	0	0	0
891	2008/135	1	0	0	0	0	0	0	0	0	0	0	0	0	0	0	1	0	0	1	0	0	0	0	0	0	0	0	0	0
892	2008/136	0	0	0	0	0	0	0	0	1	0	0	0	1	0	1	0	0	0	0	0	0	0	0	0	0	0	0	1	1
893	2008/137	0	0	0	0	0	1	0	0	0	0	0	0	0	0	1	0	0	0	1	0	0	0	0	0	0	0	0	0	0
894	2008/138	0	0	0	0	0	1	0	0	0	0	0	0	1	0	1	0	0	0	1	0	0	0	0	0	0	0	0	0	0
895	2008/139	0	0	0	0	0	0	0	0	0	0	0	0	0	0	0	0	0	0	0	0	0	0	0	0	0	0	0	0	0
896	2008/140	0	0	0	0	0	0	0	0	1	0	0	0	1	0	1	0	0	0	1	0	0	0	0	0	0	0	0	0	1
897	2008/141	1	0	0	0	0	0	0	0	0	0	0	0	1	0	1	0	0	0	1	0	0	0	0	0	0	0	0	0	0
898	2008/142	1	0	0	0	0	0	0	0	0	0	0	0	1	0	0	0	0	0	0	0	0	0	0	0	0	0	0	0	1
899	2008/143	1	0	0	0	0	1	0	0	1	1	0	0	1	0	0	0	0	0	1	0	0	0	0	0	0	0	0	0	0
903	2008/147	0	0	0	0	0	0	0	0	0	0	0	0	1	0	1	0	0	0	1	0	0	0	0	0	0	0	0	1	0
904	2008/148	0	0	0	0	0	0	0	0	0	0	0	0	0	0	0	0	0	0	0	0	0	0	0	0	0	0	0	0	0
905	2008/149	1	0	0	0	0	1	0	0	0	0	0	0	0	0	0	0	0	0	0	0	0	0	0	0	0	0	0	0	0
906	2008/149A	0	0	0	0	0	0	0	0	0	0	0	0	0	0	0	0	0	0	0	0	0	0	0	0	0	0	0	0	0
908	2008/151	0	0	0	0	0	0	0	0	0	0	0	0	1	0	1	1	0	0	0	0	0	0	0	0	0	0	0	0	0
909	2008/152	1	0	0	0	0	1	0	0	0	0	0	0	1	0	1	0	0	0	1	0	0	0	0	0	0	0	0	0	0
910	2008/153	1	0	0	0	0	1	0	0	1	0	0	0	0	0	1	1	1	0	0	0	0	0	1	0	0	0	0	0	0
911	2008/154	0	0	0	0	0	0	0	0	0	0	0	0	0	0	0	0	0	0	0	0	0	0	0	0	0	0	0	0	0
912	2008/155	0	0	0	0	0	1	0	0	0	1	0	0	1	0	0	0	0	0	0	0	0	0	0	0	1	0	0	0	0
915	2008/158	0	0	0	0	0	0	0	0	1	0	0	0	0	0	0	0	0	1	0	0	0	0	0	0	0	0	0	0	0
916	2008/159	0	0	0	0	0	0	0	0	0	0	0	0	0	0	0	0	0	0	0	0	0	0	0	0	0	0	0	0	0
917	2008/160	0	0	0	0	0	0	0	0	0	0	0	0	1	0	0	0	0	0	0	0	1	0	0	0	0	0	0	0	0
919	2008/162	1	0	0	0	0	1	0	0	1	1	0	0	1	1	1	0	1	0	1	0	1	0	0	0	1	1	0	0	0
921	2008/163R	0	0	0	0	0	0	0	0	0	0	0	0	1	1	0	0	0	1	1	0	0	0	0	0	0	0	0	0	0
922	2008/164	0	0	0	0	0	0	0	0	0	0	0	0	0	0	0	0	0	0	0	0	0	0	0	0	0	0	0	0	0
923	2008/165	0	0	0	0	1	0	0	0	0	1	0	0	0	0	0	1	0	0	1	0	0	0	0	0	0	0	0	0	0
924	2008/166	1	0	0	0	0	1	0	0	1	0	0	0	1	0	1	0	0	0	0	0	0	0	0	0	0	0	0	0	0
929	2008/171	0	0	0	0	0	1	0	0	1	0	0	0	1	0	1	0	0	0	1	0	1	0	0	0	0	0	0	0	1
930	2008/172	1	0	0	0	0	1	0	0	0	0	0	0	1	0	0	0	0	0	0	0	0	0	0	0	0	0	0	0	1
932	2008/174	0	0	0	0	1	0	0	0	0	0	0	0	1	0	1	0	0	0	0	0	0	0	0	0	0	0	0	0	0
933	2008/175	0	0	0	0	0	0	0	0	0	0	0	0	0	0	0	0	0	0	0	0	0	0	0	0	0	0	0	0	0
934	2008/176	0	0	0	0	0	1	0	0	0	0	0	0	1	0	1	0	0	0	0	0	1	0	0	0	0	0	0	0	1
935	2008/177	0	0	0	0	0	1	0	0	0	0	0	0	0	0	1	0	0	0	0	0	0	0	0	0	0	0	0	0	0
936	2008/178	1	0	0	0	0	0	0	0	0	0	0	0	0	0	0	0	0	0	0	0	1	0	0	0	0	0	0	1	0
937	2008/179	1	0	0	0	0	1	0	0	0	0	0	0	1	0	0	0	0	0	0	0	0	0	0	0	0	0	0	0	1
938	2008/180	1	0	0	0	0	1	0	0	0	0	0	0	0	0	0	0	0	0	0	0	0	0	0	0	0	0	0	0	1
939	2008/181	1	0	0	0	0	1	0	0	0	0	0	0	0	0	0	0	0	0	0	0	0	0	0	0	0	0	0	0	0
940	2008/182	1	0	1	0	0	1	0	0	1	1	0	0	0	0	0	0	1	1	0	0	1	0	0	1	1	0	0	0	0
941	2008/183	0	0	0	0	0	0	0	0	0	0	0	0	1	0	1	0	0	0	0	0	0	0	0	0	0	0	0	1	0
942	2008/184	0	0	0	0	0	0	0	0	0	0	0	0	0	0	0	0	0	0	1	0	0	0	0	0	0	0	0	0	0
943	2008/185	0	0	0	0	0	0	0	0	0	0	0	0	0	0	0	0	0	0	0	0	0	0	0	0	0	0	0	0	0
944	2008/185B	1	0	0	0	0	1	0	0	0	0	0	0	0	0	1	0	0	0	1	0	0	0	0	0	0	0	0	0	0
945	2008/185C	0	0	0	0	0	0	0	0	0	0	0	0	0	0	0	0	0	0	0	0	0	0	0	0	0	0	0	0	0
946	2008/186	1	0	0	0	0	1	0	0	0	0	0	0	0	0	0	0	0	0	0	0	0	0	0	0	0	0	0	0	0
948	2008/188	0	0	0	0	0	0	0	0	1	1	0	0	1	0	1	0	0	0	1	0	0	0	0	0	0	0	0	0	0
951	0	1	0	0	0	0	1	0	0	0	0	0	0	0	0	0	0	0	0	0	0	0	0	0	0	0	0	0	0	0
952	0	1	0	0	0	0	0	0	0	0	0	0	0	0	0	0	0	0	0	0	0	0	0	0	0	0	0	0	1	0
954	0	1	0	0	0	0	0	0	0	0	0	0	0	0	1	0	0	0	0	0	0	0	0	0	0	0	0	0	0	0
955	0	1	0	0	0	0	0	0	0	1	0	0	0	0	0	1	0	0	0	0	0	0	0	0	0	0	0	0	0	0

647	0	1	0	0	0	0	0	0	0	0	1	0	0	0	0	0	0	0	0	0	0	0	0	0	0	0	0	0	0	0	0	0	0	0	0	1	42.20535	19.89178333
649	0	0	0	0	0	0	0	0	0	0	1	0	0	0	0	0	0	0	0	0	1	0	0	0	0	0	0	0	0	0	0	0	0	0	0	1	42.22583333	19.89236667
650	0	0	0	0	1	0	0	0	0	0	1	0	0	0	0	0	0	1	1	0	1	0	0	0	0	0	0	0	0	0	0	0	0	0	0	1	42.22688333	19.905
652	0	1	0	0	0	0	0	0	0	0	1	0	0	0	0	0	0	0	0	0	0	0	0	0	0	0	0	1	0	0	0	0	0	0	0	1	42.06913333	19.41313333
653	0	0	1	0	0	0	0	0	0	0	1	0	0	0	0	0	0	0	0	0	0	0	0	0	0	0	0	1	0	0	0	0	0	0	0	1	42.07236667	19.39463333
654	1	0	0	0	0	0	0	0	0	0	0	0	0	0	0	0	0	0	0	0	0	0	0	0	0	0	0	0	0	0	0	0	0	0	0	1	42.14695	19.54543333
655	0	0	0	0	0	0	0	0	0	0	0	0	0	0	0	0	0	0	0	0	0	0	0	0	0	0	0	0	0	0	0	0	0	0	0	1	42.1523	19.5402
656	0	1	0	0	0	0	0	0	0	0	1	0	0	0	0	0	0	0	0	0	0	0	0	0	0	0	0	0	0	0	0	0	0	0	0	1	42.18966667	19.5621
658	0	1	0	0	0	0	0	0	0	0	1	0	0	0	0	0	0	0	0	0	0	0	0	0	0	0	0	0	0	0	0	0	0	0	0	1	42.2045	19.568
659	0	1	0	0	0	0	0	0	0	0	1	0	0	0	0	0	0	0	0	0	0	0	0	0	0	0	0	0	0	0	0	0	0	0	0	1	42.21146667	19.57916667
662	0	0	0	0	0	0	0	0	0	0	0	0	0	0	0	0	0	0	0	0	0	0	0	0	0	0	0	0	0	0	0	0	0	0	0	1	42.11793333	19.65896667
663	0	0	0	0	0	0	0	0	0	0	0	0	0	0	0	0	0	0	0	0	0	0	0	0	0	0	0	0	0	0	0	0	0	0	0	1	42.12571667	19.66768333
670	0	0	0	1	0	0	0	0	0	0	0	0	1	0	0	0	0	0	0	0	0	0	0	0	0	0	0	0	0	0	0	0	0	0	0	1	43.46708333	19.6512
672	0	0	0	0	0	1	0	0	0	0	0	0	0	0	0	0	0	0	0	0	0	0	0	0	0	0	0	0	0	0	0	0	0	0	0	2	40.143333	19.662222
673	0	1	0	0	0	0	0	0	0	0	0	0	0	0	0	0	0	0	0	0	0	0	0	0	0	0	0	0	0	0	0	0	0	0	0	1	40.143694	19.677
676	0	0	0	0	0	0	0	0	0	0	0	0	0	0	0	0	0	0	0	0	0	0	0	0	0	0	0	0	0	0	0	0	0	0	0	3	40.211944	19.579167
679	0	0	0	0	0	0	0	0	0	0	0	0	0	0	0	0	0	0	1	0	0	0	0	0	0	0	0	0	0	0	0	0	0	0	0	1	39.557917	20.468833
681	0	0	0	0	0	0	0	0	0	0	0	0	0	0	0	0	0	1	1	0	0	0	0	0	0	0	0	0	0	0	0	0	0	0	0	2	39.695278	20.703611
687	0	0	0	0	0	0	0	0	0	0	0	0	0	0	0	0	0	0	0	0	0	0	0	0	0	0	0	0	0	0	0	0	0	0	0	1	40.107694	21.021167
692	0	0	0	0	0	0	0	0	0	0	0	0	0	0	0	0	0	0	0	0	0	0	0	0	0	0	0	0	0	0	0	0	0	0	0	1	40.20301	20.31674
693	0	0	0	0	0	0	0	0	0	0	0	0	0	0	0	0	0	0	0	0	0	0	0	0	0	0	0	0	0	0	0	0	0	0	0	1	40.1933	20.31429
694	0	0	0	0	0	0	0	0	0	0	0	0	0	0	0	0	0	0	0	0	0	0	0	0	0	0	0	0	0	0	0	0	0	0	0	1	40.50547	20.55125
695	0	0	0	0	0	0	0	0	0	0	0	0	0	0	0	0	0	0	0	0	0	0	0	0	0	0	0	0	0	0	0	0	0	0	0	1	40.50864	20.56533
696	0	0	0	0	0	0	0	0	0	0	0	0	0	0	0	0	0	0	0	0	0	0	0	0	0	0	0	0	0	0	0	0	0	0	0	1	40.25182	19.96526
698	0	0	1	0	0	0	0	0	0	0	0	0	0	0	0	0	0	0	1	0	0	0	0	0	1	0	0	0	0	0	0	0	0	0	0	1	40.27581	20.09397
699	0	0	0	0	0	0	0	0	0	0	0	0	0	0	0	0	0	0	0	0	0	0	0	0	0	0	0	0	0	0	0	0	0	0	0	2	40.28722	20.08328
700	0	0	0	0	0	0	0	0	0	0	0	0	0	0	0	0	0	0	0	0	0	0	0	0	0	0	0	0	0	0	0	0	0	0	0	1	40.34056	19.78027
701	0	0	0	0	0	0	0	0	0	0	0	0	0	0	0	0	0	0	0	0	0	0	0	0	0	0	0	0	0	0	0	0	0	0	0	2	40.33741	19.77468
705	0	0	0	0	0	0	0	0	0	0	0	0	0	0	0	0	0	0	1	0	0	0	0	0	0	0	0	0	0	0	0	0	0	0	0	1	40.50051667	20.28601667
706	0	0	0	0	0	0	0	0	0	0	0	0	0	0	0	0	0	0	0	0	0	0	0	0	0	0	0	0	0	0	0	0	0	0	0	1	40.51638333	20.40703333
707	0	0	0	0	0	0	0	0	0	0	0	0	0	0	0	0	0	0	0	0	0	0	0	0	0	0	0	0	0	0	0	0	0	0	0	1	40.51776667	20.4124
708	0	0	0	0	0	0	0	0	0	0	0	0	0	0	0	0	0	0	1	0	0	0	0	0	0	0	0	0	0	0	0	0	0	0	0	1	40.5219	20.41388333
709	0	0	0	0	0	0	0	0	0	0	0	0	0	0	0	0	0	0	0	0	0	0	0	0	0	0	0	0	0	0	0	0	0	0	0	1	40.52921667	20.4192
710	0	0	0	1	1	0	0	0	0	0	0	0	0	0	0	0	0	0	0	0	0	0	0	0	0	0	0	0	0	0	1	0	0	0	0	1	40.53245	20.42563333
711	0	0	0	0	0	0	0	0	0	0	0	0	0	0	0	0	0	0	1	0	0	0	0	0	0	0	0	0	0	0	0	0	0	0	0	1	40.52453333	20.41633333
713	0	0	0	0	1	0	0	0	0	0	0	0	0	0	0	0	0	0	1	0	0	0	0	1	0	0	0	0	0	0	0	0	0	0	0	1	40.54348333	20.27181667
714	0	0	0	0	0	0	0	0	0	0	0	0	0	0	0	0	0	0	1	0	0	0	0	0	0	0	0	0	0	0	0	0	0	0	0	1	40.5303	20.25753333
715	0	1	0	0	1	0	0	0	0	0	0	0	0	0	0	0	0	1	1	0	0	0	0	1	0	0	0	0	0	0	0	0	0	0	0	1	40.5223	20.2574
717	0	0	0	0	0	0	0	0	0	0	0	0	0	0	0	0	0	0	0	0	0	0	0	0	0	0	0	0	0	0	0	0	0	0	0	2	40.54218333	20.346
718	0	0	0	0	1	0	0	0	0	0	0	0	0	0	0	0	0	0	1	0	0	0	0	1	0	0	0	0	0	0	0	0	0	0	0	1	40.559	20.39083333
719	0	0	0	0	0	0	0	0	0	0	0	0	0	0	0	0	0	0	0	0	0	0	0	1	0	0	0	0	0	0	0	0	0	0	0	1	40.57145	20.28798333
720	0	0	1	0	0	0	0	0	0	0	0	0	0	0	0	0	0	0	1	0	0	0	0	0	0	0	0	0	0	0	0	0	0	1	0	1	40.63281667	20.21615
721	0	0	0	0	0	0	0	0	0	0	0	0	0	0	0	0	0	0	0	0	0	0	0	0	0	0	0	0	0	0	0	0	0	1	0	1	40.64113333	20.15746667
722	0	0	0	0	0	0	0	0	0	0	0	0	0	0	0	0	0	0	0	0	0	0	0	0	0	0	0	0	0	0	0	0	0	0	0	1	40.6186	20.19908333
723	0	0	0	0	0	0	0	0	0	0	0	0	0	0	0	0	0	0	0	1	0	0	0	0	0	0	0	0	0	0	0	0	0	0	0	1	40.68131667	20.18348333
724	0	1	0	0	1	0	0	0	0	0	0	0	0	0	0	0	0	0	0	0	0	0	0	0	0	0	0	0	0	0	0	0	0	1	0	1	40.7127	20.18391667
725	0	0	0	0	1	0	0	0	0	0	0	0	0	0	0	0	0	0	1	0	0	0	0	0	0	0	0	0	0	0	0	0	0	0	0	1	40.72125	20.17201667
727	0	1	1	0	0	0	0	0	0	0	0	0	0	0	0	0	0	0	1	0	0	0	0	0	0	0	0	0	0	0	0	0	0	1	0	1	40.71638333	20.15671667
730	0	0	0	0	1	1	0	0	0	0	0	0	0	0	0	0	0	0	0	0	0	0	0	0	0	0	0	0	0	0	0	0	0	0	1	1	40.92603333	20.22316667
734	1	0	1	0	0	0	0	0	0	0	1	0	0	0	0	0	0	0	0	0	0	0	0	0	0	0	0	1	0	0	0	0	0	0	0	1	42.32533333	19.36275
739	0	0	0	0	0	0	0	0	0	1	0	0	0	0	0	0	0	0	0	1	0	0	0	0	0	0	0	0	0	0	0	0	0	0	0	1	41.760278	20.669722
744	0	0	0	0	0	0	0	0	0	0	0	0	0	0	0	0	0	0	0	0	0	0	0	0	0	0	0	0	0	0	0	0	0	0	0	3	40.976639	20.794083
748	0	0	0	0	0	0	0	0	0	0	0	0	0	0	0	0	0	0	0	0	0	0	0	0	0	0	0	0	0	0	0	0	0	0	0	1	41.843083	22.505917
750	0	0	0	0	0	0	0	0	0	0	0	0	0	0	0	0	0	0	0	0	0	0	0	0	0	0	1	0	0	0	0	0	0	0	0	1	40.82548	20.85281
751	0	0	0	0	0	0	0	0	0	0	0	0	0	0	0	0	0	0	0	0	0	0	0	0	0	0	0	0	0	0	0	0	0	0	0	1	40.8179	20.84941
752	0	0	0	0	0	0	0	0	0	0	0	0	0	0	0	0	0	0	0	0	0	0	0	0	0	0	0	0	0	0	0	0	0	0	0	1	40.81426	20.84022
753	0	0	0	0	0	0	0	0	0	0	0	0	0	0	0	0	0	0	0	0	0	0	0	0	0	1	0	0	0	0	0	0	0	0	0	1	40.80241	20.87408
754	0	0	0	0	0	0	0	0	0	0	0	0	0	0	0	0	0	0	0	0	0	0	0	0	0	0	1	0	0	0	0	0	0	0	0	1	40.81832	20.87283
755	0	0	0	0	0	0	0	0	0	0	0	0	0	0	0	0	0	0	0	0	0	0	0	0	0	0	1	0	0	0	0	0	0	0	0	1	40.82203	20.87886
757	0	0	0	0	0	0	0	0	0	0	0	0	0	0	0	0	0	0	0	0	0	0	0	0	0	0	1	0	0	0	0	0	0	0	0	1	40.74191	20.86351
758	0	0	0	1	0	0	0	0	0	0	0	0	0	0	0	0	0	0	0	0	0	0	0	0	0	0	1	0	0	0	0	0	0	0	0	1	40.7565	20.84945
759	0	0	0	0	0	0	0	0	0	0	0	0	0	0	0	0	0	0	1	0	0	0	0	0	0	0	0	0	0	0	0	0	0	0	0	1	40.53767	20.57924
760	0	0	0	0	0	0	0	0	0	0	0	0	0	0	0	0	0	1	0	0	0	0	0	0	0	0	0	0	0	0	0	0	0	0	0	1	42.3162	18.93421667
761	0	0	0	0	0	0	0	0	0	0	1	0	0	0	0	0	0	0	0	0	0	0	0	0	0	0	0	0	0	0	0	0	0	0	0	1	42.27805556	18.86
763	0	0	0	0	0	0	0	0	0	0	0	0	0	0	0	0	0	0	0	0	0	0	0	0	0	0	0	0	0	0	0	0	0	0	0	1	42.20878333	20.7512
764	0	0	0	0	0	0	0	0	0	0	0	0	0	0	0	0	0	0	0	0	0	0	0	0	0	0	0	0	0	0	0	0	0	0	0	1	42.06631667	20.48795
765	0	1	0	0	0	0	0	0	0	0	0	0	0	0	0	0	0	0	0	0	0	0	0	0	0	0	0	0	0	0	0	0	0	0	0	1	42.05883333	20.50181667
767	0	0	0	0	0	0	0	0	0	0	0	0	0	0	0	0	0	0	0	0	0	0	0	0	0	0	0	0	0	0	0	0	0	0	0	1	41.98666667	20.52858333
774	0	0	0	0	0	0	0	0	0	0	0	0	0	0	0	0	0	0	0	0	0	0	0	0	0	0	0	0	0	0	0	0	0	0	0	2	41.99465	20.48498333
775	0	0	0	0	0	0	0	0	0	0	0	0	1	0	0	0	0	0	0	0	0	0	0	0	0	0	0	0	0	0	0	0	0	0	0	1	41.99895	20.47168333
776	0	0	0	0	0	0	0	0	0	0	0	0	1	0	0	0	0	0	0	0	0	0	0	0	0	0	0	0	0	0	0	0	0	0	0	1	41.99931667	20.46133333
777	0	0	0	0	0	0	0	0	0	1	0	0	1	0	0	0	0	0	0	0	0	0	0	0	0	0	0	0	0	0	0	0	0	0	0	3	41.99956667	20.45773333
779	0	0	0	0	0	0	0	0	0	0	0	0	0	0	0	0	0	0	0	0	0	0	0	0	0	0	0	0	0	0	0	0	0	0	0	2	42.00946667	20.46965
780	0	0	0	0	0	0	0	0	0	0	0	0	0	0	0	0	0	0	0	0	0	0	0	0	0	0	0	0	0	0	0	0	0	0	0	2	42.01208333	20.46965
783	0	1	0	0	0	0	0	0	0	0	0	0	1	0	0	0	0	0	0	0	0	0	0	1	0	0	0	0	0	0	0	0	0	0	0	1	41.98971667	20.4202
784	0	0	0	0	0	0	0	0	0	0	0	0	0	0	0	0	0	0	0	0	0	0	0	0	0	0	0	0	0	0	0	0	0	0	1	1	41.92596667	20.38706667
785	0	0	0	0	0	0	0	0	0	0	0	0	0	0	0	0	0	0	0	0	0	0	0	0	0	0	0	0	0	0	0	0	0	0	0	1	41.9097	20.409
786	0	1	0	0	0	0	0	0	0	0	0	0	0	0	0	0	0	0	1	0	0	0	1	0	0	0	0	0	0	0	0	0	0	0	1	1	41.89498333	20.41733333
789	0	0	0	0	0	0	0	0	0	0	0	0	0	0	0	0	0	0	0	0	0	0	0	0	0	0	0	0	0	0	0	0	0	0	0	1	41.81885	20.50266667
790	0	0	0	1	0	0	0	0	0	0	0	0	0	0	0	0	0	0	0	0	0	0	0	0	0	0	0	0	0	0	0	0	0	0	0	1	41.8179	20.52701667
792	0	0	0	1	0	0	0	0	0	0	0	0	0	0	0	0	0	0	0	0	0	0	0	0	0	0	0	0	0	0	0	0	0	0	0	1	41.81868333	20.53733333
793	0	0	0	0	0	0	0	0	0	0	0	0	0	0	0	0	0	0	0	0	0	0	0	0	0	0	0	0	0	0	0	0	0	0	0	1	41.8052	20.56088333
795	0	0	0	0	0	0	0	0	0	0	0	0	0	0	0	0	0	0	0	0	0	0	0	0	0	0	0	0	0	0	0	0	0	0	0	1	41.80008333	20.54993333
796	0	0	0	0	0	0	0	0	0	0	0	0	0	0	0	0	0	0	0	0	0	0	0	0	0	0	0	0	0	0	0	0	0	0	0	1	41.79526667	20.54228333
797	0	0	0	0	0	0	0	0	0	0	0	0	0	0	0	0	0	0	0	0	0	0	0	0	0	0	0	0	0	0	0	0	0	0	0	2	41.79335	20.5439
798	0	0	0	0	0	0	0	0	0	0	0	0	0	0	0	0	0	0	0	0	0	0	0	0	0	0	0	0	0	0	0	0	0	0	0	1	41.79705	20.54536667
803	0	0	0	0	0	0	0	0	0	0	0	0	0	0	0	0	0	0	0	0	0	0	0	0	0	0	0	0	0	0	0	0	0	0	0	1	41.81535	20.54485
805	0	0	0	1	0	0	0	0	0	0	0	0	0	0	0	0	0	0	0	0	0	0	0	0	0	0	0	0	0	0	0	0	0	0	0	1	41.81915	20.52173333
810	0	0	0	0	0	0	0	0	0	0	0	0	0	0	0	0	0	0	0	0	0	0	0	0	0	0	0	0	0	0	0	0	0	0	0	1	41.76893333	20.27673333
813	0	1	0	0	0	0	0	0	0	0	0	0	0	0	0	0	0	0	1	0	0	0	0	0	0	0	0	0	0	0	0	0	0	0	0	1	41.81365	20.23618333
817	0	1	0	0	0	0	0	0	0	0	0	0	0	0	0	0	0	0	0	0	0	0	0	0	0	0	0	0	0	0	0	0	0	0	0	1	41.83818333	20.16646667
819	1	0	0	0	0	0	0	0	0	0	0	0	0	0	0	0	0	0	0	0	0	0	0	0	0	0	0	0	0	0	1	0	0	0	0	1	41.50988333	20.31515
823	0	1	0	0	0	0	0	0	0	0	0	0	0	0	0	0	0	0	1	1	0	0	0	0	0	0	0	0	0	0	1	0	0	0	0	1	41.43065	20.09196667
828	0	0	0	0	0	0	0	0	0	0	0	0	0	0	0	0	0	0	1	0	0	0	0	0	0	0	0	0	0	0	0	0	0	0	0	1	41.32106667	20.14736667
831	0	0	0	0	0	0	0	0	0	0	0	0	1	0	0	0	0	0	1	0	0	0	0	0	0	0	0	0	0	0	0	0	0	0	0	1	40.5378	20.56996667
836	0	0	0	1	0	0	0	0	0	0	0	0	0	0	0	0	0	0	0	0	0	0	0	0	0	0	1	0	0	0	0	0	0	0	0	1	40.79151667	20.9324
838	0	0	0	0	0	0	0	0	0	0	0	0	0	0	0	0	0	0	0	0	0	0	0	1	0	0	0	0	0	0	0	0	0	0	0	1	40.26878333	20.6065
841	0	0	0	0	1	1	0	0	0	0	0	0	0	0	0	0	0	1	1	0	0	0	0	0	0	0	0	0	0	0	0	0	0	0	0	1	40.24463333	20.4377
843	0	1	0	0	0	1	0	0	0	0	0	0	0	0	0	0	0	0	0	0	0	0	0	0	0	0	0	0	0	0	0	0	0	0	0	1	40.29883333	20.0886
844	0	0	0	0	0	0	0	0	0	0	0	0	0	0	0	0	0	0	1	0	0	0	0	0	0	0	0	0	0	0	0	0	0	0	0	1	40.86643	20.42862
845	0	0	1	0	0	0	0	0	0	0	0	0	0	0	0	0	0	0	0	0	0	0	0	0	0	0	0	0	0	0	0	0	0	0	0	1	40.84757	20.11453
846	0	1	0	0	0	0	0	0	0	0	0	0	0	0	0	0	0	1	1	0	0	0	0	0	0	0	0	0	0	0	0	0	0	0	0	1	40.0727	20.13916667
848	0	0	0	0	0	0	0	0	0	0	0	0	0	0	0	0	0	0	0	0	0	0	0	0	0	0	0	0	0	0	0	0	0	0	0	1	39.74535	20.01955
850	0	0	0	0	0	0	0	0	0	0	0	0	0	0	0	0	0	0	0	0	0	0	0	0	0	0	0	0	0	0	0	0	0	0	0	1	40.0808	19.76746667
854	0	0	1	0	0	0	0	0	0	0	0	0	0	0	0	1	0	0	0	0	0	0	0	0	0	0	0	0	0	0	0	0	0	0	0	1	41.37341667	19.91983333
855	0	0	0	0	0	0	0	0	0	0	0	0	0	0	0	1	0	0	0	0	0	0	0	0	0	0	0	0	0	0	0	0	0	0	0	3	41.37311667	19.92003333
856	0	0	1	0	0	0	0	0	0	0	0	0	0	0	0	1	0	0	1	1	0	0	0	0	0	0	0	0	0	0	0	0	0	0	0	3	41.3689	19.9221
864	0	0	0	0	0	0	0	0	0	0	0	0	0	0	0	0	0	0	0	0	0	0	0	0	0	0	0	0	0	0	0	1	0	0	0	1	41.24978	20.5044
865	0	0	0	0	0	0	0	0	0	0	0	0	0	0	0	1	0	0	0	0	0	0	0	0	0	0	0	0	0	0	0	0	0	0	0	1	42.51425	18.48736667
866	0	0	1	0	0	0	0	0	0	0	0	0	0	0	0	1	0	0	0	0	0	0	0	0	0	0	0	0	0	0	0	0	0	0	0	1	42.52776667	18.4875
867	0	0	0	0	0	0	0	0	0	0	0	0	0	0	0	0	0	0	0	0	0	0	0	0	0	0	0	0	0	0	0	0	0	0	0	3	42.54983333	18.65491667
868	0	0	0	0	0	0	0	0	0	0	0	0	0	0	0	0	0	0	0	0	0	0	0	0	0	0	0	0	0	0	0	0	0	0	0	1	42.52365	18.68546667
871	0	1	0	0	1	0	0	0	0	0	0	0	0	0	0	0	0	1	0	0	0	0	0	0	0	0	0	0	1	0	0	0	0	0	0	1	42.48616667	18.76703333
872	0	1	0	0	1	0	0	0	0	0	1	0	0	0	0	0	0	1	0	0	0	0	0	0	0	0	0	0	1	0	0	0	0	0	0	1	42.40241667	18.77496667
873	0	0	0	0	0	0	0	0	0	0	0	0	0	0	0	0	0	0	0	0	0	0	0	0	0	0	0	0	0	0	0	0	0	0	0	1	42.40036667	18.79401667
874	0	0	1	0	0	0	0	0	0	0	0	0	0	0	0	0	0	0	0	0	0	0	0	0	0	0	0	0	0	0	0	0	0	0	0	3	42.3999	18.83136667
875	0	0	0	0	0	0	0	0	0	0	0	0	0	0	0	0	0	0	0	0	0	0	0	0	0	0	0	0	0	0	0	0	0	0	0	2	42.3989	18.83808333
876	0	0	0	0	0	0	0	0	0	0	0	0	0	0	0	0	0	0	0	0	0	0	0	0	0	0	0	0	0	0	0	0	0	0	0	1	42.39963333	18.83871667
877	0	0	0	0	0	0	0	0	0	0	0	0	0	0	0	0	0	0	0	0	0	0	0	0	0	0	0	0	0	0	0	0	0	0	0	2	42.39503333	18.80905
880	0	0	0	0	1	0	0	0	0	0	0	0	0	0	0	0	0	0	0	0	0	0	0	0	0	0	0	0	0	0	0	0	0	0	0	1	42.39938333	18.78271667
882	0	0	0	0	0	0	0	0	0	0	0	0	0	0	0	0	0	1	0	0	0	0	0	0	0	0	0	0	0	0	0	0	0	0	0	3	42.60271667	18.67483333
883	0	0	0	0	0	0	0	0	0	0	0	0	0	0	0	0	0	0	0	0	0	0	0	0	0	0	0	0	0	0	0	0	0	0	0	1	42.65231667	18.67201667
884	0	0	0	0	0	0	0	0	0	0	1	0	0	0	0	0	0	0	0	0	0	0	0	0	0	0	0	0	0	0	0	0	0	0	0	3	42.67763333	18.64191667
885	0	0	0	0	0	0	0	0	0	0	1	0	0	0	0	0	0	0	0	0	0	0	0	0	0	0	0	0	0	0	0	0	0	0	0	3	42.71938333	18.60241667
886	0	0	0	0	0	0	0	0	0	0	0	0	0	0	0	0	0	0	0	0	0	0	0	0	0	0	0	0	0	0	0	0	0	0	0	1	42.76236667	18.82618333
888	0	0	0	0	0	0	0	0	0	0	1	0	0	0	0	0	0	0	0	0	0	0	0	0	0	0	0	0	0	0	0	0	0	0	0	2	42.85776667	18.94326667
889	0	0	1	0	0	0	0	0	0	1	0	0	0	0	0	0	0	0	0	0	0	0	0	0	0	0	0	0	0	0	0	0	0	0	0	3	42.9017	19.02098333
890	0	0	0	1	0	0	0	0	0	0	1	0	1	0	0	0	0	0	0	0	0	0	0	0	0	0	0	0	0	0	0	0	0	0	0	1	42.89493333	19.07943333
891	0	0	0	0	0	0	0	0	0	1	1	0	0	0	0	0	0	0	0	0	0	0	0	0	0	0	0	0	0	0	0	0	0	0	0	1	42.94763333	19.09105
892	0	1	1	1	0	0	0	0	0	0	0	0	1	0	0	0	0	0	0	0	0	0	0	0	0	0	0	0	0	0	0	0	0	0	0	1	42.94975	19.0993
893	0	0	0	0	0	0	0	0	0	0	1	0	0	0	0	0	0	0	0	0	0	0	0	0	0	0	0	0	0	0	0	0	0	0	0	1	42.9622	19.09308333
894	0	0	0	1	0	0	0	0	0	0	0	0	1	0	0	0	0	0	0	0	0	0	0	0	0	0	0	0	0	0	0	0	0	0	0	1	42.95765	19.0959
895	0	0	0	0	0	0	0	0	0	0	0	0	0	0	0	0	0	0	0	0	0	0	0	0	0	0	0	0	0	0	0	0	0	0	0	2	42.98756667	19.0668
896	0	0	1	1	0	0	0	0	0	0	1	0	1	0	0	0	0	0	0	0	0	0	0	0	0	0	0	0	0	0	0	0	0	0	0	1	42.9883	19.06783333
897	0	0	0	0	0	0	0	0	0	0	1	0	1	0	0	0	0	0	0	0	0	0	0	0	0	0	0	0	0	0	0	0	0	0	0	1	42.98156667	19.06833333
898	0	0	1	0	0	0	0	0	0	0	0	0	1	0	0	0	0	0	0	0	0	0	0	0	0	0	0	0	0	0	0	0	0	0	0	1	42.98718333	19.07993333
899	0	0	0	0	0	0	0	0	0	0	0	0	0	0	0	0	0	0	0	0	0	0	0	0	0	0	0	0	0	0	0	0	0	0	0	1	42.9507	19.1735
903	0	1	0	0	0	0	0	0	0	0	1	0	1	0	0	0	0	0	0	0	0	0	0	0	0	0	0	0	0	0	0	0	0	0	0	1	42.90093333	19.27321667
904	0	0	0	0	0	0	0	0	0	1	1	0	0	0	0	0	0	0	0	0	0	0	0	0	0	0	0	0	0	0	0	0	0	0	0	1	42.88223333	19.29768333
905	0	0	0	0	0	0	0	0	0	0	1	0	0	0	0	0	0	0	0	0	0	0	0	0	0	0	0	0	0	0	0	0	0	0	0	1	42.84868333	19.33448333
906	0	0	0	0	0	0	0	0	0	0	0	0	0	0	0	0	0	0	0	0	0	0	0	0	0	0	0	0	0	0	0	0	0	0	0	1	42.81903333	19.38101667
908	0	0	0	0	0	0	0	0	0	0	0	0	1	0	0	0	0	0	0	0	0	0	0	0	0	0	0	0	0	0	0	0	0	0	0	1	42.88206667	19.39978333
909	0	0	0	1	0	0	0	0	0	0	0	0	1	0	0	0	0	0	0	0	0	0	0	0	0	0	0	0	0	0	0	0	0	0	0	1	42.90301667	19.38221667
910	0	0	0	0	0	0	0	0	0	0	0	0	1	0	0	0	0	0	0	0	0	0	0	0	0	0	0	0	0	0	0	0	0	0	0	1	42.89715	19.38566667
911	0	0	0	0	0	0	0	0	0	0	0	0	0	0	0	0	0	0	0	0	0	0	0	0	0	0	0	0	0	0	0	0	0	0	0	3	42.89151667	19.38918333
912	0	0	0	0	0	0	0	0	0	1	0	0	1	0	0	0	0	0	0	0	0	0	0	0	0	0	0	0	0	0	0	0	0	0	0	1	42.8722	19.47948333
915	0	0	0	0	0	0	0	0	0	1	0	0	0	0	0	0	0	0	0	0	0	0	0	0	0	0	0	0	0	0	0	0	0	0	0	2	42.89495	19.64246667
916	0	0	0	0	0	0	0	0	0	0	0	0	0	0	0	0	0	0	0	0	0	0	0	0	0	0	0	0	0	0	0	0	0	0	0	2	42.88933333	19.65971667
917	0	0	0	0	0	0	0	0	0	0	0	0	0	0	0	0	0	0	0	0	0	0	0	0	0	0	0	0	0	0	0	0	0	0	0	1	42.87968333	19.74111667
919	0	0	0	1	0	0	0	0	0	1	0	0	0	0	0	0	0	0	0	0	0	0	0	0	0	0	0	0	0	0	0	0	0	0	0	1	42.51173333	19.8348
921	0	0	0	1	0	0	0	0	0	1	1	0	1	0	0	0	0	0	0	0	0	0	0	0	0	0	0	0	0	0	0	0	0	0	0	1	42.4907	19.81885
922	0	0	0	0	0	0	0	0	0	0	0	0	1	0	0	0	0	0	0	0	0	0	0	0	0	0	0	0	0	0	0	0	0	0	0	1	42.49095	19.81231667
923	0	0	0	0	0	0	0	0	0	0	1	0	0	0	0	0	0	0	0	0	0	0	0	0	0	0	0	0	0	0	0	0	0	0	0	2	42.50163333	19.82456667
924	0	0	0	1	0	0	0	0	0	0	0	0	0	0	0	0	0	0	0	0	0	0	0	0	0	0	0	0	0	0	0	0	0	0	0	1	42.6337	19.85008333
929	0	0	1	1	0	0	0	0	0	0	0	0	1	0	0	0	0	0	0	0	0	0	0	0	0	0	0	0	0	0	0	0	0	0	0	1	42.67545	19.52291667
930	0	0	1	1	0	0	0	0	0	0	1	0	1	0	0	0	0	0	0	0	0	0	0	0	0	0	0	0	0	0	0	0	0	0	0	1	42.61888333	19.55368333
932	0	0	0	1	0	0	0	0	0	0	1	0	1	0	0	0	0	0	0	0	0	0	0	0	0	0	0	0	0	0	0	0	0	0	0	1	42.56941667	19.6025
933	0	0	0	0	0	0	0	0	0	0	0	0	0	0	0	0	0	0	0	0	0	0	0	0	0	0	0	0	0	0	0	0	0	0	0	3	42.57495	19.59783333
934	0	0	1	1	0	0	0	0	0	0	1	0	1	0	0	0	0	0	0	0	0	0	0	0	0	0	0	0	0	0	0	0	0	0	0	1	42.57885	19.59216667
935	0	0	0	1	0	0	0	0	0	0	1	0	0	0	0	0	0	0	0	0	0	0	0	0	0	0	0	0	0	0	0	0	0	0	0	1	42.59308333	19.5801
936	0	1	0	0	0	0	0	0	0	0	1	0	0	0	0	0	0	0	0	0	0	0	0	0	0	0	0	0	0	0	0	0	0	0	0	1	42.59936667	19.51166667
937	0	0	1	0	0	0	0	0	0	0	0	0	0	0	0	0	0	0	0	0	0	0	0	0	0	0	0	0	0	0	0	0	0	0	0	1	42.54876667	19.45435
938	0	0	1	0	0	0	0	0	0	0	1	0	0	0	0	0	0	0	0	0	0	0	0	0	0	0	0	0	0	0	0	0	0	0	0	1	42.51081667	19.38476667
939	0	0	0	0	0	0	0	0	0	0	1	0	0	0	0	0	0	0	0	0	0	0	0	0	0	0	0	0	0	0	0	0	0	0	0	1	42.45411667	19.35115
940	0	0	0	0	0	0	0	0	0	0	1	0	0	0	0	0	0	0	0	0	0	0	0	0	0	0	0	1	0	0	0	0	0	0	0	1	42.1924	19.107
941	0	1	0	0	0	0	0	0	0	0	0	0	0	0	0	0	0	0	0	0	0	0	0	0	0	0	0	0	1	0	0	0	0	0	0	1	42.09386667	19.13423333
942	0	0	0	0	0	0	0	0	0	0	1	0	0	0	0	1	0	1	0	0	0	0	0	0	0	0	0	0	1	0	0	0	0	0	0	1	42.10043333	19.1419
943	0	0	0	0	0	0	0	0	0	0	0	0	0	0	0	0	0	0	0	0	0	0	0	0	0	0	0	0	1	0	0	0	0	0	0	1	42.09726667	19.14583333
944	0	0	0	0	0	0	0	0	0	0	0	0	0	0	0	1	0	1	0	0	0	0	0	0	0	0	0	0	1	0	0	0	0	0	0	1	42.0978	19.14466667
945	0	0	0	0	0	0	0	0	0	0	0	0	0	0	0	0	0	0	0	0	0	0	0	0	0	0	0	0	1	0	0	0	0	0	0	1	42.09965	19.14263333
946	0	0	0	1	0	0	0	0	0	0	1	0	0	0	0	0	0	0	0	0	0	0	0	0	0	0	0	1	0	0	0	0	0	0	0	1	42.21876667	19.0246
948	0	0	0	1	0	0	0	0	0	0	0	0	1	0	0	0	0	0	0	0	0	0	0	0	0	0	0	0	0	0	0	0	0	0	0	1	43.07073333	19.86145
951	0	0	0	0	0	0	0	0	0	0	0	0	0	0	0	0	0	1	0	0	0	0	0	0	0	0	0	0	0	0	0	0	0	0	0	1	41.69387	19.821554
952	0	1	0	0	0	0	0	0	0	0	0	0	0	0	0	0	0	1	0	0	0	0	0	0	0	0	0	0	0	0	0	0	0	0	0	1	41.63089	19.73321
954	0	0	0	0	0	0	0	0	0	0	0	0	0	0	0	0	0	1	0	0	0	0	0	0	0	0	0	0	0	0	0	0	0	0	0	1	40.16149	19.65018
955	0	0	0	0	0	0	0	0	0	0	0	0	0	0	0	0	0	1	0	0	0	0	0	0	0	0	0	0	0	0	0	0	0	0	0	1	39.77261	20.07333

956		0	1	39.82265	20.06533
957		0	1	39.75738	20.08182
959		0	1	42.34298	20.03205
960		0	1	42.36478	20.01736
961		0	2	42.34867	19.99474
962		0	1	42.35199	19.99302
963		0	1	42.46327	19.88637
964		0	2	42.44961	20.13148
965		0	2	42.46322	20.12577
966		0	2	42.4678	20.12229
969	2009/123	0	1	43.8713	20.11421667
970	2009/124	0	1	43.90175	20.19268333
974	2009/128	0	1	43.72348333	21.9478
975	2009/129	0	1	43.7957	21.90348333
977		1	1	41.37773	20.03291
978		0	2	41.68776	20.15676
979		0	3	41.68861	20.15571
980		0	1	41.69076	20.14681
981		0	1	41.69025	20.16586
982		0	1	40.18113	20.66172
983		0	1	39.86484	20.02188
984		0	1	39.68221	20.12899
985		0	1	40.129	19.67311
986		0	1	42.29318	19.53328
996	2010/009	1	1	41.62558333	20.24983333
997	2010/010	0	1	41.64651667	20.19013333
998	2010/011	0	1	41.66373333	20.19533333
999	2010/012	0	1	41.66508333	20.20828333
1001	2010/013B	0	1	41.67465	20.21115
1003	2010/BP1	0	2	41.67671	20.2135
1004	2010/015	0	1	41.69623333	20.13331667
1005	2010/016	0	1	41.69708333	20.13618333
1008	2010/BP2	0	1	41.69194	20.21288
1009	2010/BP4	0	1	41.68977	20.25569
1010	2010/BP5	0	1	41.69413	20.2618
1012	2010/020	0	1	41.81346667	20.13973333
1024	2010/032	0	1	41.84231667	20.09451667
1025	2010/033	0	1	41.85056667	20.09736667
1026	2010/034	0	1	41.85456667	20.1217
1028	2010/036	0	1	41.86923333	20.11863333
1029	2010/037	0	1	41.87066667	20.1085
1030	2010/038	0	1	42.13848333	20.49005
1031	2010/039	0	3	42.19235	20.53763333
1032	2010/040	0	3	42.20076667	20.5333
1033	2010/041	0	3	42.20285	20.53378333
1034	2010/042	0	2	42.20695	20.52848333
1035	2010/043	0	1	42.11456667	19.57471667
1036	2010/044	0	1	42.12485	19.6037
1037	2010/045	0	1	42.14171667	19.64743333
1038	2010/046	0	1	42.16498333	19.69515
1039	2010/047	0	1	42.17863333	19.72008333
1040	2010/048	0	1	42.207	19.70906667
1041	2010/049	0	1	42.21423333	19.70581667
1042	2010/050	0	1	42.24528333	19.71691667
1043	2010/051	0	1	42.24036667	19.72803333
1045	2010/053	0	1	42.25136667	19.74788333
1046	2010/054	0	1	42.28976667	19.79683333
1047	2010/055	0	1	42.3043	19.7969
1048	2010/056	0	3	42.40128333	19.7658
1049	2010/BP8	0	3	41.99294	20.4267
1050	2010/BP9	0	2	42.00798333	20.44608333
1051	2010/057	0	3	42.40161667	19.7577
1055	2010/061	0	1	43.21303333	19.76305
1056	2010/062	0	1	43.61168333	19.72516667
1057	2010/BP12	0	1	41.97755	20.11537
1058		0	1	42.445683	19.756067
1059		0	1	42.419	19.64347
1060		0	1	42.42166	19.67168
1061		0	1	42.43077	19.69267
1062		0	1	42.45781	19.71515
1063		0	1	42.45572	19.77465
1064		0	1	42.46108	19.71596
1065		0	2	39.70188333	20.89465
1066		0	2	39.72825	21.0839
1068		0	1	39.177167	20.734383
1069		0	1	38.722717	20.690067
1071		0	1	41.31676667	20.3774
1072		0	1	40.8665	20.377333
1073		0	1	40.08	19.954
1076	2012/103	0	1	43.18343333	19.76168333
1077	2012/104	0	3	43.02753333	19.40143333
1082	2012/109	0	1	42.40636667	19.34983333
1092	2012/119	0	3	42.22075	19.11175
1093	2012/120	0	1	42.16773333	19.19595
1095	2012/122	0	1	41.97678333	19.33868333
1104	2012/131	0	1	42.1997	19.81868333
1105	2012/132	0	1	42.19455	19.81771667
1106	2012/133	0	1	42.1892	19.83275
1108	2012/135	0	1	42.23386667	19.87413333
1110	2012/137	0	1	42.2652	19.91791667
1113	2012/140	0	1	42.2279	19.90705
1114	2012/141	0	1	42.10865	19.8264
1119	2012/146	0	1	41.36931667	19.98625
1120	2012/147	0	1	41.36701667	20.0196
1121	2012/148	1	1	41.3512	20.05021667
1122	2012/149	1	1	41.35073333	20.05826667
1125	2012/151	1	1	41.37053333	20.08546667
1126	2012/152	0	3	41.33555	20.15028333
1127	2012/153	0	1	41.33453333	20.1636
1129	2012/155	0	1	41.33923333	20.19886667
1130	2012/156	0	1	41.36035	20.2412
1134	2012/160	0	1	41.38821667	20.26055
1135	2012/161	0	1	41.37555	20.2686
1136	2012/162	1	1	41.33925	20.12878333
1138	2012/164	0	1	41.103	20.00645
1139	2012/165	1	1	41.16343333	20.23005
1141		0	1	42.01468	19.62839
1142		0	1	40.848529	20.86242
1143		0	2	41.25053	20.50336
1144		0	1	41.47944	20.08145
1147	2013/001	0	1	41.4091788	22.2527529
1148	2013/002	0	1	39.9965353	21.2855131
1149	2013/003	0	1	40.0076964	21.2805204
1150	2013/004	0	1	40.0079535	21.2846782
1151	2013/005	0	1	40.0521286	21.2393935
1152	2013/006	0	1	40.0741143	21.2016805
1153	2013/007	0	1	39.4602844	21.6007841
1154	2013/008	0	1	39.4569065	21.59613
1155	2013/009	0	1	39.4428565	21.5103952
1156	2013/010	0	1	39.4278403	21.4299602
1157	2013/011	0	1	39.4613997	21.2938452
1158	2013/012	0	1	39.3969136	21.0169787
1159	2013/013	0	1	39.4279064	21.0105573
1160	2013/014A	0	1	39.5668484	21.1230829
1161	2013/014C	0	1	39.5680853	21.1313026
1162	2013/015	0	1	39.5767837	21.0969426
1163	2013/016	0	1	39.5728428	20.986106
1164	2013/017	0	1	39.5762953	20.9771778
1167	2013/020	0	1	39.689521	20.8713509
1168	2013/021	0	1	39.6870318	20.8740156
1169	2013/022	0	1	39.6848876	20.8795284
1170	2013/023	0	1	39.6828228	20.8886677
1171	2013/024	0	1	39.6801669	20.9052407
1172	2013/025	0	1	39.6947455	20.8463411
1173	2013/026	0	1	39.697139	20.8432918
1174	2013/027	0	1	39.7244725	20.8428578
1175	2013/028	0	1	39.7679699	20.6095984
1176	2013/029	0	2	39.7642158	20.616866
1177	2013/030	0	3	39.7670511	20.6147516
1178	2013/031	0	3	39.7720295	20.61797
1179	2013/032	0	1	39.7358415	20.4536566
1180	2013/033	0	1	39.8777481	20.4657989
1181	2013/034	0	1	39.890997	20.7538141
1182	2013/035	0	1	39.8950952	20.7413356
1183	2013/036	0	1	39.9067313	20.751873
1184	2013/037	0	1	39.862	20.7749
1185	2013/038	0	1	39.9674078	20.6645715
1186	2013/039	0	1	40.0467572	20.5648481
1187	2013/040	0	1	40.03549	20.74694
1188	2013/041	0	1	40.1102296	20.7202726
1192	2013/045	0	1	40.3090431	20.8980564
1193	2013/046	0	1	40.3114583	20.8986418
1194	2013/047	0	1	40.3130587	20.8994665
1196	2013/049A	0	1	40.4430603	21.4102248
1198	2013/050	0	1	40.3781789	21.4159985
1201	2013/053	0	1	40.5904817	21.1921266
1202	2013/054	0	1	40.6555158	21.1811746
1203	2013/055	0	1	40.7429279	21.1101852
1205	2013/057	0	1	40.8315864	21.0286694
1207	2013/059	0	1	40.672265	20.9871246
1208	2013/060	0	1	40.7330062	20.87294
1209	2013/061	0	1	40.791891	20.9151703
1210	2013/062	0	1	40.7688301	20.8274532
1211	2013/063	1	1	40.9443849	20.6109453
1213	2013/065	0	1	41.0395881	20.6347365
1214	2013/066	0	1	41.0692523	20.6467607
1215	2013/067	0	2	41.0682353	20.6097743
1216		0	3	41.68	21.4399
1217		0	1	41.6827	21.4397
1218		0	1	40.3983	21.4143
1219		1	1	39.681	21.122
1221		0	1	40.684722	20.998417
1222		0	1	40.2967	20.9433
1224		0	1	40.2948	20.9381
1225		0	1	40.766806	20.927722
1226		0	1	40.3117	20.9072

1228	0	0	0	0	0	0	0	0	0	0	0	0	0	0	0	0	0	0	0	0	0	0	0	0	0	0	0	0	0	0	0	0	0	0	0	0	0	0	0	0	0	0	0	0	0	0	0	0	0	1	0	0	0	0	0	0	0	0	0	0	0	0	1	40.3449	20.8813
1229	0	0	0	0	0	0	0	0	0	0	0	0	0	1	1	0	0	0	0	0	0	0	0	0	0	0	0	0	0	0	0	0	0	0	0	0	0	0	0	0	0	0	0	0	0	0	0	0	0	0	0	0	1	0	0	0	0	0	0	0	0	0	1	40.9718	20.8788
1231	0	0	0	0	0	0	0	0	0	0	0	0	0	0	0	0	0	0	0	0	0	0	0	0	0	0	0	0	0	0	0	0	0	0	0	0	0	0	0	0	0	0	0	0	0	0	0	0	0	0	0	1	0	0	0	0	0	0	0	0	0	0	1	39.6986	20.8482
1234	0	0	0	0	0	0	0	0	0	0	0	0	0	0	0	0	0	0	0	0	0	0	0	0	0	0	0	0	0	0	0	0	0	0	0	0	0	0	0	0	0	0	0	0	0	0	0	0	0	0	0	1	0	0	0	0	0	0	0	0	0	0	1	39.72765	20.823
1236	0	0	0	0	0	0	0	0	0	0	0	0	0	0	0	0	0	0	0	0	0	0	0	0	0	0	0	0	0	0	0	0	0	0	0	0	0	0	0	0	0	0	0	0	0	0	0	0	0	0	0	1	0	0	0	0	0	0	0	0	0	0	1	39.7828	20.8041
1239	0	0	0	0	0	0	0	0	0	0	0	0	0	0	0	0	0	0	0	0	0	0	0	0	0	0	0	0	0	0	0	0	0	0	0	0	0	0	0	0	0	0	0	0	0	0	0	0	0	0	0	1	0	0	0	0	0	0	0	0	0	0	1	39.7345	20.7737
1240	0	0	0	0	0	0	0	0	0	0	0	0	0	0	0	0	0	0	0	0	0	0	0	0	0	0	0	0	0	0	0	0	0	0	0	0	0	0	0	0	0	0	0	0	0	0	0	0	0	0	1	0	0	0	0	0	0	0	0	0	0	0	1	39.9773	20.7644
1241	0	0	0	0	0	0	0	0	0	0	0	0	0	0	0	0	0	0	0	0	0	0	0	0	0	0	0	0	0	0	0	0	0	0	0	0	0	0	0	0	0	0	0	0	0	0	0	0	0	0	1	0	0	0	0	0	0	0	0	0	0	0	1	40.0706	20.7608
1252	0	0	0	0	0	0	0	0	0	0	0	0	0	0	0	0	0	0	0	0	0	0	0	0	0	0	0	0	0	0	0	0	0	0	0	0	0	0	0	0	0	0	0	0	0	0	0	0	0	0	0	0	0	0	0	0	0	0	0	0	0	1	1	41.2234	20.537
1253	0	0	0	0	0	0	0	0	0	0	0	0	0	0	0	0	0	0	0	0	0	0	0	0	0	0	0	0	0	0	0	0	0	0	0	0	0	0	0	0	0	0	0	0	0	0	0	0	0	0	0	0	0	0	0	0	0	0	0	0	0	1	1	41.203	20.532
1254	0	0	0	0	0	0	0	0	0	0	0	0	0	0	0	0	0	0	0	0	0	0	0	0	0	0	0	0	0	0	0	0	0	0	0	0	0	0	0	0	0	0	0	0	0	0	0	0	0	0	0	0	0	0	0	0	0	0	1	0	0	0	1	41.35749	20.46226
1256	0	0	0	0	0	0	0	0	0	0	0	0	0	0	0	0	0	0	0	0	0	0	0	0	0	0	0	0	0	0	0	0	0	0	0	0	0	0	0	0	0	0	0	0	0	0	0	0	0	0	0	1	0	0	0	0	0	0	0	0	0	0	1	39.7822	20.3465
1257	0	0	0	0	0	0	0	0	0	0	0	0	0	0	0	0	0	0	0	0	0	0	0	0	0	0	0	0	0	0	0	0	0	0	0	0	0	0	0	0	1	0	0	0	0	0	0	0	1	0	0	0	0	0	0	0	0	0	0	0	0	0	1	42.56	20.21
1260	0	0	0	0	0	0	0	0	0	0	0	0	0	0	0	0	0	0	0	0	0	0	0	0	0	0	0	0	0	0	0	0	0	0	0	0	0	0	0	0	0	0	0	0	0	0	0	0	0	0	0	0	0	0	1	0	0	0	0	0	0	0	1	42.05717	19.39625
1263	0	0	0	0	0	0	0	0	0	0	0	0	0	0	0	0	0	0	0	0	0	0	0	0	0	0	0	0	0	0	0	0	0	0	0	0	0	0	0	0	0	0	0	0	0	0	0	0	0	0	0	0	0	0	1	0	0	0	0	0	0	0	1	42.3539	19.3416
1264	0	0	0	0	0	0	0	0	0	0	0	0	0	0	0	0	0	0	0	0	0	0	0	0	0	0	0	0	0	0	0	0	0	0	0	0	0	0	0	0	0	0	0	0	0	0	0	0	0	0	0	0	0	0	1	0	0	0	0	0	0	0	1	42.0387	19.2673
1268	0	0	0	0	0	0	0	0	0	0	0	0	0	0	0	0	0	0	0	0	0	0	0	0	0	0	0	0	0	0	0	0	0	0	0	0	0	0	0	0	0	0	0	0	0	0	0	0	0	0	0	0	0	0	1	0	0	0	0	0	0	0	1	42.1099	19.2321
1269	0	0	0	0	0	0	0	0	0	0	0	0	0	0	0	0	0	0	0	0	0	0	0	0	0	0	0	0	0	0	0	0	0	0	0	0	0	0	0	0	0	0	0	0	0	0	0	0	0	0	0	0	0	0	1	0	0	0	0	0	0	0	1	42.1479	19.2191
1270	0	0	0	0	0	0	0	0	0	0	0	0	0	0	0	0	0	0	0	0	0	0	0	0	0	0	0	0	0	0	0	0	0	0	0	0	0	0	0	0	0	0	0	0	0	0	0	0	0	0	0	0	0	0	1	0	0	0	0	0	0	0	1	42.3132	19.1885
1271	0	0	0	0	0	0	0	0	0	0	0	0	0	0	0	0	0	0	0	0	0	0	0	0	0	0	0	0	0	0	0	0	0	0	0	0	0	0	0	0	0	0	0	0	0	0	0	0	0	0	0	0	0	0	1	0	0	0	0	0	0	0	1	42.1323	19.1434
1272	0	0	0	0	0	0	0	0	0	0	0	0	0	0	0	0	0	0	0	0	0	0	0	0	0	0	0	0	0	0	0	0	0	0	0	0	0	0	0	0	0	0	0	0	0	0	0	0	0	0	0	0	0	0	1	0	0	0	0	0	0	0	1	42.067	19.14
1273	0	0	0	0	0	0	0	0	0	0	0	0	0	0	0	0	0	0	0	0	0	0	0	0	0	0	0	0	0	0	0	0	0	0	0	0	0	0	0	0	0	0	0	0	0	0	0	0	0	0	0	0	0	0	1	0	0	0	0	0	0	0	1	42.1373	19.1398
1274	0	0	0	0	0	0	0	0	0	0	0	0	0	0	0	0	0	0	0	0	0	0	0	0	0	0	0	0	0	0	0	0	0	0	0	0	0	0	0	0	0	0	0	0	0	0	0	0	0	0	0	0	0	0	1	0	0	0	0	0	0	0	1	42.3917	19.133
1277	0	0	0	0	0	0	0	0	0	0	0	0	0	0	0	0	0	0	0	0	0	0	0	0	0	0	0	0	0	0	0	0	0	0	0	0	0	0	0	0	0	0	0	0	0	0	0	0	0	0	0	0	0	0	1	0	0	0	0	0	0	0	1	42.1512	19.1125
1278	0	0	0	0	0	0	0	0	0	0	0	0	0	0	0	0	0	0	0	0	0	0	0	0	0	0	0	0	0	0	0	0	0	0	0	0	0	0	0	0	0	0	0	0	0	0	0	0	0	0	0	0	0	0	1	0	0	0	0	0	0	0	1	42.1829	19.1091
1280	0	0	0	0	0	0	0	0	0	0	0	0	0	0	0	0	0	0	0	0	0	0	0	0	0	0	0	0	0	0	0	0	0	0	0	0	0	0	0	0	0	0	0	0	0	0	0	0	0	0	0	0	0	0	1	0	0	0	0	0	0	0	1	42.1713	19.0982
1286	0	0	0	0	0	0	0	0	0	0	0	0	0	0	0	0	0	0	0	0	0	0	0	0	0	0	0	0	0	0	0	0	0	0	0	0	0	0	0	0	0	0	0	0	0	0	0	0	0	0	0	0	0	0	1	0	0	0	0	0	0	0	1	42.2057	19.0559
1289	0	0	0	0	0	0	0	0	0	0	0	0	0	0	0	0	0	0	0	0	0	0	0	0	0	0	0	0	0	0	0	0	0	0	0	0	0	0	0	0	0	0	0	0	0	0	0	0	0	0	0	0	0	0	1	0	0	0	0	0	0	0	1	42.1998	18.9607
1291	0	0	0	0	0	0	0	0	0	0	0	0	0	0	0	0	0	0	0	0	0	0	0	0	0	0	0	0	0	0	0	0	0	0	0	0	0	0	0	0	0	0	0	0	0	0	0	0	0	0	0	0	0	0	1	0	0	0	0	0	0	0	1	42.42666667	18.83138889
1294	0	0	0	0	0	0	0	0	0	0	0	0	0	0	0	0	0	0	0	0	0	0	0	0	0	0	0	0	0	0	0	0	0	0	0	0	0	0	0	0	0	0	0	0	0	0	0	0	0	0	0	0	0	0	0	1	0	0	0	0	0	0	1	42.4116	18.7623
1295	0	0	0	0	0	0	0	0	0	0	0	0	0	0	0	0	0	0	0	0	0	0	0	0	0	0	0	0	0	0	0	0	0	0	0	0	0	0	0	0	0	0	0	0	0	0	0	0	0	0	0	0	0	0	0	1	0	0	0	0	0	0	1	42.413	18.7615
1296	0	0	0	0	0	0	0	0	0	0	0	0	0	0	0	0	0	0	0	0	0	0	0	0	0	0	0	0	0	0	0	0	0	0	0	0	0	0	0	0	0	0	0	0	0	0	0	0	0	0	0	0	0	0	0	1	0	0	0	0	0	0	1	42.4901	18.7532
1297	1972/001	0	0	0	0	0	1	0	0	0	0	0	0	1	0	0	0	0	0	1	0	0	0	1	0	0	1	0	0	0	1	0	0	0	1	0	0	0	0	0	0	0	0	0	0	0	0	0	0	0	0	0	0	0	0	0	0	0	0	0	0	0	1	43.3342	22.0792
1298	1972/002	0	0	0	0	0	0	0	0	0	1	0	0	1	0	0	0	0	0	0	0	0	0	0	0	0	0	0	0	0	1	0	0	0	1	0	0	0	0	0	0	0	0	0	0	0	0	0	0	0	0	0	0	0	0	0	0	0	0	0	0	0	1	43.3302	22.0636
1300	1972/004	1	0	0	0	0	1	0	0	0	0	0	0	1	0	0	0	0	0	1	0	0	0	0	0	0	0	0	0	0	1	0	0	0	1	0	0	0	0	0	0	0	0	0	0	0	0	0	0	0	0	0	0	0	0	0	0	0	0	0	0	0	1	41.4322	21.8516
1301	1972/005	0	0	0	0	0	0	0	0	0	0	0	0	0	0	0	0	0	0	0	0	0	0	0	0	0	0	0	0	0	0	0	0	0	0	0	0	0	0	0	0	0	0	0	0	0	0	0	0	0	0	0	0	0	0	0	0	0	0	0	0	0	1	41.361699	21.703273
1306	1972/010	1	0	0	0	0	1	0	0	0	0	0	0	0	0	0	0	0	0	0	0	0	0	0	0	0	0	0	0	0	0	0	0	0	0	0	0	0	0	0	0	0	0	0	0	0	0	0	0	0	0	0	0	0	0	0	0	0	0	0	0	0	1	41.8949	21.6864
1316	1972/020	1	0	0	0	0	1	0	0	0	0	0	0	0	0	0	0	0	0	1	0	0	0	1	0	0	0	0	0	0	0	0	0	0	0	0	0	0	0	0	0	0	0	0	0	1	0	0	0	0	0	0	0	0	0	0	0	0	0	0	0	0	1	40.95113	20.8004
1318	1972/022	1	0	0	0	0	0	0	0	0	0	0	0	0	1	0	0	0	0	1	0	0	0	0	0	0	0	0	0	0	0	0	0	0	0	0	0	0	0	0	0	0	0	0	0	1	0	0	0	0	0	0	0	0	0	0	0	0	0	0	0	0	1	40.9688	20.7905
1319	1972/023	0	0	0	0	0	1	0	0	0	0	0	0	0	0	0	0	0	0	0	0	0	0	0	0	0	0	0	0	0	1	0	0	0	0	0	0	0	0	0	0	0	0	0	0	1	0	0	0	0	0	0	1	0	0	0	0	0	0	0	0	0	1	41.1109	20.7915
1320	1972/024	0	0	0	0	0	1	0	0	0	0	0	0	0	1	0	0	0	0	0	0	0	0	0	0	0	0	0	0	0	0	0	0	0	0	0	0	0	0	0	0	0	0	0	0	1	0	0	0	0	0	0	0	0	0	0	0	0	0	0	0	0	1	41.2626	20.6379
1323	1972/027	1	0	0	0	0	1	0	0	0	0	0	0	0	1	0	0	0	0	1	0	0	0	0	0	0	0	0	0	0	1	0	0	0	0	0	0	0	0	0	0	0	0	0	0	0	0	0	0	0	0	0	0	0	0	0	0	1	0	0	0	0	1	41.4432	20.5374
1324	1972/028	1	0	0	0	0	1	0	0	0	0	0	0	0	0	0	0	0	0	0	0	0	0	0	0	0	0	0	0	0	0	0	0	0	0	0	0	0	0	0	0	0	0	0	0	0	0	0	0	0	0	0	0	0	0	0	0	0	0	0	0	0	1	41.554	20.6161
1330	1972/034	0	0	0	0	0	1	0	0	0	0	0	0	0	0	0	0	0	0	0	0	0	0	1	0	0	0	0	0	0	1	0	0	0	0	0	1	0	0	0	0	0	0	0	0	0	0	0	0	0	0	0	0	0	0	0	0	0	0	0	0	0	1	42.7711	20.2804
1331	1972/035	0	0	0	0	0	1	0	0	0	1	0	0	0	0	0	0	0	1	1	0	0	0	1	0	0	1	0	0	1	1	0	0	0	0	0	1	0	0	1	0	0	0	0	0	0	0	0	0	0	0	0	0	0	0	0	0	0	0	0	0	0	1	42.9524	20.4189
1332	1972/036	1	0	0	0	0	1	0	0	0	0	0	0	0	0	0	0	0	0	1	0	0	0	1	0	0	1	0	0	0	1	0	0	0	0	0	0	0	0	0	0	0	0	0	0	0	0	0	0	0	0	0	0	0	0	0	0	0	0	0	0	0	1	42.8544	20.2057
1333	1972/037	1	0	0	0	0	1	0	0	0	0	0	0	0	0	0	0	0	0	0	0	0	0	0	0	0	0	0	1	1	0	0	0	0	0	0	0	0	0	0	0	0	0	0	0	0	0	0	0	0	0	0	0	0	0	0	0	0	0	0	0	0	1	42.7602	19.3896
1334	1972/038	1	0	0	0	0	0	0	0	0	0	0	0	1	0	0	0	0	0	0	0	0	0	0	0	0	0	0	1	0	0	0	0	0	0	0	0	1	0	0	0	0	0	0	0	0	0	0	0	0	0	0	0	0	1	0	0	0	0	0	0	0	1	42.3828	19.1022
1336	1972/040	0	0	0	0	0	0	0	0	0	1	0	0	1	0	0	0	0	0	0	0	1	0	0	0	0	0	0	0	1	1	0	0	0	0	0	0	1	0	0	0	0	0	0	0	0	0	0	0	0	0	0	0	0	1	0	0	0	0	0	0	0	1	42.3367	18.9971
1338	1972/042	0	0	0	0	0	0	0	0	0	0	0	0	0	0	0	0	0	0	0	0	0	0	0	0	0	0	0	0	0	0	0	0	0	0	0	0	1	0	0	0	0	0	0	0	0	0	0	0	0	0	0	0	0	0	0	0	0	0	0	0	0	1	42.2711	19.1241
1339	1972/043	0	0	0	0	0	0	0	0	0	0	0	0	1	0	0	0	0	0	0	0	0	0	0	0	0	0	0	1	1	0	0	0	0	0	0	0	1	0	0	0	0	0	0	0	0	0	0	0	0	0	0	0	0	1	0	0	0	0	0	0	0	1	42.2248	19.125
1340	1972/044	0	0	0	0	0	0	0	0	0	0	0	0	0	0	0	0	0	0	0	0	0	0	0	0	0	0	0	0	0	0	0	0	0	0	0	0	1	0	0	0	0	0	0	0	0	0	0	0	0	0	0	0	0	0	0	0	0	0	0	0	0	1	42.2137	19.0152
1342	1972/046	1	0	0	0	0	1	0	0	0	1	0	0	1	0	1	0	0	0	1	0	1	0	0	0	0	0	0	1	1	0	1	0	0	0	0	0	0	0	0	0	0	1	0	1	0	0	0	0	0	0	0	0	0	0	0	0	0	0	0	0	0	1	42.5136	18.6816
1344	1972/048	1	0	0	0	0	0	0	0	0	0	0	0	0	0	0	0	0	0	0	0	0	0	0	0	0	0	0	0	0	0	1	0	0	0	0	0	0	0	0	0	0	0	0	0	0	0	0	0	0	0	0	0	0	0	0	0	0	0	0	0	0	1	42.4282	18.811
1346	1972/050	0	0	0	0	0	0	0	0	0	0	0	0	1	0	0	0	0	0	0	0	0	0	0	0	0	0	0	0	0	0	0	0	0	0	0	0	0	0	0	0	0	0	0	0	0	0	0	0	0	0	0	0	0	0	0	0	0	0	0	0	0	1	42.4302	18.8128
1347	1972/051	0	0	0	1	0	0	0	0	0	0	0	0	0	0	1	0	0	0	0	1	0	0	0	0	0	0	0	1	0	0	1	0	0	0	0	0	1	0	0	0	0	0	0	0	0	0	0	0	0	0	0	0	0	0	0	0	0	0	0	0	0	1	42.6275	18.1198
1348	1972/052	1	0	0	0	0	0	0	0	0	0	0	0	1	0	0	0	0	0	0	1	0	0	0	0	0	0	0	0	0	0	0	0	0	0	0	0	0	0	0	0	0	0	0	1	0	0	0	0	0	0	0	0	0	0	0	0	0	0	0	0	0	1	42.6568	18.0823
1350	1972/054	0	0	0	1	0	0	0	0	0	0	0	0	1	0	0	0	0	0	0	0	1	0	0	0	0	0	0	1	0	0	0	0	0	0	0	0	0	0	0	0	0	0	0	0	0	0	0	0	0	0	0	0	0	0	0	0	0	0	0	0	0	1	42.5853	18.2169
1352	1972/056	1	0	0	1	0	0	0	0	0	1	0	0	1	0	1	0	0	0	1	0	1	0	0	0	0	0	0	1	0	0	1	0	0	0	0	0	0	0	0	0	0	0	0	0	0	0	0	0	0	0	0	0	0	0	0	0	0	0	0	0	0	1	42.6708	18.1013
1354	1972/058	0	0	0	1	0	0	0	0	0	1	0	0	1	0	1	0	0	0	1	0	0	0	0	0	0	0	0	0	0	0	0	0	0	0	0	0	1	0	0	0	0	0	0	0	0	0	0	0	0	0	0	0	0	0	0	0	0	0	0	0	0	1	42.7358	17.5982
1355	1972/059	1	0	0	1	0	0	0	0	0	1	0	0	1	0	1	0	0	1	1	0	0	0	0	0	0	1	0	1	0	0	1	0	0	0	0	0	1	0	0	0	0	0	0	0	0	0	0	0	0	0	0	0	0	0	0	0	0	0	0	0	0	1	42.7368	17.6005
1356	1972/060	1	0	0	1	0	0	0	0	0	1	0	0	1	0	1	0	0	0	1	0	0	0	0	0	0	0	0	1	0	0	1	0	0	0	0	0	1	0	0	0	0	0	1	0	0	0	0	0	0	0	0	0	0	0	0	0	0	0	0	0	0	1	42.7316	17.5791
1357	1972/061	1	0	0	0	0	0	0	0	0	0	0	0	0	0	0	0	0	0	1	0	0	0	0	0	0	0	0	0	0	0	0	0	0	0	0	0	0	0	0	0	0	0	0	0	0	0	0	0	0	0	0	0	0	0	0	0	0	0	0	0	0	1	43.0281	17.651
1358	1972/062	0	0	0	0	0	0	0	0	0	0	0	0	1	0	0	0	0	0	1	0	0	0	0	0	0	0	0	1	0	0	1	0	0	0	0	0	1	0	0	0	0	0	0	0	0	0	0	0	0	0	0	0	0	0	0	0	0	0	0	0	0	1	43.0178	17.6757
1359	1972/063	1	0	0	0	0	0	0	0	0	0	0	0	1	0	1	0	0	0	1	0	0	0	0	0	0	0	0	1	0	0	1	0	0	0	0	0	1	0	0	0	0	0	0	0	0	0	0	0	0	0	0	0	0	0	0	0	0	0	0	0	0	1	43.0412	17.6605
1360	1972/064	1	0	0	0	0	0	0	0	0	0	0	0	0	0	0	0	0	0	1	0	0	0	0	0	0	0	0	0	0	0	0	0	0	0	0	0	0	0	0	0	0	1	0	0	0	0	0	0	0	0	0	0	0	0	0	0	0	0	0	0	0	1	43.4008	16.8642
1363	1972/067	0	0	0	0	0	0	0	0	0	0	0	0	0	0	0	0	0	0	0	0	0	0	0	0	0	0	0	1	0	1	0	0	0	0	0	0	0	0	0	0	0	0	0	0	0	0	0	0	0	0	0	0	0	0	0	0	0	0	0	0	0	1	44.9811	14.9808
1364	1972/068	0	0	0	0	0	0	0	0	0	0	0	0	0	0	0	0	0	0	0	0	0	0	0	0	0	0	0	0	0	0	0	0	0	0	0	0	0	0	0	0	0	0	0	0	0	0	0	0	0	0	0	0	0	0	0	0	0	0	0	0	0	1	44.7873	15.3672
1365	1972/069	1	0	0	0	0	1	0	0	0	1	0	0	1	0	1	0	0	1	1	0	0	0	1	0	0	1	0	1	1	1	0	0	0	0	0	1	0	0	1	0	0	0	0	1	0	0	0	0	0	0	0	0	0	0	0	0	0	0	0	0	0	1	44.9015	15.6087
1366	1976/005	1	0	0	0	0	0	0	0	0	1	0	0	0	0	0	0	0	0	0	0	0	0	0	0	0	0	0	0	0	0	0	0	0	0	0	0	0	0	0	0	0	0	0	0	0	0	0	0	0	0	0	0	0	0	0	0	0	0	0	0	0	1	39.4771	20.3137
1367	1976/006	0	0	0	0	0	0	0	0	0	1	0	0	0	0	0	0	0	0	0	0	0	0	0	0	0	0	0	0	0	0	0	0	0	0	0	0	0	0	0	0	0	0	0	0	0	0	0	0	0	0	0	0	0	0	0	0	0	0	0	0	0	1	39.5944	20.4918
1368	1976/007	1	0	0	0	0	0	0	0	0	0	0	0	0	0	0	0	0	0	0	0	0	0	0	0	0	0	0	0	0	0	0	0	0	0	0	0	0	0	0	0	0	0	0	0	0	0	0	0	0	0	0	0	0	0	0	0	0	0	0	0	0	1	39.6442	20.5129
1369	1976/008	0	0	0	0	0	0	0	0	0	1	0	0	0	0	0	0	0	0	0	0	0	0	0	0	0	0	0	0	0	0	0	0	0	0	0	0	0	0	0	0	0	0	0	0	0	0	0	0	0	0	0	0	0	0	0	0	0	0	0	0	0	1	39.6393	20.9145
1370	1976/009	0	0	0	0	0	0	0	0	0	0	0	0	0	0	0	0	0	0	0	0	0	0	0	0	0	0	0	0	0	0	0	0	0	0	0	0	0	0	0	0	0	0	0	0	0	0	0	0	0	0	0	0	0	0	0	0	0	0	0	0	0	1	39.7882	21.3874
1371	1976/010	0	0	0	0	0	0	0	0	0	0	0	0	1	0	0	0	0	0	0	0	0	0	0	0	0	0	0	0	0	1	0	0	0	1	0	0	0	0	0	0	0	0	0	1	0	0	0	0	0	0	0	0	0	0	0	0	0	0	0	0	0	1	40.1131	22.485
1372	1976/011	1	0	0	0	0	0	0	0	0	1	0	0	1	1	0	0	0	0	0	0	0	0	0	0	0	0	0	0	0	0	0	0	0	1	0	0	0	0	0	0	0	0	0	1	0	0	0	0	0	0	0	0	0	0	0	0	0	0	0	0	0	1	39.8796	22.587
1374	1976/013	0	0	0	0	0	1	0	0	0	0	0	0	0	1	0	0	0	0	1	0	0	0	0	0	0	0	0	0	0	0	0	0	0	1	0	0	0	0	0	0	0	0	0	0	0	0	0	0	0	0	0	0	0	0	0	0	0	0	0	0	0	1	39.6991	22.2134
1378	1976/017	1	0	0	0	0	0	0	0	0	0	0	0	0	0	0	0	0	0	1	0	0	0	0	0	0	0	0	0	0	0	0	0	0	0	0	0	0	0	0	0	0	0	0	0	0	0	0	0	0	0	0	0	0	0	0	0	0	0	0	0	0	1	39.7326	21.0589
1383	1976/023	0	0	0	0	0	0	0	0	0	0	0	0	0	0	0	0	0	0	0	0	0	0	0	0	0	0	0	0	0	0	0	0	0	0	0	0	0	0	0	0	0	0	0	0	0	0	0	0	0	0	0	0	0	0	0	0	0	0	0	0	0	1	39.4959	20.875
1384	1976/024	1	0	0	0	0	0	0	0	0	1	0	0	1	0	0	0	0	0	1	1	0	0	0	0	0	1	0	1	0	0	0	0	0	0	0	0	0	0	0	0	0	0	0	0	0	0	0	0	0	0	0	0	0	0	0	0	0	0	0	0	0	1	39.4497	20.879
1385	1976/025	1	0	0	0	0	0	0	0	0	1	0	0	0	0	0	0	0	0	1	0	0	0	0	0	0	1	0	1	0	0	0	1	0	0	0	0	0	0	0	0	0	0	0	0	0	0	0	0	0	0	0	0	0	0	0	0	0	0	0	0	0	1	39.3607	20.888
1386	1976/026	1	0	0	0	0	0	0	0	0	1	0	0	1	0	0	0	0	0	1	0	0	0	0	0	0	1	0	1	0	0	0	0	0	0	0	0	0	0	0	0	0	0	0	0	0	0	0	0	0	0	0	0	0	0	0	0	0	0	0	0	0	1	38.6419	20.7089
1387	1976/027	1	0	0	0	0	0	0	0	0	0	0	0	0	1	0	0	0	0	0	0	0	0	0	0	0	0	0	0	0	0	0	0	0	0	0	0	0	0	0	0	0	0	0	1	0	0	0	0	0	0	0	0	0	0	0	0	0	0	0	0	0	1	38.9877	20.783
1388	1976/028	1	0	0	1	0	0	0	0	0	0	0	0	0	0	0	0	0	0	1	0	0	0	0	0	0	1	0	1	0	0	0	1	0	0	0	0	0	0	0	0	0	0	0	0	0	0	0	0	0	0	0	0	0	0	0	0	0	0	0	0	0	1	38.5006	21.3674
1389	1976/029	1	0	0	1	0	0	0	0	0	0	0	0	0	1	0	0	0	0	1	0	0	0	0	0	0	0	0	0	0	0	0	1	0	0	0	0	0	0	0	0	0	0	0	0	0	0	0	0	0	0	0	0	0	0	0	0	0	0	0	0	0	1	38.3929	21.8259
1390	1976/030	0	0	0	0	0	0	0	0	0	0	0	0	0	0	0	0	0	0	0	0	0	0	0	0	0	0	0	0	0	0	0	0	0	0	0	0	0	0	0	0	0	0	0	0	0	0	0	0	0	0	0	0	0	0	0	0	0	0	0	0	0	1	38.1492	22.2296
1391	1976/031	0	0	0	0	0	0	0	0	0	0	0	0	0	0	0	0	0	0	0	0	0	0	0	0	0	0	0	0	0	0	0	0	0	0	0	0	0	0	0	0	0	0	0	0	0	0	0	0	0	0	0	0	0	0	0	0	0	0	0	0	0	1	38.1295	22.2172
1392	1976/032	0	0	0	0	0	0	0	0	0	0	0	0	1	0	0	0	0	0	0	0	0	0	0	0	0	0	0	0	0	0	0	0	0	0	0	0	0	0	0	0	0	0	0	0	0	0	0	0	0	0	0	0	0	0	0	0	0	0	0	0	0	1	38.1206	22.1719
1393	1976/033	0	0	0	0	0	0	0	0	0	0	0	0	1	0	0	0	0	0	0	0	0	0	0	0	0	0	0	0	0	0	0	0	0	0	0	0	0	0	0	0	0	0	0	0	0	0	0	0	0	0	0	0	0	0	0	0	0	0	0	0	0	1	38.0884	22.1746
1394	1976/034	1	0	0	0	0	0	0	0	0	1	0	0	0	1	0	0	0	0	0	0	0	0	0	0	0	0	0	0	0	0	0	1	0	0	0	0	0	0	0	0	0	0	0	0	0	0	0	0	0	0	0	0	0	0	0	0	0	0	0	0	0	1	38.0286	21.9965
1395	1976/035	1	0	0	0	0	0	0	0	0	0	0	0	0	0	0	0	0	0	0	0	0	0	0	0	0	0	0	0	0	0	0	1	0	0	0	0	0	0	0	0	0	0	0	0	0	0	0	0	0	0	0	0	0	0	0	0	0	0	0	0	0	1	38.0674	21.8513
1396	1976/036	1	0	0	0	0	0	0	0	0	0	0	0	0	1	0	0	0	0	0	0	0	0	0	0	0	0	0	0	0	0	0	0	0	0	0	0	0	0	0	0	0	0	0	0	0	0	0	0	0	0	0	0	0	0	0	0	0	0	0	0	0	1	37.6384	21.6307
1397	1976/037	1	0	0	0	0	0	0	0	0	0	0	0	1	1	0	0	0	0	1	0	0	0	0	0	0	0	0	0	0	0	0	0	0	0	0	0	0	0	0	0	0	0	0	0	0	0	0	0	0	0	0	0	0	0	0	0	0	0	0	0	0	1	37.6989	21.9392
1398	1976/038	1	0	0	0	0	0	0	0	0	1	0	0	0	0	0	0	0	0	1	0	0	0	0	0	0	1	0	0	0	0	0	0	0	0	0	0	0	0	0	0	0	0	0	0	0	0	0	0	0	0	0	0	0	0	0	0	0	0	0	0	0	1	37.6815	22.0147
1399	1976/039	0	0	0	0	0	0	0	0	0	0	0	0	1	0	0	0	0	0	1	0	0	0	0	0	0	0	0	0	0	0	0	0	0	0	0	0	0	0	0	0	0	0	0	0	0	0	0	0	0	0	0	0	0	0	0	0	0	0	0	0	0	1	37.6727	22.0534
1400	1976/040	1	0	0	0	0	0	0	0	0	0	0	0	1	0	0	0	0	0	0	0	0	0	0	0	0	0	0	0	0	0	0	0	0	0	0	0	0	0	0	0	0	0	0	0	0	0	0	0	0	0	0	0	0	0	0	0	0	0	0	0	0	1	37.6515	22.1685
1401	1976/041	0	0	0	0	0	0	0	0	0	0	0	0	0	0	0	0	0	0	0	0	0	0	0	0	0	0	0	0	0	0	0	0	0	0	0	0	0	0	0	0	0	0	0	0	0	0	0	0	0	0	0	0	0	0	0	0	0	0	0	0	0	1	37.4101	22.1273
1402	1976/042	1	0	0	0	0	0	0	0	0	0	0	0	0	0	0	0	0	0	1	0	1	0	0	0	0	0	0	0	0	0	0	0	0	0	0	0	0	0	0	0	0	0	0	0	0	0	0	0	0	0	0	0	0	0	0	0	0	0	0	0	0	1	37.3319	22.0743
1403	1976/043	1	0	0	0	0	0	0	0	0	0	0	0	0	1	0	0	0	0	0	0	0	0	0	0	0	0	0	0	0	0	0	0	0	0	0	0	0	0	0	0	0	0	0	0	0	0	0	0	0	0	0	0	0	0	0	0	0	0	0	0	0	1	37.0364	22.1663
1404	1976/044	1	0	0	0	0	0	0	0	0	1	0	0	1	1	0	0	0	0	1	0	0	0	0	0	0	1	0	0	0	0	0	1	0	0	0	0	0	0	0	0	0	0	0	0	0	0	0	0	0	0	0	0	0	0	0	0	0	0	0	0	0	1	37.0412	22.2001
1405	1976/045	1	0	0	0	0	0	0	0	0	1	0	0	1	0	0	0	0	0	1	0	1	0	0	0	0	0	0	0	0	0	0	0	0	0	0	0	0	0	0	0	0	0	0	0	0	0	0	0	0	0	0	0	0	0	0	0	0	0	0	0	0	1	37.1485	21.9418
1406	1976/046	1	0	0	0	0	0	0	0	0	0	0	0	1	1	0	0	0	0	0	0	0	0	0	0	0	0	0	0	0	0	0	1	0	0	0	0	0	0	0	0	0	0	0	0	0	0	0	0	0	0	0	0	0	0	0	0	0	0	0	0	0	1	37.1722	21.9179
1407	1976/047	1	0	0	0	0	0	0	0	0	0	0	0	1	1	0	0	0	0	1	0	0	0	0	0	0	1	0	0	0	0	0	1	0	0	0	0	0	0	0	0	0	0	0	0	0	0	0	0	0	0	0	0	0	0	0	0	0	0	0	0	0	1	36.9845	22.1619
1408	1976/048	1	0	0	0	0	0	0	0	0	0	0	0	0	1	0	0	0	0	0	0	0	0	0	0	0	0	0	0	0	0	0	0	0	0	0	0	0	0	0	0	0	0	0	0	0	0	0	0	0	0	0	0	0	0	0	0	0	0	0	0	0	1	36.9556	22.1913
1409	1976/049	0	0	0	0	0	0	0	0	0	0	0	0	1	0	0	0	0	0	0	0	0	0	0	0	0	0	0	0	0	0	0	0	0	0	0	0	0	0	0	0	0	0	0	0	0	0	0	0	0	0	0	0	0	0	0	0	0	0	0	0	0	1	36.9313	22.1915
1410	1976/050	1	0	0	0	0	0	0	0	0	1	0	0	1	1	0	0	0	0	1	0	0	0	0	0	0	1	0	0	0	0	0	1	0	0	0	0	0	0	0	0	0	0	0	0	0	0	0	0	0	0	0	0	0	0	0	0	0	0	0	0	0	1	37.0298	22.1865
1411	1976/051	0	0	0	0	0	0	0	0	0	0	0	0	1	0	0	0	0	0	0	0	0	0	0	0	0	0	0	0	0	0	0	0	0	0	0	0	0	0	0	0	0	0	0	0	0	0	0	0	0	0	0	0	0	0	0	0	0	0	0	0	0	1	37.0808	22.2753
1412	1976/052	0	0	0	0	0	0	0	0	0	1	0	0	0	1	0	0	0	0	0	0	0	0	0	0	0	0	0	0	0	0	0	1	0	0	0	0	0	0	0	0	0	0	0	0	0	0	0	0	0	0	0	0	0	0	0	0	0	0	0	0	0	1	37.0745	22.2955
1413	1976/053	0	0	0	0	0	0	0	0	0	0	0	0	0	1	0	0	0	0	0	0	0	0	0	0	0	1	0	0	0	0	0	1	0	0	0	0	0	0	0	0	0	0	0	0	0	0	0	0	0	0	0	0	0	0	0	0	0	0	0	0	0	1	37.0739	22.3691
1414	1976/054	0	0	0	0	0	0	0	0	0	0	0	0	0	0	0	0	0	0	0	0	0	0	0	0	0	0	0	0	0	0	0	1	0	0	0	0	0	0	0	0	0	0	0	0	0	0	0	0	0	0	0	0	0	0	0	0	0	0	0	0	0	1	37.2968	22.4175
1415	1976/055	0	0	0	0	0	0	0	0	0	0	0	0	0	0	0	0	0	0	0	0	0	0	0	0	0	0	0	0	0	0	0	0	0	0	0	0	0	0	0	0	0	0	0	0	0	0	0	0	0	0	0	0	0	0	0	0	0	0	0	0	0	1	37.3813	22.4034
1416	1976/056	0	0	0	0	0	0	0	0	0	0	0	0	0	0	0	0	0	0	0	0	0	0	0	0	0	0	0	0	0	0	0	0	0	0	0	0	0	0	0	0	0	0	0	0	0	0	0	0	0	0	0	0	0	0	0	0	0	0	0	0	0	1	37.4537	22.4235
1417	1976/057	0	0	0	0	0	0	0	0	0	0	0	0	1	0	0	0	0	0	0	0	0	0	0	0	0	0	0	0	0	0	0	1	0	0	0	0	0	0	0	0	0	0	0	0	0	0	0	0	0	0	0	0	0	0	0	0	0	0	0	0	0	1	37.5356	22.5136
1419	1976/059	0	0	0	0	0	1	0	0	0	0	0	0	1	1	0	0	0	0	0	0	0	0	0	0	0	0	0	0	0	0	0	1	0	0	0	0	0	0	0	0	0	0	0	0	0	0	0	0	0	0	0	0	0	0	0	0	0	0	0	0	0	1	37.5232	22.6404
1421	1976/061	0	0	0	0	0	1	0	0	0	0	0	0	1	1	1	0	0	0	1	0	0	0	0	0	0	0	0	0	0	0	0	1	0	0	0	0	0	0	0	0	0	0	0	0	0	0	0	0	0	0	0	0	0	0	0	0	0	0	0	0	0	1	37.599	22.8001
1422	1976/062	1	0	0	0	0	1	0	0	0	0	0	0	1	1	1	0	0	0	0	0	0	0	0	0	0	0	0	0	0	0	0	1	0	0	0	0	0	0	0	0	0	0	0	0	0	0	0	0	0	0	0	0	0	0	0	0	0	0	0	0	0	1	37.7291	22.7577
1423	1976/063	0	0	0	0	0	0	0	0	0	0	0	0	0	0	0	0	0	0	0	0	0	0	0	0	0	0	0	0	0	0	0	0	0	0	0	0	0	0	0	0	0	0	0	0	0	0	0	0	0	0	0	0	0	0	0	0	0	0	0	0	0	1	37.5936	22.9445
1424	1976/064	0	0	0	0	0	0	0	0	0	0	0	0	0	1	0	0	0	0	0	0	0	0	0	0	0	0	0	0	0	0	0	0	0	0	0	0	0	0	0	0	0	0	0	0	0	0	0	0	0	0	0	0	0	0	0	0	0	0	0	0	0	1	37.6334	23.1599
1425	1976/065	1	0	0	0	0	0	0	0	0	0	0	0	0	1	0	0	0	0	0	0	0	0	0	0	0	0	0	0	0	0	0	0	0	0	0	0	0	0	0	0	0	0	0	0	0	0	0	0	0	0	0	0	0	0	0	0	0	0	0	0	0	1	37.7334	23.101
1426	1976/066	1	0	0	0	0	0	0	0	0	1	0	0	1	1	0	0	0	0	1	0	0	0	0	0	0	1	0	0	0	0	0	0	0	0	0	0	0	0	0	0	0	0	1	0	0	0	0	0	0	0	0	0	0	0	0	0	0	0	0	0	0	1	37.9059	22.8792
1427	1976/068	0	0	0	0	0	0	0	0	0	0	0	0	0	0	0	0	0	0	0	0	0	0	0	0	0	0	0	0	0	0	0	0	0	0	0	0	0	0	0	0	0	0	0	0	0	0	0	0	0	0	0	0	0	0	0	0	0	0	0	0	0	1	37.9699	23.7334
1428	1976/200	0	0	0	0	0	0	0	0	0	0	0	0	1	1	0	0	0	0	0	1	0	0	0	0	0	0	0	0	0	0	0	0	0	0	0	0	0	0	0	1	0	0	0	0	0	0	0	0	0	0	0	0	0	0	0	0	0	0	0	0	0	1	37.983023	23.747196
1429	1976/201	0	0	0	0	0	0	0	0	0	0	0	0	1	1	0	0	0	0	0	0	0	0	0	0	0	0	0	0	0	1	0	0	0	0	0	0	0	0	0	0	0	0	0	0	0	0	0	0	0	0	0	0	0	0	0	0	0	0	0	0	0	1	38.168458	24.231141
1432	1981/B/003	0	0	0	0	0	1	0	0	1	1	0	0	1	1	1	1	0	0	0	0	0	0	0	0	0	0	0	0	0	1	0	0	0	0	0	0	0	0	0	0	0	0	0	0	0	0	0	0	0	0	0	0	0	0	0	0	0	0	0	0	0	1	41.7689	23.4267
1436	1984/A/002	0	0	0	0	0	0	0	0	0	0	0	0	0	0	1	0	0	0	1	0	0	0	0	0	0	0	0	0	0	0	0	0	0	0	0	0	0	0	0	0	0	0	0	0	0	0	0	0	0	0	0	0	0	0	0	0	0	0	0	0	0	1	43.61949	22.040561
1437	1984/A/003	0	0	0	0	0	1	0	0	1	0	0	0	1	0	1	1	1	0	1	0	1	0	0	0	0	0	0	0	0	1	0	0	0	0	0	1	0	0	0	0	0	0	0	0	0	0	0	0	0	0	0	0	0	0	0	0	0	0	0	0	0	1	43.595442	22.095047
1438	1984/A/004	0	0	0	0	0	1	0	0	0	0	0	0	1	0	1	0	0	0	1	0	0	0	0	0	0	0	0	0	0	0	0	0	0	1	0	0	0	0	0	0	0	0	0	0	0	0	0	0	0	0	0	0	0	0	0	0	0	0	0	0	0	1	43.340293	22.091135
1439	1984/A/005	0	0	0	0	0	0	0	0	1	0	0	0	1	0	0	0	0	0	0	0	0	0	0	0	0	0	0	0	0	1	0	0	0	0	0	0	0	0	0	0	0	0	0	0	0	0	0	0	0	0	0	0	0	0	0	0	0	0	0	0	0	1	41.52419	23.391398
1442	1984/A/008	0	0	0	0	0	1	0	0	1	0	1	0	1	0	1	0	0	0	0	0	1	0	0	0	0	0	0	0	0	1	0	0	0	0	0	0	0	0	0	0	1	0	0	0	0	0	0	0	0	0	0	0	0	0	0	0	0	0	0	0	0	1	42.175572	23.981482
1443	1984/A/009	0	0	0	0	0	0	0	0	1	0	0	0	1	0	0	0	0	0	0	0	0	0	0	0	0	0	0	0	0	0	0	0	0	0	0	0	0	0	0	0	1	0	0	0	0	0	0	0	0	0	0	0	0	0	0	0	0	0	0	0	0	1	42.161665	23.970234
1445	1984/A/011	0	0	0	0	0	0	0	0	1	1	0	0	1	1	1	0	0	0	0	0	0	0	0	0	0	0	0	0	0	1	0	0	0	0	0	0	0	0	0	0	1	0	0	0	0	0	0	0	0	0	0	0	0	0	0	0	0	0	0	0	0	1	41.683493	24.363566
1447	1984/A/013	0	0	0	0	0	1	0	0	0	0	0	0	1	0	1	0	0	0	0	0	0	0	0	0	0	0	0	0	0	1	0	0	0	0	0	0	0	0	0	0	0	0	0	0	0	0	0	0	0	0	0	0	0	0	0	0	0	0	0	0	0	1	41.850135	24.683023
1448	1984/A/014	0	0	0	0	0	1	0	0	0	0	0	0	1	0	1	0	0	0	1	0	0	0	0	0	0	0	0	0	0	1	0	0	0	0	0	0	0	0	0	0	0	0	0	0	0	0	0	0	0	0	0	0	0	0	0	0	0	0	0	0	0	1	41.841983	24.679184
1449	1984/A/015	0	0	0	0	0	1	0	0	0	0	0	0	0	0	1	0	0	0	0	0	0	0	0	0	0	0	0	0	0	0	0	0	0	0	0	0	0	0	0	0	0	0	0	0	0	0	0	0	0	0	0	0	0	0	0	0	0	0	0	0	0	1	41.879742	24.69584
1450	1984/A/016	1	0	0	0	0	1	0	0	1	0	0	0	1	1	1	0	0	0	1	0	1	0	0	0	0	0	0	0	0	1	0	0	0	0	0	0	0	0	0	0	0	0	0	0	0	0	0	0	0	0	0	0	0	0	0	0	0	0	0	0	0	1	41.941993	24.849907
1451	1984/A/017	1	1	1	0	1	1	0	0	1	1	1	0	1	0	0	0	0	0	0	0	1	0	0	0	1	1	0	0	0	1	0	0	0	0	0	0	0	0	0	0	0	0	0	0	0	0	0	0	0	0	0	0	0	0	0	0	0	0	0	0	0	1	41.934101	24.849523
1453	1984/A/019	1	0	0	0	0	1	0	0	0	0	0	0	1	0	1	0	0	0	0	0	0	0	0	0	0	0	0	0	0	1	0	0	0	0	0	0	0	0	0	0	0	0	0	0	0	0	0	0	0	0	0	0	0	0	0	0	0	0	0	0	0	1	42.685732	26.356405
1454	1984/A/020	1	0	0	0	0	1	0	0	0	0	0	0	0	1	1	0	0	0	0	0	0	0	0	0	0	0	0	0	0	1	0	0	0	0	0	0	0	0	0	0	0	0	0	0	0	0	0	0	0	0	0	0	0	0	0	0	0	0	0	0	0	1	42.701669	26.350307
1465	1984/A/031	1	0	0	0	1	0	0	0	1	0	1	0	1	1	1	0	1	1	1	0	0	0	1	0	1	1	0	0	0	1	0	0	0	0	0	0	0	0	0	0	0	0	0	0	0	0	0	0	0	0	0	0	0	0	0	0	0	0	0	0	0	1	42.957798	25.432085
1466	1984/A/032	0	0	0	0	0	0	0	0	0	0	0	0	0	0	0	0	0	0	0	0	0	0	0	0	0	0	0	0	0	1	0	0	0	0	0	0	0	0	0	0	1	0	0	0	0	0	0	0	0	0	0	0	0	0	0	0	0	0	0	0	0	1	42.748159	25.321604
1467	1984/A/033	1	0	0	0	0	1	1	0	1	1	0	0	1	0	1	1	1	0	1	0	1	0	1	0	0	1	0	0	0	1	0	0	0	0	0	0	0	0	0	0	1	0	0	0	0	0	0	0	0	0	0	0	0	0	0	0	0	0	0	0	0	1	41.818016	24.589782
1469	1984/A/035	0	0	0	0	0	1	0	0	0	0	0	0	0	0	1	0	0	0	1	0	0	0	0	0	0	0	0	0	0	1	0	0	0	0	0	0	0	0	0	0	0	0	0	0	0	0	0	0	0	0	0	0	0	0	0	0	0	0	0	0	0	1	41.868899	24.691987
1470	1984/A/036	0	0	0	0	0	1	0	0	1	1	0	0	1	1	1	0	0	0	0	0	0	0	0	0	0	0	0	0	0	1	0	0	0	0	0	0	0	0	0	0	0	0	0	0	0	0	0	0	0	0	0	0	0	0	0	0	0	0	0	0	0	1	41.987505	24.875516
1471	1984/A/037	0	0	0	0	0	1	0	0	0	0	0	0	0	1	0	0	0	0	0	0	0	0	0	0	0	0	0	0	0	1	0	0	0	0	0	0	0	0	0	0	0	0	0	0	0	0	0	0	0	0	0	0	0	0	0	0	0	0	0	0	0	1	43.071718	23.398599
1475	1984/A/041	0	0	0	0	0	0	0	0	0	0	0	0	1	0	0	0	0	0	0	0	0	0	0	0	0	0	0	0	0	0	0	0	0	0	0	0	0	0	0	0	0	0	0	0	0	0	0	0	0	0	0	0	0	0	0	0	0	0	0	0	0	1	43.621563	22.684626
1476	1984/A/042	1	0	0	0	0	1	0	0	1	0	0	0	1	1	1	0	0	0	1	0	1	0	1	0	0	0	0	0	0	1	0	0	0	0	0	1	0	0	0	0	1	0	0	0	0	0	0	0	0	0	0	0	0	0	0	0	0	0	0	0	0	1	43.651163	22.693433
1477	1984/A/043	1	0	0	0	0	1	0	0	1	0	0	0	1	1	1	1	0	0	1	0	1	0	1	0	0	0	0	0	0	1	0	0	0	0	0	1	0	0	0	0	1	0	0	0	0	0	0	0	0	0	0	0	0	0	0	0	0	0	0	0	0	1	43.656246	22.716653
1478	1984/A/044	0	0	0	0	0	0	0	0	0	0	0	0	0	0	1	0	0	0	0	0	0	0	0	0	0	0	0	0	0	1	0	0	0	0	0	0	0	0	0	0	0	0	0	0	0	0	0	0	0	0	0	0	0	0	0	0	0	0	0	0	0	1	43.992936	22.886648
1479	1984/A/045	0	0	0	0	0	1	0	0	0	0	0	0	0	0	1	0	0	0	1	0	0	0	0	0	0	0	0	0	0	0	0	0	0	0	0	0	0	0	0	0	0	0	0	0	0	0	0	0	0	0	0	0	0	0	0	0	0	0	0	0	0	1	43.727011	22.583722
1480	1984/A/046	0	0	0	0	0	1	0	0	0	0	0	0	1	0	1	0	0	0	0	0	0	0	0	0	0	0	0	0	0	0	0	0	0	0	0	0	0	0	0	0	0	0	0	0	0	0	0	0	0	0	0	0	0	0	0	0	0	0	0	0	0	1	43.73204	22.577151
1481	1984/A/047	1	0	0	0	1	1	0	0	1	0	0	0	1	0	1	1	0	0	1	0	1	0	0	0	0	0	0	0	0	1	0	0	0	0	0	1	0	0	1	0	0	0	0	0	0	0	0	0	0	0	0	0	0	0	0	0	0	0	0	0	0	1	44.029196	21.96296
1482	1984/A/048	0	0	0	0	0	1	0	0	1	0	0	0	1	0	1	0	0	0	1	0	1	0	0	0	0	0	0	0	0	1	0	0	0	0	0	0	0	0	0	0	0	0	0	0	0	0	0	0	0	0	0	0	0	0	0	0	0	0	0	0	0	1	43.856	21.627
1483	1985/C/001	0	0	0	0	0	0	0	0	0	0	0	0	0	0	1	0	0	0	0	0	0	0	0	0	0	0	0	0	0	1	0	0	0	0	0	0	0	0	0	0	0	0	0	0	0	0	0	0	0	0	0	0	0	0	0	0	0	0	0	0	0	1	44.1834	20.550021
1487	1985/C/005	0	0	0	0	0	1	0	0	0	0	0	0	1	1	1	0	0	0	0	0	0	0	0	0	0	1	0	0	0	1	0	0	0	0	0	0	0	0	0	0	0	0	0	0	0	0	0	0	0	0	0	0	0	0	0	0	0	0	0	0	0	1	41.891847	21.679516
1490	1985/C/008	1	0	0	0	0	1	0	0	1	0	0	0	1	1	0	1	0	0	0	0	0	0	0	0	0	0	0	0	0	0	0	0	0	0	0	0	0	0	0	0	0	0	0	0	0	0	0	0	0	0	0	0	0	0	0	0	0	0	0	0	0	1	40.944085	20.789567
1494	1985/C/012	1	0	0	0	0	0	0	0	0	0	0	0	0	1	1	0	0	0	0	0	0	0	0	0	0	0	0	0	0	0	0	0	0	0	0	0	0	0	0	0	0	0	0	0	0	0	0	0	0	0	0	0	0	0	0	0	0	1	0	0	0	1	40.993184	20.800593
1495	1985/C/013	0	0	0	0	0	1	0	0	0	0	0	0	1	0	1	0	0	0	0	0	0	0	0	0	0	0	0	0	0	0	0	0	0	0	0	0	0	0	0	0	0	0	0	0	0	0	0	0	0	0	0	0	0	0	0	0	1	0	0	0	0	1	41.358007	20.606138
1498	1985/C/016	0	0	0	0	0	1	0	0	1	1	0	0	1	0	1	1	0	0	1	0	1	0	0	0	1	1	0	0	0	1	0	0	0	0	0	1	0	0	0	0	0	0	0	0	0	0	0	0	0	0	0	0	0	0	0	0	0	0	0	0	0	1	41.71	20.694
1499	1985/C/017	0	0	0	0	0	0	0	0	0	0	0	0	1	0	1	0	0	0	0	0	0	0	0	0	0	0	0	0	0	1	0	0	0	0	0	1	0	0	0	0	0	0	0	0	0	0	0	0	0	0	0	0	0	0	0	0	0	0	0	0	0	1	42.692118	20.084411
1501	1985/C/019	0	0	0	0	0	0	0	0	0	0	0	0	1	0	1	0	0	0	0	0	0	0	0	0	0	0	0	0	0	1	0	0	0	0	0	0	0	0	1	0	0	0	0	0	0	0	0	0	0	0	0	0	0	0	0	0	0	0	0	0	0	1	42.842122	19.870445
1502	1985/C/020	0	0	0	0	0	1	0	0	0	0	0	0	0	0	0	0	0	0	1	0	0	0	0	0	0	0	0	0	0	0	0	0	0	0	0	0	0	0	0	0	0	0	0	0	0	0	0	0	0	0	0	0	0	0	0	0	0	0	0	0	0	1	42.943649	19.57763
1503	1985/C/021	1	0	0	0	0	0	0	0	0	0	0	0	1	0	1	0	0	0	0	0	0	0	0	0	0	0	0	0	0	0	0	0	0	0	0	0	0	0	0	0	0	0	0	0	0	0	0	0	0	0	0	0	0	0	0	0	0	0	0	0	0	1	42.23209	19.025966
1504	1985/C/022	0	0	0	0	0	1	0	0	0	0	0	0	0	0	1	0	0	0	0	0	0	0	0	0	0	0	0	0	0	0	0	0	0	0	0	0	1	0	0	0	0	0	0	0	0	0	0	0	0	0	0	0	0	0	0	0	0	0	0	0	0	1	42.214829	18.95099
1505	1985/C/023	1	0	0	1	0	1	0	0	0	1	0	0	1	0	1	0	0	0	1	0	0	0	0	0	0	0	0	1	1	0	0	0	0	0	0	0	0	0	0	0	0	0	0	1	0	0	0	0	0	0	0	0	0	1	0	0	0	0	0	0	0	1	42.04756	19.136
1508	1985/C/026	1	0	0	0	0	0	0	0	0	0	0	0	0	0	0	0	0	0	1	0	0	0	0	0	0	0	0	0	0	0	0	0	0	0	0	0	1	0	0	0	0	0	0	1	0	0	0	0	0	0	0	0	0	0	0	0	0	0	0	0	0	1	42.053714	19.348619
1510	1985/C/028	1	0	0	0	0	0	0	0	0	0	0	0	0	0	1	0	0	0	0	0	0	0	0	0	0	0	0	1	0	0	0	0	0	0	0	0	1	0	0	0	0	0	0	0	0	0	0	0	0	0	0	0	0	1	0	0	0	0	0	0	0	1	42.431962	19.254932
1511	1985/C/029	1	0	0	0	0	0	0	0	0	0	0	0	1	0	1	0	0	0	0	0	0	0	0	0	0	0	0	1	1	0	0	0	0	0	0	0	0	0	0	0	0	0	0	0	0	0	0	0	0	0	0	0	0	1	0	0	0	0	0	0	0	1	42.390161	19.11594
1512	1985/C/030	1	0	0	0	0	0	0	0	0	0	0	0	0	0	0	0	0	0	0	0	0	0	0	0	0	0	0	0	0	0	0	0	0	0	0	0	0	0	0	0	0	0	0	0	0	0	0	0	0	0	0	0	0	1	0	0	0	0	0	0	0	1	42.379008	18.997754
1513	1985/C/031	1	0	0	0	0	1	0	0	0	0	0	0	1	0	1	0	0	0	0	0	0	0	0	0	0	0	0	1	1	0	0	0	0	0	0	0	0	0	0	0	0	0	0	0	0	0	0	0	0	0	0	0	0	0	0	0	0	0	0	0	0	1	42.433791	18.810255
1514	1985/C/032	1	0	0	0	0	1	0	0	0	0	0	0	1	0	1	0	0	0	0	0	0	0	0	0	0	0	0	1	0	0	1	0	0	0	0	0	1	0	0	0	0	0	0	0	0	0	0	0	0	0	0	0	0	0	1	0	0	0	0	0	0	1	42.423762	18.800793
1517	1985/C/036	1	0	0	0	0	0	0	0	1	0	0	0	0	0	1	0	0	0	0	0	0	0	0	0	0	0	0	0	1	0	0	0	0	0	0	0	1	0	0	0	0	0	0	0	0	0	0	0	0	0	0	0	0	0	0	0	0	0	0	0	0	1	42.845041	17.705071
1518	1985/C/037	0	0	0	0	0	0	0	0	0	0	0	0	1	0	0	0	0	0	1	0	0	0	0	0	0	0	0	0	0	0	1	0	0	0	0	0	1	0	0	0	0	0	0	0	0	0	0	0	0	0	0	0	0	0	0	0	0	0	0	0	0	1	42.876242	17.528588
1519	1985/C/038	1	0	0	0	0	0	0	0	0	0	0	0	1	0	1	0	0	0	0	0	0	0	0	0	0	0	0	1	1	0	0	0	0	0	0	0	1	0	0	0	0	0	0	0	0	0	0	0	0	0	0	0	0	0	0	0	0	0	0	0	0	1	42.919166	17.423351
1521	1985/C/040	1	0	0	0	0	0	0	0	0	0	0	0	1	0	1	0	0	0	1	0	0	0	0	0	0	0	0	1	1	0	0	0	0	0	0	0	0	0	0	0	0	0	0	0	0	0	0	0	0	0	0	0	0	0	0	0	0	0	0	0	0	1	42.9655	17.309
1522	1985/C/041	1	0	0	0	0	0	0	0	0	0	0	0	1	0	1	0	0	0	1	0	0	0	0	0	0	0	0	1	1	0	0	0	0	0	0	0	1	0	0	0	0	0	0	0	0	0	0	0	0	0	0	0	0	0	0	0	0	0	0	0	0	1	43.003484	17.083073
1524	1985/C/043	1	0	0	1	0	0	0	0	0	0	0	0	1	0	1	0	0	0	0	0	0	0	0	0	0	0	0	1	1	0	1	0	0	0	0	0	1	0	0	0	0	0	0	0	0	0	0	0	0	0	0	0	0	0	0	0	0	0	0	0	0	1	42.719076	17.657937

1525	1985/C/044	1	0	0	1	0	0	0	0	0	0	0	0	1	0	1	0	0	0	0	0	0	0	0	0	0	0	0	0	0	0	1	1	0	0	0	0	0	0	0	1	0	0	0	0	0	0	0	0	0	0	0	0	0	0	0	0	0	0	0	0	0	0	0	0	0	0	0	1	42.736467	17.599828
1527	1985/C/046	0	0	0	0	0	0	0	0	0	0	0	0	1	0	0	0	0	0	0	0	0	0	0	0	0	0	0	0	0	0	0	1	0	1	0	0	0	0	0	1	0	0	0	0	0	0	0	0	0	0	0	0	0	0	0	0	0	0	0	0	0	0	0	0	0	0	0	1	42.903278	17.640621
1528	1985/C/047	1	0	0	1	0	0	0	0	0	0	0	0	1	0	1	0	0	0	1	0	0	1	0	0	0	0	0	0	0	0	1	1	0	1	0	0	0	0	0	1	0	0	0	0	0	0	0	0	0	0	0	0	0	0	0	0	0	0	0	0	0	0	0	0	0	0	0	1	42.969128	17.529852
1529	1985/C/048	0	0	0	0	0	0	0	0	0	0	0	0	0	0	1	0	0	0	0	0	0	0	0	0	0	0	0	0	0	0	0	0	0	1	0	0	0	0	0	1	0	0	0	0	0	0	0	0	0	0	0	0	0	0	0	0	0	0	0	0	0	0	0	0	0	0	0	1	43.046437	17.475945
1530	1985/C/049	0	0	0	0	0	0	0	0	0	0	0	0	0	0	1	0	0	0	0	0	0	0	0	0	0	0	0	0	0	0	0	0	0	0	0	0	0	0	0	1	0	0	0	0	0	0	0	0	0	0	0	0	0	0	0	0	0	0	0	0	0	0	0	0	0	0	0	1	43.156082	17.246553
1531	1985/C/050	0	0	0	0	0	0	0	0	0	0	0	0	1	0	0	0	0	0	1	0	0	1	0	0	0	0	0	0	0	0	0	1	0	0	0	0	0	0	0	0	0	0	0	0	1	0	0	1	0	0	0	0	0	0	0	0	0	0	0	0	0	0	0	0	0	0	0	1	43.368441	16.935677
1534	1985/C/054	1	0	0	0	0	0	0	0	0	0	0	0	0	0	0	0	0	0	1	0	0	1	0	0	0	0	0	0	0	0	0	0	0	0	0	0	0	0	0	1	0	0	0	0	1	0	0	1	0	0	0	0	0	0	0	0	0	0	0	0	0	0	0	0	0	0	0	1	43.41601	16.907626
1535	1985/C/055	1	0	0	1	0	0	0	0	0	0	0	0	0	0	0	0	0	0	1	0	0	1	0	0	0	0	0	0	0	0	1	0	0	1	0	0	0	0	0	1	0	0	0	0	0	0	0	0	0	0	0	0	0	0	0	0	0	0	0	0	0	0	0	0	0	0	0	1	43.296438	17.861796
1536	1985/C/056	1	0	0	0	0	0	0	0	0	0	0	0	1	0	0	0	0	0	1	0	0	1	0	0	0	0	0	0	0	0	1	0	0	0	0	0	0	0	0	0	0	0	0	0	0	0	0	0	0	0	0	0	0	0	0	0	0	0	0	0	0	0	0	0	0	0	0	1	43.281596	18.207305
1537	1985/C/057	0	0	0	0	0	1	0	0	0	0	0	0	0	0	1	0	0	0	1	0	0	1	0	0	0	0	0	0	0	0	0	0	0	0	0	0	0	0	0	1	0	0	0	0	0	0	0	0	0	0	0	0	0	0	0	0	0	0	0	0	0	0	0	0	0	0	0	1	43.2824	18.262585
1539	1985/C/059	0	0	0	0	0	1	0	0	0	0	0	0	1	0	1	0	0	0	0	0	0	0	0	1	0	0	0	0	0	0	0	0	0	0	0	0	0	0	0	1	0	0	0	0	0	0	0	0	0	0	0	0	0	0	0	0	0	0	0	0	0	0	0	0	0	0	0	1	43.244063	18.606136
1542	1985/C/062	0	0	0	0	0	0	0	0	0	0	0	0	1	0	1	0	0	0	1	0	0	1	0	0	0	0	0	0	0	0	0	1	0	0	0	0	0	0	0	0	0	1	0	0	0	0	0	0	0	0	0	0	0	0	0	0	0	0	0	0	0	0	0	0	0	0	0	1	43.293596	18.851268
1543	1985/C/063	0	0	0	0	0	0	0	0	0	0	0	0	1	0	1	0	0	0	1	0	0	1	0	0	0	0	0	0	0	0	0	0	0	0	0	0	0	0	0	0	0	1	0	0	0	0	0	0	0	0	0	0	0	0	0	0	0	0	0	0	0	0	0	0	0	0	0	1	43.210107	18.858728
1544	1985/C/064	1	0	0	0	0	1	0	0	0	0	0	0	1	0	1	0	0	0	0	0	0	0	0	0	0	0	0	0	0	0	1	1	0	0	0	0	0	0	0	1	0	0	0	0	0	0	0	0	0	0	0	0	0	0	0	0	0	0	0	0	0	0	0	0	0	0	0	1	42.795624	18.96801
1545	1985/C/065	1	0	0	0	0	0	0	0	0	0	0	0	1	0	0	0	0	0	1	0	0	1	0	0	0	0	0	0	0	0	0	0	0	0	0	0	0	0	0	1	0	1	0	0	0	0	0	0	0	0	0	0	0	0	0	0	0	0	0	0	0	0	0	0	0	0	0	1	42.862667	19.070286
1548	1985/C/067	0	0	0	0	0	0	0	0	0	0	0	0	0	0	1	0	0	0	1	0	0	1	0	0	0	0	0	0	0	0	0	1	0	0	0	0	0	0	0	0	0	1	0	0	0	0	0	0	0	0	0	0	0	0	0	0	0	0	0	0	0	0	0	0	0	0	0	1	43.03511	19.392942
1549	1985/C/068	0	0	0	0	0	0	0	0	0	0	0	0	0	0	0	0	0	0	1	0	0	1	0	0	0	0	0	0	0	0	0	0	0	0	0	0	0	0	0	0	0	0	0	0	0	0	0	0	0	0	0	0	0	0	0	0	0	0	0	0	0	0	0	0	0	0	0	1	42.84201	20.111764
1550	1985/C/069	0	0	0	0	0	1	0	0	0	0	0	0	0	0	0	0	0	0	1	0	0	1	0	0	0	0	0	0	0	0	0	0	1	0	0	0	0	0	0	0	0	1	0	0	0	0	0	0	0	0	0	0	0	0	0	0	0	0	0	0	0	0	0	0	0	0	0	1	43.131007	20.421484
1551	1985/C/070	1	0	0	0	0	0	0	0	0	0	0	0	1	0	1	0	0	0	0	0	0	0	0	0	0	0	0	0	0	0	0	0	0	0	0	0	0	0	0	0	0	1	0	0	0	0	0	0	0	0	0	0	0	0	0	0	0	0	0	0	0	0	0	0	0	0	0	1	43.755724	19.232471
1553	1985/C/072	0	0	0	0	0	0	0	0	0	0	0	0	1	0	1	0	0	0	1	0	0	1	0	0	0	0	0	0	0	0	0	0	0	0	0	0	0	0	0	0	0	0	0	0	0	0	0	0	0	0	0	0	0	0	0	0	0	0	0	0	0	0	0	0	0	0	0	1	43.851662	18.515263
1554	1985/C/073	1	0	0	0	0	0	0	0	0	1	0	0	1	0	1	0	0	0	0	0	0	0	0	0	0	0	0	0	0	0	0	1	1	0	0	0	0	0	1	0	0	0	0	0	0	0	0	0	0	0	0	0	0	0	0	0	0	0	0	0	0	0	0	0	0	0	0	1	43.736487	18.565115
1557	1985/C/076	1	0	0	0	0	0	0	0	0	0	1	0	1	0	1	0	0	0	0	0	0	0	0	0	0	0	0	0	0	0	0	1	1	0	0	0	0	0	0	0	0	0	0	0	0	0	0	0	0	0	0	0	0	0	0	0	0	0	0	0	0	0	0	0	0	0	0	1	44.337867	17.270223
1561	1986/E/004	1	0	1	0	0	1	0	0	0	1	0	0	1	1	1	0	0	0	0	0	0	0	0	0	0	0	0	0	0	0	0	0	1	0	0	0	1	0	0	0	0	0	0	0	0	0	0	0	0	0	0	0	0	0	0	0	0	0	0	0	0	0	0	0	0	0	0	1	41.171945	24.323948
1562	1986/E/005	0	0	0	0	0	1	0	0	0	0	0	0	1	1	1	0	0	0	0	0	0	0	0	0	0	0	0	0	0	0	0	0	0	0	0	0	0	0	0	0	0	0	0	0	0	0	0	0	0	0	0	0	0	0	0	0	0	0	0	0	0	0	0	0	0	0	0	1	41.227009	24.607051
1563	1986/E/006	1	0	0	0	0	1	0	0	0	0	0	0	1	1	1	0	0	0	0	0	0	0	0	0	0	0	0	0	0	0	0	0	1	0	0	0	1	0	0	0	0	0	0	0	0	0	0	0	0	0	0	0	0	0	0	0	0	0	0	0	0	0	0	0	0	0	0	1	41.203751	24.723207
1565	1986/E/008	1	0	0	0	0	0	0	0	0	0	0	0	0	1	1	0	0	0	0	0	0	0	0	0	0	0	0	0	0	0	0	0	0	0	0	0	0	0	0	0	0	0	0	0	0	0	0	0	0	0	0	0	0	0	0	0	0	0	0	0	0	0	0	0	0	0	0	1	40.780752	24.681705
1566	1986/E/009	1	0	0	0	0	1	0	0	0	0	0	0	0	1	1	0	0	0	0	0	0	0	0	0	0	0	0	0	0	0	0	0	1	0	0	0	1	0	0	0	0	0	0	0	0	0	0	0	0	0	0	0	0	0	0	0	0	0	0	0	0	0	0	0	0	0	0	1	40.644632	24.51576
1567	1986/E/010	0	0	1	0	0	1	0	0	1	1	0	0	1	1	1	0	0	0	1	0	0	1	1	0	1	0	0	0	1	0	0	0	1	0	0	0	1	0	0	0	0	0	0	0	0	0	0	0	0	0	0	0	0	0	0	0	0	0	0	0	0	0	0	0	0	0	0	1	40.660941	24.691194
1568	1986/E/011	1	0	0	0	0	0	0	0	0	0	0	0	1	1	1	0	0	0	0	0	0	0	0	0	0	0	0	0	0	0	0	0	0	0	0	0	0	0	0	0	0	0	0	0	0	0	0	0	0	0	0	0	0	0	0	0	0	0	0	0	0	0	0	0	0	0	0	1	40.603636	24.732485
1569	1986/E/012	1	0	0	0	0	1	0	0	1	0	0	0	1	1	1	0	0	0	0	0	0	0	0	0	0	0	0	0	0	0	0	0	1	0	0	0	1	0	0	0	0	0	0	0	0	0	0	0	0	0	0	0	0	0	0	0	0	0	0	0	0	0	0	0	0	0	0	1	41.082345	24.760755
1570	1986/E/013	1	0	0	0	0	1	1	0	1	1	0	0	1	1	1	0	1	0	1	1	0	1	1	0	1	1	0	0	1	0	0	0	1	0	0	0	1	0	0	0	0	0	0	0	0	0	0	0	0	0	0	0	0	0	0	0	0	0	0	0	0	0	0	0	0	0	0	1	41.02333333	24.64178333
1571	1986/E/014	1	0	0	0	0	1	0	0	0	0	0	0	1	1	1	0	0	0	0	0	0	0	0	0	0	0	0	0	0	0	0	0	1	0	0	0	1	0	0	0	0	0	0	0	0	0	0	0	0	0	0	0	0	0	0	0	0	0	0	0	0	0	0	0	0	0	0	1	41.012068	24.28474
1574	1986/E/018	1	0	0	0	0	0	0	0	0	1	0	0	1	1	1	0	0	0	1	0	0	1	1	0	0	1	0	0	1	0	0	0	1	0	0	0	1	0	0	0	0	0	0	0	0	0	0	0	0	0	0	0	0	0	0	0	0	0	0	0	0	0	0	0	0	0	0	1	39.3076	22.404957
1575	1986/E/019	0	0	0	0	0	0	0	0	0	0	0	0	0	1	1	0	0	0	0	0	0	0	0	0	0	0	0	0	0	0	0	0	0	0	0	0	0	0	0	0	0	0	0	0	0	0	0	0	0	0	0	0	0	0	0	0	0	0	0	0	0	0	0	0	0	0	0	1	38.796728	22.536564
1576	1986/E/020	0	0	0	0	0	1	0	0	0	0	0	0	1	0	1	0	0	0	0	0	0	0	0	0	0	0	0	0	0	0	0	0	0	0	0	0	0	0	0	0	0	0	1	0	0	1	0	0	0	0	0	0	0	0	0	0	0	0	0	0	0	0	0	0	0	0	0	1	38.493601	23.160416
1577	1986/E/021	0	0	0	0	0	1	0	0	0	0	0	0	1	1	1	0	0	0	0	0	0	0	0	0	0	0	0	0	0	0	0	0	1	0	0	0	0	0	0	0	0	0	1	0	0	1	0	0	0	0	0	0	0	0	0	0	0	0	0	0	0	0	0	0	0	0	0	1	38.475762	23.164042
1579	1986/E/075	0	0	0	0	0	0	0	0	0	0	0	0	1	1	1	0	0	0	0	0	0	0	0	0	0	0	0	0	0	0	0	0	0	0	0	0	0	0	0	0	0	0	0	0	0	0	0	0	0	0	0	0	0	0	0	0	0	0	0	0	0	0	0	0	0	0	0	1	37.989281	23.337772
1580	1986/E/076	0	0	0	0	0	1	0	0	0	0	0	0	0	0	1	0	0	0	0	0	0	0	0	0	0	0	0	0	0	0	0	0	0	0	0	0	0	0	0	0	0	0	1	0	0	1	0	0	0	0	0	0	0	0	0	0	0	0	0	0	0	0	0	0	0	0	0	1	37.977354	23.260457
1581	1986/E/077	0	0	0	0	0	1	0	0	0	0	0	0	1	1	1	0	0	0	0	0	0	0	0	0	0	0	0	0	0	0	0	0	0	0	1	0	0	0	0	0	0	0	1	0	0	1	0	0	0	0	0	0	0	0	0	0	0	0	0	0	0	0	0	0	0	0	0	1	37.974496	22.977521
1582	1986/E/078	1	0	0	0	0	0	0	0	0	0	0	0	0	0	0	0	0	0	0	0	0	0	0	0	0	0	0	0	0	0	0	0	0	0	1	0	0	0	0	0	0	0	0	0	0	0	0	0	0	0	0	0	0	0	0	0	0	0	0	0	0	0	0	0	0	0	0	1	37.832427	23.025536
1583	1986/E/079	1	0	0	0	0	0	0	0	0	0	0	0	1	1	1	0	0	0	0	0	0	0	0	0	0	0	0	0	0	0	0	0	0	0	1	0	0	0	0	0	0	0	0	0	0	0	0	0	0	0	0	0	0	0	0	0	0	0	0	0	0	0	0	0	0	0	0	1	37.742248	23.114409
1584	1986/E/080	0	0	1	0	0	1	0	0	0	1	0	0	1	1	1	0	0	0	1	0	0	1	0	0	0	1	0	0	1	0	0	0	0	0	1	0	0	0	0	0	0	0	0	0	0	0	0	0	0	0	0	0	0	0	0	0	0	0	0	0	0	0	0	0	0	0	0	1	37.56682	22.801439
1587	1986/E/083	0	0	0	0	0	1	0	0	0	0	0	0	1	1	0	0	0	0	0	0	0	0	0	0	0	0	0	0	0	0	0	0	0	0	1	0	0	0	0	0	0	0	0	0	0	0	0	0	0	0	0	0	0	0	0	0	0	0	0	0	0	0	0	0	0	0	0	1	37.533663	22.563775
1588	1986/E/084	0	0	0	0	0	0	0	0	0	0	0	0	1	1	1	0	0	0	0	0	0	0	0	0	0	0	0	0	0	0	0	0	0	0	1	0	0	0	0	0	0	0	0	0	0	0	0	0	0	0	0	0	0	0	0	0	0	0	0	0	0	0	0	0	0	0	0	1	36.844295	22.605407
1589	1986/E/085	0	0	0	0	0	1	0	0	0	0	0	0	1	1	1	0	0	0	0	0	0	0	0	0	0	0	0	0	0	0	0	0	0	0	1	0	0	0	0	0	0	0	0	0	0	0	0	0	0	0	0	0	0	0	0	0	0	0	0	0	0	0	0	0	0	0	0	1	36.687741	23.052125
1590	1986/E/086	0	0	0	0	0	1	0	0	0	0	0	0	0	1	1	0	0	0	0	0	0	0	0	0	0	0	0	0	0	0	1	1	0	0	1	0	0	0	0	0	0	0	0	0	0	0	0	0	0	0	0	0	0	0	0	0	0	0	0	0	0	0	0	0	0	0	0	1	36.674475	23.036393
1591	1986/E/087	0	0	0	0	0	0	0	0	0	0	0	0	0	1	1	0	0	0	0	0	0	0	0	0	0	0	0	0	0	0	0	0	0	0	1	0	0	0	0	0	0	0	0	0	0	0	0	0	0	0	0	0	0	0	0	0	0	0	0	0	0	0	0	0	0	0	0	1	36.692747	23.031056
1592	1986/E/088	0	0	0	0	0	1	0	0	0	0	0	0	0	1	1	0	0	0	0	0	0	0	0	0	0	0	0	0	0	0	0	0	0	0	1	0	0	0	0	0	0	0	0	0	0	0	0	0	0	0	0	0	0	0	0	0	0	0	0	0	0	0	0	0	0	0	0	1	36.682394	22.41508
1593	1986/E/089	0	0	0	0	0	0	0	0	1	0	0	0	0	1	1	0	0	0	0	0	0	0	0	0	0	0	0	0	0	0	1	1	0	0	1	0	0	0	0	0	0	0	0	0	0	0	0	0	0	0	0	0	0	0	0	0	0	0	0	0	0	0	0	0	0	0	0	1	36.625884	22.381854
1594	1986/E/090	0	0	0	0	0	0	0	0	0	0	0	0	0	1	1	0	0	0	0	0	0	0	0	0	0	0	0	0	0	0	1	1	0	0	1	0	0	0	0	0	0	0	0	0	0	0	0	0	0	0	0	0	0	0	0	0	0	0	0	0	0	0	0	0	0	0	0	1	36.483369	22.402312
1595	1986/E/091	0	0	0	0	0	0	0	0	0	0	0	0	0	0	1	0	0	0	0	0	0	0	0	0	0	0	0	0	0	0	1	1	0	0	1	0	0	0	0	0	0	0	0	0	0	0	0	0	0	0	0	0	0	0	0	0	0	0	0	0	0	0	0	0	0	0	0	1	36.452475	22.464682
1596	1986/E/092	0	0	0	0	0	0	0	0	0	0	0	0	0	1	0	0	0	0	0	0	0	0	0	0	0	0	0	0	0	0	0	0	0	0	1	0	0	0	0	0	0	0	0	0	0	0	0	0	0	0	0	0	0	0	0	0	0	0	0	0	0	0	0	0	0	0	0	1	37.068759	22.375788
1597	1986/E/093	0	0	0	0	0	0	0	0	0	0	0	0	1	0	1	0	0	0	0	0	0	0	0	0	0	1	0	0	0	0	1	0	0	0	1	0	0	0	0	0	0	0	0	0	0	0	0	0	0	0	0	0	0	0	0	0	0	0	0	0	0	0	0	0	0	0	0	1	37.092664	22.352155
1598	1986/E/094	0	0	0	0	0	0	0	0	0	0	0	0	0	0	0	0	0	0	0	0	0	0	0	0	0	0	0	0	0	0	0	0	0	0	1	0	0	0	0	0	0	0	0	0	0	0	0	0	0	0	0	0	0	0	0	0	0	0	0	0	0	0	0	0	0	0	0	1	37.095257	22.333108
1599	1986/E/095	0	0	0	0	0	0	0	0	0	0	0	0	1	0	1	0	0	0	0	0	0	0	0	0	0	0	0	0	0	0	0	0	0	0	1	0	0	0	0	0	0	0	0	0	0	0	0	0	0	0	0	0	0	0	0	0	0	0	0	0	0	0	0	0	0	0	0	1	37.091672	22.324695
1600	1986/E/096	0	0	0	0	0	0	0	0	0	0	0	0	0	0	0	0	0	0	0	0	0	0	0	0	0	0	0	0	0	0	0	0	0	0	1	0	0	0	0	0	0	0	0	0	0	0	0	0	0	0	0	0	0	0	0	0	0	0	0	0	0	0	0	0	0	0	0	1	37.099391	22.232647
1601	1986/E/097	1	0	0	0	0	1	0	0	0	0	0	0	1	1	1	0	0	0	0	0	0	0	0	0	0	0	0	0	0	0	1	1	0	0	1	0	0	0	0	0	0	0	0	0	0	0	0	0	0	0	0	0	0	0	0	0	0	0	0	0	0	0	0	0	0	0	0	1	37.186082	21.924932
1602	1986/E/098	1	0	0	0	0	1	0	0	0	0	0	0	0	1	0	0	0	0	0	0	0	0	0	0	0	0	0	0	0	0	0	0	0	0	1	0	0	0	0	0	0	0	0	0	0	0	0	0	0	0	0	0	0	0	1	0	0	0	0	0	0	0	0	0	0	0	0	1	37.63755	21.629628
1603	1986/E/099	0	0	0	0	0	0	0	0	0	0	0	1	1	0	1	0	0	0	0	0	0	0	0	0	0	0	0	0	0	0	0	0	0	0	0	0	0	0	0	0	0	0	0	0	0	0	0	0	0	0	0	0	0	0	0	0	0	0	0	0	0	0	0	0	0	0	0	1	38.311419	21.78118
1604	1986/E/100	1	0	0	0	0	1	0	0	1	0	0	0	1	1	1	0	0	0	0	0	0	0	0	0	0	0	0	0	0	0	0	0	0	0	1	0	0	0	0	0	0	0	0	0	0	0	0	0	0	0	0	0	0	0	0	0	0	0	0	0	0	0	0	0	0	0	0	1	38.376707	22.080272
1605	1986/E/101	1	0	0	0	0	1	0	0	0	0	0	0	1	1	1	0	0	0	1	0	0	1	0	0	0	0	0	0	0	0	0	0	0	0	0	0	0	0	0	0	0	0	1	0	0	1	0	0	0	0	0	0	0	0	0	0	0	0	0	0	0	0	0	0	0	0	0	1	38.482236	22.501286
1606	1986/E/102	0	0	0	0	0	1	0	0	0	0	0	0	0	1	1	0	0	0	0	0	0	0	0	0	0	0	0	0	0	0	0	0	0	0	0	0	0	0	0	0	0	0	1	0	0	1	0	0	0	0	0	0	0	0	0	0	0	0	0	0	0	0	0	0	0	0	0	1	38.555217	22.406658
1607	1986/E/103	1	0	0	0	0	0	0	0	0	0	0	0	1	0	0	0	0	0	0	0	0	0	0	0	0	0	0	0	0	0	0	0	0	0	1	0	0	0	0	0	0	0	1	0	0	1	0	0	0	0	0	0	0	0	0	0	0	0	0	0	0	0	0	0	0	0	0	1	38.57392	22.348678
1609	1986/E/105	1	0	0	0	0	1	0	0	0	0	0	0	1	0	1	0	0	0	0	0	0	0	0	0	0	0	0	0	0	0	0	1	0	0	1	0	0	0	0	0	0	0	0	0	0	0	0	0	0	0	1	0	0	0	0	0	0	0	0	0	0	0	0	0	0	0	0	1	39.68131	20.898251
1610	1986/E/106	1	0	0	0	0	1	0	0	1	0	0	0	1	1	1	1	0	0	0	0	0	0	0	0	0	0	0	0	0	0	0	0	0	0	0	0	0	0	0	0	0	0	0	0	0	0	0	0	0	0	1	0	0	0	0	0	0	0	0	0	0	0	0	0	0	0	0	1	39.784415	20.738764
1611	1986/E/107	1	0	0	0	0	1	0	0	1	0	0	0	0	0	1	0	0	0	0	0	0	0	0	0	0	0	0	0	0	0	0	0	0	0	0	0	0	0	0	0	0	0	0	0	0	0	0	0	0	0	1	0	0	0	0	0	0	0	0	0	0	0	0	0	0	0	0	1	40.050345	20.749602
1614	1987/D/001	1	0	0	0	0	0	0	0	1	0	0	0	1	0	1	0	0	0	1	0	0	1	0	0	0	0	0	0	0	0	0	1	1	0	0	0	0	0	1	0	0	0	0	0	0	0	0	0	0	0	0	0	0	0	0	0	0	0	0	0	0	0	0	0	0	0	0	1	46.369673	14.100606
1615	1987/D/002	1	0	0	0	0	0	0	0	1	0	0	0	1	0	0	0	0	0	0	0	0	0	0	0	0	0	0	0	0	0	0	0	1	0	0	0	0	0	1	0	0	0	0	0	0	0	0	0	0	0	0	0	0	0	0	0	0	0	0	0	0	0	0	0	0	0	0	1	46.298636	13.943756
1616	1987/D/003	1	0	0	0	0	0	0	0	0	0	0	0	1	0	1	0	0	0	1	0	0	1	0	0	0	0	0	0	0	0	0	0	1	0	0	0	0	0	1	0	0	0	0	0	0	0	0	0	0	0	0	0	0	0	0	0	0	0	0	0	0	0	0	0	0	0	0	1	46.296556	13.961751
1617	1987/D/004	0	0	0	0	0	0	0	0	1	0	0	0	0	0	1	0	0	0	0	0	0	0	0	0	0	0	0	0	0	0	0	1	1	0	0	0	0	0	1	0	0	0	0	0	0	0	0	0	0	0	0	0	0	1	0	0	0	0	0	0	0	0	0	0	0	0	0	1	46.323938	14.596928
1618	1987/D/005	1	0	0	0	0	0	1	0	1	1	0	0	1	0	1	0	0	0	0	0	0	0	0	1	0	0	0	0	0	0	0	1	1	0	0	0	0	0	1	0	0	0	0	0	0	0	0	0	0	0	0	0	0	0	0	0	0	0	0	0	0	0	0	0	0	0	0	1	46.327317	14.589992
1619	1987/D/006	0	0	0	0	0	0	0	0	0	0	0	0	0	0	1	0	0	0	0	0	0	0	0	0	0	0	0	0	0	0	0	1	1	0	0	0	0	0	0	0	0	0	0	0	0	0	0	0	0	0	0	0	0	0	0	0	0	0	0	0	0	0	0	0	0	0	0	1	46.303703	14.657447
1620	1987/D/007	0	0	0	0	0	0	0	0	0	0	0	0	0	0	1	0	0	0	0	0	0	0	0	0	0	0	0	0	0	0	0	0	0	0	0	0	0	0	1	0	0	0	0	0	0	0	0	0	0	0	0	0	0	0	0	0	0	0	0	0	0	0	0	0	0	0	0	1	46.298533	14.643751
1621	1987/D/008	1	0	0	0	0	0	0	0	0	0	0	0	1	0	1	0	0	0	0	0	0	0	0	0	0	0	0	0	0	0	0	0	1	0	0	0	0	0	1	0	0	0	0	0	0	0	0	0	0	0	0	0	0	1	0	0	0	0	0	0	0	0	0	0	0	0	0	1	46.485119	13.785666
1622	1987/D/009	1	0	0	0	0	0	0	0	0	0	1	0	1	0	1	0	0	0	0	0	0	0	0	0	0	0	0	0	0	0	0	1	1	0	0	0	0	0	0	0	0	0	0	0	0	0	0	0	0	0	0	0	0	0	0	0	0	0	0	0	0	0	0	0	0	0	0	1	46.280004	13.551622
1623	1987/D/010	1	0	0	0	0	0	0	0	1	1	0	0	1	1	1	1	0	0	1	0	0	1	0	1	0	0	0	0	0	0	0	1	1	0	0	0	0	0	1	0	0	0	0	0	0	0	0	0	0	0	0	0	0	0	0	0	0	0	0	0	0	0	0	0	0	0	0	1	46.108894	13.900068
1624	1987/D/011	1	0	0	0	0	0	0	0	1	1	0	0	1	0	1	0	0	0	0	0	0	0	0	0	0	0	0	0	0	0	0	1	1	0	0	0	0	0	1	0	0	0	0	0	0	0	0	0	0	0	0	0	0	0	0	0	0	0	0	0	0	0	0	0	0	0	0	1	45.820166	14.290147
1625	1987/D/012	1	0	0	0	0	0	0	0	1	0	0	0	1	0	1	0	0	0	1	0	0	1	0	0	0	0	0	0	0	0	0	1	1	0	0	0	0	0	1	0	0	0	0	0	0	0	0	0	0	0	0	0	0	0	0	0	0	0	0	0	0	0	0	0	0	0	0	1	45.819192	14.236644
1626	1987/D/013	1	0	0	0	0	0	0	0	0	0	0	0	1	0	1	0	0	0	1	0	0	1	0	0	0	0	0	0	0	0	1	0	0	0	0	0	0	0	0	0	0	0	0	0	0	0	0	0	0	0	0	0	0	0	0	0	0	0	0	0	0	0	0	0	0	0	0	1	45.294283	13.598113
1627	1987/D/014	1	0	0	0	0	0	0	0	0	0	1	0	1	0	1	0	0	0	1	0	0	1	0	0	0	0	0	0	0	0	1	0	1	0	0	0	0	0	0	0	0	0	0	0	0	0	0	0	0	0	0	0	0	0	0	0	0	0	0	0	0	0	0	0	0	0	0	1	45.364321	13.758298
1628	1987/D/015	1	0	0	0	0	1	0	0	0	1	0	0	1	0	1	0	0	0	1	0	0	1	1	0	0	1	0	0	0	0	1	0	1	0	0	0	0	0	0	0	0	0	0	0	1	0	0	1	0	0	0	0	0	0	0	0	0	0	0	0	0	0	0	0	0	0	0	1	45.377726	13.883069
1629	1987/D/016	1	0	0	0	0	0	0	0	0	0	0	0	0	0	0	0	0	0	1	0	0	1	0	0	0	0	0	0	0	0	1	0	0	0	0	0	0	0	0	0	0	0	0	0	0	0	0	0	0	0	0	0	0	0	0	0	0	0	0	0	0	0	0	0	0	0	0	1	45.086872	14.088397
1630	1987/D/017	0	0	0	0	0	1	0	0	0	0	0	0	0	0	1	0	0	0	1	0	0	1	1	0	0	0	0	0	0	0	1	0	0	0	0	0	0	0	0	0	0	0	0	0	0	0	0	0	0	0	0	0	0	1	0	0	0	0	0	0	0	0	0	0	0	0	0	1	45.521615	13.869281
1631	1987/D/018	1	0	1	0	0	1	1	0	0	1	0	0	1	0	1	0	1	0	1	1	0	1	1	0	0	1	0	1	1	0	1	0	1	0	0	0	0	1	0	0	0	0	0	0	1	0	0	1	0	0	0	0	0	0	0	0	0	0	0	0	0	0	0	0	0	0	0	1	45.463534	13.89747
1634	1987/D/021	0	0	0	0	0	0	0	0	0	0	0	0	1	0	1	0	0	0	0	0	0	0	0	0	0	0	0	0	0	0	0	1	0	0	0	0	0	0	0	0	0	0	0	0	0	0	0	0	0	0	0	0	0	0	0	0	0	0	0	0	0	0	0	0	0	0	0	1	44.52652	15.091466
1635	1987/D/022	1	0	0	0	0	0	0	0	0	0	0	0	0	0	0	0	0	0	1	0	0	1	0	0	0	0	0	0	0	0	0	0	0	0	0	0	0	0	0	1	0	0	0	0	1	0	0	1	0	0	0	0	0	0	0	0	0	0	0	0	0	0	0	0	0	0	0	1	44.526272	15.171664
1636	1987/D/023	1	0	0	0	0	0	0	0	0	0	0	0	1	0	0	0	0	0	1	0	0	1	0	0	0	0	0	0	0	0	0	1	0	0	0	0	0	0	0	1	1	0	0	0	0	0	0	0	0	0	0	0	0	0	0	0	0	0	0	0	0	0	0	0	0	0	0	1	44.248375	15.809073
1637	1987/D/024	1	0	0	0	0	0	0	0	0	0	0	0	1	0	0	0	0	0	1	0	0	1	0	0	0	0	0	0	0	0	1	0	0	0	0	0	0	0	0	0	0	0	0	0	0	0	0	0	0	0	0	0	0	0	0	0	0	0	0	0	0	0	0	0	0	0	0	1	44.220206	15.736781
1639	1987/D/026	1	0	0	0	0	1	0	0	0	1	0	0	1	0	1	0	0	0	1	0	0	1	0	0	0	0	0	0	0	0	0	1	0	0	0	0	0	0	1	0	0	0	0	0	0	0	0	0	0	1	0	0	0	0	0	0	0	0	0	0	0	0	0	0	0	0	0	1	45.265949	15.223881
1640	1987/D/027	1	0	0	0	0	0	0	0	0	0	0	0	0	0	1	0	0	0	0	0	0	0	0	0	0	0	0	0	0	0	0	0	0	0	0	0	0	0	0	0	0	0	0	0	0	0	0	0	0	1	0	0	0	0	0	0	0	0	0	0	0	0	0	0	0	0	0	1	45.227777	15.040426
1641	1987/D/028	1	0	0	0	1	0	0	0	1	0	0	0	0	0	1	1	0	0	0	0	0	0	0	1	0	1	0	1	1	0	0	1	1	0	0	0	0	0	1	0	0	0	0	0	0	0	0	0	0	0	0	0	0	0	0	0	0	0	0	0	0	0	0	0	0	0	0	1	44.99815	15.131953
1642	1987/D/029	1	0	0	0	0	0	0	0	0	0	0	0	1	0	0	0	0	0	0	0	0	0	1	0	0	0	0	0	0	0	1	0	0	0	0	0	0	0	0	0	0	0	0	0	1	0	0	1	0	0	0	0	0	0	0	0	0	0	0	0	0	0	0	0	0	0	0	1	44.985169	14.923147
1644	1988/C/062	0	0	0	0	0	0	0	0	0	0	0	0	0	1	0	0	0	0	0	0	0	0	0	0	0	0	0	0	0	0	0	0	0	0	0	0	0	0	0	0	0	0	0	0	0	0	0	0	0	0	0	0	0	0	0	0	0	0	0	0	0	0	0	0	0	0	0	1	38.016204	22.946963
1645	1988/C/063	1	0	0	0	0	0	0	0	0	1	0	0	0	1	0	0	0	0	0	0	0	0	0	0	0	0	0	0	0	0	0	0	0	0	0	0	0	0	0	0	0	0	0	0	0	0	0	0	0	0	0	0	0	0	0	0	0	0	0	0	0	0	0	0	0	0	0	1	38.027857	22.853764
1646	1988/C/064	1	0	0	0	0	0	0	0	0	0	0	0	1	1	0	0	0	0	1	0	0	1	0	0	0	0	0	0	0	0	0	0	0	0	0	0	0	0	0	0	0	0	0	0	0	0	0	0	0	0	0	0	0	0	0	0	0	0	0	0	0	0	0	0	0	0	0	1	37.764578	23.124674
1647	1988/C/065	1	0	0	0	0	0	0	0	0	0	0	0	1	1	0	0	0	0	0	0	0	0	0	0	0	0	0	0	0	0	0	0	0	0	0	0	0	0	0	0	0	0	0	0	0	0	0	0	0	0	0	0	0	0	0	0	0	0	0	0	0	0	0	0	0	0	0	1	37.67959	23.134569
1648	1988/C/066	0	0	0	0	0	0	0	0	0	0	0	0	1	1	0	0	0	0	0	0	0	0	0	0	0	0	0	0	0	0	0	0	0	0	0	0	0	0	0	0	0	0	0	0	0	0	0	0	0	0	0	0	0	0	0	0	0	0	0	0	0	0	0	0	0	0	0	1	37.570801	23.149432
1649	1988/C/067	0	0	0	0	0	0	0	0	0	0	0	0	0	1	0	0	0	0	0	0	0	0	0	0	0	0	0	0	0	0	0	0	0	0	0	0	0	0	0	0	0	0	0	0	0	0	0	0	0	0	0	0	0	0	0	0	0	0	0	0	0	0	0	0	0	0	0	1	37.557065	23.374457
1650	1988/C/068	0	0	0	0	0	0	0	0	0	0	0	0	0	1	0	0	0	0	0	0	0	0	0	0	0	0	0	0	0	0	0	0	0	0	0	0	0	0	0	0	0	0	0	0	0	0	0	0	0	0	0	0	0	0	0	0	0	0	0	0	0	0	0	0	0	0	0	1	37.397067	23.241321
1651	1988/C/069	0	0	0	0	0	0	0	0	0	1	0	0	0	1	0	0	0	0	0	0	0	0	0	0	0	0	0	0	0	0	0	0	0	0	0	0	0	0	0	0	0	0	0	0	0	0	0	0	0	0	0	0	0	0	0	0	0	0	0	0	0	0	0	0	0	0	0	1	37.521176	22.859773
1652	1988/C/070	0	0	0	0	0	0	0	0	0	0	0	0	0	1	0	0	0	0	0	0	0	0	0	0	0	0	0	0	0	0	0	0	0	0	0	0	0	0	0	0	0	0	0	0	0	0	0	0	0	0	0	0	0	0	0	0	0	0	0	0	0	0	0	0	0	0	0	1	37.513674	22.736441
1653	1988/C/071	1	0	0	0	0	0	0	0	0	0	0	0	0	1	0	0	0	0	0	0	0	0	0	0	0	0	0	0	0	0	0	0	0	0	0	0	0	0	0	0	0	0	0	0	0	0	0	0	0	0	0	0	0	0	0	0	0	0	0	0	0	0	0	0	0	0	0	1	37.383882	22.744632
1654	1988/C/072	1	0	0	0	0	0	0	0	0	0	0	0	0	1	0	0	0	0	0	0	0	0	0	0	0	0	0	0	0	0	0	0	0	0	0	0	0	0	0	0	0	0	0	0	0	0	0	0	0	0	0	0	0	0	0	0	0	0	0	0	0	0	0	0	0	0	0	1	37.339378	22.792704
1655	1988/C/073	0	0	0	0	0	0	0	0	0	0	0	0	0	1	0	0	0	0	0	0	0	0	0	0	0	0	0	0	0	0	0	0	0	0	0	0	0	0	0	0	0	0	0	0	0	0	0	0	0	0	0	0	0	0	0	0	0	0	0	0	0	0	0	0	0	0	0	1	37.146349	22.892263
1656	1988/C/074	0	0	0	0	0	0	0	0	0	0	0	0	1	1	0	0	0	0	0	0	0	0	0	0	0	0	0	0	0	0	0	0	0	0	0	0	0	0	0	0	0	0	0	0	0	0	0	0	0	0	0	0	0	0	0	0	0	0	0	0	0	0	0	0	0	0	0	1	37.098999	22.870154
1657	1988/C/075	0	0	0	0	0	0	0	0	0	0	0	0	0	1	0	0	0	0	0	0	0	0	0	0	0	0	0	0	0	0	0	0	0	0	0	0	0	0	0	0	0	0	0	0	0	0	0	0	0	0	0	0	0	0	0	0	0	0	0	0	0	0	0	0	0	0	0	1	37.164461	22.839021
1658	1988/C/076	0	0	0	0	0	0	0	0	0	0	0	0	0	1	0	0	0	0	0	0	0	0	0	0	0	0	0	0	0	0	0	0	0	0	0	0	0	0	0	0	0	0	0	0	0	0	0	0	0	0	0	0	0	0	0	0	0	0	0	0	0	0	0	0	0	0	0	1	37.163004	22.810529
1660	1988/C/078	0	0	0	0	0	0	0	0	0	0	0	0	0	1	0	0	0	0	0	0	0	0	0	0	0	0	0	0	0	0	0	0	0	0	0	0	0	0	0	0	0	0	0	0	0	0	0	0	0	0	0	0	0	0	0	0	0	0	0	0	0	0	0	0	0	0	0	1	37.025115	22.554651
1661	1988/C/079	0	0	0	0	0	0	0	0	0	0	0	0	0	1	0	0	0	0	0	0	0	0	0	0	0	0	0	0	0	0	0	0	0	0	0	0	0	0	0	0	0	0	0	0	0	0	0	0	0	0	0	0	0	0	0	0	0	0	0	0	0	0	0	0	0	0	0	1	37.008464	22.649885
1662	1988/C/080	0	0	0	0	0	0	0	0	0	0	0	0	1	0	0	0	0	0	0	0	0	0	0	0	0	0	0	0	0	0	0	0	0	0	0	0	0	0	0	0	0	0	0	0	0	0	0	0	0	0	0	0	0	0	0	0	0	0	0	0	0	0	0	0	0	0	0	1	37.081998	22.282809
1664	1988/C/082	0	0	0	0	0	0	0	0	0	0	0	0	1	0	0	0	0	0	0	0	0	0	0	0	0	0	0	0	0	0	0	0	0	0	0	0	0	0	0	0	0	0	0	0	0	0	0	0	0	0	0	0	0	0	0	0	0	0	0	0	0	0	0	0	0	0	0	1	37.49026	22.349446
1666	1988/C/084	1	0	0	0	0	0	0	0	0	0	0	0	0	0	0	0	0	0	0	0	0	0	0	0	0	0	0	0	0	0	0	0	0	0	0	0	0	0	0	0	0	0	0	0	0	0	0	0	0	0	0	0	0	0	0	0	0	0	0	0	0	0	0	0	0	0	0	1	37.487291	21.917913
1667	1988/C/085	1	0	0	0	0	0	0	0	0	0	0	0	0	1	0	0	0	0	0	0	0	0	0	0	0	0	0	0	0	0	0	0	0	0	0	0	0	0	0	0	0	0	0	0	0	0	0	0	0	0	0	0	0	0	0	0	0	0	0	0	0	0	0	0	0	0	0	1	37.448929	21.910396
1671	1992/A/002	1	0	0	0	0	0	0	0	0	0	0	0	1	1	0	0	0	0	1	0	0	1	0	0	0	0	0	0	0	0	0	0	0	0	0	0	0	0	0	0	0	0	0	0	0	0	0	0	0	0	0	0	0	0	0	0	0	0	0	0	0	0	0	0	0	0	0	1	37.692119	23.139573
1674	1992/A/005	1	0	0	0	0	0	0	0	0	0	0	0	1	1	0	0	0	0	1	0	0	1	0	0	0	0	0	0	1	0	0	0	0	0	0	0	0	0	0	0	0	0	0	0	0	0	0	0	1	0	0	0	0	0	0	0	0	0	0	0	0	0	0	0	0	0	0	1	37.889167	22.869722
1675	1992/A/006	1	0	0	0	0	0	0	0	0	0	0	0	0	1	0	0	0	0	0	0	0	0	0	0	0	0	0	0	0	0	0	0	0	0	0	0	0	0	0	0	0	0	0	0	0	0	0	0	0	0	0	0	0	0	0	0	0	0	0	0	0	0	0	0	0	0	0	1	37.982925	22.97693
1677	1992/A/008	1	0	0	0	0	0	0	0	0	0	0	0	0	1	0	0	0	0	0	0	0	0	0	0	0	0	0	0	0	0	0	0	0	0	0	0	0	0	0	0	0	0	0	0	0	0	0	0	0	0	0	0	0	0	0	0	0	0	0	0	0	0	0	0	0	0	0	1	37.840649	22.899091
1679	1992/A/010	1	0	0	0	0	0	0	0	0	0	0	0	1	1	0	0	0	0	1	0	0	1	0	0	0	0	0	0	0	0	0	0	0	0	0	0	0	0	0	0	0	0	0	0	0	0	0	0	0	0	0	0	0	0	0	0	0	0	0	0	0	0	0	0	0	0	0	1	37.76	22.88
1680	1992/A/011	0	0	0	0	0	0	0	0	0	0	0	0	1	0	0	0	0	0	0	0	0	0	0	0	0	0	0	0	0	0	0	0	0	0	0	0	0	0	0	0	0	0	0	0	0	0	0	0	0	0	0	0	0	0	0	0	0	0	0	0	0	0	0	0	0	0	0	1	37.753545	22.877767
1681	1992/A/012	1	0	0	0	0	0	0	0	0	0	0	0	1	1	0	0	0	0	0	0	0	0	0	0	0	0	0	0	0	0	0	0	0	0	0	0	0	0	0	0	0	0	0	0	0	0	0	0	0	0	0	0	0	0	0	0	0	0	0	0	0	0	0	0	0	0	0	1	37.756399	22.819928
1682	1992/A/013	1	0	0	0	0	0	0	0	0	0	0	0	1	1	0	0	0	0	0	0	0	0	0	0	0	0	0	0	0	0	0	0	0	0	0	0	0	0	0	0	0	0	0	0	0	0	0	0	0	0	0	0	0	0	0	0	0	0	0	0	0	0	0	0	0	0	0	1	37.739199	22.878892
1684	1992/A/015	1	0	0	0	0	0	0	0	0	0	0	0	1	1	0	0	0	0	1	0	0	1	0	0	0	0	0	0	0	0	0	0	0	0	0	0	0	0	0	0	0	0	0	0	0	0	0	0	0	0	0	0	0	0	0	0	0	0	0	0	0	0	0	0	0	0	0	1	37.710454	22.83472
1685	1992/A/016	0	0	0	0	0	0	0	0	0	0	0	0	1	1	0	0	0	0	1	0	0	1	0	0	0	0	0	0	0	0	0	0	0	0	0	0	0	0	0	0	0	0	0	0	0	0	0	0	0	0	0	0	0	0	0	0	0	0	0	0	0	0	0	0	0	0	0	1	37.638091	22.715355
1686	1992/A/017	0	0	0	0	0	0	0	0	0	0	0	0	0	1	0	0	0	0	1	0	0	1	0	0	0	0	0	0	0	0	0	0	0	0	0	0	0	0	0	0	0	0	0	0	0	0	0	0	0	0	0	0	0	0	0	0	0	0	0	0	0	0	0	0	0	0	0	1	37.520101	22.729946
1688	1992/A/019	0	0	0	0	0	0	0	0	0	0	0	0	1	1	0	0	0	0	1	0	0	1	0	0	0	0	0	0	0	0	0	0	0	0	0	0	0	0	0	0	0	0	0	0	0	0	0	0	0	0	0	0	0	0	0	0	0	0	0	0	0	0	0	0	0	0	0	1	37.339458	22.763359
1689	1992/A/020	1	0	0	0	0	0	0	0	0	0	0	0	1	1	0	0	0	0	1	0	0	1	0	0	0	0	0	0	1	0	0	0	0	0	0	0	0	0	0	0	0	0	0	0	0	0	0	0	0	0	0	0	0	0	0	0	0	0	0	0	0	0	0	0	0	0	0	1	37.328701	22.754276
1690	1992/A/021	0	0	0	0	0	0	0	0	0	0	0	0	0	1	0	0	0	0	0	0	0	0	0	0	0	0	0	0	0	0	0	0	0	0	0	0	0	0	0	0	0	0	0	0	0	0	0	0	0	0	0	0	0	0	0	0	0	0	0	0	0	0	0	0	0	0	0	1	37.264714	22.846941
1693	1992/A/024	0	0	0	0	0	0	0	0	0	0	0	0	0	1	0	0	0	0	1	0	0	1	0	0	0	0	0	0	0	0	0	0	0	0	0	0	0	0	0	0	0	0	0	0	0	0	0	0	0	0	0	0	0	0	0	0	0	0	0	0	0	0	0	0	0	0	0	1	37.152598	22.793423
1694	1992/A/025	0	0	0	0	0	0	0	0	0	0	0	0	1	1	0	0	0	0	0	0	0	0	0	0	0	0	0	0	0	0	0	0	0	0	0	0	0	0	0	0	0	0	0	0	0	0	0	0	0	0	0	0	0	0	0	0	0	0	0	0	0	0	0	0	0	0	0	1	37.143151	22.773645
1703	1992/A/034	0	0	0	0	0	0	0	0	0	0	0	0	0	1	0	0	0	0	0	0	0	0	0	0	0	0	0	0	0	0	0	0	0	0	0	0	0	0	0	0	0	0	0	0	0	0	0	0	0	0	0	0	0	0	0	0	0	0	0	0	0	0	0	0	0	0	0	1	37.322857	22.14248
1704	1992/A/035	0	0	0	0	0	0	0	0	0	0	0	0	0	1	0	0	0	0	0	0	0	0	0	0	0	0	0	0	0	0	0	0	0	0	0	0	0	0	0	0	0	0	0	0	0	0	0	0	0	0	0	0	0	0	0	0	0	0	0	0	0	0	0	0	0	0	0	1	37.319287	22.156376
1705	1992/A/036	1	0	0	0	0	0	0	0	0	0	0	0	0	1	0	0	0	0	1	0	0	1	0	0	0	0	0	0	0	0	0	0	0	0	0	0	0	0	0	0	0	0	0	0	0	0	0	0	0	0	0	0	0	0	0	0	0	0	0	0	0	0	0	0	0	0	0	1	37.26833	22.21764
1706	1992/A/037	0	0	0	0	0	0	0	0	0	0	0	0	1	0	0	0	0	0	0	0	0	0	0	0	0	0	0	0	0	0	0	0	0	0	0	0	0	0	0	0	0	0	0	0	0	0	0	0	0	0	0	0	0	0	0	0	0	0	0	0	0	0	0	0	0	0	0	1	37.2443	22.247248
1709	1992/A/040	0	0	0	0	0	0	0	0	0	0	0	0	0	1	0	0	0	0	0	0	0	0	0	0	0	0	0	0	0	0	0	0	0	0	0	0	0	0	0	0	0	0	0	0	0	0	0	0	0	0	0	0	0	0	0	0	0	0	0	0	0	0	0	0	0	0	0	1	37.026496	22.539432
1712	1992/A/043	0	0	0	0	0	0	0	0	0	0	0	0	0	1	0	0	0	0	0	0	0	0	0	0	0	0	0	0	0	0	0	0	0	0	0	0	0	0	0	0	0	0	0	0	0	0	0	0	0	0	0	0	0	0	0	0	0	0	0	0	0	0	0	0	0	0	0	1	36.939206	22.773784
1714	1992/A/045	1	0	0	0	0	0	0	0	0	0	0	0	0	1	0	0	0	0	0	0	0	0	0	0	0	0	0	0	0	0	0	0	0	0	0	0	0	0	0	0	0	0	0	0	0	0	0	0	0	0	0	0	0	0	0	0	0	0	0	0	0	0	0	0	0	0	0	1	36.688618	23.054773
1715	1992/A/046	1	0	0	0	0	0	0	0	0	0	0	0	0	1	0	0	0	0	1	0	0	1	0	0	0	0	0	0	0	0	0	0	0	0	0	0	0	0	0	0	0	0	0	0	0	0	0	0	0	0	0	0	0	0	0	0	0	0	0	0	0	0	0	0	0	0	0	1	36.688185	23.04962
1716	1992/A/047	0	0	0	0	0	0	0	0	0	0	0	0	0	1	0	0	0	0	0	0	0	0	0	0	0	0	0	0	0	0	0	0	0	0	0	0	0	0	0	0	0	0	0	0	0	0	0	0	0	0	0	0	0	0	0	0	0	0	0	0	0	0	0	0	0	0	0	1	36.773364	22.836695
1717	1992/A/048	1	0	0	0	0	0	0	0	0	0	0	0	0	1	0	0	0	0	0	0	0	0	0	0	0	0	0	0	0	0	0	0	0	0	0	0	0	0	0	0	0	0	0	0	0	0	0	0	0	0	0	0	0	0	0	0	0	0	0	0	0	0	0	0	0	0	0	1	36.801821	22.492526
1718	1992/A/049	0	0	0	0	0	0	0	0	0	0	0	0	0	0	0	0	0	0	0	0	0	0	0	0	0	0	0	0	0	0	0	0	0	0	0	0	0	0	0	0	0	0	0	0	0	0	0	0	0	0	0	0	0	0	0	0	0	0	0	0	0	0	0	0	0	0	0	1	36.815865	22.461376
1719	1992/A/050	0	0	0	0	0	0	0	0	0	0	0	0	0	1	0	0	0	0	0	0	0	0	0	0	0	0	0	0	0	0	0	0	0	0	0	0	0	0	0	0	0	0	0	0	0	0	0	0	0	0	0	0	0	0	0	0	0	0	0	0	0	0	0	0	0	0	0	1	36.978042	22.428122
1720	1992/A/051	0	0	0	0	0	0	0	0	0	0	0	0	1	0	0	0	0	0	0	0	0	0	0	0	0	0	0	0	0	0	0	0	0	0	0	0	0	0	0	0	0	0	0	0	0	0	0	0	0	0	0	0	0	0	0	0	0	0	0	0	0	0	0	0	0	0	0	1	36.966356	22.388656
1721	1992/A/052	0	0	0	0	0	0	0	0	0	0	0	0	0	1	0	0	0	0	0	0	0	0	0	0	0	0	0	0	0	0	0	0	0	0	0	0	0	0	0	0	0	0	0	0	0	0	0	0	0	0	0	0	0	0	0	0	0	0	0	0	0	0	0	0	0	0	0	1	36.669997	22.381176
1722	1992/A/053	0	0	0	0	0	0	0	0	0	0	0	0	0	1	0	0	0	0	0	0	0	0	0	0	0	0	0	0	0	0	0	0	0	0	0	0	0	0	0	0	0	0	0	0	0	0	0	0	0	0	0	0	0	0	0	0	0	0	0	0	0	0	0	0	0	0	0	1	36.638337	22.380651
1723	1992/A/054	1	0	0	0	0	0	0	0	0	0	0	0	1	0	0	0	0	0	0	0	0	0	0	0	0	0	0	0	0	0	0	0	0	0	0	0	0	0	0	0	0	0	0	0	0	0	0	0	0	0	0	0	0	0	0	0	0	0	0	0	0	0	0	0	0	0	0	1	36.770166	22.346468
1724	1992/A/055	0	0	0	0	0	0	0	0	0	0	0	0	1	1	0	0	0	0	0	0	0	0	0	0	0	0	0	0	0	0	0	0	0	0	0	0	0	0	0	0	0	0	0	0	0	0	0	0	0	0	0	0	0	0	0	0	0	0	0	0	0	0	0	0	0	0	0	1	36.869246	22.244546
1726	1992/A/057	1	0	0	0	0	0	0	0	0	0	0	0	0	1	0	0	0	0	0	0	0	0	0	0	0	0	0	0	0	0	0	0	0	0	0	0	0	0	0	0	0	0	0	0	0	0	0	0	0	0	0	0	0	0	0	0	0	0	0	0	0	0	0	0	0	0	0	1	37.648027	21.73378
1727	1992/A/058	0	0	0	0	0	0	0	0	0	0	0	0	0	1	0	0	0	0	0	0	0	0	0	0	0	0	0	0	0	0	0	0	0	0	0	0	0	0	0	0	0	0	0	0	0	0	0	0	0	0	0	0	0	0	0	0	0	0	0	0	0	0	0	0	0	0	0	1	37.701074	21.909719
1728	1992/A/059	0	0	0	0	0	0	0	0	0	0	0	0	0	0	0	0	0	0	0	0	0	0	0	0	0	0	0	0	0	0	0	0	0	0	0	0	0	0	0	0	0	0	0	0	0	0	0	0	0	0	0	0	0	0	0	0	0	0	0	0	0	0	0	0	0	0	0	1	37.693193	21.980218
1730	1992/A/061	0	0	0	0	0	0	0	0	0	0	0	0	1	1	0	0	0	0	0	0	0	0	0	0	0	0	0	0	0	0	0	0	0	0	0	0	0	0	0	0	0	0	0	0	0	0	0	0	0	0	0	0	0	0	0	0	0	0	0	0	0	0	0	0	0	0	0	1	37.674799	22.129208
1731	1992/A/062	0	0	0	0	0	0	0	0	0	0	0	0	1	0	0	0	0	0	0	0	0	0	0	0	0	0	0	0	0	0	0	0	0	0	0	0	0	0	0	0	0	0	0	0	0	0	0	0	0	0	0	0	0	0	0	0	0	0	0	0	0	0	0	0	0	0	0	1	37.824065	22.142862
1734	1992/A/065	1	0	0	0	0	0	0	0	0	0	0	0	0	1	0	0	0	0	0	0	0	0	0	0	0	0	0	0	0	0	0	0	0	0	0	0	0	0	0	0	0	0	0	0	0	0	0	0	0	0	0	0	0	0	0	0	0	0	0	0	0	0	0	0	0	0	0	1	38.054329	22.138124
1735	1992/A/066	1	0	0	0	0	0	0	0	0	0	0	0	1	1	0	0	0	0	1	0	0	1	0	0	0	0	0	0	0	0	0	0	0	0	0	0	0	0	0	0	0	0	0	0	0	0	0	0	0	0	0	0	0	0	0	0	0	0	0	0	0	0	0	0	0	0	0	1	38.368601	22.127928
1736	1992/A/067	0	0	0	0	0	0	0	0	0	0	0	0	1	0	0	0	0	0	0	0	0	0	0	0	0	0	0	0	0	0	0	0	0	0	0	0	0	0	0	0	0	0	0	0	0	0	0	0	0	0	0	0	0	0	0	0	0	0	0	0	0	0	0	0	0	0	0	1	38.34786	22.313012
1738	1992/A/069	1	0	0	0	0	0	0	0	0	0	0	0	1	1	0	0	0	0	1	0	0	1	0	0	0	0	0	0	0	0	0	0	0	0	0	0	0	0	0	0	0	0	0	0	0	0	0	0	0	0	0	0	0	0	0	0	0	0	0	0	0	0	0	0	0	0	0	1	38.452649	22.70147
1739	1992/A/070	1	0	0	0	0	0	0	0	0	0	0	0	0	1	0	0	0	0	0	0	0	0	0	0	0	0	0	0	0	0	0	0	0	0	0	0	0	0	0	0	0	0	0	0	0	0	0	0	0	0	0	0	0	0	0	0	0	0	0	0	0	0	0	0	0	0	0	1	38.171966	23.375409
1740	1994/01	1	0	0	0	0	0	0	0	0	0	0	0	1	1	0	0	0	0	1	0	0	1	0	0	0	0	0	0	0	0	0	0	0	0	0	0	0	0	0	0	0	0	0	0	0	0	0	0	0	0	0	0	0	0	0	0	0	0	0	0	0	0	0	0	0	0	0	1	45.781	14.205
1741	1994/02	1	0	0	0	0	0	0	0	0	0	0	0	0	0	0	0	0	0	0	0	0	0	0	0	0	0	0	0	1	0	1	0	0	0	0	0	0	0	0	0	0	0	0	0	0	0	0	0	0	0	0	0	0	0	0	0	0	0	0	0	0	0	0	0	0	0	0	1	45.64	14.17
1742	1994/03	1	0	0	0	0	0	0	0	0	0	0	0	1	0	0	0	0	0	1	0	0	1	0	0	0	0	0	0	1	0	1	0	1	0	0	0	0	0	0	0	0	0	0	0	0	0	0	0	0	0	0	0	0	0	0	0	0	0	0	0	0	0	0	0	0	0	0	1	45.51	14.29
1744	1994/05	1	0	0	0	0	0	0	0	0	0	0	0	0	0	0	0	0	0	1	0	0	1	0	0	0	0	0	0	0	0	0	0	0	0	0	0	0	0	0	0	0	0	0	0	0	0	0	0	0	0	0	0	0	0	0	0	0	0	0	0	0	0	0	0	0	0	0	1	45.13	14.83
1745	1994/06	0	0	0	0	0	0	0	0	0	0	0	0	1	0	0	1	0	0	1	0	0	1	1	0	0	0	0	0	0	0	1	0	1	0	0	0	0	0	0	0	0	0	0	0	0	0	0	0	0	0	0	0	0	0	0	0	0	0	0	0	0	0	0	0	0	0	0	1	44.98	14.99
1746	1994/07	1	0	0	0	0	0	0	0	0	0	0	0	1	0	0	0	0	0	1	0	0	1	0	0	0	0	0	0	0	0	1	0	0	0	0	0	0	0	0	0	0	0	0	0	0	0	0	0	0	0	0	1	0	0	0	0	0	0	0	0	0	0	0	0	0	0	0	1	44.99	14.98
1747	1994/08	1	0	0	0	0	0	0	0	0	1	0	0	1	0	0	0	0	0	1	0	0	1	1	0	0	0	0	0	0	0	1	0	0	0	0	0	0	0	0	0	0	0	0	0	0	0	0	0	0	0	0	0	0	0	1	0	0	0	0	0	0	0	0	0	0	0	0	1	44.78	14.76
1748	1994/09	1	0	0	0	0	1	0	0	0	0	0	0	1	0	0	0	0	0	1	0	0	1	0	0	0	0	0	0	0	0	1	0	0	0	0	0	0	0	0	0	0	0	0	0	0	1	0	0	0	0	1	0	0	1	0	0	0	0	0	0	0	0	0	0	0	0	0	1	44.72	14.92
1749	1994/10	1	0	0	0	0	0	0	0	0	0	0	0	0	0	0	0	0	0	1	0	0	1	0	0	0	0	0	0	0	0	1	0	0	0	0	0	0	0	0	0	0	0	0	0	0	1	0	0	1	0	0	0	0	0	0	0	0	0	0	0	0	0	0	0	0	0	0	1	45.12	14.83
1750	1994/11	1	0	0	0	0	0	0	0	0	0	0	0	0	0	0	0	0	0	0	0	0	0	0	0	0	0	0	0	0	0	0	0	0	0	0	0	0	0	0	1	0	0	0	0	0	0	0	0	0	0	0	0	0	0	0	0	0	0	0	0	0	0	0	0	0	0	0	1	45.31	14.72
1751	1994/12	1	0	0	0	0	0	0	0	0	0	0	0	1	0	0	0	0	0	1	0	0	1	0	0	0	1	0	0	0	0	1	0	0	0	0	0	0	0	0	0	0	0	0	0	0	0	0	0	0	0	0	0	0	0	0	0	0	0	0	0	0	0	0	0	0	0	0	3	45.36	14.77
1752	1994/13	1	0	0	0	0	0	0	0	0	1	0	0	0	0	0	0	0	0	1	0	0	1	0	0	0	0	0	0	0	0	1	0	0	0	0	0	0	0	0	0	0	0	0	0	0	0	0	0	0	0	0	0	0	0	0	0	0	0	0	0	0	0	0	0	0	0	0	1	45.35	13.76
1753	1994/14	1	0	0	0	0	1	0	0	0	0	0	0	1	0	0	0	0	0	1	0	0	1	0	0	0	0	0	0	0	0	1	0	0	0	0	0	0	0	0	0	0	0	0	0	0	0	0	0	0	0	0	0	0	0	0	0	0	0	0	0	0	0	0	0	0	0	0	1	45.36	13.75
1754	1994/15	1	0	0	0	0	0	0	0	0	0	0	0	0	0	0	0	0	0	1	0	0	1	0	0	0	1	0	0	0	0	0	0	1	0	0	0	0	0	0	0	0	0	0	0	0	0	0	0	0	0	0	0	0	0	0	0	0	0	0	0	0	0	0	0	0	0	0	1	45.13	13.76
1760	1995/40	0	0	0	0	0	0	0	0	0	0	0	0	0	0	0	0	0	0	1	0	0	1	0	0	0	0	0	0	0	0	0	0	0	0	0	0	0	0	0	0	0	0	0	0	0	0	0	0	0	0	0	0	0	0	0	0	0	0	0	0	0	0	0	0	0	0	0	1	44.91	22.37
1761	1995/41	0	0	0	0	0	0	0	0	0	0	0	0	0	0	0	0	0	0	0	0	0	0	0	0	0	0	0	0	0	0	0	0	1	0	0	0	0	0	0	0	0	0	0	0	0	0	0	0	0	0	0	0	0	0	0	0	0	0	0	0	0	0	0	0	0	0	0	1	45.2	22.32
1763	1995/43	0	0	0	0	0	1	0	0	0	0	0	0	1	1	0	0	0	0	0	0	0	0	0	0	0	0	0	0	0	0	0	0	1	0	0	0	0	1	0	0	0	0	0	0	0	0	0	0	0	0	0	0	0	0	0	0	0	0	0	0	0	0	0	0	0	0	0	1	41.328	23.354
1764	1995/44	0	0	0	0	0	0	0	0	0	0	0	0	0	1	0	0	0	0	0	0	0	0	0	0	0	0	0	0	0	0	0	0	1	0	0	0	0	0	0	0	0	0	0	0	0	0	0	0	0	0	0	0	0	0	0	0	0	0	0	0	0	0	0	0	0	0	0	1	40.719	23.65
1766	1995/46	0	0	0	0	0	1	0	0	0	0	0	0	0	0	0	0	0	0	1	0	0	1	0	0	0	1	0	0	0	0	0	0	1	0	0	0	0	0	0	0	0	0	1	0	0	0	1	0	0	0	0	0	0	0	0	0	0	0	0	0	0	0	0	0	0	0	0	1	40.886	24.072
1767	1995/47	0	0	0	0	0	1	0	0	0	0	0	0	0	1	0	0	0	0	0	0	0	0	0	0	0	1	0	0	0	0	0	0	0	0	0	0	0	0	0	0	0	0	1	0	0	0	1	0	0	0	0	0	0	0	0	0	0	0	0	0	0	0	0	0	0	0	0	1	40.899	24.045

1768	1995/48	0	0	0	0	0	1	0	0	0	0	0	0	1	0	0	0	0	0	1	0	0	0	0	0	0	0	0	0	0	1	0	0	0	0	0	0	0	0	0	0	1	0	0	0	0	0	0	0	0	0	0	0	0	0	0	0	0	0	0	0	0	3	40.913	24.072
1770	1995/50	0	0	0	0	0	1	0	0	0	0	0	0	0	1	0	0	0	0	1	0	0	0	1	0	0	0	0	0	0	0	0	0	0	1	0	0	0	0	0	0	0	0	0	0	0	0	0	0	0	0	0	0	0	0	0	0	0	0	0	0	0	1	40.799	23.928
1771	1995/51	1	0	0	0	0	1	0	0	0	0	0	0	0	1	0	0	0	0	0	0	0	0	0	0	0	0	0	0	0	0	0	0	0	0	0	0	0	0	0	0	0	0	0	0	0	0	0	0	0	0	0	0	0	0	0	0	0	0	0	0	0	1	40.725	23.695
1772	1995/52	1	0	0	0	0	1	0	0	0	1	0	0	0	1	0	0	0	0	0	0	0	0	0	0	0	0	0	0	0	1	0	0	0	1	0	0	0	0	0	0	1	0	0	0	0	0	0	0	0	0	0	0	0	0	0	0	0	0	0	0	0	1	40.752	24.09
1773	1995/53	1	0	0	0	0	1	0	0	0	0	0	0	0	0	0	0	0	0	0	0	0	0	0	0	0	0	0	0	0	0	0	0	0	1	0	0	0	0	0	0	0	0	0	0	0	0	0	0	0	0	0	0	0	0	0	0	0	0	0	0	0	1	40.745	24.018
1774	1995/54	1	0	0	0	0	1	0	0	0	0	0	0	1	1	0	0	0	0	1	0	0	0	0	0	0	0	0	0	0	1	0	0	0	1	0	0	0	0	0	0	0	0	0	0	0	0	0	0	0	0	0	0	0	0	0	0	0	0	0	0	0	2	41.017	24.612
1775	1995/55	1	0	0	0	0	1	0	0	0	0	0	0	1	1	0	0	0	0	0	0	0	0	0	0	0	0	0	0	0	1	0	0	0	1	0	0	0	0	0	0	0	0	0	0	0	0	0	0	0	0	0	0	0	0	0	0	0	0	0	0	0	1	41.02	24.289
1776	1995/56	0	0	0	0	0	0	0	0	0	0	0	0	1	0	0	0	0	0	0	0	0	0	0	0	0	0	0	0	0	1	0	0	0	0	0	0	0	0	0	0	1	0	0	0	0	0	0	0	0	0	0	0	0	0	0	0	0	0	0	0	0	1	40.739	24.686
1777	1995/57	1	0	0	0	0	1	0	0	0	0	0	0	0	0	0	0	0	0	0	0	0	0	0	0	0	0	0	0	0	1	0	0	0	0	0	0	0	0	0	0	1	0	0	0	0	0	0	0	0	0	0	0	0	0	0	0	0	0	0	0	0	1	40.739	24.722
1778	1995/58	0	0	0	0	0	1	0	0	0	0	0	0	0	1	0	0	0	0	1	1	0	0	0	0	0	0	0	0	0	1	0	0	0	1	0	0	0	0	0	0	0	0	0	0	0	0	0	0	0	0	0	0	0	0	0	0	0	0	0	0	0	1	41.208	23.91
1779	1995/59	0	0	0	0	0	1	0	0	0	0	0	0	1	0	0	0	0	0	0	0	0	0	0	0	0	0	0	0	0	0	0	0	0	0	0	0	0	0	0	0	0	0	0	0	0	0	0	0	0	0	0	0	0	0	0	0	0	0	0	0	0	1	41.288	23.973
1780	1995/60	1	0	0	0	0	1	0	0	0	0	0	0	0	1	0	0	0	0	0	0	0	0	0	0	0	0	0	0	0	1	0	0	0	1	0	0	0	0	0	0	0	0	0	0	0	0	0	0	0	0	0	0	0	0	0	0	0	0	0	0	0	1	41.06	23.91
1781	1995/61	1	0	0	0	0	1	0	0	0	0	0	0	0	1	0	0	0	0	0	0	0	0	0	0	0	0	0	0	0	0	0	0	0	1	0	0	0	0	0	0	0	0	0	0	0	0	0	0	0	0	0	0	0	0	0	0	0	0	0	0	0	1	41.208	23.973
1783	1995/63	0	0	0	0	0	1	0	0	0	0	0	0	1	1	1	0	0	0	0	0	0	0	0	0	0	0	0	0	0	1	0	0	0	0	0	1	0	0	0	0	1	0	0	0	0	0	0	0	0	0	0	0	0	0	0	0	0	0	0	0	0	1	43.04	23.412
1785	1996/02	0	0	0	0	0	0	0	0	0	0	0	0	0	0	0	0	0	0	1	0	0	0	0	0	0	0	0	0	0	0	0	0	0	0	0	0	0	0	1	0	0	0	0	0	0	0	0	0	0	0	0	0	0	0	0	0	0	0	0	0	0	1	44.14	19.93
1788	1997/18	0	0	0	0	0	0	0	0	0	0	0	0	0	0	0	0	0	0	0	0	0	0	0	0	0	0	0	0	0	0	0	0	0	0	0	0	0	0	0	0	0	0	0	1	0	0	0	0	0	0	0	0	0	0	0	0	0	0	0	0	0	1	44.29	15.45
1791	1997/21	1	0	0	0	0	0	0	0	0	0	0	0	1	0	0	0	0	0	1	0	0	0	0	0	0	0	0	1	0	0	0	0	0	0	0	0	0	0	0	0	0	1	0	0	0	0	0	0	0	0	0	0	0	0	0	0	0	0	0	0	0	1	43.91	15.98
1792	1997/22	0	0	0	0	0	0	0	0	0	1	0	0	0	0	0	0	0	0	1	1	0	0	0	0	0	0	0	0	0	0	0	0	0	0	0	0	0	0	0	0	0	0	0	0	0	0	0	0	0	0	0	0	0	0	0	0	0	0	0	0	0	1	43.8	15.97
1794	1997/24	0	0	0	0	0	0	0	0	0	0	0	0	1	0	0	0	0	0	0	0	0	0	0	0	0	0	0	0	0	0	0	0	0	0	0	0	0	0	0	0	0	1	0	0	0	0	0	0	0	0	0	0	0	0	0	0	0	0	0	0	0	1	42.94	17.1
1795	1997/25	0	0	0	0	0	0	0	0	0	0	0	0	1	0	0	0	0	0	0	0	0	0	0	0	0	0	0	0	0	0	0	0	0	0	0	0	0	0	0	0	0	1	0	0	0	0	0	0	0	0	0	0	0	0	0	0	0	0	0	0	0	1	42.93	17.01
1796	1997/26	1	0	0	0	0	0	0	0	0	0	0	0	1	0	0	0	0	0	1	0	0	0	0	0	0	0	0	0	0	0	1	0	0	0	0	0	0	0	0	0	0	0	0	0	0	0	0	0	0	0	0	0	0	0	0	0	0	0	0	0	0	1	42.99	17.17
1797	1997/27	1	0	0	0	0	0	0	0	0	0	0	0	1	0	0	0	0	0	1	0	0	0	0	0	0	0	0	0	0	0	0	0	0	0	0	0	0	0	0	0	0	0	0	0	0	0	0	0	0	0	0	0	0	0	0	0	0	0	0	0	0	1	42.99	17.06
1798	1997/28	0	0	0	1	0	0	0	0	0	0	0	0	0	0	0	0	0	0	1	0	0	0	0	0	0	0	0	0	0	0	1	0	0	0	0	0	0	0	0	0	0	0	0	0	0	0	0	0	0	0	0	0	0	0	0	0	0	0	0	0	0	1	42.96	17.29
1799	1997/29	0	0	0	0	0	0	0	0	0	0	0	0	0	0	0	0	0	0	0	0	0	0	0	0	0	0	0	0	1	0	1	0	0	0	0	0	0	0	0	0	0	0	0	0	0	0	0	0	0	0	0	0	0	0	0	0	0	0	0	0	0	1	42.924167	17.407
1800	1997/30	0	0	0	0	0	0	0	0	0	0	0	0	0	0	0	0	0	0	0	0	0	0	0	0	0	0	0	0	0	0	0	0	0	0	0	0	0	0	0	0	0	0	0	0	0	0	0	0	0	0	0	0	0	0	0	0	0	0	0	0	0	1	42.84	17.63
1801	1997/31	0	0	0	0	0	0	0	0	0	0	0	0	1	0	1	0	0	0	0	1	0	0	0	0	0	0	0	1	0	0	1	0	0	0	0	0	1	0	0	0	0	0	1	0	0	0	0	0	0	0	0	0	0	0	0	0	0	0	0	0	0	1	42.8456	17.704
1803	1997/33	1	0	0	1	0	0	0	0	0	0	0	0	1	0	0	0	0	0	1	0	0	0	0	0	0	0	0	0	0	0	0	0	0	0	0	0	0	0	0	0	0	1	0	0	0	0	0	0	0	0	0	0	0	0	0	0	0	0	0	0	0	1	43.16	17.24
1804	1997/34	1	0	0	0	0	0	0	0	0	0	0	0	1	0	0	0	0	0	1	0	0	0	0	0	0	0	0	0	0	0	0	0	0	0	0	0	0	0	0	0	0	1	0	0	0	0	0	0	0	0	0	0	0	0	0	0	0	0	0	0	0	1	43.19	17.17
1805	1997/35	0	0	0	0	0	0	0	0	0	0	0	0	1	0	0	0	0	0	0	0	0	0	0	0	0	0	0	0	0	0	0	0	0	0	0	0	0	0	0	0	0	1	0	0	0	0	0	0	0	0	0	0	0	0	0	0	0	0	0	0	0	1	43.22	17.12
1806	1997/36	1	0	0	1	0	0	0	0	0	0	0	0	0	0	0	0	0	0	1	0	0	0	0	0	0	0	0	1	0	0	0	0	0	0	0	0	0	0	0	0	0	1	0	0	0	0	0	0	0	0	0	0	0	0	0	0	0	0	0	0	0	1	43.32	17
1808	1997/38	1	0	0	0	0	0	0	0	0	1	0	0	1	0	0	0	0	0	1	0	0	0	0	0	0	0	0	0	0	1	0	0	0	0	0	1	0	0	0	0	0	0	0	0	0	0	0	0	0	0	0	0	0	0	0	0	0	0	0	0	0	1	44.87	15.6
1809	1997/40	1	0	0	0	0	0	0	0	0	0	0	0	0	0	0	0	0	1	0	0	0	0	0	1	0	0	0	0	0	1	0	0	0	0	0	1	0	0	1	0	1	0	0	0	0	0	0	0	0	0	0	0	0	0	0	0	0	0	0	0	0	3	44.88	21.73
1810	1997/41	0	0	0	0	0	0	0	0	0	0	0	0	0	0	0	0	0	0	1	0	0	0	0	0	0	0	0	0	0	1	0	0	0	0	0	0	0	0	1	0	0	0	0	0	0	0	0	0	0	0	0	0	0	0	0	0	0	0	0	0	0	3	45.03	21.86
1811	1997/42	0	0	0	0	0	0	0	0	0	0	0	0	0	0	0	0	0	0	1	0	0	0	1	0	0	0	0	0	0	0	0	0	0	0	0	0	0	0	0	0	0	0	0	0	0	0	0	0	0	0	0	0	0	0	0	0	0	0	0	0	0	1	44.88	22.42
1812	1997/43	1	0	0	0	0	0	0	0	0	0	0	0	1	0	0	0	0	0	1	0	0	0	1	1	0	0	0	0	0	1	0	0	0	0	0	0	0	0	0	0	1	0	0	0	0	0	0	0	0	0	0	0	0	0	0	0	0	0	0	0	0	1	44.88	22.45
1813	1997/44	1	0	0	0	0	0	0	0	0	0	0	0	0	0	0	0	0	0	1	0	0	0	0	1	0	0	0	0	0	0	0	0	0	0	0	1	0	0	0	0	0	0	0	0	0	0	0	0	0	0	0	0	0	0	0	0	0	0	0	0	0	1	44.96	22.48
1814	1997/45	1	0	0	0	0	1	0	0	0	0	0	0	0	0	0	0	0	0	0	0	0	0	0	1	0	0	0	0	0	0	0	0	0	0	0	0	0	0	1	0	0	0	0	0	0	0	0	0	0	0	0	0	0	0	0	0	0	0	0	0	0	1	44.71	22.5
1815	1997/46	1	0	0	0	0	1	0	0	0	0	0	0	0	0	0	0	0	0	1	0	0	0	0	0	0	0	0	0	0	0	0	0	0	0	0	0	0	0	0	0	1	0	0	0	0	0	0	0	0	0	0	0	0	0	0	0	0	0	0	0	0	1	43.78	22.31
1816	1997/47	0	0	0	0	0	1	0	0	0	0	0	0	0	1	0	0	0	0	1	0	0	0	0	0	0	0	0	0	0	0	0	0	0	1	0	0	0	0	0	0	1	0	0	0	0	0	0	0	0	0	0	0	0	0	0	0	0	0	0	0	0	1	43.5395	22.2888
1817	1997/48	1	0	0	0	0	1	0	0	0	0	0	0	1	1	0	0	0	0	1	0	0	0	0	0	0	0	0	0	0	1	0	0	0	0	0	0	0	0	0	0	1	0	0	0	0	0	0	0	0	0	0	0	0	0	0	0	0	0	0	0	0	3	41.954	24.844
1818	1997/49	0	0	0	0	0	1	0	0	0	0	0	0	0	0	0	0	0	0	0	0	0	0	0	0	0	0	0	0	0	1	0	0	0	0	0	0	0	0	0	0	0	0	0	0	0	0	0	0	0	0	0	0	0	0	0	0	0	0	0	0	0	2	41.888	24.661
1819	1997/50	0	0	0	0	0	0	0	0	0	1	0	0	0	1	0	1	0	0	1	0	0	0	0	0	0	0	0	0	0	1	0	0	0	0	0	0	0	0	0	0	1	0	0	0	0	0	0	0	0	0	0	0	0	0	0	0	0	0	0	0	0	3	41.795	24.607
1820	1997/51	0	0	0	0	0	0	0	0	0	0	0	0	1	0	0	1	0	0	0	0	0	0	1	0	0	0	0	0	0	1	0	0	0	0	0	0	0	0	0	0	1	0	0	0	0	0	0	0	0	0	0	0	0	0	0	0	0	0	0	0	0	1	41.802	24.57
1821	1997/52	0	0	0	0	0	1	0	0	0	0	0	0	1	1	0	0	0	0	0	0	0	0	0	0	0	0	0	0	0	1	0	0	0	0	0	0	0	0	0	0	0	0	0	0	0	0	0	0	0	0	0	0	0	0	0	0	0	0	0	0	0	1	42.007	24.89
1822	1997/53	0	0	0	0	0	0	0	0	0	0	0	0	0	0	0	0	0	0	0	0	0	0	0	0	0	0	0	0	0	1	0	0	0	0	0	0	0	0	0	0	0	0	0	0	0	0	0	0	0	0	0	0	0	0	0	0	0	0	0	0	0	1	42.677	26.325
1828	1997/59	0	0	0	0	0	1	0	0	0	1	0	0	0	0	0	0	0	0	0	1	0	0	0	0	0	1	0	0	0	1	0	0	0	0	0	0	0	0	0	0	0	0	0	0	0	0	0	0	0	0	0	0	0	0	0	0	0	0	0	0	0	1	42.03	27.624
1832	1997/63	0	0	0	0	0	1	0	0	0	0	0	0	0	0	0	0	0	0	0	0	0	0	0	0	0	0	0	0	0	1	0	0	0	0	0	0	0	0	0	0	0	0	0	0	0	0	0	0	0	0	0	0	0	0	0	0	0	0	0	0	0	1	42.026	27.477
1833	1997/64	0	0	0	0	0	0	0	0	0	0	0	0	0	0	0	0	0	1	1	0	0	0	0	1	0	0	0	0	0	1	0	0	1	0	0	0	0	0	0	0	0	0	0	0	0	0	0	0	0	0	0	0	0	0	0	0	0	0	0	0	0	1	45.18	23.82
1834	1997/65	0	0	0	0	0	0	0	0	0	0	0	0	1	0	0	0	0	0	1	0	0	0	0	1	0	0	0	0	0	0	0	0	1	0	0	0	0	0	0	0	0	0	0	0	0	0	0	0	0	0	0	0	0	0	0	0	0	0	0	0	0	1	45.14	23.14
1835	1997/70	1	0	0	0	0	0	0	0	0	0	0	0	0	0	0	0	0	0	1	0	0	0	0	0	0	0	0	0	0	0	0	0	0	0	0	1	0	0	0	0	0	1	0	0	0	0	0	0	0	0	0	0	0	0	0	0	0	0	0	0	0	1	45.24	13.93
1836	1997/71	1	0	0	0	0	0	0	0	0	0	0	0	0	0	0	0	0	0	1	0	0	0	1	0	0	0	0	1	0	0	0	0	0	0	0	0	0	0	0	0	0	1	0	0	0	0	0	0	0	0	0	0	0	0	0	0	0	0	0	0	0	1	45.14	13.73
1839	1997/74	0	0	0	0	0	0	0	0	0	0	0	0	0	0	0	0	0	0	0	0	0	0	0	0	0	0	0	0	0	0	0	0	0	0	0	0	0	0	0	0	0	0	0	0	0	0	0	0	0	0	0	0	0	0	0	0	0	0	0	0	0	1	45.09	14.13
1840	1997/75	0	0	0	0	0	0	0	0	0	0	0	0	0	0	0	0	0	0	0	0	0	0	0	0	0	0	0	0	0	0	0	0	0	0	0	0	0	0	0	0	0	0	0	0	0	0	0	0	0	0	0	0	0	0	0	0	0	0	0	0	0	1	45.14	14.19
1842	1997/77	1	0	0	0	0	0	0	0	0	0	0	0	1	0	0	0	0	0	1	0	0	0	1	0	0	0	0	0	0	1	0	0	0	0	0	0	0	0	0	0	0	0	0	0	0	0	0	0	0	0	0	0	0	0	0	0	0	0	0	0	0	1	45.4	13.97
1843	1997/78	1	0	0	0	0	0	0	0	0	0	0	0	0	0	0	1	0	0	1	0	0	0	0	0	0	0	0	0	0	0	0	0	0	0	0	0	0	0	0	0	0	0	0	0	0	0	0	0	0	0	0	0	0	0	0	0	0	0	0	0	0	1	45.65	14.02
1845	1998/03	0	0	0	0	0	0	0	0	0	0	0	0	0	0	0	0	0	0	0	0	0	0	0	0	0	0	0	0	0	1	0	0	0	0	0	0	0	0	0	0	0	0	0	0	0	0	0	0	0	0	0	0	0	0	0	0	0	0	0	0	0	1	45.55	24.26
1847	1998/05	0	0	0	0	0	0	0	0	0	0	0	0	0	0	0	0	0	0	0	0	0	0	0	0	0	0	0	0	0	1	0	0	0	0	0	0	0	0	0	0	0	0	0	0	0	0	0	0	0	0	0	0	0	0	0	0	0	0	0	0	0	1	45.4	25.15
1848	1998/06	0	0	0	0	0	0	0	0	0	0	0	0	0	0	0	0	0	0	0	0	0	0	1	0	0	0	0	0	0	1	0	0	1	0	0	0	0	0	0	0	0	0	0	0	0	0	0	0	0	0	0	0	0	0	0	0	0	0	0	0	0	1	45.42	25.21
1849	1998/07	0	0	0	0	0	0	0	0	0	0	0	0	0	0	0	0	0	0	1	0	0	0	0	0	0	0	0	0	0	1	0	0	1	0	0	0	0	0	0	0	0	0	0	0	0	0	0	0	0	0	0	0	0	0	0	0	0	0	0	0	0	1	45.44	25.23
1850	1998/08	0	0	0	0	0	0	0	0	0	0	0	0	0	0	0	0	0	0	0	0	0	0	0	0	0	0	0	0	0	0	0	0	1	0	0	0	0	0	0	0	0	0	0	0	0	0	0	0	0	0	0	0	0	0	0	0	0	0	0	0	0	1	45.51	25.36
1851	1998/09	0	0	0	0	0	0	0	0	0	0	0	0	0	0	0	0	0	0	1	0	0	0	0	0	0	0	0	0	0	1	0	0	1	0	0	0	0	0	0	0	1	0	0	0	0	0	0	0	0	0	0	0	0	0	0	0	0	0	0	0	0	1	45.57	25.26
1852	1998/10	0	0	0	0	0	0	0	0	0	0	0	0	0	0	0	0	0	0	0	0	0	0	0	0	0	0	0	0	0	1	0	0	1	0	0	0	0	0	0	0	0	0	0	0	0	0	0	0	0	0	0	0	0	0	0	0	0	0	0	0	0	1	45.56	25.28
1853	1998/11	0	0	0	0	0	0	0	0	0	1	0	0	0	0	0	0	0	0	1	0	0	0	1	0	0	0	0	0	0	1	0	0	1	0	0	0	0	0	0	0	0	0	0	0	0	0	0	0	0	0	0	0	0	0	0	0	0	0	0	0	0	1	45.51	25.5
1854	1998/12	0	0	0	0	0	0	0	0	0	0	0	0	0	0	0	0	0	0	0	0	0	0	0	0	0	0	0	0	0	1	0	0	0	0	0	0	0	0	0	0	0	0	0	0	0	0	0	0	0	0	0	0	0	0	0	0	0	0	0	0	0	1	45.56	25.6
1855	1998/13	0	0	0	0	0	0	0	0	0	1	0	0	0	0	0	0	0	0	1	0	0	0	1	0	0	0	0	0	0	1	0	0	1	0	0	0	0	0	0	0	0	0	0	0	0	0	0	0	0	0	0	0	0	0	0	0	0	0	0	0	0	1	45.51	25.89
1856	1998/14	0	0	0	0	0	0	0	0	0	0	0	0	0	0	0	0	0	0	0	0	0	0	0	0	0	0	0	0	0	1	0	0	0	0	0	0	0	0	0	0	0	0	0	0	0	0	0	0	0	0	0	0	0	0	0	0	0	0	0	0	0	1	45.51	25.9
1857	1998/15	0	0	0	0	0	1	0	0	0	0	0	0	0	0	0	0	0	0	1	0	0	0	0	0	0	0	0	0	0	1	0	0	1	0	0	1	0	0	0	0	0	0	0	0	0	0	0	0	0	0	0	0	0	0	0	0	0	0	0	0	0	1	45.49	25.93
1858	1998/16	0	0	0	0	0	0	0	0	0	0	0	0	0	0	0	0	0	0	0	0	0	0	0	0	0	0	0	0	0	0	0	0	1	0	0	0	0	0	0	0	0	0	0	0	0	0	0	0	0	0	0	0	0	0	0	0	0	0	0	0	0	1	45.49	25.94
1859	1998/17	0	0	0	0	0	0	0	0	0	0	0	0	0	0	0	0	0	0	0	0	0	0	0	0	0	0	0	0	0	0	0	0	1	0	0	0	0	0	0	0	0	0	0	0	0	0	0	0	0	0	0	0	0	0	0	0	0	0	0	0	0	1	45.51	25.93
1860	1998/18	0	0	0	0	0	0	0	0	0	0	0	0	0	0	0	0	0	0	0	0	0	0	0	0	0	0	0	0	0	0	0	0	1	0	0	0	0	0	0	0	0	0	0	0	0	0	0	0	0	0	0	0	0	0	0	0	0	0	0	0	0	1	45.4926	25.9767
1861	1998/19	0	0	0	0	0	0	0	0	0	0	0	0	0	0	0	0	0	0	0	0	0	0	0	0	0	0	0	0	0	0	0	0	1	0	0	0	0	0	0	0	0	0	0	0	0	0	0	0	0	0	0	0	0	0	0	0	0	0	0	0	0	1	45.48	25.97
1862	1998/20	0	0	0	0	0	0	0	0	0	0	0	0	0	0	0	0	0	0	0	0	0	0	0	0	0	0	0	0	0	0	0	0	0	0	0	0	0	0	0	0	0	0	0	0	0	0	0	0	0	0	0	0	0	0	0	0	0	0	0	0	0	1	45.4888	25.9732
1863	1998/21	0	0	0	0	0	0	0	0	0	0	0	0	0	0	0	0	0	0	0	0	0	0	0	0	0	0	0	0	0	0	0	0	0	0	0	0	0	0	0	0	0	0	0	0	0	0	0	0	0	0	0	0	0	0	0	0	0	0	0	0	0	1	45.4945	25.9717
1864	1998/22	0	0	0	0	0	0	0	0	0	0	0	0	0	0	0	0	0	0	0	0	0	0	0	0	0	0	0	0	0	0	0	0	1	0	0	0	0	0	0	0	0	0	0	0	0	0	0	0	0	0	0	0	0	0	0	0	0	0	0	0	0	1	45.5	25.95
1865	1998/23	0	0	0	0	0	0	0	0	0	0	0	0	0	0	0	0	0	0	0	0	0	0	0	0	0	0	0	0	0	1	0	0	0	0	0	0	0	0	0	0	0	0	0	0	0	0	0	0	0	0	0	0	0	0	0	0	0	0	0	0	0	1	45.482	25.93
1866	1998/24	0	0	0	0	0	0	0	0	0	0	0	0	0	0	0	0	0	0	0	0	0	0	0	0	0	0	0	0	0	0	0	0	1	0	0	0	0	0	0	0	0	0	0	0	0	0	0	0	0	0	0	0	0	0	0	0	0	0	0	0	0	1	45.5	25.9
1867	1998/25	0	0	0	0	0	0	0	0	0	0	0	0	0	0	0	0	0	0	0	0	0	0	0	0	0	0	0	0	0	1	0	0	1	0	0	0	0	0	0	0	0	0	0	0	0	0	0	0	0	0	0	0	0	0	0	0	0	0	0	0	0	1	45.488	25.892
1868	1998/26	0	0	0	0	0	0	0	0	0	0	0	0	0	0	0	0	0	0	0	0	0	0	0	0	0	0	0	0	0	1	0	0	0	0	0	0	0	0	0	0	0	0	0	0	0	0	0	0	0	0	0	0	0	0	0	0	0	0	0	0	0	1	45.488	25.877
1869	1998/27	1	0	0	0	0	0	0	0	0	0	0	0	0	0	0	0	0	0	0	0	0	0	0	0	0	0	0	0	0	0	0	0	0	0	0	0	0	0	0	0	0	0	0	0	0	0	0	0	0	0	0	0	0	0	0	0	0	0	0	0	0	1	44.38	16.09
1871	1998/29	1	0	0	0	0	0	0	0	0	0	0	0	1	0	0	0	0	0	1	1	0	0	1	0	0	1	0	1	0	0	0	0	0	0	0	0	0	0	0	0	0	0	0	0	0	0	0	0	0	0	0	0	0	0	0	0	0	0	0	0	0	1	43.85	16.16
1872	1998/30	1	0	0	1	0	0	0	0	0	0	0	0	1	0	0	0	0	0	0	0	0	0	0	0	0	0	0	1	0	0	0	0	0	0	0	0	0	0	0	0	0	0	0	0	0	0	0	0	0	0	0	0	0	0	0	0	0	0	0	0	0	1	43.74	15.9
1873	1998/31	1	0	0	0	0	0	0	0	0	0	0	0	0	0	1	0	0	0	1	0	0	0	0	0	0	0	0	1	0	0	0	0	0	0	0	0	0	0	0	0	0	0	0	0	0	0	0	0	0	0	0	0	0	0	0	0	0	0	0	0	0	1	43.72	16.22
1874	1998/32	1	0	0	0	0	0	0	0	0	0	0	0	0	0	0	0	0	0	0	0	0	0	0	0	0	0	0	0	0	0	0	0	0	0	0	0	0	0	0	0	0	0	0	0	0	0	0	0	0	0	0	0	0	0	0	0	0	0	0	0	0	1	43.59	16.5
1875	1998/33	1	0	0	0	0	0	0	0	0	0	0	0	0	0	0	0	0	0	1	0	0	0	0	0	0	0	0	0	0	0	0	0	0	0	0	0	0	0	0	0	0	0	0	0	0	0	0	0	0	0	0	0	0	0	0	0	0	0	0	0	0	1	43.69	16.54
1876	1998/34	1	0	0	0	0	0	0	0	0	0	0	0	1	0	0	0	0	0	0	0	0	0	0	0	0	0	0	1	0	0	0	0	0	0	0	0	0	0	0	0	0	0	0	0	0	0	0	0	0	0	0	0	0	0	0	0	0	0	0	0	0	1	43.71	16.57
1877	1998/35	1	0	0	0	0	0	0	0	0	0	0	0	1	0	0	0	0	0	1	0	0	0	0	0	0	0	0	0	0	0	0	0	0	0	0	0	0	0	0	0	0	1	0	0	0	0	0	0	0	0	0	0	0	0	0	0	0	0	0	0	0	1	43.9138	16.396
1878	1998/36	0	0	0	0	0	0	0	0	0	0	0	0	0	0	0	0	0	0	1	0	0	0	0	0	0	0	0	0	0	0	0	0	0	0	0	0	0	0	0	0	0	1	0	0	0	0	0	0	0	0	0	0	0	0	0	0	0	0	0	0	0	1	43.4	16.88
1880	1998/38	0	0	0	0	0	0	0	0	0	0	0	0	1	0	0	0	0	0	0	0	0	0	0	0	0	0	0	0	0	0	1	0	0	0	0	0	0	0	0	0	0	0	0	0	0	0	0	0	0	0	0	0	0	0	0	0	0	0	0	0	0	1	43.11	17.35
1881	1998/39	1	0	0	0	0	0	0	0	0	0	0	0	0	0	0	0	0	0	1	0	0	0	0	0	0	0	0	1	0	0	1	0	0	0	0	0	0	0	0	0	0	0	0	1	0	0	0	0	0	0	0	0	0	0	0	0	0	0	0	0	0	1	42.57	18.29
1882	1998/40	1	0	0	0	0	0	0	0	0	0	0	0	0	0	0	0	0	0	1	0	0	0	0	0	0	0	0	0	0	0	1	0	0	0	0	0	0	0	0	0	0	0	0	0	0	0	0	0	0	0	0	0	0	0	0	0	0	0	0	0	0	1	42.56	18.33
1883	1998/41	1	0	0	1	0	0	0	0	0	0	0	0	1	0	0	0	0	0	1	0	0	0	0	0	0	0	0	1	0	0	1	0	0	0	0	0	0	0	0	0	0	0	0	0	0	0	0	0	0	0	0	0	0	0	0	0	0	0	0	0	0	1	42.6	18.23
1884	1998/42	0	0	0	0	0	0	0	0	0	0	0	0	0	0	0	0	0	0	0	0	1	0	0	0	0	0	0	0	0	0	1	0	0	0	0	0	0	0	0	0	0	0	0	0	0	0	0	0	0	0	0	0	0	0	0	0	0	0	0	0	0	1	42.59	18.28
1885	1998/43	0	0	0	0	0	0	0	0	0	0	0	0	0	0	1	0	0	0	0	0	0	0	0	0	0	0	0	0	0	0	1	0	0	0	0	0	0	0	0	0	0	0	0	0	0	0	0	0	0	0	0	0	0	0	0	0	0	0	0	0	0	1	42.6	18.07
1886	1998/44	1	0	0	1	0	0	0	0	0	0	0	0	1	0	1	0	0	0	0	0	0	0	0	0	0	0	0	1	1	0	1	0	0	0	0	0	1	0	0	0	0	0	1	0	0	0	0	0	0	0	0	0	0	0	0	0	0	0	0	0	0	1	42.8402	17.6956
1888	1998/46	1	0	0	0	0	0	0	0	0	0	0	0	0	0	0	0	0	0	0	0	0	0	0	0	0	0	0	0	0	0	0	0	0	0	0	0	0	0	0	0	0	1	0	0	0	0	0	0	0	0	0	0	0	0	0	0	0	0	0	0	0	1	43.41	16.9
1890	1998/48	0	0	0	0	0	0	0	0	0	0	0	0	0	0	0	0	0	0	0	0	0	0	0	0	0	0	0	0	0	0	0	0	0	0	0	0	0	0	0	0	0	1	0	0	0	0	0	0	0	0	0	0	0	0	0	0	0	0	0	0	0	1	43.48	16.84
1891	1998/49	1	0	0	0	0	0	0	0	0	0	0	0	1	0	0	0	0	0	0	0	0	0	0	0	0	0	0	1	0	0	0	0	0	0	0	0	0	0	0	0	0	1	0	0	0	0	0	0	0	0	0	0	0	0	0	0	0	0	0	0	0	1	43.44	16.9
1892	1998/50	1	0	0	0	0	0	0	0	0	0	0	0	1	0	0	0	0	0	1	0	0	0	0	0	0	0	0	0	0	0	0	0	0	0	0	0	0	0	0	0	0	1	0	0	0	0	0	0	0	0	0	0	0	0	0	0	0	0	0	0	0	1	43.34	17.11
1893	1998/51	0	0	0	0	0	0	0	0	0	0	0	0	0	0	1	0	0	0	1	0	0	0	0	0	0	0	0	1	0	0	0	0	0	0	0	0	0	0	0	0	0	0	0	0	0	0	0	0	0	0	0	0	0	0	0	0	0	0	0	0	0	1	43.4545	17.2
1895	1999/01	1	0	0	0	0	0	0	0	0	1	0	0	0	0	1	1	0	0	0	0	1	0	0	0	0	0	0	0	0	1	0	0	0	0	0	1	0	0	0	0	0	0	0	0	0	0	0	0	0	0	0	0	0	0	0	0	0	0	0	0	0	3	45.22	15.54
1897	1999/03	1	0	0	0	0	1	0	0	0	0	0	0	0	0	0	1	0	0	1	0	0	0	0	0	0	0	0	0	0	1	0	0	0	0	0	0	0	0	0	0	0	0	0	0	0	0	0	0	0	0	0	0	0	0	0	0	0	0	0	0	0	1	44.58	15.75
1898	1999/04	1	0	0	0	0	0	0	0	0	0	0	0	1	0	0	0	0	0	1	0	0	0	0	0	0	0	0	0	0	0	0	0	0	0	0	0	0	0	0	0	0	1	0	0	0	0	0	0	0	0	0	0	0	0	0	0	0	0	0	0	0	1	44.25	15.81
1899	1999/05	1	0	0	0	0	1	0	0	0	0	0	0	1	0	0	0	0	0	1	0	0	0	0	0	0	0	0	1	0	0	0	0	0	0	0	0	0	0	0	0	0	1	0	0	0	0	0	0	0	0	0	0	0	0	0	0	0	0	0	0	0	1	44.25	15.65
1901	1999/07	1	0	0	0	0	0	0	0	0	0	0	0	0	0	0	0	0	0	0	0	0	0	0	0	0	0	0	0	0	0	0	0	0	0	0	0	0	0	0	0	0	0	0	0	0	0	0	0	0	0	0	0	0	0	0	0	0	0	0	0	0	1	44.18	15.73
1902	1999/08	1	0	0	0	0	0	0	0	0	0	0	0	1	0	0	0	0	0	1	0	0	0	0	0	0	0	0	1	0	0	0	0	0	0	0	0	0	0	0	0	0	0	0	0	0	0	0	0	0	0	0	0	0	0	0	0	0	0	0	0	0	1	44.011	16.038
1904	1999/10	1	0	0	0	0	0	0	0	0	0	0	0	0	0	0	0	0	0	0	0	0	0	0	0	0	0	0	0	0	0	0	0	0	0	0	0	0	0	0	0	0	0	0	0	0	0	0	0	0	0	0	0	0	0	0	0	0	0	0	0	0	1	43.89	16.08
1905	1999/100	0	0	0	0	0	0	0	0	0	0	0	0	0	0	0	0	0	0	0	0	0	0	0	0	0	0	0	0	0	0	0	0	0	0	0	0	0	0	0	0	1	0	0	0	0	0	0	0	0	0	0	0	0	0	0	0	0	0	0	0	0	1	45.38	23.91
1907	1999/102	0	0	0	0	0	0	0	0	0	0	0	0	0	0	0	0	0	0	0	0	0	0	0	0	0	0	0	0	0	0	0	0	0	0	0	0	0	0	0	0	0	0	0	0	0	0	0	0	0	0	0	0	0	0	0	0	0	0	0	0	0	1	45.41135	23.913183
1908	1999/103	1	0	0	0	0	0	0	0	0	1	0	0	0	0	0	1	0	0	1	0	0	0	1	1	0	1	0	0	0	1	0	0	1	0	0	1	0	0	0	0	0	0	0	0	0	0	0	0	0	0	0	0	0	0	0	0	0	0	0	0	0	1	46.467367	22.842183
1910	1999/12	1	0	0	0	0	0	0	0	0	0	0	0	1	0	0	0	0	0	1	0	0	0	0	0	0	0	0	1	0	0	0	0	0	0	0	0	0	0	0	0	0	0	0	0	0	0	0	0	0	0	0	0	0	0	0	0	0	0	0	0	0	1	44.0335	16.1914
1912	1999/13	1	0	0	0	0	0	0	0	0	0	0	0	1	0	0	0	0	0	0	0	0	0	0	0	0	0	0	0	0	0	0	0	0	0	0	0	0	0	0	0	0	0	0	0	0	0	0	0	0	0	0	0	0	0	0	0	0	0	0	0	0	1	43.92	16.46
1914	1999/15	1	0	0	0	0	1	0	0	0	0	0	0	1	0	0	0	0	0	0	0	0	0	0	0	0	0	0	1	0	0	0	0	0	0	0	0	0	0	0	0	0	0	0	0	0	0	0	0	0	0	0	0	0	0	0	0	0	0	0	0	0	1	43.76	16.63
1916	1999/17	1	0	0	0	0	0	0	0	0	0	0	0	0	0	0	0	0	0	0	0	0	0	0	0	0	0	0	0	0	0	0	0	0	0	0	0	0	0	0	0	0	0	0	0	0	0	0	0	0	0	0	0	0	0	0	0	0	0	0	0	0	1	43.71	16.58
1917	1999/18	1	0	0	0	0	0	0	0	0	0	0	0	0	0	0	0	0	0	1	0	0	0	0	0	0	0	0	0	0	0	0	0	0	0	0	0	0	0	0	0	0	0	0	0	0	0	0	0	0	0	0	0	0	0	0	0	0	0	0	0	0	1	43.68	16.52
1918	1999/19	0	0	0	0	0	0	0	0	0	0	0	0	0	0	0	0	0	0	0	0	0	0	0	0	0	0	0	0	0	0	0	0	0	0	0	0	0	0	0	0	0	0	0	0	0	0	0	0	0	0	0	0	0	0	0	0	0	0	0	0	0	1	43.69	16.49
1919	1999/20	1	0	0	0	0	1	0	0	0	0	0	0	0	0	0	0	0	0	1	0	0	0	0	0	0	0	0	0	0	0	0	0	0	0	0	0	0	0	0	0	0	0	0	0	0	0	0	0	0	0	0	0	0	0	0	0	0	0	0	0	0	1	43.58	16.6
1922	1999/23	1	0	0	1	0	0	0	0	0	0	0	0	1	0	0	0	0	0	1	0	0	0	0	0	0	0	0	0	0	0	0	0	0	0	0	0	1	0	0	0	0	1	0	0	0	0	0	0	0	0	0	0	0	0	0	0	0	0	0	0	0	1	43.458	16.763
1923	1999/24	0	0	0	0	0	0	0	0	0	0	0	0	1	0	0	0	0	0	0	0	0	0	0	0	0	0	0	0	0	0	0	0	0	0	0	0	0	0	0	0	0	0	0	0	0	0	0	0	0	0	0	0	0	0	0	0	0	0	0	0	0	1	43.44	16.94
1926	1999/27	1	0	0	0	0	0	0	0	0	0	0	0	1	0	0	0	0	0	1	1	0	0	0	0	0	0	0	0	0	0	1	0	0	0	0	0	0	0	0	0	0	0	0	0	0	0	0	0	0	0	0	0	0	0	0	0	0	0	0	0	0	1	43.08	17.36
1927	1999/28	1	0	0	0	0	0	0	0	0	0	0	0	0	0	0	0	0	0	0	0	0	0	0	0	0	0	0	0	0	0	1	0	0	0	0	0	0	0	0	0	0	0	0	0	0	0	0	0	0	0	0	0	0	0	0	0	0	0	0	0	0	1	43.04	17.43
1928	1999/29	1	0	0	0	0	0	0	0	0	0	0	0	1	0	0	0	0	0	1	0	0	0	0	0	0	0	0	0	0	0	1	0	0	0	0	0	0	0	0	0	0	0	0	0	0	0	0	0	0	0	0	0	0	0	0	0	0	0	0	0	0	1	43.14	17.73
1929	1999/30	0	0	0	0	0	0	0	0	0	0	0	0	0	0	0	0	0	0	0	0	0	0	0	0	0	0	0	0	0	0	1	0	0	0	0	0	0	0	0	0	0	0	0	0	0	0	0	0	0	0	0	0	0	0	0	0	0	0	0	0	0	1	43.26	17.9
1930	1999/31	1	0	0	0	0	0	0	0	0	1	0	0	1	0	1	0	0	0	1	0	0	0	0	0	0	1	0	1	0	0	0	0	0	0	0	0	0	0	0	0	0	0	0	0	0	0	0	0	0	0	0	0	0	0	0	0	0	0	0	0	0	1	43.44	17.93
1931	1999/32	1	0	0	1	0	0	0	0	0	0	0	0	1	0	0	0	0	0	1	0	0	0	0	0	0	0	0	0	0	0	0	0	0	0	0	0	0	0	0	0	0	0	0	0	0	0	0	0	0	0	0	0	0	0	0	0	0	0	0	0	0	1	43.42	17.87
1932	1999/33	1	0	0	0	0	0	0	0	0	0	0	0	1	0	0	0	0	0	1	0	0	0	0	0	0	0	0	0	0	0	1	0	0	0	0	0	0	0	0	0	0	0	0	0	0	0	0	0	0	0	0	0	0	0	0	0	0	0	0	0	0	1	43.28	17.87
1933	1999/34	0	0	0	0	0	0	0	0	0	0	0	0	1	0	0	0	0	0	0	0	0	0	0	0	0	0	0	0	0	0	1	0	0	0	0	0	0	0	0	0	0	0	0	0	0	0	0	0	0	0	0	0	0	0	0	0	0	0	0	0	0	1	42.98	17.54
1936	1999/37	1	0	0	0	0	0	0	0	0	0	0	0	1	0	0	0	0	0	1	0	0	0	0	0	0	0	0	0	0	0	1	0	0	0	0	0	0	0	0	0	0	0	0	0	0	0	0	0	0	0	0	0	0	0	0	0	0	0	0	0	0	1	42.92	17.42
1937	1999/38	0	0	0	0	0	0	0	0	0	0	0	0	0	0	0	0	0	0	1	0	0	0	0	0	0	0	0	0	0	0	1	0	0	0	0	0	0	0	0	0	0	0	0	0	0	0	0	0	0	0	0	0	0	0	0	0	0	0	0	0	0	1	42.7	18.04
1938	1999/39	0	0	0	0	0	0	0	0	0	0	0	0	0	0	0	0	0	0	0	0	0	0	0	0	0	0	0	0	0	0	1	0	0	0	0	0	0	0	0	0	0	0	0	0	0	0	0	0	0	0	0	0	0	0	0	0	0	0	0	0	0	1	42.62	18.12
1939	1999/40	0	0	0	0	0	0	0	0	0	0	0	0	1	0	1	0	0	0	1	0	0	0	0	0	0	0	0	0	0	0	1	0	0	0	0	0	0	0	0	0	0	0	0	0	0	0	0	0	0	0	0	0	0	0	0	0	0	0	0	0	0	1	42.61	18.13
1940	1999/41	0	0	0	0	0	0	0	0	0	0	0	0	1	0	0	0	0	0	0	0	0	0	0	0	0	0	0	0	0	0	1	0	0	0	0	0	0	0	0	0	0	0	1	0	0	0	0	0	0	0	0	0	0	0	0	0	0	0	0	0	0	1	42.58	18.22
1941	1999/42	1	0	0	0	0	0	0	0	0	0	0	0	0	0	0	0	0	0	1	0	0	0	0	0	0	0	0	0	0	0	1	0	0	0	0	0	0	0	0	0	0	0	0	0	0	0	0	0	0	0	0	0	0	0	0	0	0	0	0	0	0	1	42.63	18.18
1942	1999/43	1	0	0	0	0	0	0	0	0	0	0	0	1	0	0	0	0	0	1	0	0	0	0	0	0	0	0	0	0	0	1	0	0	0	0	0	0	0	0	0	0	0	0	0	0	0	0	0	0	0	0	0	0	0	0	0	0	0	0	0	0	1	42.64	18.19
1943	1999/44	1	0	0	0	0	0	0	0	0	0	0	0	1	0	0	0	0	0	0	0	0	0	0	0	0	0	0	1	0	0	1	0	0	0	0	0	0	0	0	0	0	0	0	0	0	0	0	0	0	0	0	0	0	0	0	0	0	0	0	0	0	1	42.64	18.16
1944	1999/45	0	0	0	0	0	0	0	0	0	0	0	0	0	0	1	0	0	0	0	0	0	0	0	0	0	0	0	0	0	0	1	0	0	0	0	0	0	0	0	0	0	0	0	0	0	0	0	0	0	0	0	0	0	0	0	0	0	0	0	0	0	1	42.65	18.12
1945	1999/46	0	0	0	0	0	0	0	0	0	0	0	0	1	0	1	0	0	0	0	0	0	0	0	0	0	0	0	0	0	0	1	0	0	0	0	0	0	0	0	0	0	0	0	0	0	0	0	0	0	0	0	0	0	0	0	0	0	0	0	0	0	1	42.99	17.53
1948	1999/49	0	0	0	0	0	0	0	0	0	0	0	0	1	0	0	0	0	0	0	0	0	0	0	0	0	0	0	0	0	0	0	0	0	0	0	0	0	0	0	0	0	1	0	0	0	0	0	0	0	0	0	0	0	0	0	0	0	0	0	0	0	1	43.31	17.04
1949	1999/50	1	0	0	0	0	0	0	0	0	0	0	0	0	0	0	0	0	0	1	0	0	0	0	0	0	0	0	0	0	0	0	0	0	0	0	0	0	0	0	0	0	0	0	0	0	0	0	0	0	0	0	0	0	0	0	0	0	0	0	0	0	1	43.78	15.83
1952	1999/52	0	0	0	0	0	0	0	0	0	0	0	0	0	0	0	0	0	0	0	0	0	0	0	0	0	0	0	0	0	0	0	0	0	0	0	0	0	0	0	0	0	0	0	0	0	0	0	0	0	0	0	0	0	0	0	0	0	0	0	0	0	1	43.57	15.87
1954	1999/54	0	0	0	0	0	0	0	0	0	0	0	0	0	0	0	0	0	0	0	0	0	0	0	0	0	0	0	0	0	0	0	0	0	0	0	0	0	0	0	0	0	0	0	0	0	0	0	0	0	0	0	0	0	0	0	0	0	0	0	0	0	1	43.18	16.45
1955	1999/55	1	0	0	0	0	0	0	0	0	1	0	0	1	0	0	0	0	0	1	0	0	0	0	0	0	0	0	0	0	0	0	0	0	0	0	0	0	0	0	0	0	0	0	0	0	0	0	0	0	0	0	0	0	0	0	0	0	0	0	0	0	2	45.65	14.22
1956	1999/56	1	0	0	0	0	0	0	0	0	0	0	0	0	0	0	0	0	0	0	0	0	0	0	0	0	0	0	0	0	0	0	0	0	0	0	0	0	0	0	0	0	0	0	0	0	0	0	0	0	0	0	0	0	0	0	0	0	0	0	0	0	1	45.32	14.54
1957	1999/57	0	0	0	0	0	0	0	0	0	0	0	0	1	0	0	0	0	0	0	1	0	0	0	0	0	0	0	1	0	0	0	0	0	0	0	0	0	0	0	0	0	0	0	1	0	0	0	0	0	0	0	0	0	0	0	0	0	0	0	0	0	1	45.03	14.58
1958	1999/58	1	0	0	0	0	0	0	0	0	1	0	0	0	0	0	0	0	0	1	0	0	0	0	0	0	1	0	1	0	0	0	0	0	0	0	0	0	0	0	0	0	0	0	0	0	0	0	0	0	0	0	0	0	0	0	0	0	0	0	0	0	1	44.97	14.67
1959	1999/59	1	0	0	0	0	0	0	0	0	0	0	0	0	0	0	0	0	0	1	1	0	0	0	0	0	0	0	1	0	0	0	0	0	0	0	0	0	0	0	0	0	0	0	0	0	0	0	0	0	0	0	0	0	0	0	0	0	0	0	0	0	1	44.93	14.92
1960	1999/60	1	0	0	0	0	0	0	0	0	0	0	0	1	0	0	0	0	0	1	0	0	0	0	0	0	0	0	1	0	0	0	0	0	0	0	0	0	0	0	0	0	1	0	0	0	0	0	0	0	0	0	0	0	0	0	0	0	0	0	0	0	1	44.53	15.14
1961	1999/61	1	0	0	0	0	0	0	0	0	0	0	0	1	0	0	0	0	0	1	0	0	0	0	0	0	0	0	0	0	0	0	0	0	0	0	0	0	0	0	0	0	0	0	0	0	0	0	0	0	0	0	0	0	0	0	0	0	0	0	0	0	1	44.4	15.25
1962	1999/62	1	0	0	0	0	0	0	0	0	1	0	0	1	0	0	0	0	0	1	0	1	0	0	0	0	0	0	1	1	0	0	0	0	0	0	0	0	0	0	0	0	1	1	0	0	0	0	0	0	0	0	0	0	0	0	0	0	0	0	0	0	1	44.3	15.47
1963	1999/63	1	0	0	0	0	0	0	0	0	0	0	0	1	0	0	0	0	0	0	0	0	0	0	0	0	0	0	0	0	0	0	0	0	0	0	0	0	0	0	0	0	1	0	0	0	0	0	0	0	0	0	0	0	0	0	0	0	0	0	0	0	1	44.325	15.475
1964	1999/64	1	0	0	0	0	0	0	0	0	1	0	0	1	0	0	0	0	0	0	0	0	0	1	0	0	0	0	0	0	0	0	0	0	0	0	1	0	0	0	0	0	0	0	0	0	0	0	0	0	0	0	0	0	0	0	0	0	0	0	0	0	2	44.35	15.48
1965	1999/65	1	0	0	0	0	1	0	0	0	0	0	0	0	0	0	0	0	0	1	0	0	0	0	0	0	0	0	1	0	0	0	0	0	0	0	0	0	0	0	0	0	0	0	0	0	0	0	0	0	0	0	0	0	0	0	0	0	0	0	0	0	1	43.96	15.57
1966	1999/66	0	0	0	0	0	0	0	0	0	0	0	0	1	0	0	0	0	0	0	0	0	0	0	0	0	0	0	0	0	0	0	0	0	0	0	0	0	0	0	0	0	0	0	0	0	0	0	0	0	0	0	0	0	0	0	0	0	0	0	0	0	1	43.95	15.64
1968	1999/68	1	0	0	0	0	0	0	0	0	0	0	0	0	0	0	0	0	0	1	0	0	0	0	0	0	0	0	1	0	1	0	0	0	0	0	0	0	0	0	0	0	0	0	0	0	0	0	0	0	0	0	0	0	0	0	0	0	0	0	0	0	1	45.35	13.75
1969	1999/69	0	0	0	0	0	0	0	0	0	0	0	0	0	0	0	0	0	0	0	0	0	0	0	0	0	0	0	0	0	0	0	0	0	0	0	0	0	0	0	0	0	0	0	0	0	0	0	0	0	0	0	0	0	0	0	0	0	0	0	0	0	1	45.118607	13.807904
1971	1999/71	1	0	0	0	0	0	0	0	0	0	0	0	0	0	0	0	0	0	1	0	0	0	0	0	0	0	0	0	0	0	0	0	0	0	0	0	0	0	0	0	0	0	0	0	0	0	0	0	0	0	0	0	0	0	0	0	0	0	0	0	0	1	45.52	14.18
1972	1999/72	1	0	0	0	0	0	0	0	0	0	0	0	0	0	0	0	0	0	1	0	0	0	0	0	0	1	0	0	0	1	0	0	0	0	0	1	0	0	0	0	0	0	0	0	0	0	0	0	0	0	0	0	0	0	0	0	0	0	0	0	0	1	45.454033	14.282588
1973	1999/73	1	0	0	0	0	0	0	0	0	0	0	0	0	0	0	0	0	0	1	0	0	0	0	0	0	0	0	0	0	0	0	0	0	0	0	0	0	0	0	0	0	0	0	0	0	0	0	0	0	0	0	0	0	0	0	0	0	0	0	0	0	1	45.975406	13.650926
1974	1999/74	1	0	0	0	0	0	0	0	0	0	0	0	1	1	0	0	0	0	1	0	0	0	0	0	0	0	0	1	0	1	0	0	0	0	0	0	0	0	0	0	0	0	0	0	0	0	0	0	0	0	0	0	0	0	0	0	0	0	0	0	0	1	45.998094	13.635327
1975	1999/75	1	0	0	0	0	0	0	0	0	0	0	0	1	0	0	0	0	0	0	0	0	0	0	0	0	0	0	0	0	0	0	0	0	0	0	0	0	0	0	0	0	0	0	0	0	0	0	0	0	0	0	0	0	0	0	0	0	0	0	0	0	1	46.106428	13.683212
1977	1999/77	1	0	0	0	0	0	0	0	0	0	0	0	1	1	0	0	0	0	0	0	0	0	0	0	0	0	0	0	0	0	0	0	0	0	0	0	0	0	0	0	0	0	0	0	0	0	0	0	0	0	0	0	0	0	0	0	0	0	0	0	0	1	46.250935	13.581232
1978	1999/78	1	0	0	0	0	0	0	0	0	0	0	0	0	0	0	0	0	0	1	0	1	0	0	0	0	0	0	1	0	1	0	0	0	0	0	0	0	0	0	0	0	0	0	0	0	0	0	0	0	0	0	0	0	0	0	0	0	0	0	0	0	1	45.87	13.9
1980	1999/80	1	0	0	0	0	0	0	0	0	0	0	0	1	0	0	0	0	0	0	0	0	0	0	0	0	0	0	0	0	0	0	0	0	0	0	0	0	0	0	0	0	0	0	0	0	0	0	0	0	0	0	0	0	0	0	0	0	0	0	0	0	1	46.27	13.88
1981	1999/88	1	0	0	0	0	0	0	0	0	0	0	0	1	0	0	0	0	0	1	0	0	0	0	0	0	0	0	0	0	0	0	0	0	0	0	0	0	0	0	0	0	0	0	0	0	0	0	0	0	0	0	0	0	0	0	0	0	0	0	0	0	1	46.42	15.26
1982	1999/89	1	0	0	0	0	0	0	0	0	0	0	0	1	0	0	0	0	0	0	0	0	0	0	0	0	0	0	0	0	0	0	0	0	0	0	0	0	0	0	0	0	0	0	0	0	0	0	0	0	0	0	0	0	0	0	0	0	0	0	0	0	1	46.34	13.56
1983	1999/90	1	0	0	0	0	0	0	0	0	0	0	0	1	0	0	0	0	0	1	0	0	0	0	0	0	0	0	0	0	0	0	0	0	0	0	0	0	0	0	0	0	0	0	0	0	0	0	0	0	0	0	0	0	0	0	0	0	0	0	0	0	1	46.3	13.48
1984	1999/92	0	0	0	0	0	0	0	0	0	1	0	0	0	0	0	0	0	0	1	0	0	0	1	1	0	0	0	0	0	1	0	0	0	0	0	1	0	0	0	0	0	0	0	0	0	0	0	0	0	0	0	0	0	0	0	0	0	0	0	0	0	1	46.04	22.87
1985	1999/93	0	0	0	0	0	0	0	0	0	0	0	0	0	0	0	0	0	0	1	0	0	0	0	0	0	0	0	0	0	1	0	0	1	0	0	0	0	0	0	0	0	0	0	0	0	0	0	0	0	0	0	0	0	0	0	0	0	0	0	0	0	1	45.76	22.89
1986	1999/94	0	0	0	0	0	0	0	0	0	0	0	0	0	0	0	0	0	0	1	0	0	0	1	0	0	0	0	0	0	0	0	0	0	0	0	0	0	0	0	0	0	0	0	0	0	0	0	0	0	0	0	0	0	0	0	0	0	0	0	0	0	1	45.53	23.14
1987	1999/95	0	0	0	0	0	0	0	0	0	1	0	0	0	0	0	1	0	0	1	0	0	0	1	1	0	1	0	0	0	1	0	0	0	0	0	1	0	0	0	0	0	0	0	0	0	0	0	0	0	0	0	0	0	0	0	0	0	0	0	0	0	1	45.45	23.32
1988	1999/96	0	0	0	0	0	0	0	0	0	1	0	0	0	0	0	0	0	0	1	0	0	1	0	0	0	1	0	0	0	1	0	0	0	0	0	1	0	0	0	0	0	0	0	0	0	0	0	0	0	0	0	0	0	0	0	0	0	0	0	0	0	1	45.47	23.18
1990	1999/98	0	0	0	0	0	0	0	0	0	1	0	0	0	0	0	0	0	0	1	0	0	0	1	1	0	0	0	0	0	1	0	0	1	0	0	0	0	0	0	0	1	0	0	0	0	0	0	0	0	0	0	0	0	0	0	0	0	0	0	0	0	1	45.16	24.05
1991	1999/99	0	0	0	0	0	0	0	0	0	0	0	0	0	0	0	0	0	0	1	0	0	0	0	0	0	0	0	0	0	1	0	0	1	0	0	1	0	0	0	0	1	0	0	0	0	0	0	0	0	0	0	0	0	0	0	0	0	0	0	0	0	1	45.22	24.22
1992	2000/01	0	0	0	0	0	0	0	0	0	0	0	0	1	0	0	0	0	0	1	0	0	0	0	0	0	0	0	0	0	0	0	0	0	0	0	0	0	0	1	0	0	0	0	0	0	0	0	0	0	0	0	0	0	0	0	0	0	0	0	0	0	1	44.68653333	17.1946
1993	2000/02	0	0	0	0	0	0	0	0	0	0	0	0	0	0	0	0	0	0	1	0	0	0	0	0	0	0	0	0	0	0	0	0	0	0	0	0	0	0	1	0	0	0	0	0	0	0	0	0	0	0	0	0	0	0	0	0	0	0	0	0	0	1	44.60721667	17.13863333
1994	2000/03	1	0	0	0	0	0	0	0	0	1	0	0	0	0	0	0	0	0	1	0	0	0	0	0	0	0	0	0	0	1	0	0	0	0	0	0	0	0	1	0	0	0	0	0	0	0	0	0	0	0	0	0	0	0	0	0	0	0	0	0	0	1	44.47268333	17.19521667
1995	2000/04	1	0	0	0	0	0	0	0	0	0	0	0	0	0	0	0	0	0	1	0	0	0	0	0	0	0	0	0	0	1	0	0	0	0	0	0	1	0	0	0	0	0	0	0	0	0	0	0	0	0	0	0	0	0	0	0	0	0	0	0	0	1	44.4105	17.2607

Table S1.5

1996	2000/05	1	0	0	0	0	1	0	0	0	0	0	0	1	0	0	0	0	0	1	0	0	0	0	0	0	0
1998	2000/07	0	0	0	0	0	0	0	0	0	0	0	0	0	0	0	0	0	0	1	0	0	0	0	0	0	0
1999	2000/08	1	0	0	0	0	0	0	0	0	0	0	0	1	0	0	0	0	0	1	0	0	0	0	0	0	0
2002	2000/11	1	0	0	0	0	0	0	0	0	0	0	0	1	0	0	0	0	0	1	0	0	0	0	0	0	0
2003	2000/12	1	0	0	0	0	0	0	0	0	0	0	0	1	0	0	0	0	0	1	0	0	0	0	0	0	0
2004	2000/13	1	0	0	0	0	0	0	0	0	0	0	0	0	0	0	1	0	0	1	0	0	0	0	0	0	0
2005	2000/62	1	0	0	0	0	1	0	0	0	1	0	0	1	0	0	0	0	0	0	0	0	0	0	0	0	0
2006	2000/63	0	0	0	0	0	1	0	0	0	0	0	0	1	0	0	0	0	0	0	0	0	0	0	0	0	0
2007	2000/64	0	0	0	0	0	1	0	0	0	0	0	0	0	0	0	0	0	0	0	0	0	0	0	0	0	0
2008	2000/65	0	0	0	0	0	0	0	0	0	0	0	0	0	0	1	0	0	0	0	0	0	0	0	0	0	0
2009	2000/66	0	0	0	0	0	0	0	0	0	0	0	0	1	0	0	0	0	0	0	0	0	0	0	0	0	0
2010	2000/67	1	0	0	0	0	0	0	0	0	1	0	0	0	0	0	0	0	0	1	0	0	0	0	0	0	0
2011	2000/68	0	0	0	0	0	0	0	0	0	0	0	0	0	0	0	0	0	0	1	0	0	0	0	0	0	0
2012	2000/86	0	0	0	0	0	0	0	0	0	1	0	0	0	0	0	0	0	0	0	0	0	0	0	0	0	0
2013	2000/87	0	0	0	0	0	0	0	0	0	1	0	0	0	0	0	0	0	0	1	0	0	0	1	0	0	0
2014	2000/88	0	0	0	0	0	0	0	0	0	0	0	0	0	0	0	0	0	0	1	0	0	0	0	0	0	0
2015	2000/88B	0	0	0	0	0	0	0	0	0	0	0	0	0	0	0	0	0	0	0	0	0	0	0	0	0	0
2016	2000/89	0	0	0	0	0	0	0	0	0	1	0	0	0	0	0	0	0	0	1	0	0	0	1	0	0	0
2017	2000/90	0	0	0	0	0	0	0	0	0	0	0	0	0	0	0	0	0	0	0	0	0	0	0	0	0	0
2018	2000/91	0	0	0	0	0	0	0	0	0	0	0	0	0	0	0	0	0	0	0	0	0	0	0	0	0	0
2019	2000/92	0	0	0	0	0	0	0	0	0	0	0	0	0	0	0	0	0	0	0	0	0	0	0	0	0	0
2020	2000/93	0	0	0	0	0	0	0	0	0	0	0	0	0	0	0	0	0	0	0	0	0	0	1	0	0	0
2021	2000/94	0	0	0	0	0	0	0	0	0	0	0	0	0	0	0	0	0	0	0	0	0	0	0	0	0	0
2022	2000/95	0	0	0	0	0	0	0	0	0	0	0	0	0	0	0	0	0	0	0	0	0	0	0	0	0	0
2023	2000/96	0	0	0	0	0	0	0	0	0	0	0	0	0	0	0	0	0	0	0	0	0	0	0	0	0	0
2024	2000/98	0	0	0	0	0	0	0	0	0	0	0	0	0	0	0	0	0	0	1	0	0	0	1	0	0	0
2025	2000/99	0	0	0	0	0	0	0	0	0	0	0	0	0	0	0	0	0	0	0	0	0	0	0	0	0	0
2027	2002/001	0	0	0	0	0	1	0	0	0	0	0	0	1	0	0	0	0	0	1	0	0	0	0	0	0	0
2028	2002/002	0	0	0	0	0	1	0	0	0	0	0	0	1	0	0	0	0	0	1	0	0	0	0	0	0	0
2030	2002/056	1	0	0	0	0	0	0	0	0	0	0	0	1	0	0	0	0	0	0	0	0	0	0	0	0	0
2031	2002/057	0	0	0	0	0	1	0	0	0	0	0	0	1	0	0	0	0	0	0	0	0	0	0	0	0	0
2032	2002/058	1	0	0	0	0	1	0	0	0	0	0	0	1	0	0	0	0	0	0	0	0	0	0	0	0	0
2033	2002/059	1	0	0	0	0	0	0	0	0	0	0	0	1	0	0	0	0	0	1	0	0	0	0	0	0	0
2034	2002/060	1	0	0	0	0	1	0	0	0	0	0	0	1	0	0	0	0	0	0	0	0	0	0	0	0	0
2035	2002/061	0	0	0	0	0	0	0	0	0	0	0	0	0	0	0	0	0	0	1	0	0	0	0	0	0	0
2036	2002/062	1	0	0	0	0	1	0	0	0	1	0	0	1	0	0	0	0	0	1	0	0	0	0	0	0	1
2037	2002/063	1	0	0	0	0	0	0	0	0	0	0	0	1	0	0	0	0	0	0	0	0	0	0	0	0	0
2038	2002/064	0	0	0	0	0	0	0	0	0	0	0	0	1	0	0	0	0	0	1	0	0	0	0	0	0	0
2039	2002/065	1	0	0	0	0	0	0	0	0	0	0	0	0	0	0	0	0	0	1	1	0	0	0	0	0	0
2040	2002/066	1	0	0	0	0	1	0	0	0	0	0	0	1	0	0	0	0	0	1	0	0	0	0	0	0	0
2041	2002/067	0	0	0	0	0	1	0	0	0	0	0	0	0	0	0	0	0	0	0	0	0	0	0	0	0	0
2042	2002/068	1	0	0	0	0	1	0	0	0	0	0	0	1	0	0	0	0	0	1	0	0	0	0	0	0	0
2043	2002/069	0	0	0	0	0	0	0	0	0	0	0	0	1	0	0	0	0	0	0	0	0	0	0	0	0	0
2044	2002/070	1	0	0	0	0	0	0	0	0	0	0	0	1	0	0	0	0	0	1	1	0	0	0	0	0	0
2045	2002/071	1	0	0	0	0	1	0	0	0	0	0	0	0	0	0	0	0	0	0	0	0	0	0	0	0	1
2046	2002/072	1	0	0	1	0	0	0	0	0	0	0	0	0	0	0	0	0	0	1	1	0	0	0	0	0	0
2047	2002/073	0	0	0	0	0	1	0	0	0	0	0	0	0	0	0	0	0	0	1	0	0	0	0	0	0	0
2048	2002/074	1	0	0	0	0	1	0	0	0	0	0	0	0	0	0	0	0	0	1	0	0	0	0	0	0	0
2049	2002/075	0	0	0	0	0	0	0	0	0	0	0	0	0	0	0	0	0	0	1	0	0	0	0	0	0	0
2050	2002/076	1	0	0	1	0	0	0	0	0	0	0	0	1	0	1	0	0	0	1	0	1	0	1	0	0	0
2051	2002/077	0	0	0	0	0	0	0	0	0	0	0	0	0	0	0	0	0	0	0	0	0	0	0	0	0	0
2052	2002/078	1	0	0	1	0	1	0	0	0	0	0	0	1	0	0	0	0	0	1	0	0	0	0	0	0	0
2053	2002/079	1	0	0	1	0	0	0	0	0	0	0	0	0	0	0	0	0	0	1	0	0	0	0	0	0	0
2054	2002/080	1	0	0	0	0	0	0	0	0	0	0	0	1	0	0	0	0	0	1	0	0	0	0	0	0	0
2055	2002/081	1	0	0	0	0	0	0	0	0	0	0	0	0	0	0	0	0	0	1	0	0	0	0	0	0	0
2056	2002/082	0	0	0	0	0	1	0	0	0	0	0	0	0	0	0	0	0	0	0	0	0	0	0	0	0	0
2057	2002/083	1	0	0	0	0	0	0	0	0	0	0	0	1	0	0	0	0	0	1	0	0	0	0	0	0	0
2058	2002/084	0	0	0	0	0	0	0	0	0	0	0	0	0	0	0	0	0	0	1	0	0	0	0	0	0	0
2059	2002/085	1	0	0	0	0	0	0	0	0	0	0	0	1	0	0	0	0	0	1	0	0	0	0	0	0	0
2060	2002/086	0	0	0	0	0	0	0	0	0	0	0	0	1	0	0	0	0	0	0	0	0	0	0	0	0	0
2061	2002/087	1	0	0	0	0	0	0	0	0	0	0	0	0	0	0	0	0	0	1	0	0	0	0	0	0	0
2062	2002/088	0	0	0	0	0	0	0	0	0	0	0	0	1	0	0	0	0	0	1	0	0	0	0	0	0	0
2063	2002/089	0	0	0	0	0	0	0	0	0	0	0	0	1	0	0	0	0	0	1	0	0	0	0	0	0	0
2064	2002/090	0	0	0	0	0	1	0	0	0	0	0	0	0	0	0	0	0	0	1	0	0	0	0	0	0	0
2065	2002/091	1	0	0	0	0	0	0	0	0	0	0	0	1	0	0	0	0	0	1	0	0	1	0	0	0	0
2066	2004/071	1	0	0	0	0	0	0	0	0	0	0	0	1	0	0	0	0	0	0	0	0	0	0	0	0	0
2067	2004/072	1	0	0	0	0	1	0	0	0	0	0	0	0	1	0	0	0	0	1	1	0	0	0	0	0	1
2068	2004/073	0	0	0	0	0	0	0	0	0	0	0	0	1	0	0	0	0	0	0	0	0	0	0	0	0	0
2071	2005/025	0	0	0	0	0	1	0	0	0	0	0	0	0	0	0	0	0	0	1	0	0	0	0	0	0	0
2072	2005/039	0	0	0	0	0	0	0	0	0	0	0	0	0	0	0	0	0	0	0	0	0	0	0	0	0	0
2073	2005/040	0	0	0	0	0	0	0	0	0	0	0	0	0	0	0	0	0	0	1	0	0	0	0	0	0	0
2082	2006/096	0	0	0	0	0	0	0	0	0	0	0	0	0	0	0	0	0	0	1	0	0	0	0	0	0	0
2087	2006/101	0	0	0	0	0	0	0	0	0	0	0	0	0	0	0	0	0	0	1	0	0	0	0	0	0	0
2100	2007/004	0	0	0	0	0	0	0	0	0	0	0	0	0	0	0	0	0	0	0	0	0	0	0	0	0	0
2102	2007/006	0	0	0	0	0	0	0	0	0	0	0	0	0	0	0	0	0	0	0	0	0	0	0	0	0	0
2103	2007/007	0	0	0	0	0	0	0	0	0	0	0	0	0	0	0	0	0	0	0	0	0	0	0	0	0	0
2109	2007/013	0	0	0	0	0	0	0	0	0	0	0	0	0	0	0	0	0	0	0	0	0	0	0	0	0	0
2111	2007/015	0	0	0	0	0	0	0	0	0	0	0	0	0	0	0	0	0	0	0	0	0	0	0	0	0	0
2112	2007/016	0	0	0	0	0	0	0	0	0	0	0	0	0	0	0	0	0	0	0	0	0	0	0	0	0	0
2113	2007/017	0	0	0	0	0	0	0	0	0	0	0	0	0	0	0	0	0	0	0	0	0	0	0	0	0	0
2114	2007/018	0	0	0	0	0	0	0	0	0	0	0	0	0	0	0	0	0	0	0	0	0	0	0	0	0	0
2117	2007/021	0	0	0	0	0	0	0	0	0	0	0	0	0	0	0	0	0	0	0	0	0	0	0	0	0	0
2119	2007/023	0	0	0	0	0	0	0	0	0	0	0	0	0	0	0	0	0	0	0	0	0	0	0	0	0	0
2127	2007/031	0	0	0	0	0	0	0	0	0	0	0	0	0	0	0	0	0	0	0	0	0	0	0	0	0	0
2128	2007/032	0	0	0	0	0	0	0	0	0	0	0	0	0	0	0	0	0	0	0	0	0	0	0	0	0	0
2129	2007/033	0	0	0	0	0	0	0	0	0	0	0	0	0	0	0	0	0	0	0	0	0	0	0	0	0	0
2130	2007/034	0	0	0	0	0	0	0	0	0	0	0	0	0	0	0	0	0	0	0	0	0	0	0	0	0	0
2131	2007/035	0	0	0	0	0	0	0	0	0	0	0	0	0	0	0	0	0	0	0	0	0	0	0	0	0	0
2137	2007/041	0	0	0	0	0	0	0	0	0	0	0	0	0	0	0	0	0	0	0	0	0	0	0	0	0	0
2139	2007/043	0	0	0	0	0	0	0	0	0	0	0	0	0	0	0	0	0	0	0	0	0	0	0	0	0	0
2142	2007/046	0	0	0	0	0	0	0	0	0	0	0	0	0	0	0	0	0	0	0	0	0	0	0	0	0	0
2143	2007/047	0	0	0	0	0	0	0	0	0	0	0	0	0	0	0	0	0	0	0	0	0	0	0	0	0	0
2145	2007/048	0	0	0	0	0	0	0	0	0	0	0	0	0	0	0	0	0	0	0	0	0	0	0	0	0	0
2146	2007/049	0	0	0	0	0	0	0	0	0	0	0	0	0	0	0	0	0	0	0	0	0	0	0	0	0	0
2149	2007/052	0	0	0	0	0	0	0	0	0	0	0	0	0	0	0	0	0	0	0	0	0	0	0	0	0	0
2151	2007/054	0	0	0	0	0	0	0	0	0	0	0	0	0	0	0	0	0	0	0	0	0	0	0	0	0	0
2152	2007/055	0	0	0	0	0	0	0	0	0	0	0	0	0	0	0	0	0	0	0	0	0	0	0	0	0	0
2154	2007/057	0	0	0	0	0	0	0	0	0	0	0	0	0	0	0	0	0	0	0	0	0	0	0	0	0	0
2156	2007/059	0	0	0	0	0	0	0	0	0	0	0	0	0	0	0	0	0	0	0	0	0	0	0	0	0	0
2157	2007/060	0	0	0	0	0	0	0	0	0	0	0	0	0	0	0	0	0	0	0	0	0	0	0	0	0	0
2160	2007/063	0	0	0	0	0	0	0	0	0	0	0	0	0	0	0	0	0	0	0	0	0	0	0	0	0	0
2173	2007/076	0	0	0	0	0	0	0	0	0	0	0	0	0	0	0	0	0	0	0	0	0	0	0	0	0	0
2175	2007/078	0	0	0	0	0	0	0	0	0	0	0	0	0	0	0	0	0	0	0	0	0	0	0	0	0	0
2176	2007/079	0	0	0	0	0	0	0	0	0	0	0	0	0	0	0	0	0	0	0	0	0	0	0	0	0	0
2188	2008/084	0	0	0	0	0	0	0	0	0	0	0	0	0	0	0	0	0	0	0	0	0	0	0	0	0	0
2189	2008/085	0	0	0	0	0	0	0	0	0	0	0	0	0	0	0	0	0	0	0	0	0	0	0	0	0	0
2190	2008/086	1	0	0	0	0	0	0	0	0	0	0	0	0	0	0	0	0	0	1	0	0	0	0	0	0	0
2191	2008/087	1	0	0	0	0	0	0	0	0	0	0	0	0	0	0	0	0	0	1	0	0	0	0	0	0	0
2192	2008/088	1	0	0	0	0	0	0	0	0	0	0	0	0	0	0	0	0	0	0	0	0	0	0	0	0	0
2193	2008/089	0	0	0	0	0	0	0	0	0	0	0	0	0	0	0	0	0	0	0	0	0	0	0	0	0	0
2194	2008/101	1	0	0	0	0	0	0	0	0	0	0	0	0	0	0	0	0	0	0	0	0	0	0	0	0	0
2196	2008/103	1	0	0	0	0	1	0	0	0	1	0	0	1	0	1	0	0	0	1	0	0	0	0	0	0	0
2197	2008/104	0	0	0	0	0	0	0	0	0	0	0	0	0	0	0	0	0	0	0	0	0	0	0	0	0	0
2198	2008/105	1	0	0	0	0	0	0	0	0	0	0	0	0	0	0	0	0	0	0	0	0	0	0	0	0	0
2199	2008/106	0	0	0	0	0	0	0	0	0	0	0	0	0	0	0	0	0	0	0	0	0	0	0	0	0	0
2200	2008/107	0	0	0	0	0	0	0	0	0	0	0	0	1	0	0	0	0	0	0	0	0	0	0	0	0	0
2201	2008/108	0	0	0	0	0	0	0	0	0	0	0	0	0	0	0	0	0	0	0	0	0	0	0	0	0	0
2202	2008/109	0	0	0	0	0	0	0	0	0	0	0	0	0	0	0	0	0	0	0	0	0	0	0	0	0	0
2211	2009/095	0	0	0	0	0	0	0	0	0	0	0	0	0	0	0	0	0	0	0	0	0	0	0	0	0	0
2218	2009/102	0	0	0	0	0	0	0	0	0	0	0	0	0	0	0	0	0	0	0	0	0	0	0	0	0	0
2223	2009/107	0	0	0	0	0	0	0	0	0	0	0	0	1	0	0	0	0	0	1	0	0	0	0	0	0	0
2224	2009/108	0	0	0	0	0	0	0	0	0	0	0	0	0	0	0	0	0	0	0	0	0	0	0	0	0	0
2227	2009/111	0	0	0	0	0	0	0	0	0	0	0	0	0	0	0	0	0	0	1	0	0	0	0	0	0	0
2228	2009/112	0	0	0	0	0	0	0	0	0	0	0	0	0	0	0	0	0	0	0	0	0	0	0	0	0	0
2230	2009/114	0	0	0	0	0	0	0	0	0	0	0	0	1	0	0	0	0	0	0	0	0	0	0	0	0	0
2232	2009/116	0	0	0	0	0	0	0	0	0	0	0	0	0	0	0	0	0	0	1	0	0	0	0	0	0	0
2235	2009/119	0	0	0	0	0	0	0	0	0	0	0	0	0	0	1	0	0	0	1	0	0	0	0	0	0	0
2236	2009/120	0	0	0	0	0	0	0	0	0	0	0	0	0	0	1	0	0	0	1	0	0	0	0	0	0	0
2237	2009/121	0	0	0	0	0	0	0	0	0	0	0	0	0	0	1	0	0	0	0	0	0	0	0	0	0	0
2238	2009/122	0	0	0	0	0	0	0	0	0	0	0	0	0	0	1	0	0	0	0	0	0	0	0	0	0	0
2239	2009/130	1	0	0	0	0	0	0	0	1	1	0	0	1	0	0	1	0	1	1	0	1	1	1	0	1	0
2245	2009/136	0	0	0	0	0	0	0	0	0	0	0	0	0	0	0	0	0	0	0	0	0	0	0	0	0	0
2247	2009/138	1	1	0	0	0	1	1	0	1	1	0	0	1	0	1	1	1	0	0	0	0	0	1	1	0	0
2248	2009/139	1	0	0	0	1	1	0	0	0	0	0	0	1	0	1	0	0	0	1	0	1	1	1	0	1	1
2250	2009/141	0	0	0	0	0	0	0	0	0	0	0	0	1	0	0	0	0	0	0	0	0	0	0	0	0	0
2251	2009/142	0	0	0	0	0	0	0	0	0	0	0	0	0	0	0	0	0	0	0	0	0	0	0	0	0	0
2255	2009/146	0	0	0	0	0	1	0	0	0	0	0	0	0	0	0	0	0	0	0	0	0	0	0	0	0	0
2261	2009/152	0	0	0	0	0	0	0	0	0	0	0	0	1	0	0	0	0	0	0	0	0	0	0	0	0	0
2264	2009/155	0	0	0	0	0	0	0	0	0	0	0	0	0	0	0	0	0	0	1	0	0	0	0	0	0	0
2265	2009/156	0	0	0	0	0	1	0	1	1	1	0	0	0	0	0	0	0	0	1	0	1	0	0	0	0	0
2267	2009/158	0	0	0	0	0	0	0	0	0	0	0	0	0	0	0	0	0	0	0	0	0	0	0	0	0	0
2276	2011/010	1	0	0	0	0	0	0	0	0	0	0	0	0	0	0	0	0	0	0	0	0	0	0	0	0	0
2277	2011/011	0	0	0	0	0	0	0	0	0	0	0	0	1	0	1	0	0	0	0	0	0	0	0	0	0	0
2279	2012/213	1	0	0	0	0	0	0	0	0	0	0	0	0	0	0	0	0	0	1	0	0	0	0	0	0	0
2281	2014/002	0	0	0	0	0	0	0	0	0	0	0	0	0	0	0	0	0	0	0	0	0	0	0	0	0	0
2282	2014/003	1	0	0	0	0	1	0	0	0	0	0	0	1	1	1	0	0	0	1	0	0	0	0	0	0	0
2286	2014/007	1	0	0	0	0	1	0	0	0	1	0	0	1	1	1	0	0	0	1	0	0	0	0	0	0	0
2289	2014/010	0	0	0	0	0	0	0	0	0	0	0	0	0	0	0	0	0	0	0	0	0	0	0	0	0	0
2291	2014/012	0	0	0	0	0	0	0	0	0	0	0	0	0	0	0	0	0	0	1	0	0	0	0	0	0	0
2292	2014/013	0	0	0	0	0	1	0	0	0	0	0	0	0	0	0	0	0	0	0	0	0	0	0	0	0	0
2293	2014/014	0	0	0	0	0	0	0	0	0	0	0	0	0	0	0	0	0	0	0	0	0	0	0	0	0	0
2294	2014/015	1	0	0	0	0	1	0	0	0	0	0	0	1	0	1	0	0	0	1	0	0	0	1	0	0	0
2297	2014/018	1	0	0	0	0	1	0	0	1	1	0	0	1	0	1	0	0	0	0	0	0	0	0	0	0	0
2298	2014/019	1	0	0	0	0	0	0	0	0	0	0	0	0	0	1	0	0	0	1	0	0	0	0	0	0	0
2299	2014/020	0	0	0	0	0	1	0	0	0	0	0	0	0	0	0	0	0	0	1	0	0	0	0	0	0	0
2300	2014/021	0	0	0	0	0	0	0	0	0	0	0	0	0	0	0	0	0	0	0	0	0	0	0	0	0	0
2301	2014/022	0	0	0	0	0	0	0	0	0	0	0	0	0	0	0	0	0	0	1	0	0	0	0	0	0	0
2302	2014/023	1	0	0	0	0	0	0	0	0	1	0	0	0	1	0	0	0	0	1	0	0	0	1	0	0	0
2303	2014/024	0	0	0	0	0	1	0	0	1	0	0	0	0	0	1	0	0	0	1	0	0	0	0	0	0	0
2304	2014/025	0	0	0	0	0	1	0	0	1	0	0	0	0	0	0	1	0	0	1	0	0	0	0	0	0	0
2305	2014/026	0	0	0	0	0	0	0	0	0	0	0	0	0	0	0	0	0	0	1	0	0	0	0	0	0	0
2307	2014/028	0	0	0	0	0	0	0	0	0	0	0	0	1	0	0	0	0	0	0	0	0	0	0	0	0	0
2308	2014/029	1	0	0	0	0	1	0	0	1	0	0	0	1	1	1	0	0	0	0	0	0	0	0	0	0	0
2312	2014/033	1	0	0	0	1	1	0	0	1	0	0	0	1	0	0	0	0	0	0	0	0	0	0	0	0	0
2314	2014/035	1	0	0	0	0	1	0	0	1	0	0	0	1	1	0	1	0	0	1	0	0	0	1	0	0	0
2315	2014/036	1	0	0	0	0	1	0	0	1	0	0	0	1	0	1	0	0	0	1	0	0	0	0	0	0	1
2316	2014/037	0	0	0	0	0	0	0	0	0	0	0	0	0	0	0	0	0	0	0	0	0	0	0	0	0	0
2317	2014/038	0	0	0	0	0	0	0	0	0	0	0	0	0	0	0	0	0	0	0	0	0	0	0	0	0	0
2321	2014/042	0	0	0	0	0	0	0	0	0	0	0	0	0	1	1	0	0	0	0	0	0	0	0	0	0	0
2324	2014/045	1	0	0	0	0	1	0	0	0	1	0	0	1	1	1	0	0	0	0	0	0	0	0	0	0	0
2325	2014/046	0	0	0	0	0	0	0	0	0	0	0	0	1	1	1	0	0	0	1	0	0	0	0	0	0	0
2326	2014/047	1	0	0	0	0	0	0	0	0	0	0	0	0	0	0	1	0	0	1	0	1	0	0	0	0	0
2329	2014/050	1	0	1	0	0	1	0	0	0	0	0	0	1	0	1	0	0	0	1	0	0	0	1	0	0	0
2330	2014/051	1	0	0	0	0	1	0	0	0	0	0	0	1	1	1	0	0	0	1	0	0	0	0	0	0	0
2331	2014/052	0	0	0	0	0	0	0	0	0	0	0	0	0	0	0	0	0	0	0	0	0	0	0	0	0	0
2332	2014/053	0	0	0	0	0	0	0	0	0	0	0	0	0	0	0	0	0	0	1	0	0	0	0	0	0	0

1996	2000/05	0	0	0	1	0	0	0	0	0	0	0	0	0	0	0	0	0	0	0	0	0	0	0	0	0	0	0	0	0	0	0	0	0	0	0	1	44.347	17.263
1998	2000/07	0	1	0	0	0	0	0	0	0	0	0	0	0	0	0	0	0	0	0	0	0	0	0	0	0	0	0	0	0	0	0	0	0	0	0	1	44.22948333	17.66846667
1999	2000/08	0	0	0	1	0	0	0	0	0	1	0	0	0	0	0	0	0	0	0	0	0	0	0	0	0	0	0	0	0	0	0	0	0	0	0	2	44.27886667	17.59206667
2002	2000/11	0	0	0	1	0	0	0	0	0	0	0	0	1	0	0	0	0	0	0	0	0	0	0	0	0	0	0	0	0	0	0	0	0	0	0	1	43.76993333	18.39406667
2003	2000/12	0	0	0	0	0	0	0	0	0	1	0	0	1	0	0	0	0	0	0	0	0	0	0	0	0	0	0	0	0	0	0	0	0	0	0	1	43.70976667	18.43921667
2004	2000/13	0	0	0	1	0	0	0	0	0	0	0	0	0	0	0	0	0	0	0	0	0	0	0	0	0	0	0	0	0	0	0	0	0	0	0	1	43.57601667	18.53733333
2005	2000/62	0	0	0	0	1	0	0	0	0	0	0	0	0	0	0	0	0	1	0	0	0	0	0	0	0	0	0	0	0	0	0	0	0	0	0	1	42.7	18.32
2006	2000/63	0	0	1	0	0	0	0	0	0	0	0	0	1	0	0	0	0	0	0	0	0	0	0	0	0	0	0	0	0	0	0	0	0	0	0	1	43.055	18.506
2007	2000/64	0	0	0	0	0	0	0	0	0	0	0	0	1	0	0	0	0	0	0	0	0	0	0	0	0	0	0	0	0	0	0	0	0	0	0	1	43.231649	18.40225
2008	2000/65	0	0	0	0	0	0	0	0	0	0	0	0	1	0	0	0	0	0	0	0	0	0	0	0	0	0	0	0	0	0	0	0	0	0	0	1	43.24776667	18.38158333
2009	2000/66	0	0	0	0	0	0	0	0	0	0	0	0	1	0	0	0	0	0	0	0	0	0	0	0	0	0	0	0	0	0	0	0	0	0	0	1	43.25101667	18.33955
2010	2000/67	0	0	0	0	0	0	0	0	0	0	0	0	0	0	0	0	0	0	0	0	0	0	0	0	0	0	0	0	0	0	0	0	0	0	0	1	43.35058333	17.89568333
2011	2000/68	0	0	0	0	0	0	0	0	0	0	0	0	1	0	0	0	0	0	0	0	0	0	0	0	0	0	0	0	0	0	0	0	0	0	0	1	43.6674	17.95896667
2012	2000/86	0	0	0	1	0	0	1	0	0	1	0	0	0	0	1	0	0	0	0	0	0	0	0	0	0	0	0	0	0	0	0	0	0	0	0	1	45.35648333	23.85803333
2013	2000/87	0	0	0	1	0	0	1	0	0	0	0	0	0	0	1	0	0	0	0	0	0	0	0	0	0	0	0	0	0	0	0	0	0	0	0	1	45.41753333	23.9158
2014	2000/88	0	0	0	0	0	0	1	0	0	1	0	0	0	0	0	0	0	0	0	0	0	0	0	0	0	0	0	0	0	0	0	0	0	0	0	1	45.39733333	23.904
2015	2000/88B	0	0	0	0	0	0	0	0	0	0	0	0	0	0	1	0	0	0	0	0	0	0	0	0	0	0	0	0	0	0	0	0	0	0	0	1	45.39445	23.91086667
2016	2000/89	0	0	0	1	0	0	1	0	0	1	0	0	0	0	0	0	0	0	0	0	0	0	0	0	0	0	0	0	0	0	0	0	0	0	0	1	45.55243333	25.89996667
2017	2000/90	0	0	0	0	0	0	1	0	0	0	0	0	0	0	0	0	0	0	0	0	0	0	0	0	0	0	0	0	0	0	0	0	0	0	0	1	45.56693333	25.88425
2018	2000/91	0	0	0	1	0	0	1	0	0	1	0	0	0	0	0	0	0	0	0	0	0	0	0	0	0	0	0	0	0	0	0	0	0	0	0	1	45.5461	25.8905
2019	2000/92	0	0	0	1	0	0	1	0	0	0	0	0	0	0	0	0	0	0	0	0	0	0	0	0	0	0	0	0	0	0	0	0	0	0	0	1	45.55161667	25.90681667
2020	2000/93	0	0	0	1	0	0	1	0	0	0	0	0	0	0	0	0	0	0	0	0	0	0	0	0	0	0	0	0	0	0	0	0	0	0	0	1	45.56238333	25.93621667
2021	2000/94	0	0	0	1	0	0	1	0	0	0	0	0	0	0	0	0	0	0	0	0	0	0	0	0	0	0	0	0	0	0	0	0	0	0	0	1	45.55963333	25.89625
2022	2000/95	0	0	0	1	0	0	1	0	0	0	0	0	0	0	0	0	0	0	0	0	0	0	0	0	0	0	0	0	0	0	0	0	0	0	0	1	45.55736667	25.89158333
2023	2000/96	0	0	0	0	0	0	1	0	0	0	0	0	0	0	0	0	0	0	0	0	0	0	0	0	0	0	0	0	0	0	0	0	0	0	0	1	45.56066667	25.8836
2024	2000/98	0	0	0	0	0	0	1	0	0	0	0	0	0	0	0	0	0	0	0	0	0	0	0	0	0	0	0	0	0	0	0	0	0	0	0	1	45.53561667	25.90751667
2025	2000/99	0	0	0	1	0	0	1	0	0	0	0	0	0	0	0	0	0	0	0	0	0	0	0	0	0	0	0	0	0	0	0	0	0	0	0	1	45.56756667	25.60383333
2027	2002/001	0	0	0	0	0	0	0	1	0	0	0	0	0	0	0	0	0	0	0	0	0	0	0	0	0	0	0	0	0	0	0	0	0	0	0	1	43.333195	22.141906
2028	2002/002	0	0	0	1	0	0	0	1	0	0	0	0	0	0	0	0	0	0	0	0	0	0	0	0	0	0	0	0	0	0	0	0	0	0	0	1	43.33568333	22.13305
2030	2002/056	0	0	0	1	0	0	0	0	0	1	0	0	0	0	0	0	0	0	0	0	0	0	0	0	0	0	0	0	0	0	0	0	0	0	0	1	45.36228	14.760714
2031	2002/057	0	0	0	0	0	0	0	0	0	1	0	0	0	0	0	0	0	0	0	0	0	0	0	0	0	0	0	0	0	0	0	0	0	0	0	1	45.30163333	14.67318333
2032	2002/058	0	1	0	1	0	0	0	0	0	1	0	0	0	0	0	0	0	0	0	0	0	0	0	0	0	0	0	0	0	0	0	0	0	0	0	1	45.29755	14.65805
2033	2002/059	0	0	0	0	0	0	0	0	0	0	0	0	0	0	0	1	0	0	0	0	0	0	0	0	0	0	0	0	0	0	0	0	0	0	0	1	45.28635	14.64531667
2034	2002/060	0	1	0	0	0	0	0	0	0	0	0	0	0	0	0	1	0	0	0	0	0	0	0	0	0	0	0	0	0	0	0	0	0	0	0	1	45.28463333	14.62855
2035	2002/061	0	0	0	0	0	0	0	0	0	0	0	0	0	0	0	0	0	0	0	0	0	0	0	0	0	0	0	0	0	0	0	0	0	0	0	1	45.324709	14.538775
2036	2002/062	0	0	0	0	0	0	0	0	0	0	0	0	0	0	0	0	0	0	0	0	0	0	0	0	0	0	0	0	0	0	0	0	0	0	0	1	45.33175	14.54301667
2037	2002/063	0	0	0	0	0	0	0	0	0	0	0	0	0	0	0	1	0	0	0	0	0	0	0	0	0	0	0	0	0	0	0	0	0	0	0	1	44.98261667	14.9754
2038	2002/064	0	1	0	0	0	0	0	0	0	0	0	0	0	0	0	0	0	0	0	0	0	0	0	0	0	0	0	0	0	0	0	0	0	0	0	1	44.96866	14.973473
2039	2002/065	0	1	0	0	0	0	0	0	0	0	0	0	0	0	0	1	0	0	0	0	0	0	0	0	0	0	0	0	0	0	0	0	0	0	0	1	44.97933333	14.94933333
2040	2002/066	0	0	0	0	0	0	0	0	0	0	0	1	0	0	0	1	0	0	0	0	0	0	0	0	0	0	0	0	0	0	0	0	0	0	0	1	44.52836667	15.14398333
2041	2002/067	0	0	0	0	0	0	0	0	0	0	1	0	0	0	0	0	0	0	0	0	0	0	0	0	0	0	0	0	0	0	0	0	0	0	0	1	44.44111667	15.51913333
2042	2002/068	0	1	0	0	0	0	0	0	0	0	1	0	0	0	0	1	0	0	0	0	0	0	0	0	0	0	0	0	0	0	0	0	0	0	0	1	44.20158333	15.69965
2043	2002/069	0	1	0	0	0	0	0	0	0	0	1	0	0	0	0	0	0	0	0	0	0	0	0	0	0	0	0	0	0	0	0	0	0	0	0	1	43.93758333	15.44123333
2044	2002/070	0	1	0	0	0	0	0	0	0	0	0	0	0	0	0	0	0	0	0	0	0	0	0	0	0	0	0	0	0	0	0	0	0	0	0	1	43.5279	15.9689
2045	2002/071	0	0	0	0	0	0	0	0	0	0	1	0	0	0	0	0	0	0	0	0	0	0	0	0	0	0	0	0	0	0	0	0	0	0	0	1	43.55986667	16.40255
2046	2002/072	0	1	0	0	0	0	0	0	0	0	0	0	0	0	0	0	0	0	0	0	0	0	0	0	0	0	0	0	0	0	0	0	0	0	0	1	43.5092	16.42173333
2047	2002/073	0	1	0	0	0	0	0	0	0	0	1	0	0	0	0	0	0	0	0	0	0	0	0	0	0	0	0	0	0	0	0	0	0	0	0	1	43.56105	16.5228
2048	2002/074	0	0	0	0	0	0	0	0	0	0	1	0	0	0	0	0	0	0	0	0	0	0	0	0	0	0	0	0	0	0	0	0	0	0	0	1	43.56001667	16.51918333
2049	2002/075	0	1	0	0	0	0	0	0	0	0	0	0	0	0	0	1	0	0	0	0	0	0	0	0	0	0	0	0	0	0	0	0	0	0	0	1	43.56223333	16.52906667
2050	2002/076	0	1	0	0	0	0	0	0	0	0	1	0	0	0	0	1	0	0	0	0	0	0	0	0	0	0	0	0	0	0	0	0	0	0	0	1	43.44803333	16.69458333
2051	2002/077	0	0	0	0	0	0	0	0	0	0	0	0	0	0	0	0	0	0	0	0	0	0	0	0	0	0	0	0	0	0	0	0	0	0	0	1	43.42926667	16.86666667
2052	2002/078	0	1	0	0	0	0	0	0	0	0	0	0	0	0	0	1	0	0	0	0	0	0	0	0	0	0	0	0	0	0	0	0	0	0	0	1	43.43131667	16.88768333
2053	2002/079	0	1	0	0	0	0	0	0	0	0	1	0	0	0	0	1	0	0	0	0	0	0	0	0	0	0	0	0	0	0	0	0	0	0	0	1	43.40301667	16.89371667
2054	2002/080	0	0	0	0	0	0	0	0	0	0	0	0	0	0	0	1	0	0	0	0	0	0	0	0	0	0	0	0	0	0	0	0	0	0	0	1	43.29345	17.0519
2055	2002/081	0	0	0	0	0	0	0	0	0	0	0	0	0	0	0	0	0	0	0	0	0	0	0	0	0	0	0	0	0	0	0	0	0	0	0	1	43.25765	17.09398333
2056	2002/082	0	0	0	0	0	0	0	0	0	0	0	0	0	0	0	1	0	0	0	0	0	0	0	0	0	0	0	0	0	0	0	0	0	0	0	1	43.31243333	17.06221667
2057	2002/083	0	0	0	0	0	0	0	0	0	0	1	0	0	0	0	1	0	0	0	0	0	0	0	0	0	0	0	0	0	0	0	0	0	0	0	1	43.31596667	17.05396667
2058	2002/084	0	1	0	0	0	0	0	0	0	0	0	0	0	0	0	0	0	0	0	0	0	0	0	0	0	0	0	0	0	0	0	0	0	0	0	1	43.2586	17.10341667
2059	2002/085	0	1	0	0	0	0	0	0	0	0	0	0	0	0	0	1	0	0	0	0	0	0	0	0	0	0	0	0	0	0	0	0	0	0	0	1	43.23421667	17.1046
2060	2002/086	0	1	0	0	0	0	0	0	0	0	0	0	0	0	0	1	0	0	0	0	0	0	0	0	0	0	0	0	0	0	0	0	0	0	0	1	43.22788333	17.10986667
2061	2002/087	0	0	0	0	0	0	0	0	0	0	0	0	0	0	0	1	0	0	0	0	0	0	0	0	0	0	0	0	0	0	0	0	0	0	0	1	43.44278333	16.70153333
2062	2002/088	0	0	0	0	0	0	0	0	0	0	0	0	0	0	0	1	0	0	0	0	0	0	0	0	0	0	0	0	0	0	0	0	0	0	0	1	43.40358333	16.88048333
2063	2002/089	0	1	0	0	0	0	0	0	0	0	1	0	0	0	0	1	0	0	0	0	0	0	0	0	0	0	0	0	0	0	0	0	0	0	0	1	43.83178333	17.00788333
2064	2002/090	0	1	0	0	0	0	0	0	0	0	0	0	0	0	0	0	0	0	0	0	0	0	0	0	0	0	0	0	0	0	0	0	0	0	0	1	44.34876667	16.36025
2065	2002/091	0	0	0	1	0	0	0	0	0	0	0	0	0	0	0	0	0	0	0	0	0	0	0	0	0	0	0	0	0	0	0	0	0	0	0	1	44.17978333	19.87128333
2066	2004/071	0	0	0	0	0	0	0	0	0	0	0	0	0	0	0	0	0	1	0	0	0	0	0	0	0	0	0	0	0	0	0	0	0	0	0	1	45.334736	13.827767
2067	2004/072	0	0	0	1	0	0	0	0	0	1	0	0	0	0	0	0	0	0	0	0	0	0	0	0	0	0	0	0	0	0	0	0	0	0	0	1	45.368641	14.015313
2068	2004/073	0	0	0	0	0	0	0	0	0	0	0	0	0	0	0	0	0	0	0	0	0	0	0	0	0	0	0	0	0	0	0	0	0	0	0	1	45.348651	14.050464
2071	2005/025	0	0	0	0	0	0	1	0	0	0	0	0	0	0	0	0	0	0	0	0	0	0	0	0	0	0	0	0	0	0	0	0	0	0	0	1	46.56373333	23.6796
2072	2005/039	0	0	0	0	0	0	0	0	0	0	0	0	0	0	0	0	0	0	0	0	0	0	0	0	0	0	0	0	0	0	0	0	0	0	0	3	45.171	19.82625
2073	2005/040	0	0	0	0	0	0	0	0	0	0	0	0	1	0	0	0	0	0	0	0	0	0	0	0	0	0	0	0	0	0	0	0	0	0	0	1	44.16038333	19.88308333
2082	2006/096	0	0	0	0	0	0	0	0	0	0	0	0	0	0	0	0	0	0	0	0	0	0	0	0	0	0	0	0	0	0	0	0	0	0	0	1	43.9065	15.97451667
2087	2006/101	0	0	0	0	0	0	0	0	0	0	0	0	0	0	0	0	0	0	0	0	0	0	0	0	0	0	0	0	0	0	0	0	0	0	0	1	43.3814	16.9108
2100	2007/004	0	0	0	0	0	0	0	0	0	0	0	0	0	0	0	0	0	0	0	0	0	0	0	0	0	0	0	0	0	0	0	0	0	0	0	1	41.28333333	23.33323333
2102	2007/006	0	0	0	1	0	0	0	1	0	0	0	0	0	0	0	0	0	0	0	0	0	0	0	0	0	0	0	0	0	0	0	0	0	0	0	3	41.39915	23.50523333
2103	2007/007	0	0	0	0	0	0	0	0	0	0	0	0	0	0	0	0	0	0	0	0	0	0	0	0	0	0	0	0	0	0	0	0	0	0	0	3	41.39273333	23.4776
2109	2007/013	0	0	0	1	0	0	0	1	0	0	0	0	0	0	1	0	0	0	0	0	0	0	0	0	0	0	0	0	0	0	0	0	0	0	0	1	41.34541667	23.67438333
2111	2007/015	0	0	0	0	0	0	0	1	0	0	0	0	0	0	0	0	0	0	0	0	0	0	0	0	0	0	0	0	0	0	0	0	0	0	0	1	41.28466667	23.85523333
2112	2007/016	0	0	0	1	0	0	0	0	0	0	0	0	0	0	0	0	0	0	0	0	0	0	0	0	0	0	0	0	0	0	0	0	0	0	0	1	41.2211	23.89316667
2113	2007/017	0	0	0	0	0	0	0	1	0	0	0	0	0	0	0	0	0	0	0	0	0	0	0	0	0	0	0	0	0	0	0	0	0	0	0	2	41.28955	24.02718333
2114	2007/018	0	0	0	0	0	0	0	1	0	0	0	0	0	0	0	0	0	0	0	0	0	0	0	0	0	0	0	0	0	0	0	0	0	0	0	3	41.29303333	24.00703333
2117	2007/021	0	0	0	0	0	0	0	0	0	0	0	0	0	0	0	0	0	0	0	0	0	0	0	0	0	0	0	0	0	0	0	0	0	0	0	1	41.38876667	24.16796667
2119	2007/023	0	0	0	0	0	0	0	0	0	0	0	0	0	0	0	0	0	0	0	0	0	0	0	0	0	0	0	0	0	0	0	0	0	0	0	1	41.42803333	24.21821667
2127	2007/031	0	0	0	0	0	0	0	0	0	0	0	0	0	0	0	0	0	0	0	0	0	0	0	0	0	0	0	0	0	0	0	0	0	0	0	1	41.30973333	24.5066
2128	2007/032	0	0	0	0	0	0	0	0	0	0	0	0	0	0	0	0	0	0	0	0	0	0	0	0	0	0	0	0	0	0	0	0	0	0	0	2	41.1662	24.64311667
2129	2007/033	0	0	0	0	0	0	0	1	0	0	0	0	0	0	0	0	0	0	0	0	0	0	0	0	0	0	0	0	0	0	0	0	0	0	0	1	41.15873333	24.60915
2130	2007/034	0	0	0	0	0	0	0	1	0	0	0	0	0	0	0	0	0	0	0	0	0	0	0	0	0	0	0	0	0	0	0	0	0	0	0	1	41.03515	24.63235
2131	2007/035	0	0	0	1	0	0	0	1	0	0	0	0	0	0	0	0	0	0	0	0	0	0	0	0	0	0	0	0	0	0	0	0	0	0	0	1	41.02708333	24.63613333
2137	2007/041	0	0	0	0	0	0	0	1	0	0	0	0	0	0	0	0	0	0	0	0	0	0	0	0	0	0	0	0	0	0	0	0	0	0	0	1	41.09325	24.7713
2139	2007/043	0	0	0	0	0	0	0	0	0	0	0	0	0	0	0	0	0	0	0	0	0	0	0	0	0	0	0	0	0	0	0	0	0	0	0	1	41.20825	24.86253333
2142	2007/046	0	0	0	0	0	0	0	0	0	0	0	0	0	0	0	0	0	0	0	0	0	0	0	0	0	0	0	0	0	0	0	0	0	0	0	3	41.29185	24.82905
2143	2007/047	0	0	0	0	0	0	0	0	0	0	0	0	0	0	0	0	0	0	0	0	0	0	0	0	0	0	0	0	0	0	0	0	0	0	0	1	41.29141667	24.84611667
2145	2007/048	0	0	0	0	0	0	0	0	0	0	0	0	0	0	0	0	0	0	0	0	0	0	0	0	0	0	0	0	0	0	0	0	0	0	0	1	41.24546667	24.90455
2146	2007/049	0	0	0	0	0	0	0	0	0	0	0	0	0	0	0	0	0	0	0	0	0	0	0	0	0	0	0	0	0	0	0	0	0	0	0	1	41.24673333	24.9137
2149	2007/052	0	0	0	1	0	0	0	0	0	0	0	0	0	0	0	0	0	0	0	0	0	0	0	0	0	0	0	0	0	0	0	0	0	0	0	1	41.18175	25.28118333
2151	2007/054	0	0	0	0	0	0	0	0	0	0	0	0	0	0	0	0	0	0	0	0	0	0	0	0	0	0	0	0	0	0	0	0	0	0	0	1	41.1902	25.29586667
2152	2007/055	0	0	0	1	0	0	0	0	0	0	0	0	0	0	0	0	0	0	0	0	0	0	0	0	0	0	0	0	0	0	0	0	0	0	0	1	41.11408333	25.8388
2154	2007/057	0	0	0	1	0	0	0	0	0	0	0	0	0	0	0	0	0	0	0	0	0	0	0	0	0	0	0	0	0	0	0	0	0	0	0	1	40.93728333	25.9117
2156	2007/059	0	0	0	1	0	0	0	0	0	0	0	0	0	0	0	0	0	0	0	0	0	0	0	0	0	0	0	0	0	0	0	0	0	0	0	2	40.90133333	25.6058
2157	2007/060	0	0	0	1	0	0	0	0	0	0	0	0	0	0	0	0	0	0	0	0	0	0	0	0	0	0	0	0	0	0	0	0	0	0	0	1	40.92886667	25.5023
2160	2007/063	0	0	0	0	0	0	0	0	0	0	0	0	0	0	0	0	0	0	0	0	0	0	0	0	0	0	0	0	0	0	0	0	0	0	0	1	40.64511667	27.01278333
2173	2007/076	0	0	0	1	0	0	0	0	0	0	0	0	0	0	0	0	0	0	0	0	0	0	0	0	0	0	0	0	0	0	0	0	0	0	0	1	41.83931667	27.55618333
2175	2007/078	0	0	0	0	0	0	0	0	0	0	0	0	0	0	0	0	0	0	0	0	0	0	0	0	0	0	0	0	0	0	0	0	0	0	0	3	42.02935	27.47363333
2176	2007/079	0	0	0	0	0	0	0	0	0	0	0	0	0	0	0	0	0	0	0	0	0	0	0	0	0	0	0	0	0	0	0	0	0	0	0	1	42.08348333	27.4277
2188	2008/084	0	0	0	0	0	0	0	0	0	0	0	0	0	0	0	0	0	0	0	0	0	0	0	0	0	0	0	0	0	0	0	0	0	0	0	1	44.51944444	14.5075
2189	2008/085	0	0	0	0	0	0	0	0	0	0	0	0	0	0	0	0	0	0	0	0	0	0	0	0	0	0	0	0	0	0	0	0	0	0	0	1	44.69333333	14.39222222
2190	2008/086	0	0	0	0	0	0	0	0	0	0	0	0	0	0	0	0	0	0	0	0	0	0	0	0	0	0	0	0	0	0	0	0	0	0	0	1	44.908	14.35593333
2191	2008/087	0	0	0	0	0	0	0	0	0	0	0	0	0	0	0	0	0	0	0	0	0	0	0	0	0	0	0	0	0	0	0	0	0	0	0	1	44.88881667	14.33096667
2192	2008/088	0	0	0	0	0	0	0	0	0	0	0	0	0	0	0	0	0	0	0	0	0	0	0	0	0	0	0	0	0	0	0	0	0	0	0	1	44.98111667	14.45193333
2193	2008/089	0	0	0	0	0	0	0	0	0	0	0	0	0	0	0	0	0	0	0	0	0	0	0	0	0	0	0	0	0	0	0	0	0	0	0	1	45.21166667	14.55638889
2194	2008/101	0	0	0	0	0	0	0	0	0	0	0	0	0	0	0	0	0	0	0	0	0	0	0	0	0	0	0	0	0	0	0	0	0	0	0	1	43.45548333	16.92051667
2196	2008/103	0	1	0	0	0	0	0	0	0	0	0	0	0	0	0	1	0	0	0	0	0	0	0	0	0	0	0	0	0	0	0	0	0	0	0	1	43.44933333	17.2112
2197	2008/104	0	0	0	0	0	0	0	0	0	0	0	0	0	0	0	1	0	0	0	0	0	0	0	0	0	0	0	0	0	0	0	0	0	0	0	1	43.44995	17.21423333
2198	2008/105	0	0	0	0	1	0	0	0	0	0	0	0	0	0	0	0	0	0	0	0	0	0	0	0	0	0	0	0	0	0	0	0	0	0	0	1	43.06936667	17.41998333
2199	2008/106	0	0	0	0	1	0	0	0	0	0	0	0	0	0	0	0	0	0	0	0	0	0	0	0	0	0	0	0	0	0	0	0	0	0	0	1	42.78776667	17.89316667
2200	2008/107	0	0	0	0	1	0	0	0	0	0	0	0	0	0	0	0	0	0	0	0	0	0	0	0	0	0	0	0	0	0	0	0	0	0	0	1	42.70488333	17.99001667
2201	2008/108	0	0	0	0	0	0	0	0	0	0	0	0	0	0	0	0	0	0	0	0	0	0	0	0	0	0	0	0	0	0	0	0	0	0	0	2	42.64276667	18.1226
2202	2008/109	0	0	0	0	0	0	0	0	0	0	0	0	0	0	0	0	0	0	0	0	0	0	0	0	0	0	0	0	0	0	0	0	0	0	0	1	42.5346	18.37683333
2211	2009/095	0	0	0	0	0	0	0	0	0	0	0	0	1	0	0	0	0	0	0	0	0	0	0	0	0	0	0	0	0	0	0	0	0	0	0	1	44.71026667	16.63946667
2218	2009/102	0	0	0	0	0	0	0	0	0	0	0	0	1	0	0	0	0	0	0	0	0	0	0	0	0	0	0	0	0	0	0	0	0	0	0	1	44.61281667	17.98883333
2223	2009/107	0	0	0	0	0	0	0	0	0	0	1	0	0	0	0	0	0	0	0	0	0	0	0	0	0	0	0	0	0	0	0	0	0	0	0	1	43.83841667	17.59053333
2224	2009/108	0	0	0	0	0	0	0	0	0	0	1	0	0	0	0	0	0	0	0	0	0	0	0	0	0	0	0	0	0	0	0	0	0	0	0	1	43.81568333	17.61398333
2227	2009/111	0	0	0	0	0	0	0	0	0	0	0	0	0	0	0	0	0	0	0	0	0	0	0	0	0	0	0	0	0	0	0	0	0	0	0	1	43.65678333	17.94911667
2228	2009/112	0	0	0	0	0	0	0	0	0	0	1	0	0	0	0	0	0	0	0	0	0	0	0	0	0	0	0	0	0	0	0	0	0	0	0	1	43.67118333	17.98403333
2230	2009/114	0	0	0	0	0	0	0	0	0	0	0	0	0	0	0	0	0	0	0	0	0	0	0	0	0	0	0	0	0	0	0	0	0	0	0	1	43.80691667	18.18841667
2232	2009/116	0	0	0	0	0	0	0	0	0	0	0	0	0	0	0	0	0	0	0	0	0	0	0	0	0	0	0	0	0	0	0	0	0	0	0	3	43.95118333	18.0881
2235	2009/119	0	0	0	0	0	0	0	0	0	0	0	0	0	0	0	0	0	0	0	0	0	0	0	0	0	0	0	0	0	0	0	0	0	0	0	1	43.81911667	18.95545
2236	2009/120	0	0	0	0	0	0	0	0	0	0	0	0	0	0	0	0	0	0	0	0	0	0	0	0	0	0	0	0	0	0	0	0	0	0	0	1	43.78521667	19.01055
2237	2009/121	0	0	0	0	0	0	0	0	0	0	0	0	1	0	0	0	0	0	0	0	0	0	0	0	0	0	0	0	0	0	0	0	0	0	0	1	43.7395	19.00311667
2238	2009/122	0	0	0	0	0	0	0	0	0	0	0	0	1	0	0	0	0	0	0	0	0	0	0	0	0	0	0	0	0	0	0	0	0	0	0	1	43.75431667	19.41788333
2239	2009/130	0	0	0	1	0	0	0	0	0	0	0	0	0	0	1	0	0	0	0	0	0	0	0	0	0	0	0	0	0	0	0	0	0	0	0	1	44.02966667	22.22335
2245	2009/136	0	0	0	0	0	0	0	0	0	0	0	0	0	0	0	0	0	0	0	0	0	0	0	0	0	0	0	0	0	0	0	0	0	0	0	3	43.13558333	23.14086667
2247	2009/138	0	0	0	1	0	0	0	0	0	0	0	0	0	0	1	0	0	0	0	0	0	0	0	0	0	0	0	0	0	0	0	0	0	0	0	1	43.20453333	23.4936
2248	2009/139	0	0	0	1	0	0	0	0	0	0	0	0	0	0	1	0	0	0	0	0	0	0	0	0	0	0	0	0	0	0	0	0	0	0	0	1	43.1907	23.53028333
2250	2009/141	0	0	0	0	0	0	0	0	0	0	0	0	0	0	1	0	0	0	0	0	0	0	0	0	0	0	0	0	0	0	0	0	0	0	0	3	42.9735	24.1594
2251	2009/142	0	0	0	0	0	0	0	0	0	0	0	0	0	0	0	0	0	0	0	0	0	0	0	0	0	0	0	0	0	0	0	0	0	0	0	3	42.97863333	24.16246667
2255	2009/146	0	0	0	0	0	0	0	0	0	0	0	0	0	0	0	0	0	0	0	0	0	0	0	0	0	0	0	0	0	0	0	0	0	0	0	1	43.18118333	24.07088333
2261	2009/152	0	0	0	0	0	0	0	0	0	0	0	0	0	0	0	0	0	0	0	0	0	0	0	0	0	0	0	0	0	0	0	0	0	0	0	1	43.70436667	24.89595
2264	2009/155	0	0	0	1	0	0	0	0	0	0	0	0	0	0	1	0	0	0	0	0	0	0	0	0	0	0	0	0	0	0	0	0	0	0	0	1	43.15883333	22.58093333
2265	2009/156	0	0	0	0	0	0	0	0	0	0	0	0	0	0	1	0	0	0	0	0	0	0	0	0	0	0	0	0	0	0	0	0	0	0	0	1	43.18791667	22.59583333
2267	2009/158	0	0	0	0	0	0	0	0	0	0	0	0	0	0	0	0	0	0	0	0	0	0	0	0	0	0	0	0	0	0	0	0	0	0	0	1	43.32	22.17316667
2276	2011/010	0	0	0	1	0	0	0	1	0	0	0	0	0	0	0	0	0	0	0	0	0	0	0	0	0	0	0	0	0	0	0	0	0	0	0	1	39.95808333	23.92501667
2277	2011/011	0	0	0	0	0	0	0	1	0	0	0	0	0	0	0	0	0	0	0	0	0	0	0	0	0	0	0	0	0	0	0	0	0	0	0	2	40.37558333	23.16671667
2279	2012/213	0	0	0	0	1	0	0	0	0	0	1	0	0	0	0	0	0	0	0	0	0	0	0	0	0	0	0	0	0	0	0	0	0	0	0	1	42.9131	17.4228
2281	2014/002	0	0	0	0	0	0	0	0	0	0	0	0	0	0	0	0	0	0	0	0	0	0	0	0	0	0	0	0	0	0	0	0	0	0	0	2	41.0505	20.6441
2282	2014/003	0	0	0	0	0	0	0	0	0	0	0	0	0	0	0	0	0	0	1	0	0	0	0	0	0	0	0	0	0	0	1	0	0	0	0	2	41.0632	20.6253
2286	2014/007	0	0	0	0	0	0	0	0	0	0	0	0	0	0	0	0	0	0	0	0	0	0	0	0	0	0	0	0	0	0	0	0	0	0	1	1	41.0042	20.4343
2289	2014/010	0	0	0	0	0	0	0	0	0	0	0	0	0	0	0	0	0	0	0	0	0	0	0	0	0	0	0	0	0	0	0	1	0	0	0	1	41.2719	20.2839
2291	2014/012	0	0	0	0	0	0	0	0	0	0	0	0	0	0	0	0	0	0	1	0	0	0	0	0	0	0	0	0	0	0	0	0	0	0	0	2	41.2727	20.2703
2292	2014/013	0	0	0	0	0	0	0	0	0	0	0	0	0	0	0	0	0	0	1	0	0	0	0	0	0	0	0	0	0	0	0	0	0	0	0	1	41.2672	20.261
2293	2014/014	0	0	0	0	0	0	0	0	0	0	0	0	0	0	0	0	0	0	0	0	0	0	0	0	0	0	0	0	0	0	0	0	0	0	0	2	41.2652	20.2422
2294	2014/015	0	0	0	0	0	0	0	0	0	0	0	0	0	0	0	0	0	0	1	0	0	0	0	0	0	0	0	0	0	0	0	1	0	0	0	1	41.2802	20.21
2297	2014/018	0	0	0	0	0	0	0	0	0	0	0	0	0	0	0	0	0	0	0	0	0	0	0	0	0	0	0	0	0	0	0	0	0	0	1	1	41.2776	20.3375
2298	2014/019	0	0	0	0	0	0	0	0	0	0	0	0	0	0	0	0	0	0	1	0	0	0	0	0	0	0	0	0	0	0	0	1	0	0	0	1	41.2966	20.3741
2299	2014/020	0	0	0	0	0	0	0	0	0	0	0	0	0	0	0	0	0	0	1	0	0	0	0	0	0	0	0	0	0	0	0	1	0	0	0	1	41.3611	20.3967
2300	2014/021	0	0	0	0	0	0	0	0	0	0	0	0	0	0	0	0	0	0	1	0	0	0	0	0	0	0	0	0	0	0	0	1	0	0	0	1	41.3462	20.3904
2301	2014/022	0	0	0	0	0	0	0	0	0	0	0	0	0	0	0	0	0	0	0	0	0	0	0	0	0	0	0	0	0	0	0	1	0	0	0	1	41.3339	20.3958
2302	2014/023	0	0	0	0	0	0	0	0	0	0	0	0	0	0	0	0	0	1	1	0	0	0	0	0	0	0	0	0	0	0	0	0	0	0	0	3	41.3238	20.4224
2303	2014/024	0	0	0	0	0	0	0	0	0	0	0	0	0	0	0	0	0	0	0	0	0	0	0	0	0	0	0	0	0	0	0	1	0	0	0	1	41.325	20.4457
2304	2014/025	0	0	0	0	0	0	0	0	0	0	0	0	0	0	0	0	0	0	1	0	0	0	0	0	0	0	0	0	0	0	0	1	0	0	0	1	41.3646	20.4685
2305	2014/026	0	0	0	0	0	0	0	0	0	0	0	0	0	0	0	0	0	0	0	0	0	0	0	0	0	0	0	0	0	0	0	1	0	0	0	1	41.3792	20.4687
2307	2014/028	0	0	0	0	0	0	0	0	0	0	0	0	0	0	0	0	0	0	0	0	0	0	0	0	0	0	0	0	0	0	0	1	0	0	0	1	41.4199	20.4633
2308	2014/029	0	0	0	0	0	0	0	0	0	0	0	0	0	0	0	0	0	1	1	1	0	0	0	0	0	0	0	0	0	0	0	1	0	0	0	1	41.4404	20.5052
2312	2014/033	0	1	0	1	0	0	0	0	0	0	0	0	0	0	0	0	0	0	1	0	0	0	0	0	0	0	0	0	0	0	1	0	0	0	0	1	41.6097	20.4431
2314	2014/035	0	1	1	0	0	0	0	0	0	0	0	0	0	0	0	0	0	0	1	0	0	0	0	0	0	0	0	0	0	0	0	0	0	0	1	1	41.6308	20.2983
2315	2014/036	0	1	1	1	0	0	0	0	0	0	0	0	0	0	0	0	0	0	1	0	0	0	0	0	0	0	0	0	0	0	0	0	0	0	1	1	41.628	20.2741
2316	2014/037	0	0	0	0	0	0	0	0	0	0	0	0	0	0	0	0	0	0	0	0	0	0	0	0	0	0	0	0	0	0	0	0	0	0	1	1	41.6228	20.2886
2317	2014/038	0	0	0	0	0	0	0	0	0	0	0	0	0	0	0	0	0	1	0	0	0	0	0	0	0	0	0	0	0	0	0	0	0	0	0	1	41.6541	20.3129
2321	2014/042	0	0	0	0	0	0	0	0	0	0	0	0	0	0	0	0	0	0	0	0	0	0	0	0	0	0	0	0	0	0	0	2	0	0	0	1	41.514	20.316
2324	2014/045	0	1	1	0	0	0	0	0	0	0	0	0	0	0	0	0	0	0	0	0	0	0	0	0	0	0	0	0	0	0	1	1	0	0	0	1	41.4677	20.1048
2325	2014/046	0	1	0	0	0	0	0	0	0	0	0	0	0	0	0	0	0	0	0	0	0	0	0	0	0	0	0	0	0	0	1	0	0	0	0	1	41.4685	20.0904
2326	2014/047	0	0	0	0	0	0	0	0	0	0	0	0	0	0	0	0	0	0	1	0	0	0	0	0	0	0	0	0	0	0	0	0	0	1	0	3	41.4361	20.0485
2329	2014/050	0	1	0	0	1	0	0	0	0	0	1	0	0	0	0	0	0	1	0	0	0	0	1	0	0	0	0	0	0	0	0	0	0	0	0	1	41.6919	19.8318
2330	2014/051	0	1	0	1	0	0	0	0	0	0	1	0	0	0	0	0	0	0	0	0	0	0	0	0	0	0	0	0	0	0	0	0	0	0	0	1	41.7002	19.7052
2331	2014/052	0	0	0	0	0	0	0	0	0	0	1	0	0	0	0	0	0	0	1	0	0	0	0	0	0	0	0	0	0	0	0	0	0	0	0	3	41.6388	19.7286
2332	2014/053	0	0	0	0	1	0	0	0	0	0	1	0	0	0	0	0	0	0	0	0	0	0	0	0	0	0	0	0	0	0	0	0	0	0	0	1	41.6296	19.733

2334	2014/055	1	0	0	0	0	0	0	0	0	0	0	0	0	0	0	0	0	0	0	0	0	0	0	0	0	0	0	0	0	0	0	0	0	0	0	0	0	0	0	0	0	0	0	0	0	0	0	0	0	0	0	0	0	0	0	0	0	0	0	0	0	0	0	1	1	41.4412	19.8636
2335	2014/056	1	0	0	0	0	0	0	0	0	0	0	0	1	0	0	0	0	0	0	0	0	0	0	0	0	0	0	0	0	0	0	0	0	0	0	0	0	0	0	0	0	0	0	0	0	0	0	0	0	0	0	0	0	0	0	0	0	0	0	0	0	0	0	1	1	41.3948	19.9
2336	2014/057	1	0	0	0	0	1	0	0	1	1	0	0	1	1	1	0	0	0	1	0	0	0	1	0	0	0	0	1	1	0	1	0	0	1	0	0	0	0	0	0	0	0	0	0	0	0	0	1	0	0	0	0	0	0	0	0	0	0	0	0	0	0	0	1	1	41.2598	19.9676
2338	2014/058	1	0	0	0	0	0	0	0	0	1	0	0	0	0	0	0	0	0	0	0	0	0	0	0	0	0	0	0	0	1	0	0	1	0	0	0	0	0	0	0	0	0	0	0	0	0	0	1	0	0	0	0	0	0	0	0	0	0	0	0	0	0	0	0	1	41.1005	20.1249
2343	2014/063	1	0	0	0	0	0	0	0	1	1	0	0	1	0	1	0	0	1	1	0	0	0	0	0	1	1	0	0	0	1	0	0	1	0	0	0	0	0	1	0	0	1	0	0	0	0	0	0	0	0	0	0	0	0	0	0	0	0	0	0	0	0	0	0	1	43.85131809	19.82230472
2346	2014/066	0	0	0	0	0	0	0	0	0	0	0	0	1	0	0	0	0	0	0	0	0	0	0	0	0	0	0	0	0	1	0	0	0	0	0	0	0	0	0	0	0	0	0	0	0	0	0	0	0	0	0	0	0	0	0	0	0	0	0	0	0	0	0	0	1	42.39132207	19.73029716
2348	2014/068	1	0	0	0	0	1	0	0	1	1	0	0	1	0	0	0	0	0	1	0	0	0	0	0	0	0	0	1	1	0	1	0	0	1	0	0	0	0	0	0	0	0	0	0	0	0	0	0	0	0	0	0	0	0	0	0	0	0	0	0	0	0	0	0	1	42.13060297	19.71060824
2349	2014/069	1	0	0	0	0	1	0	0	0	0	0	0	1	0	0	0	0	0	1	0	1	0	0	0	0	0	0	0	1	0	1	0	0	1	0	0	0	0	0	0	0	0	0	0	0	0	0	0	0	0	0	0	0	0	0	0	0	0	0	0	0	0	0	0	1	42.12730469	19.71185186
2350	2014/070	0	0	0	0	0	1	0	0	0	0	0	0	1	0	0	0	0	0	0	0	0	0	0	0	0	0	0	1	0	0	0	0	0	0	0	0	0	0	0	0	0	0	0	0	0	0	0	0	0	0	0	0	0	0	0	0	0	0	0	0	0	0	0	0	1	41.85994897	20.118936
2351	2014/071	1	0	0	0	0	1	0	0	0	0	0	0	1	1	0	0	0	0	0	0	0	0	0	0	0	0	0	0	0	0	0	0	0	0	0	0	0	0	0	0	0	0	0	0	0	0	0	0	0	0	0	0	0	0	0	0	0	0	0	0	0	0	0	0	1	41.85607913	20.12075471
2352	2014/072	0	0	0	0	0	0	0	0	1	0	0	0	1	0	0	0	0	0	0	0	0	0	0	0	0	0	0	0	0	0	0	0	0	0	0	0	0	0	0	0	0	0	0	0	0	0	0	0	0	0	0	0	0	0	0	0	0	0	0	0	0	0	0	0	3	41.84553872	20.12990389
2353	2014/073	1	0	0	0	0	1	0	0	0	0	0	0	1	1	0	0	0	0	1	0	0	0	0	0	0	0	0	1	1	0	0	0	0	0	0	0	0	0	0	0	0	0	0	0	0	0	0	0	0	0	0	0	0	0	0	0	0	0	0	0	0	0	0	0	3	41.8524242	20.12344865
2354	2014/073B	0	0	0	0	0	0	0	0	0	0	0	0	0	0	0	0	0	0	0	0	0	0	0	0	0	0	0	0	0	0	0	0	0	0	0	0	0	0	0	0	0	0	0	0	0	0	0	0	0	0	0	0	0	0	0	0	0	0	0	0	0	0	0	0	1	41.85440015	20.12372442
2355	2014/074	0	0	0	0	0	0	0	0	0	0	0	0	0	0	0	0	0	0	0	0	0	0	0	0	0	0	0	0	0	0	0	0	0	0	0	0	0	0	0	0	0	0	0	0	0	0	0	0	0	0	0	0	0	0	0	0	0	0	0	0	0	0	0	0	1	41.84756345	20.11680348
2356	2014/075	0	0	0	0	0	0	0	0	0	0	0	0	0	0	0	0	0	0	0	0	0	0	0	0	0	0	0	0	0	0	0	0	0	0	0	0	0	0	0	0	0	0	0	0	0	0	0	0	0	0	0	0	0	0	0	0	0	0	0	0	0	0	0	0	1	41.84325482	20.11186
2357	2014/076	1	0	0	0	0	0	0	0	0	0	0	0	0	0	0	0	0	0	1	0	0	0	0	0	0	0	0	0	0	0	0	0	0	0	0	0	0	0	0	0	0	0	0	0	0	0	0	1	0	0	0	0	0	0	0	0	0	0	0	0	0	0	0	0	1	41.85947715	20.14933557
2358	2014/077	0	0	0	0	0	1	0	0	0	0	0	0	0	0	1	0	0	0	0	0	1	0	0	0	0	0	0	0	0	0	0	0	0	0	0	0	0	0	0	0	0	0	0	0	0	0	0	0	0	0	0	0	0	0	0	0	0	0	0	0	0	0	0	0	1	41.87470883	20.16569914
2359	2014/078	1	0	0	0	0	1	0	0	0	0	0	0	0	0	1	0	0	0	1	0	1	0	0	0	0	0	0	0	0	0	0	0	0	0	0	0	0	0	0	0	0	0	0	0	0	0	0	1	0	0	0	0	0	0	0	0	0	0	0	0	0	0	0	0	1	41.87930086	20.16633684
2360	2014/079	1	0	0	0	0	1	0	0	0	0	0	0	1	0	0	0	0	0	1	0	1	0	0	0	0	0	0	1	1	0	0	0	0	0	0	0	0	0	0	0	0	0	0	0	0	0	0	1	0	0	0	0	0	0	0	0	0	0	0	0	0	0	0	0	1	41.88691397	20.17148634
2361	2014/080	0	0	0	0	0	0	0	0	0	0	0	0	1	1	0	0	0	0	0	0	0	0	0	0	0	0	0	0	0	0	0	0	0	0	0	0	0	0	0	0	0	0	0	0	0	0	0	1	0	0	0	0	0	0	0	0	0	0	0	0	0	0	0	0	1	41.89182946	20.1873734
2362	2014/081	1	0	0	0	0	1	0	0	1	1	0	0	1	1	0	0	0	0	1	0	1	0	0	0	0	0	0	0	0	0	0	0	0	0	0	0	0	0	0	0	0	0	0	0	0	0	0	1	0	0	0	0	0	0	0	0	0	0	0	0	0	0	0	0	3	41.89971976	20.19479549
2363	2014/082	0	0	0	0	0	0	0	0	1	0	0	0	1	0	0	0	0	0	1	0	0	0	0	0	0	0	0	0	0	0	0	0	0	0	0	0	0	0	0	0	0	0	0	0	0	0	0	0	0	0	0	0	0	0	0	0	0	0	0	0	0	0	0	0	1	41.90106925	20.19663281
2364	2014/083	0	0	0	0	0	0	0	0	0	0	0	0	0	0	0	0	0	0	0	0	0	0	0	0	0	0	0	0	0	0	0	0	0	0	0	0	0	0	0	0	0	0	0	0	0	0	0	0	0	0	0	0	0	0	0	0	0	0	0	0	0	0	0	0	3	41.89704459	20.19823501
2365	2014/083B	0	0	0	0	0	0	0	0	0	0	0	0	0	0	0	0	0	0	0	0	0	0	0	0	0	0	0	0	0	0	0	0	0	0	0	0	0	0	0	0	0	0	0	0	0	0	0	1	0	0	0	0	0	0	0	0	0	0	0	0	0	0	0	0	3	41.89556535	20.1974347
2367	2014/085	0	0	0	0	0	0	0	0	0	0	0	0	0	0	0	0	0	0	1	0	0	0	0	0	0	0	0	0	0	0	0	0	0	0	0	0	0	0	0	0	0	0	0	0	0	0	0	0	0	0	0	0	0	0	0	0	0	0	0	0	0	0	0	0	1	41.88615172	20.18682674
2368	2014/086	0	0	0	0	0	0	0	0	0	0	0	0	0	0	0	0	0	0	0	0	0	0	0	0	0	0	0	0	0	0	0	0	0	0	0	0	0	0	0	0	0	0	0	0	0	0	0	0	0	0	0	0	0	0	0	0	0	0	0	0	0	0	0	0	1	41.88390059	20.1724915
2369	2014/087	0	0	0	0	0	0	0	0	0	0	0	0	0	0	0	0	0	0	1	0	0	0	0	0	0	0	0	0	0	0	0	0	0	0	0	0	0	0	0	0	0	0	0	0	0	0	0	1	0	0	0	0	0	0	0	0	0	0	0	0	0	0	0	0	1	41.9201	20.1989
2370	2014/089	0	0	0	0	0	0	0	0	0	0	0	0	0	0	0	0	0	0	1	0	0	0	0	0	0	0	0	0	0	0	0	0	0	0	0	0	0	0	0	0	0	0	0	0	0	0	0	0	0	0	0	0	0	0	0	0	0	0	0	0	0	0	0	0	1	41.92943822	20.18502354
2371	2014/090	0	0	0	0	0	1	0	0	0	0	0	0	0	1	0	0	0	0	0	0	0	0	0	0	0	0	0	0	1	0	0	0	0	0	0	0	0	0	1	0	0	0	0	0	0	0	0	0	1	0	0	0	0	0	0	0	0	0	0	0	0	0	0	0	3	41.88749274	20.17427199
2372	2014/091	1	0	0	0	0	1	0	0	0	0	0	0	1	1	0	0	0	0	1	0	0	0	0	0	0	0	0	0	0	1	0	0	1	0	0	0	0	0	0	0	0	1	0	0	0	0	0	0	0	0	0	0	0	0	0	0	0	0	0	0	0	0	0	0	3	42.55327636	20.24923646
2373	2014/092	1	0	0	0	0	1	0	0	0	0	0	0	1	0	0	0	0	0	1	0	0	0	0	0	0	0	0	0	0	0	0	0	0	0	0	0	0	0	0	1	0	0	0	0	0	0	0	0	0	0	0	0	0	0	0	0	0	0	0	0	0	0	0	0	1	42.43131464	20.14990035
2374	2014/093	0	0	0	0	0	0	0	0	0	0	0	0	0	0	0	0	0	0	0	0	0	0	0	0	0	0	0	0	0	0	0	0	0	0	0	0	0	0	0	1	0	0	0	0	0	0	0	0	0	0	0	0	0	0	0	0	0	0	0	0	0	0	0	0	3	42.45462262	20.15778135
2375	2014/094	1	0	0	0	0	1	0	0	0	0	0	0	1	0	0	0	0	0	1	0	0	0	0	0	0	0	0	0	0	1	0	0	1	0	0	0	0	0	1	0	0	1	0	0	0	0	0	0	0	1	0	0	0	0	0	0	0	0	0	0	0	0	0	0	3	42.4692	20.1554
2376	2014/095	1	0	0	0	0	0	0	0	0	0	0	0	1	0	0	0	0	0	1	0	1	0	1	0	0	0	0	0	0	1	0	0	1	0	0	0	0	0	0	0	0	1	0	0	0	0	0	0	0	1	0	0	0	0	0	0	0	0	0	0	0	0	0	0	3	42.474	20.152
2378	2014/097	1	0	0	0	0	0	0	0	1	0	0	0	1	1	1	0	0	0	1	0	0	0	1	0	0	0	0	1	0	0	0	0	0	0	0	0	0	0	0	0	0	0	0	0	0	1	1	1	0	0	0	0	0	0	0	0	0	0	0	0	1	0	0	0	1	40.70414077	19.94787167
2381	2014/100	1	0	0	0	0	0	0	0	0	0	0	0	1	0	0	0	0	0	0	0	0	0	0	0	0	0	0	0	0	0	0	1	0	0	1	0	0	0	0	0	0	0	0	0	0	0	1	0	0	0	0	0	0	0	0	0	0	0	0	0	0	0	0	0	1	40.11851538	20.0142897
2382	2014/101	1	0	0	0	0	0	0	0	0	0	0	0	0	0	0	0	0	0	0	0	0	0	0	0	0	0	0	0	0	0	0	1	0	0	1	0	0	0	0	0	0	0	0	0	0	0	1	0	0	0	0	0	0	0	0	0	0	0	0	0	0	0	0	0	1	40.11658821	20.00020718
2383	2014/102	1	0	0	0	0	0	0	0	0	0	0	0	0	0	1	0	0	0	0	0	0	0	1	0	0	0	0	0	0	0	0	0	0	0	0	0	0	0	0	0	0	0	0	0	0	0	1	0	0	0	0	0	0	0	0	0	0	0	0	0	0	0	0	0	1	40.0949	19.991734
2384	2014/103	1	0	0	0	0	1	0	0	1	0	0	0	1	0	1	1	0	0	0	0	0	0	0	0	0	0	0	0	0	0	0	1	0	0	1	0	0	0	0	0	0	0	0	0	0	0	1	1	0	0	0	0	0	0	0	0	0	0	0	0	0	0	0	0	1	40.08816979	19.96313788
2385	2014/104	1	0	0	0	0	1	0	0	0	0	0	0	1	0	1	0	0	0	1	0	0	0	1	0	0	0	0	0	1	0	0	0	0	0	0	0	0	0	0	0	0	0	0	0	0	0	0	0	0	0	0	0	0	1	0	0	0	0	0	0	0	0	0	0	1	40.27575167	20.09470141
2386	2014/105	0	0	0	0	0	0	0	0	1	1	0	0	1	0	0	0	0	0	1	0	0	0	0	0	0	0	0	0	0	0	0	1	0	0	1	0	0	0	0	0	0	0	0	0	0	0	0	0	0	0	0	0	0	0	0	0	0	0	0	0	0	0	0	0	1	40.258292	20.07898903
2388	2014/107	1	0	0	0	0	0	0	0	0	0	0	0	0	0	1	0	0	0	1	0	0	0	1	0	0	0	0	1	0	0	0	1	0	0	1	0	0	0	0	0	0	0	0	0	0	0	0	1	0	0	0	0	0	1	0	0	0	0	0	0	0	0	0	0	1	40.29608474	20.10760124
2389	2014/108	1	0	0	0	0	0	0	0	1	0	0	0	1	0	1	0	0	0	1	0	0	0	1	0	0	0	0	1	0	0	0	0	0	0	0	0	0	0	0	0	0	0	0	0	0	0	0	1	0	0	0	0	0	1	0	0	0	0	0	0	0	0	0	0	3	40.12257448	20.48048562
2391	2014/110	0	0	0	0	0	0	0	0	0	0	0	0	0	0	0	0	0	0	0	0	0	0	0	0	0	0	0	0	0	0	0	0	0	0	0	0	0	0	0	0	0	0	0	0	0	0	0	0	0	0	0	0	1	0	0	0	0	0	0	0	0	0	0	0	1	40.24180354	20.64135958
2394	2014/113	1	0	0	0	0	1	0	0	1	0	0	0	1	0	0	0	0	0	1	0	0	0	1	0	0	0	0	0	0	0	1	0	0	1	0	0	0	0	0	0	0	0	0	0	0	0	0	1	0	0	0	0	0	0	0	0	0	0	0	0	0	0	0	0	1	40.73013313	20.50389863
2395	2014/114	1	0	0	0	0	0	0	0	0	0	0	0	1	0	0	1	0	0	0	0	0	0	0	0	0	0	0	0	1	0	1	0	0	1	0	0	0	0	0	0	0	0	0	0	0	0	0	1	0	0	0	0	0	0	0	1	0	0	0	0	0	0	0	1	3	40.74798541	20.52191997
2396	2014/115	1	0	0	0	0	1	0	0	0	0	0	0	0	0	0	0	0	0	1	0	0	0	1	0	0	0	0	0	0	0	1	0	0	1	0	0	0	0	0	0	0	0	0	0	0	0	0	0	0	0	0	0	0	0	0	1	0	0	0	0	0	0	0	0	1	40.74616964	20.51225656
2398	2014/117	1	0	0	0	0	0	0	0	0	0	0	0	1	0	1	0	0	0	1	0	0	0	0	0	1	0	0	0	0	0	1	0	0	1	0	0	0	0	0	0	0	0	0	0	1	0	0	0	0	0	0	0	0	0	0	0	0	0	0	0	0	0	0	0	1	41.00030777	20.24742781
2400	2014/119	0	0	0	0	0	1	0	0	1	0	0	0	0	0	0	1	0	0	0	0	0	0	0	0	0	0	0	0	0	1	0	0	1	0	0	0	0	0	0	0	0	0	0	0	0	0	0	0	0	0	0	0	0	0	1	0	0	0	0	0	0	0	0	0	1	40.96267	20.81707
2402	2014/121	0	0	1	0	0	1	0	0	1	0	0	0	0	0	0	1	0	0	1	0	0	0	0	0	0	1	0	0	0	1	0	0	1	0	0	0	0	0	0	0	0	0	0	0	0	0	0	0	0	0	0	0	0	0	1	0	0	0	0	0	0	0	0	0	1	40.914	20.7406
2403	2014/122	0	0	0	0	0	0	0	0	0	0	0	0	0	0	0	0	0	0	0	0	0	0	0	0	0	0	0	0	0	0	0	0	0	0	0	0	0	0	0	0	0	0	0	0	0	0	0	0	0	0	0	0	0	0	1	0	0	0	0	0	0	0	0	0	1	41.1147	20.7876
2404	BPP2014/5	0	0	0	0	0	0	0	0	0	0	0	0	0	0	0	0	0	0	0	0	0	0	0	0	0	0	0	1	0	0	0	0	0	0	0	0	0	0	0	1	0	0	0	0	0	0	0	0	0	0	0	0	0	0	0	0	0	0	1	0	0	0	0	0	2	41.85691	19.50439
2405	BPP2014/14A	0	0	0	0	0	0	0	0	0	0	0	0	0	0	0	0	0	0	0	0	0	0	0	0	0	0	0	1	0	0	0	0	0	0	0	0	0	0	0	0	0	0	0	0	0	0	0	0	0	0	0	0	0	0	0	0	0	0	0	0	0	0	0	0	2	40.42874	19.33526
2406	BPP2014/19	1	0	0	0	0	0	0	0	0	0	0	0	0	0	0	0	0	0	0	0	0	0	0	0	0	0	0	0	0	0	0	0	0	0	0	0	0	0	0	0	0	0	0	0	0	0	0	0	0	0	0	0	0	0	0	0	0	0	0	0	0	0	0	0	2	39.7175	20.01643
2407	BPP2014/23D	1	0	0	0	0	0	0	0	0	0	0	0	0	0	0	0	0	0	0	0	0	0	0	0	0	0	0	0	0	0	0	1	0	0	1	0	0	0	0	0	0	0	0	0	0	0	1	0	0	0	0	0	0	0	0	0	0	0	0	0	0	0	0	0	2	39.90273	20.17079
2408	BPP2014/25	1	0	0	0	0	0	0	0	0	0	0	0	0	0	0	0	0	0	1	0	0	0	0	0	0	0	0	0	0	0	0	0	0	0	0	0	0	0	0	0	0	0	0	0	0	0	0	0	0	0	0	0	0	0	0	0	0	0	0	0	0	0	0	0	2	40.24988	20.11788
2409	BPP2014/25B	1	0	0	0	0	0	0	0	0	0	0	0	0	0	0	0	0	0	0	0	0	0	0	0	0	0	0	0	0	0	0	1	0	0	1	0	0	0	0	0	0	0	0	0	0	0	0	0	0	0	0	0	0	0	0	0	0	0	0	0	0	0	0	0	1	40.24512	20.11061
2410	BPP2014/26	0	0	0	0	0	0	0	0	0	0	0	0	0	0	0	0	0	0	0	0	0	0	0	0	0	0	0	0	0	0	0	0	0	0	0	0	0	0	0	0	0	0	0	0	0	0	0	1	0	0	0	0	0	0	0	0	0	0	0	0	1	0	0	0	1	40.49264	19.72609
2412	2014/123	0	0	0	0	0	0	0	0	0	0	0	0	0	0	1	0	0	0	0	0	0	0	0	0	0	0	0	0	0	1	0	0	1	0	0	0	0	0	1	0	0	0	0	0	0	0	0	0	0	0	0	0	0	0	0	0	0	0	0	0	0	0	0	0	3	41.65089098	20.71347621
2413	2014/124	0	0	0	0	0	1	0	0	1	1	0	0	0	0	1	1	0	0	1	0	0	0	0	0	0	0	0	0	0	0	0	0	0	0	0	0	0	0	0	0	0	0	0	0	0	0	0	0	0	0	0	0	0	0	0	0	0	0	0	0	0	0	0	1	3	41.5942097	20.67159771
2414	2014/125	0	0	0	0	0	1	0	0	1	0	0	0	1	0	1	1	0	0	0	0	0	0	0	0	0	0	0	0	0	0	0	0	0	0	0	0	0	0	0	0	0	0	0	0	0	0	0	0	1	0	0	0	0	0	0	0	0	0	0	0	0	0	0	1	1	41.59357175	20.65752852
2415	2014/126	0	0	0	0	0	1	0	0	0	1	0	0	0	0	1	1	0	0	0	0	0	0	0	0	0	0	0	0	0	1	0	0	1	0	0	0	0	0	0	0	0	0	0	0	0	0	0	0	0	0	0	0	0	0	0	0	0	0	0	0	0	0	0	1	1	41.62395129	20.67683933
2416	2014/127	0	0	0	0	0	0	0	0	1	0	0	0	0	0	0	1	0	0	1	0	0	0	0	0	0	0	0	0	0	1	0	0	1	0	0	0	0	0	0	0	0	0	0	0	0	0	0	0	0	0	0	0	0	0	0	0	0	0	0	0	0	0	0	0	1	41.64226826	20.69765336
2417	2014/128	0	0	0	0	0	0	0	0	1	1	0	0	1	0	0	0	0	0	0	0	1	0	0	0	0	0	0	0	0	1	0	0	1	0	0	0	0	0	0	0	0	0	0	0	0	0	0	0	0	0	0	0	0	0	0	0	0	0	0	0	0	0	0	0	1	41.64685031	20.70980569
2418	2014/129	0	0	0	0	0	1	0	0	1	0	0	0	1	0	1	0	0	0	1	0	0	0	1	0	0	1	0	0	0	1	0	0	1	0	0	0	0	0	1	0	0	0	0	0	0	0	0	0	1	0	0	0	0	0	0	0	0	0	0	0	0	0	0	0	1	41.71171619	20.71232286
2419	2014/130	0	0	0	0	0	1	0	0	1	0	0	0	1	0	0	0	0	0	1	0	0	0	0	0	0	0	0	0	0	1	0	0	1	0	0	0	0	0	0	0	0	0	0	0	0	0	0	0	0	0	0	0	0	0	0	0	0	0	0	0	0	0	0	1	1	41.72026657	20.66822106
2420	2014/131	0	0	0	0	0	0	0	0	0	0	0	0	0	0	0	0	0	0	1	0	0	0	0	0	0	0	0	0	0	0	0	0	0	0	0	0	0	0	1	0	0	0	0	0	0	0	0	0	0	0	0	0	0	0	0	0	0	0	0	0	0	0	0	1	1	41.76549599	20.66659589
2421	2014/132	0	0	0	0	0	1	0	0	1	0	0	0	0	0	1	0	0	0	0	0	0	0	0	0	0	0	0	0	0	0	0	0	0	0	0	0	0	0	1	0	0	0	0	0	0	0	0	0	0	0	0	0	0	0	0	0	0	0	0	0	0	0	0	1	1	41.7102	20.6564
2422	2014/133	0	0	0	0	0	1	0	0	1	0	0	0	1	0	1	0	0	0	1	0	0	0	0	0	0	0	0	0	0	1	0	0	1	0	0	0	0	0	1	0	0	0	0	0	0	0	0	0	1	0	0	0	0	0	0	0	0	0	0	0	0	0	0	1	1	41.70793352	20.6500164
2423	2014/134	0	0	0	0	0	0	0	0	1	0	0	0	1	0	0	0	0	0	0	0	0	0	0	0	0	0	0	0	0	1	0	0	1	0	0	0	0	0	0	0	0	0	0	0	0	0	0	0	0	0	0	0	0	0	0	0	0	0	0	0	0	0	1	0	1	41.69940996	20.64784809
2424	2014/135	0	0	0	0	0	0	0	0	0	0	0	0	0	0	0	0	0	0	0	0	0	0	0	0	0	0	0	0	0	0	0	0	0	0	0	0	0	0	0	0	0	0	0	0	0	0	0	0	0	0	0	0	0	0	0	0	0	0	0	1	0	0	0	0	2	41.49481357	20.50522431
2425	2014/136	0	0	0	0	0	0	0	0	0	0	0	0	0	0	0	0	0	0	0	0	0	0	0	0	0	0	0	0	0	0	0	0	0	0	0	0	0	0	0	0	0	0	0	0	0	0	0	1	0	0	0	0	0	0	0	0	0	0	0	0	0	0	0	0	1	41.44393735	20.55075321
2426	2014/137	0	0	0	0	0	0	0	0	0	0	0	0	0	0	0	0	0	0	0	0	0	0	0	0	0	0	0	0	0	0	0	0	0	0	0	0	0	0	0	0	0	0	0	0	0	0	0	0	0	0	0	0	0	0	0	0	0	0	0	0	0	0	0	0	1	41.2997583	20.6200454
2427	2014/138	0	0	0	0	0	1	0	0	1	1	0	0	1	0	1	0	0	0	0	0	0	0	0	0	0	0	0	0	0	0	0	0	0	0	0	0	0	0	0	0	0	0	0	0	0	0	1	1	0	0	0	0	0	0	0	0	0	0	0	0	0	0	1	0	1	41.32985277	20.63693494
2428	2014/139	1	0	0	0	0	1	0	0	1	0	0	0	1	1	0	0	0	0	0	0	0	0	0	0	0	0	0	0	0	1	0	0	1	0	0	0	0	0	0	0	0	0	0	0	0	0	0	1	0	0	0	0	0	0	0	0	0	0	0	0	0	0	1	0	1	41.3397	20.6273
2430	2014/141	1	0	0	0	0	1	0	0	1	1	0	0	1	1	1	0	0	0	1	0	0	0	0	0	0	0	0	0	0	1	0	0	1	0	0	0	0	0	0	0	0	0	0	0	0	0	0	1	0	0	0	0	0	0	0	0	0	0	0	0	0	0	1	0	1	41.35451392	20.61244343
2431	2014/142	0	0	0	0	0	1	0	0	0	0	0	0	1	0	0	0	0	0	0	0	0	0	0	0	0	0	0	0	0	0	0	0	0	0	0	0	0	0	0	0	0	0	0	0	0	0	1	1	0	0	0	0	0	0	0	0	0	0	0	0	1	0	0	0	1	41.36230314	20.60361788
2432	2014/143	1	0	0	0	0	1	0	0	0	0	0	0	0	0	0	0	0	0	1	0	0	0	0	0	0	0	0	0	0	0	0	0	0	0	0	0	0	0	0	0	0	0	0	0	0	0	0	0	0	0	0	0	0	0	0	0	0	0	0	1	0	0	0	0	1	41.36378967	20.60489343
2433	2014/144	1	0	0	0	0	1	0	0	1	1	0	0	1	0	1	0	0	0	0	0	0	0	0	0	0	0	0	0	0	0	0	0	0	0	0	0	0	0	0	0	0	0	0	0	0	0	0	0	0	0	0	0	0	0	0	0	0	0	0	1	0	0	0	0	1	41.36664086	20.60492738
2434	2014/145	0	0	0	0	0	0	0	0	0	0	0	0	0	0	0	0	0	0	0	0	0	0	0	0	0	0	0	0	0	0	0	0	0	0	0	0	0	0	0	0	0	0	0	0	0	0	0	0	0	0	0	0	0	0	0	0	0	0	0	1	0	0	0	0	1	41.36720781	20.60463049
2435	2014/146	1	0	0	0	0	1	0	0	1	1	0	0	1	0	1	1	0	0	1	0	0	0	0	0	1	0	0	0	0	1	0	0	1	0	0	0	0	0	0	0	0	0	0	0	0	0	0	0	0	0	0	0	0	0	0	0	0	0	0	1	0	0	0	0	1	41.36945827	20.60207795
2436	2014/147	0	0	0	0	0	0	0	0	0	0	0	0	1	0	0	0	0	0	0	0	0	0	0	0	0	0	0	0	0	1	0	0	1	0	0	0	0	0	0	0	0	0	0	0	0	0	0	0	0	0	0	0	0	0	0	0	0	0	0	1	0	0	0	0	1	41.37047214	20.59794609
2437	2014/148	0	0	0	0	0	0	0	0	1	0	0	0	0	0	0	0	0	0	0	0	0	0	0	0	0	0	0	0	0	1	0	0	0	0	0	0	0	0	0	0	0	0	0	0	0	0	0	0	0	0	0	0	0	0	0	0	0	0	0	1	0	0	0	0	1	41.37134554	20.59709994
2438	2014/149	0	0	0	0	0	0	0	0	0	0	0	0	0	0	0	0	0	0	0	0	0	0	0	0	0	0	0	0	0	0	0	0	0	0	0	0	0	0	0	0	0	0	0	0	0	0	0	0	0	0	0	0	0	0	0	0	0	0	0	0	0	0	0	0	1	41.40042353	20.58493369
2439	2014/150	1	0	0	0	0	1	0	0	0	0	0	0	0	0	0	0	0	0	0	0	0	0	0	0	0	0	0	0	0	0	0	0	0	0	0	0	0	0	0	0	0	0	0	0	0	0	0	0	0	0	0	0	0	0	0	0	0	0	0	1	0	0	0	0	1	41.39784526	20.58994733
2440	2014/151	0	0	0	0	0	0	0	0	0	0	0	0	1	0	1	0	0	0	0	0	0	0	0	0	0	0	0	0	0	0	0	0	0	0	0	0	0	0	0	0	0	0	0	0	0	0	0	0	0	0	0	0	0	0	0	0	0	0	0	1	0	0	0	0	1	41.38158244	20.58807707
2441	2014/152	0	0	0	0	0	0	0	0	1	1	0	0	1	1	0	1	0	0	1	0	0	0	1	0	1	0	0	0	0	1	0	0	1	0	0	0	0	0	0	0	0	0	0	0	0	0	0	0	0	0	0	0	0	0	0	0	0	0	0	0	0	0	0	1	1	41.22019627	20.91150999
2443	2014/154	0	0	0	0	0	0	0	0	0	0	0	0	0	0	0	0	0	0	0	0	0	0	0	0	0	0	0	0	0	1	0	0	1	0	0	0	0	0	0	0	0	0	0	0	0	0	0	1	0	0	0	0	0	0	0	0	0	0	0	0	1	0	0	0	1	41.084319	20.79733053
2444	2014/155	0	0	0	0	0	1	0	0	0	0	0	0	0	0	1	1	0	0	1	0	0	0	0	0	0	0	0	0	0	0	0	0	0	0	0	0	0	0	0	0	0	0	0	0	0	0	0	0	0	0	0	0	0	0	1	0	0	0	0	0	0	0	0	0	2	40.99335699	20.85693542
2445	2014/156	0	0	0	0	0	1	0	0	1	0	0	0	0	1	1	1	0	0	0	0	0	0	0	0	0	0	0	0	0	0	0	0	0	0	0	0	0	0	0	0	0	0	0	0	0	0	0	0	0	0	0	0	0	0	1	0	0	0	0	0	0	0	0	0	2	41.0026045	20.85337671
2447	2014/158	0	0	0	0	0	1	0	0	0	0	0	0	0	0	1	0	0	0	0	0	0	0	0	0	0	0	0	0	0	0	0	0	0	0	0	0	0	0	0	0	0	0	0	0	0	0	0	0	0	0	0	0	0	0	1	0	0	0	0	0	0	0	0	0	2	41.00331821	20.84876633
2448	2014/159	1	0	0	0	0	1	0	0	1	0	0	0	0	0	1	1	0	0	0	0	1	0	0	0	0	0	0	0	0	0	0	0	0	0	0	0	0	0	0	0	0	0	0	0	0	0	0	0	0	0	0	0	0	0	1	0	0	0	0	0	0	0	0	0	2	41.00371292	20.84695542
2449	2014/160	1	0	0	0	0	0	0	0	0	0	0	0	1	0	0	0	0	0	0	0	1	0	0	0	0	0	0	0	0	1	0	0	1	0	0	0	0	0	0	0	0	0	0	0	0	0	0	0	0	0	0	0	0	0	1	0	0	0	0	0	0	0	0	0	2	41.00456486	20.84318834
2450	2014/161	1	0	0	0	0	1	0	0	0	0	0	0	0	0	0	0	0	0	0	0	1	0	0	0	0	0	0	0	0	1	0	0	1	0	0	0	0	0	0	0	0	0	0	0	0	0	0	0	0	0	0	0	0	0	1	0	0	0	0	0	0	0	0	0	2	41.00506509	20.84616811
2451	2014/162	1	0	0	0	0	1	0	0	1	0	0	0	1	1	1	0	0	0	1	0	0	0	0	0	0	0	0	0	0	1	0	0	1	0	0	0	0	0	0	0	0	0	0	0	0	0	0	1	0	0	0	0	0	0	1	0	0	0	0	0	0	0	0	0	1	40.95738078	20.77780623
2452	2014/163	1	0	0	0	0	1	0	0	0	0	0	0	0	1	0	0	0	0	1	0	0	0	0	0	0	0	0	0	0	1	0	0	1	0	0	0	0	0	0	0	0	0	0	0	0	0	0	1	0	0	0	0	0	0	1	0	0	0	0	0	1	0	0	0	2	40.9937315	20.79917514
2453	2014/164	1	0	0	0	0	1	0	0	1	0	0	0	0	1	1	0	0	0	0	0	0	0	0	0	0	0	0	0	0	0	0	0	0	0	0	0	0	0	0	0	0	0	0	0	0	0	0	1	0	0	0	0	0	0	0	0	0	0	0	0	1	0	0	0	1	41.00917499	20.80557724
2454	2014/165	1	0	0	0	0	1	0	0	0	0	0	0	1	1	1	0	0	0	1	0	0	0	0	0	0	0	0	0	0	0	0	0	0	0	0	0	0	0	0	0	0	0	0	0	0	0	0	1	0	0	0	0	0	0	1	0	0	0	0	0	0	0	0	0	1	41.03454868	20.81052901
2455	2014/166	0	0	0	0	0	1	0	0	0	0	0	0	0	0	1	1	0	0	1	0	0	0	0	0	0	0	0	0	0	1	0	0	1	0	0	0	0	0	0	0	0	0	0	0	0	0	0	1	1	0	0	0	0	0	1	0	0	0	0	0	0	0	0	0	1	40.96742992	20.81490646
2456	2014/167	0	0	0	0	0	1	0	0	0	0	0	0	1	1	1	0	0	0	0	0	0	0	0	0	0	0	0	0	0	1	0	0	1	0	0	0	0	0	0	0	0	0	0	0	0	0	0	0	0	0	0	0	0	0	1	0	0	0	0	0	0	0	0	0	1	40.89242723	20.9393851
2457	2014/168	0	0	0	0	0	0	0	0	0	0	0	0	1	0	0	0	0	0	0	0	0	0	0	0	0	0	0	0	0	0	0	0	0	0	0	0	0	0	0	0	0	0	0	0	0	0	0	0	0	0	0	0	0	0	0	1	0	0	0	0	0	0	0	0	1	40.85800904	20.94259998
2458	2014/169	0	0	0	0	0	0	0	0	0	0	0	0	0	1	0	0	0	0	0	0	0	0	0	0	0	0	0	0	0	0	0	0	0	0	0	0	0	0	0	0	0	0	0	0	0	0	0	1	0	0	0	0	0	0	0	0	0	0	0	0	0	0	0	0	2	40.81220319	20.90447774
2459	2014/170	0	0	0	0	0	0	0	0	0	0	0	0	1	0	1	1	0	0	1	0	0	0	1	0	0	0	0	0	0	0	0	0	0	0	0	0	0	0	0	0	0	0	0	0	0	0	0	0	0	0	0	0	0	0	1	0	0	0	0	0	0	0	0	0	1	40.6344356	21.09154883
2460	2014/171	1	0	0	0	0	1	0	0	0	0	0	0	1	1	1	0	0	0	1	0	0	0	0	0	0	0	0	0	0	1	0	0	1	0	0	0	0	0	0	0	0	0	0	0	0	0	0	0	0	0	0	0	0	0	1	0	0	0	0	0	0	0	0	0	1	40.52332627	21.27467615
2462	2014/173	0	0	0	0	0	0	0	0	0	0	0	0	0	0	0	0	0	0	0	0	0	0	0	0	0	0	0	0	0	0	0	0	0	0	0	0	0	0	0	0	0	0	0	0	0	0	0	0	0	0	0	0	0	0	1	0	0	0	0	0	0	0	0	0	1	40.80872211	20.99792314
2463	2014/174	0	0	0	0	0	1	0	0	0	0	0	0	0	1	1	0	0	0	0	0	0	0	0	0	0	0	0	0	0	0	0	0	0	0	0	0	0	0	0	0	0	0	0	0	0	0	0	1	0	0	0	0	0	0	0	0	0	0	0	0	0	0	0	0	1	40.81996283	21.05221474
2464	2014/175	0	0	0	0	0	1	0	0	1	0	0	0	1	0	1	0	0	0	0	0	0	0	0	0	0	0	0	0	0	0	0	0	0	0	0	0	0	0	0	0	0	0	0	0	0	0	0	0	0	0	0	0	0	0	0	1	0	0	0	0	0	0	0	0	1	40.79977711	21.07147157
2465	2014/176	0	0	0	0	0	1	0	0	1	0	0	0	1	1	0	0	0	0	1	0	0	0	0	0	0	0	0	0	1	0	0	0	0	0	0	0	0	0	0	0	0	0	0	0	0	0	0	1	0	0	0	0	0	0	0	1	0	0	0	0	0	0	0	0	1	40.71921872	21.03408761
2466	2014/177	0	0	0	0	0	1	0	0	1	0	0	0	1	1	1	0	0	0	0	0	0	0	0	0	0	0	0	0	0	1	0	0	1	0	0	0	0	0	0	0	0	0	0	0	0	0	0	0	0	0	0	0	0	0	1	0	0	0	0	0	0	0	0	0	1	40.81053067	21.07019073
2467	2014/178	0	0	0	0	0	1	0	0	1	0	0	0	0	1	1	0	0	0	0	0	0	0	0	0	0	0	0	0	0	0	0	0	0	0	0	0	0	0	0	0	0	0	0	0	0	0	0	0	0	0	0	0	0	0	1	0	0	0	0	0	0	0	0	0	1	40.65323889	21.17894132
2468	2014/179	0	0	0	0	0	1	0	0	0	0	0	0	0	0	1	0	0	0	0	0	0	0	0	0	0	0	0	0	0	0	0	0	0	0	0	0	0	0	0	0	0	0	0	0	0	0	0	0	0	0	0	0	0	0	0	0	0	0	0	0	0	0	0	0	2	41.37982568	21.74413774
2473	2015/004	1	0	0	0	0	0	0	0	0	0	0	0	1	0	1	0	0	0	0	0	0	0	0	0	0	0	0	0	1	0	0	0	0	0	0	0	0	0	0	1	0	0	0	0	0	0	0	0	0	0	0	0	0	0	0	0	0	0	0	0	0	0	0	0	1	42.643544	19.377819
2474	2015/005	1	0	0	0	0	0	0	0	0	0	0	0	0	0	0	0	0	0	0	0	0	0	0	0	0	0	0	0	1	0	0	0	0	0	0	0	0	0	0	0	0	0	0	0	0	0	0	0	0	0	0	0	0	0	0	0	0	0	0	0	0	0	0	0	1	42.62853	19.372128
2475	2015/006	1	0	0	0	0	0	0	0	0	0	0	0	0	0	1	0	0	0	0	0	0	0	0	0	0	0	0	0	1	0	0	0	0	0	0	0	0	0	0	1	0	0	0	0	0	0	0	0	0	0	0	0	0	0	0	0	1	0	0	0	0	0	0	0	1	42.37066	19.313889
2476	2015/007	0	0	0	0	0	0	0	0	0	0	0	0	1	0	0	0	0	0	0	0	0	0	0	0	0	0	0	0	1	0	0	0	0	0	0	0	0	0	0	1	0	0	0	0	0	0	0	0	0	0	0	0	0	0	0	0	1	0	0	0	0	0	0	0	1	42.317159	19.156679
2477	2015/008	0	0	0	0	0	0	0	0	0	0	0	0	0	0	0	0	0	0	0	0	0	0	0	0	0	0	0	0	0	0	0	0	0	0	0	0	0	0	0	1	0	0	0	0	0	0	0	0	0	0	0	0	0	0	0	0	0	0	0	0	0	0	0	0	1	42.314265	19.185085
2478	2015/009	0	0	0	0	0	0	0	0	0	0	0	0	0	0	0	0	0	0	0	0	0	0	0	0	0	0	0	0	0	0	0	0	0	0	0	0	0	0	0	1	0	0	0	0	0	0	0	0	0	0	0	0	0	0	0	0	0	0	0	0	0	0	0	0	1	42.280996	19.137376
2479	2015/010	0	0	0	0	0	0	0	0	0	0	0	0	0	0	1	0	0	0	0	0	0	0	0	0	0	0	0	0	0	0	0	0	0	0	0	0	0	0	0	1	0	0	0	0	0	0	0	0	0	0	0	0	0	0	0	0	1	0	0	0	0	0	0	0	1	42.245994	19.091719
2480	2015/011	0	0	0	0	0	0	0	0	0	0	0	0	1	0	0	0	0	0	0	0	0	0	0	0	0	0	0	0	0	0	0	0	0	0	0	0	0	0	0	1	0	0	0	0	0	0	1	0	0	0	0	0	0	0	0	0	1	0	0	0	0	0	0	0	1	42.220461	19.12407
2481	2015/012	1	0	0	0	0	0	0	0	0	0	0	0	0	0	0	0	0	0	0	0	0	0	0	0	0	0	0	0	0	0	0	0	0	0	0	0	0	0	0	1	0	0	0	0	0	0	0	0	0	0	0	0	0	0	0	0	1	0	0	0	0	0	0	0	1	42.225604	19.065948
2482	2015/013	0	0	0	0	0	0	0	0	0	0	0	0	0	0	0	0	0	0	0	0	0	0	0	0	0	0	0	0	0	0	0	0	0	0	0	0	0	0	0	0	0	0	0	0	0	0	0	0	0	0	0	0	0	0	0	0	1	0	0	0	0	0	0	0	1	42.251329	19.087318
2483	2015/014	0	0	0	0	0	1	0	0	0	0	0	0	0	0	1	0	0	0	0	0	0	0	0	0	0	0	0	0	0	0	0	0	0	0	0	0	0	0	0	0	0	0	0	0	0	0	0	0	0	0	0	0	0	0	0	0	1	0	0	0	0	0	0	0	1	42.252115	19.087521
2484	2015/015	1	0	0	0	0	0	0	0	0	0	0	0	0	0	1	0	0	0	0	0	0	0	0	0	0	0	0	0	0	0	0	0	0	0	0	0	0	0	0	1	0	0	0	0	0	0	0	0	0	0	0	0	0	0	0	0	1	0	0	0	0	0	0	0	1	42.254687	19.089632
2485	2015/016	1	0	0	1	0	1	0	0	0	0	0	0	0	0	0	0	0	0	0	0	0	0	0	0	0	0	0	1	0	0	0	0	0	0	0	0	0	0	0	1	0	0	0	0	0	0	0	0	0	0	0	0	0	0	0	0	1	0	0	0	0	0	0	0	1	42.305786	19.04807
2486	2015/017	1	0	0	0	0	0	0	0	0	0	0	0	1	0	0	0	0	0	0	0	0	0	0	0	0	0	0	0	1	0	0	0	0	0	0	0	0	0	0	0	0	0	0	0	0	0	0	0	0	0	0	0	0	0	0	0	1	0	0	0	0	0	0	0	1	42.352334	19.004744
2488	2015/019	1	0	0	0	0	0	0	0	0	0	0	0	0	0	1	0	0	0	0	0	0	0	0	0	0	0	0	0	1	0	0	0	0	0	0	0	0	0	0	1	0	0	0	0	0	0	0	0	0	0	0	0	0	0	0	0	1	0	0	0	0	0	0	0	1	42.365065	18.992377
2489	2015/020	1	0	0	0	0	1	0	0	0	0	0	0	1	0	0	0	0	0	0	0	1	0	0	0	0	0	0	0	1	0	0	0	0	0	0	0	0	0	0	0	0	0	0	0	0	0	0	0	0	0	0	0	0	0	0	0	1	0	0	0	0	0	0	0	1	42.381462	19.008372
2490	2015/021	1	0	0	0	0	0	0	0	0	0	0	0	1	0	0	0	0	0	0	0	0	0	0	0	0	0	0	1	0	0	0	0	0	0	0	0	0	0	0	1	0	0	0	0	0	0	0	0	0	0	0	0	0	0	0	0	1	0	0	0	0	0	0	0	1	42.386509	19.057116
2491	2015/022	0	0	0	0	0	0	0	0	0	0	0	0	0	0	0	0	0	0	0	0	0	0	0	0	0	0	0	0	0	0	0	0	0	0	0	0	0	0	0	0	0	0	0	0	0	0	0	0	0	0	0	0	0	0	0	0	0	0	0	0	0	0	0	0	1	42.375799	18.986275
2492	2015/023	1	0	0	0	0	0	0	0	0	0	0	0	0	0	1	0	0	0	0	0	0	0	0	0	0	0	0	0	0	0	0	0	0	0	0	0	0	0	0	1	0	0	0	0	0	0	0	0	0	0	0	0	0	0	0	0	1	0	0	0	0	0	0	0	1	42.400564	18.915211
2493	2015/024	1	0	0	0	0	0	0	0	0	0	0	0	0	0	0	0	0	0	0	0	0	0	0	0	0	0	0	0	0	0	0	0	0	0	0	0	0	0	0	0	0	0	0	0	0	0	1	0	0	0	0	0	0	0	0	0	0	0	0	0	0	0	0	0	1	42.305591	18.940213
2494	2015/025	1	0	0	0	0	0	0	0	1	0	0	0	0	0	1	0	0	0	0	0	0	0	0	0	0	0	0	0	0	0	0	0	0	0	0	0	0	0	0	0	0	0	0	0	0	0	1	0	0	0	0	0	0	0	0	0	0	1	0	0	0	0	0	0	1	42.227072	18.911729
2495	2015/026	0	0	0	0	0	1	0	0	0	0	0	0	0	0	1	0	0	0	1	0	0	0	0	0	0	0	0	0	0	0	1	0	0	1	0	0	0	0	0	0	0	0	0	0	0	0	1	0	0	0	0	0	0	0	0	0	0	1	0	0	0	0	0	0	1	42.217765	18.954533
2496	2015/027	1	0	0	0	0	0	0	0	0	0	0	0	0	0	0	0	0	0	1	0	0	0	0	0	0	0	0	0	0	0	0	0	0	0	0	0	0	0	0	1	0	0	0	0	0	0	0	0	0	0	0	0	0	0	0	0	0	1	0	0	0	0	0	0	1	42.164913	19.006938
2497	2015/028	1	0	0	1	0	0	0	0	0	0	0	0	0	0	1	0	0	0	1	0	0	0	0	0	0	0	0	0	0	0	0	0	0	0	0	0	0	0	0	0	0	0	0	0	0	0	0	0	0	0	0	0	0	0	0	0	0	1	0	0	0	0	0	0	1	42.153035	19.023731
2498	2015/029	1	0	0	0	0	0	0	0	0	0	0	0	0	0	0	0	0	0	0	0	0	0	0	0	0	0	0	0	0	0	0	0	0	0	0	0	0	0	0	1	0	0	0	0	0	0	0	0	0	0	0	0	0	0	0	0	0	0	0	0	0	0	0	0	1	42.137167	19.047373
2500	2015/031	0	0	0	0	0	1	0	0	0	0	0	0	0	0	0	0	0	0	0	0	0	0	0	0	0	0	0	0	0	0	0	0	0	0	0	0	0	0	0	0	0	0	0	0	0	0	0	0	0	0	0	0	0	0	0	0	1	0	0	0	0	0	0	0	1	42.057189	19.127777
2504	2015/035	0	0	0	0	0	0	0	0	0	0	0	0	0	0	0	0	0	0	0	0	0	0	0	0	0	0	0	0	0	0	0	0	0	0	0	0	0	0	0	1	0	0	0	0	0	0	1	0	0	0	0	0	0	0	0	0	1	0	0	0	0	0	0	0	1	42.050584	19.416234
2505	2015/036	0	0	0	0	0	0	0	0	0	0	0	0	0	0	0	0	0	0	0	0	0	0	0	0	0	0	0	0	0	0	0	0	0	0	0	0	0	0	0	0	0	0	0	0	0	0	0	0	0	0	0	0	0	0	0	0	0	0	0	0	0	0	0	0	1	42.051539	19.417129
2506	2015/037	1	0	0	0	0	1	0	0	0	0	0	0	0	0	1	0	0	0	1	0	0	0	0	0	0	0	0	0	0	0	1	0	0	1	0	0	0	0	0	1	0	0	0	0	0	0	1	0	0	0	0	0	0	0	0	0	1	0	0	0	0	0	0	0	1	42.052444	19.418228
2508	2015/039	0	0	0	0	0	0	0	0	0	0	0	0	0	0	0	0	0	0	0	0	0	0	0	0	0	0	0	0	0	0	0	0	0	0	0	0	0	0	0	1	0	0	0	0	0	0	0	0	0	0	0	0	0	0	0	0	0	0	0	0	0	0	0	0	1	41.898021	19.442456
2509	2015/040	0	0	0	0	0	0	0	0	0	0	0	0	0	0	1	0	0	0	0	0	0	0	0	0	0	0	0	0	0	0	0	0	0	0	0	0	0	0	0	1	0	0	0	0	0	0	0	0	0	0	0	0	0	0	0	0	0	1	0	0	0	0	0	0	1	41.857694	19.492337
2510	2015/041	0	0	0	1	0	1	0	0	0	0	0	0	1	0	0	0	0	0	0	0	0	0	0	0	0	0	0	1	1	0	0	0	0	0	0	0	0	0	0	1	0	0	0	0	0	0	1	0	0	0	0	0	0	0	0	0	0	0	0	0	0	0	0	0	1	41.876295	19.459235
2511	2015/042	0	0	0	1	0	0	0	0	0	1	0	0	0	0	0	0	0	0	0	0	0	0	0	0	0	0	0	0	0	0	0	0	0	0	0	0	0	0	0	1	0	0	0	0	0	0	0	0	0	0	0	0	0	0	0	0	0	0	0	0	0	0	0	0	1	41.883146	19.453315
2512	2015/043	1	0	0	0	0	1	0	0	0	1	0	0	0	0	1	0	0	0	0	0	0	0	0	0	0	0	0	0	0	0	0	0	0	0	0	0	0	0	0	1	0	0	0	0	0	0	1	0	0	0	0	0	0	0	0	0	0	0	0	0	0	0	0	0	1	41.916055	19.426763
2513	2015/044	0	0	0	0	0	0	0	0	0	0	0	0	0	0	1	0	0	0	0	0	0	0	0	0	0	0	0	0	0	0	0	0	0	0	0	0	0	0	0	1	0	0	0	0	0	0	0	0	0	0	0	0	0	0	0	0	0	0	0	0	0	0	0	0	1	41.927042	19.438694
2516	2015/047	1	0	0	0	0	1	0	0	0	1	0	0	0	1	0	0	0	0	1	0	0	0	0	0	0	0	0	0	1	0	0	0	0	0	0	0	0	0	0	1	0	0	0	0	0	0	1	0	0	0	0	0	0	0	0	0	1	0	0	0	0	0	0	0	1	42.058248	19.353537
2517	2015/048	1	0	0	1	0	0	0	0	0	0	0	0	0	0	0	0	0	0	0	0	0	0	0	0	0	0	0	0	1	0	0	0	0	0	0	0	0	0	0	1	0	0	0	0	0	0	0	0	0	0	0	0	0	0	0	0	1	0	0	0	0	0	0	0	1	42.190589	18.989795
2518	2015/049	1	0	0	0	0	1	0	0	0	0	0	0	0	0	0	0	0	0	0	0	0	0	0	0	0	0	0	0	0	0	0	0	0	0	0	0	0	0	0	0	0	0	0	0	0	0	0	0	0	0	0	0	0	0	0	0	0	0	0	0	0	0	0	0	1	42.431244	18.754857
2519	2015/050	1	0	0	0	0	0	0	0	0	1	0	0	1	0	1	0	0	0	1	0	0	0	0	0	0	0	0	1	0	0	0	0	0	0	0	0	0	0	0	1	0	0	0	0	0	0	0	0	0	0	0	0	0	0	0	0	0	1	0	0	0	0	0	0	1	42.4269	18.7756
2521	2015/052	0	0	0	0	0	0	0	0	0	0	0	0	1	0	0	0	0	0	0	0	0	0	0	0	0	0	0	0	0	0	0	0	0	0	0	0	0	0	0	0	0	0	0	0	0	0	0	0	0	0	0	0	0	0	0	0	0	1	0	0	0	0	0	0	1	42.409482	18.778685
2522	2015/053	1	0	0	0	0	1	0	0	0	1	0	0	1	0	0	0	0	0	0	0	0	0	1	0	0	0	0	0	1	0	0	0	0	0	0	0	0	0	0	0	0	0	0	0	0	0	0	0	0	0	0	0	0	0	0	0	0	0	0	0	0	0	0	0	1	42.399268	18.783629
2523	2015/054	1	0	0	0	0	0	0	0	0	0	0	0	1	0	0	0	0	0	1	0	0	0	0	0	0	0	0	1	1	0	0	0	0	0	0	0	0	0	0	0	0	0	0	0	0	0	0	0	0	0	0	0	0	0	0	0	1	0	0	0	0	0	0	0	1	42.431639	18.812576
2526	2015/057	1	0	0	0	0	0	0	0	0	0	0	0	0	0	1	0	0	0	1	0	0	0	0	0	0	0	0	1	0	0	0	0	0	0	0	0	0	0	0	0	0	0	0	0	1	0	0	0	0	0	0	0	0	0	0	0	0	0	0	0	0	0	0	0	1	42.487689	18.560329
2527	2015/058	0	0	0	0	0	0	0	0	1	0	0	0	0	0	1	0	0	0	0	0	0	0	0	0	0	0	0	0	0	0	0	0	0	0	0	0	0	0	0	1	0	0	0	0	0	0	0	0	0	0	0	0	0	0	0	0	0	0	0	0	0	0	0	0	1	42.445502	18.504971
2528	2015/059	1	0	0	0	0	0	0	0	0	1	0	0	1	0	0	0	0	0	1	0	1	0	0	0	0	0	0	0	0	0	0	0	0	0	0	0	0	0	0	0	0	0	0	0	0	0	0	0	0	0	0	0	0	0	0	0	1	0	0	0	0	0	0	0	1	42.731904	18.938231
2529	2015/060	1	0	0	0	0	1	0	0	0	0	0	0	1	0	0	0	0	0	0	0	0	0	0	0	0	0	0	0	1	0	0	0	0	0	0	0	0	0	0	1	0	0	0	0	1	0	1	0	0	0	0	0	0	0	0	0	0	0	0	0	0	0	0	0	1	42.675074	19.029887
2534	2015/065	0	0	0	0	0	1	0	0	1	0	0	0	0	1	0	1	0	0	1	0	0	0	0	0	0	0	0	0	0	1	0	0	1	0	0	0	0	0	0	0	0	0	0	0	0	0	0	0	1	0	0	0	0	0	1	0	0	0	0	0	0	0	0	0	3	40.94447231	20.82700524
2535	2015/065B	0	0	0	0	0	0	0	0	1	1	0	0	0	0	0	0	0	0	0	0	0	0	0	0	0	0	0	0	0	1	0	0	1	0	0	0	0	0	0	0	0	0	0	0	0	0	0	0	0	0	0	0	0	0	1	0	0	0	0	0	0	0	0	0	2	40.94391751	20.82827174
2536	2015/065C	0	0	0	0	0	1	0	0	0	1	0	0	1	0	0	0	0	0	0	0	1	0	0	0	0	0	0	0	0	1	0	0	1	0	0	0	0	0	0	0	0	0	0	0	0	0	0	0	0	0	0	0	0	0	1	0	0	0	0	0	0	0	0	0	1	40.9429225	20.82516398
2537	2015/066	0	0	0	0	0	1	0	0	0	0	0	0	0	0	1	0	0	0	0	0	0	0	0	0	0	0	0	0	0	1	0	0	1	0	0	0	0	0	0	0	0	0	0	0	0	0	0	0	0	0	0	0	0	0	1	0	0	0	0	0	0	0	0	0	1	40.88723372	20.97285219
2538	2015/067	0	0	0	0	0	1	0	0	1	0	0	0	0	1	0	0	0	0	0	0	0	0	0	0	0	0	0	0	0	1	0	0	1	0	0	0	0	0	0	0	0	0	0	0	0	0	0	0	0	0	0	0	0	0	1	0	0	0	0	0	0	0	0	0	1	40.88087177	20.95902013
2540	2015/069	0	0	0	0	0	0	0	0	1	0	0	0	0	0	0	0	0	0	0	0	0	0	0	0	0	0	0	0	0	0	0	0	0	0	0	0	0	0	0	0	0	0	0	0	0	0	0	0	0	0	0	0	0	0	0	0	0	0	0	0	0	0	0	0	1	40.86990623	20.92949186
2541	2015/070	0	0	0	0	0	1	0	0	0	0	0	0	0	1	1	0	0	0	1	0	0	0	0	0	0	0	0	0	0	1	0	0	1	0	0	0	0	0	0	0	0	0	0	0	0	0	0	0	0	0	0	0	0	0	0	1	0	0	0	0	0	0	0	0	1	40.84218845	20.96164442
2542	2015/071	0	0	0	0	0	0	0	0	0	0	0	0	0	0	0	0	0	0	0	0	0	0	0	0	0	0	0	0	0	0	0	0	0	0	0	0	0	0	0	0	0	0	0	0	0	0	0	0	0	0	0	0	0	0	0	1	0	0	0	0	0	0	0	0	1	40.8584	20.9474
2544	2015/073	0	0	0	0	0	0	0	0	0	0	0	0	0	0	0	0	0	0	0	0	0	0	0	0	0	0	0	0	0	0	0	0	0	0	0	0	0	0	0	0	0	0	0	0	0	0	0	0	0	0	0	0	0	0	0	0	0	0	0	0	0	0	0	0	1	41.30277654	20.62352984
2545	2015/074	0	0	0	0	0	0	0	0	1	0	0	0	0	0	0	0	0	0	0	0	0	0	0	0	0	0	0	0	0	0	0	0	0	0	0	0	0	0	0	0	0	0	0	0	0	0	0	0	0	0	0	0	0	0	0	0	0	0	0	0	0	0	0	0	3	41.39583377	20.58952983
2546	2015/075	0	0	0	0	0	0	0	0	0	1	0	0	1	0	0	0	0	0	0	0	0	0	0	0	0	0	0	0	0	0	0	0	0	0	0	0	0	0	0	0	0	0	0	0	0	0	0	0	0	0	0	0	0	0	0	0	0	0	0	0	0	0	0	0	1	41.39471672	20.59765541
2551	2015/079	0	0	0	0	0	1	0	0	1	1	0	0	1	1	1	0	0	1	1	0	0	0	0	0	0	0	0	0	0	0	0	0	0	0	0	0	0	0	0	0	0	0	0	0	0	0	0	0	0	0	0	0	0	0	0	0	0	0	0	0	0	0	0	0	1	41.85151082	20.32823372
2552	2015/080	0	0	0	0	0	0	0	0	0	0	0	0	0	0	0	0	0	1	0	0	0	0	0	0	0	0	0	0	0	0	0	0	0	0	0	0	0	0	0	0	0	0	0	0	0	0	0	0	0	0	0	0	0	0	0	0	0	0	0	0	0	0	0	0	1	41.84653482	20.33040832
2555	2015/083	1	0	0	0	0	1	0	0	1	0	0	0	1	0	1	0	0	0	0	0	0	0	0	0	0	0	0	0	0	0	0	0	0	0	0	0	0	0	0	0	0	0	0	0	0	0	0	0	0	0	0	0	0	0	0	0	0	0	0	0	0	0	0	1	1	41.88304371	20.33700555
2556	2015/084	0	0	0	0	0	0	0	0	0	1	0	0	0	0	0	0	0	1	0	0	0	0	0	0	0	0	0	0	0	0	0	0	0	0	0	0	0	0	0	0	0	0	0	0	0	0	0	0	0	0	0	0	0	0	0	0	0	0	0	0	0	0	0	1	1	41.9253802	20.35690787
2558	2015/086	0	0	0	0	0	0	0	0	0	0	0	0	0	0	0	0	0	0	0	0	0	0	0	0	0	0	0	0	0	0	0	0	0	0	0	0	0	0	0	0	0	0	0	0	0	0	0	0	0	0	0	0	0	0	0	0	0	0	0	0	0	0	0	0	1	41.94538445	20.37157402
2559	2015/087	1	0	0	0	0	1	0	0	0	1	0	0	0	0	0	0	0	1	1	0	0	0	0	0	0	0	0	1	0	0	0	0	0	0	0	0	0	0	0	0	0	0	0	0	0	0	0	0	0	0	0	0	0	0	0	0	0	0	0	0	0	0	0	0	1	41.95019332	20.38137884
2560	2015/088	0	0	0	0	0	1	0	0	0	0	0	0	1	0	0	0	0	0	0	0	0	0	0	0	0	0	0	0	0	0	0	0	0	0	0	0	0	0	0	0	0	0	0	0	0	0	0	0	0	0	0	0	0	0	0	0	0	0	0	0	0	0	0	0	1	42.10154166	20.42091854
2561	2015/089	0	0	0	0	0	0	0	0	0	0	0	0	0	0	0	0	0	0	0	0	0	0	0	0	0	0	0	0	0	0	0	0	0	0	0	0	0	0	0	0	0	0	0	0	0	0	0	0	0	0	0	0	0	0	0	0	0	0	0	0	0	0	0	0	1	42.19204559	20.41665978

Table S1.5

2562	2015/090		0	0	0	0	0	1	0	0	0	0	0	0	1	0	0	0	0	0	1	0	0	0	0	0	0	0	0	0	0	0	0	0	0	0	0	0	1	0	0	0	0	0	0	0	0	0	0	0	0	0	0	0	0	0	0	0	0	0	0	0	0	1	42.34468063	20.03529819
2563	2015/091		1	0	0	0	0	1	0	0	1	1	0	0	1	1	0	0	1	0	1	0	0	0	0	0	1	0	0	0	0	1	0	0	0	0	0	1	0	0	0	0	0	0	0	0	0	1	0	0	0	0	0	0	0	0	0	0	0	0	0	0	0	3	42.36687088	20.02698384
2564	2015/092		1	0	0	0	0	0	0	0	1	0	0	0	1	0	0	0	0	0	1	0	0	0	0	0	0	0	0	0	0	1	0	0	0	0	0	1	0	0	0	0	0	0	0	0	0	1	0	0	0	0	0	0	0	0	0	0	0	0	0	0	0	3	42.36780253	20.009351
2567	2015/094		0	0	0	0	0	0	0	0	0	0	0	0	0	0	0	0	0	0	0	0	0	0	0	0	0	0	0	0	0	0	0	0	0	0	0	0	1	0	0	0	0	0	0	0	0	0	0	0	0	0	0	0	0	0	0	0	0	0	0	0	0	1	42.35562044	20.0701711
2574	2015/101		1	0	0	0	0	0	0	0	0	1	0	0	0	0	0	0	0	0	1	0	0	0	0	1	0	0	0	0	0	0	0	0	0	0	0	0	1	0	0	0	0	0	0	0	0	0	0	0	0	0	0	0	0	0	0	0	0	0	0	0	0	1	42.12477369	19.61498422
2575	2015/102		1	0	0	0	0	1	0	0	0	1	0	0	0	0	0	0	0	0	0	0	0	0	0	0	0	0	0	0	0	0	0	0	0	0	0	0	1	0	0	0	0	0	0	0	0	0	0	0	0	0	0	0	0	0	0	0	0	0	0	0	0	1	42.32498316	19.59398403
2576	2015/103		1	0	1	0	0	1	0	0	0	1	0	0	0	0	1	0	0	0	0	0	0	0	0	1	0	0	0	0	0	0	0	0	0	0	0	0	1	0	0	0	0	0	0	0	0	0	0	0	0	0	0	0	0	0	0	0	0	0	0	0	0	1	42.33062913	19.60567578
2577	2015/104		1	0	0	0	0	0	0	0	0	0	0	0	0	0	0	0	0	0	0	0	0	0	0	0	0	0	0	0	0	0	0	0	0	0	0	0	0	0	0	0	0	0	0	0	0	0	0	0	0	0	0	0	0	0	0	0	0	0	0	0	0	1	42.276362	19.516466
2578	2015/105		0	0	0	0	0	0	0	0	0	1	0	0	0	0	0	0	0	0	0	0	0	0	0	1	0	0	0	0	0	0	0	0	0	0	0	0	0	0	0	0	0	0	0	0	0	0	0	0	0	0	0	0	0	0	0	0	0	0	0	0	0	1	42.31955177	19.36934819
2581		0	1	0	0	0	0	0	0	0	0	0	0	0	0	0	0	0	0	0	1	0	0	0	0	0	0	0	0	0	0	0	0	0	0	0	0	0	0	0	0	0	0	0	0	0	0	0	0	0	0	0	0	0	0	0	0	0	0	0	0	0	0	3	46.378056	14.207361
2582		0	0	0	0	0	0	0	0	0	0	0	0	0	0	0	0	0	0	0	1	0	0	0	0	0	0	0	0	0	0	0	0	0	0	0	0	0	0	0	0	0	0	0	0	0	0	0	0	0	0	0	0	0	0	0	0	0	0	0	0	0	0	3	40.107694	21.021167
2583		0	0	0	0	0	0	0	0	0	0	0	0	0	0	0	0	0	0	0	1	0	0	0	0	0	0	0	0	0	0	0	0	0	0	0	0	0	0	0	0	0	0	0	0	0	0	0	0	0	0	0	0	0	0	0	0	0	0	0	0	0	0	1	44.60935	22.274417
2584	DL85		0	0	0	0	0	0	0	0	0	0	0	0	0	0	0	0	0	0	1	0	0	0	0	0	0	0	0	0	0	0	0	0	0	0	0	0	0	0	0	0	0	0	0	0	0	0	0	0	0	0	0	0	0	0	0	0	0	0	0	0	0	1	45.029186	14.497518
2585	DL250		0	0	0	0	0	0	0	0	0	0	0	0	0	0	0	0	0	0	1	0	0	0	0	0	0	0	0	0	0	0	0	0	0	0	0	0	0	0	0	0	0	0	0	0	0	0	0	0	0	0	0	0	0	0	0	0	0	0	0	0	0	1	44.6988	14.9082
2586	DL152		0	0	0	0	0	0	0	0	0	0	0	0	0	0	0	0	0	0	1	0	0	0	0	0	0	0	0	0	0	0	0	0	0	0	0	0	0	0	0	0	0	0	0	0	0	0	0	0	0	0	0	0	0	0	0	0	0	0	0	0	0	1	44.292212	15.175082
2587		0	1	0	0	0	0	0	0	0	0	0	0	0	0	0	0	0	0	0	0	0	0	0	0	0	0	0	0	0	0	0	0	0	0	0	0	0	0	0	0	0	0	0	0	0	0	0	0	0	0	0	0	0	0	0	0	0	0	0	0	0	0	1	43.511722	18.671806
2588		0	1	0	0	0	0	0	0	0	0	0	0	0	0	0	0	0	0	0	0	0	0	0	0	0	0	0	0	0	0	0	0	0	0	0	0	0	0	0	0	0	0	0	0	0	0	0	0	0	0	0	0	0	0	0	0	0	0	0	0	0	0	1	43.724183	18.604183
2589		0	1	0	0	0	0	0	0	0	0	0	0	0	0	0	0	0	0	0	0	0	0	0	0	0	0	0	0	0	0	0	0	0	0	0	0	0	0	0	0	0	0	0	0	0	0	0	0	0	0	0	0	0	0	0	0	0	0	0	0	0	0	1	43.7661	18.54655
2590		0	1	0	0	0	0	0	0	0	0	0	0	0	0	0	0	0	0	0	0	0	0	0	0	0	0	0	0	0	0	0	0	0	0	0	0	0	0	0	0	0	0	0	0	0	0	0	0	0	0	0	0	0	0	0	0	0	0	0	0	0	0	3	46.366542	14.306749
2591	DL20		1	0	0	0	0	0	0	0	0	0	0	0	0	0	0	0	0	0	0	0	0	0	0	0	0	0	0	0	0	0	0	0	0	0	0	0	0	0	0	0	0	0	0	0	0	0	0	0	0	0	0	0	0	0	0	0	0	0	0	0	0	3	46.406944	14.146111
2592	DL246		1	0	0	0	0	0	0	0	0	0	0	0	0	0	0	0	0	0	0	0	0	0	0	0	0	0	0	0	0	0	0	0	0	0	0	0	0	0	0	0	0	0	0	0	0	0	0	0	0	0	0	0	0	0	0	0	0	0	0	0	0	1	44.782167	14.985567
2593		0	1	0	0	0	0	0	0	0	0	0	0	0	0	0	0	0	0	0	0	0	0	0	0	0	0	0	0	0	0	0	0	0	0	0	0	0	0	0	0	0	0	0	0	0	0	0	0	0	0	0	0	0	0	0	0	0	0	0	0	0	0	1	44.17295	16.5761
2594		0	1	0	0	0	0	0	0	0	0	0	0	0	0	0	0	0	0	0	0	0	0	0	0	0	0	0	0	0	0	0	0	0	0	0	0	0	0	0	0	0	0	0	0	0	0	0	0	0	0	0	0	0	0	0	0	0	0	0	0	0	0	1	44.276211	15.874985
2595		0	1	0	0	0	0	0	0	0	0	0	0	0	0	0	0	0	0	0	0	0	0	0	0	0	0	0	0	0	0	0	0	0	0	0	0	0	0	0	0	0	0	0	0	0	0	0	0	0	0	0	0	0	0	0	0	0	0	0	0	0	0	1	45.745363	14.46735
2596	DL90		0	0	0	0	0	0	0	0	0	0	0	0	0	0	0	0	0	0	0	0	0	0	0	0	0	0	0	0	0	0	0	0	0	0	0	0	0	0	1	0	0	0	0	0	0	0	0	0	0	0	0	0	0	0	0	0	0	0	0	0	0	1	43.289528	18.617778
2597		0	0	0	0	0	0	0	0	0	0	0	0	0	0	0	0	0	0	0	0	0	0	0	0	0	0	0	0	0	0	0	0	0	0	0	0	0	0	0	1	0	0	0	0	0	0	0	0	0	0	0	0	0	0	0	0	0	0	0	0	0	0	1	44.60935	22.274417
2598		0	0	0	0	0	0	0	0	0	0	0	0	0	0	0	0	0	0	0	0	0	0	0	0	0	0	0	0	0	0	0	0	1	0	0	0	0	0	0	0	0	0	0	0	0	0	0	0	0	0	0	0	0	0	0	0	0	0	0	0	0	0	1	37.640833	22.206611
2599		0	0	0	0	0	0	0	0	0	0	0	0	0	0	0	0	0	0	0	0	0	0	0	0	0	0	0	0	0	0	0	0	1	0	0	0	0	0	0	0	0	0	0	0	0	0	0	0	0	0	0	0	0	0	0	0	0	0	0	0	0	0	1	36.8871	22.67085
2600		0	0	0	0	0	0	0	0	0	0	0	0	0	0	0	0	0	0	0	0	0	0	0	0	0	0	0	0	0	0	0	0	1	0	0	0	0	0	0	0	0	0	0	0	0	0	0	0	0	0	0	0	0	0	0	0	0	0	0	0	0	0	1	36.804183	22.6181
2601		0	0	0	0	0	0	0	0	0	0	0	0	0	0	0	0	0	0	0	0	0	0	0	0	0	0	0	0	0	0	0	0	1	0	0	0	0	0	0	0	0	0	0	0	0	0	0	0	0	0	0	0	0	0	0	0	0	0	0	0	0	0	1	37.335467	22.644983
2602		0	0	0	0	0	0	0	0	0	0	0	0	0	0	0	0	0	0	0	0	0	0	0	0	0	0	0	0	0	0	0	0	1	0	0	0	0	0	0	0	0	0	0	0	0	0	0	0	0	0	0	0	0	0	0	0	0	0	0	0	0	0	1	37.094917	22.33285
2603		0	0	0	0	0	0	0	0	0	0	0	0	0	0	0	0	0	0	0	0	0	0	0	0	0	0	0	0	0	0	0	0	1	0	0	0	0	0	0	0	0	0	0	0	0	0	0	0	0	0	0	0	0	0	0	0	0	0	0	0	0	0	1	37.079183	22.291133
2604		0	0	0	0	0	0	0	0	0	0	0	0	0	0	0	0	0	0	0	0	0	0	0	0	0	0	0	0	0	0	0	0	1	0	0	0	0	0	0	0	0	0	0	0	0	0	0	0	0	0	0	0	0	0	0	0	0	0	0	0	0	0	1	38.084983	22.033933
2605		0	1	0	0	0	0	0	0	0	0	0	0	0	0	0	0	0	0	0	0	0	0	0	0	0	0	0	0	0	0	0	0	1	0	0	0	0	0	0	0	0	0	0	0	0	0	0	0	0	0	0	0	0	0	0	0	0	0	0	0	0	0	1	37.243117	21.82745
2606		0	0	0	0	0	0	0	0	0	0	0	0	0	0	0	0	0	0	0	0	0	0	0	0	0	0	0	0	0	0	0	0	1	0	0	0	0	0	0	0	0	0	0	0	0	0	0	0	0	0	0	0	0	0	0	0	0	0	0	0	0	0	1	37.539633	22.046467
2607		0	0	0	0	0	0	0	0	0	0	0	0	0	0	0	0	0	0	0	0	0	0	0	0	0	0	0	0	0	0	0	0	1	0	0	0	0	0	0	0	0	0	0	0	0	0	0	0	0	0	0	0	0	0	0	0	0	0	0	0	0	0	1	37.533667	22.053183
2608		0	0	0	0	0	0	0	0	0	0	0	0	0	0	0	0	0	0	0	0	0	0	0	0	0	0	0	0	0	0	0	0	1	0	0	0	0	0	0	0	0	0	0	0	0	0	0	0	0	0	0	0	0	0	0	0	0	0	0	0	0	0	1	37.622183	22.050617
2609		0	0	0	0	0	0	0	0	0	0	0	0	0	0	0	0	0	0	0	0	0	0	0	0	0	0	0	0	0	0	0	0	1	0	0	0	0	0	0	0	0	0	0	0	0	0	0	0	0	0	0	0	0	0	0	0	0	0	0	0	0	0	1	37.9338	22.211783
2610		0	0	0	0	0	0	0	0	0	0	0	0	0	0	0	0	0	0	0	0	0	0	0	0	0	0	0	0	0	0	0	0	1	0	0	0	0	0	0	0	0	0	0	0	0	0	0	0	0	0	0	0	0	0	0	0	0	0	0	0	0	0	2	37.942383	22.23445
2611		0	1	0	0	0	0	0	0	0	0	0	0	0	0	0	0	0	0	0	0	0	0	0	0	0	0	0	0	0	0	0	0	1	0	0	0	0	0	0	0	0	0	0	0	0	0	0	0	0	0	0	0	0	0	0	0	0	0	0	0	0	0	1	37.9337	22.166183
2612		0	0	0	0	0	0	0	0	0	0	0	0	0	0	0	0	0	0	0	0	0	0	0	0	0	0	0	0	0	0	0	0	1	0	0	0	0	0	0	0	0	0	0	0	0	0	0	0	0	0	0	0	0	0	0	0	0	0	0	0	0	0	1	38.36505	22.128117
2613		0	0	0	0	0	0	0	0	0	0	0	0	0	0	0	0	0	0	0	0	0	0	0	0	0	0	0	0	0	0	0	0	1	0	0	0	0	0	0	0	0	0	0	0	0	0	0	0	0	0	0	0	0	0	0	0	0	0	0	0	0	0	1	38.347367	22.3141
2614		0	1	0	0	0	0	0	0	0	0	0	0	0	0	0	0	0	0	0	0	0	0	0	0	0	0	0	0	0	0	0	0	1	0	0	0	0	0	0	0	0	0	0	0	0	0	0	0	0	0	0	0	0	0	0	0	0	0	0	0	0	0	1	38.563783	22.348983
2615		0	1	0	0	0	0	0	0	0	0	0	0	0	0	0	0	0	0	0	0	0	0	0	0	0	0	0	0	0	0	0	0	1	0	0	0	0	0	0	0	0	0	0	0	0	0	0	0	0	0	0	0	0	0	0	0	0	0	0	0	0	0	1	38.600117	22.326617
2616		0	1	0	0	0	0	0	0	0	0	0	0	0	0	0	0	0	0	0	0	0	0	0	0	0	0	0	0	0	0	0	0	0	0	0	0	0	0	0	0	0	0	0	0	0	0	0	0	0	0	0	0	0	0	0	0	0	0	0	0	0	0	1	38.420278	22.242222
2617		0	1	0	0	0	0	0	0	0	0	0	0	0	0	0	0	0	0	0	0	0	0	0	0	0	0	0	0	0	0	0	0	0	0	0	0	0	0	0	0	0	0	0	0	0	0	0	0	0	0	0	0	0	0	0	0	0	0	0	0	0	0	1	38.624722	23.844444
2618		0	1	0	0	0	0	0	0	0	0	0	0	0	0	0	0	0	0	0	0	0	0	0	0	0	0	0	0	0	0	0	0	0	0	0	0	0	0	0	0	0	0	0	0	0	0	0	0	0	0	0	0	0	0	0	0	0	0	0	0	0	0	1	37.186667	21.913889
2619		0	1	0	0	0	0	0	0	0	0	0	0	0	0	0	0	0	0	0	0	0	0	0	0	0	0	0	0	0	0	0	0	0	0	0	0	0	0	0	0	0	0	0	0	0	0	0	0	0	0	0	0	0	0	0	0	0	0	0	0	0	0	1	39.967778	20.665278
2620		0	1	0	0	0	0	0	0	0	0	0	0	0	0	0	0	0	0	0	0	0	0	0	0	0	0	0	0	0	0	0	0	0	0	0	0	0	0	0	0	0	0	0	0	0	0	0	0	0	0	0	0	0	0	0	0	0	0	0	0	0	0	1	38.398778	22.005556
2621		0	1	0	0	0	0	1	0	0	0	0	0	0	1	0	0	0	0	0	1	0	0	0	0	0	0	0	0	0	0	1	0	0	0	0	0	0	0	0	1	0	0	0	0	0	0	0	0	0	0	0	0	0	0	0	0	0	0	0	0	0	0	1	44.101144	21.469257
2622		0	0	0	0	0	0	0	0	0	0	0	0	0	0	0	0	0	0	0	1	0	0	0	0	0	0	0	0	0	0	1	0	0	0	0	0	0	0	0	1	0	0	0	0	0	0	0	0	0	0	0	0	0	0	0	0	0	0	0	0	0	0	1	43.974361	21.515333
2623		0	1	0	0	0	0	0	0	0	0	0	0	0	1	0	0	0	0	0	1	0	0	0	0	0	1	0	0	0	0	1	0	0	0	0	0	0	0	0	1	0	0	0	0	0	0	0	0	0	0	0	0	0	0	0	0	0	0	0	0	0	0	1	44.028972	21.957889
2624		0	0	0	0	0	0	0	0	0	0	0	0	0	1	0	0	0	0	0	1	0	0	0	1	0	0	0	0	0	0	1	0	0	0	1	0	0	0	0	0	0	1	0	0	0	0	0	0	0	0	0	0	0	0	0	0	0	0	0	0	0	0	1	43.281361	22.062111
2625		0	0	0	0	0	0	0	0	0	0	0	0	0	1	0	0	0	0	0	1	0	0	0	0	0	0	0	0	0	0	1	0	0	0	0	0	0	0	0	0	0	0	0	0	0	0	0	0	0	0	0	0	0	0	0	0	0	0	0	0	0	0	1	43.795306	21.9035
2626		0	0	0	0	0	0	1	0	0	0	0	0	0	0	0	0	0	0	0	0	0	0	0	0	0	0	0	0	0	0	0	0	0	0	0	0	0	0	0	0	0	0	0	0	0	0	0	0	0	0	0	0	0	0	0	0	0	0	0	0	0	0	2	41.80347	21.300001
2627		0	1	0	0	0	0	0	0	0	0	0	0	0	1	0	0	0	0	0	1	0	0	0	0	0	0	0	0	0	0	0	0	0	0	0	0	0	0	0	0	0	1	0	0	0	0	0	0	0	0	0	0	0	0	0	0	0	0	0	0	0	0	1	43.020667	22.364306
2628		0	1	0	0	0	0	0	0	0	0	0	0	0	1	0	0	0	0	0	1	0	0	0	1	0	0	0	0	0	0	0	0	0	0	0	0	0	0	0	0	0	1	0	0	0	0	0	0	0	0	0	0	0	0	0	0	0	0	0	0	0	0	1	43.030389	22.388806
2629		0	0	0	0	0	0	0	0	0	0	1	0	0	0	0	0	0	0	0	1	0	0	0	0	0	0	0	0	0	0	0	0	0	0	0	0	0	0	0	0	0	1	0	0	0	0	0	0	0	0	0	0	0	0	0	0	0	0	0	0	0	0	1	43.429222	22.4175
2630		0	0	0	0	0	0	1	0	0	0	0	0	0	0	1	0	0	0	0	1	0	0	0	0	0	0	0	0	0	0	1	0	0	0	0	0	0	0	0	0	0	1	0	0	0	0	0	0	0	0	0	0	0	0	0	0	0	0	0	0	0	0	1	43.536528	22.285278
2631		0	0	0	0	0	0	0	0	0	0	0	0	0	0	0	0	0	0	0	1	0	0	0	0	0	0	0	0	0	0	0	0	0	0	0	0	0	0	0	0	0	0	0	0	0	0	0	0	0	0	0	0	0	0	0	0	0	0	0	0	0	0	1	43.760471	22.310321
2632		0	0	0	0	0	0	1	0	0	0	0	0	0	1	0	0	0	0	0	0	0	0	0	0	0	0	0	0	0	0	0	0	0	0	0	0	0	0	0	0	0	0	0	0	0	0	0	0	0	0	0	0	0	0	0	0	0	0	0	0	0	0	1	43.834444	21.9545
2633		0	1	0	0	0	0	1	0	0	1	0	0	0	1	0	0	0	0	1	1	0	0	0	1	0	1	1	0	0	0	1	0	0	0	0	0	0	0	0	0	0	1	0	0	0	0	0	0	0	0	0	0	0	0	0	0	0	0	0	0	0	0	3	44.382417	22.341694
2634		0	1	0	0	0	0	0	0	0	1	1	0	0	0	0	0	0	0	1	1	0	0	0	0	0	1	1	0	0	0	1	0	0	0	0	0	0	0	0	0	0	0	0	0	0	0	0	0	0	0	0	0	0	0	0	0	0	0	0	0	0	0	1	44.292306	22.277583
2635		0	1	0	0	0	0	1	0	0	1	1	0	0	1	0	0	1	1	1	1	0	1	1	1	0	1	1	0	0	0	1	0	0	0	0	0	0	0	0	0	0	0	0	0	0	0	0	0	0	0	0	0	0	0	0	0	0	0	0	0	0	0	1	44.185727	22.154682
2636		0	1	0	0	0	0	0	0	0	1	1	0	0	1	0	1	1	1	1	1	0	1	1	1	0	1	1	0	0	0	1	0	0	0	0	0	1	0	0	1	0	0	0	0	0	0	0	0	0	0	0	0	0	0	0	0	0	0	0	0	0	0	1	44.61375	22.275222
2637		0	0	0	0	0	0	0	0	0	0	0	0	0	1	0	0	0	0	0	0	0	1	0	0	0	0	0	0	0	0	1	0	0	0	0	0	0	0	0	0	0	0	0	0	0	0	0	0	0	0	0	0	0	0	0	0	0	0	0	0	0	0	3	44.441475	21.953446

Table S1.6

site_no	site_code	prosobranch	Carychiidae	Ferussaciidae	Azeciidae	Cochlicopidae	Enidae	Euconulidae	Gastrodontidae	Oxychilidae	Pristilomatidae	Bradybaenidae	Cochlicellidae	Helicidae	Helicodontidae	Hygromiidae	Vitrinidae	Punctidae	Argnidae
3	1992/22	1	0	0	0	0	1	0	0	1	1	0	0	1	0	1	0	0	0
16	1993/14	1	0	0	0	0	0	0	0	0	0	0	1	1	0	1	0	0	0
19	1993/16	1	0	0	0	0	0	0	0	1	0	0	0	1	0	1	1	0	0
20	1993/12	1	0	0	0	0	1	0	0	0	0	0	0	0	1	1	0	0	0
25	1994/53	0	0	0	0	0	1	0	0	1	1	0	0	1	0	1	0	0	0
28	1994/57	1	0	0	0	0	1	0	0	0	0	0	0	1	0	1	0	0	0
33	1994/62	0	0	0	0	0	0	0	0	0	0	0	0	0	0	0	0	0	0
34	1996/03	0	0	0	0	0	0	0	0	0	0	0	0	0	0	0	0	0	0
36	1996/05	1	0	0	0	0	0	0	0	0	0	0	0	0	0	0	0	0	0
38	1996/07	0	0	0	0	0	1	0	0	0	0	0	0	1	0	0	0	0	0
39	1996/08	0	0	0	0	0	0	0	0	0	0	0	0	0	0	1	0	0	0
41	1996/10	0	0	0	0	0	1	0	0	0	0	0	0	1	0	0	0	0	0
42	1996/11	0	0	0	0	0	0	0	0	0	0	0	0	0	0	0	0	0	0
43	1996/12	0	0	0	0	0	1	0	0	0	0	0	0	0	0	0	0	0	0
46	1996/15	1	0	0	0	0	0	0	0	0	0	0	0	1	0	0	0	0	0
48	1996/17	0	0	0	0	0	0	0	0	0	0	0	0	0	0	0	0	0	0
52	1996/21	1	0	0	0	0	0	0	0	0	0	0	0	1	0	1	0	0	0
53	1996/22	1	0	0	0	0	0	0	0	0	0	0	0	1	0	1	0	0	0
54	1996/23	0	0	0	0	0	0	0	0	0	0	0	0	0	0	1	0	0	0
57	1996/26	0	0	0	0	0	0	0	0	0	0	0	0	0	0	1	0	0	0
58	1996/27	1	0	0	0	0	0	0	0	0	0	0	0	0	1	1	0	0	0
59	1996/28	1	0	0	0	0	0	0	0	0	0	0	0	0	0	1	0	0	0
60	1996/29	1	0	0	0	0	0	0	0	0	0	0	0	0	0	1	0	0	0
61	1996/30	1	0	1	0	0	1	0	0	1	1	0	0	0	0	0	0	0	1
62	1996/31	1	0	0	0	0	1	0	0	0	1	0	0	0	0	0	0	0	1
64	1996/33	1	0	0	0	0	0	0	0	1	0	0	0	1	1	1	0	0	0
66	1996/35	1	0	0	1	0	0	0	0	0	1	0	0	1	0	1	0	0	0
70	1996/39	1	0	0	0	0	0	0	0	1	0	0	0	1	1	1	0	0	0
73	1996/42	1	0	0	0	0	0	0	0	0	0	0	0	0	0	0	0	0	0
74	1996/43	1	0	0	0	0	0	0	0	1	0	0	0	1	1	0	0	0	0
75	1996/44	1	0	0	0	0	0	0	0	0	0	0	0	0	0	0	0	0	0
77	1996/46	1	0	0	0	0	0	0	0	0	0	0	0	0	1	1	0	0	0
78	1996/47	1	0	0	0	0	0	0	0	0	0	0	0	0	0	0	0	0	0
79	1996/48	1	0	0	0	0	0	0	0	0	0	0	0	1	0	1	0	0	0
82	1996/51	1	0	0	0	0	0	0	0	1	0	0	0	0	0	1	0	0	0
83	1996/52	0	0	0	0	0	0	0	0	0	0	0	0	0	1	1	0	0	0
84	1996/53	1	0	0	0	0	1	0	0	0	0	0	0	1	0	1	0	0	0
85	1996/54	1	0	0	0	0	1	0	0	1	1	0	0	1	1	1	0	0	0
88	1996/57	1	0	0	0	0	1	0	0	0	0	0	0	0	1	1	0	0	0
89	1996/58	0	0	0	0	0	0	0	0	0	0	0	0	0	0	1	0	0	0
92	1996/61	0	0	0	0	0	1	0	0	0	0	0	0	0	0	0	0	0	0
94	1996/63	1	0	0	0	0	0	0	0	0	0	0	0	1	0	1	0	0	0
95	1996/64	1	0	0	0	0	0	0	0	0	0	0	1	0	1	1	0	0	0
96	1996/65	1	0	1	1	0	1	0	0	0	1	0	0	1	0	0	0	0	0
97	1996/66	1	0	1	1	0	0	0	0	0	0	0	0	0	1	1	0	0	0
98	1996/67	1	0	0	0	0	0	0	0	0	0	0	0	0	1	1	0	0	0
100	1996/69	1	0	0	0	0	0	0	0	0	0	0	0	0	1	1	0	0	0
102	1996/71	1	0	1	1	0	0	0	0	0	0	0	0	1	1	1	0	0	0
103	1996/72	1	0	0	0	0	1	0	0	0	0	0	0	0	0	1	0	0	0
105	1996/74	1	0	0	0	0	0	0	0	0	0	0	0	1	0	0	0	0	0
114	1996/83	0	0	0	0	0	1	0	0	0	0	0	0	0	0	1	1	0	0
115	2000/14	1	0	0	0	0	1	0	0	0	0	0	0	1	0	1	0	0	0
116	2000/15	0	0	0	0	0	0	0	0	0	0	0	0	0	0	0	0	0	0
117	2000/16	0	0	0	0	0	0	0	0	0	1	0	0	0	0	1	0	0	0
118	2000/17	1	0	0	0	0	1	0	0	0	1	0	0	1	0	0	1	1	0
119	2000/18	1	0	0	0	0	1	0	0	1	1	0	0	1	0	0	1	0	0
120	2000/19	0	0	0	0	0	0	0	0	0	0	0	0	0	0	0	0	0	0
122	2000/21	0	0	0	0	0	1	0	0	1	1	0	0	0	0	1	0	0	0
125	2000/23	1	0	0	0	0	0	0	0	0	0	0	0	1	0	1	1	0	0
126	2000/24	1	0	0	0	0	0	0	0	0	0	0	0	0	0	0	0	0	0
127	2000/25	0	0	0	0	0	0	0	0	0	0	0	0	0	0	1	0	0	0
128	2000/26	0	0	0	0	0	0	0	0	1	0	0	0	0	0	1	0	0	0
130	2000/28	0	0	0	0	0	1	0	0	0	0	0	0	1	0	1	1	0	0
131	2000/29	1	0	0	0	0	0	0	0	0	0	0	0	0	0	1	0	0	0
132	2000/30	0	0	0	0	0	0	0	0	1	0	0	0	0	0	0	1	0	0
134	2000/32	1	0	0	0	0	0	0	0	0	0	0	0	0	0	0	0	0	0
135	2000/33	1	0	0	0	0	0	0	0	0	0	0	0	0	0	0	0	0	0
136	2000/34	1	0	0	1	0	0	0	0	0	0	0	0	1	0	1	0	0	0
138	2000/35	1	0	0	0	0	1	0	0	1	1	0	0	1	0	1	0	0	0
141	2000/38	0	0	0	0	0	0	0	0	0	0	0	0	0	0	0	0	0	0
142	2000/39	1	0	0	0	0	1	0	0	0	1	0	0	0	0	0	0	0	0
146	2000/43	0	0	0	1	0	0	0	0	0	0	0	0	1	0	1	0	0	0
147	2000/44	1	0	0	1	0	1	0	0	0	1	0	0	1	0	1	0	1	0
148	2000/45	1	0	0	0	0	1	0	0	0	0	0	0	0	0	1	0	0	0
149	2000/46	1	0	0	1	0	1	0	0	0	0	0	0	1	1	1	0	0	0
150	2000/47	1	0	0	0	0	0	0	0	0	0	0	0	0	0	0	0	0	0
151	2000/48	1	0	0	0	0	0	0	0	0	0	0	0	0	0	0	0	0	0
152	2000/49	0	0	0	0	0	0	0	0	0	0	0	0	0	0	0	0	0	0
153	2000/50	1	0	0	1	0	1	0	0	0	0	0	0	0	0	1	0	0	0
157	2000/54	1	0	0	0	0	0	0	0	0	0	0	0	1	0	0	0	0	0
158	2000/55	1	0	0	0	0	1	0	0	1	0	0	0	1	0	1	0	0	0
159	2000/56	1	0	0	0	0	1	0	0	0	1	0	0	1	0	1	0	0	0
161	2000/58	1	0	0	0	0	0	0	0	0	0	0	0	1	0	1	0	0	0
164	2000/61	1	0	0	0	0	0	0	0	0	0	0	0	1	0	0	0	0	0
165	2001/002	0	0	0	0	0	0	0	0	0	0	0	0	1	0	1	0	0	0
168	2001/005	0	0	0	0	0	1	0	0	0	0	0	0	0	0	0	0	0	0
169	2001/006	0	0	0	0	0	0	0	0	0	0	0	0	0	0	1	0	0	0
170	2001/007	0	0	0	0	0	1	0	0	0	0	0	0	0	0	0	0	0	0
172	2001/009	1	0	0	0	0	0	0	0	0	0	0	0	0	0	1	0	0	0
173	2001/010	1	0	0	0	0	1	0	0	0	0	0	0	0	0	1	0	0	0
174	2001/011	1	0	0	0	0	1	0	0	0	1	0	0	1	0	1	0	0	0
176	2001/013	0	0	0	0	0	0	0	0	0	0	0	0	0	0	1	0	0	0
177	2001/014	1	0	0	0	0	0	0	0	1	0	0	0	0	1	1	0	0	0
178	2001/015	1	0	1	0	0	0	0	0	0	0	0	0	1	0	1	0	0	0
181	2001/018	1	0	0	0	0	1	0	0	1	0	0	0	1	0	1	0	0	0
182	2001/019	1	0	0	0	0	1	0	0	0	0	0	0	0	0	1	0	0	0
183	2001/020	1	0	0	0	0	0	0	0	0	1	0	0	1	0	0	0	0	0
187	2001/024	1	0	0	0	0	0	0	0	0	0	0	0	1	0	1	0	0	0
188	2001/025	1	0	0	0	0	0	0	0	0	0	0	0	0	1	1	0	0	0
189	2001/026	1	0	0	0	0	0	0	0	0	1	0	0	1	1	1	0	0	0
190	2001/027	1	0	0	0	0	0	0	0	1	0	0	0	1	0	0	0	0	0
191	2001/028	1	0	0	0	0	0	0	0	0	0	0	0	0	0	0	0	0	0
194	2001/031	1	0	0	0	0	0	0	0	1	1	0	0	1	1	1	0	0	0
196	2001/033	1	0	0	0	0	0	0	0	1	0	0	0	1	1	1	0	0	0
197	2001/034	0	0	0	0	0	0	0	0	0	0	0	0	0	0	0	0	0	0
199	2001/036	1	0	0	0	0	0	0	0	0	0	0	0	0	1	1	0	0	0
200	2001/037	0	0	0	0	0	1	0	0	0	0	0	0	0	1	1	0	0	0
201	2001/038	0	0	0	0	0	0	0	0	0	0	0	0	1	1	1	0	0	0
202	2001/039	1	0	0	0	0	0	0	0	0	0	0	0	0	0	0	0	0	0
203	2001/040	0	0	0	0	0	0	0	0	0	0	0	0	0	1	1	0	0	0
206	2001/042	0	0	0	0	0	0	0	0	0	0	0	0	0	0	0	0	0	0
208	2001/044	1	0	0	0	0	0	0	0	0	0	0	0	0	1	1	0	0	0
209	2001/045	1	0	0	0	0	1	0	0	1	0	0	0	0	1	1	0	0	0
216	2002/095	1	0	0	0	0	1	0	0	0	0	0	0	0	0	1	0	0	0
218	2002/097	1	0	0	0	0	1	0	0	1	0	0	0	1	1	1	0	0	0
219	2002/098	1	0	0	0	0	0	0	0	0	0	0	0	1	0	0	0	0	0
223	2002/102	0	0	0	0	0	1	0	0	0	0	0	0	0	0	0	0	0	0
226	2002/105	1	0	0	0	0	1	0	0	0	0	0	0	1	1	1	1	0	0
227	2002/106	0	0	0	0	0	0	0	0	1	0	0	0	0	0	0	0	0	0
228	2002/107	1	0	0	0	0	0	0	0	0	0	0	0	0	1	1	0	0	0
229	2002/108	1	0	0	0	0	0	0	0	1	0	0	0	0	1	1	0	1	0
230	2002/109	1	0	0	0	0	0	0	0	0	0	0	0	1	0	1	1	0	0
232	2002/111	1	0	0	0	0	1	0	0	0	0	0	0	0	0	1	0	0	0
233	2002/112	1	0	0	0	0	1	0	0	0	0	0	0	1	1	1	1	0	0
237	2002/116	0	0	0	0	0	0	0	0	0	0	0	0	0	1	1	0	0	0
244	2002/123	0	0	0	0	0	1	0	0	1	0	0	0	0	0	1	0	0	0
253	2002/132	1	0	0	0	0	1	0	1	1	0	0	0	1	0	1	0	0	0
256	2002/135	0	0	0	0	0	1	0	0	0	0	0	0	0	0	0	0	0	0
257	2002/136	0	0	0	0	0	1	0	0	0	0	0	0	0	0	1	0	0	0
260	2002/139	0	0	0	0	0	1	0	0	0	1	0	0	1	1	1	0	0	0
263	2002/142	1	0	0	0	0	0	0	0	1	0	0	0	1	0	0	0	0	0
264	2002/143	1	0	0	0	0	0	0	0	0	0	0	0	1	0	0	0	0	0
265	2002/144	1	0	0	0	0	1	0	0	0	0	0	0	1	1	0	0	0	0
267	2002/146	1	0	0	0	0	1	0	0	0	0	0	0	0	0	1	0	0	0
268	2002/147	1	0	0	0	0	1	0	0	1	0	0	0	0	0	1	1	0	0
269	2002/148	0	0	0	0	0	0	0	0	0	0	0	0	1	0	1	0	0	0
270	2003/013	0	0	0	0	0	0	0	0	0	0	0	0	0	0	0	0	0	0
272	2003/015	1	0	0	0	0	1	0	0	1	1	0	0	0	0	0	0	0	0
277	2003/020	0	0	0	0	0	0	0	0	0	0	0	0	0	0	1	0	0	0
278	2003/021	1	0	0	0	0	0	0	0	0	0	0	0	0	0	1	0	0	0
281	2003/023	1	0	0	0	0	1	0	0	1	1	0	0	1	1	1	1	0	0
286	2003/028	0	0	0	0	0	1	0	0	0	0	0	0	0	0	0	0	0	0
289	2003/030	0	0	0	0	0	0	0	0	0	0	0	0	0	0	0	0	0	0
293	2003/034	1	0	0	0	0	1	0	0	0	0	0	0	1	0	1	0	0	0
294	2003/035	1	0	0	0	0	0	0	0	0	0	0	0	1	0	1	0	0	0
295	2003/036	1	0	0	0	0	1	0	0	0	0	0	0	1	0	0	0	0	0
296	2003/037	1	0	0	0	0	1	0	0	0	0	0	0	1	0	0	0	0	0
297	2003/037B	0	1	0	0	0	0	1	0	1	0	0	0	1	0	0	0	0	0
302	2003/041	1	0	0	0	0	1	0	0	0	0	0	0	1	0	0	0	0	0
303	2003/042	1	0	0	0	0	1	0	0	0	1	0	0	1	1	1	0	0	0
305	2003/044	1	0	0	0	0	0	0	0	1	1	0	0	1	1	1	0	0	0
306	2003/045	0	0	0	0	0	0	0	0	0	0	0	0	1	0	1	0	0	0
308	2003/047	1	0	0	0	0	1	0	0	1	1	0	0	1	1	1	0	0	0
309	2003/048	1	0	0	0	0	1	0	0	0	0	0	0	0	0	0	0	0	0
310	2003/049	1	0	0	0	0	1	0	0	0	0	0	0	1	0	0	0	0	0
312	2003/050	1	0	0	0	0	1	0	0	1	1	0	0	0	0	1	0	0	1
313	2003/051	1	0	0	0	0	1	0	0	0	0	0	0	0	0	0	0	0	0
318	2003/056	1	0	0	0	0	0	0	0	1	0	0	0	1	0	1	1	0	0
319	2003/057	1	0	0	0	0	1	0	0	1	1	0	0	1	0	0	0	0	1
320	2003/058	1	0	0	0	0	0	0	0	0	0	0	0	1	0	0	0	0	0
327	2003/064	0	0	0	0	0	1	0	0	0	0	0	0	0	0	0	0	0	0
329	2003/066	0	0	0	0	0	0	0	0	0	0	0	0	1	0	1	0	0	0
330	2003/067	0	0	0	0	0	1	0	0	0	0	0	0	0	0	1	0	0	0
331	2003/068	1	0	0	0	0	1	0	0	0	1	0	0	1	0	0	0	0	0
332	2003/069	1	0	0	0	0	1	0	0	0	0	0	0	0	0	1	0	0	0
334	2003/071	0	0	0	0	0	0	0	0	0	0	0	0	0	1	0	0	0	0
335	2003/072	0	0	0	0	0	1	0	0	0	0	0	0	0	0	1	0	0	0
336	2003/073	0	0	0	0	0	0	0	0	0	0	0	0	1	0	1	0	0	0
339	2003/076	1	0	0	0	0	1	0	0	1	1	0	0	1	0	0	0	0	0
342	2003/078	1	0	0	0	0	1	0	0	0	0	0	0	1	1	0	0	0	0
343	2003/079	1	0	0	0	0	1	0	0	1	1	0	0	0	0	1	0	0	0
345	2003/081	1	0	0	0	0	0	0	0	1	0	0	0	0	0	1	0	0	0
346	2003/082	1	0	0	0	0	0	0	0	1	1	0	0	1	0	1	0	1	0
347	2003/083	0	0	0	0	0	0	0	0	0	0	0	0	0	0	1	0	0	0
348	2003/084	1	0	0	0	0	0	0	0	0	0	0	0	1	0	1	0	0	0

site_no	Chondrinidae	Lauriidae	Orculidae	Pupillidae	Pyramidulidae	Strobilopsidae	Valloniidae	Vertiginidae	Succineidae	Oleacinidae	Zonitidae	Clausiliidae	Agathylla	Albinaria	Alopia	Carinigera	Inchoatia	Cochlodina	Delima	Dilataria	Herilla	Isabellaria
3	1	0	0	0	0	0	0	0	0	1	0	0	0	0	0	0	0	0	1	0	0	0
16	0	0	0	0	0	0	0	0	0	0	0	0	0	0	0	0	0	0	0	0	0	0
19	0	0	0	0	0	0	0	0	0	1	0	0	0	0	0	0	0	0	0	0	0	0
20	0	0	0	0	0	0	0	0	0	1	0	0	0	0	0	0	0	0	1	0	0	0
25	0	0	0	0	0	0	0	0	0	0	0	0	0	0	0	0	0	0	1	0	0	0
28	1	0	0	0	0	0	0	0	0	1	1	0	0	0	0	0	0	0	1	0	0	0
33	0	0	0	0	0	0	0	0	0	0	0	0	0	0	0	0	0	0	1	0	0	0
34	1	0	0	0	0	0	0	0	0	0	0	0	0	0	0	0	0	0	0	0	0	0
36	1	0	0	0	0	0	0	0	0	0	0	0	0	0	0	0	0	0	0	0	0	0
38	0	0	0	0	0	0	0	0	0	0	1	0	0	0	0	0	0	0	0	0	0	0
39	0	0	0	0	0	0	0	0	0	0	1	1	0	0	0	0	0	0	0	0	0	0
41	1	0	0	0	0	0	0	0	0	0	0	0	0	0	0	0	0	0	0	0	1	0
42	0	0	0	0	0	0	0	0	0	0	0	1	0	0	0	0	0	1	0	0	0	0
43	1	0	0	0	0	0	0	0	0	0	0	0	0	0	0	0	0	0	0	0	1	0
46	1	0	0	0	0	0	0	0	0	0	0	1	0	0	0	0	0	0	1	0	1	0
48	0	0	0	0	0	0	0	0	0	0	1	0	0	0	0	0	0	0	1	0	0	0
52	0	0	0	0	0	0	0	0	0	0	0	0	0	0	0	0	0	0	0	0	0	0
53	1	0	0	0	0	0	0	0	0	1	0	0	0	0	0	0	0	0	0	0	0	0
54	0	0	0	0	0	0	0	0	0	0	0	0	0	0	0	0	0	0	0	0	0	0
57	0	0	0	0	0	0	0	0	0	0	0	0	0	0	0	0	0	0	1	0	0	0
58	0	0	0	0	0	0	0	0	0	1	0	0	0	0	0	0	0	0	1	0	0	0
59	0	0	0	0	0	0	0	0	0	0	1	0	0	0	0	0	0	0	0	0	0	0
60	0	0	0	0	0	0	0	0	0	1	0	0	0	0	0	0	0	0	1	0	0	0
61	1	0	1	0	1	0	0	0	0	1	0	0	0	0	0	0	0	0	1	0	0	0
62	0	0	0	0	0	0	0	0	0	1	0	0	0	0	0	0	0	0	1	0	0	0
64	1	0	0	0	0	0	0	0	0	1	0	0	0	0	0	0	0	0	1	0	0	0
66	1	0	0	0	0	0	0	0	0	0	1	0	0	0	0	0	0	0	1	0	0	0
70	1	0	0	0	0	0	0	0	0	1	1	0	0	0	0	0	0	0	0	0	0	0
73	0	0	0	0	0	0	0	0	0	0	0	0	0	1	0	0	0	0	0	0	0	0
74	1	0	0	0	0	0	0	0	0	1	1	0	0	0	0	0	0	0	0	0	0	0
75	0	0	0	0	0	0	0	0	0	0	0	0	0	0	0	0	0	0	0	0	0	0
77	1	0	0	0	0	0	0	0	0	1	0	0	0	1	0	0	0	0	0	0	0	0
78	0	0	0	0	0	0	0	0	0	0	0	0	0	0	0	0	0	0	0	0	0	0
79	0	0	0	0	0	0	0	0	0	1	0	0	0	1	0	0	0	0	0	0	0	0
82	1	0	0	0	1	0	0	0	0	1	0	0	0	1	0	0	0	0	0	0	0	0
83	1	0	0	0	1	0	0	0	0	0	0	0	1	0	0	0	0	0	0	0	0	0
84	0	0	0	0	1	0	0	0	0	1	0	0	0	0	0	0	0	0	0	0	0	0
85	1	0	0	0	1	0	0	0	0	1	0	0	0	0	0	0	0	0	0	0	0	0
88	1	0	0	0	0	0	0	0	0	0	0	0	0	0	0	0	0	0	0	0	0	0
89	0	0	0	0	0	0	0	0	0	0	0	0	0	0	0	0	0	0	0	0	0	0
92	0	0	0	0	0	0	0	0	0	0	0	0	0	0	0	1	0	0	0	0	0	0
94	0	0	0	0	0	0	0	0	0	1	0	0	0	0	0	0	0	0	0	0	0	0
95	0	0	0	0	0	0	0	0	0	0	0	0	0	0	0	0	0	0	0	0	0	0
96	0	0	0	0	0	0	0	0	0	1	0	1	0	1	0	0	0	0	0	0	0	0
97	1	0	0	0	0	0	0	0	0	1	0	0	0	1	0	0	0	0	0	0	0	0
98	0	0	0	0	0	0	0	0	0	0	0	0	0	1	0	0	0	0	0	0	0	0
100	1	0	0	0	0	0	0	0	0	1	0	0	0	0	0	0	0	0	0	0	0	0
102	1	0	0	0	0	0	0	0	0	1	0	0	0	1	0	0	0	0	0	0	0	0
103	1	0	0	0	0	0	0	0	0	1	0	0	0	0	0	0	1	0	0	0	0	0
105	1	0	1	0	1	0	0	0	0	0	0	0	0	0	0	0	0	0	0	0	0	0
114	1	0	1	0	0	0	0	0	0	0	0	1	0	0	0	0	0	0	0	0	0	0
115	0	0	0	0	0	0	0	0	0	0	0	0	0	0	0	0	0	0	0	0	1	0
116	0	0	0	0	0	0	0	0	0	0	0	0	0	0	0	0	0	0	0	0	1	0
117	0	0	0	0	0	0	0	0	0	0	0	0	0	0	0	0	0	0	0	0	1	0
118	0	0	0	0	0	0	0	0	0	0	0	0	0	0	0	0	0	0	0	0	0	0
119	0	0	0	0	0	0	0	0	0	0	1	1	0	0	0	0	0	1	0	0	1	0
120	1	0	0	0	0	0	0	0	0	0	1	0	0	0	0	0	0	0	1	0	0	0
122	1	0	0	0	0	0	0	0	0	0	0	0	0	0	0	0	0	0	0	0	1	0
125	0	0	0	0	0	0	0	0	0	0	0	1	0	0	0	0	0	1	0	0	1	0
126	1	0	0	0	0	0	0	0	0	0	0	1	0	0	0	0	0	0	0	0	1	0
127	0	0	0	0	0	0	0	0	0	0	0	0	0	0	0	0	0	0	0	0	1	0
128	1	0	0	0	0	0	0	0	0	0	1	1	0	0	0	0	0	0	0	0	1	0
130	0	0	0	0	0	0	0	0	0	0	0	0	0	0	0	0	0	1	0	0	0	0
131	1	0	0	0	0	0	0	0	0	0	0	0	0	0	0	0	0	0	0	0	0	0
132	0	0	0	0	0	0	0	0	0	0	0	1	0	0	0	0	0	0	0	0	0	0
134	0	0	0	0	0	0	0	0	0	0	0	0	0	0	0	0	0	0	0	0	0	0
135	0	0	0	0	0	0	0	0	0	0	0	0	0	0	0	0	0	0	0	0	0	0
136	0	0	0	0	0	0	0	0	0	0	1	0	0	0	0	0	0	0	1	0	0	0
138	1	0	0	0	0	0	0	0	0	1	1	0	0	0	0	0	0	0	1	0	0	0
141	0	0	0	0	1	0	0	0	0	0	0	0	0	0	0	0	0	0	0	0	0	0
142	0	0	0	0	0	0	0	0	0	0	0	0	0	0	0	0	0	0	1	0	0	0
146	0	1	0	0	0	0	0	0	0	1	0	0	0	0	0	0	0	0	1	0	0	0
147	1	0	1	0	0	0	1	1	0	1	1	0	0	0	0	0	0	0	1	0	0	0
148	1	0	0	0	0	0	0	0	0	1	1	0	0	0	0	0	0	0	0	0	0	0
149	1	0	0	0	0	0	0	0	0	1	1	0	0	0	0	0	0	0	1	0	0	0
150	1	0	0	0	0	0	0	0	0	0	1	0	0	0	0	0	0	0	1	0	0	0
151	0	0	0	0	0	0	0	0	0	0	0	0	0	0	0	0	0	0	0	0	0	0
152	0	0	0	0	0	0	0	0	0	0	0	0	0	0	0	0	0	0	1	0	0	0
153	0	0	0	0	0	0	0	0	0	1	0	0	0	0	0	0	0	0	1	0	0	0
157	1	0	0	0	0	0	0	0	0	1	1	0	0	0	0	0	0	0	0	0	0	0
158	1	0	0	0	0	0	0	0	0	0	1	0	1	0	0	0	0	0	0	0	0	0
159	1	0	1	0	0	0	0	0	0	0	1	0	0	0	0	0	0	0	1	0	0	0
161	0	0	0	0	0	0	0	0	0	0	0	0	1	0	0	0	0	0	0	0	0	0
164	0	0	0	0	0	0	0	0	0	0	1	0	0	0	0	0	0	0	0	0	0	0
165	1	0	0	0	0	0	0	0	0	0	0	1	0	0	0	0	0	0	0	0	0	0
168	1	0	0	0	0	0	0	0	0	0	0	0	0	0	0	0	0	0	0	0	0	0
169	0	0	0	0	0	0	0	0	0	0	0	0	0	0	0	0	0	0	0	0	0	0
170	0	0	0	0	0	0	0	0	0	0	0	1	0	0	0	0	0	0	0	0	0	0
172	0	0	0	0	0	0	0	0	0	0	0	0	0	0	0	0	0	0	1	0	0	0
173	0	0	0	0	0	0	0	0	0	1	1	0	0	0	0	0	0	0	0	0	0	0
174	0	0	0	0	0	0	0	0	0	1	1	0	0	0	0	0	0	0	1	0	0	0
176	1	0	0	0	0	0	0	0	0	0	0	0	0	0	0	0	0	0	1	0	0	0
177	1	0	0	0	0	0	0	0	0	0	0	0	1	0	0	0	0	0	1	0	0	0
178	1	0	0	0	1	0	0	0	0	1	0	0	1	0	0	0	0	0	1	0	0	0
181	1	0	0	0	0	0	0	0	0	1	0	0	0	0	0	0	0	0	0	0	0	0
182	1	0	0	0	0	0	0	0	0	0	1	0	0	0	0	0	0	0	0	0	0	0
183	1	0	0	0	0	0	0	0	0	1	0	0	0	0	0	0	0	0	1	0	0	0
187	0	0	0	0	0	0	0	0	0	0	0	0	0	0	0	0	0	0	1	0	0	0
188	0	0	0	0	0	0	0	0	0	0	0	0	0	0	0	0	0	0	1	0	0	0
189	0	0	0	0	0	0	0	0	0	0	0	0	0	0	0	0	0	0	1	0	0	0
190	0	0	0	0	0	0	0	0	0	1	1	0	0	0	0	0	0	0	0	0	0	0
191	1	0	0	0	1	0	0	0	0	0	0	0	0	0	0	0	0	0	0	0	0	0
194	1	0	0	0	1	0	0	0	0	1	0	0	1	0	0	0	0	0	0	0	0	0
196	1	0	0	0	1	0	0	0	0	0	0	0	1	0	0	0	0	0	0	0	0	0
197	0	0	0	0	0	0	0	0	0	0	0	0	0	0	0	0	0	0	0	0	0	0
199	0	0	0	0	1	0	0	0	0	1	0	0	0	0	0	0	0	0	0	0	0	0
200	0	0	0	0	0	0	0	0	0	0	0	0	0	0	0	0	0	0	0	0	0	0
201	1	0	0	0	0	0	0	0	0	1	0	0	0	0	0	0	0	0	0	0	0	0
202	1	0	0	0	0	0	0	0	0	0	0	0	1	0	0	0	0	0	0	0	0	0
203	1	0	0	0	0	0	0	0	0	1	0	0	0	0	0	0	0	0	0	0	0	0
206	0	0	0	0	1	0	0	0	0	0	0	0	0	1	0	0	0	0	0	0	0	0
208	1	0	0	0	0	0	0	0	0	0	0	0	0	0	0	0	0	0	0	0	0	0
209	0	0	0	0	1	0	0	0	0	0	0	0	0	1	0	0	0	0	0	0	0	0
216	0	0	0	0	0	0	0	0	0	0	1	0	0	0	0	0	0	0	1	0	0	0
218	1	0	1	0	0	0	1	0	0	1	1	1	0	0	0	0	0	0	1	0	0	0
219	0	0	1	0	0	0	1	0	0	0	0	0	0	0	0	0	0	0	1	0	0	0
223	0	0	0	0	0	0	0	0	0	0	0	0	0	0	0	0	0	0	1	0	0	0
226	1	0	1	0	1	0	0	0	0	0	1	0	0	0	0	0	0	0	0	0	0	0
227	0	0	0	0	0	0	0	0	0	0	0	0	0	0	0	0	0	0	1	0	0	0
228	1	0	0	0	0	0	0	0	0	1	0	0	0	0	0	0	0	0	0	0	0	0
229	1	0	1	0	0	0	0	0	0	1	0	0	0	0	0	0	0	0	0	0	0	0
230	1	0	1	0	0	0	0	0	0	0	0	0	0	0	0	0	0	0	0	0	0	0
232	1	0	1	0	1	0	0	0	0	1	0	0	0	0	0	0	0	0	0	0	0	0
233	1	0	0	0	1	0	0	0	0	0	0	0	0	0	0	0	0	0	1	0	0	0
237	0	0	0	0	0	0	0	0	0	0	0	0	0	0	0	0	0	0	0	0	0	0
244	1	0	0	0	0	0	0	0	0	1	0	0	0	0	0	0	0	0	0	0	0	0
253	0	0	0	0	0	0	0	0	1	0	0	0	0	0	0	0	0	0	0	0	0	0
256	0	0	0	0	0	0	0	0	0	0	0	0	0	0	0	0	0	0	0	0	0	0
257	1	0	0	0	0	0	0	0	0	0	0	0	0	0	0	0	0	0	0	0	0	0
260	1	0	0	0	0	0	0	0	0	1	1	0	0	0	0	0	0	0	0	0	0	0
263	1	0	1	0	1	0	0	0	0	1	1	0	0	0	0	0	0	0	0	0	0	0
264	0	0	0	0	0	0	0	0	0	0	0	0	0	0	0	0	0	0	0	0	0	0
265	0	0	0	0	0	0	0	0	0	0	0	0	0	0	0	0	0	0	0	0	0	0
267	0	0	0	0	0	0	0	0	0	1	1	0	0	0	0	0	0	0	1	0	0	0
268	0	0	0	0	1	0	0	0	0	1	1	1	0	0	0	0	0	0	1	0	0	0
269	1	0	0	0	0	0	0	0	0	0	0	1	0	0	0	0	0	1	0	0	0	0
270	0	0	0	0	0	0	0	0	0	0	0	0	0	0	0	0	0	0	0	0	0	0
272	0	0	0	0	1	0	0	0	0	1	1	0	0	0	0	0	0	0	0	0	0	0
277	0	0	0	0	0	0	0	0	0	0	0	0	0	0	0	0	0	0	0	0	0	0
278	0	0	0	0	0	0	0	0	0	0	0	0	0	0	0	0	0	0	0	0	0	0
281	1	0	1	0	0	0	0	0	0	1	0	0	0	0	0	0	0	0	1	0	0	0
286	1	0	0	0	1	0	0	0	0	0	0	0	0	0	0	0	0	0	0	0	0	0
289	1	0	0	0	0	0	0	0	0	0	0	0	0	0	0	0	0	0	0	0	0	0
293	1	0	0	0	1	0	0	0	0	1	0	0	0	0	0	0	0	0	1	0	0	0
294	0	0	0	0	0	0	0	0	0	0	0	0	0	0	0	0	0	0	0	0	0	0
295	1	0	0	0	0	0	0	0	0	1	0	0	0	0	0	0	0	0	0	0	0	0
296	1	0	0	0	1	0	0	0	0	1	0	0	0	0	0	0	0	0	0	0	0	0
297	0	0	1	0	1	0	1	1	0	0	0	0	0	0	0	0	0	0	0	0	0	0
302	1	0	0	0	1	0	0	0	0	0	0	0	0	0	0	0	0	0	0	0	0	0
303	1	0	0	0	1	0	0	0	0	1	1	0	1	0	0	0	0	0	0	0	0	0
305	1	0	0	0	1	0	0	0	0	1	0	0	0	0	0	0	0	0	0	0	0	0
306	0	0	0	0	0	0	0	0	0	0	0	0	0	0	0	0	0	0	0	0	0	0
308	1	0	0	0	0	0	0	0	0	1	0	0	0	0	0	0	0	0	0	0	0	0
309	1	0	0	0	1	0	0	0	0	0	0	0	0	0	0	0	0	0	0	0	0	0
310	0	0	0	0	1	0	0	0	0	0	0	0	0	0	0	0	0	0	0	0	0	0
312	1	0	0	0	0	0	0	0	0	0	0	0	0	0	0	0	0	0	0	0	0	0
313	1	0	0	0	0	0	0	0	0	0	0	0	0	0	0	0	0	0	0	0	0	0
318	1	0	0	0	1	0	0	0	0	0	1	0	0	0	0	0	0	0	0	0	0	0
319	1	0	0	0	0	0	0	0	0	0	1	0	0	0	0	0	0	0	0	0	0	0
320	1	0	1	0	0	0	0	0	0	0	0	0	0	0	0	0	0	0	0	0	0	0
327	0	0	0	0	0	0	0	0	0	0	0	0	0	0	0	0	0	0	0	0	0	0
329	1	0	0	0	0	0	0	0	0	0	0	0	0	0	0	0	0	0	0	0	0	0
330	1	0	0	0	0	0	0	0	0	0	0	0	0	0	0	0	0	0	0	0	0	0
331	1	0	0	0	1	0	0	0	0	0	0	0	0	0	0	0	0	0	0	0	0	0
332	1	0	0	0	0	0	0	0	0	0	0	0	0	0	0	0	0	0	0	0	0	0
334	1	0	0	0	0	0	0	0	0	0	0	0	0	0	0	0	0	0	0	0	0	0
335	0	0	0	0	0	0	0	0	0	0	0	0	0	0	0	0	0	0	0	0	0	0
336	1	0	0	0	1	0	0	0	0	0	0	0	1	0	0	0	0	0	0	0	0	0
339	1	0	0	0	1	0	0	0	0	0	0	0	1	0	0	0	0	0	0	0	0	0
342	1	0	0	0	0	0	0	0	0	0	0	0	0	0	0	0	0	0	0	0	0	0
343	1	0	0	0	1	0	0	0	0	1	0	0	1	0	0	0	0	0	0	0	0	0
345	0	0	0	0	0	0	0	0	0	0	0	1	0	0	0	0	0	1	1	0	0	0
346	1	0	1	0	0	0	0	0	0	0	0	1	0	0	0	0	0	0	1	0	0	0
347	0	0	0	0	0	0	1	1	0	0	0	0	0	0	0	0	0	0	1	0	0	0
348	0	0	1	0	0	0	0	0	0	0	0	0	0	0	0	0	0	0	1	0	1	0

site_no	Macedonica	Medora	Papillifera	Charpentieria	Strigilodelima	Triloba	Mat	Mct	Mdo	Mde	Mfu	Mgm	M50	Mhe	Mhi	Mjn	Mlx	M17	Mmn	Mna	Mok	Mpe	Mx6	Mrl	Msk	Mso	Msp	Mst	M21	Msr	Mti	Mto	Mzi	habitat_type	Latitude	Longitude
3	0	1	0	0	0	0	0	0	0	0	0	0	0	1	0	0	0	0	0	0	0	0	0	0	0	0	0	0	0	0	0	0	0	1	41.518	19.8008
16	0	0	0	1	0	0	0	0	0	0	0	0	0	0	0	0	0	0	0	0	0	0	0	0	0	0	0	0	0	0	0	0	0	1	39.8644	20.0153
19	0	0	0	1	0	0	0	0	0	0	0	0	0	0	0	0	0	0	0	0	0	0	0	0	0	0	0	0	0	0	0	0	0	1	40.25030556	20.06522222
20	0	0	0	0	0	0	0	0	0	0	0	0	0	0	0	0	0	0	0	0	0	0	0	0	0	0	0	0	0	0	0	0	0	2	41.35	19.89
25	0	0	0	0	0	0	0	0	0	0	0	0	0	1	0	0	0	0	0	0	0	0	0	0	0	0	0	0	0	0	0	0	0	1	41.5085	19.7961
28	0	0	0	1	1	0	0	0	0	0	0	0	0	0	0	0	0	0	0	0	0	0	0	0	0	0	0	0	0	0	0	0	0	1	41.3603	19.8916
33	0	0	0	0	0	0	0	0	0	0	0	0	0	0	0	0	0	0	0	0	0	0	0	0	0	0	0	0	0	0	0	0	0	1	41.8677	19.5665
34	0	0	0	0	0	0	0	0	0	0	0	0	0	0	0	0	0	0	0	0	0	0	0	0	0	0	0	0	0	0	0	0	0	1	43.9511	19.989
36	0	0	0	0	0	0	0	0	0	0	0	0	0	0	0	0	0	0	0	0	0	0	0	0	0	0	0	0	0	0	0	0	0	1	43.5988	19.7196
38	0	0	0	0	0	0	0	0	0	0	0	0	0	0	0	0	0	0	0	0	0	0	0	0	0	0	0	0	0	0	0	0	0	1	43.36	19.58
39	0	0	0	0	0	0	0	0	0	0	0	0	0	0	0	0	0	0	0	0	0	0	0	0	0	0	0	0	0	0	0	0	0	1	43.34	19.31
41	0	0	0	0	0	0	0	0	0	0	0	0	0	0	0	0	0	0	0	0	0	0	0	0	0	0	0	0	0	0	0	0	0	1	43.02238333	19.15628333
42	0	0	0	0	0	0	0	0	0	0	0	0	0	0	0	0	0	0	0	0	0	0	0	0	0	0	0	0	0	0	0	0	0	1	43.0003	19.1601
43	0	0	0	0	0	0	0	0	0	0	0	0	0	0	0	0	0	0	0	0	0	0	0	0	0	0	0	0	0	0	0	0	0	1	42.9656	19.0952
46	0	0	0	0	0	0	0	0	0	0	0	0	0	0	0	0	0	0	0	0	0	0	0	0	0	0	0	0	0	0	0	0	0	1	42.94591667	19.08355
48	0	0	0	0	0	0	0	0	0	0	0	0	0	0	0	0	0	0	0	0	0	0	0	0	0	0	0	0	0	0	0	0	0	1	42.8981	19.0534
52	0	0	0	1	0	0	0	0	0	0	0	0	0	0	0	0	0	0	0	0	0	0	0	0	0	0	0	0	0	0	0	0	0	1	42.5008	18.6927
53	0	0	0	0	0	0	0	1	0	0	0	0	0	0	0	0	0	0	0	0	0	0	0	0	0	0	0	0	0	0	0	0	0	1	42.4213	18.7716
54	0	0	0	0	0	0	0	0	0	0	0	0	0	0	0	0	0	0	0	0	0	0	0	0	0	0	0	0	0	0	0	0	0	2	42.1947	18.967
57	0	0	0	0	0	0	0	0	0	0	0	0	0	0	0	0	0	0	0	0	0	0	0	0	0	0	0	0	0	0	0	0	0	1	42.2715	19.1254
58	0	0	0	0	0	0	0	0	0	0	0	0	0	0	0	0	0	0	0	0	0	0	0	0	0	0	0	0	0	1	0	0	0	1	42.3732	19.3193
59	0	0	0	0	0	0	0	0	0	0	0	0	0	0	0	0	0	0	0	0	0	0	0	0	0	0	0	0	0	1	0	0	0	1	42.3193	19.3696
60	0	0	0	0	0	0	0	0	0	0	0	0	0	0	0	0	0	0	0	0	0	0	0	0	0	0	0	0	0	0	0	0	0	1	42.3741	19.4647
61	0	0	0	1	0	0	0	0	0	0	0	0	0	0	0	0	0	0	0	0	0	0	0	0	0	0	0	0	0	0	0	0	0	1	42.41333333	19.50385
62	0	0	0	0	0	0	0	0	0	0	0	0	0	0	0	0	0	0	0	0	0	0	0	0	0	0	0	0	0	0	0	0	0	1	42.3315	19.6005
64	0	0	0	0	0	0	0	0	0	0	0	0	0	0	0	0	0	0	0	0	0	0	0	0	0	0	0	0	0	1	0	0	0	1	42.048	19.4976
66	0	0	0	1	0	0	0	0	0	0	0	0	0	0	0	0	0	0	0	0	0	0	0	0	0	0	0	0	0	1	0	0	0	1	41.90006667	19.51298333
70	0	1	0	0	1	0	0	0	0	0	0	0	0	0	0	0	0	0	0	0	0	0	0	0	0	0	0	0	0	0	0	0	0	1	41.3599	19.9146
73	0	0	0	0	0	0	0	0	0	0	0	0	0	0	0	0	0	0	0	0	0	0	0	0	0	0	0	0	0	0	0	0	0	1	40.2627	19.5315
74	0	0	0	0	0	0	0	0	0	0	0	0	0	0	0	0	0	0	0	0	0	0	0	0	0	0	0	0	0	0	0	0	0	3	40.2055	19.591
75	0	0	0	0	0	0	0	0	0	0	0	0	0	0	0	0	0	0	0	0	0	0	0	0	0	0	0	0	0	0	0	0	0	1	40.1904	19.6049
77	0	0	0	1	0	0	0	0	0	0	0	0	0	0	0	0	0	0	0	0	0	0	0	0	0	0	0	0	0	0	0	0	0	1	40.069	19.8588
78	0	0	0	1	0	0	0	0	0	0	0	0	0	0	0	0	0	0	0	0	0	0	0	0	0	0	0	0	0	0	0	0	0	1	39.9195	20.1404
79	0	0	0	1	0	0	0	0	0	0	0	0	0	0	0	0	0	0	0	0	0	0	0	0	0	0	0	0	0	0	0	0	0	1	39.9479	20.25
82	0	0	0	1	1	0	0	0	0	0	0	0	0	0	0	0	0	0	0	0	0	0	0	1	0	0	0	0	0	0	0	0	0	1	40.2086	20.4147
83	0	0	0	0	1	0	0	0	0	0	0	0	0	0	0	0	0	0	0	0	0	0	0	0	0	0	0	0	0	0	0	0	0	1	40.1608	20.5682
84	0	0	0	0	0	0	0	0	0	0	0	0	0	0	0	0	0	0	0	0	0	0	0	0	0	0	0	0	0	0	0	0	0	1	40.2441	20.6386
85	0	0	0	0	0	0	0	0	0	0	0	0	0	0	0	0	0	0	0	0	0	0	0	0	1	0	0	0	0	0	0	0	0	1	40.2656	20.59991667
88	0	0	0	0	1	0	0	0	0	0	0	0	0	0	0	0	0	0	0	0	0	0	0	0	0	0	0	0	0	0	0	0	0	1	40.9029	20.7298
89	0	0	0	0	0	0	0	0	0	0	0	0	0	0	0	0	0	0	0	0	0	0	0	0	0	0	0	0	0	0	0	0	0	1	43.6308	20.5434
92	0	0	0	0	0	0	0	0	0	0	0	0	0	0	0	0	0	0	0	0	0	0	0	0	0	0	0	0	0	0	0	0	0	1	40.44	21.34
94	0	0	0	0	0	0	0	0	0	0	0	0	0	0	0	0	0	0	0	0	0	0	0	0	0	0	0	0	0	0	0	0	0	1	39.5603	20.469
95	0	0	1	1	0	0	0	0	0	0	0	0	0	0	0	0	0	0	0	0	0	0	0	0	0	0	0	0	0	0	0	0	0	1	39.704	19.806
96	0	0	0	0	0	0	0	0	0	0	0	0	0	0	0	0	0	0	0	0	0	0	0	0	0	0	0	0	0	0	0	0	0	1	39.72	19.87
97	0	0	0	0	0	0	0	0	0	0	0	0	0	0	0	0	0	0	0	0	0	0	0	0	0	0	0	0	0	0	0	0	0	1	39.73	19.9
98	0	0	0	0	0	0	0	0	0	0	0	0	0	0	0	0	0	0	0	0	0	0	0	0	0	0	0	0	0	0	0	0	0	1	39.736	19.798
100	0	0	0	0	0	0	0	0	0	0	0	0	0	0	0	0	0	0	0	0	0	0	0	0	0	0	0	0	0	0	0	0	0	1	39.7054	19.7611
102	0	0	0	0	0	0	0	0	0	0	0	0	0	0	0	0	0	0	0	0	0	0	0	0	0	0	0	0	0	0	0	0	0	1	39.6436	20.3133
103	0	0	0	0	0	0	0	0	0	0	0	0	0	0	0	0	0	0	0	0	0	0	0	0	0	0	0	0	0	0	0	0	0	1	39.5	20.52
105	0	0	0	0	1	0	0	0	0	0	0	0	0	0	0	0	0	0	0	0	0	0	0	0	0	0	0	0	0	0	0	0	0	1	39.866	20.717
114	0	0	0	0	0	0	0	0	0	0	0	0	0	0	0	0	0	0	0	0	0	0	0	0	0	0	0	0	0	0	0	0	0	1	41.7	20.742
115	0	0	0	0	0	0	0	0	0	0	0	0	0	0	0	0	0	0	0	0	0	0	0	0	0	0	0	0	0	0	0	0	0	1	43.28956667	18.85161667
116	0	0	0	0	0	0	0	0	0	0	0	0	0	0	0	0	0	0	0	0	0	0	0	0	0	0	0	0	0	0	0	0	0	1	43.2737	18.8428
117	0	0	0	0	0	0	0	0	0	0	0	0	0	0	0	0	0	0	0	0	0	0	0	0	0	0	0	0	0	0	0	0	0	1	43.1717	18.8519
118	0	0	0	0	0	0	0	0	0	0	0	0	0	0	0	0	0	0	0	0	0	0	0	0	0	0	0	0	0	0	0	0	0	1	42.97508333	18.90563333
119	0	0	0	0	0	0	0	0	0	0	0	0	0	0	0	0	0	0	0	0	0	0	0	0	0	0	0	0	0	0	0	0	0	1	42.8932	19.07138333
120	0	0	0	0	0	0	0	0	0	0	0	0	0	0	0	0	0	0	0	0	0	0	0	0	0	0	0	0	0	0	0	0	0	1	42.92766667	19.0712
122	0	0	0	0	0	0	0	0	0	0	0	0	0	0	0	0	0	0	0	0	0	0	0	0	0	0	0	0	0	0	0	0	0	1	42.9424	19.10423333
125	0	0	0	0	0	0	0	0	0	0	0	0	0	0	0	0	0	0	0	0	0	0	0	0	0	0	0	0	0	0	0	0	0	1	43.151	19.27883333
126	0	0	0	0	0	0	0	0	0	0	0	0	0	0	0	0	0	0	0	0	0	0	0	0	0	0	0	0	0	0	0	0	0	1	43.1358	19.2999
127	0	0	0	0	0	0	0	0	0	0	0	0	0	0	0	0	0	0	0	0	0	0	0	0	0	0	0	0	0	0	0	0	0	1	43.1241	19.3246
128	0	0	0	0	0	0	0	0	0	0	0	0	0	0	0	0	0	0	0	0	0	0	0	0	0	0	0	0	0	0	0	0	0	1	43.0726	19.38336667
130	0	0	0	0	0	0	0	0	0	0	0	0	0	0	0	0	0	0	0	0	0	0	0	0	0	0	0	0	0	0	0	0	0	1	42.88908333	19.53473333
131	0	0	0	0	0	0	0	0	0	0	0	0	0	0	0	0	0	0	0	0	0	0	0	0	0	0	0	0	0	0	0	0	0	1	42.852	19.5271
132	0	0	0	0	0	0	0	0	0	0	0	0	0	0	0	0	0	0	0	0	0	0	0	0	0	0	0	0	0	0	0	0	0	3	42.7415	19.6898
134	0	0	0	0	0	0	0	0	0	0	0	0	0	0	0	0	0	0	0	0	0	0	0	0	0	0	0	0	0	0	0	0	0	1	42.707	19.3771
135	0	1	0	0	0	0	0	0	0	0	0	0	0	0	0	0	0	0	0	0	0	0	0	0	0	0	0	0	0	0	0	0	0	1	42.6908	19.3796
136	0	0	0	0	0	0	0	0	0	0	0	0	0	0	0	0	0	0	0	0	0	0	0	0	0	0	0	0	0	1	0	0	0	1	42.2436	19.0952
138	0	0	0	0	0	0	0	0	0	0	0	0	0	0	0	0	0	0	0	0	0	0	0	0	0	0	0	0	0	1	0	0	0	1	42.27651667	19.07088333
141	0	0	0	0	0	0	0	0	0	0	0	0	0	0	0	0	0	0	0	0	0	0	0	0	0	0	0	0	0	0	0	0	0	1	42.1487	19.12
142	0	0	0	0	0	0	0	0	0	0	0	0	0	0	0	0	0	0	0	0	0	0	0	0	0	0	0	0	0	1	0	0	0	1	42.141	19.1377
146	0	0	1	1	0	0	0	0	0	0	0	0	0	0	0	0	0	0	0	0	0	0	0	0	0	0	0	0	0	0	0	0	0	1	41.9247	19.2049
147	0	0	0	1	0	0	0	0	0	0	0	0	0	0	0	0	0	0	0	0	0	0	0	0	0	0	0	0	0	1	0	0	0	1	41.979361	19.259112
148	0	0	0	1	0	0	0	0	0	0	0	0	0	0	0	0	0	0	0	0	0	0	0	0	0	0	0	0	0	1	0	0	0	1	42.002	19.2639
149	0	0	0	0	0	0	0	0	0	0	0	0	0	0	0	0	0	0	0	0	0	0	0	0	0	0	0	0	0	1	0	0	0	1	41.9864	19.3156
150	0	0	0	1	0	0	0	0	0	0	0	0	0	0	0	0	0	0	0	0	0	0	0	0	0	0	0	0	0	0	0	0	0	1	42.04765	19.3229
151	0	0	0	0	0	0	0	0	0	0	0	0	0	0	0	0	0	0	0	0	0	0	0	0	0	0	0	0	0	0	0	0	0	1	42.0645	19.13193333
152	0	0	0	1	0	0	0	0	0	0	0	0	0	0	0	0	0	0	0	0	0	0	0	0	0	0	0	0	0	0	0	0	0	1	42.2048	18.9575
153	0	0	0	0	0	0	0	0	0	0	0	0	0	0	0	0	0	0	0	0	0	0	0	0	0	0	0	0	0	0	0	0	0	1	42.2766	18.8869
157	0	0	0	0	0	0	0	0	0	0	0	0	0	0	0	0	0	0	0	0	0	0	0	0	0	0	0	0	0	0	0	0	0	1	42.42026667	18.7938
158	0	1	0	0	0	0	0	0	0	0	0	0	0	0	0	0	0	0	0	0	0	0	0	0	0	0	0	0	0	0	0	0	0	1	42.42156667	18.79798333
159	0	0	0	0	0	0	0	0	0	0	0	0	0	0	0	0	0	0	0	0	0	0	0	0	0	0	0	0	0	1	0	0	0	1	42.434	18.8189
161	0	0	0	1	0	0	0	0	0	0	0	0	0	0	0	0	0	0	0	0	0	0	0	0	0	0	0	0	0	0	0	0	0	1	42.4744	18.5362
164	0	0	0	0	0	0	0	0	0	0	0	0	0	0	0	0	0	0	0	0	0	0	0	0	0	0	0	0	0	0	0	0	0	1	42.59	18.67
165	0	0	0	0	0	0	0	0	0	0	0	0	0	0	0	0	0	0	0	0	0	0	0	0	0	0	0	0	0	0	0	0	0	1	43.96716667	19.96116667
168	0	0	0	0	0	0	0	0	0	0	0	0	0	0	0	0	0	0	0	0	0	0	0	0	0	0	0	0	0	0	0	0	0	1	43.33	19.669
169	0	0	0	0	0	0	0	0	0	0	0	0	0	0	0	0	0	0	0	0	0	0	0	0	0	0	0	0	0	0	0	0	0	1	43.2659	19.7142
170	0	0	0	0	0	0	0	0	0	0	0	0	0	0	0	0	0	0	0	0	0	0	0	0	0	0	0	0	0	0	0	0	0	1	43.89483333	19.53866667
172	0	1	0	0	0	0	0	0	0	0	0	0	0	0	0	0	0	0	0	0	0	0	0	0	0	0	0	0	0	0	0	0	0	1	42.697	19.374
173	0	1	0	1	0	0	0	0	0	0	0	0	0	0	0	0	0	0	0	0	0	0	0	0	0	0	0	0	0	0	0	0	0	1	42.614	19.39
174	0	0	0	0	0	0	0	0	0	0	0	0	0	0	0	0	0	0	0	0	0	0	0	0	0	0	0	0	0	0	0	0	0	1	42.51733333	19.19566667
176	0	0	0	0	0	0	0	0	0	0	0	0	0	0	0	0	0	0	0	0	0	0	0	0	0	0	0	0	0	0	0	0	0	1	42.11816667	19.57583333
177	0	0	0	0	0	0	0	0	0	0	0	0	0	0	0	0	0	0	0	0	0	0	0	0	0	0	0	0	0	1	0	0	0	1	42.0446	19.4945
178	0	0	0	0	0	0	0	0	0	0	0	0	0	0	0	0	0	0	0	0	0	0	0	0	0	0	0	0	0	0	0	0	0	1	42.01583333	19.62183333
181	0	0	0	0	0	0	0	0	0	0	0	0	0	0	0	0	0	0	0	0	0	0	0	0	0	0	0	0	0	0	0	0	0	1	41.8895	20.12083333
182	0	0	0	0	0	0	0	0	0	0	0	0	0	0	0	0	0	0	0	0	0	0	0	0	0	0	0	0	0	0	0	0	0	1	41.92016667	20.14516667
183	0	0	0	0	0	0	0	0	0	0	0	0	0	0	0	0	0	0	0	0	0	0	0	0	0	0	0	0	0	0	0	0	0	1	41.76433333	19.7815
187	0	0	0	0	0	0	0	0	0	0	0	0	0	0	0	0	1	0	0	0	0	0	0	0	0	0	0	0	0	0	0	0	0	1	41.37963	19.8607
188	0	0	0	1	1	0	0	0	0	0	0	0	0	0	0	0	1	0	0	0	0	0	0	0	0	0	0	0	0	0	0	0	0	1	41.3859	19.861
189	0	0	0	0	0	0	0	0	0	0	0	0	0	0	0	0	1	0	0	0	0	0	0	0	0	0	0	0	0	0	0	0	0	1	41.39416667	19.88433333
190	0	1	0	0	0	0	0	0	0	0	0	0	0	0	0	0	1	0	0	0	0	0	0	0	0	0	0	0	0	0	0	0	0	1	41.40216667	19.90116667
191	0	1	0	0	0	0	0	0	0	0	0	0	0	0	0	0	0	0	0	0	0	0	0	0	0	0	0	0	0	0	0	0	0	1	41.35466667	19.916
194	0	1	0	0	1	0	0	0	0	0	0	0	0	0	0	0	0	0	0	0	0	0	0	0	0	0	0	0	0	0	0	0	0	1	41.23483333	19.97716667
196	0	0	0	0	1	0	0	0	0	0	0	0	0	0	0	0	0	0	0	0	0	0	0	0	0	0	0	0	0	0	0	0	0	1	40.4698	20.2561
197	0	0	0	0	1	0	0	0	0	0	0	0	0	0	0	0	0	0	0	0	0	0	0	0	0	0	0	0	0	0	0	0	0	1	40.49466667	20.2705
199	0	0	0	1	1	0	0	0	0	0	0	0	0	0	0	0	0	0	0	0	0	0	0	0	0	0	0	0	0	0	0	0	0	1	40.6972	19.9535
200	0	0	0	0	0	0	0	0	0	0	0	0	0	0	0	0	0	0	0	0	0	0	0	0	0	0	0	0	0	0	0	0	0	2	40.5865	19.99666667
201	0	0	0	0	1	0	0	0	0	0	0	0	0	0	0	0	0	0	0	0	0	0	0	0	0	0	0	0	0	0	0	0	0	1	40.542	20.007
202	0	0	0	0	1	0	0	0	0	0	0	0	0	0	0	0	0	0	0	0	0	0	0	0	0	0	0	0	0	0	0	0	0	1	40.50283333	19.9845
203	0	0	0	0	1	0	0	0	0	0	0	0	0	0	0	0	0	0	0	0	0	0	0	0	0	0	0	0	0	0	0	0	0	1	40.34783333	20.16016667
206	0	0	0	0	1	0	0	0	0	0	0	0	0	0	0	0	0	0	0	0	0	0	0	0	0	0	0	0	0	0	0	0	0	1	40.23683333	20.35183333
208	0	0	0	1	0	0	0	0	0	0	0	0	0	0	0	0	0	0	0	0	0	0	0	0	0	0	0	0	0	0	0	0	0	1	40.29366667	20.01716667
209	0	0	0	1	1	0	0	0	0	0	0	0	0	0	0	0	0	0	0	0	0	0	0	0	0	0	0	0	0	0	0	0	0	1	40.30133333	20.02166667
216	0	0	0	0	0	0	0	0	0	0	0	0	0	0	0	0	0	0	0	0	0	0	0	0	0	0	0	0	0	0	0	0	0	1	42.3201	19.58575
218	0	0	0	0	1	0	0	0	0	0	0	0	0	0	0	0	0	0	0	0	0	0	0	0	0	0	0	0	0	0	0	0	0	3	42.39205	19.70716667
219	0	0	0	0	0	0	0	0	0	0	0	0	0	0	0	0	0	0	0	0	1	0	0	0	0	0	0	0	0	0	0	0	0	1	42.39228333	19.7297
223	0	0	0	0	0	0	0	0	0	0	0	0	0	0	0	0	0	0	0	0	0	0	0	0	0	0	0	0	0	0	0	0	0	1	41.72	19.77
226	0	0	0	0	1	0	0	0	0	0	0	0	0	0	0	0	0	0	0	0	0	0	0	0	0	0	0	0	0	0	0	0	0	1	41.83253333	20.10056667
227	0	0	0	1	0	0	0	0	0	0	0	0	0	0	0	0	0	0	0	0	0	0	0	0	0	0	0	0	0	0	0	0	0	1	41.34731667	19.89703333
228	0	0	0	0	1	0	0	0	0	0	0	0	0	0	0	0	0	0	0	0	0	0	0	0	0	0	0	0	0	0	0	0	0	1	41.32651667	19.94403333
229	0	0	0	0	1	0	0	0	0	0	0	0	0	0	0	0	0	0	0	0	0	0	0	0	0	0	0	0	0	0	0	0	0	1	41.32681667	19.95755
230	0	0	0	0	1	0	0	0	0	0	0	0	0	0	0	0	0	0	0	0	0	0	0	0	0	0	0	0	0	0	0	0	0	1	41.34036667	19.95628333
232	0	0	0	0	1	0	0	0	0	0	0	0	0	0	0	0	0	0	0	0	0	0	0	0	0	0	0	0	0	0	0	0	0	1	41.37216667	20.005
233	0	0	0	0	1	0	0	0	0	0	0	0	0	0	0	0	0	0	0	0	0	0	0	0	0	0	0	0	0	0	0	0	0	1	41.35913333	20.0273
237	0	0	0	0	1	0	0	0	0	0	0	0	0	0	0	0	0	0	0	0	0	0	0	0	0	0	0	0	0	0	0	0	0	1	41.19851667	19.93473333
244	0	0	0	0	1	0	0	0	0	0	0	0	0	0	0	0	1	0	0	0	0	0	0	0	0	0	0	0	0	0	0	0	0	1	41.16566667	20.23773333
253	0	0	0	0	0	0	0	0	0	0	0	0	0	0	0	0	0	0	0	0	0	1	0	0	0	0	0	0	0	0	0	0	0	1	41.51596667	20.3906
256	0	0	0	0	1	0	0	0	0	0	0	0	0	0	0	0	0	0	0	0	0	0	0	0	0	0	0	0	0	0	0	0	0	1	41.42086667	20.47971667
257	0	0	0	0	1	0	0	0	0	0	0	0	0	0	0	0	0	0	0	0	0	1	0	0	0	0	0	0	0	0	0	0	0	1	41.41833333	20.45686667
260	0	0	0	0	0	0	0	0	0	0	0	0	0	0	0	0	0	0	0	0	0	0	0	0	0	0	0	0	0	0	1	0	0	1	41.47181667	20.0967
263	0	0	0	0	0	1	0	0	0	0	0	0	0	0	0	0	0	0	0	0	0	0	0	0	0	0	0	0	0	0	0	0	0	3	41.52085	19.8983
264	0	1	0	0	0	0	0	0	0	0	0	0	0	0	0	0	0	0	0	0	0	0	0	0	0	0	0	0	0	0	0	0	0	1	41.52058333	19.85606667
265	0	1	0	0	0	0	0	0	0	0	0	0	0	1	0	0	0	0	0	0	0	0	0	0	0	0	0	0	0	0	0	0	0	1	41.5229	19.81645
267	0	1	0	1	0	0	0	0	0	0	0	0	0	0	0	0	0	0	0	0	0	0	0	0	0	0	0	0	0	0	0	0	0	1	42.65166667	19.37838333
268	0	0	0	0	0	0	0	0	0	0	0	0	0	0	0	0	0	0	0	0	0	0	0	0	0	0	0	0	0	0	0	0	0	1	42.766	19.389
269	0	0	0	0	0	0	0	0	0	0	0	0	0	0	0	0	0	0	0	0	0	0	0	0	0	0	0	0	0	0	0	0	0	1	43.1552	19.78068333
270	0	0	0	0	0	0	0	0	0	0	0	0	0	0	0	0	0	0	0	0	0	0	0	0	0	0	0	0	0	0	0	0	0	1	43.97628333	19.9338
272	0	0	0	1	0	0	0	0	0	0	0	0	0	0	0	0	0	0	0	0	0	0	0	0	0	0	0	0	0	0	0	0	0	1	42.47108333	19.3651
277	0	0	0	0	0	0	0	0	0	0	0	0	0	0	0	0	0	0	0	0	0	0	0	0	0	0	0	0	0	0	0	0	0	1	42.06425	20.49298333
278	0	0	0	0	0	0	0	0	0	0	0	0	0	0	0	0	0	0	0	0	0	0	0	0	0	0	0	0	0	0	0	0	0	1	42.07275	20.4599
281	0	0	0	0	1	0	0	0	0	0	0	0	0	0	0	0	0	0	0	0	0	0	0	0	0	1	0	0	0	0	0	0	0	1	41.95983333	20.39375
286	0	0	0	0	0	0	0	0	0	0	0	0	0	0	0	0	0	0	0	0	0	0	0	0	0	0	0	0	0	0	0	0	0	1	41.63225	20.2217
289	0	0	0	0	1	1	0	0	0	0	0	0	0	0	0	0	0	0	0	0	0	0	0	0	0	0	0	0	0	0	0	0	0	1	41.64333333	20.15716667
293	0	0	0	0	0	0	0	0	0	0	0	0	0	0	0	0	0	0	0	0	0	0	0	0	0	0	0	0	0	0	0	0	0	1	41.69501667	19.81418333
294	0	0	0	0	0	0	0	0	0	0	0	0	0	0	0	0	0	0	0	0	0	0	0	0	0	0	0	0	0	0	0	0	0	1	41.76071667	19.77716667
295	0	0	0	0	0	0	0	0	0	0	0	0	0	0	0	0	0	0	1	0	0	0	0	0	0	0	0	0	0	0	0	0	0	1	41.78833333	20.10681667
296	0	0	0	0	0	0	0	0	0	0	0	0	0	0	0	0	0	0	0	0	0	0	0	0	0	0	0	0	0	0	0	0	0	1	41.83611667	20.17396667
297	0	0	0	0	1	0	0	0	0	0	0	0	0	0	0	0	0	0	0	0	0	0	0	0	0	0	0	0	0	0	0	0	0	1	41.83174	20.17713
302	0	0	0	0	0	0	0	0	0	0	0	0	0	0	0	0	0	0	1	0	0	0	0	0	0	0	0	0	0	0	0	0	0	1	41.79415	20.11205
303	0	0	0	0	1	0	0	0	0	0	0	0	0	0	0	0	0	0	0	0	0	0	0	0	0	0	0	0	0	0	0	0	0	1	41.2278	19.98141667
305	0	0	0	0	1	0	0	0	0	0	0	0	0	1	0	0	0	0	0	0	0	0	0	0	0	0	0	0	0	0	0	0	0	1	40.92798333	20.03055
306	0	0	0	0	1	0	0	0	0	0	0	0	0	0	0	0	0	0	0	0	0	0	0	0	0	0	0	0	0	0	0	0	0	1	41.11203333	20.08118333
308	0	0	0	1	1	0	0	0	0	0	0	0	0	0	0	0	1	0	0	0	0	0	0	0	0	0	0	0	0	0	0	0	0	1	41.1336	20.12776667
309	0	0	0	0	1	0	0	0	0	0	0	0	0	0	0	0	1	0	0	0	0	0	0	0	0	0	0	0	0	0	0	0	0	1	41.16756667	20.24983333
310	0	0	0	0	0	1	0	0	0	0	0	0	0	0	0	0	0	0	0	0	0	0	0	0	0	0	0	0	0	0	0	0	0	1	41.09621667	20.44281667
312	0	0	0	1	1	0	0	0	0	0	0	0	0	0	0	0	1	0	0	0	0	0	0	0	0	0	0	0	0	0	0	0	0	1	41.0937	20.44025
313	0	0	0	0	1	0	0	0	0	0	0	0	0	0	0	0	0	0	0	0	0	0	0	0	0	0	0	0	0	0	0	0	0	1	41.06506667	20.59711667
318	0	0	0	0	1	0	0	0	0	0	0	0	0	0	0	0	0	0	0	0	0	0	0	0	0	0	0	0	0	0	0	0	0	1	40.93781667	20.45406667
319	0	0	0	0	1	0	0	0	0	0	0	0	0	0	0	0	1	0	0	0	0	0	0	0	0	0	0	0	0	0	0	0	0	1	40.92096667	20.4491
320	0	0	0	0	1	0	0	0	0	0	0	0	0	0	0	0	0	0	0	0	0	0	0	0	0	0	0	0	0	0	0	0	0	1	40.92333333	20.44386667
327	0	0	0	0	0	0	0	0	0	0	0	0	0	0	1	0	0	0	0	0	0	0	0	0	0	0	0	0	0	0	0	0	0	1	40.7891	20.90725
329	0	0	0	0	1	0	0	0	0	0	0	0	0	0	0	0	0	0	0	0	0	0	0	0	0	0	0	0	0	0	0	0	0	1	40.52556667	20.68865
330	0	0	0	0	1	0	0	0	0	0	0	0	0	0	0	0	0	0	0	0	0	0	0	0	0	0	0	0	0	0	0	0	0	1	40.52835	20.63436667
331	0	0	0	0	0	0	0	0	0	0	0	0	0	0	0	0	0	0	0	0	0	0	0	0	0	0	0	0	0	0	0	0	0	1	40.5333	20.62276667
332	0	0	0	1	0	0	0	0	0	0	0	0	0	0	0	0	0	0	0	0	0	0	0	0	1	0	0	0	0	0	0	0	0	1	40.29153333	20.6281
334	0	0	0	0	0	0	0	0	0	0	0	0	0	0	0	0	0	0	0	0	0	0	0	0	0	0	0	0	0	0	0	0	0	1	40.23783333	20.64371667
335	0	0	0	0	1	0	0	0	0	0	0	0	0	0	0	0	0	0	0	0	0	0	0	0	0	0	0	0	0	0	0	0	0	1	40.15266667	20.61966667
336	0	0	0	0	0	0	0	0	0	0	0	0	0	0	0	0	0	0	0	0	0	0	0	0	0	0	0	0	0	0	0	0	0	1	40.15093333	20.59491667
339	0	0	0	1	1	0	0	0	0	0	0	0	0	0	0	0	0	0	0	0	0	0	0	0	1	0	0	0	0	0	0	0	0	1	40.69201667	20.50015
342	0	0	0	0	1	0	0	0	0	0	0	0	0	0	0	0	0	0	0	0	0	0	0	0	0	0	0	0	0	0	0	0	0	1	40.71361667	20.36178333
343	0	0	0	0	1	0	0	0	0	0	0	0	0	0	0	0	0	0	0	0	0	0	0	0	0	0	0	0	0	0	0	0	0	1	40.88303333	20.24433333
345	0	0	0	0	0	0	0	0	0	0	0	0	0	0	0	0	0	0	0	0	0	0	0	0	0	0	0	0	0	0	0	0	0	1	42.38846667	19.7363
346	0	0	0	0	0	0	0	0	0	0	0	0	0	0	0	0	0	0	0	0	0	0	0	0	0	0	0	0	0	0	0	0	0	1	42.40228333	19.76318333
347	0	0	0	0	0	0	0	0	0	0	0	0	0	0	0	0	0	0	0	0	0	0	0	0	0	0	0	0	0	0	0	0	0	1	42.42773333	19.76173333
348	0	0	0	0	0	0	0	0	0	0	0	0	0	0	0	0	0	0	0	0	0	0	0	0	0	0	0	0	0	0	0	0	0	1	42.4438	19.77095

349	2003/085		0	0	0	2	42.41478333	19.75681667
350	2003/086		1	0	0	3	42.40188333	19.7493
351	2003/087		0	0	0	1	42.38951667	19.72703333
353	2003/089		1	0	0	1	42.43933333	19.53088333
354	2003/090		1	0	0	1	42.48103333	19.58266667
355	2003/091		1	0	0	1	42.5077	19.61598333
356	2003/092		1	0	0	1	42.52241667	19.6319
358	2003/094		0	0	0	1	42.5364	19.6884
359	2003/095		1	0	0	1	42.53228333	19.65086667
360		0	0	0	0	1	42.6306	19.0085
361		0	1	0	0	1	42.4203	18.8239
362		0	1	0	0	1	42.5208	18.6908
363	2004/003		0	0	0	1	41.8265	21.6865
364	2004/004		0	0	0	1	41.704	21.7885
365	2004/005		0	0	0	1	41.70633333	21.78133333
367	2004/007		0	0	0	1	41.681	21.43033333
368	2004/009		1	0	0	1	41.43683333	21.86716667
369	2004/010		1	0	0	1	41.43666667	21.81666667
370	2004/011		0	0	0	1	41.3655	21.7165
373	2004/014		0	0	0	1	40.935	20.93666667
374	2004/015		0	0	0	1	40.96883333	20.91016667
376	2004/017		0	0	0	2	40.95583333	20.82033333
377	2004/018		0	0	0	1	40.954	20.8125
379	2004/020		0	0	0	3	40.97116667	20.82066667
380	2004/021		0	0	0	1	40.96466667	20.81466667
388	2004/028		0	0	0	1	40.9656	20.78585
391	2004/031		1	0	0	1	41.074	20.803
392	2004/032		0	0	0	1	41.103	20.81433333
393	2004/033		0	0	0	1	41.11133333	20.7905
394	2004/034		0	0	0	1	41.19783333	20.91733333
396	2004/036		0	0	0	1	41.13883333	20.7625
397	2004/037		0	0	0	1	41.18283333	20.63233333
399	2004/038		0	0	0	1	41.14933333	20.64933333
401	2004/040		0	0	0	1	41.3355	20.6345
406	2004/045		1	0	0	3	41.621	20.60916667
418	2004/057		0	0	0	1	42.02083333	20.902
419	2004/058		0	0	0	2	42.01566667	20.8855
420	2004/059		0	0	0	3	42.01133333	20.90233333
421	2004/060		1	0	0	1	41.961	21.29433333
422	2004/061		0	0	0	1	43.85766667	21.6145
424		0	0	0	0	1	40.69865	20.096633
425	2004/075		1	0	0	1	40.55355	20.27832
426	2004/076		1	0	0	1	40.55387	20.27148
427	2004/077		1	0	0	2	40.56024	20.26453
428	2004/078		0	0	0	1	40.56464	20.26193
429	2004/079		1	1	0	1	40.56856	20.25854
430	2004/080		0	1	0	1	40.57754	20.25825
431	2004/081		0	0	0	1	40.58657	20.24382
432	2004/082		0	1	0	1	40.58961	20.24137
433	2004/083		0	1	0	1	40.59128	20.23847
434	2004/084		1	1	0	1	40.59518	20.22139
435	2004/085		0	0	0	2	40.59848	20.21548
436	2004/086		0	0	0	1	40.6248	20.1742
437	2004/087		0	1	0	1	40.63063	20.16619
439	2004/089		1	1	0	2	40.64401	20.15902
440	2004/090		1	0	0	1	40.65156	20.15729
441	2004/091		1	0	0	1	40.65334	20.16117
442	2004/092		0	0	0	1	40.6745	20.15773
443	2004/093		1	1	0	1	40.68722	20.15067
444	2004/094		1	1	0	1	40.68886	20.14611
445	2004/095		0	0	0	2	40.69866	20.14496
446	2004/096		0	1	0	3	40.68797	20.13987
447	2004/097		0	1	0	3	40.68832	20.1341
448	2004/098		0	0	0	1	40.68779	20.12874
450	2004/100		1	1	0	1	40.67884	20.11085
451	2004/101		1	0	0	1	40.21584	19.60634
452	2004/102		1	0	0	2	40.21383	19.61098
453	2004/103		0	0	0	2	40.20966	19.61786
454	2004/104		1	0	0	1	40.19852	19.59163
456	2004/106		1	0	0	1	42.66346	19.37159
459	2004/109		1	0	0	1	41.5065	19.92183333
466	2004/115		0	0	0	1	41.47083333	20.07783333
472	2004/121		1	0	0	1	41.35147222	20.11627778
475	2004/124		0	0	0	1	40.516	20.24263889
476	2004/125		1	0	0	1	40.5235	20.25311111
480	2004/128		1	0	0	1	40.53561111	19.79638889
481	2004/130		1	0	0	1	40.24916667	19.99397222
482	2004/131		0	0	0	2	40.24077778	19.96152778
483	2004/132		1	0	0	1	40.21022222	19.96141667
484	2004/133		1	0	0	2	40.2135	19.93541667
485	2004/134		1	0	0	1	40.21297222	19.93052778
486	2004/135		1	0	0	1	40.26347222	20.00708333
487	2004/136		1	0	0	1	40.29380556	20.00875
489	2004/138		1	0	0	1	40.08827778	20.27166667
490	2004/139		1	0	0	1	40.09147222	20.29219444
491	2004/140		1	0	0	1	40.11641667	20.32941667
492	2004/141		1	0	0	1	40.17011111	20.31511111
493	2004/142		1	0	0	1	40.18558333	20.33236111
495	2004/144		1	0	0	1	40.07444444	20.14063889
496	2004/145		1	0	0	3	39.92461111	20.1925
499	2004/147		1	0	0	1	40.05277778	19.81280556
500	2004/148		1	0	0	1	40.15577778	19.63936111
501	2004/149		0	0	0	1	42.74833333	19.40666667
502	2004/150		0	0	0	1	43.29027778	19.714
506	2005/B02		1	0	0	1	40.26658	20.00596
507	2005/B03		1	0	0	1	40.26201	19.98091
508	2005/B04		1	0	0	1	40.26385	19.9704
509	2005/B05		1	0	0	1	40.26494	19.95902
510	2005/B06		1	0	0	1	40.27073	19.92616
511	2005/B07		1	0	0	1	40.27602	19.9013
512	2005/B08		0	0	0	1	40.27822	19.87347
513	2005/B09		1	0	0	1	40.2843	19.85666
514	2005/B10		1	0	0	1	40.28706	19.8528
515	2005/B10B		0	0	0	1	40.28698	19.8614
517		0	1	0	0	1	42.44313	19.76374
518		0	0	0	0	1	42.40623	19.80768
520	2005/O02		0	0	0	1	40.51995	20.41771
522	2005/O04		0	0	0	1	40.52602	20.41528
524	2005/O13		0	0	0	1	40.57649	20.44733
525	2005/O15		1	0	0	1	40.56065	20.39377
528	2005/043		0	0	0	1	42.55023333	19.82476667
529	2005/044		0	0	0	1	42.57818333	19.81493333
532	2005/047		1	0	0	1	42.57208333	19.73991667
534	2005/049		1	0	0	1	42.51563333	19.73713333
540	2005/054		1	0	0	1	42.68468333	20.05356667
541	2005/055		1	0	0	1	42.7723	20.27901667
544	2005/058		0	0	0	1	42.38841667	20.0729
545	2005/059		1	0	0	1	42.41638333	20.01271667
546	2005/060		0	0	0	1	42.4364	19.98465
547	2005/061		0	0	0	1	42.41033333	19.82276667
548	2005/062		0	0	0	1	42.41135	19.81475
549	2005/063		1	0	0	1	42.40678333	19.8122
552	2005/065		1	0	0	1	42.29878333	20.02885
553	2005/066		1	0	0	1	42.28676667	20.02145
554	2005/067		1	0	0	1	42.26306667	19.99
555	2005/068		0	0	0	1	42.2678	19.94943333
557	2005/070		1	0	0	1	42.22025	20.09765
561	2005/074		0	0	0	1	41.94721667	20.27955
562	2005/075		0	0	0	1	41.92416667	20.2813
563	2005/076		1	0	0	1	41.9163	20.2526
564	2005/077		1	0	0	1	41.91881667	20.26928333
568	2005/081		0	0	0	1	41.94046667	20.37601667
569	2005/082		0	0	0	1	41.93515	20.3632
570	2005/083		0	0	0	1	41.92371667	20.35395
571	2005/084		1	0	0	1	41.94231667	20.35933333
572	2005/085		0	0	0	1	41.90813333	20.35825
573	2005/086		1	0	0	1	41.89078333	20.337
574	2005/087		1	0	0	1	41.88803333	20.31651667
576	2005/089		1	0	0	1	41.73908333	20.29681667
578	2005/090A		1	0	0	1	41.75248333	20.24553333
579	2005/090B		0	0	0	1	41.7506	20.26256667
580	2005/091		0	0	0	1	41.78555	20.28701667
581	2005/092		1	0	0	1	41.7994	20.28061667
582	2005/093		1	0	0	3	41.81486667	20.2775
586	2005/097		1	0	0	1	41.61865	20.28953333
587	2005/098		0	0	0	1	42.73731667	20.3068
588	2005/099		0	0	0	1	42.85305	20.20396667
589	2005/100		1	0	0	1	42.90683333	20.33436667
590	2005/101		0	0	0	1	43.13183333	20.41605
591		0	1	0	0	1	42.305	18.8889
593		0	1	0	0	1	42.474306	18.53625
596	2006/005		1	0	0	1	41.03578333	20.06861667
598	2006/007		1	0	0	1	40.74306667	20.12713333
599	2006/008		0	0	0	1	41.15756667	20.12466667
600	2006/009		1	0	0	1	41.20353333	20.11458333
601	2006/010		1	0	0	1	41.21271667	20.1364
602	2006/011		0	0	0	1	41.2226	20.12675
604	2006/013		0	0	0	1	41.23886667	20.0976
607	2006/016		1	0	0	1	41.29093333	20.0832
608	2006/017		0	0	0	3	41.2951	20.10085
609	2006/018		1	0	0	1	41.29063333	20.11888333
611	2006/020		1	0	0	1	41.27475	20.26793333
612	2006/021		0	0	0	1	41.266	20.25955
615	2006/024		1	0	0	1	41.26601667	20.3182
622	2006/031A		1	0	0	1	41.75205	20.25241667
623	2006/031B		1	0	0	1	41.75283333	20.25048333
625	2006/032		1	0	0	1	41.75713333	20.23888333
628	2006/034		0	0	0	1	41.66241667	20.31728333
632	2006/038		0	0	0	1	41.64511667	20.18231667
633	2006/039		1	0	0	1	41.64605	20.1806
635	2006/041		1	0	0	1	41.65025	20.18606667
637	2006/043		1	0	0	1	41.63516667	20.12433333
642	2006/048		1	0	0	1	42.1217	19.83136667
643	2006/049		1	0	0	1	42.11655	19.83738333
644	2006/050		1	0	0	1	42.08093333	19.73621667
646	2006/052		1	0	0	1	42.18715	19.86955

647	2006/053		1	0	[illegible]	0	0	0	0	1	42.20535	19.89178333
649	2006/055		1	0	[illegible]	0	0	0	0	1	42.22583333	19.89236667
650	2006/056		1	1	[illegible]	0	0	0	0	1	42.22688333	19.905
652	2006/058		0	0	[illegible]	1	0	0	0	1	42.06913333	19.41313333
653	2006/059		1	0	[illegible]	1	0	0	0	1	42.07236667	19.39463333
654	2006/060		1	0	[illegible]	0	0	0	0	1	42.14695	19.54543333
655	2006/061		1	0	[illegible]	0	0	0	0	1	42.1523	19.5402
656	2006/062		1	0	[illegible]	0	0	0	0	1	42.18966667	19.5621
658	2006/064		1	0	[illegible]	0	0	0	0	1	42.2045	19.568
659	2006/065		1	0	[illegible]	0	0	0	0	1	42.21146667	19.57916667
662	2006/068		1	0	[illegible]	0	0	0	0	1	42.11793333	19.65896667
663	2006/069		1	0	[illegible]	0	0	0	0	1	42.12571667	19.66768333
670	2006/075		0	0	[illegible]	0	0	0	0	1	43.46708333	19.6512
672		0	1	0	[illegible]	0	0	0	0	2	40.143333	19.662222
673		0	1	0	[illegible]	0	0	0	0	1	40.143694	19.677
676		0	0	0	[illegible]	0	0	0	0	3	40.211944	19.579167
679		0	0	0	[illegible]	0	0	0	0	1	39.557917	20.468833
681		0	0	0	[illegible]	0	0	0	0	2	39.695278	20.703611
687		0	0	0	[illegible]	0	0	0	0	1	40.107694	21.021167
692		0	0	0	[illegible]	0	0	0	0	1	40.20301	20.31674
693		0	0	0	[illegible]	0	0	0	0	1	40.1933	20.31429
694		0	0	0	[illegible]	0	0	0	0	1	40.50547	20.55125
695		0	0	0	[illegible]	0	0	0	0	1	40.50864	20.56533
696		0	1	0	[illegible]	0	0	0	0	1	40.25182	19.96526
698		0	1	0	[illegible]	0	0	0	0	1	40.27581	20.09397
699		0	1	0	[illegible]	0	0	0	0	2	40.28722	20.08328
700		0	1	0	[illegible]	0	0	0	0	1	40.34056	19.78027
701		0	0	0	[illegible]	0	0	0	0	2	40.33741	19.77468
705	2006/134		1	0	[illegible]	0	0	0	0	1	40.50051667	20.28601667
706	2006/135		0	0	[illegible]	0	0	0	0	1	40.51638333	20.40703333
707	2006/135B		0	0	[illegible]	0	0	0	0	1	40.51776667	20.4124
708	2006/136		0	0	[illegible]	0	0	0	0	1	40.5219	20.41388333
709	2006/137		0	0	[illegible]	0	0	0	0	1	40.52921667	20.4192
710	2006/138		1	0	[illegible]	0	0	0	0	1	40.53245	20.42563333
711	2006/139		0	0	[illegible]	0	0	0	0	1	40.52453333	20.41633333
713	2006/141		1	0	[illegible]	0	0	0	0	1	40.54348333	20.27181667
714	2006/142		0	0	[illegible]	0	0	0	0	1	40.5303	20.25753333
715	2006/143		1	0	[illegible]	0	0	0	0	1	40.5223	20.2574
717	2006/145		0	0	[illegible]	0	0	0	0	2	40.54218333	20.346
718	2006/146		1	0	[illegible]	0	0	0	0	1	40.559	20.39083333
719	2006/147		1	0	[illegible]	0	0	0	0	1	40.57145	20.28798333
720	2006/148		1	0	[illegible]	0	0	1	0	1	40.63281667	20.21615
721	2006/149		0	0	[illegible]	0	0	1	0	1	40.64113333	20.15746667
722	2006/150		0	0	[illegible]	0	0	0	0	1	40.6186	20.19908333
723	2006/151		1	0	[illegible]	0	0	0	0	1	40.68131667	20.18348333
724	2006/152		1	0	[illegible]	0	0	1	0	1	40.7127	20.18391667
725	2006/153		1	0	[illegible]	0	0	0	0	1	40.72125	20.17201667
727	2006/155		1	0	[illegible]	0	0	1	0	1	40.71638333	20.15671667
730	2006/158		1	0	[illegible]	0	0	0	0	1	40.92603333	20.22316667
734	2006/162		0	0	[illegible]	1	0	0	0	1	42.32533333	19.36275
739		0	0	0	[illegible]	0	0	0	0	1	41.760278	20.669722
744		0	1	0	[illegible]	0	0	0	0	3	40.976639	20.794083
748		0	0	0	[illegible]	0	0	0	0	1	41.843083	22.505917
750		0	0	0	[illegible]	0	0	0	0	1	40.82548	20.85281
751		0	0	0	[illegible]	0	0	0	0	1	40.8179	20.84941
752		0	0	0	[illegible]	0	0	0	0	1	40.81426	20.84022
753		0	0	0	[illegible]	0	0	0	0	1	40.80241	20.87408
754		0	0	0	[illegible]	0	0	0	0	1	40.81832	20.87283
755		0	0	0	[illegible]	0	0	0	0	1	40.82203	20.87886
757		0	0	0	[illegible]	0	0	0	0	1	40.74191	20.86351
758		0	1	0	[illegible]	0	0	0	0	1	40.7565	20.84945
759		0	1	0	[illegible]	0	0	0	0	1	40.53767	20.57924
760	2007/091		0	0	[illegible]	0	0	0	0	1	42.3162	18.93421667
761	2007/092		0	0	[illegible]	0	0	0	0	1	42.27805556	18.86
763	2007/102		0	0	[illegible]	0	0	0	0	1	42.20878333	20.7512
764	2007/103		0	0	[illegible]	0	0	0	0	1	42.06631667	20.48795
765	2007/104		0	0	[illegible]	0	0	0	0	1	42.05883333	20.50181667
767	2007/106		0	0	[illegible]	0	0	0	0	1	41.98666667	20.52858333
774	2007/112		0	0	[illegible]	0	0	0	0	2	41.99465	20.48498333
775	2007/113		0	0	[illegible]	0	0	0	0	1	41.99895	20.47168333
776	2007/114		0	0	[illegible]	0	0	0	0	1	41.99931667	20.46133333
777	2007/115		1	0	[illegible]	0	0	0	0	3	41.99956667	20.45773333
779	2007/117		0	0	[illegible]	0	0	0	0	2	42.00946667	20.46965
780	2007/117B		0	0	[illegible]	0	0	0	0	2	42.01208333	20.46965
783	2007/120		1	0	[illegible]	0	0	0	0	1	41.98971667	20.4202
784	2007/121		0	0	[illegible]	0	0	0	0	1	41.92596667	20.38706667
785	2007/122		0	0	[illegible]	0	0	0	0	1	41.9097	20.409
786	2007/123		1	0	[illegible]	0	0	0	0	1	41.89498333	20.41733333
789	2007/126		0	0	[illegible]	0	0	0	0	1	41.81885	20.50266667
790	2007/127		0	0	[illegible]	0	0	0	0	1	41.8179	20.52701667
792	2007/128		0	0	[illegible]	0	0	0	0	1	41.81868333	20.53733333
793	2007/129		0	0	[illegible]	0	0	0	0	1	41.8052	20.56088333
795	2007/131		0	0	[illegible]	0	0	0	0	1	41.80008333	20.54993333
796	2007/132		0	0	[illegible]	0	0	0	0	1	41.79526667	20.54228333
797	2007/133		0	0	[illegible]	0	0	0	0	2	41.79335	20.5439
798	2007/134		0	0	[illegible]	0	0	0	0	1	41.79705	20.54536667
803	2007/139		0	0	[illegible]	0	0	0	0	1	41.81535	20.54485
805	2007/141		0	0	[illegible]	0	0	0	0	1	41.81915	20.52173333
810	2007/146		0	0	[illegible]	0	0	0	0	1	41.76893333	20.27673333
813	2007/149		1	0	[illegible]	0	0	0	0	1	41.81365	20.23618333
817	2007/153		1	0	[illegible]	0	0	0	0	1	41.83818333	20.16646667
819	2007/155		0	0	[illegible]	0	0	0	0	1	41.50988333	20.31515
823	2007/159		1	0	[illegible]	0	0	0	0	1	41.43065	20.09196667
828	2007/164		1	0	[illegible]	0	0	0	0	1	41.32106667	20.14736667
831	2007/167		1	0	[illegible]	0	0	0	0	1	40.5378	20.56996667
836	2007/172		0	0	[illegible]	0	0	0	0	1	40.79151667	20.9324
838	2007/174		1	0	[illegible]	0	0	0	0	1	40.26878333	20.6065
841	2007/177		1	0	[illegible]	0	0	0	0	1	40.24463333	20.4377
843	2007/179		1	0	[illegible]	0	0	0	0	1	40.29883333	20.0886
844		0	0	0	[illegible]	0	0	0	0	1	40.86643	20.42862
845		0	0	0	[illegible]	0	0	0	0	1	40.84757	20.11453
846	2007/180		1	0	[illegible]	0	0	0	0	1	40.0727	20.13916667
848	2007/182		1	0	[illegible]	0	0	0	0	1	39.74535	20.01955
850	2007/184		1	0	[illegible]	0	0	0	0	1	40.0808	19.76746667
854	2007/188		1	0	[illegible]	0	0	0	0	1	41.37341667	19.91983333
855	2007/189		1	0	[illegible]	0	0	0	0	3	41.37311667	19.92003333
856	2007/190		0	0	[illegible]	0	0	0	0	3	41.3689	19.9221
864		0	1	0	[illegible]	0	0	0	0	1	41.24978	20.5044
865	2008/110		1	0	[illegible]	0	0	0	0	1	42.51425	18.48736667
866	2008/111		1	0	[illegible]	0	0	0	0	1	42.52776667	18.4875
867	2008/112		0	0	[illegible]	0	0	0	0	3	42.54983333	18.65491667
868	2008/113		1	0	[illegible]	0	0	0	0	1	42.52365	18.68546667
871	2008/116		1	0	[illegible]	0	0	0	0	1	42.48616667	18.76703333
872	2008/117		1	0	[illegible]	0	0	0	0	1	42.40241667	18.77496667
873	2008/118		1	0	[illegible]	0	0	0	0	1	42.40036667	18.79401667
874	2008/119		0	0	[illegible]	0	0	0	0	3	42.3999	18.83136667
875	2008/120		1	0	[illegible]	0	0	0	0	2	42.3989	18.83808333
876	2008/120A		0	0	[illegible]	0	0	0	0	1	42.39963333	18.83871667
877	2008/121		1	0	[illegible]	0	0	0	0	2	42.39503333	18.80905
880	2008/124		0	0	[illegible]	0	0	0	0	1	42.39938333	18.78271667
882	2008/126		0	0	[illegible]	0	0	0	0	3	42.60271667	18.67483333
883	2008/127		0	0	[illegible]	0	0	0	0	1	42.65231667	18.67201667
884	2008/128		0	0	[illegible]	0	0	0	0	3	42.67763333	18.64191667
885	2008/129		1	0	[illegible]	0	0	0	0	3	42.71938333	18.60241667
886	2008/130		1	0	[illegible]	0	0	0	0	1	42.76236667	18.82618333
888	2008/132		0	0	[illegible]	0	0	0	0	2	42.85776667	18.94326667
889	2008/133		0	0	[illegible]	0	0	0	0	3	42.9017	19.02098333
890	2008/134		1	0	[illegible]	0	0	0	0	1	42.89493333	19.07943333
891	2008/135		1	0	[illegible]	0	0	0	0	1	42.94763333	19.09105
892	2008/136		0	0	[illegible]	0	0	0	0	1	42.94975	19.0993
893	2008/137		0	0	[illegible]	0	0	0	0	1	42.9622	19.09308333
894	2008/138		0	0	[illegible]	0	0	0	0	1	42.95765	19.0959
895	2008/139		0	0	[illegible]	0	0	0	0	2	42.98756667	19.0668
896	2008/140		0	0	[illegible]	0	0	0	0	1	42.9883	19.06783333
897	2008/141		1	0	[illegible]	0	0	0	0	1	42.98156667	19.06833333
898	2008/142		1	0	[illegible]	0	0	0	0	1	42.98718333	19.07993333
899	2008/143		1	0	[illegible]	0	0	0	0	1	42.9507	19.1735
903	2008/147		0	0	[illegible]	0	0	0	0	1	42.90093333	19.27321667
904	2008/148		0	0	[illegible]	0	0	0	0	1	42.88223333	19.29768333
905	2008/149		1	0	[illegible]	0	0	0	0	1	42.84868333	19.33448333
906	2008/149A		0	0	[illegible]	0	0	0	0	1	42.81903333	19.38101667
908	2008/151		0	0	[illegible]	0	0	0	0	1	42.88206667	19.39978333
909	2008/152		1	0	[illegible]	0	0	0	0	1	42.90301667	19.38221667
910	2008/153		1	0	[illegible]	0	0	0	0	1	42.89715	19.38566667
911	2008/154		0	0	[illegible]	0	0	0	0	3	42.89151667	19.38918333
912	2008/155		0	0	[illegible]	0	0	0	0	1	42.8722	19.47948333
915	2008/158		0	0	[illegible]	0	0	0	0	2	42.89495	19.64246667
916	2008/159		0	0	[illegible]	0	0	0	0	2	42.88933333	19.65971667
917	2008/160		0	0	[illegible]	0	0	0	0	1	42.87968333	19.74111667
919	2008/162		1	0	[illegible]	0	0	0	0	1	42.51173333	19.8348
921	2008/163R		0	0	[illegible]	0	0	0	0	1	42.4907	19.81885
922	2008/164		0	0	[illegible]	0	0	0	0	1	42.49095	19.81231667
923	2008/165		0	0	[illegible]	0	0	0	0	2	42.50163333	19.82456667
924	2008/166		1	0	[illegible]	0	0	0	0	1	42.6337	19.85008333
929	2008/171		0	0	[illegible]	0	0	0	0	1	42.67545	19.52291667
930	2008/172		1	0	[illegible]	0	0	0	0	1	42.61888333	19.55368333
932	2008/174		0	0	[illegible]	0	0	0	0	1	42.56941667	19.6025
933	2008/175		0	0	[illegible]	0	0	0	0	3	42.57495	19.59783333
934	2008/176		0	0	[illegible]	0	0	0	0	1	42.57885	19.59216667
935	2008/177		0	0	[illegible]	0	0	0	0	1	42.59308333	19.5801
936	2008/178		1	0	[illegible]	0	0	0	0	1	42.59936667	19.51166667
937	2008/179		1	0	[illegible]	0	0	0	0	1	42.54876667	19.45435
938	2008/180		1	0	[illegible]	0	0	0	0	1	42.51081667	19.38476667
939	2008/181		1	0	[illegible]	0	0	0	0	1	42.45411667	19.35115
940	2008/182		1	0	[illegible]	1	0	0	0	1	42.1924	19.107
941	2008/183		0	0	[illegible]	0	0	0	0	1	42.09386667	19.13423333
942	2008/184		0	0	[illegible]	0	0	0	0	1	42.10043333	19.1419
943	2008/185		0	0	[illegible]	0	0	0	0	1	42.09726667	19.14583333
944	2008/185B		1	0	[illegible]	0	0	0	0	1	42.0978	19.14466667
945	2008/185C		0	0	[illegible]	0	0	0	0	1	42.09965	19.14263333
946	2008/186		1	0	[illegible]	1	0	0	0	1	42.21876667	19.0246
948	2008/188		0	0	[illegible]	0	0	0	0	1	43.07073333	19.86145
951		0	1	0	[illegible]	0	0	0	0	1	41.69387	19.821554
952		0	1	0	[illegible]	0	0	0	0	1	41.63089	19.73321
954		0	1	0	[illegible]	0	0	0	0	1	40.16149	19.65018
955		0	1	0	[illegible]	0	0	0	0	1	39.77261	20.07333

956		0	1	0	[illegible]	0	0	1	39.82265	20.06533
957		0	0	0	[illegible]	0	0	1	39.75738	20.08182
959		0	0	0	[illegible]	0	0	1	42.34298	20.03205
960		0	1	0	[illegible]	0	0	1	42.36478	20.01736
961		0	1	0	[illegible]	0	0	2	42.34867	19.99474
962		0	1	0	[illegible]	0	0	1	42.35199	19.99302
963		0	0	0	[illegible]	0	0	1	42.46327	19.88637
964		0	0	0	[illegible]	0	0	2	42.44961	20.13148
965		0	1	0	[illegible]	0	0	2	42.46322	20.12577
966		0	0	0	[illegible]	0	0	2	42.4678	20.12229
969	2009/123		0	0	[illegible]	0	0	1	43.8713	20.11421667
970	2009/124		0	0	[illegible]	0	0	1	43.90175	20.19268333
974	2009/128		0	0	[illegible]	0	0	1	43.72348333	21.9478
975	2009/129		0	0	[illegible]	0	0	1	43.7957	21.90348333
977		0	1	0	[illegible]	0	0	1	41.37773	20.03291
978		0	0	0	[illegible]	0	0	2	41.68776	20.15676
979		0	0	0	[illegible]	0	0	3	41.68861	20.15571
980		0	0	0	[illegible]	0	0	1	41.69076	20.14681
981		0	0	0	[illegible]	0	0	1	41.69025	20.16586
982		0	1	0	[illegible]	0	0	1	40.18113	20.66172
983		0	1	0	[illegible]	0	0	1	39.86484	20.02188
984		0	1	0	[illegible]	0	0	1	39.68221	20.12899
985		0	1	0	[illegible]	0	0	1	40.129	19.67311
986		0	1	0	[illegible]	0	0	1	42.29318	19.53328
996	2010/009		0	0	[illegible]	0	0	1	41.62558333	20.24983333
997	2010/010		1	0	[illegible]	0	0	1	41.64651667	20.19013333
998	2010/011		0	0	[illegible]	0	0	1	41.66373333	20.19533333
999	2010/012		0	0	[illegible]	0	0	1	41.66508333	20.20828333
1001	2010/013B		1	0	[illegible]	0	0	1	41.67465	20.21115
1003	2010/BP1		0	0	[illegible]	0	0	2	41.67671	20.2135
1004	2010/015		1	0	[illegible]	0	0	1	41.69623333	20.13331667
1005	2010/016		1	0	[illegible]	0	0	1	41.69708333	20.13618333
1008	2010/BP2		0	0	[illegible]	0	0	1	41.69194	20.21288
1009	2010/BP4		0	0	[illegible]	0	0	1	41.68977	20.25569
1010	2010/BP5		0	0	[illegible]	0	0	1	41.69413	20.2618
1012	2010/020		1	0	[illegible]	0	0	1	41.81346667	20.13973333
1024	2010/032		1	0	[illegible]	0	0	1	41.84231667	20.09451667
1025	2010/033		1	0	[illegible]	0	0	1	41.85056667	20.09736667
1026	2010/034		1	0	[illegible]	0	0	1	41.85456667	20.1217
1028	2010/036		1	0	[illegible]	0	0	1	41.86923333	20.11863333
1029	2010/037		0	0	[illegible]	0	0	1	41.87066667	20.1085
1030	2010/038		0	0	[illegible]	0	0	1	42.13848333	20.49005
1031	2010/039		0	0	[illegible]	0	0	3	42.19235	20.53763333
1032	2010/040		0	0	[illegible]	0	0	3	42.20076667	20.5333
1033	2010/041		1	0	[illegible]	0	0	3	42.20285	20.53378333
1034	2010/042		1	0	[illegible]	0	0	2	42.20695	20.52848333
1035	2010/043		1	0	[illegible]	0	0	1	42.11456667	19.57471667
1036	2010/044		1	0	[illegible]	0	0	1	42.12485	19.6037
1037	2010/045		1	0	[illegible]	0	0	1	42.14171667	19.64743333
1038	2010/046		1	0	[illegible]	0	0	1	42.16498333	19.69515
1039	2010/047		1	0	[illegible]	0	0	1	42.17863333	19.72008333
1040	2010/048		1	0	[illegible]	0	0	1	42.207	19.70906667
1041	2010/049		1	0	[illegible]	0	0	1	42.21423333	19.70581667
1042	2010/050		0	0	[illegible]	0	0	1	42.24528333	19.71691667
1043	2010/051		0	0	[illegible]	0	0	1	42.24036667	19.72803333
1045	2010/053		0	0	[illegible]	0	0	1	42.25136667	19.74788333
1046	2010/054		1	0	[illegible]	0	0	1	42.28976667	19.79683333
1047	2010/055		0	1	[illegible]	0	0	1	42.3043	19.7969
1048	2010/056		0	0	[illegible]	0	0	3	42.40128333	19.7658
1049	2010/BP8		0	0	[illegible]	0	0	3	41.99294	20.4267
1050	2010/BP9		0	0	[illegible]	0	0	2	42.00798333	20.44608333
1051	2010/057		0	0	[illegible]	0	0	3	42.40161667	19.7577
1055	2010/061		1	0	[illegible]	0	0	1	43.21303333	19.76305
1056	2010/062		1	0	[illegible]	0	0	1	43.61168333	19.72516667
1057	2010/BP12		0	0	[illegible]	0	0	1	41.97755	20.11537
1058		0	0	0	[illegible]	0	0	1	42.445683	19.756067
1059		0	0	0	[illegible]	0	0	1	42.419	19.64347
1060		0	0	0	[illegible]	0	0	1	42.42166	19.67168
1061		0	0	0	[illegible]	0	0	1	42.43077	19.69267
1062		0	0	0	[illegible]	0	0	1	42.45781	19.71515
1063		0	0	0	[illegible]	0	0	1	42.45572	19.77465
1064		0	0	0	[illegible]	0	0	1	42.46108	19.71596
1065		0	0	0	[illegible]	0	0	2	39.70188333	20.89465
1066		0	0	0	[illegible]	0	0	2	39.72825	21.0839
1068		0	0	0	[illegible]	0	0	1	39.177167	20.734383
1069		0	1	0	[illegible]	0	0	1	38.722717	20.690067
1071		0	0	0	[illegible]	0	0	1	41.31676667	20.3774
1072		0	1	0	[illegible]	0	0	1	40.8665	20.377333
1073		0	1	0	[illegible]	0	0	1	40.08	19.954
1076	2012/103		0	0	[illegible]	0	0	1	43.18343333	19.76168333
1077	2012/104		1	0	[illegible]	0	0	3	43.02753333	19.40143333
1082	2012/109		1	0	[illegible]	0	0	1	42.40636667	19.34983333
1092	2012/119		0	0	[illegible]	0	0	3	42.22075	19.11175
1093	2012/120		1	0	[illegible]	0	0	1	42.16773333	19.19595
1095	2012/122		1	0	[illegible]	0	0	1	41.97678333	19.33868333
1104	2012/131		1	0	[illegible]	0	0	1	42.1997	19.81868333
1105	2012/132		1	0	[illegible]	0	0	1	42.19455	19.81771667
1106	2012/133		1	0	[illegible]	0	0	1	42.1892	19.83275
1108	2012/135		1	0	[illegible]	0	0	1	42.23386667	19.87413333
1110	2012/137		1	0	[illegible]	0	0	1	42.2652	19.91791667
1113	2012/140		1	0	[illegible]	0	0	1	42.2279	19.90705
1114	2012/141		1	0	[illegible]	0	0	1	42.10865	19.8264
1119	2012/146		0	0	[illegible]	0	0	1	41.36931667	19.98625
1120	2012/147		0	0	[illegible]	0	0	1	41.36701667	20.0196
1121	2012/148		0	0	[illegible]	0	0	1	41.3512	20.05021667
1122	2012/149		1	0	[illegible]	0	0	1	41.35073333	20.05826667
1125	2012/151		1	0	[illegible]	0	0	1	41.37053333	20.08546667
1126	2012/152		1	0	[illegible]	0	0	3	41.33555	20.15028333
1127	2012/153		0	0	[illegible]	0	0	1	41.33453333	20.1636
1129	2012/155		1	0	[illegible]	0	0	1	41.33923333	20.19886667
1130	2012/156		1	0	[illegible]	0	0	1	41.36035	20.2412
1134	2012/160		1	0	[illegible]	0	0	1	41.38821667	20.26055
1135	2012/161		0	0	[illegible]	0	0	1	41.37555	20.2686
1136	2012/162		1	0	[illegible]	0	0	1	41.33925	20.12878333
1138	2012/164		1	0	[illegible]	0	0	1	41.103	20.00645
1139	2012/165		1	0	[illegible]	0	0	1	41.16343333	20.23005
1141		0	0	0	[illegible]	0	0	1	42.01468	19.62839
1142		0	0	0	[illegible]	0	0	1	40.848529	20.86242
1143		0	0	0	[illegible]	0	0	2	41.25053	20.50336
1144		0	1	0	[illegible]	0	0	1	41.47944	20.08145
1147	2013/001		1	0	[illegible]	0	0	1	41.4091788	22.2527529
1148	2013/002		1	0	[illegible]	0	0	1	39.9965353	21.2855131
1149	2013/003		0	0	[illegible]	0	0	1	40.0076964	21.2805204
1150	2013/004		1	0	[illegible]	0	0	1	40.0079535	21.2846782
1151	2013/005		0	0	[illegible]	0	0	1	40.0521286	21.2393935
1152	2013/006		1	0	[illegible]	0	0	1	40.0741143	21.2016805
1153	2013/007		1	0	[illegible]	0	1	1	39.4602844	21.6007841
1154	2013/008		1	0	[illegible]	0	0	1	39.4569065	21.59613
1155	2013/009		1	0	[illegible]	0	0	1	39.4428565	21.5103952
1156	2013/010		1	0	[illegible]	0	0	1	39.4278403	21.4299602
1157	2013/011		0	0	[illegible]	0	0	1	39.4613997	21.2938452
1158	2013/012		1	0	[illegible]	0	0	1	39.3969136	21.0169787
1159	2013/013		1	0	[illegible]	0	0	1	39.4279064	21.0105573
1160	2013/014A		1	0	[illegible]	0	0	1	39.5668484	21.1230829
1161	2013/014C		0	0	[illegible]	0	0	1	39.5680853	21.1313026
1162	2013/015		0	0	[illegible]	0	0	1	39.5767837	21.0969426
1163	2013/016		1	0	[illegible]	0	0	1	39.5728428	20.986106
1164	2013/017		1	0	[illegible]	0	0	1	39.5762953	20.9771778
1167	2013/020		1	0	[illegible]	0	0	1	39.689521	20.8713509
1168	2013/021		0	0	[illegible]	0	0	1	39.6870318	20.8740156
1169	2013/022		1	0	[illegible]	0	0	1	39.6848876	20.8795284
1170	2013/023		1	0	[illegible]	0	0	1	39.6828228	20.8886677
1171	2013/024		1	0	[illegible]	0	0	1	39.6801669	20.9052407
1172	2013/025		1	0	[illegible]	0	0	1	39.6947455	20.8463411
1173	2013/026		1	0	[illegible]	0	0	1	39.697139	20.8432918
1174	2013/027		1	0	[illegible]	0	0	1	39.7244725	20.8428578
1175	2013/028		0	0	[illegible]	0	0	1	39.7679699	20.6095984
1176	2013/029		1	0	[illegible]	0	0	2	39.7642158	20.616866
1177	2013/030		1	0	[illegible]	0	0	3	39.7670511	20.6147516
1178	2013/031		1	0	[illegible]	0	0	3	39.7720295	20.61797
1179	2013/032		0	0	[illegible]	0	0	1	39.7358415	20.4536566
1180	2013/033		1	0	[illegible]	0	0	1	39.8777481	20.4657989
1181	2013/034		1	0	[illegible]	0	0	1	39.890997	20.7538141
1182	2013/035		1	0	[illegible]	0	0	1	39.8950952	20.7413356
1183	2013/036		1	0	[illegible]	0	0	1	39.9067313	20.751873
1184	2013/037		1	0	[illegible]	0	0	1	39.862	20.7749
1185	2013/038		0	0	[illegible]	0	0	1	39.9674078	20.6645715
1186	2013/039		1	0	[illegible]	0	0	1	40.0467572	20.5648481
1187	2013/040		1	0	[illegible]	0	0	1	40.03549	20.74694
1188	2013/041		1	0	[illegible]	0	0	1	40.1102296	20.7202726
1192	2013/045		0	0	[illegible]	0	0	1	40.3090431	20.8980564
1193	2013/046		0	0	[illegible]	0	0	1	40.3114583	20.8986418
1194	2013/047		0	0	[illegible]	0	0	1	40.3130587	20.8994665
1196	2013/049A		0	0	[illegible]	0	0	1	40.4430603	21.4102248
1198	2013/050		0	0	[illegible]	0	0	1	40.3781789	21.4159985
1201	2013/053		0	0	[illegible]	0	0	1	40.5904817	21.1921266
1202	2013/054		0	0	[illegible]	0	0	1	40.6555158	21.1811746
1203	2013/055		0	0	[illegible]	0	0	1	40.7429279	21.1101852
1205	2013/057		0	0	[illegible]	0	0	1	40.8315864	21.0286694
1207	2013/059		0	0	[illegible]	0	0	1	40.672265	20.9871246
1208	2013/060		0	0	[illegible]	0	0	1	40.7330062	20.87294
1209	2013/061		0	0	[illegible]	0	0	1	40.791891	20.9151703
1210	2013/062		0	0	[illegible]	0	0	1	40.7688301	20.8274532
1211	2013/063		0	0	[illegible]	0	0	1	40.9443849	20.6109453
1213	2013/065		0	0	[illegible]	0	0	1	41.0395881	20.6347365
1214	2013/066		1	0	[illegible]	0	0	1	41.0692523	20.6467607
1215	2013/067		0	0	[illegible]	0	0	2	41.0682353	20.6097743
1216		0	0	0	[illegible]	0	0	3	41.68	21.4399
1217		0	0	0	[illegible]	0	0	1	41.6827	21.4397
1218		0	0	0	[illegible]	0	0	1	40.3983	21.4143
1219		0	0	0	[illegible]	0	0	1	39.681	21.122
1221		0	0	0	[illegible]	0	0	1	40.684722	20.998417
1222		0	0	0	[illegible]	0	0	1	40.2967	20.9433
1224		0	0	0	[illegible]	0	0	1	40.2948	20.9381
1225		0	0	0	[illegible]	0	0	1	40.766806	20.927722
1226		0	0	0	[illegible]	0	0	1	40.3117	20.9072

1228	0	0	[illegible]	0	0	0	0	0	1	40.3449	20.8813
1229	0	0	[illegible]	0	0	0	0	0	1	40.9718	20.8788
1231	0	0	[illegible]	0	0	0	0	0	1	39.6986	20.8482
1234	0	0	[illegible]	0	0	0	0	0	1	39.72765	20.823
1236	0	0	[illegible]	0	0	0	0	0	1	39.7828	20.8041
1239	0	0	[illegible]	0	0	0	0	0	1	39.7345	20.7737
1240	0	0	[illegible]	0	0	0	0	0	1	39.9773	20.7644
1241	0	0	[illegible]	0	0	0	0	0	1	40.0706	20.7608
1252	0	0	[illegible]	0	0	0	0	0	1	41.2234	20.537
1253	0	0	[illegible]	0	0	0	0	0	1	41.203	20.532
1254	0	0	[illegible]	0	0	0	0	0	1	41.35749	20.46226
1256	0	0	[illegible]	0	0	0	0	0	1	39.7822	20.3465
1257	0	0	[illegible]	0	0	0	0	0	1	42.56	20.21
1260	0	0	[illegible]	0	1	0	0	0	1	42.05717	19.39625
1263	0	0	[illegible]	0	1	0	0	0	1	42.3539	19.3416
1264	0	0	[illegible]	0	1	0	0	0	1	42.0387	19.2673
1268	0	0	[illegible]	0	1	0	0	0	1	42.1099	19.2321
1269	0	0	[illegible]	0	1	0	0	0	1	42.1479	19.2191
1270	0	0	[illegible]	0	1	0	0	0	1	42.3132	19.1885
1271	0	0	[illegible]	0	1	0	0	0	1	42.1323	19.1434
1272	0	0	[illegible]	0	1	0	0	0	1	42.067	19.14
1273	0	0	[illegible]	0	1	0	0	0	1	42.1373	19.1398
1274	0	0	[illegible]	0	1	0	0	0	1	42.3917	19.133
1277	0	0	[illegible]	0	1	0	0	0	1	42.1512	19.1125
1278	0	0	[illegible]	0	1	0	0	0	1	42.1829	19.1091
1280	0	0	[illegible]	0	1	0	0	0	1	42.1713	19.0982
1286	0	0	[illegible]	0	1	0	0	0	1	42.2057	19.0559
1289	0	0	[illegible]	0	1	0	0	0	1	42.1998	18.9607
1291	0	0	[illegible]	0	1	0	0	0	1	42.42666667	18.83138889
1294	0	0	[illegible]	0	0	0	0	0	1	42.4116	18.7623
1295	0	0	[illegible]	0	0	0	0	0	1	42.413	18.7615
1296	0	0	[illegible]	0	0	0	0	0	1	42.4901	18.7532
1297	1972/001	0	[illegible]	0	0	0	0	0	1	43.3342	22.0792
1298	1972/002	0	[illegible]	0	0	0	0	0	1	43.3302	22.0636
1300	1972/004	1	[illegible]	0	0	0	0	0	1	41.4322	21.8516
1301	1972/005	0	[illegible]	0	0	0	0	0	1	41.361699	21.703273
1306	1972/010	1	[illegible]	0	0	0	0	0	1	41.8949	21.6864
1316	1972/020	1	[illegible]	0	0	0	0	0	1	40.95113	20.8004
1318	1972/022	1	[illegible]	0	0	0	0	0	1	40.9688	20.7905
1319	1972/023	0	[illegible]	0	0	0	0	0	1	41.1109	20.7915
1320	1972/024	0	[illegible]	0	0	0	0	0	1	41.2626	20.6379
1323	1972/027	1	[illegible]	0	0	0	0	0	1	41.4432	20.5374
1324	1972/028	1	[illegible]	0	0	0	0	0	1	41.554	20.6161
1330	1972/034	0	[illegible]	0	0	0	0	0	1	42.7711	20.2804
1331	1972/035	0	[illegible]	0	0	0	0	0	1	42.9524	20.4189
1332	1972/036	1	[illegible]	0	0	0	0	0	1	42.8544	20.2057
1333	1972/037	1	[illegible]	0	0	0	0	0	1	42.7602	19.3896
1334	1972/038	1	[illegible]	0	1	0	0	0	1	42.3828	19.1022
1336	1972/040	0	[illegible]	0	1	0	0	0	1	42.3367	18.9971
1338	1972/042	0	[illegible]	0	0	0	0	0	1	42.2711	19.1241
1339	1972/043	0	[illegible]	0	1	0	0	0	1	42.2248	19.125
1340	1972/044	0	[illegible]	0	0	0	0	0	1	42.2137	19.0152
1342	1972/046	1	[illegible]	0	0	0	0	0	1	42.5136	18.6816
1344	1972/048	1	[illegible]	0	0	0	0	0	1	42.4282	18.811
1346	1972/050	0	[illegible]	0	0	0	0	0	1	42.4302	18.8128
1347	1972/051	0	[illegible]	0	0	0	0	0	1	42.6275	18.1198
1348	1972/052	1	[illegible]	0	0	0	0	0	1	42.6568	18.0823
1350	1972/054	0	[illegible]	0	0	0	0	0	1	42.5853	18.2169
1352	1972/056	1	[illegible]	0	0	0	0	0	1	42.6708	18.1013
1354	1972/058	0	[illegible]	0	0	0	0	0	1	42.7358	17.5982
1355	1972/059	1	[illegible]	0	0	0	0	0	1	42.7368	17.6005
1356	1972/060	1	[illegible]	0	0	0	0	0	1	42.7316	17.5791
1357	1972/061	1	[illegible]	0	0	0	0	0	1	43.0281	17.651
1358	1972/062	0	[illegible]	0	0	0	0	0	1	43.0178	17.6757
1359	1972/063	1	[illegible]	0	0	0	0	0	1	43.0412	17.6605
1360	1972/064	1	[illegible]	0	0	0	0	0	1	43.4008	16.8642
1363	1972/067	0	[illegible]	0	0	0	0	0	1	44.9811	14.9808
1364	1972/068	0	[illegible]	0	0	0	0	0	1	44.7873	15.3672
1365	1972/069	1	[illegible]	0	0	0	0	0	1	44.9015	15.6087
1366	1976/005	1	[illegible]	0	0	0	0	0	1	39.4771	20.3137
1367	1976/006	0	[illegible]	0	0	0	0	0	1	39.5944	20.4918
1368	1976/007	1	[illegible]	0	0	0	0	0	1	39.6442	20.5129
1369	1976/008	0	[illegible]	0	0	0	0	0	1	39.6393	20.9145
1370	1976/009	0	[illegible]	0	0	0	0	0	1	39.7882	21.3874
1371	1976/010	0	[illegible]	0	0	0	0	0	1	40.1131	22.485
1372	1976/011	1	[illegible]	0	0	0	0	0	1	39.8796	22.587
1374	1976/013	0	[illegible]	0	0	0	0	0	1	39.6991	22.2134
1378	1976/017	1	[illegible]	0	0	0	0	0	1	39.7326	21.0589
1383	1976/023	0	[illegible]	0	0	0	0	0	1	39.4959	20.875
1384	1976/024	1	[illegible]	0	0	0	0	0	1	39.4497	20.879
1385	1976/025	1	[illegible]	0	0	0	0	0	1	39.3607	20.888
1386	1976/026	1	[illegible]	0	0	0	0	0	1	38.6419	20.7089
1387	1976/027	1	[illegible]	0	0	0	0	0	1	38.9877	20.783
1388	1976/028	1	[illegible]	0	0	0	0	0	1	38.5006	21.3674
1389	1976/029	1	[illegible]	0	0	0	0	0	1	38.3929	21.8259
1390	1976/030	0	[illegible]	0	0	0	0	0	1	38.1492	22.2296
1391	1976/031	0	[illegible]	0	0	0	0	0	1	38.1295	22.2172
1392	1976/032	0	[illegible]	0	0	0	0	0	1	38.1206	22.1719
1393	1976/033	0	[illegible]	0	0	0	0	0	1	38.0884	22.1746
1394	1976/034	1	[illegible]	0	0	0	0	0	1	38.0286	21.9965
1395	1976/035	1	[illegible]	0	0	0	0	0	1	38.0674	21.8513
1396	1976/036	1	[illegible]	0	0	0	0	0	1	37.6384	21.6307
1397	1976/037	1	[illegible]	0	0	0	0	0	1	37.6989	21.9392
1398	1976/038	1	[illegible]	0	0	0	0	0	1	37.6815	22.0147
1399	1976/039	0	[illegible]	0	0	0	0	0	1	37.6727	22.0534
1400	1976/040	1	[illegible]	0	0	0	0	0	1	37.6515	22.1685
1401	1976/041	0	[illegible]	0	0	0	0	0	1	37.4101	22.1273
1402	1976/042	1	[illegible]	0	0	0	0	0	1	37.3319	22.0743
1403	1976/043	1	[illegible]	0	0	0	0	0	1	37.0364	22.1663
1404	1976/044	1	[illegible]	0	0	0	0	0	1	37.0412	22.2001
1405	1976/045	1	[illegible]	0	0	0	0	0	1	37.1485	21.9418
1406	1976/046	1	[illegible]	0	0	0	0	0	1	37.1722	21.9179
1407	1976/047	1	[illegible]	0	0	0	0	0	1	36.9845	22.1619
1408	1976/048	1	[illegible]	0	0	0	0	0	1	36.9556	22.1913
1409	1976/049	0	[illegible]	0	0	0	0	0	1	36.9313	22.1915
1410	1976/050	1	[illegible]	0	0	0	0	0	1	37.0298	22.1865
1411	1976/051	0	[illegible]	0	0	0	0	0	1	37.0808	22.2753
1412	1976/052	0	[illegible]	0	0	0	0	0	1	37.0745	22.2955
1413	1976/053	0	[illegible]	0	0	0	0	0	1	37.0739	22.3691
1414	1976/054	0	[illegible]	0	0	0	0	0	1	37.2968	22.4175
1415	1976/055	0	[illegible]	0	0	0	0	0	1	37.3813	22.4034
1416	1976/056	0	[illegible]	0	0	0	0	0	1	37.4537	22.4235
1417	1976/057	0	[illegible]	0	0	0	0	0	1	37.5356	22.5136
1419	1976/059	0	[illegible]	0	0	0	0	0	1	37.5232	22.6404
1421	1976/061	0	[illegible]	0	0	0	0	0	1	37.599	22.8001
1422	1976/062	1	[illegible]	0	0	0	0	0	1	37.7291	22.7577
1423	1976/063	0	[illegible]	0	0	0	0	0	1	37.5936	22.9445
1424	1976/064	0	[illegible]	0	0	0	0	0	1	37.6334	23.1599
1425	1976/065	1	[illegible]	0	0	0	0	0	1	37.7334	23.101
1426	1976/066	1	[illegible]	0	0	0	0	0	1	37.9059	22.8792
1427	1976/068	0	[illegible]	0	0	0	0	0	1	37.9699	23.7334
1428	1976/200	0	[illegible]	0	0	0	0	0	1	37.983023	23.747196
1429	1976/201	0	[illegible]	0	0	0	0	0	1	38.168458	24.231141
1432	1981/B/003	0	[illegible]	0	0	0	0	0	1	41.7689	23.4267
1436	1984/A/002	0	[illegible]	0	0	0	0	0	1	43.61949	22.040561
1437	1984/A/003	0	[illegible]	0	0	0	0	0	1	43.595442	22.095047
1438	1984/A/004	0	[illegible]	0	0	0	0	0	1	43.340293	22.091135
1439	1984/A/005	0	[illegible]	0	0	0	0	0	1	41.52419	23.391398
1442	1984/A/008	0	[illegible]	0	0	0	0	0	1	42.175572	23.981482
1443	1984/A/009	0	[illegible]	0	0	0	0	0	1	42.161665	23.970234
1445	1984/A/011	0	[illegible]	0	0	0	0	0	1	41.683493	24.363566
1447	1984/A/013	0	[illegible]	0	0	0	0	0	1	41.850135	24.683023
1448	1984/A/014	0	[illegible]	0	0	0	0	0	1	41.841983	24.679184
1449	1984/A/015	0	[illegible]	0	0	0	0	0	1	41.879742	24.69584
1450	1984/A/016	1	[illegible]	0	0	0	0	0	1	41.941993	24.849907
1451	1984/A/017	1	[illegible]	0	0	0	0	0	1	41.934101	24.849523
1453	1984/A/019	1	[illegible]	0	0	0	0	0	1	42.685732	26.356405
1454	1984/A/020	1	[illegible]	0	0	0	0	0	1	42.701669	26.350307
1465	1984/A/031	1	[illegible]	0	0	0	0	0	1	42.957798	25.432085
1466	1984/A/032	0	[illegible]	0	0	0	0	0	1	42.748159	25.321604
1467	1984/A/033	1	[illegible]	0	0	0	0	0	1	41.818016	24.589782
1469	1984/A/035	0	[illegible]	0	0	0	0	0	1	41.868899	24.691987
1470	1984/A/036	0	[illegible]	0	0	0	0	0	1	41.987505	24.875516
1471	1984/A/037	0	[illegible]	0	0	0	0	0	1	43.071718	23.398599
1475	1984/A/041	0	[illegible]	0	0	0	0	0	1	43.621563	22.684626
1476	1984/A/042	1	[illegible]	0	0	0	0	0	1	43.651163	22.693433
1477	1984/A/043	1	[illegible]	0	0	0	0	0	1	43.656246	22.716653
1478	1984/A/044	0	[illegible]	0	0	0	0	0	1	43.992936	22.886648
1479	1984/A/045	0	[illegible]	0	0	0	0	0	1	43.727011	22.583722
1480	1984/A/046	0	[illegible]	0	0	0	0	0	1	43.73204	22.577151
1481	1984/A/047	1	[illegible]	0	0	0	0	0	1	44.029196	21.96296
1482	1984/A/048	0	[illegible]	0	0	0	0	0	1	43.856	21.627
1483	1985/C/001	0	[illegible]	0	0	0	0	0	1	44.1834	20.550021
1487	1985/C/005	0	[illegible]	0	0	0	0	0	1	41.891847	21.679516
1490	1985/C/008	1	[illegible]	0	0	0	0	0	1	40.944085	20.789567
1494	1985/C/012	1	[illegible]	0	0	0	0	0	1	40.993184	20.800593
1495	1985/C/013	0	[illegible]	0	0	0	0	0	1	41.358007	20.606138
1498	1985/C/016	0	[illegible]	0	0	0	0	0	1	41.71	20.694
1499	1985/C/017	0	[illegible]	0	0	0	0	0	1	42.692118	20.084411
1501	1985/C/019	0	[illegible]	0	0	0	0	0	1	42.842122	19.870445
1502	1985/C/020	0	[illegible]	0	0	0	0	0	1	42.943649	19.57763
1503	1985/C/021	1	[illegible]	0	0	0	0	0	1	42.23209	19.025966
1504	1985/C/022	0	[illegible]	0	0	0	0	0	1	42.214829	18.95099
1505	1985/C/023	1	[illegible]	0	1	0	0	0	1	42.04756	19.136
1508	1985/C/026	1	[illegible]	0	0	0	0	0	1	42.053714	19.348619
1510	1985/C/028	1	[illegible]	0	1	0	0	0	1	42.431962	19.254932
1511	1985/C/029	1	[illegible]	0	1	0	0	0	1	42.390161	19.11594
1512	1985/C/030	1	[illegible]	0	1	0	0	0	1	42.379008	18.997754
1513	1985/C/031	1	[illegible]	0	0	0	0	0	1	42.433791	18.810255
1514	1985/C/032	1	[illegible]	0	0	0	0	0	1	42.423762	18.800793
1517	1985/C/036	1	[illegible]	0	0	0	0	0	1	42.845041	17.705071
1518	1985/C/037	0	[illegible]	0	0	0	0	0	1	42.876242	17.528588
1519	1985/C/038	1	[illegible]	0	0	0	0	0	1	42.919166	17.423351
1521	1985/C/040	1	[illegible]	0	0	0	0	0	1	42.9655	17.309
1522	1985/C/041	1	[illegible]	0	0	0	0	0	1	43.003484	17.083073
1524	1985/C/043	1	[illegible]	0	0	0	0	0	1	42.719076	17.657937

1525	1985/C/044	1	[illegible]	0	0	1	42.736467	17.599828
1527	1985/C/046	0	[illegible]	0	0	1	42.903278	17.640621
1528	1985/C/047	1	[illegible]	0	0	1	42.969128	17.529852
1529	1985/C/048	0	[illegible]	0	0	1	43.046437	17.475945
1530	1985/C/049	0	[illegible]	0	0	1	43.156082	17.246553
1531	1985/C/050	0	[illegible]	0	0	1	43.368441	16.935677
1534	1985/C/054	1	[illegible]	0	0	1	43.41601	16.907626
1535	1985/C/055	1	[illegible]	0	0	1	43.296438	17.861796
1536	1985/C/056	1	[illegible]	0	0	1	43.281596	18.207305
1537	1985/C/057	0	[illegible]	0	0	1	43.2824	18.262585
1539	1985/C/059	0	[illegible]	0	0	1	43.244063	18.606136
1542	1985/C/062	0	[illegible]	0	0	1	43.293596	18.851268
1543	1985/C/063	0	[illegible]	0	0	1	43.210107	18.858728
1544	1985/C/064	1	[illegible]	0	0	1	42.795624	18.96801
1545	1985/C/065	1	[illegible]	0	0	1	42.862667	19.070286
1548	1985/C/067	0	[illegible]	0	0	1	43.03511	19.392942
1549	1985/C/068	0	[illegible]	0	0	1	42.84201	20.111764
1550	1985/C/069	0	[illegible]	0	0	1	43.131007	20.421484
1551	1985/C/070	1	[illegible]	0	0	1	43.755724	19.232471
1553	1985/C/072	0	[illegible]	0	0	1	43.851662	18.515263
1554	1985/C/073	1	[illegible]	0	0	1	43.736487	18.565115
1557	1985/C/076	1	[illegible]	0	0	1	44.337867	17.270223
1561	1986/E/004	1	[illegible]	0	0	1	41.171945	24.323948
1562	1986/E/005	0	[illegible]	0	0	1	41.227009	24.607051
1563	1986/E/006	1	[illegible]	0	0	1	41.203751	24.723207
1565	1986/E/008	1	[illegible]	0	0	1	40.780752	24.681705
1566	1986/E/009	1	[illegible]	0	0	1	40.644632	24.51576
1567	1986/E/010	0	[illegible]	0	0	1	40.660941	24.691194
1568	1986/E/011	1	[illegible]	0	0	1	40.603636	24.732485
1569	1986/E/012	1	[illegible]	0	0	1	41.082345	24.760755
1570	1986/E/013	1	[illegible]	0	0	1	41.02333333	24.64178333
1571	1986/E/014	1	[illegible]	0	0	1	41.012068	24.28474
1574	1986/E/018	1	[illegible]	0	0	1	39.3076	22.404957
1575	1986/E/019	0	[illegible]	0	0	1	38.796728	22.536564
1576	1986/E/020	0	[illegible]	0	0	1	38.493601	23.160416
1577	1986/E/021	0	[illegible]	0	0	1	38.475762	23.164042
1579	1986/E/075	0	[illegible]	0	0	1	37.989281	23.337772
1580	1986/E/076	0	[illegible]	0	0	1	37.977354	23.260457
1581	1986/E/077	0	[illegible]	0	0	1	37.974496	22.977521
1582	1986/E/078	1	[illegible]	0	0	1	37.832427	23.025536
1583	1986/E/079	1	[illegible]	0	0	1	37.742248	23.114409
1584	1986/E/080	0	[illegible]	0	0	1	37.56682	22.801439
1587	1986/E/083	0	[illegible]	0	0	1	37.533663	22.563775
1588	1986/E/084	0	[illegible]	0	0	1	36.844295	22.605407
1589	1986/E/085	0	[illegible]	0	0	1	36.687741	23.052125
1590	1986/E/086	0	[illegible]	0	0	1	36.674475	23.036393
1591	1986/E/087	0	[illegible]	0	0	1	36.692747	23.031056
1592	1986/E/088	0	[illegible]	0	0	1	36.682394	22.41508
1593	1986/E/089	0	[illegible]	0	0	1	36.625884	22.381854
1594	1986/E/090	0	[illegible]	0	0	1	36.483369	22.402312
1595	1986/E/091	0	[illegible]	0	0	1	36.452475	22.464682
1596	1986/E/092	0	[illegible]	0	0	1	37.068759	22.375788
1597	1986/E/093	0	[illegible]	0	0	1	37.092664	22.352155
1598	1986/E/094	0	[illegible]	0	0	1	37.095257	22.333108
1599	1986/E/095	0	[illegible]	0	0	1	37.091672	22.324695
1600	1986/E/096	0	[illegible]	0	0	1	37.099391	22.232647
1601	1986/E/097	1	[illegible]	0	0	1	37.186082	21.924932
1602	1986/E/098	1	[illegible]	0	0	1	37.63755	21.629628
1603	1986/E/099	0	[illegible]	0	0	1	38.311419	21.78118
1604	1986/E/100	1	[illegible]	0	0	1	38.376707	22.080272
1605	1986/E/101	1	[illegible]	0	0	1	38.482236	22.501286
1606	1986/E/102	0	[illegible]	0	0	1	38.555217	22.406658
1607	1986/E/103	1	[illegible]	0	0	1	38.57392	22.348678
1609	1986/E/105	1	[illegible]	0	0	1	39.68131	20.898251
1610	1986/E/106	1	[illegible]	0	0	1	39.784415	20.738764
1611	1986/E/107	1	[illegible]	0	0	1	40.050345	20.749602
1614	1987/D/001	1	[illegible]	0	0	1	46.369673	14.100606
1615	1987/D/002	1	[illegible]	0	0	1	46.298636	13.943756
1616	1987/D/003	1	[illegible]	0	0	1	46.296556	13.961751
1617	1987/D/004	0	[illegible]	0	0	1	46.323938	14.596928
1618	1987/D/005	1	[illegible]	0	0	1	46.327317	14.589992
1619	1987/D/006	0	[illegible]	0	0	1	46.303703	14.657447
1620	1987/D/007	0	[illegible]	0	0	1	46.298533	14.643751
1621	1987/D/008	1	[illegible]	0	0	1	46.485119	13.785666
1622	1987/D/009	1	[illegible]	0	0	1	46.280004	13.551622
1623	1987/D/010	1	[illegible]	0	0	1	46.108894	13.900068
1624	1987/D/011	1	[illegible]	0	0	1	45.820166	14.290147
1625	1987/D/012	1	[illegible]	0	0	1	45.819192	14.236644
1626	1987/D/013	1	[illegible]	0	0	1	45.294283	13.598113
1627	1987/D/014	1	[illegible]	0	0	1	45.364321	13.758298
1628	1987/D/015	1	[illegible]	0	0	1	45.377726	13.883069
1629	1987/D/016	1	[illegible]	0	0	1	45.086872	14.088397
1630	1987/D/017	0	[illegible]	0	0	1	45.521615	13.869281
1631	1987/D/018	1	[illegible]	0	0	1	45.463534	13.89747
1634	1987/D/021	0	[illegible]	0	0	1	44.52652	15.091466
1635	1987/D/022	1	[illegible]	0	0	1	44.526272	15.171664
1636	1987/D/023	1	[illegible]	0	0	1	44.248375	15.809073
1637	1987/D/024	1	[illegible]	0	0	1	44.220206	15.736781
1639	1987/D/026	1	[illegible]	0	0	1	45.265949	15.223881
1640	1987/D/027	1	[illegible]	0	0	1	45.227777	15.040426
1641	1987/D/028	1	[illegible]	0	0	1	44.99815	15.131953
1642	1987/D/029	1	[illegible]	0	0	1	44.985169	14.923147
1644	1988/C/062	0	[illegible]	0	0	1	38.016204	22.946963
1645	1988/C/063	1	[illegible]	0	0	1	38.027857	22.853764
1646	1988/C/064	1	[illegible]	0	0	1	37.764578	23.124674
1647	1988/C/065	1	[illegible]	0	0	1	37.67959	23.134569
1648	1988/C/066	0	[illegible]	0	0	1	37.570801	23.149432
1649	1988/C/067	0	[illegible]	0	0	1	37.557065	23.374457
1650	1988/C/068	0	[illegible]	0	0	1	37.397067	23.241321
1651	1988/C/069	0	[illegible]	0	0	1	37.521176	22.859773
1652	1988/C/070	0	[illegible]	0	0	1	37.513674	22.736441
1653	1988/C/071	1	[illegible]	0	0	1	37.383882	22.744632
1654	1988/C/072	1	[illegible]	0	0	1	37.339378	22.792704
1655	1988/C/073	0	[illegible]	0	0	1	37.146349	22.892263
1656	1988/C/074	0	[illegible]	0	0	1	37.098999	22.870154
1657	1988/C/075	0	[illegible]	0	0	1	37.164461	22.839021
1658	1988/C/076	0	[illegible]	0	0	1	37.163004	22.810529
1660	1988/C/078	0	[illegible]	0	0	1	37.025115	22.554651
1661	1988/C/079	0	[illegible]	0	0	1	37.008464	22.649885
1662	1988/C/080	0	[illegible]	0	0	1	37.081998	22.282809
1664	1988/C/082	0	[illegible]	0	0	1	37.49026	22.349446
1666	1988/C/084	1	[illegible]	0	0	1	37.487291	21.917913
1667	1988/C/085	1	[illegible]	0	0	1	37.448929	21.910396
1671	1992/A/002	1	[illegible]	0	0	1	37.692119	23.139573
1674	1992/A/005	1	[illegible]	0	0	1	37.889167	22.869722
1675	1992/A/006	1	[illegible]	0	0	1	37.982925	22.97693
1677	1992/A/008	1	[illegible]	0	0	1	37.840649	22.899091
1679	1992/A/010	1	[illegible]	0	0	1	37.76	22.88
1680	1992/A/011	0	[illegible]	0	0	1	37.753545	22.877767
1681	1992/A/012	1	[illegible]	0	0	1	37.756399	22.819928
1682	1992/A/013	1	[illegible]	0	0	1	37.739199	22.878892
1684	1992/A/015	1	[illegible]	0	0	1	37.710454	22.83472
1685	1992/A/016	0	[illegible]	0	0	1	37.638091	22.715355
1686	1992/A/017	0	[illegible]	0	0	1	37.520101	22.729946
1688	1992/A/019	0	[illegible]	0	0	1	37.339458	22.763359
1689	1992/A/020	1	[illegible]	0	0	1	37.328701	22.754276
1690	1992/A/021	0	[illegible]	0	0	1	37.264714	22.846941
1693	1992/A/024	0	[illegible]	0	0	1	37.152598	22.793423
1694	1992/A/025	0	[illegible]	0	0	1	37.143151	22.773645
1703	1992/A/034	0	[illegible]	0	0	1	37.322857	22.14248
1704	1992/A/035	0	[illegible]	0	0	1	37.319287	22.156376
1705	1992/A/036	1	[illegible]	0	0	1	37.26833	22.21764
1706	1992/A/037	0	[illegible]	0	0	1	37.2443	22.247248
1709	1992/A/040	0	[illegible]	0	0	1	37.026496	22.539432
1712	1992/A/043	0	[illegible]	0	0	1	36.939206	22.773784
1714	1992/A/045	1	[illegible]	0	0	1	36.688618	23.054773
1715	1992/A/046	1	[illegible]	0	0	1	36.688185	23.04962
1716	1992/A/047	0	[illegible]	0	0	1	36.773364	22.836695
1717	1992/A/048	1	[illegible]	0	0	1	36.801821	22.492526
1718	1992/A/049	0	[illegible]	0	0	1	36.815865	22.461376
1719	1992/A/050	0	[illegible]	0	0	1	36.978042	22.428122
1720	1992/A/051	0	[illegible]	0	0	1	36.966356	22.388656
1721	1992/A/052	0	[illegible]	0	0	1	36.669997	22.381176
1722	1992/A/053	0	[illegible]	0	0	1	36.638337	22.380651
1723	1992/A/054	1	[illegible]	0	0	1	36.770166	22.346468
1724	1992/A/055	0	[illegible]	0	0	1	36.869246	22.244546
1726	1992/A/057	1	[illegible]	0	0	1	37.648027	21.73378
1727	1992/A/058	0	[illegible]	0	0	1	37.701074	21.909719
1728	1992/A/059	0	[illegible]	0	0	1	37.693193	21.980218
1730	1992/A/061	0	[illegible]	0	0	1	37.674799	22.129208
1731	1992/A/062	0	[illegible]	0	0	1	37.824065	22.142862
1734	1992/A/065	1	[illegible]	0	0	1	38.054329	22.138124
1735	1992/A/066	1	[illegible]	0	0	1	38.368601	22.127928
1736	1992/A/067	0	[illegible]	0	0	1	38.34786	22.313012
1738	1992/A/069	1	[illegible]	0	0	1	38.452649	22.70147
1739	1992/A/070	1	[illegible]	0	0	1	38.171966	23.375409
1740	1994/01	1	[illegible]	0	0	1	45.781	14.205
1741	1994/02	1	[illegible]	0	0	1	45.64	14.17
1742	1994/03	1	[illegible]	0	0	1	45.51	14.29
1744	1994/05	1	[illegible]	0	0	1	45.13	14.83
1745	1994/06	0	[illegible]	0	0	1	44.98	14.99
1746	1994/07	1	[illegible]	0	0	1	44.99	14.98
1747	1994/08	1	[illegible]	0	0	1	44.78	14.76
1748	1994/09	1	[illegible]	0	0	1	44.72	14.92
1749	1994/10	1	[illegible]	0	0	1	45.12	14.83
1750	1994/11	1	[illegible]	0	0	1	45.31	14.72
1751	1994/12	1	[illegible]	0	0	3	45.36	14.77
1752	1994/13	1	[illegible]	0	0	1	45.35	13.76
1753	1994/14	1	[illegible]	0	0	1	45.36	13.75
1754	1994/15	1	[illegible]	0	0	1	45.13	13.76
1760	1995/40	0	[illegible]	0	0	1	44.91	22.37
1761	1995/41	0	[illegible]	0	0	1	45.2	22.32
1763	1995/43	0	[illegible]	0	0	1	41.328	23.354
1764	1995/44	0	[illegible]	0	0	1	40.719	23.65
1766	1995/46	0	[illegible]	0	0	1	40.886	24.072
1767	1995/47	0	[illegible]	0	0	1	40.899	24.045

Table S1.6

1768	1995/48	0	[illegible]	0	0	3	40.913	24.072
1770	1995/50	0	[illegible]	0	0	1	40.799	23.928
1771	1995/51	1	[illegible]	0	0	1	40.725	23.695
1772	1995/52	1	[illegible]	0	0	1	40.752	24.09
1773	1995/53	1	[illegible]	0	0	1	40.745	24.018
1774	1995/54	1	[illegible]	0	0	2	41.017	24.612
1775	1995/55	1	[illegible]	0	0	1	41.02	24.289
1776	1995/56	0	[illegible]	0	0	1	40.739	24.686
1777	1995/57	1	[illegible]	0	0	1	40.739	24.722
1778	1995/58	0	[illegible]	0	0	1	41.208	23.91
1779	1995/59	0	[illegible]	0	0	1	41.288	23.973
1780	1995/60	1	[illegible]	0	0	1	41.06	23.91
1781	1995/61	1	[illegible]	0	0	1	41.208	23.973
1783	1995/63	0	[illegible]	0	0	1	43.04	23.412
1785	1996/02	0	[illegible]	0	0	1	44.14	19.93
1788	1997/18	0	[illegible]	0	0	1	44.29	15.45
1791	1997/21	1	[illegible]	0	0	1	43.91	15.98
1792	1997/22	0	[illegible]	0	0	1	43.8	15.97
1794	1997/24	0	[illegible]	0	0	1	42.94	17.1
1795	1997/25	0	[illegible]	0	0	1	42.93	17.01
1796	1997/26	1	[illegible]	0	0	1	42.99	17.17
1797	1997/27	1	[illegible]	0	0	1	42.99	17.06
1798	1997/28	0	[illegible]	0	0	1	42.96	17.29
1799	1997/29	0	[illegible]	0	0	1	42.924167	17.407
1800	1997/30	0	[illegible]	0	0	1	42.84	17.63
1801	1997/31	0	[illegible]	0	0	1	42.8456	17.704
1803	1997/33	1	[illegible]	0	0	1	43.16	17.24
1804	1997/34	1	[illegible]	0	0	1	43.19	17.17
1805	1997/35	0	[illegible]	0	0	1	43.22	17.12
1806	1997/36	1	[illegible]	0	0	1	43.32	17
1808	1997/38	1	[illegible]	0	0	1	44.87	15.6
1809	1997/40	1	[illegible]	0	0	3	44.88	21.73
1810	1997/41	0	[illegible]	0	0	3	45.03	21.86
1811	1997/42	0	[illegible]	0	0	1	44.88	22.42
1812	1997/43	1	[illegible]	0	0	1	44.88	22.45
1813	1997/44	1	[illegible]	0	0	1	44.96	22.48
1814	1997/45	1	[illegible]	0	0	1	44.71	22.5
1815	1997/46	1	[illegible]	0	0	1	43.78	22.31
1816	1997/47	0	[illegible]	0	0	1	43.5395	22.2888
1817	1997/48	1	[illegible]	0	0	3	41.954	24.844
1818	1997/49	0	[illegible]	0	0	2	41.888	24.661
1819	1997/50	0	[illegible]	0	0	3	41.795	24.607
1820	1997/51	0	[illegible]	0	0	1	41.802	24.57
1821	1997/52	0	[illegible]	0	0	1	42.007	24.89
1822	1997/53	0	[illegible]	0	0	1	42.677	26.325
1828	1997/59	0	[illegible]	0	0	1	42.03	27.624
1832	1997/63	0	[illegible]	0	0	1	42.026	27.477
1833	1997/64	0	[illegible]	0	0	1	45.18	23.82
1834	1997/65	0	[illegible]	0	0	1	45.14	23.14
1835	1997/70	1	[illegible]	0	0	1	45.24	13.93
1836	1997/71	1	[illegible]	0	0	1	45.14	13.73
1839	1997/74	0	[illegible]	0	0	1	45.09	14.13
1840	1997/75	0	[illegible]	0	0	1	45.14	14.19
1842	1997/77	1	[illegible]	0	0	1	45.4	13.97
1843	1997/78	1	[illegible]	0	0	1	45.65	14.02
1845	1998/03	0	[illegible]	0	0	1	45.55	24.26
1847	1998/05	0	[illegible]	0	0	1	45.4	25.15
1848	1998/06	0	[illegible]	0	0	1	45.42	25.21
1849	1998/07	0	[illegible]	0	0	1	45.44	25.23
1850	1998/08	0	[illegible]	0	0	1	45.51	25.36
1851	1998/09	0	[illegible]	0	0	1	45.57	25.26
1852	1998/10	0	[illegible]	0	0	1	45.56	25.28
1853	1998/11	0	[illegible]	0	0	1	45.51	25.5
1854	1998/12	0	[illegible]	0	0	1	45.56	25.6
1855	1998/13	0	[illegible]	0	0	1	45.51	25.89
1856	1998/14	0	[illegible]	0	0	1	45.51	25.9
1857	1998/15	0	[illegible]	0	0	1	45.49	25.93
1858	1998/16	0	[illegible]	0	0	1	45.49	25.94
1859	1998/17	0	[illegible]	0	0	1	45.51	25.93
1860	1998/18	0	[illegible]	0	0	1	45.4926	25.9767
1861	1998/19	0	[illegible]	0	0	1	45.48	25.97
1862	1998/20	0	[illegible]	0	0	1	45.4888	25.9732
1863	1998/21	0	[illegible]	0	0	1	45.4945	25.9717
1864	1998/22	0	[illegible]	0	0	1	45.5	25.95
1865	1998/23	0	[illegible]	0	0	1	45.482	25.93
1866	1998/24	0	[illegible]	0	0	1	45.5	25.9
1867	1998/25	0	[illegible]	0	0	1	45.488	25.892
1868	1998/26	0	[illegible]	0	0	1	45.488	25.877
1869	1998/27	1	[illegible]	0	0	1	44.38	16.09
1871	1998/29	1	[illegible]	0	0	1	43.85	16.16
1872	1998/30	1	[illegible]	0	0	1	43.74	15.9
1873	1998/31	1	[illegible]	0	0	1	43.72	16.22
1874	1998/32	1	[illegible]	0	0	1	43.59	16.5
1875	1998/33	1	[illegible]	0	0	1	43.69	16.54
1876	1998/34	1	[illegible]	0	0	1	43.71	16.57
1877	1998/35	1	[illegible]	0	0	1	43.9138	16.396
1878	1998/36	0	[illegible]	0	0	1	43.4	16.88
1880	1998/38	0	[illegible]	0	0	1	43.11	17.35
1881	1998/39	1	[illegible]	0	0	1	42.57	18.29
1882	1998/40	1	[illegible]	0	0	1	42.56	18.33
1883	1998/41	1	[illegible]	0	0	1	42.6	18.23
1884	1998/42	0	[illegible]	0	0	1	42.59	18.28
1885	1998/43	0	[illegible]	0	0	1	42.6	18.07
1886	1998/44	1	[illegible]	0	0	1	42.8402	17.6956
1888	1998/46	1	[illegible]	0	0	1	43.41	16.9
1890	1998/48	0	[illegible]	0	0	1	43.48	16.84
1891	1998/49	1	[illegible]	0	0	1	43.44	16.9
1892	1998/50	1	[illegible]	0	0	1	43.34	17.11
1893	1998/51	0	[illegible]	0	0	1	43.4545	17.2
1895	1999/01	1	[illegible]	0	0	3	45.22	15.54
1897	1999/03	1	[illegible]	0	0	1	44.58	15.75
1898	1999/04	1	[illegible]	0	0	1	44.25	15.81
1899	1999/05	1	[illegible]	0	0	1	44.25	15.65
1901	1999/07	1	[illegible]	0	0	1	44.18	15.73
1902	1999/08	1	[illegible]	0	0	1	44.011	16.038
1904	1999/10	1	[illegible]	0	0	1	43.89	16.08
1905	1999/100	0	[illegible]	0	0	1	45.38	23.91
1907	1999/102	0	[illegible]	0	0	1	45.41135	23.913183
1908	1999/103	1	[illegible]	0	0	1	46.467367	22.842183
1910	1999/12	1	[illegible]	0	0	1	44.0335	16.1914
1912	1999/13	1	[illegible]	0	0	1	43.92	16.46
1914	1999/15	1	[illegible]	0	0	1	43.76	16.63
1916	1999/17	1	[illegible]	0	0	1	43.71	16.58
1917	1999/18	1	[illegible]	0	0	1	43.68	16.52
1918	1999/19	0	[illegible]	0	0	1	43.69	16.49
1919	1999/20	1	[illegible]	0	0	1	43.58	16.6
1922	1999/23	1	[illegible]	0	0	1	43.458	16.763
1923	1999/24	0	[illegible]	0	0	1	43.44	16.94
1926	1999/27	1	[illegible]	0	0	1	43.08	17.36
1927	1999/28	1	[illegible]	0	0	1	43.04	17.43
1928	1999/29	1	[illegible]	0	0	1	43.14	17.73
1929	1999/30	0	[illegible]	0	0	1	43.26	17.9
1930	1999/31	1	[illegible]	0	0	1	43.44	17.93
1931	1999/32	1	[illegible]	0	0	1	43.42	17.87
1932	1999/33	1	[illegible]	0	0	1	43.28	17.87
1933	1999/34	0	[illegible]	0	0	1	42.98	17.54
1936	1999/37	1	[illegible]	0	0	1	42.92	17.42
1937	1999/38	0	[illegible]	0	0	1	42.7	18.04
1938	1999/39	0	[illegible]	0	0	1	42.62	18.12
1939	1999/40	0	[illegible]	0	0	1	42.61	18.13
1940	1999/41	0	[illegible]	0	0	1	42.58	18.22
1941	1999/42	1	[illegible]	0	0	1	42.63	18.18
1942	1999/43	1	[illegible]	0	0	1	42.64	18.19
1943	1999/44	1	[illegible]	0	0	1	42.64	18.16
1944	1999/45	0	[illegible]	0	0	1	42.65	18.12
1945	1999/46	0	[illegible]	0	0	1	42.99	17.53
1948	1999/49	0	[illegible]	0	0	1	43.31	17.04
1949	1999/50	1	[illegible]	0	0	1	43.78	15.83
1952	1999/52	0	[illegible]	0	0	1	43.57	15.87
1954	1999/54	0	[illegible]	0	0	1	43.18	16.45
1955	1999/55	1	[illegible]	0	0	2	45.65	14.22
1956	1999/56	1	[illegible]	0	0	1	45.32	14.54
1957	1999/57	0	[illegible]	0	0	1	45.03	14.58
1958	1999/58	1	[illegible]	0	0	1	44.97	14.67
1959	1999/59	1	[illegible]	0	0	1	44.93	14.92
1960	1999/60	1	[illegible]	0	0	1	44.53	15.14
1961	1999/61	1	[illegible]	0	0	1	44.4	15.25
1962	1999/62	1	[illegible]	0	0	1	44.3	15.47
1963	1999/63	1	[illegible]	0	0	1	44.325	15.475
1964	1999/64	1	[illegible]	0	0	2	44.35	15.48
1965	1999/65	1	[illegible]	0	0	1	43.96	15.57
1966	1999/66	0	[illegible]	0	0	1	43.95	15.64
1968	1999/68	1	[illegible]	0	0	1	45.35	13.75
1969	1999/69	0	[illegible]	0	0	1	45.118607	13.807904
1971	1999/71	1	[illegible]	0	0	1	45.52	14.18
1972	1999/72	1	[illegible]	0	0	1	45.454033	14.282588
1973	1999/73	1	[illegible]	0	0	1	45.975406	13.650926
1974	1999/74	1	[illegible]	0	0	1	45.998094	13.635327
1975	1999/75	1	[illegible]	0	0	1	46.106428	13.683212
1977	1999/77	1	[illegible]	0	0	1	46.250935	13.581232
1978	1999/78	1	[illegible]	0	0	1	45.87	13.9
1980	1999/80	1	[illegible]	0	0	1	46.27	13.88
1981	1999/88	1	[illegible]	0	0	1	46.42	15.26
1982	1999/89	1	[illegible]	0	0	1	46.34	13.56
1983	1999/90	1	[illegible]	0	0	1	46.3	13.48
1984	1999/92	0	[illegible]	0	0	1	46.04	22.87
1985	1999/93	0	[illegible]	0	0	1	45.76	22.89
1986	1999/94	0	[illegible]	0	0	1	45.53	23.14
1987	1999/95	0	[illegible]	0	0	1	45.45	23.32
1988	1999/96	0	[illegible]	0	0	1	45.47	23.18
1990	1999/98	0	[illegible]	0	0	1	45.16	24.05
1991	1999/99	0	[illegible]	0	0	1	45.22	24.22
1992	2000/01	0	[illegible]	0	0	1	44.68653333	17.1946
1993	2000/02	0	[illegible]	0	0	1	44.60721667	17.13863333
1994	2000/03	1	[illegible]	0	0	1	44.47268333	17.19521667
1995	2000/04	1	[illegible]	0	0	1	44.4105	17.2607

1996	2000/05	1	[illegible]	1	44.347	17.263
1998	2000/07	0	[illegible]	1	44.22948333	17.66846667
1999	2000/08	1	[illegible]	2	44.27886667	17.59206667
2002	2000/11	1	[illegible]	1	43.76993333	18.39406667
2003	2000/12	1	[illegible]	1	43.70976667	18.43921667
2004	2000/13	1	[illegible]	1	43.57601667	18.53733333
2005	2000/62	1	[illegible]	1	42.7	18.32
2006	2000/63	0	[illegible]	1	43.055	18.506
2007	2000/64	0	[illegible]	1	43.231649	18.40225
2008	2000/65	0	[illegible]	1	43.24776667	18.38158333
2009	2000/66	0	[illegible]	1	43.25101667	18.33955
2010	2000/67	1	[illegible]	1	43.35058333	17.89568333
2011	2000/68	0	[illegible]	1	43.6674	17.95896667
2012	2000/86	0	[illegible]	1	45.35648333	23.85803333
2013	2000/87	0	[illegible]	1	45.41753333	23.9158
2014	2000/88	0	[illegible]	1	45.39733333	23.904
2015	2000/88B	0	[illegible]	1	45.39445	23.91086667
2016	2000/89	0	[illegible]	1	45.55243333	25.89996667
2017	2000/90	0	[illegible]	1	45.56693333	25.88425
2018	2000/91	0	[illegible]	1	45.5461	25.8905
2019	2000/92	0	[illegible]	1	45.55161667	25.90681667
2020	2000/93	0	[illegible]	1	45.56238333	25.93621667
2021	2000/94	0	[illegible]	1	45.55963333	25.89625
2022	2000/95	0	[illegible]	1	45.55736667	25.89158333
2023	2000/96	0	[illegible]	1	45.56066667	25.8836
2024	2000/98	0	[illegible]	1	45.53561667	25.90751667
2025	2000/99	0	[illegible]	1	45.56756667	25.60383333
2027	2002/001	0	[illegible]	1	43.333195	22.141906
2028	2002/002	0	[illegible]	1	43.33568333	22.13305
2030	2002/056	1	[illegible]	1	45.36228	14.760714
2031	2002/057	0	[illegible]	1	45.30163333	14.67318333
2032	2002/058	1	[illegible]	1	45.29755	14.65805
2033	2002/059	1	[illegible]	1	45.28635	14.64531667
2034	2002/060	1	[illegible]	1	45.28463333	14.62855
2035	2002/061	0	[illegible]	1	45.324709	14.538775
2036	2002/062	1	[illegible]	1	45.33175	14.54301667
2037	2002/063	1	[illegible]	1	44.98261667	14.9754
2038	2002/064	0	[illegible]	1	44.96866	14.973473
2039	2002/065	1	[illegible]	1	44.97933333	14.94933333
2040	2002/066	1	[illegible]	1	44.52836667	15.14398333
2041	2002/067	0	[illegible]	1	44.44111667	15.51913333
2042	2002/068	1	[illegible]	1	44.20158333	15.69965
2043	2002/069	0	[illegible]	1	43.93758333	15.44123333
2044	2002/070	1	[illegible]	1	43.5279	15.9689
2045	2002/071	1	[illegible]	1	43.55986667	16.40255
2046	2002/072	1	[illegible]	1	43.5092	16.42173333
2047	2002/073	0	[illegible]	1	43.56105	16.5228
2048	2002/074	1	[illegible]	1	43.56001667	16.51918333
2049	2002/075	0	[illegible]	1	43.56223333	16.52906667
2050	2002/076	1	[illegible]	1	43.44803333	16.69458333
2051	2002/077	0	[illegible]	1	43.42926667	16.86666667
2052	2002/078	1	[illegible]	1	43.43131667	16.88768333
2053	2002/079	1	[illegible]	1	43.40301667	16.89371667
2054	2002/080	1	[illegible]	1	43.29345	17.0519
2055	2002/081	1	[illegible]	1	43.25765	17.09398333
2056	2002/082	0	[illegible]	1	43.31243333	17.06221667
2057	2002/083	1	[illegible]	1	43.31596667	17.05396667
2058	2002/084	0	[illegible]	1	43.2586	17.10341667
2059	2002/085	1	[illegible]	1	43.23421667	17.1046
2060	2002/086	0	[illegible]	1	43.22788333	17.10986667
2061	2002/087	1	[illegible]	1	43.44278333	16.70153333
2062	2002/088	0	[illegible]	1	43.40358333	16.88048333
2063	2002/089	0	[illegible]	1	43.83178333	17.00788333
2064	2002/090	0	[illegible]	1	44.34876667	16.36025
2065	2002/091	1	[illegible]	1	44.17978333	19.87128333
2066	2004/071	1	[illegible]	1	45.334736	13.827767
2067	2004/072	1	[illegible]	1	45.368641	14.015313
2068	2004/073	0	[illegible]	1	45.348651	14.050464
2071	2005/025	0	[illegible]	1	46.56373333	23.6796
2072	2005/039	0	[illegible]	3	45.171	19.82625
2073	2005/040	0	[illegible]	1	44.16038333	19.88308333
2082	2006/096	0	[illegible]	1	43.9065	15.97451667
2087	2006/101	0	[illegible]	1	43.3814	16.9108
2100	2007/004	0	[illegible]	1	41.28333333	23.33323333
2102	2007/006	0	[illegible]	3	41.39915	23.50523333
2103	2007/007	0	[illegible]	3	41.39273333	23.4776
2109	2007/013	0	[illegible]	1	41.34541667	23.67438333
2111	2007/015	0	[illegible]	1	41.28466667	23.85523333
2112	2007/016	0	[illegible]	1	41.2211	23.89316667
2113	2007/017	0	[illegible]	2	41.28955	24.02718333
2114	2007/018	0	[illegible]	3	41.29303333	24.00703333
2117	2007/021	0	[illegible]	1	41.38876667	24.16796667
2119	2007/023	0	[illegible]	1	41.42803333	24.21821667
2127	2007/031	0	[illegible]	1	41.30973333	24.5066
2128	2007/032	0	[illegible]	2	41.1662	24.64311667
2129	2007/033	0	[illegible]	1	41.15873333	24.60915
2130	2007/034	0	[illegible]	1	41.03515	24.63235
2131	2007/035	0	[illegible]	1	41.02708333	24.63613333
2137	2007/041	0	[illegible]	1	41.09325	24.7713
2139	2007/043	0	[illegible]	1	41.20825	24.86253333
2142	2007/046	0	[illegible]	3	41.29185	24.82905
2143	2007/047	0	[illegible]	1	41.29141667	24.84611667
2145	2007/048	0	[illegible]	1	41.24546667	24.90455
2146	2007/049	0	[illegible]	1	41.24673333	24.9137
2149	2007/052	0	[illegible]	1	41.18175	25.28118333
2151	2007/054	0	[illegible]	1	41.1902	25.29586667
2152	2007/055	0	[illegible]	1	41.11408333	25.8388
2154	2007/057	0	[illegible]	1	40.93728333	25.9117
2156	2007/059	0	[illegible]	2	40.90133333	25.6058
2157	2007/060	0	[illegible]	1	40.92886667	25.5023
2160	2007/063	0	[illegible]	1	40.64511667	27.01278333
2173	2007/076	0	[illegible]	1	41.83931667	27.55618333
2175	2007/078	0	[illegible]	3	42.02935	27.47363333
2176	2007/079	0	[illegible]	1	42.08348333	27.4277
2188	2008/084	0	[illegible]	1	44.51944444	14.5075
2189	2008/085	0	[illegible]	1	44.69333333	14.39222222
2190	2008/086	1	[illegible]	1	44.908	14.35593333
2191	2008/087	1	[illegible]	1	44.88881667	14.33096667
2192	2008/088	1	[illegible]	1	44.98111667	14.45193333
2193	2008/089	0	[illegible]	1	45.21166667	14.55638889
2194	2008/101	1	[illegible]	1	43.45548333	16.92051667
2196	2008/103	1	[illegible]	1	43.44933333	17.2112
2197	2008/104	0	[illegible]	1	43.44995	17.21423333
2198	2008/105	1	[illegible]	1	43.06936667	17.41998333
2199	2008/106	0	[illegible]	1	42.78776667	17.89316667
2200	2008/107	0	[illegible]	1	42.70488333	17.99001667
2201	2008/108	0	[illegible]	2	42.64276667	18.1226
2202	2008/109	0	[illegible]	1	42.5346	18.37683333
2211	2009/095	0	[illegible]	1	44.71026667	16.63946667
2218	2009/102	0	[illegible]	1	44.61281667	17.98883333
2223	2009/107	0	[illegible]	1	43.83841667	17.59053333
2224	2009/108	0	[illegible]	1	43.81568333	17.61398333
2227	2009/111	0	[illegible]	1	43.65678333	17.94911667
2228	2009/112	0	[illegible]	1	43.67118333	17.98403333
2230	2009/114	0	[illegible]	1	43.80691667	18.18841667
2232	2009/116	0	[illegible]	3	43.95118333	18.0881
2235	2009/119	0	[illegible]	1	43.81911667	18.95545
2236	2009/120	0	[illegible]	1	43.78521667	19.01055
2237	2009/121	0	[illegible]	1	43.7395	19.00311667
2238	2009/122	0	[illegible]	1	43.75431667	19.41788333
2239	2009/130	1	[illegible]	1	44.02966667	22.22335
2245	2009/136	0	[illegible]	3	43.13558333	23.14086667
2247	2009/138	1	[illegible]	1	43.20453333	23.4936
2248	2009/139	1	[illegible]	1	43.1907	23.53028333
2250	2009/141	0	[illegible]	3	42.9735	24.1594
2251	2009/142	0	[illegible]	3	42.97863333	24.16246667
2255	2009/146	0	[illegible]	1	43.18118333	24.07088333
2261	2009/152	0	[illegible]	1	43.70436667	24.89595
2264	2009/155	0	[illegible]	1	43.15883333	22.58093333
2265	2009/156	0	[illegible]	1	43.18791667	22.59583333
2267	2009/158	0	[illegible]	1	43.32	22.17316667
2276	2011/010	1	[illegible]	1	39.95808333	23.92501667
2277	2011/011	0	[illegible]	2	40.37558333	23.16671667
2279	2012/213	1	[illegible]	1	42.9131	17.4228
2281	2014/002	0	[illegible]	2	41.0505	20.6441
2282	2014/003	1	[illegible]	2	41.0632	20.6253
2286	2014/007	1	[illegible]	1	41.0042	20.4343
2289	2014/010	0	[illegible]	1	41.2719	20.2839
2291	2014/012	0	[illegible]	2	41.2727	20.2703
2292	2014/013	0	[illegible]	1	41.2672	20.261
2293	2014/014	0	[illegible]	2	41.2652	20.2422
2294	2014/015	1	[illegible]	1	41.2802	20.21
2297	2014/018	1	[illegible]	1	41.2776	20.3375
2298	2014/019	1	[illegible]	1	41.2966	20.3741
2299	2014/020	0	[illegible]	1	41.3611	20.3967
2300	2014/021	0	[illegible]	1	41.3462	20.3904
2301	2014/022	0	[illegible]	1	41.3339	20.3958
2302	2014/023	1	[illegible]	3	41.3238	20.4224
2303	2014/024	0	[illegible]	1	41.325	20.4457
2304	2014/025	0	[illegible]	1	41.3646	20.4685
2305	2014/026	0	[illegible]	1	41.3792	20.4687
2307	2014/028	0	[illegible]	1	41.4199	20.4633
2308	2014/029	1	[illegible]	1	41.4404	20.5052
2312	2014/033	1	[illegible]	1	41.6097	20.4431
2314	2014/035	1	[illegible]	1	41.6308	20.2983
2315	2014/036	1	[illegible]	1	41.628	20.2741
2316	2014/037	0	[illegible]	1	41.6228	20.2886
2317	2014/038	0	[illegible]	1	41.6541	20.3129
2321	2014/042	0	[illegible]	1	41.514	20.316
2324	2014/045	1	[illegible]	1	41.4677	20.1048
2325	2014/046	0	[illegible]	1	41.4685	20.0904
2326	2014/047	1	[illegible]	3	41.4361	20.0485
2329	2014/050	1	[illegible]	1	41.6919	19.8318
2330	2014/051	1	[illegible]	1	41.7002	19.7052
2331	2014/052	0	[illegible]	3	41.6388	19.7286
2332	2014/053	0	[illegible]	1	41.6296	19.733

2334	2014/055	1	0	1	41.4412	19.8636
2335	2014/056	1	0	1	41.3948	19.9
2336	2014/057	1	0	1	41.2598	19.9676
2338	2014/058	1	0	1	41.1005	20.1249
2343	2014/063	1	0	1	43.85131809	19.82230472
2346	2014/066	0	0	1	42.39132207	19.73029716
2348	2014/068	1	0	1	42.13060297	19.71060824
2349	2014/069	1	0	1	42.12730469	19.71185186
2350	2014/070	0	0	1	41.85994897	20.118936
2351	2014/071	1	0	1	41.85607913	20.12075471
2352	2014/072	0	0	3	41.84553872	20.12990389
2353	2014/073	1	0	3	41.8524242	20.12344865
2354	2014/073B	0	0	1	41.85440015	20.12372442
2355	2014/074	0	0	1	41.84756345	20.11680348
2356	2014/075	0	0	1	41.84325482	20.11186
2357	2014/076	1	0	1	41.85947715	20.14933557
2358	2014/077	0	0	1	41.87470883	20.16569914
2359	2014/078	1	0	1	41.87930086	20.16633684
2360	2014/079	1	0	1	41.88691397	20.17148634
2361	2014/080	0	0	1	41.89182946	20.1873734
2362	2014/081	1	0	3	41.89971976	20.19479549
2363	2014/082	0	0	1	41.90106925	20.19663281
2364	2014/083	0	0	3	41.89704459	20.19823501
2365	2014/083B	0	0	3	41.89556535	20.1974347
2367	2014/085	0	0	1	41.88615172	20.18682674
2368	2014/086	0	0	1	41.88390059	20.1724915
2369	2014/087	0	0	1	41.9201	20.1989
2370	2014/089	0	0	1	41.92943822	20.18502354
2371	2014/090	0	0	3	41.88749274	20.17427199
2372	2014/091	1	0	3	42.55327636	20.24923646
2373	2014/092	1	0	1	42.43131464	20.14990035
2374	2014/093	0	0	3	42.45462262	20.15778135
2375	2014/094	1	0	3	42.4692	20.1554
2376	2014/095	1	0	3	42.474	20.152
2378	2014/097	1	0	1	40.70414077	19.94787167
2381	2014/100	1	0	1	40.11851538	20.0142897
2382	2014/101	1	0	1	40.11658821	20.00020718
2383	2014/102	1	0	1	40.0949	19.991734
2384	2014/103	1	0	1	40.08816979	19.96313788
2385	2014/104	1	0	1	40.27575167	20.09470141
2386	2014/105	0	0	1	40.258292	20.07898903
2388	2014/107	1	0	1	40.29608474	20.10760124
2389	2014/108	1	0	3	40.12257448	20.48048562
2391	2014/110	0	0	1	40.24180354	20.64135958
2394	2014/113	1	0	1	40.73013313	20.50389863
2395	2014/114	1	0	3	40.74798541	20.52191997
2396	2014/115	1	0	1	40.74616964	20.51225656
2398	2014/117	1	0	1	41.00030777	20.24742781
2400	2014/119	0	0	1	40.96267	20.81707
2402	2014/121	0	0	1	40.914	20.7406
2403	2014/122	0	0	1	41.1147	20.7876
2404	BPP2014/5	0	0	2	41.85691	19.50439
2405	BPP2014/14A	0	0	2	40.42874	19.33526
2406	BPP2014/19	1	0	2	39.7175	20.01643
2407	BPP2014/23D	1	0	2	39.90273	20.17079
2408	BPP2014/25	1	0	2	40.24988	20.11788
2409	BPP2014/25B	1	0	1	40.24512	20.11061
2410	BPP2014/26	0	0	1	40.49264	19.72609
2412	2014/123	0	0	3	41.65089098	20.71347621
2413	2014/124	0	0	3	41.5942097	20.67159771
2414	2014/125	0	0	1	41.59357175	20.65752852
2415	2014/126	0	0	1	41.62395129	20.67683933
2416	2014/127	0	0	1	41.64226826	20.69765336
2417	2014/128	0	0	1	41.64685031	20.70980569
2418	2014/129	0	0	1	41.71171619	20.71232286
2419	2014/130	0	0	1	41.72026657	20.66822106
2420	2014/131	0	0	1	41.76549599	20.66659589
2421	2014/132	0	0	1	41.7102	20.6564
2422	2014/133	0	0	1	41.70793352	20.6500164
2423	2014/134	0	0	1	41.69940996	20.64784809
2424	2014/135	0	0	2	41.49481357	20.50522431
2425	2014/136	0	0	1	41.44393735	20.55075321
2426	2014/137	0	0	1	41.2997583	20.6200454
2427	2014/138	0	0	1	41.32985277	20.63693494
2428	2014/139	1	0	1	41.3397	20.6273
2430	2014/141	1	0	1	41.35451392	20.61244343
2431	2014/142	0	0	1	41.36230314	20.60361788
2432	2014/143	1	0	1	41.36378967	20.60489343
2433	2014/144	1	0	1	41.36664086	20.60492738
2434	2014/145	0	0	1	41.36720781	20.60463049
2435	2014/146	1	0	1	41.36945827	20.60207795
2436	2014/147	0	0	1	41.37047214	20.59794609
2437	2014/148	0	0	1	41.37134554	20.59709994
2438	2014/149	0	0	1	41.40042353	20.58493369
2439	2014/150	1	0	1	41.39784526	20.58994733
2440	2014/151	0	0	1	41.38158244	20.58807707
2441	2014/152	0	0	1	41.22019627	20.91150999
2443	2014/154	0	0	1	41.084319	20.79733053
2444	2014/155	0	0	2	40.99335699	20.85693542
2445	2014/156	0	0	2	41.0026045	20.85337671
2447	2014/158	0	0	2	41.00331821	20.84876633
2448	2014/159	1	0	2	41.00371292	20.84695542
2449	2014/160	1	0	2	41.00456486	20.84318834
2450	2014/161	1	0	2	41.00506509	20.84616811
2451	2014/162	1	0	1	40.95738078	20.77780623
2452	2014/163	1	0	2	40.9937315	20.79917514
2453	2014/164	1	0	1	41.00917499	20.80557724
2454	2014/165	1	0	1	41.03454868	20.81052901
2455	2014/166	0	0	1	40.96742992	20.81490646
2456	2014/167	0	0	1	40.89242723	20.9393851
2457	2014/168	0	0	1	40.85800904	20.94259998
2458	2014/169	0	0	2	40.81220319	20.90447774
2459	2014/170	0	0	1	40.6344356	21.09154883
2460	2014/171	1	0	1	40.52332627	21.27467615
2462	2014/173	0	0	1	40.80872211	20.99792314
2463	2014/174	0	0	1	40.81996283	21.05221474
2464	2014/175	0	0	1	40.79977711	21.07147157
2465	2014/176	0	0	1	40.71921872	21.03408761
2466	2014/177	0	0	1	40.81053067	21.07019073
2467	2014/178	0	0	1	40.65323889	21.17894132
2468	2014/179	0	0	2	41.37982568	21.74413774
2473	2015/004	1	0	1	42.643544	19.377819
2474	2015/005	1	0	1	42.62853	19.372128
2475	2015/006	1	0	1	42.37066	19.313889
2476	2015/007	0	0	1	42.317159	19.156679
2477	2015/008	0	0	1	42.314265	19.185085
2478	2015/009	0	0	1	42.280996	19.137376
2479	2015/010	0	0	1	42.245994	19.091719
2480	2015/011	0	0	1	42.220461	19.12407
2481	2015/012	1	0	1	42.225604	19.065948
2482	2015/013	0	0	1	42.251329	19.087318
2483	2015/014	0	0	1	42.252115	19.087521
2484	2015/015	1	0	1	42.254687	19.089632
2485	2015/016	1	0	1	42.305786	19.04807
2486	2015/017	1	0	1	42.352334	19.004744
2488	2015/019	1	0	1	42.365065	18.992377
2489	2015/020	1	0	1	42.381462	19.008372
2490	2015/021	1	0	1	42.386509	19.057116
2491	2015/022	0	0	1	42.375799	18.986275
2492	2015/023	1	0	1	42.400564	18.915211
2493	2015/024	1	0	1	42.305591	18.940213
2494	2015/025	1	0	1	42.227072	18.911729
2495	2015/026	0	0	1	42.217765	18.954533
2496	2015/027	1	0	1	42.164913	19.006938
2497	2015/028	1	0	1	42.153035	19.023731
2498	2015/029	1	0	1	42.137167	19.047373
2500	2015/031	0	0	1	42.057189	19.127777
2504	2015/035	0	0	1	42.050584	19.416234
2505	2015/036	0	0	1	42.051539	19.417129
2506	2015/037	1	0	1	42.052444	19.418228
2508	2015/039	0	0	1	41.898021	19.442456
2509	2015/040	0	0	1	41.857694	19.492337
2510	2015/041	0	0	1	41.876295	19.459235
2511	2015/042	0	0	1	41.883146	19.453315
2512	2015/043	1	0	1	41.916055	19.426763
2513	2015/044	0	0	1	41.927042	19.438694
2516	2015/047	1	0	1	42.058248	19.353537
2517	2015/048	1	0	1	42.190589	18.989795
2518	2015/049	1	0	1	42.431244	18.754857
2519	2015/050	1	0	1	42.4269	18.7756
2521	2015/052	0	0	1	42.409482	18.778685
2522	2015/053	1	0	1	42.399268	18.783629
2523	2015/054	1	0	1	42.431639	18.812576
2526	2015/057	1	0	1	42.487689	18.560329
2527	2015/058	0	0	1	42.445502	18.504971
2528	2015/059	1	0	1	42.731904	18.938231
2529	2015/060	1	0	1	42.675074	19.029887
2534	2015/065	0	0	3	40.94447231	20.82700524
2535	2015/065B	0	0	2	40.94391751	20.82827174
2536	2015/065C	0	0	1	40.9429225	20.82516398
2537	2015/066	0	0	1	40.88723372	20.97285219
2538	2015/067	0	0	1	40.88087177	20.95902013
2540	2015/069	0	0	1	40.86990623	20.92949186
2541	2015/070	0	0	1	40.84218845	20.96164442
2542	2015/071	0	0	1	40.8584	20.9474
2544	2015/073	0	0	1	41.30277654	20.62352984
2545	2015/074	0	0	3	41.39583377	20.58952983
2546	2015/075	0	0	1	41.39471672	20.59765541
2551	2015/079	0	0	1	41.85151082	20.32823372
2552	2015/080	0	0	1	41.84653482	20.33040832
2555	2015/083	1	0	1	41.88304371	20.33700555
2556	2015/084	0	0	1	41.9253802	20.35690787
2558	2015/086	0	0	1	41.94538445	20.37157402
2559	2015/087	1	0	1	41.95019332	20.38137884
2560	2015/088	0	0	1	42.10154166	20.42091854
2561	2015/089	0	0	1	42.19204559	20.41665978

2562	2015/090		0	0	0	0	0	1	0	0	0	0	0	0	1	0	0	0	0	0	1	0	0	0	0	0	0	0	0	0	0	0	0	0	0	0	0	0	1	0	0	0	0	0	0	0	0	0	0	0	0	0	0	0	0	0	0	0	0	0	0	0	0	0	0	0	0	0	0	0	0	0	0	0	0	1	42.34468063	20.03529819
2563	2015/091		1	0	0	0	0	1	0	0	1	1	0	0	1	1	0	0	1	0	1	0	0	0	0	0	1	0	0	0	0	1	0	0	0	0	0	1	0	0	0	0	0	0	0	0	0	1	0	0	0	0	0	0	0	0	0	0	0	0	0	0	0	0	0	0	0	0	0	0	0	0	0	0	0	3	42.36687088	20.02698384
2564	2015/092		1	0	0	0	0	0	0	0	1	0	0	0	1	0	0	0	0	0	1	0	0	0	0	0	0	0	0	0	0	1	0	0	0	0	0	1	0	0	0	0	0	0	0	0	0	1	0	0	0	0	0	0	0	0	0	0	0	0	0	0	0	0	0	0	0	0	0	0	0	0	0	0	0	3	42.36780253	20.009351
2567	2015/094		0	0	0	0	0	0	0	0	0	0	0	0	0	0	0	0	0	0	0	0	0	0	0	0	0	0	0	0	0	0	0	0	0	0	0	0	1	0	0	0	0	0	0	0	0	0	0	0	0	0	0	0	0	0	0	0	0	0	0	0	0	0	0	0	0	0	0	0	0	0	0	0	0	1	42.35562044	20.0701711
2574	2015/101		1	0	0	0	0	0	0	0	0	1	0	0	0	0	0	0	0	0	1	0	0	0	0	1	0	0	0	0	0	0	0	0	0	0	0	0	1	0	0	0	0	0	0	0	0	0	0	0	0	0	0	0	0	0	0	0	0	0	0	0	0	0	0	0	0	0	0	0	0	0	0	0	0	1	42.12477369	19.61498422
2575	2015/102		1	0	0	0	0	1	0	0	0	1	0	0	0	0	0	0	0	0	0	0	0	0	0	0	0	0	0	0	0	0	0	0	0	0	0	0	1	0	0	0	0	0	0	0	0	0	0	0	0	0	0	0	0	0	0	0	0	0	0	0	0	0	0	0	0	0	0	0	0	0	0	0	0	1	42.32498316	19.59398403
2576	2015/103		1	0	1	0	0	1	0	0	0	1	0	0	0	0	1	0	0	0	0	0	0	0	0	1	0	0	0	0	0	0	0	0	0	0	0	0	1	0	0	0	0	0	0	0	0	0	0	0	0	0	0	0	0	0	0	0	0	0	0	0	0	0	0	0	0	0	0	0	0	0	0	0	0	1	42.33062913	19.60567578
2577	2015/104		1	0	0	0	0	0	0	0	0	0	0	0	0	0	0	0	0	0	0	0	0	0	0	0	0	0	0	0	0	0	0	0	0	0	0	0	0	0	0	0	0	0	0	0	0	0	0	0	0	0	0	0	0	0	0	0	0	0	0	0	0	0	0	0	0	0	0	0	0	0	0	0	0	1	42.276362	19.516466
2578	2015/105		0	0	0	0	0	0	0	0	0	1	0	0	0	0	0	0	0	0	0	0	0	0	0	1	0	0	0	0	0	0	0	0	0	0	0	0	0	0	0	0	0	0	0	0	0	0	0	0	0	0	0	0	0	0	0	0	0	0	0	0	0	0	0	0	0	0	0	0	0	0	0	0	0	1	42.31955177	19.36934819
2581		0	1	0	0	0	0	0	0	0	0	0	0	0	0	0	0	0	0	0	1	0	0	0	0	0	0	0	0	0	0	0	0	0	0	0	0	0	0	0	0	0	0	0	0	0	0	0	0	0	0	0	0	0	0	0	0	0	0	0	0	0	0	0	0	0	0	0	0	0	0	0	0	0	0	3	46.378056	14.207361
2582		0	0	0	0	0	0	0	0	0	0	0	0	0	0	0	0	0	0	0	1	0	0	0	0	0	0	0	0	0	0	0	0	0	0	0	0	0	0	0	0	0	0	0	0	0	0	0	0	0	0	0	0	0	0	0	0	0	0	0	0	0	0	0	0	0	0	0	0	0	0	0	0	0	0	3	40.107694	21.021167
2583		0	0	0	0	0	0	0	0	0	0	0	0	0	0	0	0	0	0	0	1	0	0	0	0	0	0	0	0	0	0	0	0	0	0	0	0	0	0	0	0	0	0	0	0	0	0	0	0	0	0	0	0	0	0	0	0	0	0	0	0	0	0	0	0	0	0	0	0	0	0	0	0	0	0	1	44.60935	22.274417
2584	DL85		0	0	0	0	0	0	0	0	0	0	0	0	0	0	0	0	0	0	1	0	0	0	0	0	0	0	0	0	0	0	0	0	0	0	0	0	0	0	0	0	0	0	0	0	0	0	0	0	0	0	0	0	0	0	0	0	0	0	0	0	0	0	0	0	0	0	0	0	0	0	0	0	0	1	45.029186	14.497518
2585	DL250		0	0	0	0	0	0	0	0	0	0	0	0	0	0	0	0	0	0	1	0	0	0	0	0	0	0	0	0	0	0	0	0	0	0	0	0	0	0	0	0	0	0	0	0	0	0	0	0	0	0	0	0	0	0	0	0	0	0	0	0	0	0	0	0	0	0	0	0	0	0	0	0	0	1	44.6988	14.9082
2586	DL152		0	0	0	0	0	0	0	0	0	0	0	0	0	0	0	0	0	0	1	0	0	0	0	0	0	0	0	0	0	0	0	0	0	0	0	0	0	0	0	0	0	0	0	0	0	0	0	0	0	0	0	0	0	0	0	0	0	0	0	0	0	0	0	0	0	0	0	0	0	0	0	0	0	1	44.292212	15.175082
2587		0	1	0	0	0	0	0	0	0	0	0	0	0	0	0	0	0	0	0	0	0	0	0	0	0	0	0	0	0	0	0	0	0	0	0	0	0	0	0	0	0	0	0	0	0	0	0	0	0	0	0	0	0	0	0	0	0	0	0	0	0	0	0	0	0	0	0	0	0	0	0	0	0	0	1	43.511722	18.671806
2588		0	1	0	0	0	0	0	0	0	0	0	0	0	0	0	0	0	0	0	0	0	0	0	0	0	0	0	0	0	0	0	0	0	0	0	0	0	0	0	0	0	0	0	0	0	0	0	0	0	0	0	0	0	0	0	0	0	0	0	0	0	0	0	0	0	0	0	0	0	0	0	0	0	0	1	43.724183	18.604183
2589		0	1	0	0	0	0	0	0	0	0	0	0	0	0	0	0	0	0	0	0	0	0	0	0	0	0	0	0	0	0	0	0	0	0	0	0	0	0	0	0	0	0	0	0	0	0	0	0	0	0	0	0	0	0	0	0	0	0	0	0	0	0	0	0	0	0	0	0	0	0	0	0	0	0	1	43.7661	18.54655
2590		0	1	0	0	0	0	0	0	0	0	0	0	0	0	0	0	0	0	0	0	0	0	0	0	0	0	0	0	0	0	0	0	0	0	0	0	0	0	0	0	0	0	0	0	0	0	0	0	0	0	0	0	0	0	0	0	0	0	0	0	0	0	0	0	0	0	0	0	0	0	0	0	0	0	3	46.366542	14.306749
2591	DL20		1	0	0	0	0	0	0	0	0	0	0	0	0	0	0	0	0	0	0	0	0	0	0	0	0	0	0	0	0	0	0	0	0	0	0	0	0	0	0	0	0	0	0	0	0	0	0	0	0	0	0	0	0	0	0	0	0	0	0	0	0	0	0	0	0	0	0	0	0	0	0	0	0	3	46.406944	14.146111
2592	DL246		1	0	0	0	0	0	0	0	0	0	0	0	0	0	0	0	0	0	0	0	0	0	0	0	0	0	0	0	0	0	0	0	0	0	0	0	0	0	0	0	0	0	0	0	0	0	0	0	0	0	0	0	0	0	0	0	0	0	0	0	0	0	0	0	0	0	0	0	0	0	0	0	0	1	44.782167	14.985567
2593		0	1	0	0	0	0	0	0	0	0	0	0	0	0	0	0	0	0	0	0	0	0	0	0	0	0	0	0	0	0	0	0	0	0	0	0	0	0	0	0	0	0	0	0	0	0	0	0	0	0	0	0	0	0	0	0	0	0	0	0	0	0	0	0	0	0	0	0	0	0	0	0	0	0	1	44.17295	16.5761
2594		0	1	0	0	0	0	0	0	0	0	0	0	0	0	0	0	0	0	0	0	0	0	0	0	0	0	0	0	0	0	0	0	0	0	0	0	0	0	0	0	0	0	0	0	0	0	0	0	0	0	0	0	0	0	0	0	0	0	0	0	0	0	0	0	0	0	0	0	0	0	0	0	0	0	1	44.276211	15.874985
2595		0	1	0	0	0	0	0	0	0	0	0	0	0	0	0	0	0	0	0	0	0	0	0	0	0	0	0	0	0	0	0	0	0	0	0	0	0	0	0	0	0	0	0	0	0	0	0	0	0	0	0	0	0	0	0	0	0	0	0	0	0	0	0	0	0	0	0	0	0	0	0	0	0	0	1	45.745363	14.46735
2596	DL90		0	0	0	0	0	0	0	0	0	0	0	0	0	0	0	0	0	0	0	0	0	0	0	0	0	0	0	0	0	0	0	0	0	0	0	0	0	0	1	0	0	0	0	0	0	0	0	0	0	0	0	0	0	0	0	0	0	0	0	0	0	0	0	0	0	0	0	0	0	0	0	0	0	1	43.289528	18.617778
2597		0	0	0	0	0	0	0	0	0	0	0	0	0	0	0	0	0	0	0	0	0	0	0	0	0	0	0	0	0	0	0	0	0	0	0	0	0	0	0	1	0	0	0	0	0	0	0	0	0	0	0	0	0	0	0	0	0	0	0	0	0	0	0	0	0	0	0	0	0	0	0	0	0	0	1	44.60935	22.274417
2598		0	0	0	0	0	0	0	0	0	0	0	0	0	0	0	0	0	0	0	0	0	0	0	0	0	0	0	0	0	0	0	0	1	0	0	0	0	0	0	0	0	0	0	0	0	0	0	0	0	0	0	0	0	0	0	0	0	0	0	0	0	0	0	0	0	0	0	0	0	0	0	0	0	0	1	37.640833	22.206611
2599		0	0	0	0	0	0	0	0	0	0	0	0	0	0	0	0	0	0	0	0	0	0	0	0	0	0	0	0	0	0	0	0	1	0	0	0	0	0	0	0	0	0	0	0	0	0	0	0	0	0	0	0	0	0	0	0	0	0	0	0	0	0	0	0	0	0	0	0	0	0	0	0	0	0	1	36.8871	22.67085
2600		0	0	0	0	0	0	0	0	0	0	0	0	0	0	0	0	0	0	0	0	0	0	0	0	0	0	0	0	0	0	0	0	1	0	0	0	0	0	0	0	0	0	0	0	0	0	0	0	0	0	0	0	0	0	0	0	0	0	0	0	0	0	0	0	0	0	0	0	0	0	0	0	0	0	1	36.804183	22.6181
2601		0	0	0	0	0	0	0	0	0	0	0	0	0	0	0	0	0	0	0	0	0	0	0	0	0	0	0	0	0	0	0	0	1	0	0	0	0	0	0	0	0	0	0	0	0	0	0	0	0	0	0	0	0	0	0	0	0	0	0	0	0	0	0	0	0	0	0	0	0	0	0	0	0	0	1	37.335467	22.644983
2602		0	0	0	0	0	0	0	0	0	0	0	0	0	0	0	0	0	0	0	0	0	0	0	0	0	0	0	0	0	0	0	0	1	0	0	0	0	0	0	0	0	0	0	0	0	0	0	0	0	0	0	0	0	0	0	0	0	0	0	0	0	0	0	0	0	0	0	0	0	0	0	0	0	0	1	37.094917	22.33285
2603		0	0	0	0	0	0	0	0	0	0	0	0	0	0	0	0	0	0	0	0	0	0	0	0	0	0	0	0	0	0	0	0	1	0	0	0	0	0	0	0	0	0	0	0	0	0	0	0	0	0	0	0	0	0	0	0	0	0	0	0	0	0	0	0	0	0	0	0	0	0	0	0	0	0	1	37.079183	22.291133
2604		0	0	0	0	0	0	0	0	0	0	0	0	0	0	0	0	0	0	0	0	0	0	0	0	0	0	0	0	0	0	0	0	1	0	0	0	0	0	0	0	0	0	0	0	0	0	0	0	0	0	0	0	0	0	0	0	0	0	0	0	0	0	0	0	0	0	0	0	0	0	0	0	0	0	1	38.084983	22.033933
2605		0	1	0	0	0	0	0	0	0	0	0	0	0	0	0	0	0	0	0	0	0	0	0	0	0	0	0	0	0	0	0	0	1	0	0	0	0	0	0	0	0	0	0	0	0	0	0	0	0	0	0	0	0	0	0	0	0	0	0	0	0	0	0	0	0	0	0	0	0	0	0	0	0	0	1	37.243117	21.82745
2606		0	0	0	0	0	0	0	0	0	0	0	0	0	0	0	0	0	0	0	0	0	0	0	0	0	0	0	0	0	0	0	0	1	0	0	0	0	0	0	0	0	0	0	0	0	0	0	0	0	0	0	0	0	0	0	0	0	0	0	0	0	0	0	0	0	0	0	0	0	0	0	0	0	0	1	37.539633	22.046467
2607		0	0	0	0	0	0	0	0	0	0	0	0	0	0	0	0	0	0	0	0	0	0	0	0	0	0	0	0	0	0	0	0	1	0	0	0	0	0	0	0	0	0	0	0	0	0	0	0	0	0	0	0	0	0	0	0	0	0	0	0	0	0	0	0	0	0	0	0	0	0	0	0	0	0	1	37.533667	22.053183
2608		0	0	0	0	0	0	0	0	0	0	0	0	0	0	0	0	0	0	0	0	0	0	0	0	0	0	0	0	0	0	0	0	1	0	0	0	0	0	0	0	0	0	0	0	0	0	0	0	0	0	0	0	0	0	0	0	0	0	0	0	0	0	0	0	0	0	0	0	0	0	0	0	0	0	1	37.622183	22.050617
2609		0	0	0	0	0	0	0	0	0	0	0	0	0	0	0	0	0	0	0	0	0	0	0	0	0	0	0	0	0	0	0	0	1	0	0	0	0	0	0	0	0	0	0	0	0	0	0	0	0	0	0	0	0	0	0	0	0	0	0	0	0	0	0	0	0	0	0	0	0	0	0	0	0	0	1	37.9338	22.211783
2610		0	0	0	0	0	0	0	0	0	0	0	0	0	0	0	0	0	0	0	0	0	0	0	0	0	0	0	0	0	0	0	0	1	0	0	0	0	0	0	0	0	0	0	0	0	0	0	0	0	0	0	0	0	0	0	0	0	0	0	0	0	0	0	0	0	0	0	0	0	0	0	0	0	0	2	37.942383	22.23445
2611		0	1	0	0	0	0	0	0	0	0	0	0	0	0	0	0	0	0	0	0	0	0	0	0	0	0	0	0	0	0	0	0	1	0	0	0	0	0	0	0	0	0	0	0	0	0	0	0	0	0	0	0	0	0	0	0	0	0	0	0	0	0	0	0	0	0	0	0	0	0	0	0	0	0	1	37.9337	22.166183
2612		0	0	0	0	0	0	0	0	0	0	0	0	0	0	0	0	0	0	0	0	0	0	0	0	0	0	0	0	0	0	0	0	1	0	0	0	0	0	0	0	0	0	0	0	0	0	0	0	0	0	0	0	0	0	0	0	0	0	0	0	0	0	0	0	0	0	0	0	0	0	0	0	0	0	1	38.36505	22.128117
2613		0	0	0	0	0	0	0	0	0	0	0	0	0	0	0	0	0	0	0	0	0	0	0	0	0	0	0	0	0	0	0	0	1	0	0	0	0	0	0	0	0	0	0	0	0	0	0	0	0	0	0	0	0	0	0	0	0	0	0	0	0	0	0	0	0	0	0	0	0	0	0	0	0	0	1	38.347367	22.3141
2614		0	1	0	0	0	0	0	0	0	0	0	0	0	0	0	0	0	0	0	0	0	0	0	0	0	0	0	0	0	0	0	0	1	0	0	0	0	0	0	0	0	0	0	0	0	0	0	0	0	0	0	0	0	0	0	0	0	0	0	0	0	0	0	0	0	0	0	0	0	0	0	0	0	0	1	38.563783	22.348983
2615		0	1	0	0	0	0	0	0	0	0	0	0	0	0	0	0	0	0	0	0	0	0	0	0	0	0	0	0	0	0	0	0	1	0	0	0	0	0	0	0	0	0	0	0	0	0	0	0	0	0	0	0	0	0	0	0	0	0	0	0	0	0	0	0	0	0	0	0	0	0	0	0	0	0	1	38.600117	22.326617
2616		0	1	0	0	0	0	0	0	0	0	0	0	0	0	0	0	0	0	0	0	0	0	0	0	0	0	0	0	0	0	0	0	0	0	0	0	0	0	0	0	0	0	0	0	0	0	0	0	0	0	0	0	0	0	0	0	0	0	0	0	0	0	0	0	0	0	0	0	0	0	0	0	0	0	1	38.420278	22.242222
2617		0	1	0	0	0	0	0	0	0	0	0	0	0	0	0	0	0	0	0	0	0	0	0	0	0	0	0	0	0	0	0	0	0	0	0	0	0	0	0	0	0	0	0	0	0	0	0	0	0	0	0	0	0	0	0	0	0	0	0	0	0	0	0	0	0	0	0	0	0	0	0	0	0	0	1	38.624722	23.844444
2618		0	1	0	0	0	0	0	0	0	0	0	0	0	0	0	0	0	0	0	0	0	0	0	0	0	0	0	0	0	0	0	0	0	0	0	0	0	0	0	0	0	0	0	0	0	0	0	0	0	0	0	0	0	0	0	0	0	0	0	0	0	0	0	0	0	0	0	0	0	0	0	0	0	0	1	37.186667	21.913889
2619		0	1	0	0	0	0	0	0	0	0	0	0	0	0	0	0	0	0	0	0	0	0	0	0	0	0	0	0	0	0	0	0	0	0	0	0	0	0	0	0	0	0	0	0	0	0	0	0	0	0	0	0	0	0	0	0	0	0	0	0	0	0	0	0	0	0	0	0	0	0	0	0	0	0	1	39.967778	20.665278
2620		0	1	0	0	0	0	0	0	0	0	0	0	0	0	0	0	0	0	0	0	0	0	0	0	0	0	0	0	0	0	0	0	0	0	0	0	0	0	0	0	0	0	0	0	0	0	0	0	0	0	0	0	0	0	0	0	0	0	0	0	0	0	0	0	0	0	0	0	0	0	0	0	0	0	1	38.398778	22.005556
2621		0	1	0	0	0	0	1	0	0	0	0	0	0	1	0	0	0	0	0	1	0	0	0	0	0	0	0	0	0	0	1	0	0	0	0	0	0	0	0	1	0	0	0	0	0	0	0	0	0	0	0	0	0	0	0	0	0	0	0	0	0	0	0	0	0	0	0	0	0	0	0	0	0	0	1	44.101144	21.469257
2622		0	0	0	0	0	0	0	0	0	0	0	0	0	0	0	0	0	0	0	1	0	0	0	0	0	0	0	0	0	0	1	0	0	0	0	0	0	0	0	1	0	0	0	0	0	0	0	0	0	0	0	0	0	0	0	0	0	0	0	0	0	0	0	0	0	0	0	0	0	0	0	0	0	0	1	43.974361	21.515333
2623		0	1	0	0	0	0	0	0	0	0	0	0	0	1	0	0	0	0	0	1	0	0	0	0	0	1	0	0	0	0	1	0	0	0	0	0	0	0	0	1	0	0	0	0	0	0	0	0	0	0	0	0	0	0	0	0	0	0	0	0	0	0	0	0	0	0	0	0	0	0	0	0	0	0	1	44.028972	21.957889
2624		0	0	0	0	0	0	0	0	0	0	0	0	0	1	0	0	0	0	0	1	0	0	0	1	0	0	0	0	0	0	1	0	0	0	1	0	0	0	0	0	0	1	0	0	0	0	0	0	0	0	0	0	0	0	0	0	0	0	0	0	0	0	0	0	0	0	0	0	0	0	0	0	0	0	1	43.281361	22.062111
2625		0	0	0	0	0	0	0	0	0	0	0	0	0	1	0	0	0	0	0	1	0	0	0	0	0	0	0	0	0	0	1	0	0	0	0	0	0	0	0	0	0	0	0	0	0	0	0	0	0	0	0	0	0	0	0	0	0	0	0	0	0	0	0	0	0	0	0	0	0	0	0	0	0	0	1	43.795306	21.9035
2626		0	0	0	0	0	0	1	0	0	0	0	0	0	0	0	0	0	0	0	0	0	0	0	0	0	0	0	0	0	0	0	0	0	0	0	0	0	0	0	0	0	0	0	0	0	0	0	0	0	0	0	0	0	0	0	0	0	0	0	0	0	0	0	0	0	0	0	0	0	0	0	0	0	0	2	41.80347	21.300001
2627		0	1	0	0	0	0	0	0	0	0	0	0	0	1	0	0	0	0	0	1	0	0	0	0	0	0	0	0	0	0	0	0	0	0	0	0	0	0	0	0	0	1	0	0	0	0	0	0	0	0	0	0	0	0	0	0	0	0	0	0	0	0	0	0	0	0	0	0	0	0	0	0	0	0	1	43.020667	22.364306
2628		0	1	0	0	0	0	0	0	0	0	0	0	0	1	0	0	0	0	0	1	0	0	0	1	0	0	0	0	0	0	0	0	0	0	0	0	0	0	0	0	0	1	0	0	0	0	0	0	0	0	0	0	0	0	0	0	0	0	0	0	0	0	0	0	0	0	0	0	0	0	0	0	0	0	1	43.030389	22.388806
2629		0	0	0	0	0	0	0	0	0	0	1	0	0	0	0	0	0	0	0	1	0	0	0	0	0	0	0	0	0	0	0	0	0	0	0	0	0	0	0	0	0	1	0	0	0	0	0	0	0	0	0	0	0	0	0	0	0	0	0	0	0	0	0	0	0	0	0	0	0	0	0	0	0	0	1	43.429222	22.4175
2630		0	0	0	0	0	0	1	0	0	0	0	0	0	0	1	0	0	0	0	1	0	0	0	0	0	0	0	0	0	0	1	0	0	0	0	0	0	0	0	0	0	1	0	0	0	0	0	0	0	0	0	0	0	0	0	0	0	0	0	0	0	0	0	0	0	0	0	0	0	0	0	0	0	0	1	43.536528	22.285278
2631		0	0	0	0	0	0	0	0	0	0	0	0	0	0	0	0	0	0	0	1	0	0	0	0	0	0	0	0	0	0	0	0	0	0	0	0	0	0	0	0	0	0	0	0	0	0	0	0	0	0	0	0	0	0	0	0	0	0	0	0	0	0	0	0	0	0	0	0	0	0	0	0	0	0	1	43.760471	22.310321
2632		0	0	0	0	0	0	1	0	0	0	0	0	0	1	0	0	0	0	0	0	0	0	0	0	0	0	0	0	0	0	0	0	0	0	0	0	0	0	0	0	0	0	0	0	0	0	0	0	0	0	0	0	0	0	0	0	0	0	0	0	0	0	0	0	0	0	0	0	0	0	0	0	0	0	1	43.834444	21.9545
2633		0	1	0	0	0	0	1	0	0	1	0	0	0	1	0	0	0	0	1	1	0	0	0	1	0	1	1	0	0	0	1	0	0	0	0	0	0	0	0	0	0	1	0	0	0	0	0	0	0	0	0	0	0	0	0	0	0	0	0	0	0	0	0	0	0	0	0	0	0	0	0	0	0	0	3	44.382417	22.341694
2634		0	1	0	0	0	0	0	0	0	1	1	0	0	0	0	0	0	0	1	1	0	0	0	0	0	1	1	0	0	0	1	0	0	0	0	0	0	0	0	0	0	0	0	0	0	0	0	0	0	0	0	0	0	0	0	0	0	0	0	0	0	0	0	0	0	0	0	0	0	0	0	0	0	0	1	44.292306	22.277583
2635		0	1	0	0	0	0	1	0	0	1	1	0	0	1	0	0	1	1	1	1	0	1	1	1	0	1	1	0	0	0	1	0	0	0	0	0	0	0	0	0	0	0	0	0	0	0	0	0	0	0	0	0	0	0	0	0	0	0	0	0	0	0	0	0	0	0	0	0	0	0	0	0	0	0	1	44.185727	22.154682
2636		0	1	0	0	0	0	0	0	0	1	1	0	0	1	0	1	1	1	1	1	0	1	1	1	0	1	1	0	0	0	1	0	0	0	0	0	1	0	0	1	0	0	0	0	0	0	0	0	0	0	0	0	0	0	0	0	0	0	0	0	0	0	0	0	0	0	0	0	0	0	0	0	0	0	1	44.61375	22.275222
2637		0	0	0	0	0	0	0	0	0	0	0	0	0	1	0	0	0	0	0	0	0	1	0	0	0	0	0	0	0	0	1	0	0	0	0	0	0	0	0	0	0	0	0	0	0	0	0	0	0	0	0	0	0	0	0	0	0	0	0	0	0	0	0	0	0	0	0	0	0	0	0	0	0	0	3	44.441475	21.953446
