# Supplementary material for: Range‐constrained co‐occurrence simulation reveals little niche partitioning among rock‐dwelling Montenegrina land snails (Gastropoda: Clausiliidae)
Source: J Biogeogr. 2018 Apr 16;45(6):1444–57. doi: 10.1111/jbi.13220 (PMC6027963; doi:10.1111/jbi.13220)
Supplement: Supplementary file 2 [file JBI-45-1444-s002.pdf]

Journal of Biogeography

SUPPORTING INFORMATION

Fehér Z, Mason K, Szekeres M, Haring E, Bamberger S, Páll-Gergely B, Sólymos P: Range-constrained co-occurrence simulation reveals little niche partitioning among rock-dwelling *Montenegrina* land snails (Gastropoda: Clausiliidae). DOI: 10.1111/jbi.13220

Appendix S2. RaCoCOS workflow

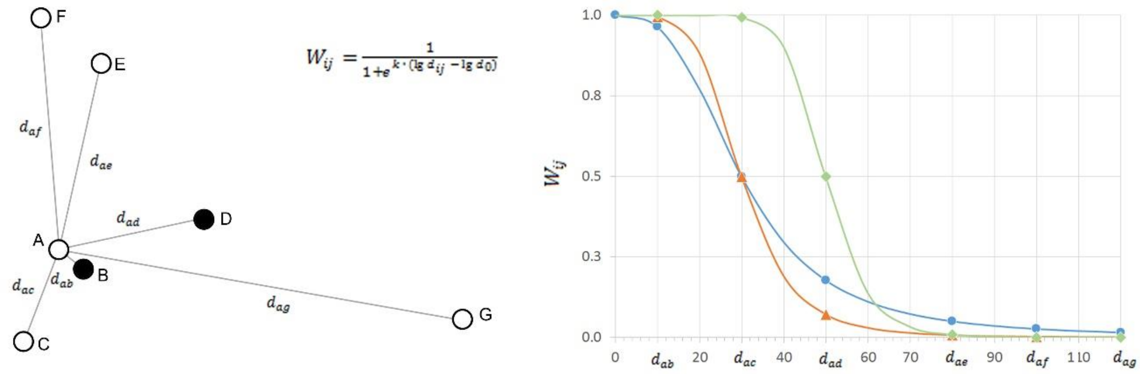

site	p/a	distance from A	weight (W_{aj}) in OP_a formula under different k/d_0 settings				Y_j	$W_{ij} \times Y_j$		
				k=3/d_0=30	k=5/d_0=30	k=10/d_0=50		k=3/d_0=30	k=5/d_0=30	k=10/d_0=50
A	absent	d_{aa} = 0 km	W_{aa} =	1.000	1.000	1.000	$Y_a = 0$	0	0	0
B	present	d_{ab} = 10 km	W_{ab} =	0.964	0.996	1.000	$Y_b = 1$	0.964	0.996	1.000
C	absent	d_{ac} = 30 km	W_{ac} =	0.500	0.500	0.994	$Y_c = 0$	0	0	0
D	present	d_{ad} = 50 km	W_{ad} =	0.178	0.072	0.500	$Y_d = 1$	0.178	0.072	0.500
E	absent	d_{ae} = 80 km	W_{ae} =	0.050	0.007	0.009	$Y_e = 0$	0	0	0
F	absent	d_{af} = 100 km	W_{af} =	0.026	0.002	0.001	$Y_f = 0$	0	0	0
G	absent	d_{ag} = 120 km	W_{ag} =	0.015	0.001	0.000	$Y_g = 0$	0	0	0
			ΣW_{aj} =	2.733	2.578	3.504	$\Sigma(W_{aj} \times Y_j)$ =	1.142	1.068	1.500
			$OP_a = \frac{\sum(W_{aj} \times Y_j)}{\sum W_{aj}} = \frac{0 + W_{ab} + 0 + W_{ad} + 0 + 0 + 0}{W_{aa} + W_{ab} + \cdots + W_{af} + W_{ag}}$				OP_a =	0.418	0.414	0.428

Figure S2.2. An example to illustrate how the occurrence probability of a given taxon at a given site (site A) is calculated. In this simple example, there are only six further sites (B to G), surrounding site A at different distances (d_{ab} to d_{ag}). Of the seven sites, the taxon in question is present at two (B and D) and absent at five (A, C, E, F and G) sites. In the occurrence probability calculation all sites weight differently depending on their distances from the site A, and on the selection of k and d_0 parameters of eq. 1. The occurrence probability of the given taxon at site A (OP_a) is calculated according to eq. 2, where Y_j is a binary value that takes 1 or 0, depending on whether the taxon is present or absent on the sites A to G.

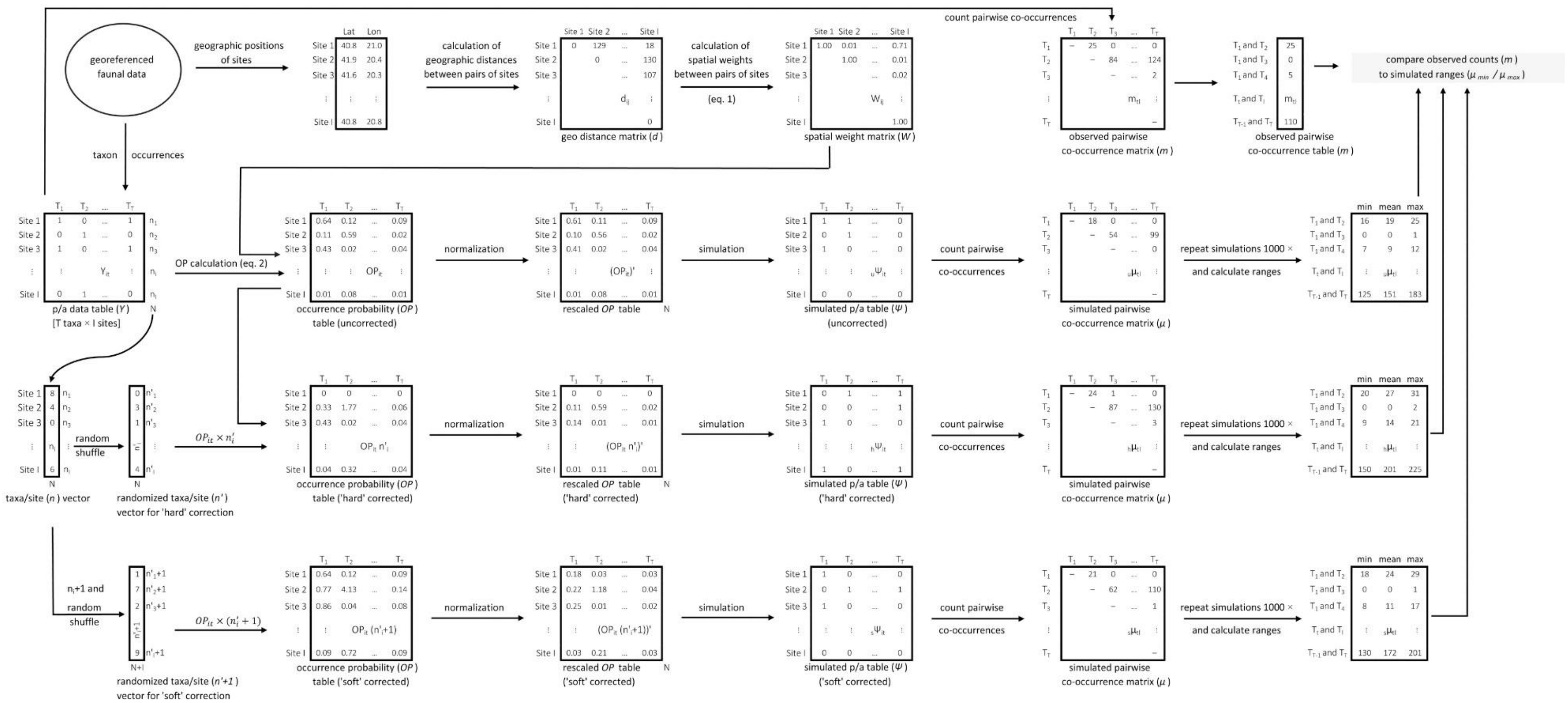

Figure S2.3. Workflow of the co-occurrence simulation.

The calculation of the *W* matrix initiates from the geographic positions of the collecting sites (see Fig. S2.2 in more details). Simulation initiates from the observed presence–absence values, which are arranged in a binary matrix (*Y*) of I rows representing sites and T columns representing taxa. Sums of rows give the number of taxa per each site (n) and the total sum (N) is equal to the number of presence records in the data table. Depending on the spatial distribution of a given taxon and on the geographical distances between sites we calculated the probability of occurrence (*OP*) of each taxon at each site (see equations 1 and 2 in the text). In the 'uncorrected' model, the raw *OP* values were rescaled resulting in a matrix, the total sum of which is equal to N. During the 'hard correction' we created a correction vector of I elements by randomly shuffling the number of taxa per site values. Each element in a row of the *OP* matrix was multiplied by the value in the corresponding row of this correction vector (i.e. *OP* values of a site were multiplied by the taxon count of another site). Normalization and simulation was done in the same way as for the uncorrected model. During the 'soft' correction, to avoid eventual zero values in the correction vector, 1 was added to each of its elements.). Based on the rescaled *OP'* matrices, occurrence data tables (denoted as ${}_u\Psi$, ${}_h\Psi$ and ${}_s\Psi$ for the uncorrected, the 'hard' corrected and the 'soft' corrected simulations) were simulated based on unequal probabilities of selection. Simulated co-occurrences (μ) were calculated from Ψ matrices as the number of sites where both taxa were present. These steps of the distribution simulation and co-ocurrence calculation were repeated 1000 times and minimum, maximum and mean values were calculated for each of the simulated pairwise co-occurrences.
