# Supplementary material for: Range‐constrained co‐occurrence simulation reveals little niche partitioning among rock‐dwelling Montenegrina land snails (Gastropoda: Clausiliidae)
Source: J Biogeogr. 2018 Apr 16;45(6):1444–57. doi: 10.1111/jbi.13220 (PMC6027963; doi:10.1111/jbi.13220)
Supplement: Supplementary file 3 [file JBI-45-1444-s003.pdf]

Journal of Biogeography

SUPPORTING INFORMATION

Fehér Z, Mason K, Szekeres M, Haring E, Bamberger S, Páll-Gergely B, Sólymos P: Range-constrained co-occurrence simulation reveals little niche partitioning among rock-dwelling *Montenegrina* land snails (Gastropoda: Clausiliidae). DOI: 10.1111/jbi.13220

Appendix S3. Simulated vs. observed co-occurrences.

Figure S3.4. Pairwise co-occurrences of the studied taxa. Observed pairwise co-occurrences are compared to those simulated by 'hard' model correction with $k = 5$ and $d_0 = 30$ km settings. Dark grey (brown) and mid-grey (blue) indicate when observed pairwise values are lower or higher than the simulated range, stars indicate when the observed zero values fall within the simulated range. The results of this as well as the other eight model settings are summarized in Table 1., detailed values of taxon pairs formed by *Montenegrina* with the other taxa are given in Table S3.10).

Table S3.7. Observed co-occurrences as well as the mean values and ranges (min–max) of 1000 co-occurrence simulations among *Montenegrina* and 46 other landsnail taxa. Simulations were performed under nine different combinations of model corrections and smoothing parameter settings. When observed values are lower or higher than the range of the simulated values it is indicated by * and ‡, respectively.

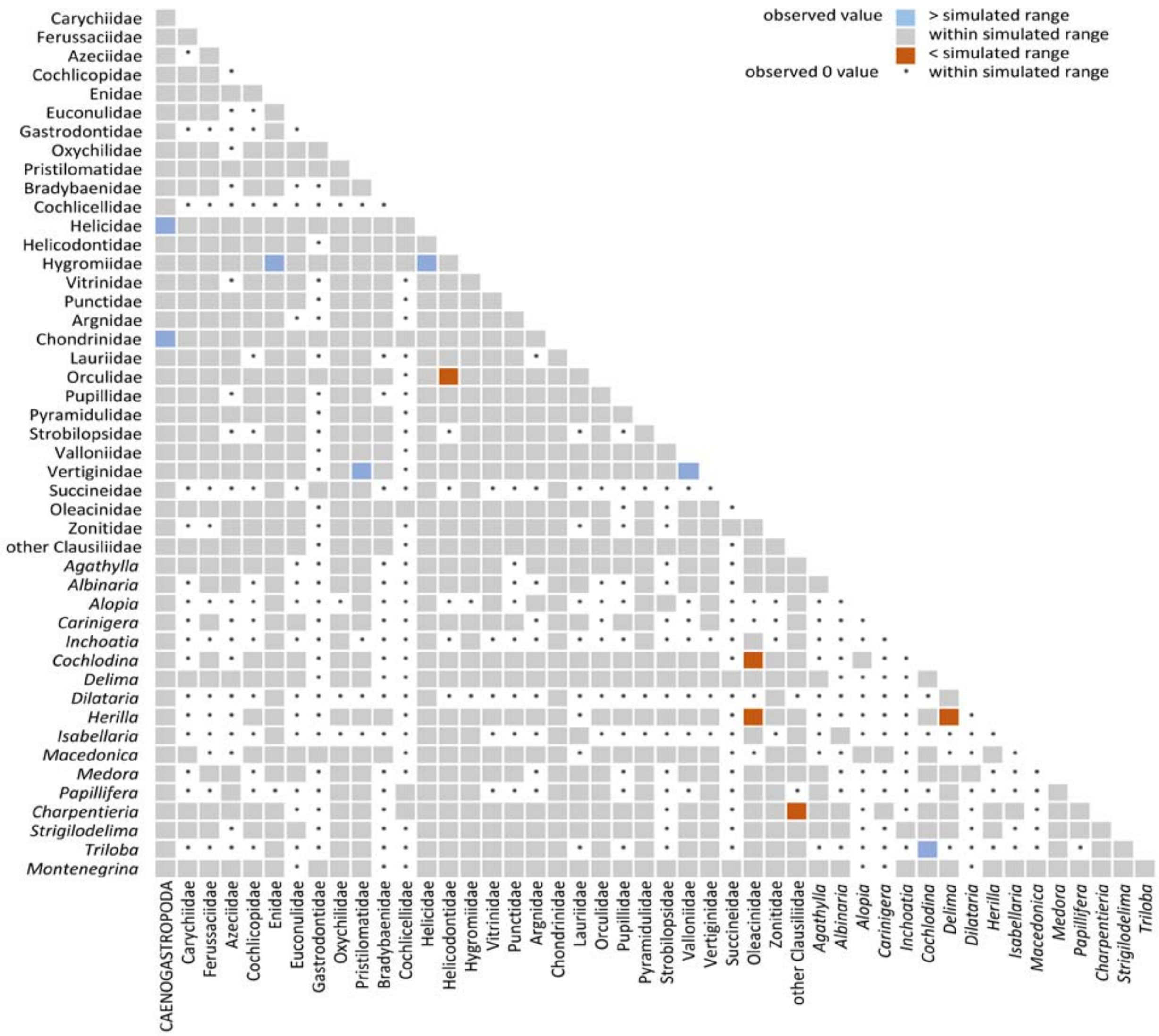
observed value
> simulated range
within simulated range
< simulated range
observed 0 value
within simulated range
Carychiidae
Ferussaciidae
Azeciidae
Cochlicopidae
Enidae
Euconulidae
Gastrodontidae
Oxychilidae
Pristilomatidae
Bradybaenidae
Cochlicellidae
Helicidae
Helicodontidae
Hygromiidae
Vitrinidae
Punctidae
Argnidae
Chondrinidae
Lauriidae
Orculidae
Pupillidae
Pyramidulidae
Strobilopsidae
Valloniidae
Vertiginidae
Succineidae
Oleacinidae
Zonitidae
other Clausiliidae
Agathylla
Albinaria
Alopia
Carinigera
Inchoatia
Cochlodina
Delima
Dilataria
Herilla
Isabellaria
Macedonica
Medora
Papillifera
Charpentieria
Strigilodelima
Triloba
Montenegrina
CAENOGASTROPODA

			observed values	simulated values																	
				uncorr, k=3, d_0=30		uncorr, k=5, d_0=30		uncorr, k=10, d_0=50		'soft', k=3, d_0=30		'soft', k=5, d_0=30		'soft', k=10, d_0=50		'hard', k=3, d_0=30		'hard', k=5, d_0=30		'hard', k=10, d_0=50	
				mean	(min–max)	mean	(min–max)	mean	(min–max)	mean	(min–max)	mean	(min–max)	mean	(min–max)	mean	(min–max)	mean	(min–max)	mean	(min–max)
Caenogastropoda			193	169.5	(134–207)	162.4	(132–204)	166.1	(129–208)	191.7	(151–235)	187.7	(147–221)	186.6	(152–228)	204.8	(166–237)	194.4	(160–235)	198.3	(168–233)
Pulmonata		Carychiidae	1	1.6	(0–7)	1.4	(0–7)	1.6	(0–7)	2.4	(0–8)	2.2	(0–9)	2.3	(0–9)	2.7	(0–8)	2.3	(0–8)	2.6	(0–9)
		Ferussaciidae	5	6.5	(0–17)	5.9	(0–15)	6.1	(0–15)	8.4	(1–20)	8.4	(1–18)	8.4	(0–19)	9.9	(3–26)	8.9	(1–20)	9.7	(2–21)
		Azeciidae	9	7.7	(1–19)	7.3	(1–17)	6.6	(0–15)	10.2	(2–23)	8.7	(1–19)	8.8	(0–19)	11.6	(3–25)	10.5	(3–20)	10	(1–23)
		Cochlicopidae	3	2.5	(0–9)	2	(0–9)	2.3	(0–8)	3.6	(0–12)	3.2	(0–9)	3.1	(0–10)	4.3	(0–12)	3.3	(0–10)	3.7	(0–13)
		Enidae	174	143.3	(111–180)	144	(115–173)‡	142.1	(105–181)	165.1	(131–198)	161.9	(131–203)	162.7	(131–201)	178	(144–218)	175.3	(140–209)	174.2	(136–204)
		Euconulidae	0	1	(0–7)	0.8	(0–5)	0.8	(0–5)	1.4	(0–5)	1.1	(0–5)	1	(0–6)	1.6	(0–7)	1.4	(0–6)	1.3	(0–6)
		Gastrodontidae	1	0.7	(0–4)	0.8	(0–6)	0.8	(0–5)	0.9	(0–5)	0.9	(0–7)	0.9	(0–5)	1	(0–5)	1	(0–6)	1.1	(0–4)
		Oxychilidae	104	86.5	(64–115)	85	(60–116)	86.3	(59–112)	103.7	(77–139)	103.9	(79–133)	103.5	(77–135)	115.4	(81–144)	111.5	(87–146)	113.6	(85–140)
		Pristilomatidae	71	68.1	(45–96)	66.3	(42–94)	67.4	(47–90)	84.4	(53–113)	84.1	(61–110)	84.2	(59–115)	94.4	(69–119)	91.7	(68–118)	93.8	(67–119)
		Bradybaenidae	0	0.3	(0–3)	0	(0–1)	0	(0–2)	0.4	(0–4)	0	(0–1)	0	(0–1)	0.5	(0–4)	0.1	(0–2)	0	(0–1)
		Cochlicellidae	0	0.7	(0–5)	0.4	(0–3)	0.5	(0–4)	1.1	(0–6)	0.8	(0–6)	0.8	(0–5)	1.2	(0–6)	0.6	(0–4)	0.9	(0–7)
		Helicidae	178	143.9	(102–180)	139	(92–172)‡	138.4	(104–173)‡	166.8	(130–204)	161.2	(125–198)	161.4	(128–205)	181	(145–217)	172.3	(141–206)	174	(143–209)
		Helicodontidae	95	76.8	(54–113)	77.8	(55–104)	74.6	(44–104)	93.4	(70–134)	89.9	(66–124)	90.6	(59–121)	102.9	(76–134)	102.2	(74–131)	99.5	(69–124)
		Hygromiidae	176	145.6	(106–178)	143.6	(113–173)‡	142.7	(111–183)	168.2	(135–205)	164.6	(133–200)	165.3	(131–203)	181.4	(150–214)	176.9	(142–224)	177.5	(145–207)
		Vitrinidae	39	35.2	(15–51)	35.3	(17–54)	34.1	(15–54)	44.3	(25–65)	42.6	(25–65)	42.8	(26–66)	49.8	(32–76)	49.3	(30–74)	47.8	(27–69)
		Punctidae	6	7.5	(0–18)	7.3	(1–18)	6.9	(1–18)	10.2	(2–21)	9.2	(2–18)	9.4	(1–19)	11.6	(3–22)	10.8	(3–22)	10.4	(2–26)
		Argnidae	9	9.9	(1–23)	9.2	(1–18)	9.5	(2–21)	13.2	(3–25)	12.9	(3–27)	12.8	(3–28)	15.2	(4–30)	14.2	(3–27)	14.8	(4–30)
		Chondrinidae	156	144.4	(112–179)	138.8	(106–171)	140.8	(112–182)	167.5	(133–204)	162	(130–194)	162.2	(131–202)	180.9	(148–215)	171.5	(142–207)	174.2	(133–205)
		Lauriidae	5	5.5	(0–15)	5.2	(0–12)	4.9	(0–15)	7.3	(0–17)	6.8	(1–16)	6.7	(1–16)	8.5	(1–17)	7.7	(1–20)	7.6	(0–19)
		Orculidae	35	39.8	(23–61)	39.5	(22–63)	38.4	(18–56)	50.8	(31–72)	49.2	(30–73)	49.2	(32–71)	57.5	(35–81)	56.3	(32–80)	55.3	(34–80)
		Pupillidae	5	3.4	(0–10)	3	(0–10)	3.3	(0–11)	4.6	(0–12)	4.3	(0–15)	4.2	(0–11)	5.2	(0–14)	4.7	(0–14)	4.7	(0–13)
		Pyramidulidae	61	58.5	(35–80)	57.6	(35–90)	60.1	(35–84)	72.8	(51–100)	73.9	(52–99)	73.8	(51–98)	81.1	(56–104)	79.4	(55–106)	82.1	(59–108)
		Strobilopsidae	1	2.1	(0–9)	1.9	(0–8)	1.9	(0–7)	2.9	(0–10)	2.7	(0–8)	2.7	(0–8)	3.2	(0–9)	2.9	(0–10)	3.1	(0–10)
		Valloniidae	9	8.8	(1–19)	8.1	(1–18)	8.4	(1–21)	11.6	(3–25)	11.1	(3–25)	11.2	(2–24)	13.5	(4–29)	12.1	(2–24)	12.7	(4–26)
		Vertiginidae	15	19.1	(7–34)	18.5	(6–33)	18.6	(5–33)	25.3	(11–42)	24.4	(9–41)	24.4	(12–39)	28.5	(14–47)	27.2	(9–45)	27.1	(13–46)
		Succineidae	4	2.5	(0–9)	2.8	(0–8)	2.9	(0–9)	3.2	(0–12)	3.8	(0–11)	3.9	(0–13)	3.8	(0–10)	4.1	(0–14)	4.4	(0–12)
		Oleacinidae	92	74.4	(49–100)	72.8	(45–104)	73.9	(51–98)	91.7	(65–122)	90.3	(60–114)	90.2	(66–120)	101.5	(72–130)	97.3	(69–123)	99.7	(73–129)
		Zonitidae	44	42.6	(23–61)	42.2	(26–63)	43.3	(24–70)	54.1	(30–77)	54.4	(33–78)	54.8	(36–79)	61.2	(37–82)	59	(38–80)	60.9	(41–88)
Clausiliidae		other Clausiliidae	54	48.6	(29–71)	43.5	(27–62)	43.3	(25–61)	59.7	(40–86)	53.3	(35–75)	53.1	(34–76)	66.8	(44–92)	57.8	(36–81)	58.7	(33–80)
	Alopiinae	*Agathylla*	24	26.9	(13–48)	24.1	(9–42)	26.5	(13–45)	34.7	(18–50)	34.2	(18–51)	34.2	(18–55)	39.3	(17–60)	34.8	(18–53)	38.7	(21–59)
		Albinaria	13	16.5	(6–31)	14.3	(2–27)	15.9	(5–29)	20.9	(8–37)	20.1	(4–34)	19.8	(9–34)	23.4	(10–37)	20	(8–33)	22.4	(11–40)
		Alopia	0	0.2	(0–3)	0	(0–0)	0	(0–0)	0.3	(0–3)	0	(0–0)	0	(0–0)	0.3	(0–3)	0	(0–1)	0	(0–0)
		Carinigera	0	3.3	(0–10)	1.7	(0–7)	1.8	(0–6)	4.3	(0–14)	2.2	(0–7)	2.2	(0–7)	4.9	(0–13)	2.3	(0–7)	2.4	(0–8)
		Inchoatia	1	0.9	(0–4)	0.9	(0–5)	1	(0–5)	1.1	(0–5)	1.3	(0–6)	1.2	(0–10)	1.3	(0–6)	1.3	(0–6)	1.4	(0–7)
		Cochlodina	9	13.5	(4–27)	10.4	(1–22)	11.7	(2–26)	17.9	(7–32)	15.2	(5–28)	15.2	(2–27)	20.8	(7–35)	15.6	(5–29)	17.6	(4–31)
		Delima	55	53.4	(35–77)	48.7	(29–77)	50.8	(33–69)	64	(40–90)	59.7	(34–83)	60.5	(42–86)	70.5	(49–92)	62.3	(39–82)	65.6	(40–89)
		Dilataria	0	0	(0–1)	0	(0–0)	0	(0–0)	0	(0–2)	0	(0–0)	0	(0–0)	0	(0–1)	0	(0–0)	0	(0–0)
		Herilla	4	13.9	(2–27)	8.1	(1–20)	9.6	(1–20)	18.5	(8–33)*	12.8	(4–26)	12.8	(4–24)	21.6	(9–35)*	12.7	(4–24)	15	(5–28)*
		Isabellaria	2	1.5	(0–7)	1.4	(0–8)	1.2	(0–6)	1.7	(0–7)	1.6	(0–6)	1.6	(0–7)	2.1	(0–8)	2.1	(0–7)	1.7	(0–7)
		Macedonica	2	2.4	(0–11)	1	(0–5)	1.1	(0–5)	3.1	(0–11)	1.6	(0–6)	1.5	(0–8)	3.5	(0–10)	1.5	(0–6)	1.8	(0–9)
		Medora	7	11.6	(2–24)	10.6	(2–22)	10.9	(2–21)	15	(3–28)	14.4	(2–29)	14.3	(4–26)	17.3	(7–33)	15.7	(6–29)	16	(5–28)
		Papillifera	1	1.3	(0–6)	1	(0–5)	1	(0–6)	1.8	(0–7)	1.4	(0–6)	1.4	(0–5)	2.2	(0–8)	1.5	(0–8)	1.7	(0–8)
		Charpentieria	40	41.9	(22–63)	40.4	(20–65)	41.6	(23–59)	52.8	(33–78)	53.1	(34–77)	53	(36–72)	59.3	(34–81)	56.9	(35–80)	59	(38–82)
		Strigilodelima	89	90	(63–116)	92	(68–118)	91.5	(62–116)	104.8	(77–135)	106.4	(78–133)	106.9	(80–138)	114.1	(86–146)	114.2	(85–145)	114.8	(87–140)
		Triloba	12	14.1	(4–26)	14.8	(5–28)	14.7	(2–27)	18.1	(7–34)	18.7	(7–31)	18.8	(8–32)	20.9	(9–40)	21.6	(10–37)	20.9	(7–37)
